# Strengthening women's empowerment and gender equality in fragile contexts towards peaceful and inclusive societies: A systematic review and meta‐analysis

**DOI:** 10.1002/cl2.1214

**Published:** 2022-03-08

**Authors:** Etienne Lwamba, Shannon Shisler, Will Ridlehoover, Meital Kupfer, Nkululeko Tshabalala, Promise Nduku, Laurenz Langer, Sean Grant, Ada Sonnenfeld, Daniela Anda, John Eyers, Birte Snilstveit

**Affiliations:** ^1^ International Initiative for Impact Evaluation (3ie) London UK; ^2^ Africa Centre for Evidence University of Johannesburg South Africa; ^3^ Richard M. Fairbanks School of Public Health Indiana University Indianapolis Indiana

## Abstract

**Background:**

Across the globe, gender disparities still exist with regard to equitable access to resources, participation in decision‐making processes, and gender and sexual‐based violence. This is particularly true in fragile and conflict‐affected settings, where women and girls are affected by both fragility and conflict in unique ways. While women have been acknowledged as key actors in peace processes and post‐conflict reconstruction (e.g., through the United Nations Security Council Resolution 1325 and the Women, Peace and Security Agenda) evidence on the effectiveness of gender‐specific and gender‐transformative interventions to improve women's empowerment in fragile and conflict‐affected states and situations (FCAS) remains understudied.

**Objectives:**

The purpose of this review was to synthesize the body of evidence around gender‐specific and gender‐transformative interventions aimed at improving women's empowerment in fragile and conflict‐affected settings with high levels of gender inequality. We also aimed to identify barriers and facilitators that could affect the effectiveness of these interventions and to provide implications for policy, practice and research designs within the field of transitional aid.

**Methods:**

We searched for and screened over 100,000 experimental and quasi‐experimental studies focused on FCAS at the individual and community levels. We used standard methodological procedures outlined by the Campbell Collaboration for the data collection and analysis, including quantitative and qualitative analyses, and completed the Grading of Recommendations, Assessment, Development and Evaluations (GRADE) methodology to assess the certainty around each body of evidence.

**Results:**

We identified 104 impact evaluations (75% randomised controlled trials) assessing the effects of 14 different types of interventions in FCAS. About 28% of included studies were assessed as having a high risk of bias (45% among quasi‐experimental designs). Interventions supporting women's empowerment and gender equality in FCAS produced positive effects on the outcomes related to the primary focus of the intervention. There are no significant negative effects of any included interventions. However, we observe smaller effects on behavioural outcomes further along the causal chain of empowerment. Qualitative syntheses indicated that gender norms and practices are potential barriers to intervention effectiveness, while working with local powers and institutions can facilitate the uptake and legitimacy of interventions.

**Conclusions:**

We observe gaps of rigorous evidence in certain regions (notably MENA and Latin America) and in interventions specifically targeting women as actors of peacebuilding. Gender norms and practices are important elements to consider in programme design and implementation to maximise potential benefits: focusing on empowerment only might not be enough in the absence of targeting the restrictive gender norms and practices that may undermine intervention effectiveness. Lastly, programme designers and implementation should consider explicitly targeting specific empowerment outcomes, promoting social capital and exchange, and tailoring the intervention components to the desired empowerment‐related outcomes.

**Abbreviations**
3ie: International Initiative for Impact Evaluation
AAWAZ: Voice and Accountability Programme
AFD: French Agency for Development
AG: Advisory Group
AGEP: Adolescent Girls Empowerment Programme
AGI: Adolescent Girls Initiative
AU: African Union
AWPSs: all‐women police stations
BCC: behaviour change communication
BISP: Benazir Income Support Programme
BMZ: German Ministry for Economic Cooperation and Development
BRAC: Bangladesh Rural Advancement Committee
BWC: Business Women Connect
CAs: community activists
CB: capacity building
CBT: cognitive behavioural therapy
CCT: conditional cash transfer
CDC: community development councils
CDD: community‐driven development
CEDAW: Convention on the Elimination of Discrimination Against Women
CETA: Common Elements Treatment Approach
CFU: Conservation Farming Unit
COMPASS: creating opportunities through mentoring, parental involvement and safe spaces
CPSL: Centre for Promoting Sustainable Livelihoods
CSO: civil society organisation
DDR: disarmament, demobilisation and reintegration
DFID: Department for International Development
DRC: Democratic Republic of Congo
EDs: electoral divisions
EGM: evidence and gap map
E‐HFP: enhanced‐homestead food production
ELA: Empowerment and Livelihood for Adolescents
EMAP: engaging men through accountable practices
EPAG: Economic Empowerment of Adolescent Girls and Young Women
EU: European Union
FCAs: Farmer Community Associations
FCAS: fragile and conflict‐affected states and situations
FCDO: Foreign Commonwealth and Development Office
FCFA: Central African CFA franc
FSVGD: Food‐Security Vulnerable Group Development
FT: Fambul Tok
FTS: full text screening
GALS: Gender Action Learning System
GCRF: Global Challenge Research Fund
GE: Girl Empower
GHA: Gendered Household Approach
GLS: grey literature search
GPI: Global Peace Index
GSR: gender systematic review
HKI: Helen Keller International
IDP: internally displaced people
IFS: Integrated Food Security
IGVGD: Income‐Generating Vulnerable Group Development
ILO: International Labour Organisation
INGO: International Nongovernmental Organisation
IPV: intimate partner violence
IRC: International Rescue Committee
JLOS: justice and rule of law sector
KES: Kenyan shilling
LMICs: low‐ and middle‐income countries
MASVAW: Men's Action to Stop Violence Against Women
MDGs: Millennium Development Goals
MENA: Middle East and North Africa
MoFA: Ministry of Foreign Affairs
NAP: National Action Plan
NGO: Nongovernmental Organisation
NSP: National Solidarity Programme
OECD: Organisation of Economic Co‐operation and Development
PFP: Pigs for Peace
PKR: Pakistani rupee
PSM: propensity score matching
PSNP: Productive Safety Net Programme
QED: quasi‐experimental design
RAIN: Realigning Agriculture for Improved Nutrition
RCT: randomised controlled trial
RDD: regression discontinuity design
REDD+: reducing emissions from deforestation and degradation
RISHTA: research and intervention in sexual health: theory to action
RoB: risk of bias
RSS: regional stabilisation strategy
SATYA: Social Awakening Through Youth Action
SDGs: Sustainable Development Goals
SEA: sexual abuse and exploitation
SEWA: Self‐Employed Women's Association
SfC: Saving for Change
SGBV: sexual and gender‐based violence
SGSY: Swarnajayanti Gram Swarozgar Yojana
SHGs: self‐help groups
SR: systematic review
SRHR: sexual and reproductive health rights
TAS: title and abstract screening
TEVAW: Together to End Violence
TMRI: Transfer Modality Research Initiative
ToC: theory of change
TT: Tchova Tchova
TTHV: Tchova Tchova Histórias de Vida: Diálogos Comunitários
TUP: Targeting the Ultra‐Poor Programme
TVET: technical and vocational education and training
UBL: Unite for a Better Life
UCT: unconditional cash transfer
UN: United Nations
UNSC(R): United Nations Security Council (Resolution)
USD: United States dollar
VDC: village development committee
VFLs: village farm leaders
VGD: Vulnerable Group Development Programme
VMFs: village model farms
VSLAs: Village Savings and Loan Associations
WfWI: Women for Women International
WINGS: Women's Income‐Generating Support
WPS(A): Women Peace and Security (Agenda)
ZCTP: Zomba Cash Transfer Programme

## 1 PLAIN LANGUAGE SUMMARY

This 3ie systematic review (SR) aims to assess the effects of gender‐specific and transformative interventions on women's empowerment and gender equality in fragile and conflict‐affected states and situations (FCAS) with high levels of gender inequality and their contribution to building peaceful and inclusive societies.

### 1.1 Gender‐specific or transformative interventions increase women's empowerment and gender equality

Interventions supporting women's empowerment and gender equality in FCAS produced, on average, positive effects on the outcomes related to the focus of the intervention. There are no significant negative effects of any included interventions. Interventions do not achieve positive and significant effects for behavioural outcomes such as intimate partner violence (IPV).

### 1.2 What is this review about?

Across the globe, women face tremendous challenges when it comes to equitable access to resources, exercising meaningful agency and decision‐making power and aspiring to and accomplishing achievements. Despite gains made in recent decades towards inclusion and empowerment, significant challenges still remain for women, particularly in fragile and conflict‐affected settings where these challenges are exacerbated.

To help identify what approaches can improve women's empowerment in fragile settings with high levels of gender inequality, this SR synthesises the evidence on the effects of 14 different types of gender‐sensitive and gender‐transformative interventions.

### 1.3 What studies are included?

Our SR includes 104 unique studies covering 55 identified programmes and 32 linked impact evaluations papers, in addition to 90 linked qualitative and process evaluations. These studies took place in 29 countries identified as being particularly fragile and having a high level of gender inequality and all were published between 2009 and 2021.

Of the quasi‐experimental studies included, almost half were deemed as having a high risk of bias, and we assessed nearly all remainding quasi‐experimental studies as having some risk of biasconcerns. Among the studies using an experimental design, only around one‐third were assessed as having a high risk of bias.

### 1.4 What are the main findings of this review?

#### 1.4.1 Do gender‐specific or transformative interventions increase women's empowerment and gender equality?

Most of the identified interventions have significant positive effects on empowerment and gender equality outcomes closely related to the purpose of the intervention. Asset transfers and cash transfers show some of the largest effects on women's access to and ownership of assets, credit and income. Village Savings and Loan Associations and institutional provisions of savings and loans have the largest effects on women's capacity to use and understand financial, banking and business services effectively. Life skills and capacity building programmes realise their largest effects on women having better life skills. Inclusive community‐driven development has the largest effect on increased representation of women in local and subnational civil and political processes.

#### 1.4.2 What are the potential barriers and facilitating factors that affect the effectiveness of those interventions?

Through our qualitative synthesis, we explored potential explanations for the lack of effects further along the causal chain. Despite interventions demonstrating a positive effect on the outcomes they specifically targeted, there is little evidence of trickle‐down effects on other empowerment outcomes, which includes resources, agency and achievements. Few interventions led to positive effects on outcomes such as the attitudes of men towards women, agreement with justifications for IPV or women's participation in decision‐making processes at the household and community levels.

#### 1.4.3 What is the certainty of the body of evidence?

The Grading of Recommendations, Assessment, Development and Evaluations (GRADE) methodology was used to summarise and rate certainty of bodies of evidence. Of the 190 included bodies of evidence (including those based on single studies), the majority of the evidence is from very low (57%) and low (27%) confidence, while 14% are of moderate and 2% are of high confidence. Summary of findings tables in the main report provide concise explanations of how certainty of evidence ratings is assessed, allowing readers to understand any concerns surrounding a given effect estimate.

### 1.5 What do the findings of this review mean?

Qualitative findings show that gender and social norms acted as significant barriers to programme uptake across multiple intervention types, including cash transfers and safe spaces. Policy makers must ensure they recognise embedded power dynamics and specifically address patriarchal norms, as without these components, interventions are unlikely to succeed in their stated aims. Without addressing these barriers, secondary outcomes that are not specifically targeted by the interventions are unlikely to be affected. For example, only one intervention group, self‐help groups, resulted in a significant reduction of violence at the household level and no interventions reduced IPV.

There is a lack of evidence from certain regions and intervention types. Very few studies came from the Arabian Peninsula, although it is a highly relevant region considering the focus of our review. We also observe gaps of evidence related to gender‐specific and transformative interventions focusing either on recovery and relief or on the active role of women in peacebuilding.

The body of evidence covered in our review suggests that interventions explicitly addressing gender norms are well‐positioned to strengthen women's empowerment in fragile and conflict‐affected settings. Targeting the right beneficiaries, designing interventions to be gender‐transformative, and implementing with the context in mind are key to realising the goals of empowering women, promoting gender equity and building more peaceful and inclusive societies.

Although only a few interventions focused on the direct role of women as agents for peacebuilding, a large body of evidence focused on the indirect impact of gender equality towards inclusive and peaceful societies. Addressing women's empowerment and gender equality is then a major contribution to the reduction of violence and the building of sustainable peace.

#### 1.5.1 How up‐to‐date is this review?

The review authors searched for relevant studies in December 2020.

## 2 BACKGROUND

### 2.1 The problem, condition or issue

Despite gains made over the last decades towards equity, inclusion and empowerment, significant gaps remain for women accessing resources, earning livelihoods, achieving legislative and political representation, and participating in important decision‐making processes (Cornwall, 2016; United Nations, 2010). A growing body of literature indicates that these challenges are exacerbated in contexts of conflict and fragility (Bouta et al., 2004; Buvinic et al., 2013; Caprioli, 2000).

This is particularly alarming when presented alongside the reality that the prevalence of fragility and conflict‐affected states is on the rise. The World Bank reports that over the last ten years, the number of fragile and conflict‐affected states and situations (FCAS) has increased from 36 to 39 countries or states, with 17 classified as a ‘situation of medium‐ to high‐intensity conflict’ (World Bank, 2021). The Institute for Economics and Peace reports a similar deterioration of peacefulness, finding a 0.34% point drop last year in their Global Peace Index (GPI), which measures societal safety and security, ongoing domestic and international conflict, and degree of militarisation (Institute for Economics and Peace, 2020). According to the Organisation for Economic Co‐operation and Development (OECD), a staggering 23% of the world's population is living in a fragile context, including 76.5% of all those living in extreme poverty (OECD, 2020).

#### 2.1.1 Definition of key terms

##### 2.1.1.1 Fragile contexts

Defining fragility is a complex and sometimes contentious challenge due to the conceptual ambiguities that characterise it (Faust et al., 2013). We adopt the same definition for situations of fragility as used in the International Initiative for Impact Evaluation's (3ie) *Building peaceful societies: An evidence gap map* (Sonnenfeld et al., 2020). Situations of fragility can be understood as ‘…the combination of exposure to risk and insufficient coping capacity of the state, system and/or communities to manage, absorb or mitigate those risks. Fragility can lead to negative outcomes, including violence, the breakdown of institutions, displacement, humanitarian crises or other emergencies’ (OECD, 2016a). The focus on situations of fragility recognises that exposure to risks and vulnerabilities is not constant, neither over time nor within a state. For example, within a state that is not considered fragile, there may be communities that have the characteristics of fragility. Note that we operationalise a metrics‐based definition of fragility for our inclusion criteria which can be found in Methodology of Selection of Population.

##### 2.1.1.2 Gender equality and women's empowerment

Our SR will use Naila Kabeer's definition of women's empowerment: ‘…a process by which women who have been denied the ability to make strategic life choices acquire such an ability’ (Kabeer, 1999). This ability to exercise choice relies on three interrelated dimensions:

- *Resources*: material, human and social resources which serve to enhance the ability to exercise choice;
- *Agency*: ability to define one's goals and act upon them and operationalised decision‐making; and
- *Achievement*: ways of being and doing which can be realised by different individuals.

The different dimensions emphasise that *empowerment* encompasses different categories of daily life and as such, empowerment‐focused interventions for women and girls may take the form of many different programmes, as further specified in the *interventions* section of this protocol (Lwamba et al., 2021). Overall, empowerment contributes to the overall equality between men and women, improving one's ability to make choices and live a safe and fulfilling life (Cornwall, 2000).

##### 2.1.1.3 Peacebuilding

The former United Nations (UN) Secretary‐General Boutros Boutros‐Ghali provided one of the first definitions of the concept of *peacebuilding* as ‘an action to identify and support structures which will tend to strengthen and solidify peace to avoid relapse into conflict’ (Barnett et al., 2007). Like the *Building peaceful societies* evidence and gap map (EGM), our SR adopts the definition of peacebuilding developed by the UN Secretary General's Policy Committee in 2007, which defines peacebuilding as ‘a range of measures aimed at reducing the risk of lapsing or relapsing into [violent] conflict, by strengthening national capacities for conflict management and laying the foundations for sustainable peace. It is a complex, long‐term process aimed at creating the necessary conditions for positive and sustainable peace by addressing the deep‐rooted structural causes of violent conflict in a comprehensive manner’ (United Nations Peacebuilding Support Office, 2010).

There are three types of peacebuilding processes:

- Track I describes activities that bring parties to a conflict into direct negotiation to achieve an agreement or a resolution through official discussions between high level governmental and military leaders (includes ceasefires, peace talks, treaties, etc.);
- Track II describes activities of unofficial dialogue and problem‐solving aimed at building relationships between civil society leaders and influential individuals that can impact Track I; and
- Track III describes activities of people‐to‐people interactions at the grassroots level to encourage interaction and understanding between communities (includes meetings, media exposure, political and legal advocacy, etc.) (Dudouet, 2017).

Sustainable peace relies on numerous stakeholders, at all levels and beyond the lifetime of an active conflict or crisis. Our SR analyses the role of these stakeholders: private sector actors, the public, civil society organisations, civil servants and service providers, and individual households.

##### 2.1.1.4 Human security

A broad approach to fostering peaceful societies is one that examines and understands the dangers of fragility to not only be about physical security, but human security as well. The concept of human security originates from UNOCHA's Commission on Human Security to develop a world ‘free from want’ and ‘free from fear’ (UNOCHA, 2009). The Berghof Foundation defines human security as ‘…a comprehensive, people‐centred and prevention‐oriented concept that includes protection from threats in the area of economic, food, health, environmental, personal, community and political security’ (Berghof Foundation, 2019). Human security expands the concept of security beyond a state‐centric framework to focus more on the macro‐level of households and individuals. For women, there are vast differences and obstacles in attaining the same level of human security as men.

##### 2.1.1.5 Peaceful and inclusive societies

As presented in the Building Peaceful Societies EGM, peaceful and inclusive societies cover a wider spectrum than the absence of violence and the resolution of conflict. It is then important to understand the concept of peaceful and inclusive societies as the conjunction of addressed fragility, human security, positive peace but also sustainable and inclusive governance. This target of peaceful societies has been made a priority by a series of international agencies such as the Sustainable Development Goals (Keuleers, 2016) and the UK Foreign and Commonwealth Office who identified four objectives for peaceful states and societies (Department for International Development, 2010):

1. Address the causes and effects of conflict and fragility, and build conflict resolution mechanisms;
2. Support inclusive political settlements and processes;
3. Develop core state functions; and
4. Respond to public expectations.

Building on the approach initiated in the Building Peaceful Societies EGM, our review adopts a definition of *peaceful and inclusive societies* based on the association of the following concepts:

- Addressing the causes and drivers of fragility by building sustainable economic foundations and livelihoods.
- Addressing the roots causes and drivers of conflicts, strengthening social cohesion, supporting peace processes to build sustainable peace.
- Supporting human security through the prevention of violence and protection of human rights and security from any form of violence.
- Supporting good governance through the development of responsive and sustainable institutions and governance practices.
- Developing inclusivity by addressing the specific needs of marginalised and vulnerable groups (including women and girls).

#### 2.1.2 The issue: Women's empowerment in FCAS

**Figure 1:**
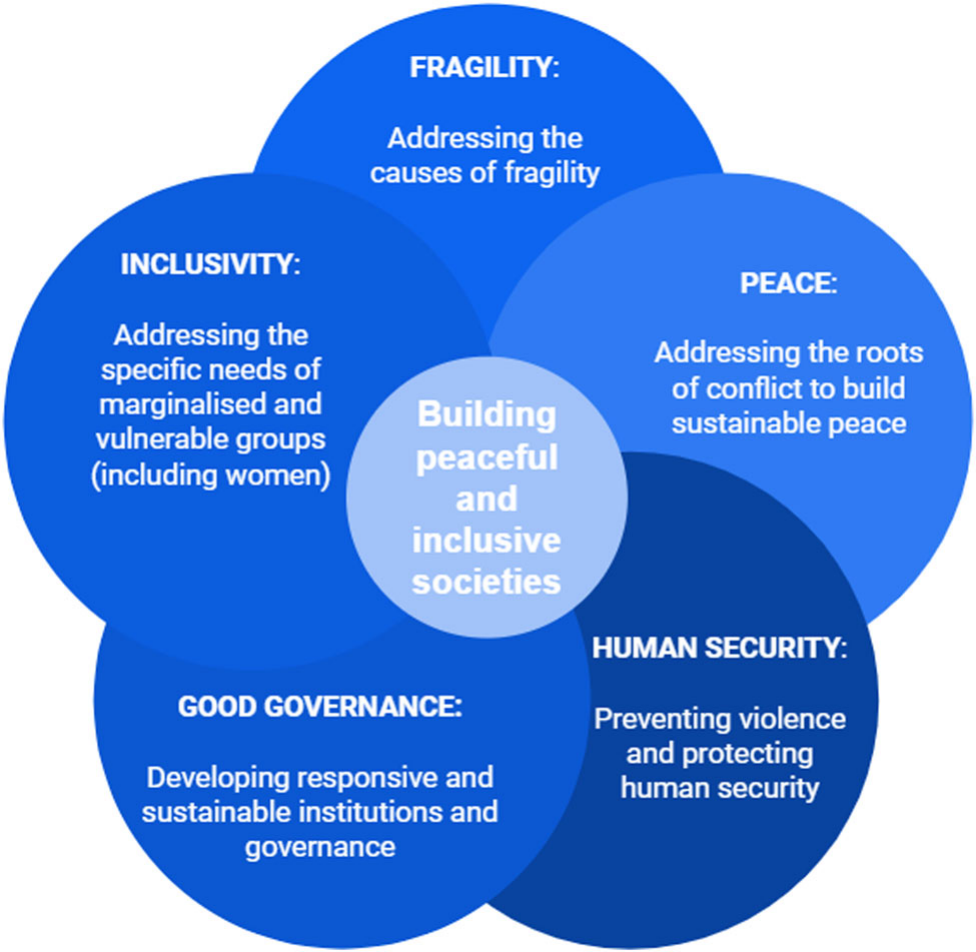
Building peaceful and inclusive societies

Women and girls are economically disempowered through restricted access to livelihoods and resources (Sweetman & Rowlands, 2016). The causes of this disenfranchisement extend from a lack of economic or social protection, to social norms and traditions, to legal and political barriers (Chen et al., 2006; Doss, 2013; Strickland, 2004). Examples of the kinds of systemic barriers that discourage women's economic autonomy are early and forced marriage, bureaucratic hurdles to accessing capital for entrepreneurship, sexual and gender‐based violence (SGBV), and discrimination and harassment (OECD, 2016b; Perrin et al., 2019) (Figure 1).

In addition to economic disempowerment, women are also often excluded from having a seat at the policy or decision‐making table. Women's movements are localised and often restricted to the grassroots, and when they have broader national reach, are often co‐opted by NGOs or government bodies (Jong & Kimm, 2017). Women and girls have insurmountable hurdles if they seek to enact change at all levels, from their communities to the national government (Goetz, 2008). Furthermore, at the legislative and political levels, equal rights that may be enjoyed by women and girls in national legislation are often not codified at the local or subnational level due to contravening customary law and social norms (UN Women, 2015).

In FCAS, these kinds of disadvantages and inequalities are greatly amplified. (Speake, 2013). Violence and fragility affect women and girls in many ways, some of which differ from their impact on men and boys. For example, sexual assault and exploitation of women are often used as ‘tools of war’, and threats, such as child marriage and human trafficking of women, are often exacerbated during conflicts (USAID, 2007). There are many drivers of the differences in effects of conflict on women and girls from men and boys, such as the implications of the different social roles assigned to men and women, which vary by context. Understood in this way, the effects of conflict on women do not stem from any intrinsic weakness in women, but are rather a consequence of their position in society (Pankhurst, 2000).

In addition to the immediate threats to women, conflicts can have a destabilising effect on gender norms in the long term. Gender roles can change during conflicts by increasing the burden and responsibilities of women while men are away fighting. Such shifts in gender and social norms can manifest as new sources of conflict when women are expected to return to pre‐conflict status (United States Institute for Peace, 2012).

At the root of this issue is the exclusion of women from the processes that foster peaceful and inclusive societies. A study of 31 major peace processes between 1992 and 2011 by UN Women found that only 2.4% of chief mediators, 4% of peace signatories and 9% of negotiating delegations were women (O'Reilly, 2013). Between 2000 and 2010, less than a third of peace agreements signed contained a gender reference (Hedström & Senarathna, 2015). The exclusion of women's voices and contributions to peace processes not only leave women disenfranchised, underrepresented and vulnerable, but studies have shown that limiting women's participation is associated with greater recidivism and return to conflict (Bigio & Vogelsteing, 2016; Council on Foreign Relations, n.d.; Hedström & Senarathna, 2015; O'Driscoll, 2017).

In summary, there cannot be positive peace, or the creation of social systems and foundations for durable, peaceful and inclusive societies, without an empowered female population that participates in the restoration and/or construction of relationships (Galtung, 1996).

#### 2.1.3 A solution: Women's empowerment and gender equality in FCAS

Processes that develop peaceful societies are not only opportunities to strengthen, reconstruct or promote the resilience of social cohesion but also to transform the roles and definition of social relations towards inclusivity, participation and peace (Aall & Crocker, 2019). Rebuilding social cohesion and inclusive societies may be buttressed by women's empowerment and by the promotion of gender equality for peace and through peace (Björkdahl & Höglund, 2013). Protecting women and girls' human rights, safety, physical and mental health and security, promotion of their Socioeconomic recovery and increased participation in decision‐making processes and responses related to conflict or fragility are key processes that lead to overall progress towards gender equality and women's empowerment (Buvinic et al., 2013).

Empowering women across all three dimensions of gender equality and women's empowerment, according to Kabeer, often requires structural and systematic changes (Kabeer, 1999). Implementing transitions based on peace, nonviolence and inclusion can be driven by repealing old laws and instituting new structures, but must also transform harmful social norms that exclude women from publicly and meaningfully participating in society (McWilliams & Kilmurray, 2015). This includes ensuring that women have equal rights, are treated equally and have their voices heard and needs met. Promoting the role of women and facilitating exchanges between them help change those mindsets and promote greater gender equality, reduce conflict, and discourage extremism. In that sense, women's empowerment can both be a driver and a consequence of peace: empowerment *through* and *from* peace (Cheldelin & Mutisi, 2016).

#### 2.1.4 UNSCR 1325 as the starting point of change towards inclusive peace

When the United Nations Security Council Resolution (UNSCR) 1325 (United Nations Security Council, 2000) was adopted on 31 October 2000, it represented a major milestone in acknowledging the disproportionate impact of armed conflict on women and girls in continuation of previous work started in the 20th century. The resolution, which has been used extensively as a policy tool to implement gender‐sensitive conflict‐related policies, affirms three major points:

I. The recognition of the inordinate impact of violent conflicts and war on women and girls;
II. The recognition of the crucial role that women should play in conflict prevention, resolutions, building peaceful and inclusive societies; and
III. The necessary adoption of a gender perspective in conflict prevention, resolutions, and building peaceful and inclusive societies.

UNSCR 1325 has widely been interpreted as asserting those objectives by establishing four dimensions of women's roles in conflict: participation, prevention, protection and relief and recovery through gender equality and for gender equality.

Although UNSCR 1325 prioritised women's empowerment for peace and initiated the Women, Peace and Security (WPS) Agenda, some criticisms have been raised (Senarathna, 2015). First, women are not a homogeneous group that can be represented as an entity in peace processes. Gender is both multidimensional and intersectional and thus a *one‐size‐fits‐all* approach may not be appropriate. Second, the formulation of *women as agents of peace* has been criticised as being *instrumentalist*, focusing on what women can bring to peace and not what peace can bring to women. Finally, women are not only exposed to violence in periods of conflict but may also be highly exposed to violence in times of peace and both should be considered in the WPS. Despite these critiques, the UNSCR 1325 has been the starting point of 20 years of change toward inclusive peace (Desmidt & Davis, 2019). See Supporting Information Appendix SI, Table 2 for a more complete chronology of the impact of UNSCR 1325.

UNSCR 1325 has initiated a global movement towards consensus on the importance of the role of women in building peaceful and inclusive societies, but concrete implementation of the recommendations remains scattered. In 2018, out of 52 agreements across a range of issues for peace, only four contained gender‐related provisions (UN Women, 2020). In 2019, only 41% of the UN Member States had adopted a National Action Plan (NAP) for the UNSCR 1325 (Desmidt & Davis, 2019). Though it is still early to evaluate its impact, the novel coronavirus (COVID‐19) has and will continue to aggravate existing intersectional risks, strain the coping capacities of those most vulnerable, increase marginalisation and it is quite likely that these will be variables to consider in current or ongoing peace processes. This SR operationalises UNSCR 1325 by incorporating its four pillars into our framework to understand and classify intervention groups.

### 2.2 The intervention

#### 2.2.1 Conceptual scope of the review

A wide breadth of different interventions is currently being implemented to help empower women in FCAS contexts. As per Table 6, to structure our review of this broad range of interventions and to specify the types of intervention we include, we have developed a guiding framework to:

1. Include only those intervention groups that are either *gender‐specific* or *gender‐transformative*, as guided by the World Health Organization's *Gender Responsiveness Assessment Scale* (World Health Organisation, n.d.). Gender‐specific interventions are those that consider gender norms, roles and relations for women and men and how they affect access to and control over resources. They intentionally target and benefit a specific group of women or men to achieve certain policy or programme goals or meet certain needs. Gender‐transformative interventions are those that address the causes of gender‐based inequities and include ways to transform harmful gender norms, roles and relations to foster progressive changes in power relationships between women and men. Given that our review aims to identify the effectiveness of interventions whose specific and explicit aim is to empower women in FCAS, we have used this device as a filter to exclude studies which may include a disaggregation for sex in their study design, but for which the intervention was more broadly targeted. As a result, we considered intervention groups of *all* genders and ages, with an explicit focus on gender. This includes any focus on Lesbian, Gay, Bisexual, Transgender, Queer and/or Questioning, Intersex and Asexual (LGBTQIA+) groups to represent sexual and gender diversity.
2. Include only intervention groups that operate within the micro‐ (individual) and meso‐ (household and community) spheres of the ecological framework not those which operate at the macro (or society) level. As is outlined by Bronfenbrenner, we recognised that an individual's immediate setting ‘…is affected by the larger context in which the settings are embedded’ (Bronfenbrenner, 1979). That said, our decision to focus on the lower levels of the ecological framework was driven by this review's focus on those interventions which work directly with individuals and their communities.

Classify interventions based on the four pillars of UNSCR 1325. While some interventions work across multiple pillars, we have decided to operationalise this widely used framework to help organise our findings. In doing so, our review is in line with national, international, and multilateral development priorities and the evidence that we present is easily navigable and applicable for policy makers and programme managers (Table 1).

**Table 1:** Conceptual scope of the SR

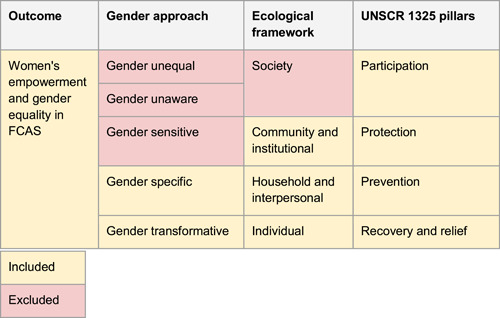

These UNSCR 1325 pillars are larger than the possible scope of this review, and not all interventions within them are included. Further detail follows in the summary of included interventions.

#### 2.2.2 Summary of included interventions

The scope of efforts which aim to empower women in FCAS is vast, and likewise, the body of impact evaluations (IEs) significant. In Table 6, we operationalise our conceptual scope as a tool to help identify specific categories of interventions to include in this SR. As an extension of that, the following are descriptions of the categories of interventions we intended to include within each of the four UNSCR pillars.

##### 2.2.2.1 Participation

For our review, we define participation interventions as those that create opportunities for, build acceptance of, or strengthen capacities for the equal participation and full involvement of women and girls in political, economic and social institutions and decision‐making processes (Table 2).

**Table 2:** Participation pillar, included studies

Intervention	Description
Civil society, associations and networks	Interventions that aim to strengthen the technical capacity and institutionalisation of civil society, associations and networks, governments and market driven associations working for gender equality or women's empowerment. This also includes the establishment and support of women's economic associations, such as cooperatives and activity groups
Economic rights and entitlements	Interventions that protect or support women's access to land rights and entitlements, such as unemployment benefits
Formal education	System, school and teacher‐level interventions that promote gender equality, girls' access to and the gender sensitivity of formal education and/or develop human capital amongst girls and women through school‐based education. This can also include education financing and planning interventions such as vouchers for girls to attend low‐fee private schools
Nonformal education	Access to education and educational resources for women and girls that occurs outside of traditional schools including community‐based education, camp‐based education, etc.
Technical and vocational education and training	Education for women and girls that provides knowledge and skills useful for employment, jobs placement in the formal sector
Micro, small and medium enterprises	Support for women‐run micro, small and medium enterprises. This may include incubation‐style interventions, loans, technical support, entrepreneurship training, mentorship or start‐up kits
Cash‐based approaches to support women's access to and participation in education and/or the economy	Cash‐based interventions, which aim to develop human capital (e.g., enable girls' access to education) or empower women to participate in the economy
Financial inclusion	Interventions that provide access to credit, savings, insurance and other financial products, either on an individual or group‐based approach. This includes self‐help groups, Village Savings and Loan Associations, savings groups, etc.
Community and leisure activities	Interventions that create opportunities or encourage support for women's participation in community and leisure activities, such as sports, art, theatre, etc.
Civic education and youth leadership	Interventions that build capacity and create opportunities for women and girls to understand and participate in politics and political processes. This also includes support to girls and young women to learn skills for and access mentorship and opportunities to become active members of their communities
Voice and participation in local and subnational governance and development bodies	Interventions that aim to increase the voice and participation of women and girls in local government through quotas, outreach and encouragement campaigns, etc. This also includes efforts to strengthen the responsiveness of local and subnational governance and development bodies to women's needs and priorities. Finally, it includes interventions that provide training or opportunities enabling women and girls to hold public services and government accountable for service provision and protection of their rights

##### 2.2.2.2 Prevention

For our review, we define prevention pillar interventions as those that build capacities and systems to support the gender‐responsiveness and inclusivity of violence prevention and conflict transformation processes. This also includes efforts to hold perpetrators of violence accountable through formal or informal means (Table 3).

**Table 3:** Prevention pillar, included studies

Intervention	Description
Conflict early warning systems	Interventions that facilitate women's involvement in, or the gender‐inclusiveness or gender‐responsiveness of community‐based or online conflict early warning systems
Dialogue groups	Interventions that either facilitate women's involvement in or set up women‐specific community dialogue groups. These can be forums that draw participants across the community to exchange ideas in face‐to‐face moderated sessions, share personal stories and experiences, express perspectives, clarify viewpoints and develop solutions to problems. These may include community conflict prevention fora, dialogues to resolve or transform specific conflicts or dialogues to build social cohesion
Community consultations	This includes interventions that create specific consultations for women at community and subnational levels as part of a formal peace process
Capacity building for conflict transformation	Interventions that build women's capacity to participate in or the gender‐sensitivity of community and subnational conflict transformation processes. These may also be referred to as building skills for mediation, negotiation, conflict resolution or conflict prevention
Peace education	This includes interventions that aim to teach people about the importance of including women in peace processes

##### 2.2.2.3 Protection

For our review, we define the protection pillar interventions as those that create, facilitate access to, or build awareness of and support for legal or social protections for women's and girls' rights. This includes behavioural, legal and environmental interventions that aim to reduce women and girls' risk of experiencing SGBV (Table 4).

**Table 4:** Protection pillar, included studies

Intervention	Description
Legal rights education	This includes interventions that disseminate information and build understanding of women's rights, among men and women, and capacity‐building on ways to demand their rights
Behaviour change communication around support for women's rights and preventing SGBV	Community‐based and subnational‐level interventions that aim to change behaviours, attitudes and beliefs around gender equality and the role of women in society, as well as efforts to reduce the prevalence of SGBV. This could include the targeting of gender norms within communities through social institutions, such as churches, community groups or cooperatives. It could also include efforts to sensitise families on the negative consequences of SGBV, particularly for children. These interventions may target local community and civil society leaders, local and subnational government, the private sector, etc. This would also include media interventions such as radio dramas or participatory theatre aiming to encourage discourse around women's rights and empowerment
Capacity building and technical support to subnational government officials to strengthen service provision for women and gender equality	These interventions build the capacity of subnational and local government officials to provide services for women, meet women's needs and improve understanding of women's rights and the importance of gender equality. This may target gender equality‐specific officials such as officials within a Department of Women's Affairs or aim to strengthen the capacity and service provision for women within sector‐specific services such as education officials
Preventative protection measures	These interventions comprise nonpolice or security force‐based efforts to reduce incidences of violence, especially SGBV. They include making the physical environment less conducive to such acts and minimising the exposure of vulnerable groups to risky situations. This can include crime prevention through environmental design intervention, installing lighting in public spaces, women‐friendly transport systems and public facilities, removing obstacles so there is a better line of sight and reclaiming spaces for positive community activities. They may also include interventions to reduce vulnerable groups' risk of exposure to violence (e.g., through providing firewood or alternative fuel sources to women in refugee camps)
Gender‐sensitive policing	Interventions that strengthen the approaches and capacity of police forces to prevent SGBV, improve police support to and treatment of women and girls, and support victims in a respectful way that does no harm. This includes, for example, training and capacity building interventions targeting police forces that strengthen their awareness of the importance of gender equality and women's legal rights, train them on best practices for protecting victims and interventions that create women‐specific police officers to facilitate access to reporting crimes
Informal judicial system	Interventions supporting women's access to or participation in informal or semi‐formal justice processes, such as alternative dispute resolution mechanisms or village courts

Abbreviation: SGBV, sexual and gender‐based violence.

##### 2.2.2.4 Relief and recovery

For our review, we define relief and recovery interventions as those that build capacities and systems to support the gender‐responsiveness and inclusivity of relief and recovery processes related to conflict, displacement and natural disasters (Table 5).

**Table 5:** Recovery and relief pillar, included studies

Intervention	Description
(Re)integration of forcibly displaced populations	This includes gender‐specific or transformative interventions that target forcibly displaced populations, with the aim of supporting (re)integration into host or home communities. This may include economic support such as cash‐based approaches (e.g., cash transfers and vouchers); social reintegration such as through the organisation of social activities or sports leagues; and community development inclusion such as strengthening the inclusion of displaced women in community‐driven development interventions
Disaster risk reduction	Interventions to support women's participation in or the gender responsiveness of community‐based disaster risk reduction activities (such as hazard vulnerability and risk assessments)
Disarmament, demobilisation and reintegration	These interventions support forcibly displaced populations to (re)integrate into host or home communities. This may include economic support such as cash‐based approaches (e.g., cash transfers and vouchers); social reintegration such as through the organisation of social activities or sports leagues; and community development inclusion such as strengthening the inclusion of displaced women in community‐driven development interventions

### 2.3 How the intervention might work

Our framework acknowledges both the wider and more specific contexts within which gender‐specific and transformative interventions are implemented in FCAS. Interventions operate within a wide context of international standards and practices, including UNSCR 1325, the 2030 SDGs or the WPS Agenda, and this global context overlays the unique local context of any given intervention where those general standards are applied. The framework captures the reality that while broader principles often guide intervention design, the characteristics of fragility and conflict depend on the location, motivations, interactions and local specificities of the intervention fields.

**Figure 2:**
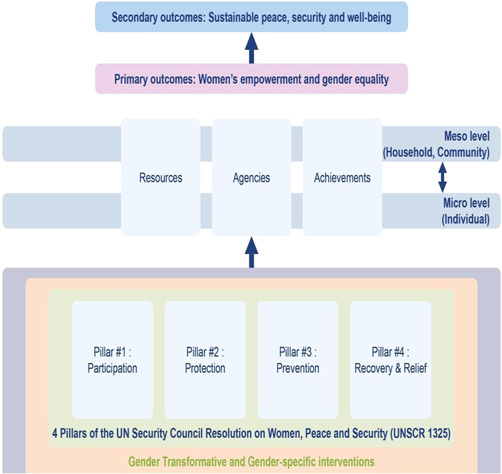
Our systematic review's theory of change

As per the conceptual scope, the below theory of change (ToC) (Figure 2) includes interventions operating in the micro‐ and meso‐levels of the ecological framework and that are gender‐specific or transformative and those that work to develop the resources, agency and achievement levers of women's empowerment and gender equality.

Importantly, the UNSCR Pillars are not necessarily mutually exclusive. Interventions can work across multiple pillars, levels and levers with the common objective to impact women's empowerment and gender equality. Lastly, the primary outcome of the review is women's empowerment and gender equality that will then contribute to achieving the secondary outcome of peaceful and inclusive societies.

### 2.4 Why it is important to do this review

#### 2.4.1 Review of existing literature

##### 2.4.1.1 The existing literature

3ie's recent EGM, *Building peaceful societies*, demonstrated that a considerable body of literature exists regarding the impact of interventions that focus on women and girls in FCAS. There is a wide scope of experimental and quasi‐experimental primary research focusing on peaceful and inclusive societies and women's empowerment. As is outlined above, much of this evidence suggests a link between the integration of gender‐sensitive approaches in FCAS context and lasting achievements in building, ensuring human rights, and promoting inclusive societies. That said, only a handful of SRs focused on the role of women in peace processes and there is a stronger emphasis on empowerment of women in fragile contexts for sustainable peace.

After completing a comprehensive scoping of existing SRs and IEs through the 3ie Evidence Portal, we identified seven IEs and two EGMs focusing on the topics of building peaceful societies and fragility. 3ie notably contributed to the production of the two evidence gaps maps focusing on *Building peaceful societies* (Sonnenfeld et al., 2020) as an update and expansion of the 2015 EGM on *Evidence for peacebuilding* (Cameron et al., 2015). The *Evidence for peacebuilding* EGM identified over 100 IE, four SR and 2 protocols. The *Building peaceful societies* EGM identified about 250 IEs, 34 SRs and 4 protocols.

Women's empowerment in fragile contexts has been substantially reviewed. A search of 3ie's Development Evidence Portal identified 39 IEs, 16 SRs, and one EGM focusing on topics of women and girls' empowerment, including contexts in FCAS.^1^ As identified through the focus of the EGM (Picon et al., 2017), most of the reviews with an explicit and specific focus on women in FCAS focus on the prevention of and recovery from intimate partner violence (IPV).

There is also a substantial literature of SRs and IEs integrated within the second pillar of our framework (prevention) with a high prevalence of IEs focusing on the incidence or reaction to IPV with 62 out of 153 identified IEs. SGBV and IPV are also the focus of five SRs and two protocols (Delkhosh et al., 2017; Signorelli et al., 2018).

The Spangaro‐led review (Spangaro, Adogu, et al., 2013) notably found that although guidelines and training courses have been developed at the policy level, few initiatives have been implemented and evidence is lacking to judge the effectiveness of interventions designed to address or prevent sexual violence in conflict‐ or crisis‐affected settings. IPV has also been the focus of a *systematic review of structural interventions for IPV in LMICs* (Bourey et al., 2015) *and in humanitarian crises* (Warren et al., 2015). Some reviews also focused on the recovery and health aspects of IPV (Rivas et al., 2015; Tol et al., 2013).

Our literature scoping also identified a cluster of studies focusing on women's economic empowerment. Reviews of economic empowerment interventions include community‐based programmes such as economic self‐help groups where some reviews identified positive effects on women's political and economic empowerment (Brody et al., 2017). Others focus on the role of economic resources transfer for women's empowerment (Kabeer & Waddington, 2015). A third type of economic empowerment focuses on the impacts of business‐oriented programmes such as business and vocational training to improve women's labour market outcomes that tends to have more impact when they are combined with cash transfer or life skills training (Balarin et al., 2017).

Several reviews include a specific focus on livelihoods (Gibbs et al., 2012) at the community level to complement reviews integrating a health lens at the individual level (Kraft et al., 2014). Such characteristics of the literature landscape confirms the relevance of analysing women's empowerment through an ecological framework.

The political dimension of empowerment is also highly reliant on the community level of the ecological framework through the mobilisation of civil society groups to serve empowerment purposes notably in the subcategory of reproductive health for marginalised groups (Handanagic et al., 2016; Moore et al., 2014). Although we were able to find numerous references to gender empowerment in SRs, none of them have a specific focus on gender empowerment for and through building peaceful and inclusive societies processes in FCAS.

Additionally, though women's empowerment in fragile contexts is at the core of our review, it is important not to forget the wider spectrum of governance and social protection themes integrated in this review. The 3ie Evidence Portal gathers over 170 IEs, 11 SRs and 2 EGMs, including one in‐progress, covering issues of governance. Themes such as accountability, transparency, management of resources, public administration are all relevant aspects for the analysis of fragile and conflict‐affected situations (FCAS) as they are both sources of conflict and opportunities for peace. The two EGMs notably focused on the State‐Society relations (Coffey et al., 2017) (18 SRs, 2 protocols and 365 IEs) with a major focus on public institutions and services and on the effect of rule of law interventions on justice outcomes (Doherty et al., 2020).

##### 2.4.1.2 Our contribution to the existing literature

The above summary of findings from existing reviews reveals that although both fragility and women's empowerment are key areas of focus of systematic research, only a handful of studies focus on women's empowerment in FCAS and/or gendered approaches to address fragility. Even when they do, there usually is only particular emphasis on the impact of fragility on women rather than women's roles in shaping peace.

This initial summary confirms the relevance of the conceptual scope of our review as we saw that several reviews focused on some of its aspects, including a specific attention to gender‐transformative and gender‐specific IE in the SRs covering women empowerment that matches our approach. Many of the SRs focus through the lens of one of the levels of the ecological model with many of the reviews focusing on the community level. Finally, gender‐ and peacebuilding‐focused SRs overlapped on one or several of the UNSCR 1325 pillars (participation, prevention, protection and recovery and relief).

These gaps identified in the systematic literature should not be interpreted as a lack of interest for women's empowerment for peace and inclusivity. This literature review allowed us to identify several IEs already covering relevant aspects for our study. The gaps of literature are mainly due to the challenging analytical aspects that this topic raises. The high amount of nonsystematic literature covered through the scoping stages of our research is a good illustration of the interest of these topics for researchers. This review contributes to the SR literature through the development of understanding of the causal impacts of gender sensitive and gender transformative interventions in FCAS.

#### 2.4.2 Relevance to policy and practice

##### 2.4.2.1 Relevance to international and national development goals

An objective of our study is to feed into some of the international goals and approaches supporting women empowerment and gender equality in fragile contexts. Internationally, the interest in gender equality and its relationship to peaceful societies can be measured in the number of investments being made in both the SDGs relevant to gender equality and fragility, and national and regional‐level initiatives. An estimated $1 trillion USD would be necessary to meet SDG #5 on gender equality and #16 on peaceful and inclusive societies (Peace Women, 2016). There is growing recognition that investment in military spending is not the only solution for peace, but that solution can also come through an increased investment and recognition of women's rights and gender equality worldwide. Some international mechanisms have been created, such as the Addis Ababa Action Plan on Transformative Financing for gender equality and women's empowerment (Peace Women, n.d.), the Compact on WPS‐HA of the Generation Equality Forum, and the Global Acceleration Instrument (GAI) for WPS and Humanitarian Action, that represent pooled, transnational sources of funding for gender equality and women's empowerment.

At the national level, donors and countries have taken some measures to target women's empowerment and gender equality in FCAS since 1325 was introduced but more generally in the support of achievement of international development and growth. The German international development agency, GiZ, published its vision for promoting gender equality as one of the key values of its approaches abroad and as a prerequisite and driver of sustainable development (Langenkamp, n.d.). Global Affairs Canada (GAC) updated its international assistance policy in 2020 to include a feminist perspective. The updated policy recognises the importance of supporting gender equality and the empowerment of women and girls as it is the best way to build a more peaceful, more inclusive and more prosperous world (Global Affairs Canada, 2017). The United Kingdom's former Department for International Development (DfID), which is now the Foreign, Commonwealth, and Development Office (FCDO), published a Strategic Vision for gender equality in 2018. The document is presented as a call to action, recognising that action is needed if gender equality is to become a lasting reality (Department for International Development, n.d.‐c). The Ministry of Foreign Affairs of the Kingdom of the Netherlands publishes reports every 4 years to focus its impact on UNSCR 1325 and the WPS Agenda (Ministry of Foreign Affairs, n.d.).

##### 2.4.2.2 Relevance for donors and intervention design

Donors and implementing agencies have led interesting and innovative interventions in FCAS to increase the role of women in fostering peaceful societies.

The German Ministry for Economic Cooperation and Development (BMZ) implements a project in conjunction with the African Union (AU), entitled ‘Support to the African Peace and Security Architecture’ (APSA), to work with regional organisations and other international stakeholders to improve the participation of women in activities in the peace and security sector. BMZ has also supported the operationalisation of the AU‐supported Network of African Women in Conflict Prevention and Mediation (*FemWise‐Africa Network*), which collaborates with civil society to build the mediation skills of young women and bridge intergenerational gaps. The project seeks to promote the role of women in preventive diplomacy and mediation at all levels, ensure channels for their meaningful participation, initiate their action in line with the SDGs, bridging the gap between different levels of mediation and establishing local and national peace infrastructure for long‐term initiatives. Germany also recently adopted its third UNSCR 1325 NAP for the period 2021–2024 following its two previous plans for 2017–2020 and 2013–2016.

BMZ also supports compliance training addressing fundamental human rights and zero tolerance for sexual abuse and exploitation (SEA). More recently, BMZ has offered support to the implementation of the regional stabilisation strategy for the Lake Chad Basin region, mental health support in Boko Haram‐affected regions and hosted regional workshops on SGBV.

Since 2011, the *Red Nacional de Mujeres* publishes an annual report on monitoring the progress of the implementation of UNSCR 1325 (Red nacional de mujeres, n.d.), which has been in conjunction with the country's NAP on 1325. *Ruta Pacífica de las Mujeres*, an organisation that was key in advocating for the inclusion of women's voices in the 2016 Colombian Peace Treaty, includes a list of indicators across all levels of the ecological framework in their strategic plan to monitor progress on women's empowerment in the Colombian context (Ruta pacifica de las mujeres, 2016).

In Pakistan, the FCDO funds the Voice and Accountability programme (AAWAZ), which brings together three critical components focusing on the interconnection of gender, conflict resolution and citizen engagement (Department for International Development, n.d.‐a). This strives to focus on the enhanced political participation of women and in larger public life without fear of sexual or gender‐based violence, while striving to attain social harmony. This is set against the backdrop of promoting civil society, by having active and informed participation of citizens and their organised groups. More broadly, the FCDO also funds the Global Challenge Research Fund (GCRF) Gender, Justice and Security Hub (Department for International Development, n.d.‐b). On a higher level, the Hub functions to deliver ambitious and impactful research facilitating gender justice and inclusive security in FCAS.

The Dutch Ministry of Foreign Affairs funds, through its SDG5 Fund, a variety of modalities, including the WPS fund, the Power of Women fund and grants for protecting women and girls sexual and reproductive health rights. Consequently, the overall objective of the strategic WPS‐partnerships is to contribute to an enabling environment for women's participation and empowerment in conflict and post‐conflict environments, so they can meaningfully participate in conflict prevention, resolution, fostering peaceful societies, protection, relief and recovery.

##### 2.4.2.3 Relevance for policy design and decision making

Although it is still an ongoing process, we can already see some positive impacts of the WPS agenda. Data notably shows an increase in bilateral aid in support of gender equality and women's empowerment in FCAS (UN Women, n.d.) from some of the key states of funding of international development programmes. According to UN Women, all top bilateral donors increased their proportion of spending in support of gender equality and women's empowerment in FCAS over the last decade alone. For example, the United States gave approximately $2 billion USD in 2010, yet increased that number still to nearly $3 billion in 2017. However, we see that the proportion allocated by these same countries to bilateral aid dedicated to gender equality and women's empowerment as a principal objective in FCAS is lower than 6% of aid and has decreased over the last decade.

The adoption of NAPs by individual countries to adopt the WPS Agenda as outlined in UNSCR 1325 has some encouraging results in Central and West Africa and Latin America but is still an ongoing process in Southeast Asia and in the MENA region; we see a relatively low rate of adoption of NAP in medium to high level of violence conflict regions (UN Women, n.d.). Some examples of NAP are briefly presented below (London School of Economics, 2019). See Supporting Information Appendix SI, Table 1 for some examples of different National Plans for Action on UNSCR 1325.

Since the first NAP was pioneered in 2005 by Denmark, over 80 countries have piloted their own NAPs focusing on the WPS agenda (Peace Women, 2014). However, despite there being nearly 84 NAPs (as of December 2019), very few (33%) contain an allocated implementation budget. Additionally, only 31% have references to disarmament. Lastly, while the majority of NAPs mention the role of grassroots leaders and civil society (75%), most allocate them to an advisory role. There are 11 additional regional action plans, such as for regional bodies like the AU and EU. Regional coordination efforts also include the Asia‐Pacific Regional Symposium on NAPs on WPS where the Member States, alongside civil society representatives, share their lessons learned and best practices in the implementation of UNSCR 1325.

Twenty years after the introduction of UNSCR 1325, the purpose of NAPs and their impact has yet to be fully explored. The dynamic nature of conflict and the intersections of gender often mean that perspectives are constantly shifted and not always accurately captured in plans or data (Hamilton et al., 2020). Additionally, without implementation plans or budgets, NAPs ultimately end up being solely documents that do not move past rhetoric towards commitment. These trends illustrate the challenges raised to increase the role of women in building peaceful and inclusive societies. Our review then helps inform decisions about how to spend the resources available by providing a comprehensive review of the evidence on the impact of women as agents of change to build peaceful and inclusive societies in FCAS.

## 3 OBJECTIVES

This review builds on 3ie's EGM of the IE and SR evidence base of interventions aiming to promote peaceful and inclusive societies in fragile contexts (Sonnenfeld et al., 2020). The EGM identified a cluster of studies evaluating gender equality‐focused behaviour change communication (BCC) programmes and raised interest in investigating the evidence base for understanding the role of women more broadly as agents of change in developing peaceful and inclusive societies.

Building on the cluster of evidence identified in the EGM, our review increased generalisability of findings from single studies and focuses on interventions across a broad range of geographical locations, settings and populations, types of implementations and outcomes. We also address (when possible) the identified gaps in literature regarding meta‐analysis in conflict‐affected contexts.

As such, we propose the following objectives:

1. The primary objective of this review is to identify, assess and synthesise evidence on the effect of gender specific and gender transformative interventions within the context of the four pillars of UNSCR 1325 on women's empowerment and gender equality in FCAS. The SR facilitates the use of evidence in informing policy and practice decisions within the field of transition aid, particularly as it relates to gender focused programming.
2. Our second objective is to assess how these interventions contribute to inclusive and sustainable peace in conflict affected situations. We compare the effectiveness of these different types of interventions through the lenses of their ecological level, types of impact on women's empowerment, local context of gender inequality and conflict.

To achieve these objectives, we aimed to answer the following questions:

1. What are the impacts of gender transformative and specific interventions on women's empowerment and gender equality in FCAS?
2. What are the effects of these interventions on sustainable peace?
3. To what extent do effects vary by population group, ecological level and types of interventions?
4. What are contextual barriers to and facilitators of intervention effectiveness?

## 4 METHODS

The review followed the Campbell and Cochrane Collaborations approaches to systematic reviewing (Cochrane Collaboration, n.d.; Hammerstrøm et al., 2010; Shemilt et al., 2013). The review also drew on the concepts of theory‐based IE (White, 2009) and theory‐based SRs (Snilstveit, 2012) to provide a mixed‐methods SR and analysis along the causal chain, reviewing evidence on context, process and implementation to identify barriers and facilitators and analyse the effectiveness of interventions aiming to improve women's empowerment and gender equality outcomes.

The review systematically collected and synthesised quantitative evidence from IEs meeting our inclusion criteria to answer our review questions. If sufficient data was available, outcomes were synthesised in sub‐groups of populations, types of interventions and local contexts. For the review to be more useful for policymakers and practitioners, we also collected qualitative evidence from the included studies to assess factors that determine or hinder the effectiveness of interventions using a combination of qualitative synthesis and meta‐regression analysis.

**Figure 3:**
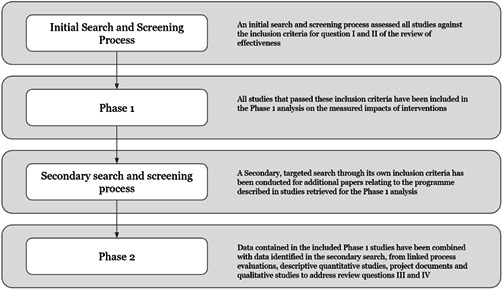
Overview of the review process

The review included studies in two phases as per Figure 3. To address Questions 1 and 2, we included studies meeting the IE study design inclusion criteria. To address the Questions 3 and 4, IE studies that pass these criteria were then used as the basis for a second phase to identify and include qualitative studies, project documents and process evaluations.

### 4.1 Criteria for considering studies for this review

#### 4.1.1 Types of study design

We included studies in two stages, in a similar approach to Snilstveit et al. (2015, 2019). In the first stage, we included studies that assessed the effects of interventions using experimental designs or quasi‐experimental designs (QEDs) with nonrandom assignment that allow for causal inference (to address primary research objectives 1 and 2). Specifically, we included the following:

- Randomised controlled trials (RCTs), with assignment at individual, household, community or other cluster level, and quasi‐RCTs using prospective methods of assignment such as alternation.
- Non‐randomised studies with selection on unobservables:
  o Regression discontinuity designs, where assignment is done on a threshold measured at pre‐test, and the study uses prospective or retrospective approaches of analysis to control for unobservable confounding.
  o Studies using design or methods to control for unobservable confounding, such as natural experiments with clearly defined intervention and comparison groups, which exploit natural randomness in implementation assignment by decision makers (e.g., public lottery) or random errors in implementation, and instrumental variables estimation.

- Non‐randomised studies with pre‐intervention and post‐intervention outcomes data in intervention and comparisons groups, where data are individual level panel or pseudo‐panels (repeated cross‐sections), which use the following methods to control for confounding:
  o Studies controlling for time‐invariant unobservable confounding, including difference‐in‐differences, or fixed‐ or random‐effects models with an interaction term between time and intervention for pre‐intervention and post‐intervention observations; and
  o Studies assessing changes in trends in outcomes over a series of time points (interrupted time series, ITS), with or without contemporaneous comparison (controlled ITS), with sufficient observations to establish a trend and control for effects on outcomes due to factors other than the intervention (e.g., seasonality).
- Non‐randomised studies with control for observable confounding, including non‐parametric approaches (e.g., statistical matching, covariate matching, coarsened‐exact matching, propensity score matching) and parametric approaches (e.g., propensity‐weighted multiple regression analysis).

In a second stage, to address Questions 3 and 4 on factors related to intervention design, implementation and context, we extracted descriptive and qualitative data from the included experimental and quasi‐experimental studies. In addition, we conducted a targeted search for additional papers on the interventions covered by the included IEs to provide additional detail on these factors. We have included paper related to the intervention in the included IEs and corresponding to one or more of the following types of studies (Snilstveit, 2012):

- A *qualitative study* collecting primary data using mixed‐ methods or quantitative methods of data collection and analysis and reporting some information on all of the following: the research question, procedures for collecting data, procedures for analysing data, and information on sampling and recruitment, including at least two sample characteristics.
- A *descriptive quantitative study* collecting primary data using quantitative methods of data collection and descriptive quantitative analysis and report some information on all of the following: the research question, procedures for collecting data, procedures for analysing data, and information on sampling and recruitment, including at least two sample characteristics.
- A *process evaluation* assessing whether an intervention is being implemented as intended and what is felt to be working well, and why. Process evaluations may include the collection of qualitative and quantitative data from different stakeholders to cover subjective issues, such as perceptions of intervention success or more objective issues, such as how an intervention was operationalised. They might also be used to collect organisational information.
- A *project document* providing information about planned, ongoing or completed interventions. Such documents may describe the background and design of an intervention, or the resources available for a project. As such, these documents do not typically include much analysis of primary evidence, but they provide information about interventions. The purpose of including them in our review was to ensure we had sufficient information about the context and interventions in included studies.

#### 4.1.2 Types of participants

We included participants from fragile communities in low‐ and middle‐income countries (LMICs), including participants from the general population from all ages and genders and those from specific population sub‐groups, such as displaced populations, refugees, women, youthand so on. This wide definition of the population then allowed us to run subgroup analyses in our review such as rural and urban populations, socioeconomic groups or classes and castes, age, sex and so on.

##### 4.1.2.1 Challenges for the population definition

The definition of the population for this review raised a series of challenges to be considered:

- *The combination of women's empowerment and fragility criteria*: there is a gap in data available on both women's empowerment *and* fragile contexts. The team had run a scoping of indicators, indexes, and data available on women's empowerment in FCAS. The only index that covers both topics is the Women Peace and Security index designed by the University of Georgetown in 2017 but this index is not consensual in the research sphere as an indicator we could use on its own (Mundkur & Shepherd, 2018). Although this index is relevant to our SR, its time frame was not close enough to our start date of intervention (2000) to be used as the only criteria for inclusion. This gap then required the team to build its population criteria on the combination of existing indexes on gender *or* on FCAS.
- *The analysis of conflict affected situations at state‐level*: as presented in the previous sections, the study focuses on the micro‐ and meso‐ levels of the ecological framework. There are currently no indices that allowed us to draw our population selection on community and local levels indicators of fragility. Data on fragility are generally available at the State level with indexes such as the OECD and World Bank FCAS lists, the Fragile States Index (FSI) and the GPI. This gap then required the team to build its population criteria on State‐indicators although the analysis focused on lower levels. While the core team attempted to extract data from available international datasets on femicide, homicide and other SGBV‐related indicators, most datasets were incomplete, resulting in an inability to add any value to the analysis.
- *The potential size of the population*: the time extension and thematic focus would have made almost all LMICs eligible for inclusion in our SR. The resources available and timeframe would then not make it do‐able for the team and would have required the team to set a series of thresholds on the identified indexes to build its inclusion criteria. This then meant that some countries and regions have been prioritised for this study and that some excluded could have brought relevant studies for inclusion.

##### 4.1.2.2 Methodology of selection of the population

We created binary variables for whether a country was considered fragile or not, or gender unequal or not depending on the index, for each year for which data were available, using the thresholds noted above. We then created a series of consolidated lists for whether a country had appeared as fragile or unequal for at least 1 year during the period 2000–2020. We then grouped the countries into five sets, as follows:

- Set 1: There are 51 countries that appear on each of the World Bank, FSI^2^, OECD and Gender Inequality Index (GII)^3^ lists for at least 1 year.
- Set 2: There are 62 countries that appear on the GII and at least one of the fragility indices for at least 1 year (FSI, OECD or World Bank).
- Set 3: There are 23 countries that appear on the WPS list, but do not fall within Sets 1 or 2. This includes 10 countries that appear on the gender unequal GII list but none of the fragility indices, including contexts such as Brazil, El Salvador and India that were highlighted as potentially relevant contexts during the AG call. It further includes three countries that appear on the FSI list but not the GII index. Finally, it also includes contexts such as Mexico that were identified as of interest by members of the Advisory Group (AG) due to high levels of violence.
- Set 4: There are 11 countries that have appeared on one of the fragility indices since 2000, but not the GII or WPS.
- Set 5: There is one country that appeared on the GII list for at least 1 year, but never on the WPS or other fragility lists (Argentina).

We developed two options for addressing the risk that including all countries within Set 3 at the title and abstract (TA) stage could create an unrealistic scope during full‐text screening:

- Set 3a: restrict the threshold for the WPS index to <0.6 instead of <0.7. This would shrink Set 3 to three countries (Benin, Gabon and India). However, it would exclude the potential contexts highlighted by the AG as of interest.
- Set 3b: include only the 10 countries that show up on both the WPS and the GII (Benin, Botswana, Brazil, El Salvador, Gabon, India, Morocco, Paraguay, Peru and Saudi Arabia). This would effectively treat the WPS as a similar fragility index to the ones included within Set 2. From the above‐noted countries, it would exclude Mexico, but it may be the most realistic approach that is nonetheless drawn on consistent criteria.

##### 4.1.2.3 Inclusion criteria at title and abstract screening (TAS) stage

Based on the challenges identified above and the preferred options for the Sets 1, 2 and 3b, we included studies based on the following population criteria at TAS stage:

- LMICs that are listed on all our indicators: in the World Bank list of FCAS (since 2006), on the OECD list of FCAS (since 2007), on the FSI with a score higher than 90 (since 2009) and that have a score of at least 0.6 on the GII for at least 1 year since 2000.
- LMICs that are listed in one of the fragility lists or had at least 1 year rated higher than 80 on FSI and that have a score of at least 0.6 on the GII for at least 1 year since 2000.
- LMICs that do not appear on the fragility lists but are listed with a score lower than 0.7 on the Women Peace Security Index between 2016 and 2019 and that have a score of at least 0.6 on the GII for at least 1 year since 2000.

**Figure 4:**
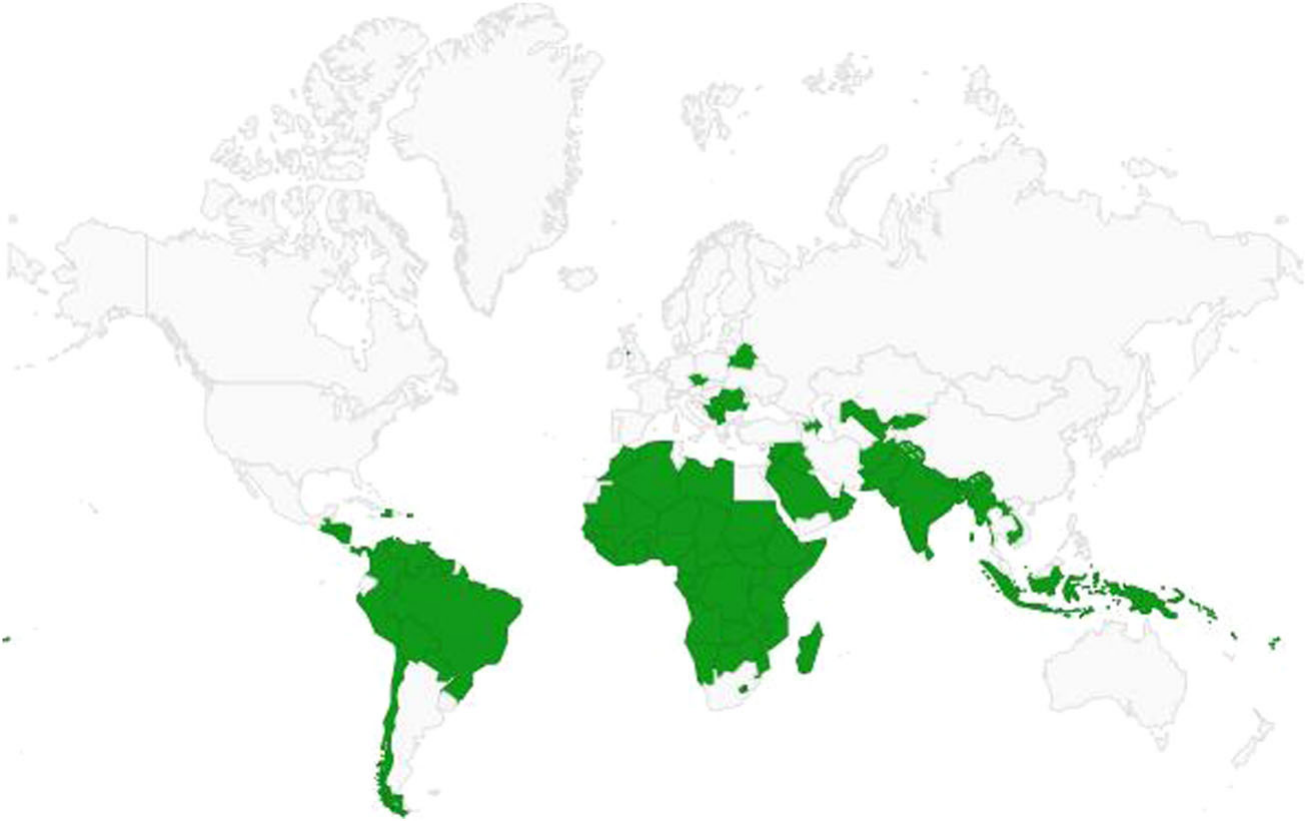
Map of includable countries for the systematic review

The above criteria allowed the team to build a list of included countries as below (Tables 6 and 11, Figure 4).

**Table 6:** List of included countries for the systematic review

Set	Countries	
Set 1	Afghanistan	Mali
There are 51 countries that appear on each of the World Bank, FSI, OECD and GII lists for at least 1 year	Angola	Marshall Islands
	Burkina Faso	Mauritania
	Burundi	Micronesia, Fed. Sts.
	Cambodia	Mozambique
	Cameroon	Myanmar
	Central African Republic	Nepal
	Chad	Niger
	Comoros	Nigeria
	Congo, Dem. Rep.	Papua New Guinea
	Congo, Rep.	São Tomé and Príncipe
	Côte d'Ivoire	Sierra Leone
	Djibouti	Solomon Islands
	Eritrea	Somalia
	Guinea	South Sudan
	Guinea‐Bissau	Sudan
	Haiti	Syrian Arab Republic
	Iraq	Timor‐Leste
	Kiribati	Tonga
	Kosovo	Tuvalu
	Lebanon	Uzbekistan
	Liberia	Vanuatu
	Libya	Venezuela, RB
	Madagascar	West Bank and Gaza
	Malawi	Yemen, Rep.
		Zimbabwe
Set 2	Algeria	Korea, Dem. People's Rep.
There are 62 countries that appear on the GII and at least one of the fragility indices for at least 1 year (FSI, OECD or World Bank)	American Samoa	Kyrgyz Republic
	Antigua and Barbuda	Lao PDR
	Azerbaijan	Lesotho
	Bahrain	Maldives
	Bangladesh	Montenegro
	Belarus	Namibia
	Bhutan	Nauru
	Bolivia	Nicaragua
	Bosnia and Herzegovina	North Macedonia
	Cabo Verde	Northern Mariana Islands
	Chile	Oman
	Colombia	Pakistan
	Czech Republic	Palau
	Dominica	Panama
	Egypt, Arab Rep.	Puerto Rico
	Equatorial Guinea	Romania
	ESwatini	Rwanda
	Ethiopia	Senegal
	Fiji	Serbia
	The Gambia	Seychelles
	Ghana	Sri Lanka
	Grenada	St. Kitts and Nevis
	Guatemala	St. Lucia
	Guyana	St. Vincent and the Grenadines
	Honduras	Suriname
	Indonesia	Tanzania
	Iran, Islamic Rep.	Togo
	Isle of Man	Trinidad and Tobago
	Jordan	Uganda
	Kenya	Zambia
Set 3b	Benin	India
This list includes countries that are not in the previous list and appear at least once in the WPS index also showed up on the GII for at least 1 year (similar to Set 2)	Botswana	Morocco
	Brazil	Paraguay
	El Salvador	Peru
	Gabon	Saudi Arabia

Abbreviations: FSI, Fragile State Index; OECD, Organisation of Economic Co‐operation and Development; WPS, Women Peace and Security.

##### 4.1.2.4 Inclusion criteria at full text screening (FTS) stage

Given constraints in time and scope, for the full‐text screening level, we developed a more nuanced operationalisation of fragility which considers the fact that fragility is not time constant, and decisions need to be made for each country within the context of the year in which the intervention took place. To do so, we applied the following inclusion criteria at full‐text screening:

- Does the country score over 0.6 on GII at the start date of the intervention or under 0.7 on the WPS for studies starting between 2014 and 2020 (if intervention start date not available use publication date)?
- Does the country score over 80.0 on FSI or is listed on WB or OECD list at the start date of the intervention (if intervention start date not available use publication date)? or, if after 2016, does the country belongs to the fourth or fifth quintile of the WPS index (under 0.700)?

We are aware of some of the limitations of the international indices measuring fragility levels. An important limitation for us has been the absence of an index allowing us to measure fragility at the community and local level. To address this limitation, we made the decision that if a study was not meeting the FSI, WB or OECD criterion we would look at whether the local level of implementation of the study was meeting our definition of fragility, human security, building peaceful and inclusive societies and overall relevant characteristics of our definition of developing *peaceful and inclusive societies*. A study meeting the characteristics of our definition at the local or community level could then be included on this criterion.

##### 4.1.2.5 Inclusion of refugee populations

Few populations are as inherently vulnerable, fragile, and exposed to conflict as refugees, including those that have been resettled as well as those that are living in camps. For that reason, this review has taken the decision to include any study whose explicit population is refugees from a country which would otherwise have been included and are living in a LMIC that would not be otherwise included. An example of that would be the evaluation of an intervention targeting Syrian refugees in Jordan (an LMIC country that would otherwise be excluded on the FCAS measure). Refugees living in a high‐income country (HIC) have not been included in the population in our review.

#### 4.1.3 Types of interventions

The main challenge of the definition of the intervention criteria for our SR is the wide range of interventions that fall under the four pillars of UNSCR 1325. In a similar way as for the population, including all the gender specific or transformative interventions that fall under the four pillars of UNSCR 1325 would be too broad. A set of exclusion grounds has then been agreed:

- We recognise the importance of the health sector for women's empowerment but considering the broad range of literature on this topics and existing research, health has not been included in the SR.
- We have not—focusing on the *treatment* of SGBV and only included studies focusing on preventing SGBV.
- We recognise the definition of the four pillars of UNSCR 1325 but have adopted a specific list of interventions under some pillars:
  o Participation: we have broadened the scope of the pillar to include interventions targeting economic, social and political participation.
  o Protection: we have narrowed inclusion to efforts to combat SGBV.
  o Recovery and relief: we have narrowed inclusion to reintegration intervention and DDR.

We used an intervention typology based on the four pillars to specify interventions groupings we included in our review. We will not repeat the descriptions of all interventions here, but Table 12 summarises included interventions under each pillar (Table 7).

**Table 7:** List of included interventions for the systematic review

Pillar	Intervention type
Participation	Civil society, associations and networks
	Land rights
	Formal and Nonformal education
	Technical and vocational education and training
	Financial inclusion
	MSMEs and entrepreneurship support
	Cash‐based approaches to support access to and participation in education and/or the economy
	Voice and participation in local and subnational governance and development bodies
	Community and leisure activities
	Civic education and youth leadership
Prevention	Conflict early warning systems
	Dialogue groups
	Community consultations
	Capacity building for conflict resolution
	Peace education
Protection	Legal rights education
	Behaviour change communication around support for women's rights and preventing SGBV
	Preventative protection measures
	Capacity building and technical support to subnational government officials to strengthen service provision for women and gender equality
	Gender‐sensitive policing
	Informal judicial system
Recovery and relief	(Re)integration of forcibly displaced populations
	Disaster risk reduction
	Disarmament, demobilisation and reintegration

Abbreviation: SGBV, sexual and gender‐based violence.

The following interventions do not meet the inclusion criteria and have been excluded from the review:

- *SGBV punishment*: we focused our work on proactive interventions for women's empowerment and gender equality and excluded reactive interventions focusing on the treatment of violence or inequality.
- *Formal peace processes*: this component would mainly focus on the higher levels of the ecological framework, and we only included the community and lower levels for inclusion.
- *Formal judicial system*: this group was excluded based on the ecological framework.
- *SGBV ‐ medical and psychosocial treatment and services for victims*: this group was excluded on the basis that Health‐focused intervention has not been included in this study and we have not focused on treatments of SGBV.
- *Family planning/reproductive rights*: this might be included if it was a component of a larger intervention but excluded it the intervention exclusively focuses on it.
- Capacity building of national‐level actors on the importance of and how to support women's empowerment and gender equality: this group is excluded based on the ecological framework.
- *Women's health*: health has not been covered in this review.
- *Education in emergencies*: Education has only been covered in the aspect of peace education but not general education.
- *Monetary policy*: these interventions are rarely gender‐specific and mainly are concentrated at the national/society level.
- *Cash‐based approaches to meet basic needs*: these might be included if it is a component of a larger intervention but excluded if the intervention exclusively focuses on it.
- *Humanitarian aid and emergency response*: this intervention type is too broad, and we have exclusively focused on conflict‐related emergencies.

Both our list of inclusion and exclusion are not exhaustive, and we therefore expect these lists to change based on the results of our search and screening process.

#### 4.1.4 Types of comparison

We included studies that compare the effects of an intervention aiming to empower women in fragile and/or conflict‐affected situations against similar situations where individuals, households and/or communities either received a ‘placebo’ intervention or ‘business as usual’ conditions.

#### 4.1.5 Types of outcome measures

The review takes a rights‐based approach that recognises the importance of gender equality as a priority in and of itself. Given the expected limited evidence for effects of gender equality programmes on outcomes of sustainable peace, the primary objective of the review is to understand the effects on women's empowerment and gender equality. Secondary objectives include understanding effects on outcomes of sustainable peace, human security, and wellbeing.

Given our use of Kabeer's (and Sen's) three‐pronged definition of women's empowerment, we developed a taxonomy of outcomes organised by category (Agency, Resources and Achievement), subcategory and specific outcome. The subcategories are organised as follows:

- We define Agency as the ability to act upon one's goals, but also be able to bring meaning, motivation and purpose to an activity. We divide the outcomes into the following sub‐categories:
  o Individual Agency
  o Community Level Agency
  o Institutions Supporting Agency
- We define Resources, under Kabeer's umbrella, broadly as defined to include not only access, but also future claims, to both material and human and social resources. We divide the outcomes into the following sub‐categories:
  o Access to Justice and Legal Services
  o Economic and Livelihoods Related Resources
  o Access to Employment
- We define Achievements as the capabilities that underlie both agency and resources, as a means of facilitating their procurement. We divide the outcomes into the following sub‐categories:
  o Improved Systems
  o Norms and Behaviour Change
  o Empowerment Index

The complete list of outcomes, their definitions and associated UNSCR 1325 pillars is found in Supporting Information Appendix SH.

##### 4.1.5.1 Duration of follow‐up

We have not included or excluded studies based on duration of follow‐up. If studies included multiple follow‐ups, we have included the outcomes measures most like those presented in the other studies included in any single meta‐analysis and report any additional follow‐ups narratively.

#### 4.1.6 Types of settings

We include interventions started between 2000 and 2020 conducted in FCAS in LMICs only. We have noted above that fragility varies over time and within states. To identify interventions implemented in fragile situations, we operationalised the following approach. First, to facilitate initial screening, we developed a list of eligible LMICs that have been classified as fragile according to one of a few state‐level lists For TAS criteria, we included studies where the population falls within at least one of the following categories:

- LMICs that are listed on all our indicators: in the World Bank list of FCAS (since 2006), on the OECD list of FCAS (since 2007), on the FSI with a score higher than 80 (since 2009) and that have a score of at least 0.6 on the GII for at least 1 year since 2000.
- LMICs that are listed in one of the fragility lists or had at least 1 year rated higher than 80 on FSI and that have a score of at least 0.6 on the GII for at least 1 year since 2000.
- LMICs that do not appear on the fragility lists but are listed with a score lower than 0.6 on the Women Peace Security Index between 2016 and 2019.

For FTS criteria, the call for inclusion has been made on a series of criteria depending on the characteristics of the population on the first year of implementation against the following indicators:

- LMICs status of the country of implementation
- GII score
- List of FCAS according to the FSI, World Bank and OECD
- Nuanced definition of fragile contexts applied in previous work
- Specific local context

An additional point of interest is the emphasis on FCAS, rather than nation‐states. As a result, countries that do not score within our inclusion criteria might still be included in the final list of studies due to a variety of reasons. As indices are often capturing national‐level data, as a research team we sought to recognise that sub‐nationally, the context may be varying including in situations such as refugee camps in non‐LMICs or rural, unstable regions in non‐fragile states.

#### 4.1.7 Other

We included both completed and ongoing studies, such as protocols of ongoing studies that appear to meet all other inclusion criteria or studies listed in registries of ongoing IEs.

We included studies published in any language, although search terms were in English only. We included studies published in 2000 or after, including interventions implemented in 2000 or after, taking the UNSCR 1325 as the starting point of our study.

### 4.2 Search methods for identification of studies

A comprehensive search of the literature for a SR on a topic in international development should cover key bibliographic databases, those specific to international development, those specific to social sciences and specific to the subject of the review (Kabeer & Waddington, 2015). The search strategy has been developed in collaboration with an information specialist and with reference to guidance in Hammerstrøm and colleagues (2010).

To capture the relevant literature as comprehensively as possible, we have developed both a general set of search terms and series of sub‐strategies and terms grouping around the typologies of the types of interventions included in our review, gender empowerment and gender equality, and building peaceful and inclusive societies. All searches were limited by the list of countries filters and by the year from 2000 onwards and integrated diverse range of literature: articles, IEs, reports, dissertations, conference documents and so on.

#### 4.2.1 Electronic searches

We searched the following academic databases:

- Africa‐Wide (Ebsco): https://www.ebsco.com/products/research-databases/africa-wide-information
- CAB Global health: https://www.cabi.org/publishing-products/global-health/
- Communication and Mass Media: https://www.ebsco.com/academic-libraries/subjects/communication-mass-media
- Econlit: https://www.aeaweb.org/econlit/
- Embase: https://www.embase.com/login
- Gender Studies: https://www.ebsco.com/products/research-databases/gender-studies-database
- International Political Science Abstracts: https://www.ebsco.com/products/research-databases/international-political-science-abstracts
- Medline: https://pubmed.ncbi.nlm.nih.gov/
- PsycInfo: https://www.apa.org/pubs/databases/psycinfo
- REpec: http://www.repec.org/
- Web of Science (SSCI): https://clarivate.com/webofsciencegroup/solutions/webofscience-ssci/
- World Bank e‐library: https://elibrary.worldbank.org/

#### 4.2.2 Grey literature searches

We searched the following specialist organisational databases:

- CARE International: http://www.careevaluations.org/
- Catholic Relief Services: https://www.crs.org/our-work-overseas/research-publications
- Centre for Public Impact: https://www.centreforpublicimpact.org/observatory/
- Chemonics International: https://www.chemonics.com/technical-areas/democracy-and-governance/
- EGAP (Evidence in Governance and Politics): http://egap.org/biblio
- IRC: https://www.rescue.org/reports-and-resources
- LSE ICG: https://www.theigc.org/search/?select-post_type%5B%5D=publication
- Mercy Corps: https://www.mercycorps.org/research
- Oxfam International: https://policy-practice.oxfam.org.uk/publications
- Search for Common Ground: https://www.sfcg.org/ilt/evaluations/
- FHI360: https://www.fhi360.org/
- World Vision: http://www.wvi.org/resources

Bilateral and multilateral agencies and general repositories of IEs in international development to be searched include:

- 3ie Repository of Impact Evaluations: https://developmentevidence.3ieimpact.org/
- 3ie RIDIE (Registry for International Development Impact Evaluations): http://ridie.3ieimpact.org/
- African Development Bank (AfDB): https://www.afdb.org/en/documents/publications/
- AgEcon: https://ageconsearch.umn.edu/?ln=en
- AGRIS: http://agris.fao.org/agris-search/index.do
- Asian Development Bank (ADB): https://www.adb.org/publications
- BREAD: http://ibread.org/bread/papers
- Center for Effective Global Action (CEGA): http://cega.berkeley.edu/evidence/
- CGIAR: Consultative Group on International Agricultural Research: https://cgspace.cgiar.org/handle/10568/83389
- Design, Monitoring and Evaluation for Peace: www.dmeforpeace.org/learn/resources/
- DEval: https://www.deval.org/en/home.html
- DFID Research for Development (R4D): http://r4d.dfid.gov.uk/
- GEF (Global Environmental Facility) evaluation database: http://www.gefieo.org/evaluations/all?f[0]=field_ieo_grouping%3A312
- Global Facility for Disaster Reduction and Recovery: https://www.gfdrr.org/en/publications
- ICNL Research Centre: http://www.icnl.org/research/library/ol/
- IFPRI: http://www.ifpri.org/publications
- Independent Development Evaluation, AfDB: http://idev.afdb.org/en/page/evaluations
- Innovations for Poverty Action (IPA): http://www.poverty-action.org/search-studies
- Inter‐American Development Bank Publications: https://publications.iadb.org/facet-view?locale-attribute=enandfield=type_view
- J‐Poverty Action Lab (J‐PAL): https://www.povertyactionlab.org/evaluations
- Locus (International Development Coalition): https://locus.ngo/resources
- OECD: http://www.oecd.org/derec/?hf=5andb=0ands=score
- UNEG Database of Evaluation Reports: http://www.uneval.org/evaluation/reports
- USAID: https://dec.usaid.gov/dec/search/SearchResults.aspx?q=KERvY3VtZW50cy5CaWJ0eXBlX05hbWU6KCgiU3BlY2lhbCBFdmFsdWF0aW9uIikgT1IgKCJGaW5hbCBFdmFsdWF0aW9uIFJlcG9ydCIpKSk=

Our methodology for the grey literature search followed a systematic approach:

1. First, a list of the most common variants of the intervention terms should be extrapolated based on any scoping work and existing studies for inclusion identified. Considering the broad aspect of our review we used a short set of terms related to gender equality and women's empowerment.
2. The strategy differs for sites with more or less than 300 resources. First, any relevant filters within the specific site should be applied (e.g., choosing only relevant document types, sectors, etc.).
  a. For repositories with more than 300 resources:
    i. Each search term is run alongside the methodology term ‘IE’ and ‘impact assessment’ for a total of 10 searches per site (women, men, girls, boys and gender).
    ii. Screen results until no potentially relevant results are identified on two consecutive pages of 50 search results (i.e., 100 excludes in a row).
  b. For repositories with fewer than 300 resources:
    i. Only the methodology term ‘IE’ is searched.
    ii. Screen all results.
3. For all sites, where possible, display settings are set to show 50 results per page, and the first page of results gets printed to PDF. Each set of PDFs gets saved in a folder with the site name and date of search, and the search data get recorded in a master Excel file as follows:
  a. For each website searched, the following information should be recorded:
    i. the date of the search;
    ii. the specific URL of the website searched (e.g., to differentiate between a search of publications vs. a search of programmes, which on J‐PAL's website will return different results);
    iii. the specific search terms used in each search (e.g., 1: IE; 2: ‘IE’; 3… etc.);
    iv. the number of hits for each search result (which can be recorded in a single cell with a formula, =[no. results from first search] +  [no. results from second search]+…+[no. results from nth search]);
    v. the number of potentially relevant documents identified and uploaded to EPPI, recorded by search as in (iv)
4. For each set of results, the titles and any abstract information shown on the main page are screened for potential inclusion, and if clearly irrelevant based on intervention, methodology or population, are ignored. If the title indicates possible relevance, click through to the full‐text and screen the abstract or executive summary.
5. Any result that appears of potential relevance gets downloaded and saved in the GLS shared folder for that site.
6. The documents collected from the GLS are then added to the project's EPPI environment.
  a. Create a new record in EPPI for each identified study.
  b. Key bibliographic information should be added: Title, all authors, year of publication, URL to the full text, and publisher. Abstract is not required.
  c. Once the bibliographic info is added, click ‘save’, which will then allow you to upload the full text.
  d. The study should be marked as ‘include’ at TAS and coding marked as completed (with the record open in EPPI, right‐click the heading for TAS, select ‘Properties’ then check the box next to ‘Coding completed’).
  e. The study should be marked as grey literature search under ‘origin’.

When information is not directly available on the listed website, we made use of Google Search (especially Google Scholar) for the retrieval of missing and/or additional references.

Given the breadth of our SR we are going to trial web scraping tools at the beginning of the search. We tried the use of a data *miner* to extract grey literature reference that would then be screened through EPPI:

- The data scraping was only tested on platforms presenting both the TA of the listed reports and resources
- The same approach as for the standard Grey Literature Method and save a PDF copy of the results of the search term
- Using data miner, we then extracted the data of the results pages to an excel template that would allow the upload of all the results on EPPI for TAS

We piloted this web scraping approach by testing it on similar websites to the standard method and assess the interest in scaling it up.

#### 4.2.3 Searching other resources

##### 4.2.3.1 General approach

We screened the bibliographies of included studies and existing reviews for additional eligible studies and conducted forward citation‐tracking of included studies in Google Scholar:

- Backwards citation search: Comb through the list of references in the study's full text and see if you can identify any potentially relevant studies from the titles, looking up any that look even possibly relevant to check their abstract.

Forwards citation search: We used Google Scholar to find the included paper, then under the entry, clicked the hyperlink where it says ‘Cited by X’ and screened the subsequent list of papers citing the included study.

This citation search applied to the studies shortlisted through the TAS and Grey Literature search and, considering the potential size of our sample, not to the retrieved literature and citations selected for analysis through the Full Text Screening. Considering the potential size of our sample we only did one level of citation search and did not do rounds of citation searches until no resources can be found.

We also published a blog post presenting our SR and calling for the provision of includable studies from the international development and humanitarian community. We also identified and contacted key researchers and organisations working in the relevant fields of our study for suggestions and engaged our AG for suggestions of relevant studies. We also hand‐searched journals of relevance to the review to identify papers that have not yet been indexed, covering issues published in the last 12 months. This hand‐search was also based on journals citing the included studies.

Titles and abstracts have been screened against the inclusion criteria and relevant records have been downloaded into the review management software EPPI reviewer. The initial screening of records was conducted by several reviewers screening the records from different databases. At this stage we have been over‐inclusive to ensure relevant studies are not omitted because sufficient information is not reported in title or abstract. Two reviewers then independently reviewed abstracts that have been judged to be potentially relevant at the first stage in more detail to determine which papers should be retrieved and reviewed at full text. Two reviewers then independently assessed full text studies for inclusion, with any disagreements resolved by a third reviewer.

##### 4.2.3.2 Targeted search for addressing review questions 3 and 4

Once we identified our set of included IEs, we undertook targeted searching for qualitative studies, process evaluations and project documents for those interventions evaluated in the included studies. We conducted citation tracking of included studies to identify relevant sister papers and conduct internet and database searches using the names of programmes from included studies. To identify relevant project documents, process evaluations and other qualitative studies, we conducted targeted searches of Google and Google Scholar as well as the funder and implementer websites of the identified programmes.

### 4.3 Data collection and analysis

#### 4.3.1 Description of methods used in primary research

Using the inclusion criteria set out in the previous sections, we anticipated that primary studies included in this review would use experimental or quasi‐experimental study designs and/or analysis methods to examine the extent to which changes in outcomes can be attributed to the intervention under study. To this end, we included randomised studies as well as non‐randomised studies that were able to suitably account for selection and confounding bias (Waddington et al., 2017).

#### 4.3.2 Criteria for determination of independent findings

Complex data structures are a common occurrence in meta‐analyses of IEs. There are several scenarios through which these complex structures with dependent effect sizes might occur. For instance, there could be several publications that stem from one study, or several studies based on the same data set. Some studies might have multiple treatment arms that are all compared to a single control group. Other studies may report outcome measurements from several time points or use multiple outcome measures to assess related outcome constructs. All such cases yield a set of statistically dependent effect size estimates (Borenstein et al., 2009).

The research team assessed the extent to which relationships existed across the studies included in the review. We avoided double counting of identical evidence by linking papers before data analysis. Where we have several publications reporting on the exact same effect, we used effect sizes from the most recent publication. We utilised information provided in studies to support these assessments, such as sample sizes, programme characteristics and key implementing and/or funding partners.

We extracted effects reported across different outcomes or subgroups within a study, and where information is collected on the same programme for different outcomes at the same or different periods of time, we extracted information on the full range of outcomes over time. Where studies report effects from multiple model specifications, we used the author's preferred model specification. If this is not stated or is unclear, we used the specification with the most controls. When studies reported multiple outcome sub‐groups for the same outcome construct, we calculated calculate a ‘synthetic effect size’ (Borenstein et al., 2009, chapter 24). Where studies reported multiple outcomes or evidence according to sub‐groups of participants, we recorded and reported data on relevant sub‐groups separately. Further information on criteria for determining independent effect sizes is presented below.

We dealt with dependent effect sizes through data processing and selection techniques, utilising several criteria to select one effect estimate per study. When we had several publications reporting on the same study, we used effect sizes from the most recent publication. For studies with outcome measures at different time points, we followed De La Rue and colleagues (2013) and synthesised outcomes measured immediately after the intervention (defined as 1–6 months) and at follow‐up (longer than 6 months) separately. If multiple time points exist within these time periods, we used the most recent measure. We anticipated many of the interventions we include in our review would be ongoing programmes and the follow‐up would, therefore, reflect duration in a programme rather than time since intervention. When such studies reported outcome measures at different time points, we identified the most common follow‐up period and included the follow up measures that match this most closely in the meta‐analysis. When studies included multiple outcome measures to assess related outcome constructs, we followed Macdonald et al. (2012) and selected the outcome that appears to most accurately reflect the construct of interest without reference to the results. If studies included multiple treatment arms with only one control group and the treatments represent separate treatment constructs, we calculated the effect size for treatment A versus control and treatment B versus control and included them in separate meta‐analyses according to the treatment construct. If treatments A and B represented variations of the same treatment construct, we calculated the weighted mean and standard deviation for treatment A and B before calculating the effect size for the merged group versus control group, following the procedures outlined in Borenstein and colleagues (2009, chapter 25). Where different studies reported on the same programme but use different samples (e.g., from different regions, or separately for men and women) we included both estimates, treating them as independent samples, provided effect sizes are measured relative to separate control or comparison groups.

#### 4.3.3 Selection of studies

We imported all search results into EPPI‐Reviewer 4 (Thomas & Brunton, 2010) and removed duplicates through the EPPI‐Reviewer 4 deduplication process. We double screened at TA for the first 10% of search results, including any studies we knew would be included, to train the machine learning (ML) algorithm. In this review, we took advantage of an innovative text‐mining ML capability of EPPI‐Reviewer 4 to reduce the initial screening workload: the inclusion/exclusion classifier (O'Mara‐Eves et al., 2015; Thomas et al., 2011).

We utilised the inclusion/exclusion classifier to organise studies into groups based on their probability of inclusion in the review. We conducted piloting and verification of the ML functioning and based on our experience in previous reviews, we expected to be able to exclude all studies with less than 20% probability of inclusion automatically from the review. We screened a random 10% sample of the automatically excluded studies to double check the accuracy of the function, and if all are excludable, we would auto‐exclude the rest. We would then double screen at TA all records with likelihood of inclusion at 20% or greater.

Where a study's TA do not include sufficient information to determine relevance, we included the study for review at full text. We double screened all studies flagged for full‐text review using two independent reviewers. We resolved disagreements on inclusion or exclusion by discussion with a core review team member and the input of an additional core reviewer if necessary. We assessed the results of the study‐specific key‐word searches for relevance, that is, whether they cover one of the programmes included to answer our research questions and whether they provide information on the design, implementation processes, context or mechanisms at play.

We also expected to identify multiple papers related to the same study. In this case, we used the Linked studies functionality of EPPI reviewer to identify the main study and other linked studies. The main study would be the study used for data extraction and the linked studies would complement the potential missing information of the main studies. To identify the main study, the priority was given to journal articles, in the case of multiple journal articles or only reports/working papers the most comprehensive was selected. In the case of equivalent quality of the paper, the most recent paper was selected.

#### 4.3.4 Data extraction and management

We extracted the following descriptive, methodological, qualitative and quantitative data from each included study using standardised data extraction forms (see Supporting Information Appendix SB):

- Descriptive data including authors, publication date and status, as well as other information to characterise the study including country, type of intervention and outcome, population and context.
- Methodological information on study design, analysis method and type of comparison (if relevant).
- Quantitative data for outcome measures, including outcome descriptive information, sample size in each intervention group, outcomes means and standard deviations, and test statistics (e.g., *t*‐test, *F*‐test, *p*‐values, 95% confidence intervals).
- Information on intervention design, including how the intervention incorporates participation, inclusion, transparency and accountability characteristics, participant adherence, contextual factors and programme mechanisms.

We extracted all quantitative, qualitative, descriptive, and methodological data using Excel. Descriptive and qualitative data was single coded by one reviewer and checked by a second reviewer. Two independent reviewers double coded quantitative data for outcomes analysis and risk of bias assessments, and any disagreement was resolved through discussion with a third reviewer (who was a core team member).

Once all effect sizes are calculated and converted to a standardised mean difference (SMD, as described in detail below), we examined the data for outliers. We defined outliers as any effect sizes ±3.29 standard deviations from the mean, following the guidance of Tabachnick and Fidell (2001), and utilized an outlier analysis on the entire data set during the data cleaning process to identify any potential errors. We examined sensitivity to outliers as discussed in the section on sensitivity analysis below.

For the qualitative analysis, we extracted detailed data on population characteristics, intervention design and implementation, and contextual variables (e.g., region, political climate) to address Questions 3 and 4.

#### 4.3.5 Assessment of risk of bias in included studies

We assessed the risk of bias in the included studies by drawing on the signalling questions in the 3ie risk of bias tool, which covers both internal validity and statistical conclusion validity of experimental and quasi‐experimental IE designs (Waddington et al., 2012). It includes the bias domains and extensions to Cochrane's ROBINS‐I tool and RoB2.0 (Higgins et al., 2016; Sterne et al., 2016). The risk of bias assessment helps us to determine the extent to which the findings in each study are reliable. Two reviewers undertook the risk of bias assessment independently. If there are disagreements, we resolved them by discussion and the involvement of a third reviewer (who must be a member of the core team), as necessary. The risk of bias tool can be found in Supporting Information Appendix SC. We did the risk of bias at the paper level.

We assessed risk of bias based on the following criteria, coding each paper as ‘Yes’, ‘Probably Yes’, ‘Probably No’, ‘No’ and ‘No Information’ according to how they address each domain:

- Factors relating to baseline confounding and biases arising from differential selection into and out of the study (e.g., assignment mechanism).
- Factors relating to bias due to missing outcome data (e.g., assessment of attrition).
- Factors relating to biases due to deviations from intended interventions (e.g., performance bias and survey effects) and motivation bias (Hawthorne effects).
- Factors relating to biases in outcomes measurement (e.g., social desirability or courtesy bias, recall bias).
- Factors relating to biases in reporting of analysis.

We reported the results of the assessment for each of the assessed criteria for each study. In addition, we used the results of the risk of bias assessments to produce an overall rating for each study as either ‘High risk of bias’, ‘Some concerns’ or ‘Low risk of bias’, drawing on the decision rules in RoB2.0 (Higgins et al., 2016), rating studies as follows:

- ‘High risk of bias’: if any of the bias domains were assessed as ‘No’ or ‘Probably No’.
- ‘Some concerns’: if one or several domains were assessed as ‘No Information’ and none were ‘No’ or ‘Probably No’.
- ‘Low risk of bias’: if all the bias domains were assessed as ‘Yes’ or ‘Probably Yes’.

In addition, we attempted to explore whether there are systematic differences in outcome effects between primary studies with different risk of bias. If meta‐analysis was feasible, we conducted sensitivity analysis to assess the robustness of the results to the risk of bias in included studies.

#### 4.3.6 Measures of treatment effect

An effect size expresses the magnitude (or strength) and direction of the relationship of interest (Borenstein et al., 2009; Valentine et al., 2015). We extracted data from each individual study to calculate standardised effect sizes for cross‐study comparison wherever possible. For continuous outcomes comparing group means in a treatment and control group, we calculated the SMD, or Cohen's *d*, its variance and standard error using formulae provided in Borenstein and colleagues (2009). A SMD is a difference in means between the treatment and control groups divided by the pooled standard deviation of the outcome measure. Cohen's *d* can be biased in cases where sample sizes are small. Therefore, in all cases we simply adjusted *d* using Hedges' method, adjusting Cohen's *d* to Hedges' *g* using the following formula (Ellis, 2010):

$$g≅d\left(1-\frac{3}{4(n_{1}+n_{2})-9}\right)$$

We chose the appropriate formulae for effect size calculations in reference to, and dependent upon, the data provided in included studies. For example, for studies reporting means (*X*) and pooled standard deviation (SD) for treatment (*T*) and control or comparison (*C*) at follow up only:

$$d=\frac{x_{Tp+1}-x_{Cp+1}}{\text{SD}}$$

If the study does not report the pooled standard deviation, it is possible to calculate it using the following formula:

$${\text{SD}}_{p+1}=\sqrt{\frac{(n_{Tp+1}-1){\text{SD}}_{Tp+1}^{2}+(n_{Cp+1}-1){\text{SD}}_{Cp+1}^{2}}{n_{Tp+1}+n_{Cp+1}-2}}$$

Where the intervention was expected to change the standard deviation of the outcome variable, we used the standard deviation of the control group only.

For studies reporting means ($\underline{X})$ and standard deviations (SD) for treatment and control or comparison groups at baseline (*p*) and follow up (*p* + 1):

$$d=\;\frac{\Delta {\underline{X}}_{p+1}-\Delta {\underline{X}}_{p}}{{\text{SD}}_{p+1}}$$

For studies reporting mean differences ($∆\underline{X})$ between treatment and control and standard deviation (SD) at follow up (*p* + 1):

$$d=\frac{{∆\underline{X}}_{p+1}}{{\text{SD}}_{p+1}}=\;\frac{{\underline{X}}_{\mathrm{Tp}+1}-{\underline{X}}_{\mathrm{Cp}+1}}{{\text{SD}}_{p+1}}$$

For studies reporting mean differences between treatment and control, standard error (SE) and sample size (*n*):

$$d=\frac{{∆\underline{X}}_{p+1}}{\text{SE}\sqrt{n}}$$

As primary studies have become increasingly complex, it has become commonplace for authors to extract partial effect sizes (e.g., a regression coefficient adjusted for covariates) in the context of meta‐analysis. For studies reporting regression results, we followed the approach suggested by Keef and Roberts (2004) using the regression coefficient and the pooled standard deviation of the outcome. Where the pooled standard deviation of the outcome is unavailable, we used regression coefficients and standard errors or *t*‐statistics to do the following, where sample size information is available in each group:

$$d=\;t\sqrt{\frac{1}{n_{T}}+\frac{1}{n_{C}}}$$

where *n* denotes the sample size of treatment group and control. We used the following where only the total sample size information (*N*) is available, as suggested in Polanin et al. (2016):

$$d=\frac{2t}{\sqrt{N}}{\text{Var}}_{d}=\frac{4}{N}+\frac{d^{2}}{4N}$$

We calculated the *t*‐statistic (*t*) by dividing the coefficient by the standard error. If the authors only reported confidence intervals and no standard error, we calculated the standard error from the confidence intervals. If the study does not report the standard error, but report *t*, we extracted and used this as reported by the authors. In cases in which significance levels are reported rather than *t* or SE (b), then *t* was imputed as follows:

Prob>0.1:t=0.5

0.1≥Prob>0.05:t=1.8

0.05≥Prob>0.01:t=2.4

0.01≥Prob:t=2.8

Where outcomes are reported in proportions of individuals, we calculated the Cox‐transformed log odds ratio effect size (Sánchez‐Meca et al., 2003):

$$d=\frac{\ln(\text{OR})}{1.65}$$

where OR is the odds ratio calculated from the two‐by‐two frequency table.

Where outcomes were reported based on proportions of events or days, we used the standardised proportion difference effect size:

$$d=\frac{p_{T}-p_{C}}{\text{SD}(p)}$$

where *p*_t_ is the proportion in the treatment group and *p*_c_ the proportion in the comparison group, and the denominator is given by:

$$\text{SD}(p)=\sqrt{p(1-p)}$$

where *p* is the weighted average of *p*_c_ and *p*_t_:

$$p=\frac{n_{T}\;p_{T}+n_{C}\;p_{C}}{n_{T}+n_{C}}$$

An independent reviewer evaluated a random selection of 10% of effect sizes to ensure that the correct formulae were employed in effect size calculations. In all cases after synthesis, we converted pooled effect sizes to commonly used metrics such as percentage changes and mean differences in outcome metrics typically used (e.g., weight in kg) whenever feasible.

#### 4.3.7 Unit of analysis issues

Unit of analysis errors can arise when the unit of allocation of a treatment is different to the unit of analysis of effect size estimate, and this is not accounted for in the analysis (e.g., by clustering standard errors at the level of allocation). We assessed studies for unit of analysis errors (The Campbell Collaboration, 2020), and where they exist, we corrected for them by adjusting the standard errors according to the following formula (Hedges et al., 2010; Higgins & Thomas, 2020; Waddington et al., 2012):

$${\left(d\right)}^{\prime }=(d)\times \sqrt{1+(m-1)c}$$

where *m* is the average number of observations per cluster and *c* is the intra‐cluster correlation coefficient. Where included studies used robust Huber–White standard errors to correct for clustering, we calculated the standard error of *d* by dividing *d* by the *t*‐statistic on the coefficient of interest.

#### 4.3.8 Dealing with missing data

In cases of relevant missing or incomplete data in studies identified for inclusion, we contacted study authors to obtain the required information. If we were unable to obtain the necessary data, we reported the characteristics of the study but state that it could not be included in the meta‐analysis or reporting of effect sizes due to missing data.

#### 4.3.9 Assessment of heterogeneity

We assessed heterogeneity by calculating the *Q*‐statistic, *I* ^2^ and $\tau^{2}$ to provide an estimate of the amount of variability in the distribution of the true effect sizes (Borenstein et al., 2009). We complemented this with an assessment of heterogeneity of effect sizes graphically using forest plots. Additionally, we explored heterogeneity using moderator analysis in meta‐regression specifications where there were at least four studies and significant heterogeneity. While some have suggested 10 studies as a minimum for moderator analysis, as Borenstein and colleagues (2009) note, there are no hard and fast rules. However, we ensured that for categorical moderators, there were a minimum of two effects per cell.

#### 4.3.10 Assessment of reporting biases

To reduce the possibility of publication bias, we searched for and included unpublished studies in the review. We also tested for the presence of publication bias using funnel plots and statistical tests. Specifically, the rank correlation test (Begg & Mazumdar, 1994) and the regression test (Sterne & Egger, 2005), using the standard error of the observed outcomes as predictor, were used to check for funnel plot asymmetry.

#### 4.3.11 Data synthesis

We conducted meta‐analyses of studies that we assess to be sufficiently similar. The inclusion criteria for the review were broad and we anticipated including studies that report on a diverse set of interventions, sectors and outcomes. It was therefore difficult to predict how meta‐analysis would be used in the review prospectively. However, the minimum criteria were to only combine studies using meta‐analysis when we identify two or more effect sizes using a similar outcome construct and where the comparison group state is judged to be similar across the two, similar to the approach taken by Wilson et al. (2011). We combined studies in the same analysis when they evaluate the same intervention type, or the same outcome type. Moderator analyses can take into account multiple interventions as moderator variables, allowing us to also examine the impact of different intervention types by outcome. Where there were too few studies, or included studies are considered too heterogeneous in terms of interventions or outcomes, we presented a discussion of individual effect sizes along the causal chain. As heterogeneity exists in theory due to the variety of interventions and contexts included, we used inverse‐variance weighted, random effects meta‐analytic models (Higgins & Thomas, 2020).

We used the metafor package (version 2.4.0; Viechtbauer, 2010) in R software to conduct the meta‐analyses (version 4.0.4; R Core Team, 2020). The amount of heterogeneity (i.e., $\tau^{2}$), was estimated using the DerSimonian–Laird estimator (DerSimonian & Laird, 2015). In addition to the estimate of $\tau^{2}$, the Q‐test for heterogeneity (Cochran, 1954) and the $I^{2}$ statistic (Higgins & Thompson, 2002) are reported.

We conducted separate analyses for the major outcome categories for each intervention group: primary outcome (women's empowerment, gender equality), secondary outcome (well‐being, security, peace) and other important outcomes such as changes in knowledge (of rights), attitudes and behaviours, tolerance of violence, self‐confidence, self‐efficacy. Based on an analysis of the interventions that we find, we attempted to further elaborate on the pathway of change that was outlined above to the extent possible. We also used sub‐group analysis to explore heterogeneity by different treatment sub‐groups (described in more detail in the section on subgroup analysis and investigation of heterogeneity).

We also collected qualitative information from studies about the interventions. This information may subsequently be coded quantitatively to be used in moderator analysis. It may also be used to classify intervention mechanisms in synthesis or in the further development of intervention causal chains. These characteristics may include: intervention objectives (to change processes, behaviours or both); whether interventions are strategic (complex, adaptable strategy to realise change) or tactical (tool‐based); the source of intervention (local, NGO, government or researcher‐led); the scale of the intervention (pilot experiment vs. adoption of formal policy/law); extent to which members of both targeted groups are engaged (equally or primarily one group); and initial power differences between the groups targeted.

#### 4.3.12 Subgroup analysis and investigation of heterogeneity

Whenever feasible, we conducted moderator analyses to investigate sources of heterogeneity. Following the PROGRESS‐PLUS approach (Gough et al., 2017), we assessed moderators falling into three broad categories of extrinsic, methodological and substantive characteristics to address inequity aspects within the gender equality context. Examples of these categories include:

- Extrinsic characteristics: funder of the study (e.g., NGO vs. private sector vs government investments), publication type, publication date.
- Methodological characteristics: study design, risk of bias, study quality characteristics, evaluation period, length of follow‐up.
- Substantive characteristics: participant characteristics (gender, age, Socioeconomic status, education, land ownership), context (geographical setting, market access), intervention type, intervention features, type of implementing agency.

We used random effects meta‐regression to investigate the association between moderator variables and heterogeneity of treatment effects (Borenstein et al., 2009) and sub‐group analyses to investigate heterogeneity by treatment sub‐groups (e.g., men and women, poor and nonpoor, and so on). If the latter strategies were not possible (i.e., if we do not have enough studies or data), we discussed and explored the factors which may be driving heterogeneity of results narratively by conducting cross‐case comparisons (Miles & Huberman, 1994).

The qualitative analysis also included the following subgroups: women's age, education, marital status, health (currently pregnant or not), ethnically tudentized group, race and caste. For groups, heterogeneity by mixed‐sex groups and women‐only groups. If possible, esex of service/intervention frontline provider.

#### 4.3.13 Sensitivity analysis

We conducted sensitivity analysis to assess whether the results of the meta‐analysis were sensitive to the removal of any single study. We did this by removing studies from the meta‐analysis one‐by one and assessing changes in results. We also assessed sensitivity of results to inclusion of high risk of bias studies by removing these studies from the meta‐analysis and comparing results to the main meta‐analysis results. Studentised residuals and Cook's distances are used to examine whether studies may be outliers and/or influential in the context of the model (Viechtbauer & Cheung, 2010). Studentised residuals express the difference between the predicted effect size (based on the entire body of evidence in the analysis) and the observed effect size for any given study. These are a standard diagnostic tool for outliers in meta‐analysis. Studies with a tudentized residual larger than the 100×(1−0.05/(2×k)) the percentile of a standard normal distribution are considered potential outliers (i.e., using a Bonferroni correction with two‐sided α=0.05 for k studies included in the meta‐analysis). Studies with a Cook's distance larger than the median plus six times the interquartile range of the Cook's distances are influential.

#### 4.3.14 Treatment of qualitative research

As a mixed‐methods SR, we included a distinct review component to synthesise qualitative evidence on the review Questions 3 and 4. While the identification of qualitative evidence is limited to studies linked to the included IEs, the process of data extraction, critical appraisal and evidence synthesis is independent and follows methods and guidelines tailored to the conduct of qualitative evidence synthesis (Noyes et al., 2020). This approach to mixed‐methods synthesis of development interventions builds on Snilstveit and colleagues (2015, 2019).

##### 4.3.14.1 Assessment of quality in descriptive quantitative studies, qualitative studies and process evaluations

We assessed the quality of included qualitative studies, process evaluations, and descriptive quantitative studies using a mixed‐methods appraisal tool developed by Langer and colleagues (2017) and applied in Snilstveit and colleagues (2019). This tool builds on the Critical Appraisal Skills Programme checklist (Brice, 2006) and Pluye and colleagues' (2011) mixed‐methods appraisal tool and is provided in Supporting Information Appendix SA. Our appraisal tool made judgments on the adequacy of reporting, data collection, presentation, analysis and conclusions drawn. The appraisal assessed the quality of the included qualitative studies and descriptive quantitative studies using six appraisal domains:

1. The defensibility of the applied research design to answer the research question under investigation.
2. The defensibility of the selected research sample and the process of selecting research participants.
3. The rigour of the technical research conduct, including the transparency of reporting.
4. The rigour of the applied analysis and credibility of study's claims given the nature of the presented data.
5. The consideration of the study's context (for qualitative studies only).
6. The reflexivity of the reported research (for qualitative studies only).

We filtered out studies of particularly low quality at this stage, using a fatal flaw approach following Dixon‐Woods et al. (2005). Studies that did not meet either criterion of appraisal domains 1–4 above were excluded from the synthesis. That is, they were included in the review, and we reported on the studies' descriptive data, for example, applied intervention. However, no research findings were extracted from these studies to feed into the review's synthesis. Each appraisal domain was assessed from a scale of critical trustworthiness to low, medium and high trustworthiness. An overall critical appraisal judgement per study was allocated using a numerical threshold of the appraised quality domains (Supporting Information Appendix SA).

We did not undertake a critical appraisal of included project documents. They typically provide information about planned, ongoing or completed programmes, providing information about the design or resources available for a project for instance. As such these documents did not typically include much analysis of primary evidence, but they provided information about interventions. The purpose of including them in our review was to ensure we had sufficient information about the context and interventions included. We therefore focused the appraisal on assessing the relevance of the documents against the interventions assessed in our review. Before extracting any data, we ensured that the name of the intervention, the implementing agency, context and timeline of the intervention described in the project document corresponds to the intervention assessed in the IE included in our review. Finally, collecting data from a range of sources, especially if used for triangulation, can enhance confidence in the trustworthiness of the information included. If several sources were available, we extracted data from all sources for purposes of triangulation.

##### 4.3.14.2 Methods for qualitative evidence synthesis (applicable to review questions 3 and 4)

To address Questions 3 and 4, we complemented any statistical meta‐regressions with a qualitative evidence synthesis (Noyes et al., 2020). After having completed the detailed coding of all of the included studies as described above, we assessed the coding of data on factors related context, intervention design and implementation, and population characteristics for the most relevant qualitative synthesis approach. The identified approach to qualitative evidence synthesis aligns and is informed by the review's overall ToC as outlined in Figure 2. We expected thematic synthesis (Thomas & Harden, 2008) and qualitative comparative analysis (Rihoux, 2006; Thomas et al., 2014) to present two relevant approaches to the qualitative synthesis in this review. Following a review of the included qualitative evidence base, we opted for thematic synthesis as the most suitable synthesis method.^4^

##### 4.3.14.3 Thematic synthesis

This qualitative evidence synthesis approach was applied as we had sufficient in‐depth qualitative studies and empirical primary data reported across the identified evidence‐base and linked to groups of interventions and outcomes along the review's ToC. Its objective was to identify analytical themes on intervention mechanisms and contexts that mitigate or reinforce intervention effects to complement the review's statistical moderator analysis and/or meta‐regression. Following Thomas and Harden's (2008) thematic synthesis, we used inductive coding techniques to first identify common descriptive themes based on the reported findings of the primary studies. We used EPPI‐Reviewer's coding software to illustrate the link between the inductive codes in the primary studies and the identified descriptive themes. In a second step, following the identification of descriptive themes, these then were configured into higher level analytical themes, which present the results of the thematic synthesis. Again, this configuration from descriptive to analytical themes was conducted in EPPI‐Reviewer and we produced an overview table of both types of themes and their linkages for transparency in this final synthesis step. The process of configuring descriptive and analytical themes from the inductive coding applied the same consistency checks as the general data extraction process outlined above. The process of generating inductive codes, descriptive themes, and final analytical themes was configured around four analytical lenses derived from the research questions 3 and 4 of this review. These refer to the interplay of context, intervention design, intervention implementation, and population characteristics with programme effects, and are outlined in more detail below:

I. *Context*: Any variable related to external factors beyond the programme's control that affect programme impact. This can refer to political factors such as types of governance, societal factors such as norms, economic factors such as a recession, and cultural factors such as beliefs.
II. *Intervention design*: any variable that is related to the design and planning of the applied intervention. Design and planning of an intervention refers to the blueprint or schedule of the intervention and will typically outline what components the intervention consists of and in what sequence they will be applied. Examples of design variables refer to: size or type of cash transfer; outreach strategy, posters; reminders; type of training.
III. *Intervention implementation*: any variable that is related to the implementation of the intervention in practice. This refers to variables that emerge while the intervention is applied and are usually not known in advance. Examples of implementation variables refer to the lack of attendance or uptake, payment difficulties, corruption, elite capture.
IV. *Population characteristics*: any variable related to the population targeted by the intervention or the population in which the effects are measured (in cases where these differ). This can refer to the Socioeconomic status of the population, its educational status and asset ownership.
V. *Interplay with programme effect*: extracted data and codes need to relate to the programme effect, outcome or impact. That is, we did not extract descriptive data on the intervention design, implementation, context, and population—we were only interested in data that reports on how variables in these four categories are affecting programme effects.

#### 4.3.15 Confidence in cumulative bodies of evidence

To summarise our confidence in the cumulative body of evidence, we used the Grading of Recommendations Assessment, Development and Evaluation (GRADE) approach for confidence in quantitative bodies of evidence on intervention effects (Guyatt et al., 2008), and we explored use of the GRADE‐CERQual (Confidence in the Evidence from Reviews of Qualitative research) approach for confidence in qualitative bodies of evidence on barriers, facilitators, and moderators of intervention effectiveness (Lewin et al., 2018).

##### 4.3.15.1 GRADE approach for quantitative bodies of evidence

We used the GRADE approach to rate our confidence in the body of evidence for each intervention contrasts on all primary and secondary outcomes. We specifically rated our confidence that the true effect of an intervention (vs. a comparator) on a given outcome lies on one side of the line of no effect, or ‘difference from the null’ (Hultcrantz et al., 2017). Accordingly, our ratings referred to our confidence in the existence (or not) of intervention effects and the direction of effects; they not referred to our confidence in the magnitude of effects. There are four possible levels of confidence:

- High confidence: It is highly likely that the intervention does (not) have an effect on the outcome of interest.
- Moderate confidence: It is likely that the intervention does (not) have an effect on the outcome of interest.
- Low confidence: It is possible that the intervention does (not) have an effect on the outcome of interest.
- Very low confidence: It is not clear whether the intervention does (not) have an effect on the outcome of interest.

We followed the traditional GRADE approach, in which evidence from RCTs starts at high confidence, while evidence from all other study designs starts as low confidence. We assessed the body of evidence according to four factors that can lead to a downgrading confidence in the evidence by one level (serious concern) or two levels (very serious): limitations in individual studies (risks of bias), inconsistency of results, imprecision and publication bias. We did not downgrade based on indirectness of the evidence, the reasons for which are addressed thoroughly in the *Limitations Related to the Use of GRADE* section. We then assessed the body of evidence according to three factors that can lead to an upgrading confidence in the evidence: large magnitude of an effect, a dose–response gradient, and the effect of plausible residual confounding. We followed recent guidance on how to consider these factors when adopting a complexity perspective in a SR on intervention effects (Montgomery et al., 2019), such as using sources of intervention complexity to explain substantial heterogeneity and using information on intervention implementation to inform assessments of a dose–response gradient.

We presented and explained the results of our GRADE ratings in summary of findings tables (Santesso et al., 2016). In addition, we described our GRADE ratings narratively using language approved by the GRADE Working Group (Santesso et al., 2020):

- High confidence: The intervention reduces/increases the outcome.
- Moderate confidence: The intervention probably reduces/increases the outcome.
- Low confidence: The intervention may reduce/increase the outcome.
- Very low confidence: We are very uncertain about the effect of the intervention on the outcome.

Finally, researchers ranked the importance of different bodies of evidence by applying a framework inspired Maslow's hierarchy of needs to different outcome types as follows:

- Critical: All outcome groups that are directly related to the physical wellbeing of participants, including those related to security, IPV, protection, child‐marriage, and so on.
- Some importance, but not critical: All outcome groups related to basic needs, including economic security and livelihoods.
- Limited importance: All other measures of empowerment including capacity, autonomy, attitudes, political representation, and so on.

##### 4.3.15.2 Confidence in the body of evidence included in the qualitative review component

We explored the relevance and feasibility of conducting a confidence in the body of evidence assessment for the qualitative review component. The decision to conduct this assessment depended on the size and nature of the included qualitative evidence‐base as well as the contribution of the qualitative review findings to the stated research questions. If relevant and feasible, we aimed to apply GRADE‐CERQual (Lewin et al., 2018) to conduct the confidence of the evidence assessment. However, given the narrow contribution of the qualitative review component, which is an explanatory supplement to the quantitative findings, we opted against conducting a confidence of the evidence assessment for the qualitative component. As the qualitative review does not search for an independent body of qualitative evidence, and only focused on qualitative evidence linked to the included IE, its external validity is reduced by design limiting the contribution of the CERQual application.

## 5 RESULTS

### 5.1 Description of studies: Search results and characteristics of the evidence base

In this chapter, we report descriptive results for the review to provide an overview of the characteristics and distribution of the evidence base across all interventions covered in the review. We start by providing the results of the search and screening of the literature followed by a section providing a summary of the characteristics of included studies.

#### 5.1.1 Results of the search

##### 5.1.1.1 Search process

**Figure 5:**
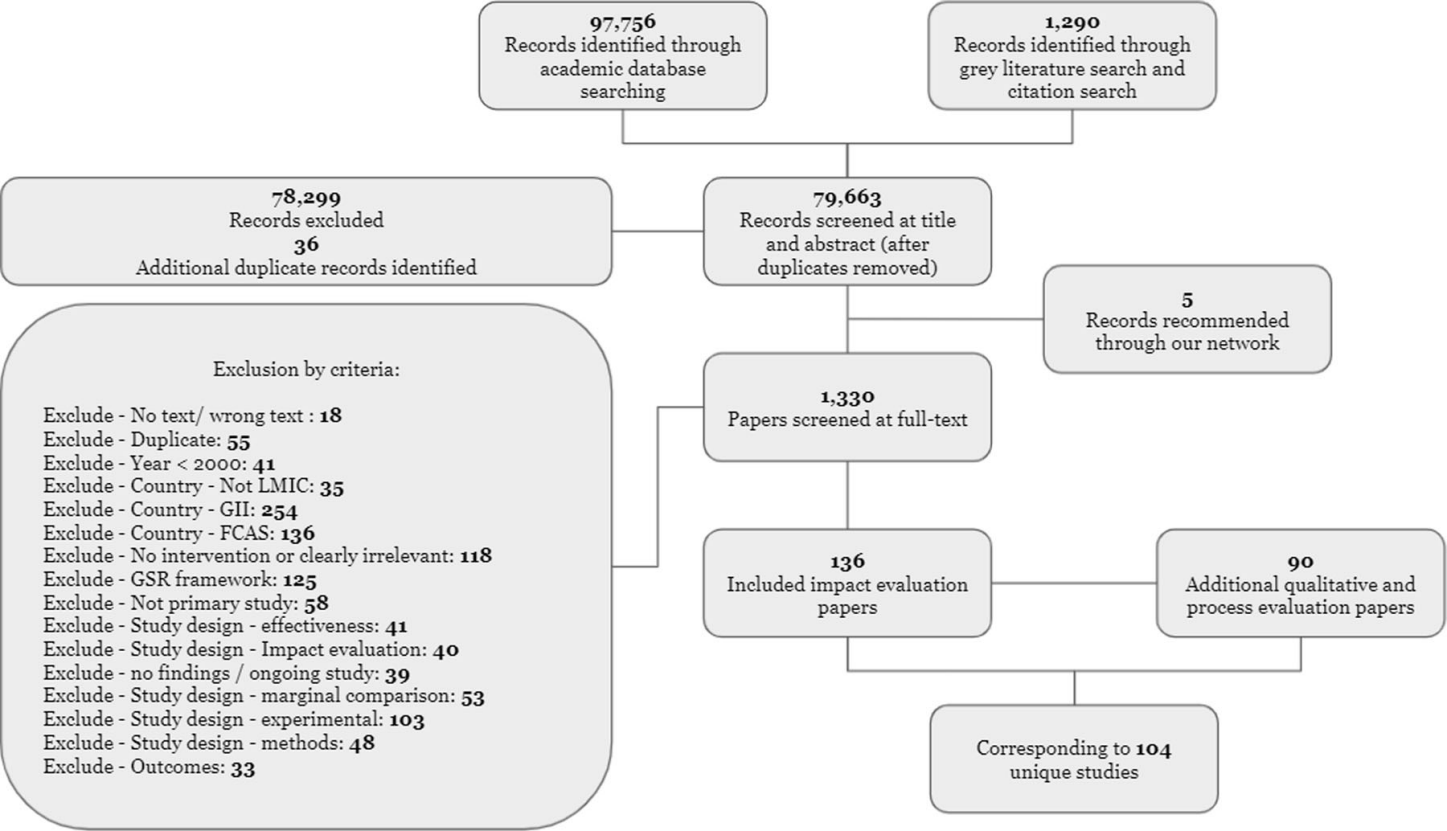
PRISMA flow diagram of systematic search and screening results

The PRISMA flow diagram below (Figure 5) details the results of the search and screening process, following guidance from reporting in SRs (Page et al., 2021). The initial academic search identified 97,756 studies. Hand searches of relevant grey literature, citation searches, journal searches and additional suggestions brought 1290 results. Following the removal of duplicates, a total of 79,663 studies were left for screening at TA. TAS identified 1330 potentially relevant records for inclusion in the review, which were then screened at full text. The full‐text screening ultimately identified 136 IE papers, corresponding to 104 studies of 55 programmes for inclusion in this review. In addition, 39 ongoing studies, or without published findings, were also identified that appear likely to meet inclusion criteria. Although not included in the review, these are listed in the Supporting Information Appendix SJ. There are also an additional 90 papers with complementary qualitative components that underwent qualitative extraction.

The number of included papers is larger than the number of included studies. This is because it is common for studies to be reported in more than one paper, typically one or more working papers and a journal version. As noted in our methodology, our approach was to make the most recent version of the journal article the main paper, and then include any other version(s) of the paper which contained additional information. Typically, working papers and other ‘unpublished’ reports included more detail than journal versions, such as effects on additional outcomes, sub‐groups or more details about the programme and would therefore be included.

##### 5.1.1.2 Reasons for exclusion

The main reasons for exclusion at TA stage were:

- The country of focus of the study was not included;
- The study had no intervention or was not relevant for our study: meaning that the intervention was not focusing on gender or, if focusing on gender, was not gender specific or gender transformative; or
- The study was not included through our study framework: the focus was outside of our ecological level or on a non‐includable intervention type.

Our flow chart (Figure 5) provides a detailed breakdown of the reasons for exclusion at full text. The most common reason for excluding studies at full text was that they did not our criteria for country inclusion either by not scoring enough (or having a highly relevant context) on GII (*n* = 254) or by not being a FCAS (or having a highly relevant context) on the first year of intervention (*n* = 136). The exclusion on the ground of no intervention or clearly irrelevant (*n* = 116) or GSR framework (*n* = 125) was still high because some cases of exclusion from TA required more details from the full text to decide. The absence of a valid counterfactual was the main reason for exclusion by study design. In addition to full‐text exclusion, 18 texts were not able to be retrieved after three rounds of full‐text retrieval. Fifty‐five duplicates were identified within the included and excluded studies and removed. The list of all studies excluded at full text with reasons for exclusion is available in Supporting Information Appendix SJ.

However, many studies that could have otherwise been included in this review were excluded based on study design. With quantitative methods, we looked for rigorous methods that included a counterfactual. Reporting on baseline was not present in many studies; without this data it was difficult to ascertain changes between baseline and the reporting of results. Overall, while this review is significantly larger than many existing SRs on this topic, the breadth of interventions were concentrated in specific interventions and outcomes, with other interventions lacking consistent or sufficient data to be used in quantitative analysis.

##### 5.1.1.3 Inclusion of process evaluations and qualitative studies

The qualitative study identification process involved three stages of work namely searching, screening, and critical appraisal. The search and screening for qualitative studies as indicated in the methods sections yielded 420 relevant results that meet the inclusion criteria for qualitative studies. The conducted searches were related to each unique programme identified in the included IEs, though multiple studies may have covered the same programme. The reasons for exclusion used for the screening process were limited to document type, for example, excluding project PowerPoint Presentations, newspaper articles, budgetary reports and other informal reports.

The 420 identified results referred to qualitative studies, descriptive quantitative studies, process evaluations, and projects documents. Project documents did not undergo the critical appraisal process. Typically, project documents provide information about planned, ongoing or completed programmes as well as, for instance, details about the design or resources available for a project. Consequently, project documents did not contain the relevant analysis of primary evidence, but they provided information about interventions. They were retained in the review to ensure there was sufficient information about the context and interventions included and consulted as background information in the qualitative synthesis. In total, 109 project documents were identified and excluded from the critical appraisal process.

This left a total of 311 studies for critically appraisal. Following the critical appraisal of these studies, a total of 90 qualitative, descriptive quantitative and process evaluation studies were included for the data extraction process (see Section 4.1.2 for more detail).

#### 5.1.2 Characteristics of included studies

##### 5.1.2.1 General characteristics of included studies

The search and screening process resulted in 104 unique studies. A single evaluation study was often reported in more than one report. In this case, the reviewed 104 studies were reported in 136 evaluation reports, inclusive of 32 linked publications.

**Figure 6:**
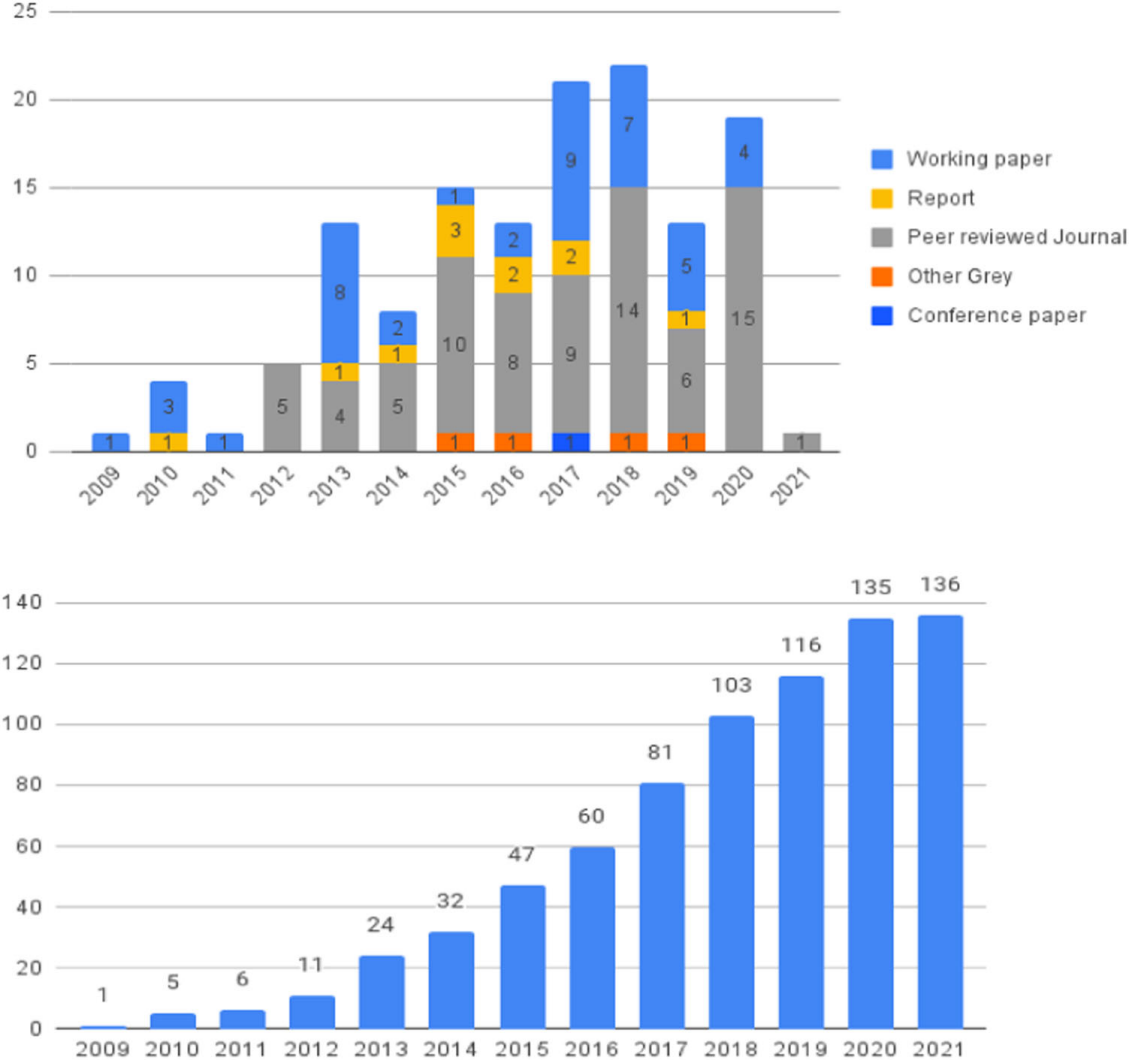
Annual (panel A, top) and cumulative (panel B, bottom) number of included publications over the review period from 2000 to 2021

In line with this review's specific focus on interventions that commenced on or after 2000, the earliest included publication was Legovini (2005) and the most recent was Leight et al. (2021). The annual number of publications saw a steady increase over the years with 89 (65% of the total) published between 2016 and 2021. 2018 ranked the highest, with 22 publications. Figure 6 panel A presents the number of publications, classified by types, while panel B presents the cumulative number of publications over the period under consideration.

**Figure 7:**
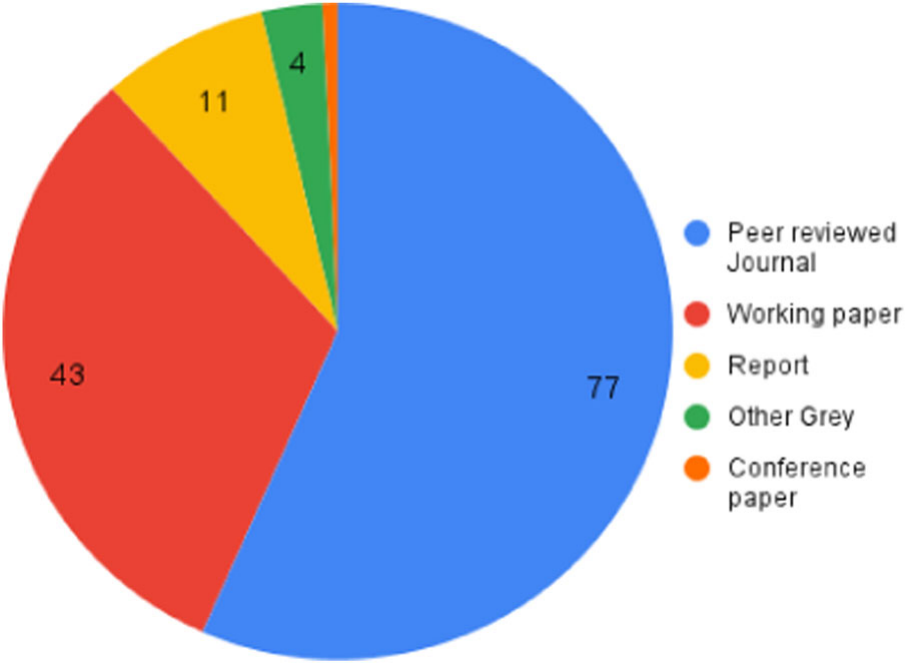
Types of included publications

Of the 136 included publications, 77 were published in peer‐reviewed journals. Employing systematic search for grey literature and citation searches of the included publications, this review also includes 43 working papers and 11 organisational reports. Other publications included intervention implementation documents, conference papers and book or book chapters. Figure 7 presents the types of included publications.

##### 5.1.2.2 Geographies of included studies

**Figure 8:**
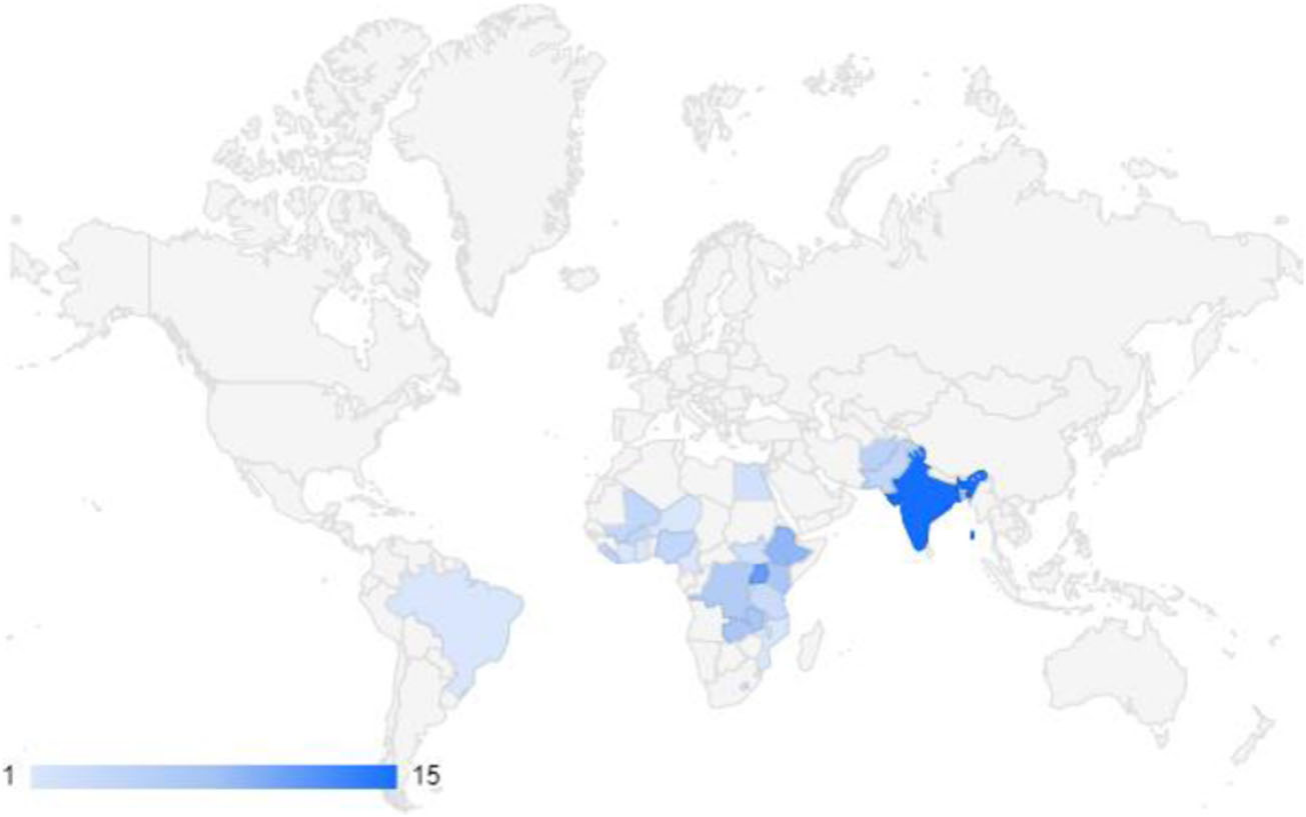
Map of countries of included studies

**Figure 9:**
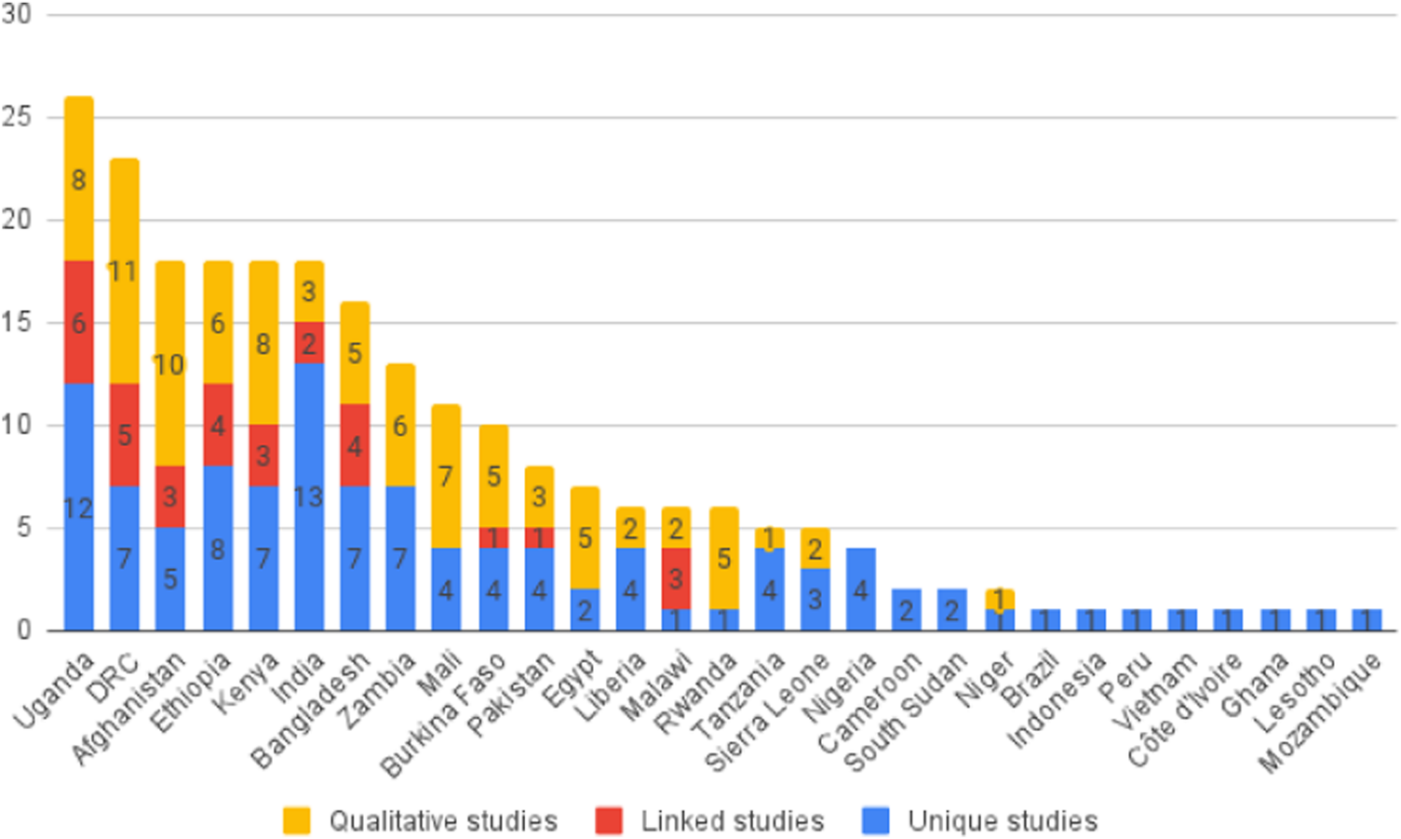
Studies per country

**Figure 10:**
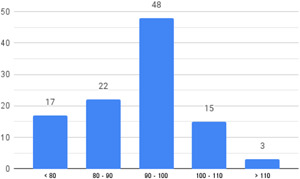
Study Fragile States Index scores

The included unique studies retained after the screening process were geographically diverse (Figures 9 and 10) and representative of several fragile contexts around the globe. Most of the studies originated in Southeast Asia and Sub‐Saharan Africa, with the highest number of studies coming from India. Other regions represented include East Asia and the Pacific, Latin America and Caribbean and the Middle East and North Africa (Figure 8).

The inclusion criteria for our review specified lower and lower‐middle income countries only. Given this, we saw 75 studies from low‐income countries and 35 studies from lower‐middle income countries, with some studies occurring across multiple countries (Figures 9 and 10).

**Figure 11:**
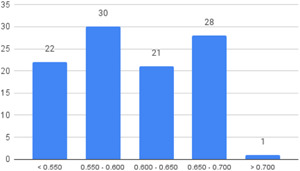
Study Gender Inequality Index scores

Additionally, the studies were situated in a variety of contexts that differ in terms of gender equity, fragility and human security. We evaluated the distribution of study contexts on the included dimensions. All but five included studies scored above 0.5 on the GII, with 50% scoring at or above 0.6 (Figure 11). On the FSI, the highest number of studies (*n* = 47) scored between 90 and 100 (Figure 10). Twenty‐two studies were classified as FCAS by the World Bank while 65 studies were classified as the same by the OECD. Lastly, out of the 11 studies for which we were able to gather WPS Index data, five studies scored below 0.6.

#### 5.1.3 Types of interventions of included studies

In this section, we describe the interventions, including their components, duration and target populations. We also discuss the ways in which the interventions aimed to build gender equality and women's empowerment towards peaceful and inclusive societies.

While we began the extraction process with 27 intervention groups, the scope of literature quickly revealed that there was significant overlap. After several sessions of brainstorming and discussion, the team reduced it to 14 groups. Table 13 provides an overview of the intervention categories and included studies (Table 8).

**Table 8:** Summary of included studies per intervention type^a^

Intervention group	Included studies—country
*Participation pillar*		
Asset transfer (*n *=* *14)	Ahmed et al. (2009)—Bangladesh
	Bandiera et al. (2017)—Bangladesh
	Bedoya et al. (2019)—Afghanistan
	Bedoya et al. (2020)—Afghanistan
	Glass et al. (2017)—Democratic Republic of the Congo
	Nkonya et al. (2012)—Nigeria
	Olney et al. (2015)—Burkina Faso
	Pradhan and Sulaiman (2017)—Bangladesh
	van den Bold et al. (2015)—Burkina Faso
	Roy et al. (2019)—Bangladesh
	Bandiera et al. (2013)—Bangladesh
	Das et al. (2013)—Bangladesh
	Roy et al. (2015)—Bangladesh
	Roy et al. (2013)—Bangladesh
Cash transfer (*n *=* *30)	Ambler and De Brauw (2017)—Pakistan
	Baird et al. (2010)—Malawi
	Blattman et al. (2016)—Uganda
	Bonilla et al. (2017)—Zambia
	Breisinger et al. (2018)—Egypt
	Brooks et al. (2020)—Kenya
	Clark et al. (2019)—Kenya
	Eze (2019)—Cameroon
	Gelagay and Lecoutere (2019)—Ethiopia
	Gobin et al. (2017)—Kenya
	Green et al. (2015)—Uganda
	Handa et al. (2015)—Kenya
	Haushofer and Shapiro (2018)—Kenya
	Heath et al. (2020)—Mali
	Iqbal et al. (2020)—Pakistan
	Karimli et al. (2021)—Burkina Faso
	Malaeb and Uzor (2017)—Uganda
	Mekonnen (2017)—Ethiopia
	Müller et al. (2019)—South Sudan
	Natali et al. (2016)—Zambia
	Sarah et al. (2017)—Malawi
	Baird et al. (2021)—Malawi
	Baird et al. (2013)—Malawi
	Blattman et al. (2013)—Uganda
	Green et al. (2016)—Uganda
	Haushofer et al. (2019)—Kenya
	Haushofer and Shapiro (2013)—Kenya
	Haushofer and Shapiro (2016)—Kenya
	Iqbal et al. (2020)—Pakistan
	Karimli et al. (2019)—Burkina Faso
Community‐based services (*n *=* *1)	Nandi et al. (2020)—India
Inclusive community‐driven development (*n *=* *7)	Beath et al. (2013a, 2013b)—Afghanistan
	Beath et al. (2015)—Afghanistan
	Larson et al. (2018)—Brazil, Cameroon, Vietnam
	Laudati et al. (2018)—DRC
	Beath et al. (2013b)—Afghanistan
	Van der Windt et al. (2018)—DRC
	Mvukiyehe and van der Windt (2020)—DRC
Institutional provision of loans and savings (*n *=* *8)	Tarozzi et al. (2013)—Ethiopia
	Tarozzi et al. (2015)—Ethiopia
	Duflo et al. (2013)—India
	Banerjee et al. (2015)—India
	Ifelunini and Wosowei (2012)—Nigeria
	Olajide et al. (2016)—Nigeria
	Johnson (2018)—Liberia
	Weber and Ahmad (2014)—Pakistan
Quotas (*n *=* *2)	Beaman et al. (2012)—India
	Clayton (2015)—Lesotho
Self‐help groups and VSLAs (*n *=* *13)	Alemu et al. (2018)—Ethiopia
	Bass et al. (2016)—Democratic Republic of the Congo
	Beaman et al. (2014)—Mali
	De Hoop et al. (2010)—India
	De Hoop et al. (2014)—India
	Deininger and Liu (2013)—India
	Desai and Joshic (2013)—India
	Ismayilova et al. (2018)—Burkina Faso
	Karlan et al. (2017)—Ghana, Malawi, Uganda
	Kundu and Mukherjee (2011)—India
	Lombardini and Yoshikawa (2015)—Uganda
	Vigneri and Lombardini (2017)—Mali
	Yaron et al. (2018)—India
Technical and Vocational Education and Training (TVET) (*n *=* *12)	Adoho et al. (2014)—Liberia
	Amin et al. (2016)—Bangladesh
	Bandiera et al. (2012)—Uganda
	Bandiera et al. (2018)—Uganda
	Croke et al. (2017)—Nigeria
	Gibbs et al. (2020)—Afghanistan
	Lecoutere (2018)—Uganda
	Lombardini and Bowman (2015)—Pakistan
	Maitra and Mani (2017)—India
	Mckenzie et al. (2019)—Kenya
	Bandiera (2018)—Sierra Leone
	Gibbs et al. (2018)—Afghanistan
*Prevention pillar*		
Community dialogues and reconciliations (*n *=* *2)	Cilliers et al. (2018)—Sierra Leone
	Figueroa et al. (2016)—Mozambique
*Protection pillar*		
All‐women police stations (*n *=* *1)	Jassal (2020)—India
Sensitisation campaigns (*n *=* *9)	Abramsky et al. (2014)—Uganda
	Das et al. (2012)—India
	Decker et al. (2020)—Malawi
	Dunkle et al. (2020)—Rwanda
	Green et al. (2020)—Uganda
	Quisumbing et al. (2020)—Bangladesh
	Schensul et al. (2015)—India
	Green et al. (2018)—Uganda
	Watts et al. (2015)—Uganda
*Multi‐pillar*		
Discussion groups (*n *=* *5)	Hossain et al. (2014)—Cote d'Ivoire
	Lubega et al. (2017)—Uganda
	Mvukiyehe (2017)—Liberia
	Vaillant et al. (2020)—DRC
	Vaillant (2016)—DRC
Life, social and livelihood skills and capacity building (*n *=* *26)	Ashraf et al. (2018)—Zambia
	Bastian et al. (2018)—Tanzania
	Buehren, Chakravarty, et al. (2017)—South Sudan
	Croke et al. (2020)—DRC
	Chinen and Elmeski (2016)—Uganda
	Decker et al. (2018)—Malawi
	Field et al. (2010)—India
	Gottlieb (2016)—Mali
	Halim et al. (2019)—Tanzania
	Lecoutere and Wuyts (2021)—Uganda
	Leight et al. (2021)—Ethiopia
	Murray et al. (2020)—Zambia
	Noble et al. (2019)—Afghanistan
	Fuller (2014)—Sierra Leone
	Özler et al. (2020)—Liberia
	Scales et al. (2013)—Bangladesh
	Stark, Seff, et al. (2018)—DRC
	Stark, Asghar, et al. (2018)—Ethiopia
	Tanner and O'Connor (2017a)–DRC
	Tanner and O'Connor (2017b)—Ethiopia
	Lecoutere (2018)—Uganda
	Sharma et al. (2020)—Ethiopia
	Stark, Asghar, et al. (2018)—Ethiopia
	Tanner and O'Connor (2017a, 2017b)—DRC, Ethiopia
	Kumar et al. (2018)—Zambia
Safe spaces (*n *=* *6)	Buehren, Goldstein, et al. (2017)—Tanzania
	Erulkar and Medhin (2017)—Ethiopia
	Sieverding and Elbadawy (2016)—Egypt
	Mercy Corps (2015)—Niger
	Austrian and Muthengi (2014)—Uganda
	Austrian et al. (2020)—Zambia

^a^ Italics are linked studies.

**Figure 12:**
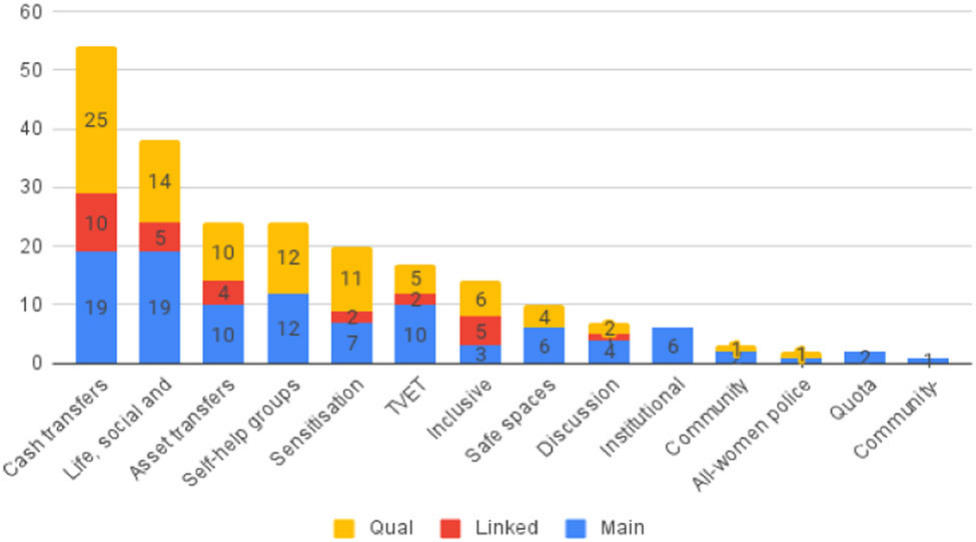
Distribution of intervention types. TVET, technical and vocational education and training

Figure 12 provides a list of all included interventions and the corresponding number of studies identified for included.

**Figure 13:**
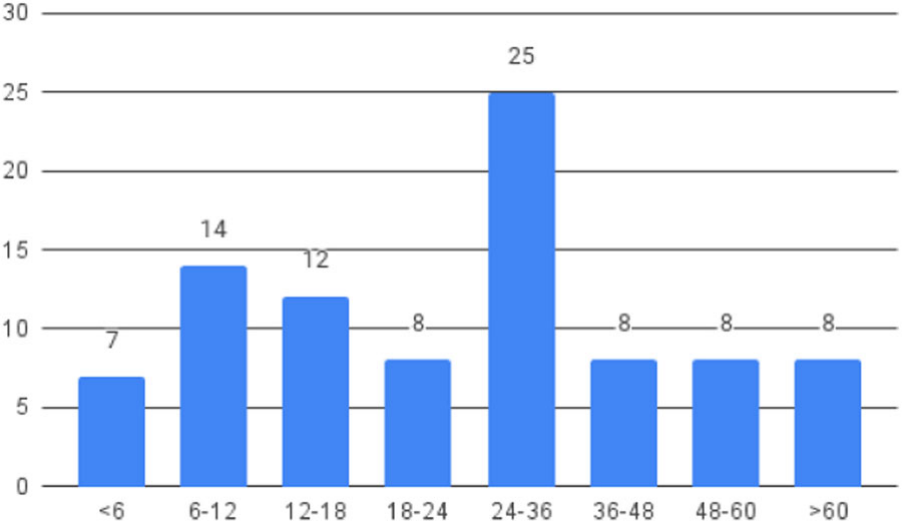
Intervention length (months)

The focus of the intervention types aligned with the UNSCR Pillars covered by our studies: The Participation pillar was the most frequently occurring pillar in our sample (*n* = 96) followed by prevention (*n* = 31), protection (*n* = 15) and recovery and relief (*n* = 5). As demonstrated in Figure 13, the diversity of the types of interventions were also illustrated by the range of duration of intervention from 0.5 months to over 140 months, although most of our studies (*n* = 74) had an intervention length below 48 months.

The ecological framework, or targeting level, of studies was primarily at the individual level (*n* = 78). Studies would often focus on both the individual and household level or integrate community‐level (*n* = 35) interventions for broad‐based interventions. The household (*n* = 32) was also a key location for interventions. Women are the target group of most of our studies (*n* = 84), but men were also a key target group (*n* = 33) for our studies, either through their involvement in cross‐group interventions or through targeted interventions engaging directly with men for gender equality and women's empowerment. This is followed by girls (*n* = 29), with only a minority of studies specifically focusing on boys (*n* = 5). Our studies also highlight a gap in literature on interventions focusing on nonbinary individuals. This further corroborates the lack of studies overall in LGBTQIA+ communities in fragile contexts.

We observed a lack of reporting on the interventions funding (to be distinguished from the study funding) since approximately one‐third of the included studies do not report on the source of funding of the intervention (*n* = 40). The main sources of intervention funding were governments (*n* = 35) and multilateral organisations (*n* = 16), followed by NGOs (*n* = 14), foundations (*n* = 11) and private sources (*n* = 7). The teams of the study and the intervention were often distinct since a vast majority of interventions were implemented by an implementing agency (*n* = 72) and only a minority were implemented by researchers (*n* = 9).

##### 5.1.3.1 Equity dimensions of included studies

A key aspect of studies was equity. Equity is the absence of avoidable and unfair conditions between or among people that hinder or prevent them from attaining their full potential. It is inherently a moral and socioeconomic judgment of fairness. Often, vulnerable populations have intersecting equity concerns, such as gender, socioeconomic class, race, ethnicity and religious belief.

The focus on gender specific and transformative interventions resulted in 100% (*n* = 104) of all included unique studies demonstrating an equity dimension. We evaluated studies on eight different measures of equity, including the disaggregation of data by sex or other sub‐group analysis, equity sensitive analytical frameworks or ToC, equity‐sensitive methodologies, targeting specific vulnerable population(s), measuring effects on a gender or equity outcome, as well as a research process informed by gender or equity.

The most common equity markers were vulnerable population(s) targeted (*n* = 90). As mentioned earlier, women were the largest target group. Because women tend to have intersecting identities and vulnerabilities that are compounded by those identities, most studies were placed into this category. Equity sensitive analytical framework (*n* = 85) and equity sensitive methodology (*n* = 82) follow closely behind. For example, multiple studies, including looked at differential impacts on social and structural inequalities, such as the stratification of inequality towards indigenous versus nonindigenous women. Other IEs include equity‐driven theories of change that consider gendered social relations in a community or household and how they may reinforce or change gender norms and roles. This is coupled with a focus on an equity sensitive research process (*n* = 66) that considers the safety and bias of potential respondents when collecting data. For example, Abramsky et al. (2014) ensured that women's security in treatment communities for the SASA! programme was prioritised during data collection.

Measuring effects on an inequality outcome was frequent (*n* = 66), as the outcomes of interest for this review (gender equality and women's empowerment, as well as human security outcomes) automatically were inclusive of the concept of inequality. When interventions targeted both men and women or boys and girls, data was disaggregated by sex (*n* = 48), though a smaller portion of studies had disaggregation by sub‐groups, including caste, ethnicity and age. Lastly, approximately one‐third of the included studies (*n* = 33) reported having ethical approval from an institutional review board or other governing body.

We also evaluated 19 different equity dimensions that go beyond the demographics of study participants but look at specific factors or dimensions of a study population. Primarily mentioned (*n* = 97) is sex, including the use of gender denoting biological sex, followed by place of residence (*n* = 80), as economic empowerment and financial inclusion studies tended to focus on rural or informally settled populations.

Additionally, studies stratified findings by age (*n* = 55) to look at the differential impacts on adolescents or young adults, including educational level/social capital (*n* = 36) and whether the treatment group is the head of household (*n* = 18). Conflict‐affected (*n* = 24) situations were also common, as studies conducted in post‐conflict situations or ongoing war contexts were applicable. Socioeconomic status (*n* = 63), including income or poverty status and social capital were most common in economic‐focused studies that took participants' socioeconomic status into account.

Less mentioned dimensions or not appearing altogether included people living with HIV/AIDS (PLHIV), land size, ownership, disability, culture, refugees and religion, as well as sexual orientation and identity. The latter did not appear in any studies despite the inclusion of an additional search strategy for papers using LGBTQIA+ search terms.

##### 5.1.3.2 Study funding and independence

Study funding and independence refers to funding of the included papers, the evaluations and the independence of study authors to the programmes being evaluated. Funding for included studies came from five sources: public institutions (*n* = 51), private institutions (*n* = 4), multilateral organisations (*n* = 12), foundations (*n* = 21) and NGOs (*n* = 9). Seventeen remaining studies did not provide information on their funding and two were not funded. Several studies were funded through multiple sources.

Independence of a study was defined by two variables: the relationship between the implementing agency and the study team, and whether the data collected by an independent party. For 55 studies, the funding and author team were independent of implementers/funders of the programme. In eight cases, the funding was independent of implementers/funders, but authors of the study were from the programmes' funder/implementers. Ten studies were not independent since the evaluation was funded and undertaken by funders/implementers of the programme being evaluated. Finally, 29 cases did not have clear enough data to verify independence.

For whether the data had been collected by an independent party, 46 studies reported independence in data collection, 13 did not have independently collected reported data and 43 did not provide enough information to form a decision.

We also searched for whether there were any issues with conflict of interest. This meant searching for whether there was any declaration of interest from the study authors. For most studies (*n* = 63), there was not enough information to make any clear decision, and in 31 there was no conflict of interest. Eight studies had a clear conflict of interest, whether due to the fact the study was an internal evaluation, or that evaluators were associated with international organisations. All studies (*n* = 104) were published in English.

##### 5.1.3.3 Implementation of study protocol of included studies

In this section, we describe the studies, including their components, duration and implementation characteristics. We also discuss the ways studies reported on generalisability of their findings and achievements of the ToC of the programme.

Our studies were assessed through three components:

- Reporting on adherence to project and programme activities: refers to the actual participation of local population and target groups to the activities of the programme. This can be notably reviewed through the level of attrition in the programme or project: it refers to the loss of study units from a sample over the course of the programme or project. Did the beneficiaries take part in the project over the implementation period?
- Reporting on uptake of project and programme activities: refers to the level of engagement of the beneficiaries to the activities of the project. Did the beneficiaries take active part in the activities during and after the course of the project?
- Reporting on the fidelity of implementation of project and programme activities: refers to the level of implementation of the study protocol by the study and/or intervention teams. Did the teams implement the intervention and study as initially planned?

**Figure 14:**
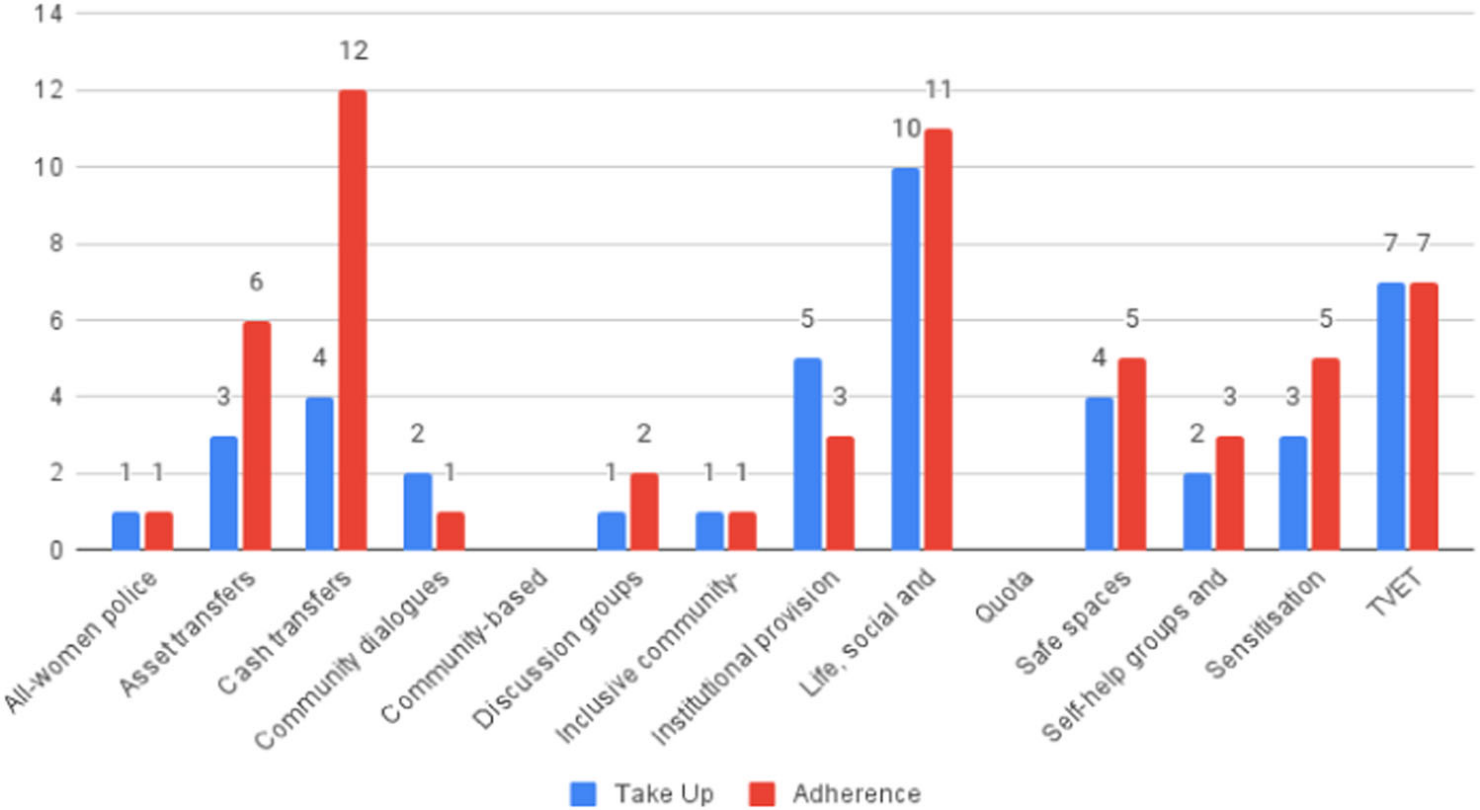
Reporting on uptake and adherence per intervention type. TVET, technical and vocational education and training

Approximately half of the studies reported on programme adherence (*n* = 56) but only 26 formally assessed programme adherence. Among these, the main reporting methods were commentaries (i.e., mentioned without formal analysis) by the authors (*n* = 40) and/or observation by implementation staff (*n* = 42). Only 39 studies reported uptake, and formally assessed by 15. Once again, when reported, uptake is covered through commentaries by the author (*n* = 24) and/or observation by the implementation staff (*n* = 28) (Figure 14).

This adherence and uptake could be encouraged by the frequency of contact since most of the studies' activities were conducted on a weekly (*n* = 37) or monthly basis (*n* = 24), followed by daily contact (*n* = 15). A vast majority of the studies were 40 months or less in duration (*n* = 70) and only three were 60 months or longer. Incentives were rarely used to drive adherence and uptake; only 16 studies clearly reported using incentives and the use of incentives was not clear for 43 studies. We observed that uptake and adherence were often reported together and not always distinguished within studies.

Implementation fidelity, to the contrary, was rarely covered in our studies (*n* = 30) and only three studies formally assessed it. This is not the result of a lack of reporting on the programme characteristics and information since 68 studies make an explicit reference to ToC of the programme to design their study protocol. A hypothesis on low reporting of implementation fidelity may be that most of our studies were peer journal articles where other information was prioritised.

A non‐negligible proportion of our sample of included studies provided analysis not only on the effectiveness of the interventions but also on the causal mechanism, barriers and enablers that drive the success or failure of interventions (*n* = 46). This reporting was primarily done through commentaries by the author (*n* = 33). Sixty‐five studies covered questions of generalisability of their findings. On the other hand, we observed that only 25 of our studies reported on cost, which appears to contrast with the fact that a high portion of our interventions focused on financial inclusion and cash‐based support.

Another element to note is that a vast majority of the included studies measure the impact of intervention directly after the end of the intervention: 66% (*n* = 86) of the included studies have a follow‐up period of less than a month, 9% (*n* = 12) have a follow‐up period of 1–6 months, 10% (*n* = 13) of 7–12 months, 1% (*n* = 1) of 13–18 months, 2% (*n* = 3) of 19–24 months, and only 3% (*n* = 4) over 25 months.

##### 5.1.3.4 Outcomes

Our outcome framework was based on the three dimensions of women's empowerment and gender equality designed by Kabeer for a total of 44 unique outcomes as per Table 14 (Table 9).

**Table 9:** List of outcomes and number of studies covering them

Outcome categories	Outcomes
Resources (*n *=* *71) material, human and social resources which serve to enhance the ability to exercise choice	Access to justice and legal services
	AA1 Women have access to rights, services and opportunities (*n *=* *3) AA2 Increased employment of women in law and justice sector agencies (*n *=* *1) AA3 Women have improved access to labour rights (*n *=* *0)
	Economic livelihood related resources
	AB1 Increased capacity of women to understand and use financial, banking, and business services effectively (*n *=* *15) AB2 Women have increased access and ownership to assets, credit and income (*n *=* *64) AB3 Women and girls have equitable access to livelihood support services (*n *=* *3) AB4 Women and men benefit equitably from employment and livelihood opportunities during recovery and reconstruction (*n *=* *0) AB5 Durable and reliable housing for vulnerable populations, including women and girls (*n *=* *1)
	Access to employment
	AC1 More women engaged in other micro, small and medium‐sized enterprises (*n *=* *6) AC2 Women can access decent work (formal and informal employment) (*n *=* *27) AC3 Improved capacity of women entrepreneurs (*n *=* *12)
Agency (*n *=* *87) ability to define one's goals and act upon them and operationalised decision‐making	Individual agency
	BA1 Women have improved success in the workplace (*n *=* *3) BA2 Women have more and better control over their bodies and sexual health (*n *=* *21) BA3 Women have increased freedom of movement and association (*n *=* *28) BA4 Women are more aware of their rights and the roles and responsibilities of duty bearers (*n *=* *10) BA5 women have more positive attitude towards taking action to claim their rights (*n *=* *16) BA6 Reduced percentage of women agreeing with certain reasons that justify violence against women and girls (*n *=* *19) BA7 Women are equipped with better life skills that allow them to be prepared for crisis or shocks and recover from them (*n *=* *22) BA8 Reduced support for or occurrence of child and forced marriage (*n *=* *12)
	Community level agency
	BB1 Increased participation in decision making by Women at the household or community level, including during crisis response (*n *=* *48) BB2 Women participate more in their community (*n *=* *14) BB3 Increasingly women‐led organisations have the capacity or greater capacity to actively engage and influence other actors (*n *=* *0) BB4 Women increasingly form their own organisations (*n *=* *2)
	Institutions supporting agency
	BC1 The rights, safety, and security of women, men, girls, and boys are protected during relief, recovery, and reconstruction (*n *=* *1) BC2 Increased capacity of women to protect themselves from rights violations and advocate for and monitor service delivery including during relief, recovery, and reconstruction (*n *=* *0) BC3 The rights of girls and boys are promoted through curricula and teaching methods (*n *=* *0)
Achievements (*n *=* *85) ways of being and doing which can be realised by different individuals	Improved systems
	CA1 Power holders have improved awareness and responsiveness to the demands, claims, rights and inputs of women (*n *=* *3) CA2 increased representation of women in local and subnational civil and political processes, including during peacebuilding and post conflict restoration (*n *=* *15) CA3 Institutional mechanisms and structures monitor human rights violations and ensure the security, safety and health of women and girls (*n *=* *1) CA4 Increased attention and focus on the needs and priorities of women and girls, and other vulnerable groups during relief and recovery in conflict and post‐conflict settings (*n *=* *0) CA5 Increased representation of women at all levels as decision‐makers in post‐conflict countries (*n *=* *1) CA6 Effective prevention strategies supported to end violence against women and girls (*n *=* *5) CA7 Women have improved and equitable access to justice (*n *=* *3) CA8 Increased community support for women's and children's human, economic and legal rights (*n *=* *11) CA9 Strengthened, accountable, and gender‐responsive law and justice system (*n *=* *0) CA10 CSOs have greater capacity to support and be responsive for women and marginalised groups to take collective action and hold power holders accountable (*n *=* *0) CA11 CSOs are more inclusive to all genders (*n *=* *1)
	Norms and behaviour change
	CB1 Increased awareness in communities of the issues affecting women (*n *=* *3) CB2 Communities have a more positive attitude towards women/marginalised groups (*n *=* *10) CB3 Women have improved attitudes, self‐image and confidence (*n *=* *29) CB4 Improved attitudes and increased support for women's economic, social and human rights by men, household and family members and community members (*n *=* *25) CB5 Decreased violence/discrimination at the household level (*n *=* *9) CB6 Safer and more secure household, communities and areas/territories for women, girls, men, and boys (*n *=* *15) CB7 Reduced frequency and distribution of types of violence by an intimate partner (*n *=* *23) CB8 Improved quality of relationships between women and their household and community members (*n *=* *31)
	Empowerment index
	CC1 Empowerment/Equality Index (*n *=* *27)

##### 5.1.3.5 Study design

Across the entire review, 97% or 75%, of the included studies were RCTs (including cluster RCTs) where random assignment was done at the individual or cluster level. The remaining 25% were QEDs. See Table 15 for an overview (Table 10).

**Table 10:** List of outcomes and number of studies covering them

Category	Study design	Number	% of total
Experimental designs	Randomised controlled trial (RCT)	29	21%
	Cluster randomised controlled trial (Cluster RCT)	72	53%
Quasi experimental designs	Natural experiment: Randomised or as‐if randomised	1	1%
	Natural experiment: Regression discontinuity (RD)	6	4%
	CBA (non‐randomised assignment with treatment and contemporaneous comparison group, baseline and end line data collection)—individual repeated measurement	13	10%
	CBA pseudo panel (repeated measurement for groups but different individuals)	1	1%
	Interrupted time series (with or without contemporaneous control group)	0	0%
	Panel data, but no baseline (pre‐test)	0	0%
	Comparison group with end line data only	14	10%

##### 5.1.3.6 Characteristics of process evaluations and qualitative studies

Out of the 90 included qualitative studies, cash transfers (*n* = 25) were the most common intervention category. This was followed by life skills (*n* = 14), self‐help groups and VSLAs (*n* = 12), asset transfers and sensitisation campaigns (both *n* = 10). The remainder of the intervention categories are as follows: inclusive community‐driven development (*n* = 6), TVET (*n* = 5), safe spaces (*n* = 4), discussion groups (*n* = 2), All‐women police stations (AWPSs) (*n* = 1) and community dialogues and reconciliation (*n* = 1). Finally, no qualitative studies and process evaluations were found for community‐based services, institutional provision of loans and savings and quotas. Most of the qualitative evidence base comprises of descriptive quantitative studies (*n* = 47) while process evaluations account for the smallest study type category (*n* = 14). Only 29 qualitative research studies were included for the qualitative synthesis.

**Figure 15:**
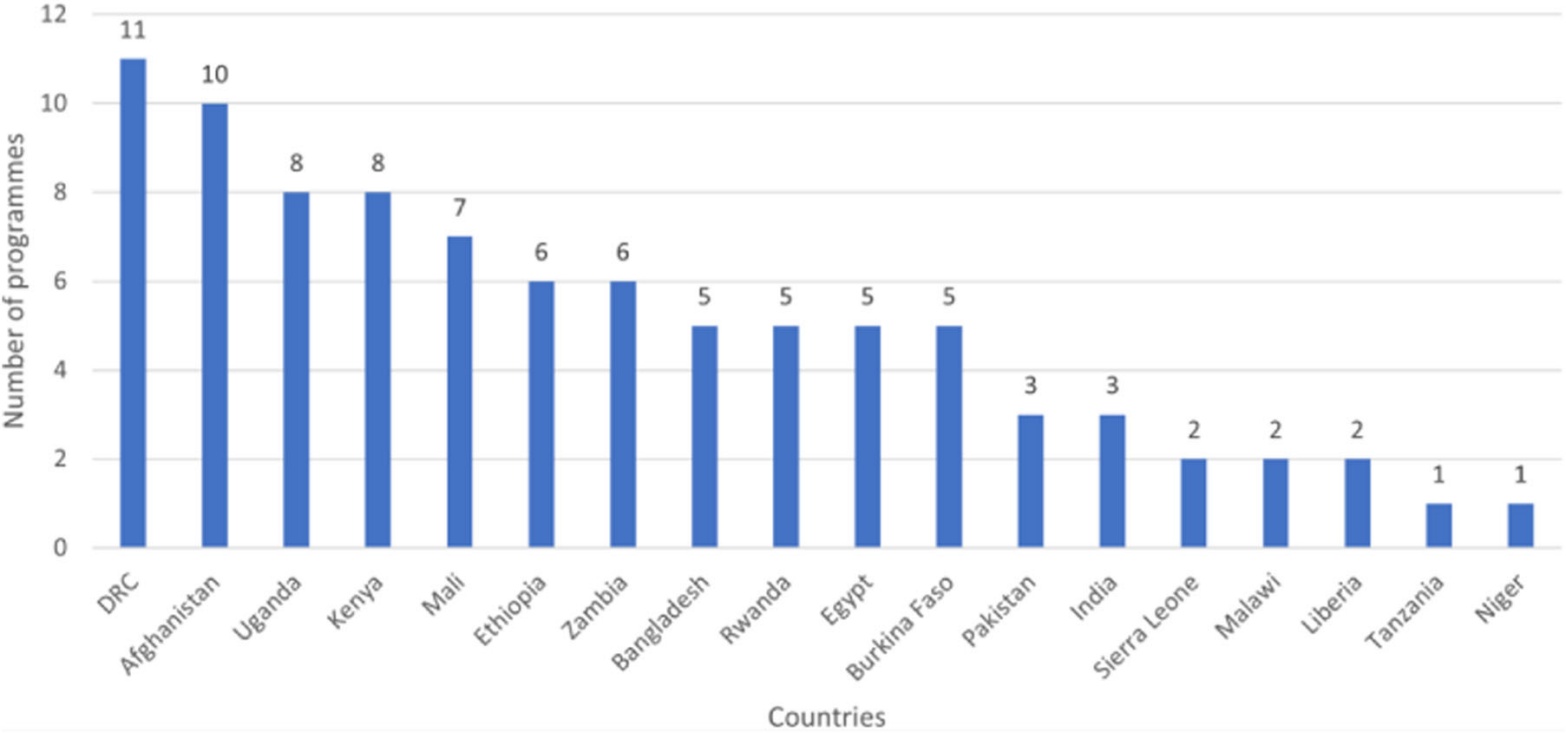
Distribution of qualitative studies, by country. DRC, Democratic Republic of Congo

Figure 15 shows the distribution of these qualitative studies by country. The qualitative studies covered 18 different countries. The qualitative evidence base was dominated by studies in Sub‐Saharan Africa (77%) and the remainder are collectively from Afghanistan, Bangladesh, Pakistan and India. As the studies in the quantitative review component, qualitative studies were heavily concentrated in a small number of countries, including DRC and Uganda. DRC accounts for the greatest number of qualitative studies (*n* = 11), followed by Afghanistan (*n* = 10). Uganda and Kenya each have eight qualitative studies focused on programmes from these two countries. The least number of qualitative studies found were based on programmes in Niger and Tanzania both account for only one study each.

As indicated above, we explored the data reported in the 90 studies included in the qualitative review component according to four analytical lenses in the thematic synthesis: intervention design, intervention implementation, population characteristics, and contextual variables; and how they interact with programme effects. In this process, we generated total of 149 descriptive themes across all studies and 37 analytical themes. These analytical themes present the qualitative synthesis results and are outlined per intervention category below.

#### 5.1.4 Risk of bias and critical appraisal of included studies

##### 5.1.4.1 Risk of bias

The risk of bias tool aimed to assess the risk of bias in each included study based on an assessment of key features of study design and analysis. We undertook a RoB assessment using the Cochrane Collaboration Standards for QED and RCT (Cochrane Collaboration, n.d.). The Figures 17 and 18 provide a summary of the assessment of risk of bias in the included studies, with a breakdown for each domain for QED and for RCT. Supporting Information Appendix SC gives a full summary of our risk of bias assessment at the study level. As we can see, the quality of the included IEs varied.

**Figure 16:**
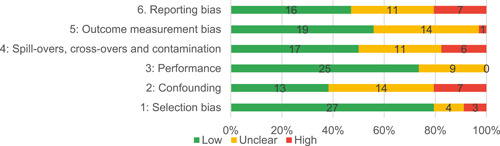
Risk of bias assessment of quasi‐experimental design included studies

Among the 34 QED studies, only 6% (*n* = 2) have an overall low risk of bias, while 41% (*n* = 14) have a high risk of bias and 53% (*n* = 18) raise concerns. By RoB domain, confounding and reporting bias are the categories with the most studies rating high risk of bias or some concerns. For confounding, only about a third of the studies were judged to be low risk (*n* = 13), which means that most studies were unclear or raised at least some concerns about results being confounded by some unobservable characteristic that was not or could not be accounted for during the analysis. For reporting bias, 45% of included QED studies (*n* = 16) have a low risk, while the rest are either not clear or present concerns related to authors not using a credible analysis method, some evidence that the outcomes were selectively reported or lack of sensitivity analysis or robustness checks to add credibility to the results. Also, for spill‐overs, crossovers and contamination, just above half of the included studies (*n* = 17) do not present implementation issues, comparisons are unlikely to receive the intervention, no external program can systematically affect treatment and control groups differently, and there are no survey effects, the rest of studies were unclear or raised some concerns related to any or all of these issues. The other three RoB domains (selection bias, performance and outcome measurement) raised less concerns that will be explored in more detail in each of the intervention sections (Figure 16).

**Figure 17:**
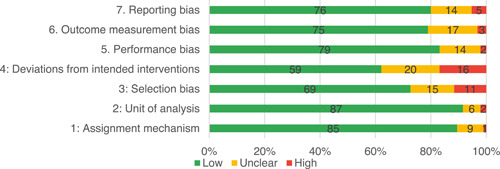
Risk of bias assessment of randomised controlled trial included studies

**Figure 18:**
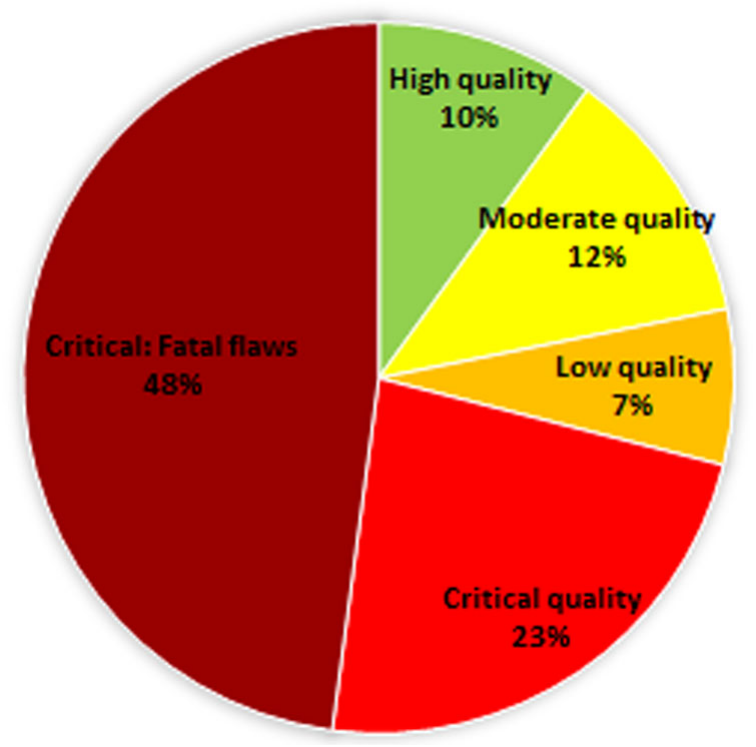
Summary of overall critical appraisal ratings across studies included in the qualitative synthesis

As expected, RCT studies raised less concerns regarding risks of bias. Among the 95 included studies using a RCT design, 26%(*n* = 25) of the studies received an overall rating at low risk of bias and 28%(*n* = 27) at high risk, reasons for concern vary across studies. If we look at the specific domains, only two have a relatively large number of studies scoring high risk or being unclear. The first is deviations from intended interventions because of implementation issues or spill‐overs to comparisons (*n* = 16 studies scored high risk and *n* = 20 were unclear). We noticed in this domain that many papers, despite being prospective evaluations, do not discuss or consider potential contamination issues from other programs. The second domain with the largest number of high‐risk studies was selection bias related to issues with attrition or purposeful sampling (*n* = 11 high risk and *n* = 15 unclear). For all other domains (assignment mechanism, unit of analysis, performance, outcome measurement and reporting bias) at least 80% of the studies scored low risk of bias (Figures 17 and 18).

##### 5.1.4.2 Critical appraisal of process evaluations and qualitative studies

All studies included in the qualitative review component were critically appraised for the trustworthiness of their contribution to the thematic synthesis. We applied a predefined mixed‐methods appraisal tool developed by Langer et al. (2017) and applied in Snilstveit et al. (2019) (see Supporting Information Appendix SA). The appraisal tool made judgments on the adequacy of reporting, data collection, presentation, analysis and conclusions drawn. The appraisal tool assessed the quality of the included qualitative studies, descriptive quantitative studies and process evaluations.

**Figure 19:**
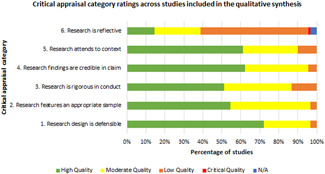
Critical appraisal category ratings across studies included in the qualitative synthesis^5^

We rated studies on a scale from high quality, to moderate, low and critical trustworthiness. Figure 18 provides the results of the critical appraisal on aggregate while Figure 19 presents the breakdown of appraisal ratings per appraisal category. Due to the mixed availability of qualitative documentation from the included studies, a few intervention groups did not have qualitative synthesis including community‐based services; loans and savings; discussion groups; AWPSs; community dialogues and reconciliations; and quotas.

Of 311 critically appraised studies, 71% were rated as critical while the remaining 29% were either of high (10%), moderate (12%) and low quality (7%). This translates into a total of 221 studies being excluded from the synthesis due to critical trustworthiness. Of these, 149 were rated critical based on the presented fatal flaws (Supporting Information Appendix SA) which refers to a significant lack of reported information within identified studies (e.g., no research question or objective reported; no evidence of empirical data being provided). These qualitative studies subject to fatal flaws constituted 48% of the critically appraised studies and provided a main driver of the high number of studies excluded from the synthesis. The second main reason for critical appraisal ratings referred to the appraisal domain of reflectiveness and lack of triangulation (15%); with the reminder of the critical appraisal rating being distributed equally across the remaining domains.

We included a total of 90 studies in our qualitative evidence base following the removal of critically rated studies. Out of these 90 studies, we ranked 31 as high empirical quality, 37 as moderate empirical quality and 22 as low empirical quality. This implies that just over a third of the included studies (34%) were of high trustworthiness while the remaining studies were of either moderate or low trustworthiness (66%). Overall, however, the qualitative evidence base was of poor quality as indicated by the large number of studies includes as of a critical appraisal rating.

For the 90 included studies, Figure 19 provides a breakdown of the critical appraisal ratings by domain. It indicates many high‐quality ratings on the defensibility of the research design (72% of studies), the credibility in research claim (62% of studies) and the relevance for context (61% of studies). In contrast, most of the included studies rated low on trustworthiness on the depth of reflection and triangulation (57% of studies).

### 5.2 Effects of interventions

**Figure 20:**
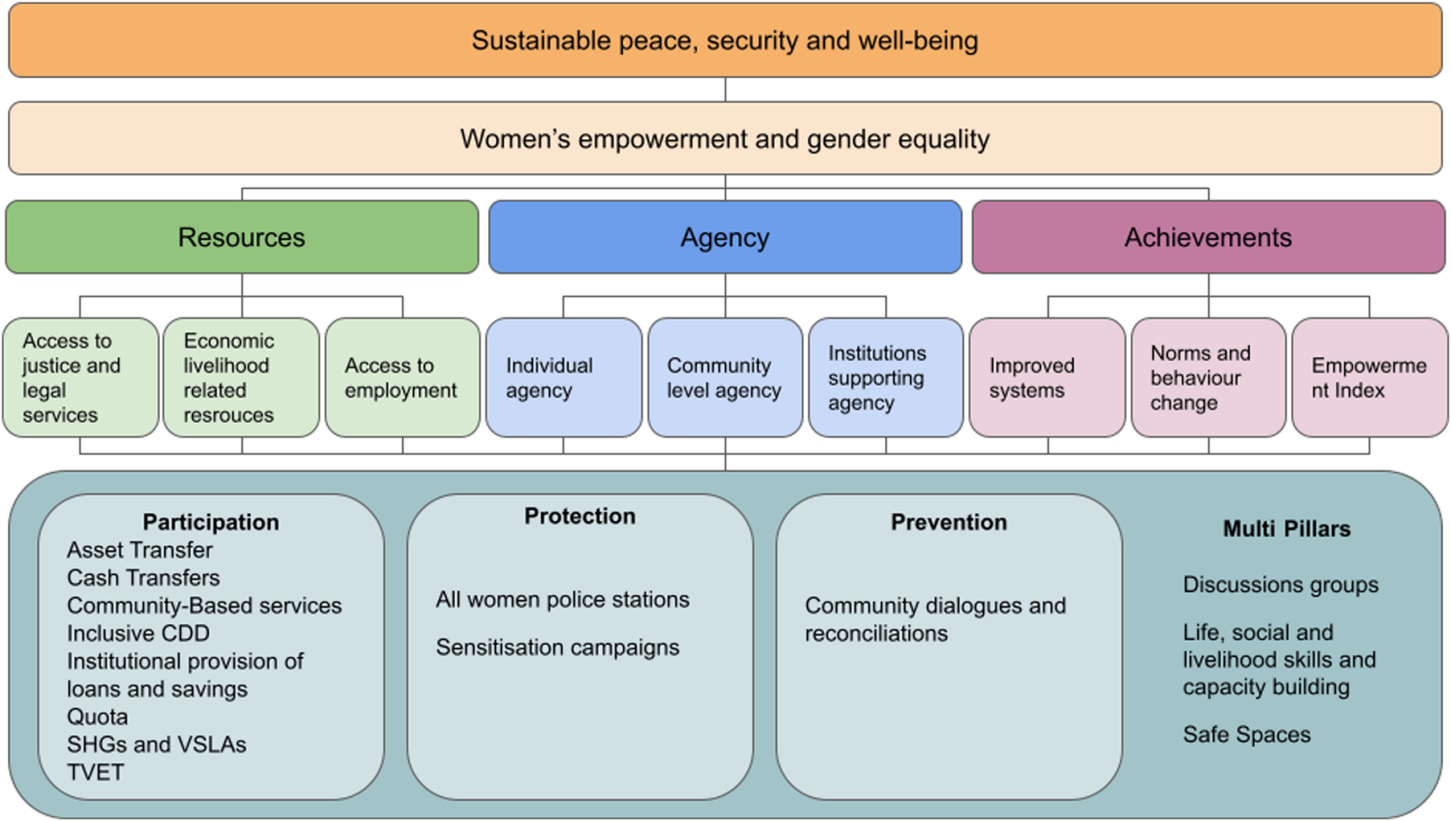
Theory of change of included studies

In this chapter we report the results of the quantitative and qualitative analysis of the effects of our interventions in the included studies of this review. Our analysis is structured around the four pillars of UNSCR 1325 and includes interventions working across multiple pillars. None of the included studies' interventions primarily targeted the Recovery and Relief Pillar. Based on the framework developed in our protocol we identified 14 types of interventions contributing to the achievement of nine immediate outcomes, three secondary outcomes (Resources, Agency and Achievements) and one long term outcome: gender equality and women's empowerment for peaceful and inclusive societies. Figure 20 provides an overview of the intended effects of the interventions of our included studies covering 290,484 beneficiaries (Figure 20).

The following sections will present the effects of the 14 intervention types at the outcome level and grouped into the UNSCR Pillars. Supporting Information Appendix SO provides details on the characteristics of the studies and programmes included in our review.

### 5.3 Effects of interventions under the UNSCR participation pillar

The participation pillar encompasses a set of interventions that create opportunities for, build acceptance of or strengthen capacities for the equal participation and full involvement of women and girls in political, economic and social institutions and decision‐making processes.

In our SR, this pillar gathered evidence from the following types of interventions covering 231,084 beneficiaries:

- Asset transfers
- Cash transfers
- Community‐based services
- Inclusive community‐driven development (CDD)
- Institutional provision of loans and savings
- Quotas
- Self‐help groups (SHGs) and Village Savings and Loan Associations (VSLAs)
- Technical and vocational education and training (TVET)

**Figure 21:**
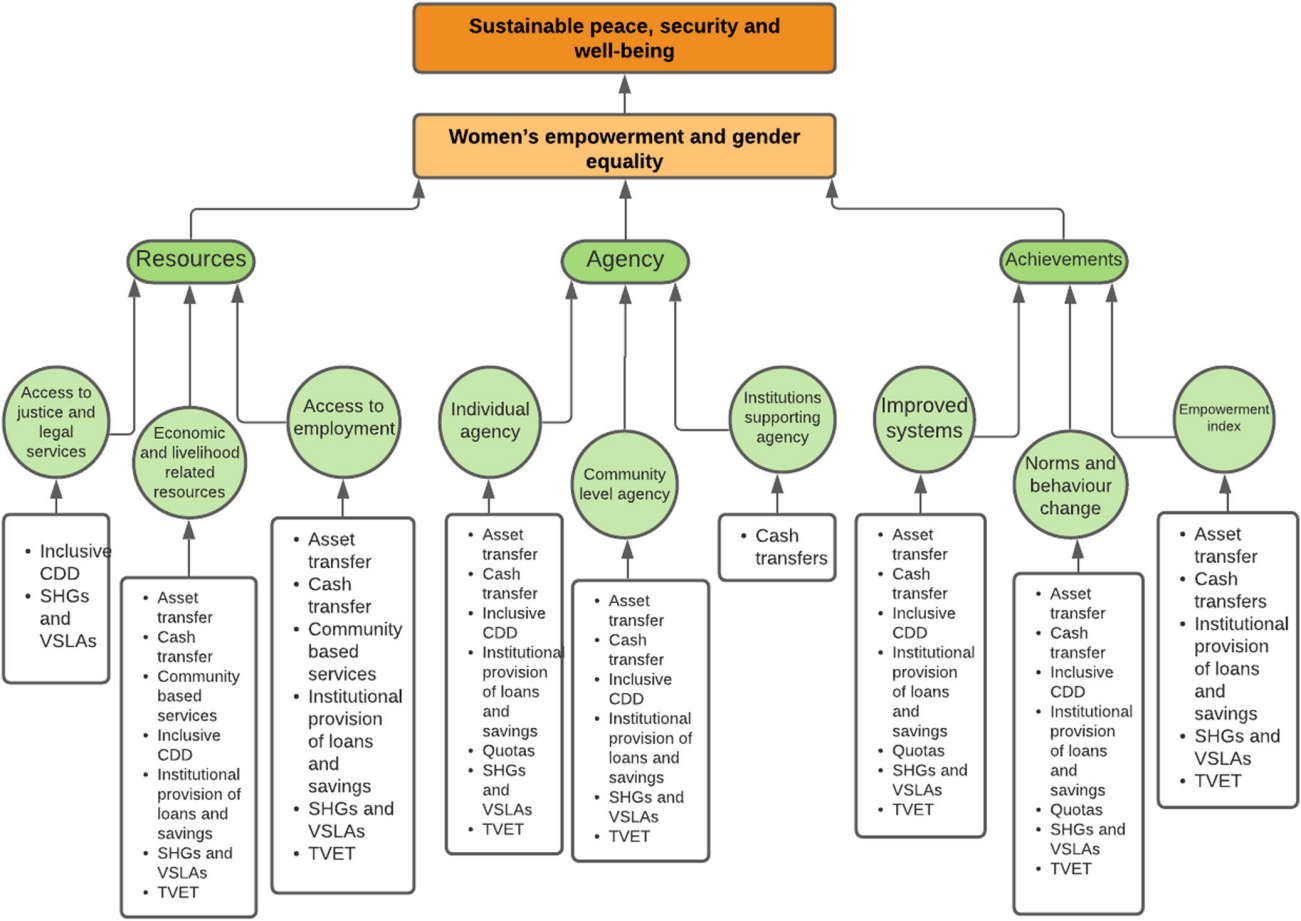
Theory of change of included studies under the participation pillar

This section provides the findings of our synthesis of the 75 included studies evaluating the effect of these interventions on gender equality, women's empowerment and peaceful and inclusive societies. The section is organised by each intervention group. Each sub‐section begins with a description of each intervention group, their activities and ToC, followed by descriptive results and the findings addressing our research questions. Figure 21 provides a summary of the outcomes and targeted effects of the interventions included under the participation pillar:

#### 5.3.1 Asset transfer

We define asset transfers interventions as the transfer of publicly owned land, buildings or goods (including materials, livestock, etc.) to community organisations, individuals or groups (Das et al., 2013).

##### 5.3.1.1 How do asset transfers affect gender equality, women's empowerment and Peace outcomes?

**Figure 22:**
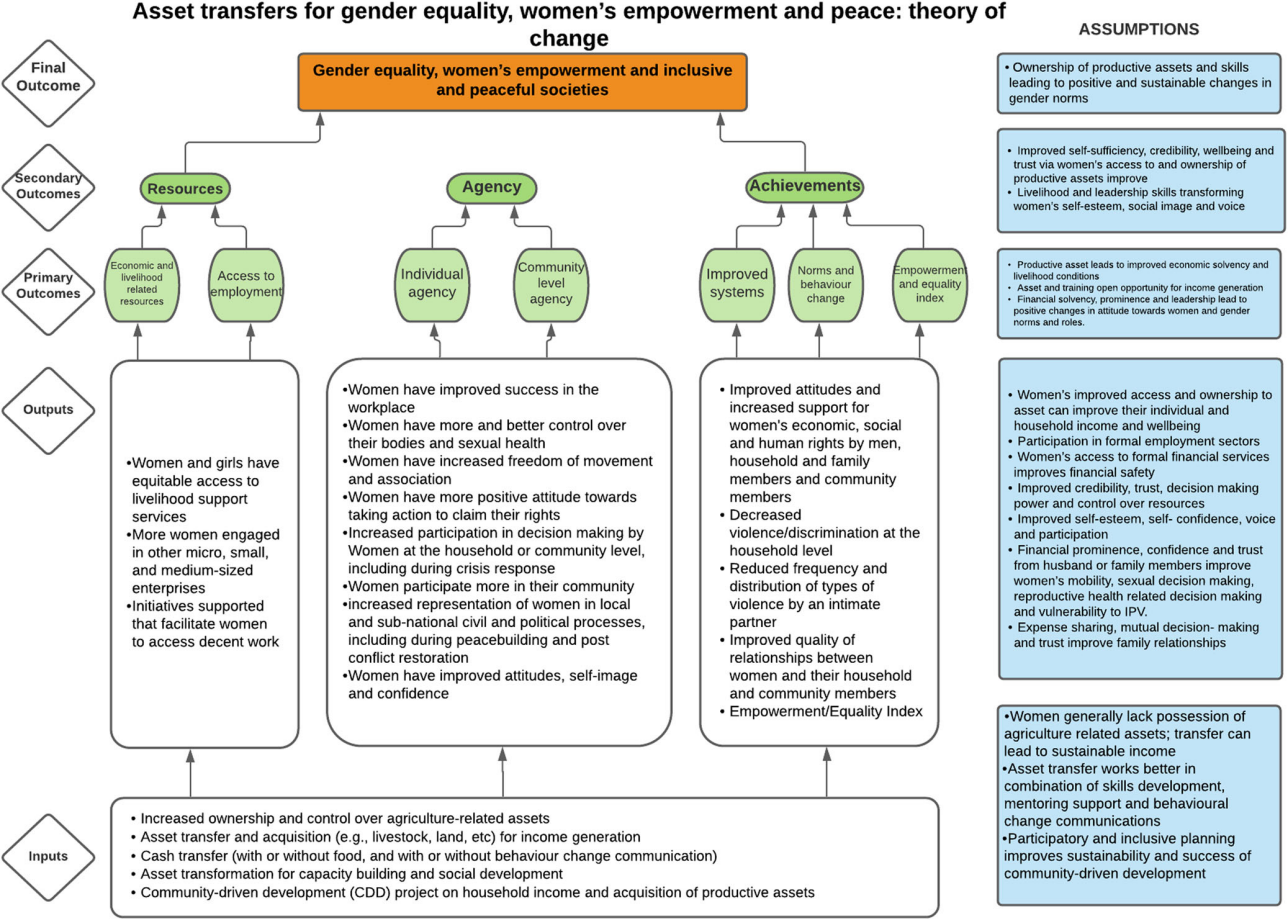
Asset transfers for gender equality, women's empowerment and peace: Theory of change

Figure 22 maps out the causal chain of how asset transfers may improve gender equality, women's empowerment and peace outcomes.

Improving women's and girls' access to productive assets creates multifaceted opportunities in terms of gender equity and women's empowerment. Assets, along with necessary skills development and leadership training, can improve women's access to employment in formal and informal sectors. Financial solvency, on one hand, can ensure personal financial freedom and on the other hand supports families’ wellbeing, children's health and educational expenses (which in many cultures are considered a women's responsibility) along with other household needs. Shared responsibilities, mutual decision making, trust and confidence reduce conflict, violence and improves intra‐family and social relation and cohesion. Financial prominence, control over assets and other relevant qualities can have positive impacts on socio‐cultural norms, gender roles and attitudes unfavourable to women. Mutual respect and understanding can also have positive effects in reducing conflict and SGBV.

##### 5.3.1.2 Description of included studies

We included 10 studies reported in 14 different papers evaluating the effect of eight programmes. We included more than one paper evaluating the same programme if the author(s) reported different outcomes over several papers.

##### 5.3.1.3 Population

Asset transfer programmes in this review primarily targeted women and girls from poor households. However, a few of the programmes also engaged with men. Most of the programmes worked with either individual beneficiaries or households. Only one study (Nkonya et al., 2012) evaluated a programme that entailed community level activities.

The included studies evaluated eight different programmes and trials in South Asia and Sub‐Saharan Africa, totalling five countries: Afghanistan, Bangladesh, Burkina Faso, DRC and Nigeria.

##### 5.3.1.4 Intervention, inputs and activities

The included studies evaluated a range of different asset transfer activities and inputs including:

- Improved land rights for increased ownership and control over agriculture assets for women (*n* = 1): The activities evaluated by the study under this category involved the establishment of village model farms (VMFs) run by women village farm leaders (VFLs). The VMFs served as training sites for women to learn about homestead food production and small animal rearing.
- Asset transfer for income generation and training/support (*n* = 2): Activities in this category included the transfer of productive assets such as livestock, assets for establishing and running small‐scale retail operations, tree nurseries and vegetable growing. Often, assets were offered with a package of complementary training and support.
- Asset transformation for women's social development and skills training (*n* = 1): The included study evaluated a public safety net programme that supported women‐headed poor households (between 18 and 50 years) with a monthly food ration (30 kg of rice) over a period of 24 months. In addition, to integrate food security with development objectives, the programme also provided training on income generating activities such as livestock rearing, vegetable gardening and awareness‐raising on social, legal, and health and nutrition issues.
- Cash transfers for economic empowerment with or without food, and with or without BCC (*n* = 4): The interventions covered by the included studies included cash or food support, with or without intensive nutrition BCC.
- Community‐driven development (CDD) on household income and acquisition of productive assets (*n* = 1): The included study evaluated a CDD project developed by local farmer groups who designed their own local development plans.
- Asset transfer and integration of skills training and learning to improve economic independence and reduce food insecurity (*n* = 1): Activities in this category included food and cash transfers along with training on livelihood skills, awareness on social, legal, health, and nutrition issues, basic literacy and numeracy training and access to credit; public works for community infrastructure development and productive asset creation.
- Livestock asset transfer and economic skills training (*n* = 1): Pigs for Peace (PFP) was a livestock transfer program wherein two piglets aged 2–4 months were provided to participating households under the condition that the household creates a pigpen and a compost pit. The programme also provided practical training for nutrition and care of the livestock asset and support by trained staff.
- Productive asset (e.g., livestock) and cash transfer (*n* = 2): The programmes evaluated consisted of the transfer of a productive asset in the form of livestock, a monthly cash transfer/stipend, basic training on livestock rearing and entrepreneurship and a ‘health subsidy’ which included the provision of a basic hygiene kit and reimbursement for medical expenses or latrine improvements. In one case, the transfer component was accompanied by mentoring visits.
- Provision of land and agricultural training for economic empowerment (*n* = 1): The included activity was an integrated agriculture and nutrition programme that provided land, input and training to women to promote consumption of nutrient rich foods and generate additional income.

Table 16 describes the activities associated with each included study (Table 11).

**Table 11:** Asset transfer activities: Features of included studies

Study	Activity/input	Length of treatment	Intervention frequency
Ahmed et al. (2009)	Asset transfer and integration of skills training and learning to improve economic independence and reduce food insecurity	66 months	Monthly
Bandiera et al. (2013)	Asset transfer for income generation and training/support	Not reported	Not reported
Bandiera et al. (2017)	Asset transfer for income generation and training/support	144 months	Monthly
Bedoya et al. (2019)	Productive asset (e.g., livestock) and a cash transfer	12 months	Weekly
Bedoya et al. (2020)	Productive asset (e.g., livestock) and a cash transfer	36 months	Annually
Das et al. (2013)	Cash transfer for economic empowerment with or without food, and with or without behaviour change communication	Not reported	Not reported
Glass et al. (2017)	Livestock asset transfer and economic skills training	7 months	Weekly
Nkonya et al. (2012)	Community‐driven development project on household income and acquisition of productive assets	24 months	Not reported
Olney et al. (2016)	Provision of land and agricultural training for economic empowerment	24 months	Not reported
Pradhan and Sulaiman (2017)	Asset transformation for women's social development and skills training	24 months	Monthly
Roy et al. (2015)	Cash transfer for economic empowerment with or without food, and with or without behaviour change communication	Not reported	Not reported
Roy et al. (2013)	Cash transfer for economic empowerment with or without food, and with or without behaviour change communication	Not reported	Not reported
Roy et al. (2019)	Cash transfer for economic empowerment with or without food, and with or without behaviour change communication	24 months	Monthly
van den Bold et al. (2015)	Access to land right for increase ownership and control over agriculture‐related asset for women	24 months	Not reported

##### 5.3.1.5 Comparison

All our included studies compared treated groups to comparison groups receiving no intervention. Six studies included multiple treatment arms.

##### 5.3.1.6 Outcomes

The included studies reported on several relevant outcomes, including the following:

- Women have increased access and ownership to assets, credit and income (*n* = 11): Women can apply for, receive and manage assets/credit and income and have support to manage, claim and execute their assets without pressure or influence from external actors, including male family members, husbands and cultural leaders.
- Initiatives that facilitated women to access decent work (formal and informal employment), including people with disabilities (*n* = 7): Women were able to apply for, receive and work in jobs (and have support for the above), without discrimination for sex, gender or other identifying factors, including development of skills for improved access.
- Increased participation in decision making by Women at the household or community level, including during crisis response (*n* = 6): Women take part in all or any step of the decision‐making process at the household community and district level, but also are able to meaningfully take part and have influence on the final decision, including in crisis response.
- Empowerment/Equality Index (*n* = 4): Indices using multiple outcomes of the list to aggregate them into a gender equality and/or women's empowerment score.

Our list of 44 outcomes reported in our included studies is grouped into nine immediate outcomes directly contributing to the three secondary outcomes as per Kabeer's dimensions of women's empowerment and gender equality (Resources, Agency and Achievement). The division into the immediate and secondary outcomes in asset transfer is reported in Table 17 (Table 12).

**Table 12:** Asset transfer summary of secondary and immediate outcomes

Secondary outcome category	Immediate outcome	Number of studies
Resources material, human and social resources which serve to enhance the ability to exercise choice	Access to justice and legal services	0
	Economic and livelihood related resources	13
	Access to employment	6
Agency ability to define one's goals and act upon them and operationalised decision‐making	Individual agency	5
	Community level agency	6
	Institutions supporting agency	0
Achievement ways of being and doing which can be realised by different individuals	Improved systems	1
	Norms and behaviour change	6
	Empowerment index	3

##### 5.3.1.7 Study design

**Figure 23:**
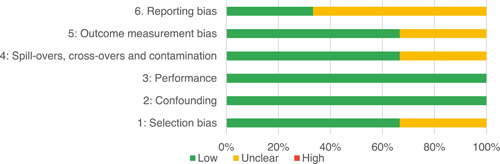
Asset transfer quasi‐experimental design risk of bias assessment

Three of our asset transfer studies used a QED (Ahmed et al., 2009; Nkonya et al., 2012; Pradhan & Sulaiman, 2017). One of the three QEDs in this category was assessed as having a low risk of bias, the other two were assessed as having some concerns. As detailed in Figure 23, high risk of bias was not identified in any RoB domains. All three studies show no limitations regarding confounding (i.e., we did not identify any issues with selection in to the program being done according to clear rules, which are used for the matching even if there are still slight imbalances remaining after matching). Also, no major concern was observed regarding performance bias as we did not identify any issues regarding the monitoring processes. However, the quality assessment of the studies was limited by the lack of discussion by the authors about potential issues with the study design so that we could not discard limitations related to selection bias, data collection and treatment implementation processes were also not discussed in detail so issues related to spill‐overs and outcome measurement could not be discarded. Finally, an unclear description of the methods used in two of the three studies do not allow us to discard any concerns related to potential reporting bias.

**Figure 24:**
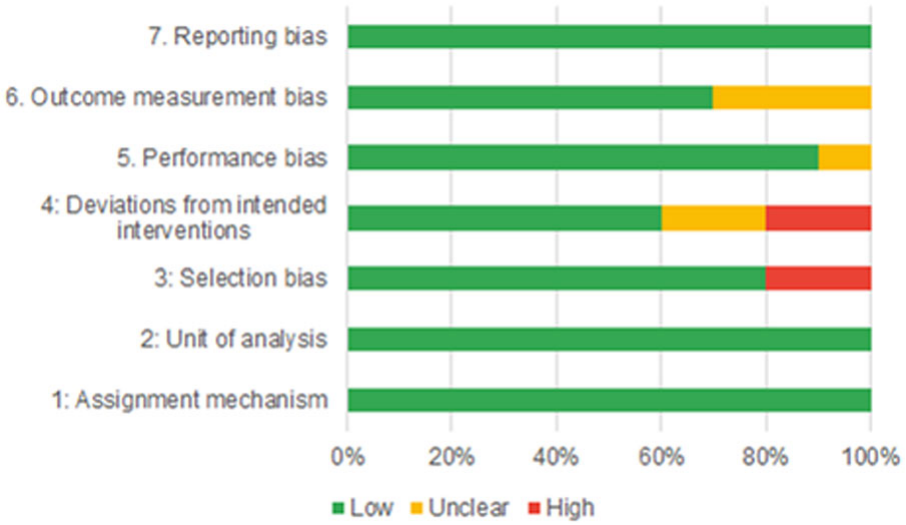
Asset transfer randomised controlled trial risk of bias assessment

Seven of our asset transfer studies used an experimental design (Das et al., 2013; Bandiera et al., 2017, 2013; Bedoya et al., 2019, 2020; Glass et al., 2017; Olney et al., 2016; Roy et al., 2015, 2019; van den Bold et al., 2015). Approximately 20% of the papers scored a low risk of bias, 40% a high risk of bias and the rest showed some concerns. As detailed in Figure 24, high risk of bias was identified in two RoB domains (selection, and deviation from intended interventions) and both represented 20% of the sample of included studies.

Risk of selection bias concerns in the asset transfer interventions using RCT designs came from differences in attrition between treatment and control groups that were not adjusted for in the analysis (Olney et al., 2016; van den Bold et al., 2015). Further, risks of deviations from intended interventions were observed because of assignment at the household level within the same villages, where the lack of geographic separation between treatment and control groups poses a risk for contamination (Bedoya et al., 2019; Glass et al., 2017), in other cases spill‐overs could not be discarded because participants in control villages were also showing changes in the outcome variables (van den Bold et al., 2015) or authors d0 not report enough discussion to discard implementation issues (Bandiera et al., 2013). No major concerns were observed for the rest of domains, but again we could not discard limitations related to outcome measurement in three studies (Das et al., 2013; Roy et al., 2015, 2019) and to performance in Glass et al. (2017). We did not identify limitations related to the assignment mechanism, the unit of analysis or selective reporting of outcomes in any of the included studies.

##### 5.3.1.8 Qualitative studies, process evaluations and project documents

We identified ten additional documents related to seven programmes covered by the Asset Transfer group of included studies.

- Pigs for Peace (DRC): one qualitative study
- Enhanced‐homestead food production (E‐HFP) programme (Burkina Faso): two process evaluations and one qualitative study
- Transfer Modality Research Initiative (TMRI) (Bangladesh): one process evaluation
- Targeting the Ultra Poor (TUP) programme (Afghanistan): one qualitative document and two descriptive quantitative documents
- Income‐Generating Vulnerable Group Development (IGVGD)—Bangladesh: one process evaluation
- Targeted Ultra‐Poor (TUP) Programme—Bangladesh: one descriptive quantitative document

The asset transfer qualitative documents were ranked as high‐quality empirical research (*n* = 4), followed by moderate (*n* = 5) and low quality (*n* = 1).

##### 5.3.1.9 Synthesis of findings

The following subsection presents the results of effectiveness of asset transfers on Gender Equality, Women's Empowerment and Peace outcomes.

##### 5.3.1.10 Quantitative findings

*Effects of asset transfers on increased capacity of women to understand and use financial, banking and business services*

Bedoya et al. (2020), an experimental study in Afghanistan, was the only study evaluating the impact of asset transfers on the increased capacity of women to understand and use financial, banking and business services. The study reported a large effect (*g *=* *0.62, 95% CI [0.50, 0.74]). We assessed this study as having a low risk of bias.

*Effects of asset transfers on women's access to and ownership of assets, credit and income*

**Figure 25:**
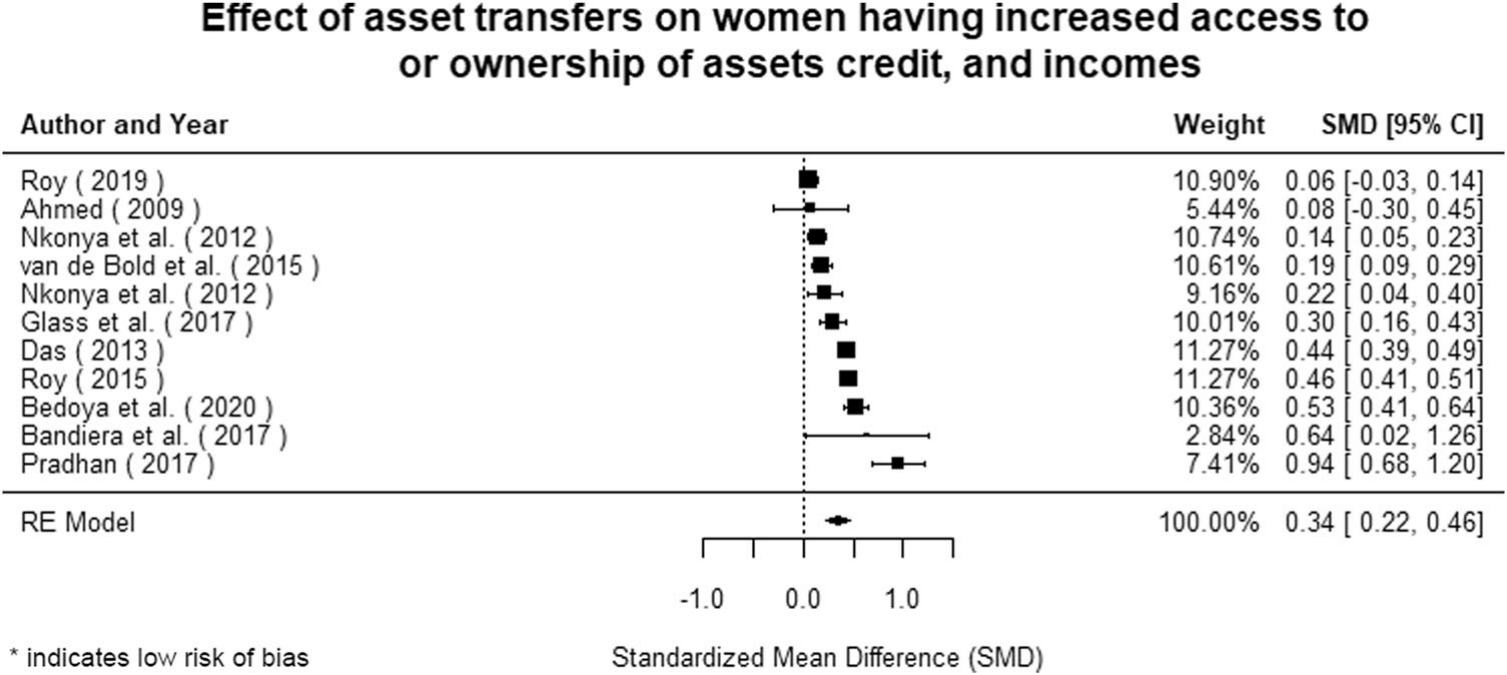
AssetAB2: Forest plot showing the observed outcomes and the estimate of the random‐effects model

We included a total of k=11 studies. We assessed three studies as low risk of bias, six as some concerns and two as high risk of bias. The observed outcomes ranged from 0.06 to 0.94. The estimated average outcome based on the random‐effects model was $\hat{\mu }=0.34$ (95% CI: 0.22to0.46). Therefore, the average outcome differed significantly from zero (z=5.55, p<0.001). A forest plot showing the observed outcomes and the estimate based on the random‐effects model is shown in Figure 25 (AssetAB2).

According to the Q‐test, the true outcomes appear to be heterogeneous (Q(10)=139.89, p<0.01, ${\hat{\tau }}^{2}=0.03$, $I^{2}=92.85$%). An examination of the studentised residuals revealed that one study (Pradhan & Sulaiman, 2017) had a value larger than ±2.84 and may be a potential outlier in the context of this model. According to the Cook's distances, none of the studies could be considered to be overly influential.

**Figure 26:**
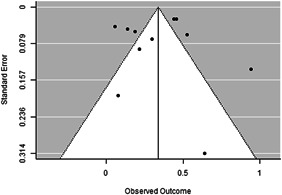
AssetAB2b: Funnel plot

A funnel plot of the estimates is shown in Figure 26 (AssetAB2b). Neither the rank correlation nor the regression test indicated any funnel plot asymmetry (p=0.76 and p=0.36, respectively). We had sufficient data to test most moderators for this intervention and outcome group (ROB score, exposure to intervention in months, evaluation period in months, study design [RCT vs. QED], whether or not the model was adjusted for covariates, gender inequality score, and fragile states index score), but no moderators were significant. We were unable to test the number of items in the outcome measure given that most were single items related to annual income. Finally, for asset transfers we wanted to examine whether there was a difference between interventions with just an asset transfer versus those with an asset transfer plus an additional training component. However, only one programme offered an asset without an accompanying training, so we were unable to test this as a potential moderator of the size of the effect.

*Effects of asset transfers on women's engagement in other micro, small and medium‐sized enterprises*

Bedoya et al.'s (2019) experimental study in Afghanistan was the only study evaluating the impact of asset transfer on more women being engaged in other micro, small and medium‐sized enterprises. There was a medium, and statistically significant, effect (*g *=* *0.24, 95% CI [0.13, 0.36]), however we assessed the study as having high risk of bias.

*Effects of asset transfers on women's access to decent work (formal and informal employment)*

**Figure 27:**
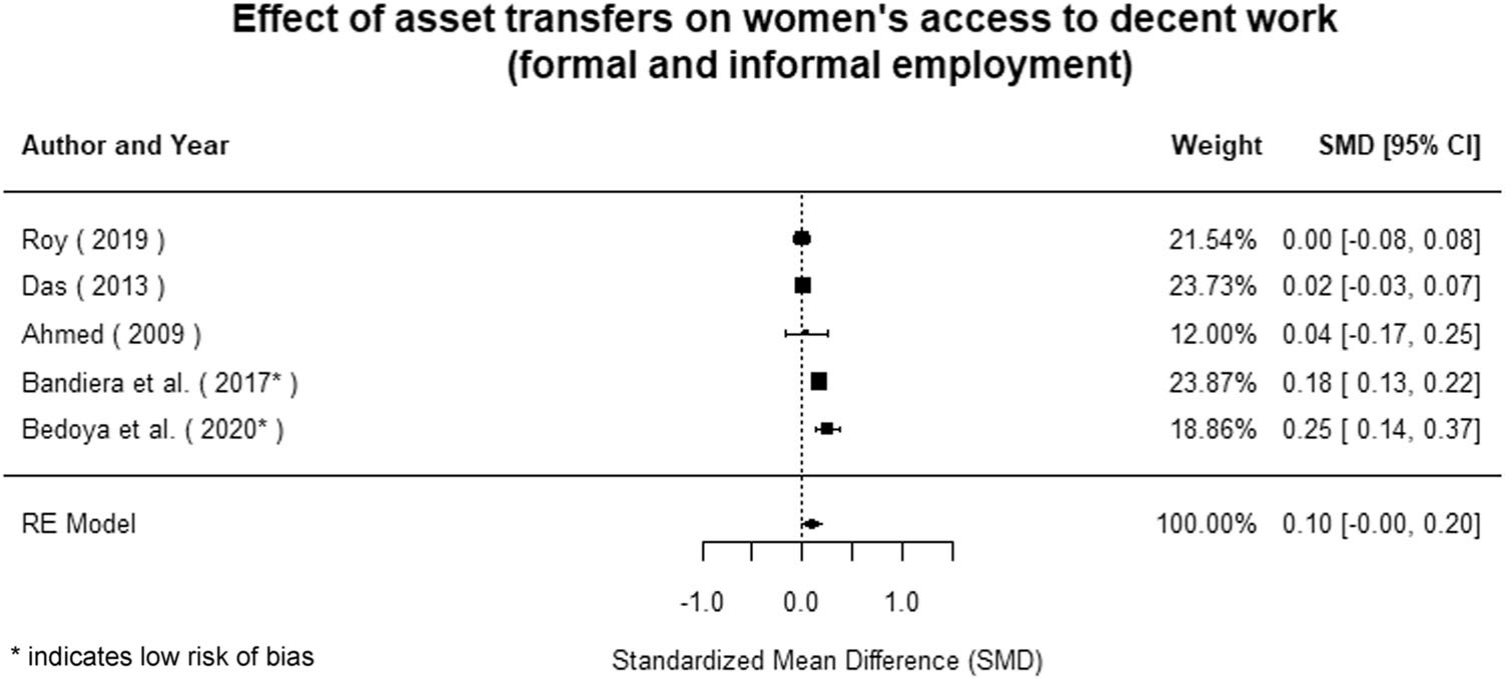
AssetAC2: Forest plot showing the observed outcomes and the estimate of the random‐effects model

We included a total of k=5 studies in the analysis. We assessed two studies as low risk of bias, three as some concerns, and none as high risk of bias. The observed outcomes ranged from 0.00 to 0.25. The estimated average outcome based on the random‐effects model was $\hat{\mu }=0.10$ (95% CI: −0.00 to 0.20). Therefore, the average outcome did not differ significantly from zero (z=1.93, p=0.05). A forest plot showing the observed outcomes and the estimate based on the random‐effects model is shown in Figure 25 (AssetAC2) (Figure 27).

According to the Q‐test, the true outcomes appear to be heterogeneous (Q(4)=33.11, p<0.01, ${\hat{\tau }}^{2}=0.01$, $I^{2}=87.92$%). An examination of the tudentized residuals revealed that none of the studies had a value larger than ±2.58 and hence there was no indication of outliers in the context of this model. According to the Cook's distances, none of the studies could be considered to be overly influential.

We had sufficient data to test most moderators for this intervention and outcome group (ROB score, exposure to intervention in months, evaluation period in months, study design (RCT vs. QED), whether or not the model was adjusted for covariates, gender inequality score, and fragile states index score), but no moderators were significant. We were unable to test the number of items in the outcome measure given that most were single items related to annual income. We were also unable to test whether asset transfers with a training component produced greater effects than asset transfers alone because all of the interventions in this outcome category included the training component.

*Effects of asset transfers on improved capacity of women entrepreneurs*

Bedoya and colleagues' (2020) experimental study in Afghanistan was the only study evaluating the impact of asset transfer on improved capacity of women entrepreneurs. Their report included two effects that fell into this outcome category (primary woman participates in market work and primary woman is owner or manager of an HH non‐agro business). The effects were positive and significant in both cases, the first was a large effect (*g *=* *0.46, 95% CI [0.34, 0.58]) and the second a small one (*g *=* *0.15, 95% CI [0.04, 0.27]). However, we assessed the study as having high risk of bias.

*Effects of asset transfers on women having improved success in the workplace*

Bandiera and colleagues’ (2013) experimental study in Bangladesh was the only study evaluating the impact of asset transfer on women having improved success in the workplace. Their report included two effects that fell into this outcome category (labour productivity measured by hourly earnings—at 24 and 48 months). The point estimates were very small and not statistically different form zero, (*g *=* *−0.03, 95% CI [−0.08, 0.02]) and (*g *=* *0.09, 95% CI [0.04, 0.14]), respectively. We assessed the study as having some risk of bias concerns.

*Effects of asset transfers on women having more and better control over their bodies and sexual health*

Ahmed et al.'s (2009) quasi‐experimental study in Bangladesh was the only study evaluating the impact of asset transfer on women having more and better control over their bodies and sexual health. For each of the three interventions being evaluated (food, cash and both), their report included four effects that fell into this outcome category (e.g., whether woman ever used birth control). The effects were null, ranging from *g *=* *0.00 [95% CI: −0.22, 0.22]) to *g *=* *0.14 [95% CI: −0.10, 0.38]). We assessed the study as having some risk of bias concerns.

*Effects of asset transfers on women's freedom of movement and association*

Ahmed et al.'s (2009) quasi‐experimental study in Bangladesh was the only study evaluating the impact of asset transfer on women having increased freedom of movement and association. For each of the three interventions being evaluated (food, cash and both), their report included five effects that fell into this outcome category (e.g., whether woman decides by herself to go outside the community to visit friends or relatives). The effects ranged from medium, negative effects (*g *=* *–0.24, 95% CI [−0.46, −0.03]) to small, positive but nonsignificant point estimates (*g *=* *0.17, 95% CI [−0.07, 0.41]). Only one of the 15 effect estimates (a negative one) was statistically significant. We assessed the study as having some risk of bias concerns.

*Effects of asset transfers on reduced instances of child or forced marriage*

Bedoya and colleagues' (2020) experimental study in Afghanistan was the only study evaluating the impact of asset transfer on reduced instances of child or forced marriage. There was a small negative but non‐signifiant point estimate (*g *=* *–0.14, 95% CI [−0.31, 0.02]), and we assessed the study as having low risk of bias.

*Effects of asset transfers on participation in decision making by women at the household or community level*

**Figure 28:**
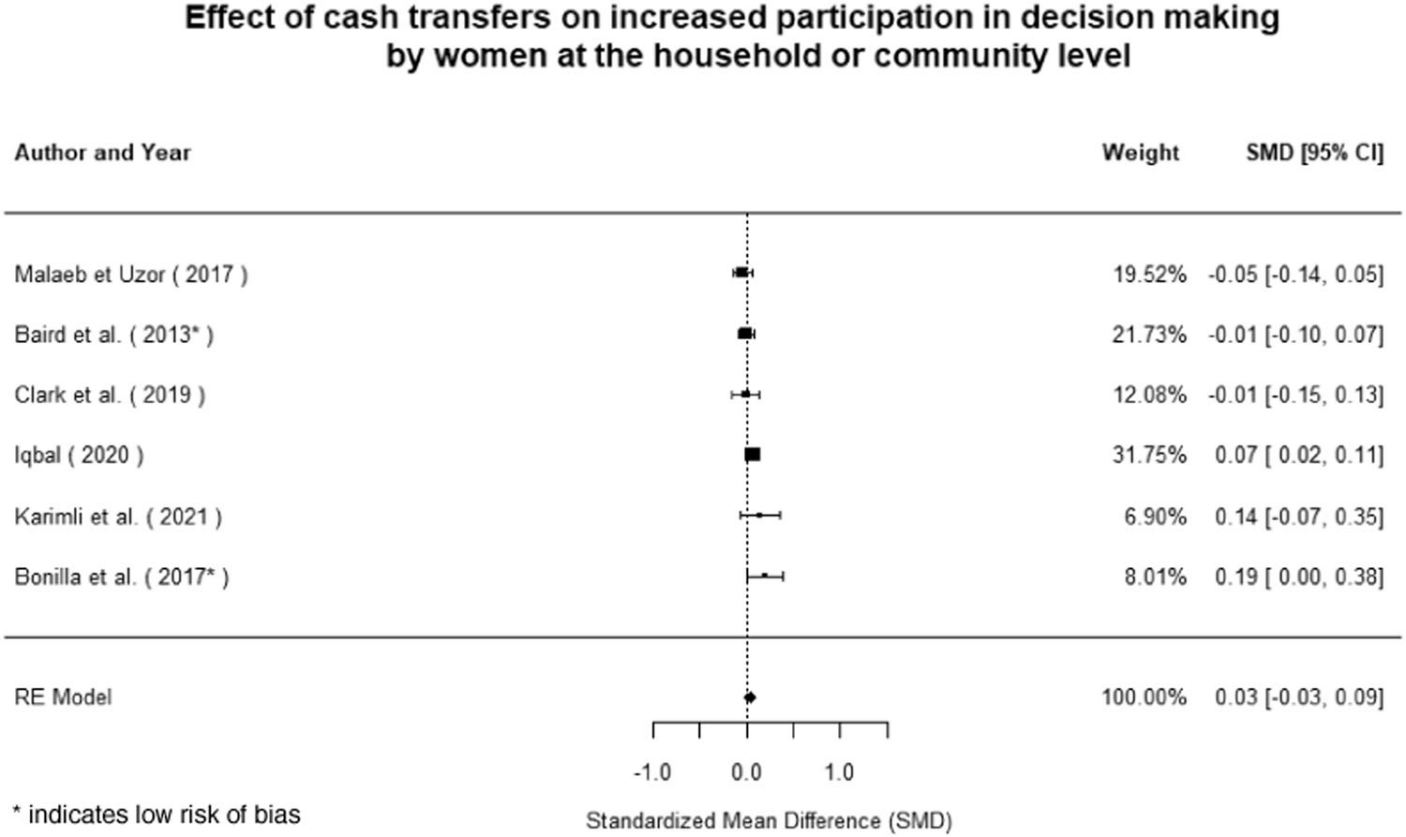
AssetBB1. Forest plot showing the observed outcomes and the estimate of the random‐effects model

We included a total of k=5 studies in the analysis. We assessed one study as low risk of bias, three as some concerns, and one as high risk of bias. The observed outcomes ranged from −0.01 to 0.11. The estimated average outcome based on the random‐effects model was $\hat{\mu }=0.07$ (95% CI: 0.04 to 0.11). Therefore, the average outcome differed significantly from zero (z=4.51, p<0.01). A forest plot showing the observed outcomes and the estimate based on the random‐effects model is shown in Figure 28 (AssetBB1).

According to the Q‐test, there was no significant amount of heterogeneity in the true outcomes (Q(4)=3.06, p=0.55, ${\hat{\tau }}^{2}=0.00$, $I^{2}=0.00$%). An examination of the studentised residuals revealed that none of the studies had a value larger than ±2.58 and hence there was no indication of outliers in the context of this model. According to the Cook's distances, none of the studies could be considered to be overly influential. With no heterogeneity among these effects, moderator analyses were not appropriate.

*Effects of asset transfers on women's participation in their community*

Olney and colleagues' (2016) experimental study in Burkina Faso was the only study evaluating the impact of asset transfer on women participating more in their community. There was a medium, and statistically significant, positive effect (*g *=* *0.27, 95% CI [0.15, 0.39]), however we assessed the study as having high risk of bias.

*Effects of asset transfers on representation of women in local and subnational civil and political processes*

Bedoya and colleagues' (2020) experimental study in Afghanistan was the only study evaluating the impact of asset transfer on increased representation of women in local and subnational civil and political processes, including during peacebuilding and post conflict restoration. Their report included 5 effects that fell into this outcome category (e.g., primary woman belongs to a political party). The point estimates ranged from very small, positive and not statistically significant (*g *=* *0.02, 95% CI [−0.10, 0.14]) to large, positive and significant effects (*g *=* *0.48, 95% CI [0.37, 0.60]). We assessed the study as having low risk of bias.

*Effects of asset transfers on women having improved attitudes, self‐image and confidence*

**Figure 29:**
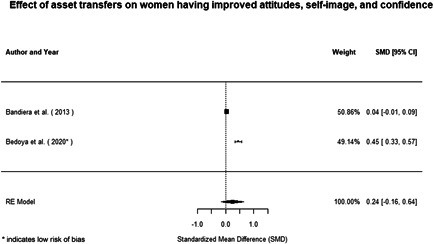
AssetCB3. Forest plot showing the observed outcomes and the estimate of the random‐effects model. CI, confidence interval

Only two studies reported the impact of asset transfers on women having improved attitudes, self‐image, and confidence, thus we included *k *=* *2 studies in the analysis. We assessed one of the studies as low risk of bias and the other as having some concerns of risk of bias. The estimated average outcome based on the random‐effects model was $\hat{\mu }=0.24$ (95% CI: −0.16to0.64). This positive effect did not differ significantly from zero (z=1.17, p=0.24). A forest plot showing the observed outcomes and the estimate based on the random‐effects model is shown in Figure 29 (AssetCB3). Given the small number of studies, this result should be interpreted with caution. According to the Q‐test, there was no significant amount of heterogeneity in the true outcomes (Q(1)=40.83, p<0.001, ${\hat{\tau }}^{2}=0.08$, $I^{2}=97.55$%). With only two studies, moderator analyses were not possible, and tests of publication bias are not valid.

*Effects of asset transfers on attitudes and support for women's economic, social and human rights by men, household and family members and community members*

Bedoya's (2019) experimental study in Afghanistan was the only study evaluating the impact of asset transfer on improved attitudes and increased support for women's economic, social and human rights by men, household and family members and community members. There was a very small, but not statistically significant, point estimate (*g *=* *−0.01, 95% CI [−0.14, 0.12]), and we assessed the study as having high risk of bias.

*Effects of asset transfers on reduced occurrence of intimate partner violence*

**Figure 30:**
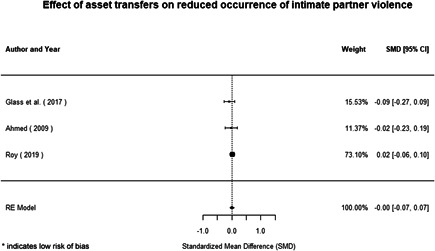
AssetCB7: Forest plot showing the observed outcomes and the estimate of the random‐effects model. CI, confidence interval

We included a total of k=3 studies in the analysis. We assessed none of the studies as low risk of bias, two as some concerns, and one as high risk of bias. The observed outcomes ranged from −0.09 to 0.02. The estimated average outcome based on the random‐effects model was $\hat{\mu }=-0.001$ (95% CI: −0.07 to 0.07). Therefore, the average outcome did not differ significantly from zero (z=−0.04, p=0.97). A forest plot showing the observed outcomes and the estimate based on the random‐effects model is shown in Figure 30 (AssetCB7).

According to the Q‐test, there was no significant amount of heterogeneity in the true outcomes (Q(2)=1.16, p=0.56, ${\hat{\tau }}^{2}=0.00$, $I^{2}=0.00$%). An examination of the studentised residuals revealed that none of the studies had a value larger than ±2.39 and hence there was no indication of outliers in the context of this model. According to the Cook's distances, none of the studies could be considered to be overly influential. With only three studies and no heterogeneity, moderator analyses were not appropriate.

*Effects of asset transfers on improved quality of relationships between women and their household and community members*

**Figure 31:**
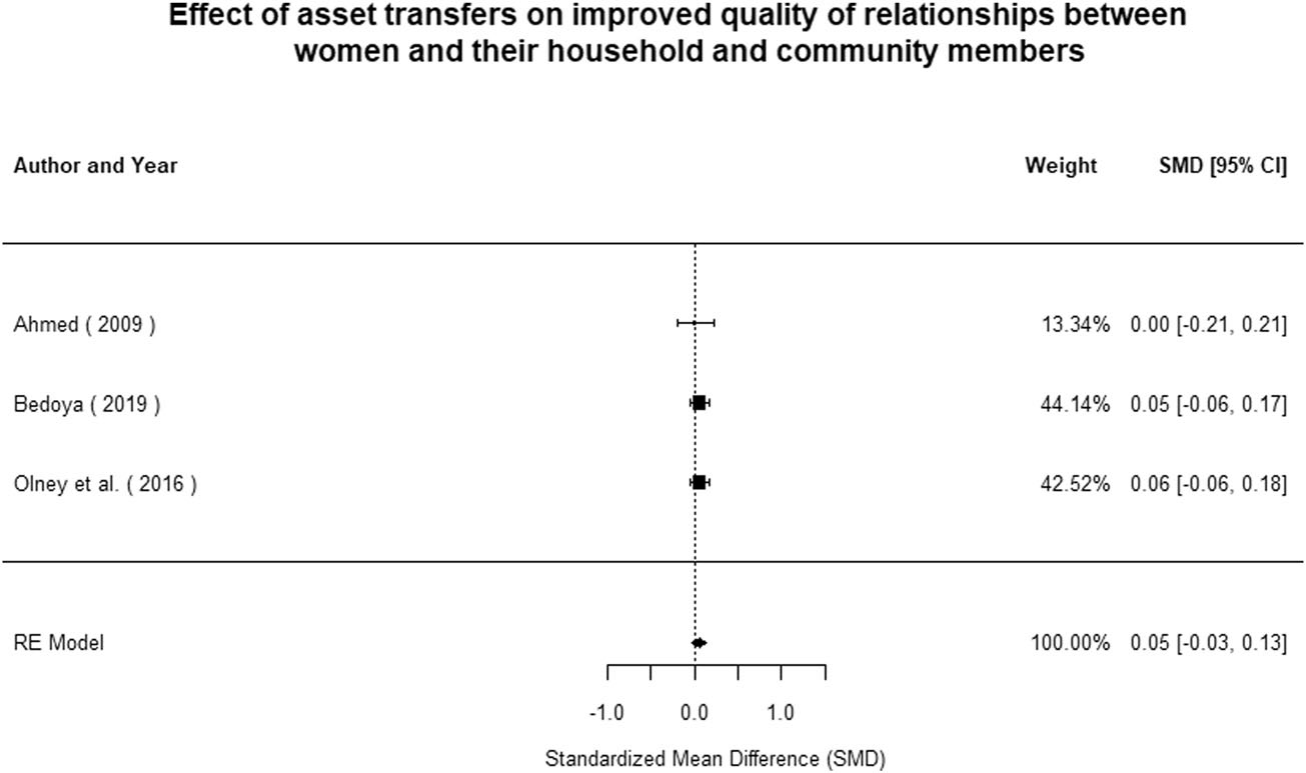
AssetCB8: Forest plot showing the observed outcomes and the estimate of the random‐effects model. CI, confidence interval

We included a total of k=3 studies in the analysis. We assessed none of the studies as low risk of bias, one as some concerns, and two as high risk of bias. The observed outcomes ranged from 0.003 to 0.06. The estimated average outcome based on the random‐effects model was $\hat{\mu }=0.05$ (95% CI: −0.03 to 0.13). Therefore, the average outcome did not differ significantly from zero (z=1.26, p=0.21). A forest plot showing the observed outcomes and the estimate based on the random‐effects model is shown in Figure 31 (AssetCB8).

According to the Q‐test, there was no significant amount of heterogeneity in the true outcomes (Q(2)=0.21, p=0.90, ${\hat{\tau }}^{2}=0.00$, $I^{2}=0.00$%). An examination of the studentised residuals revealed that none of the studies had a value larger than ±2.39 and hence there was no indication of outliers in the context of this model. According to the Cook's distances, none of the studies could be considered to be overly influential. With only three studies and no heterogeneity, moderator analyses were not appropriate.

*Effects of asset transfers on women's empowerment index*

**Figure 32:**
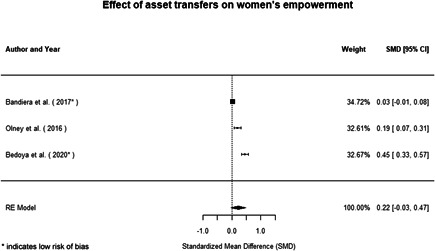
AssetCC1: Forest plot showing the observed outcomes and the estimate of the random‐effects model. CI, confidence interval

We included a total of k=3 studies in the analysis. We assessed two studies as low risk of bias, none as some concerns, and one as high risk of bias. The observed outcomes ranged from 0.034 to 0.449. The estimated average outcome based on the random‐effects model was $\hat{\mu }=0.22$ (95% CI: −0.03 to 0.47). Therefore, the average outcome did not differ significantly from zero (z=1.73, p=0.08). A forest plot showing the observed outcomes and the estimate based on the random‐effects model is shown in Figure 32 (AssetCC1).

According to the Q‐test, the true outcomes appear to be heterogeneous (Q(2)=44.13, p<0.001, ${\hat{\tau }}^{2}=0.05$, $I^{2}=95.47$%). An examination of the andomized residuals revealed that one study (Bedoya et al., 2020) had a value larger than ±2.39 and may be a potential outlier in the context of this model. Indeed, sensitivity analyses leaving out each study indicated that removing Bedoya (2010) would reduce the overall average effect $(\hat{\mu }=0.19\;\left[95\%\;\text{CI}:-0.05\;\text{to}\;0.26\right]$. The effect would still be positive, but no longer significant (*z *=* *1.31, *p *=* *0.19). According to the Cook's distances, none of the studies could be considered to be overly influential. With only three studies, moderator analyses were not appropriate.

##### 5.3.1.11 Qualitative findings

We conducted a thematic synthesis on the ten linked qualitative studies to the included asset transfer interventions. As indicated above, this thematic synthesis aims to identify themes related to the interplay of intervention design, intervention implementation, target population, and contextual variables with intervention outcomes and effects. In total, we identified 15 descriptive themes, which we configured into four analytical themes (Supporting Information Appendix SN). These four analytical themes present the synthesis results and are discussed in more detail below.

*Theme 1: Water access issues, extreme weather conditions, lack of adequate inputs, animal and pest attacks negatively affect crop and livestock yields*.

Asset transfer programmes face challenges that could affect the ability of women to use land and to maximise yields (Olney et al., 2015; Van den Bold, 2013). In Burkina Faso, findings from Van den Bold (2013) showed that even though there was an indication of greater ability for women to use land for agricultural purposes in the intervention villages, primary obstacles included rudimental rainfall patterns, inadequate agricultural inputs and general lack of land to cultivate. In the same country, Olney and colleagues (2015) findings showed whilst there were improvements in production, a significant number of the beneficiaries (83%) had faced challenges with crops they were growing. Specifically, 64% of the beneficiaries faced problems with insufficient water and whilst 31% did not have adequate materials for irrigation. Animal (goats, lizards) and pest (insects) attacks on gardens was an issue for 29% of beneficiaries. 27% of the beneficiaries indicated having insufficient or absence of fences to protect their gardens from pests. In the TUP programme in Bangladesh, beneficiaries reported insufficient nutritional support for animals that they had reared. This had implications on creating more work for beneficiaries to ensure that grass was available for these animals to consume (Karimli et al., 2019; Sarkar & Karim, 2018).

There is evidence that livestock death and poor‐quality seeds received may have affected the implementation of programme activities. Some beneficiaries of the HKI E‐HFP programme in Burkina Faso mentioned that the chickens provided by the programme died soon after receipt and two people reported having problems with the seeds they received (Olney et al., 2015).

The TUP programme in Bangladeshreported negative spill over effects of the programme caused by animal destruction of nonmember crops. Frequent destruction of neighbours’ crops by some beneficiaries’ livestock caused conflicts within the community. The issues were resolved by committees that andomize and brokered compensation by beneficiaries (Sarkar & Karim, 2018), potentially andomized programme gains from asset transfers in the process.

*Theme 2: Traditional practices related to gender roles and women's rights to land, absence of spousal, family and/or community support can hinder success of assets transfer initiatives*.

Qualitative findings showed that asset transfers enhanced women's ability to own land (Van den Bold, 2013), promoted women's household decision making (Ahmed et al., 2014), and supported marital and family relationships (Ahmed et al., 2014; Sarkar & Karim, 2018). That said, such findings are prone to the mitigating effects of traditional practices (which stifle support) on gender roles and women's right to land. For instance, the HKI E‐HFP programme in Burkina Faso observed changes in opinions regarding women's landownerships in villages and their potential to own and use the land. However, men continued to have majority control over and ownership of land and assets resulting from traditional practices of land ownership A community level nutrition trainer from the HC pointed out ‘the weight of tradition’ as a key barrier to the adoption of new community level practices (Van den Bold, 2013).

In Bangladesh, women reported to continue to have little say in decision making and limited mobility within the community. Focus group and case study evidence from Ahmed et al. (2014) showed evidence of strict adherence to stringent gender roles and norms with minimum appreciation for the work that women carry out in the household. In some cases, there were no reported changes to gender divisions of labour within the household despite women taking on more work outside the household (Ahmed et al., 2014). In fact, access to new resources could trigger insecurities among other family members who try to maintain control over beneficiary women (Ahmed et al., 2014). Qualitative findings from Olney and colleagues (2015) study on Burkina Faso's HKI E‐HFP indicated lack of trust in women's activities by their husbands and other household members. Women also reported that husbands, other members of the household or community members viewed asset transfer related activities as diverting from traditional values. These factors acted as barriers to implementing learnt practices.

Lack of community support emerges as a barrier in asset transfers that brought conflicts between beneficiaries and non‐beneficiaries in Bangladesh's TUP programme. For example, one respondent reported non‐beneficiary refusal to allow beneficiaries to use paths that cross their lands as they felt excluded. This together with suspicions of beneficiary conversion to a different religion led to resentment and andomizedize of some beneficiaries (Sarkar & Karim, 2018). Other participants reported incidences of verbal abuse by other villagers for participating in these programmes as they considered it inappropriate for women to engage in manual labour (Ahmed et al., 2014) In Burkina Faso, however, community level support was expressed through accepting practices, encouragement to implement lessons learnt and by asking beneficiaries for advice or being seen as role models. One beneficiary mentioned that ‘The people are interested; they often ask us questions and advice. It pleases us to inform others’.

*Theme 3: Multicomponent asset transfer programmes can encourage more sustainable programme impact in improving well‐being and beneficiary empowerment*.

To maximise the effect of asset transfers, interventions could incorporate different components such as legal support for beneficiaries, compulsory saving opportunities, microcredit access, and supplementary cash benefits. Training and capacity building form an important part of these components and is discussed in theme four below. The multiple intervention components complement one another to support livelihoods, social status, beneficiary confidence and awareness of beneficiary rights. In some cases, allowing women to work for their benefits instilled a greater sense of pride. An additional factor to consider is the size of the transfer benefit because it determines how beneficiaries andomi them. There are generally reported mixed preferences for asset and cash transfers. In the case of physical assets, there is less reported undue interference from male counterparts within the household (Olney et al., 2015).

Asset transfer initiatives that are designed to enhance women's well‐being and empowerment may require additional components to fully realise this objective. For instance, the IGVGD programme in Bangladesh included a microfinance facility which allowed women to use loans for a multitude of purposes (Ahmed et al., 2007). One woman reported greater economic involvement because: ‘After I had joined VGD, I received a 4,000‐taka loan from BRAC. With that money I rented a small piece of land for 2 years. My husband and I grow potatoes, chilies, and vegetables on that land. We sell most of what we produce’. Another beneficiary enjoyed the same satisfaction as she proudly said: ‘I became a BRAC member right after I had received the [IGVGD] card. I got training from BRAC on how to develop a nursery (to raise vegetable seedlings). After six months of training, I borrowed 3,000 taka from BRAC and started a nursery. Many people come to see my nursery. The current value of the nursery is 10,000 to 15,000 taka’ (Ahmed et al., 2007). In the TUP programme, women in focus group discussions expressed that improvements in economic circumstances and training across a range of areas (including nutrition, hygiene and literacy) created a greater sense of empowerment (Sarkar & Karim, 2018). Importantly, the design of programme activities such as compulsory training sessions could be tailored in a manner that does not interfere with standard income‐generating activities such as usual labour participation (Ahmed et al., 2007). The additional provision of mobile phones for beneficiary communication with implementation staff for the TMRI programme was reported as satisfactying for most of the participants. Preferences on asset type across the included asset transfer programmes revealed diverse views from beneficiaries (Ahmed et al., 2007, 2014). Across the qualitative evidence base, asset provision was often paired with cash benefits and the overall views on which options were preferred are mixed. For example, beneficiaries of the TMRI largely preferred their current benefit package while the majority of IGVGD preferred food packages (Ahmed et al., 2007, 2014).

*Theme 4: The provision of quality training promotes knowledge and skills of participants to adequately carry out asset transfer related activities. Compensation of trainers is a key factor to consider improving the adequate delivery of quality training*.

A consistent theme that we identified was the adequate provision and quality of agricultural training to asset transfer beneficiaries. The embedding of a training and capacity‐building component within asset transfer programmes was reflected as a positive design feature throughout the identified evidence base. In Burkina Faso, beneficiaries of the E‐HFP conveyed that the prescribed training led to betterment in practical skills and business knowledge, knowledge of own rights, social status and social capital. One beneficiary of the E‐HFP explained that: ‘…the training is good quality because since receiving the training I no longer have problems with my vegetable production’ (Olney et al., 2015). Qualitative evidence on the impact of E‐HFP on vegetable production was mainly positive as one woman explained that: ‘Before we didn't produce vegetables during the dry season. But now thanks to the programme we have vegetables all year long’. A master agriculture trainer expressed: ‘…we were able to touch on the points that I wanted to further understand like raising poultry and small animals’. There were some exceptions who found the quality of the training to be poor as another trainer observed that: ‘the training leader was not a specialist in gardening’. Overall, participating farmers suggested that a more participatory and ‘hands‐on’ pedagogy and more time for training would improve their learning and retention of information. Additionally, qualitative findings from the IGVGD initiative indicated that training activities should be arranged conveniently and on a frequent basis so that they do not conflict with existing income‐generating activities (Ahmed et al., 2007).

Trainers’ compensation and motivation of trainers to carry out appropriate agricultural training should be considered as this may suppress the learning process of beneficiaries. Sentiments by two trainers indicated low remuneration which was not in tandem with the workload, ‘What can I do about it? The tasks are heavy, but I have to do it; it's my job’. Another echoed a similar view: ‘If you look at the work we do, it's a small compensation. We train them and also follow‐up with them; it's not easy and everything is expensive these days’ (Olney et al., 2015).

##### 5.3.1.12 Discussion

The quantitative evidence base contains 10 studies (*k *=* *10). Results from six analysed outcomes from the quantitative meta‐analysis are positive and statistically significant in three areas: (1) women's access to and ownership of assets, credit and income, (2) women's access to decent work (formal and informal employment) and (3) increased participation in decision making by women at the household or community level. While the improved quality of relationships between women and their household and community members and women's empowerment index were positive, these results were not statistically significant. There was no effect on the occurrence of intimate partner violence or women attitudes, self‐image and confidence.

Some possible explanations that emerge from the qualitative findings can be andomized as follows:

- The provision of assets or tools for cultivation or livestock farming can generally support asset endowment, creditworthiness and income. If assets are andomiz adequately, it opens pathways to credit use options and income‐generating activities if used productively.
- The location of access to assets is an important factor for both participation and productive use. If training or farming facilities are inconveniently selected or costly to access for beneficiaries, it can hamper the active participation of beneficiaries.
- Poor quality of training for farmers can hinder willingness to participate. Similarly, the inadequacy of training (on both duration and content) may be a perverse problem—particularly for less experienced farmers (or farming communities).
- Substandard equipment quality and inadequate inputs can impede productivity for beneficiaries and delay programme activities
- Programme activities (including compulsory training) may affect time use preferences for women. Activities that disrupt usual day‐to‐day work obligations can be ineffective in recruiting and retaining target beneficiaries unless women see it as being beneficial.
- On the lack of impact on the prevalence of IPV, while asset transfer programmes can support asset endowment for beneficiaries more directly, it seems limited on behavioural and social changes. Moreover, if there are poverty reduction effects, household relationships can be less constrained.
- Due to the ownership and management of animals and crops, women can experience improved decision‐making and social status within households and around communities.
- Multicomponent interventions can be an important design strategy to ensure the productive and sustained use of assets. The use of training coupled with the provision of loans and saving facilities can lead to more sustained benefits. Interventions should be tailored to target outcomes of interest.
- The qualitative evidence base suggests that beneficiaries tend to prefer the asset type that they have been assigned to or endowed with.

##### 5.3.1.13 Summary of findings and discussion

We included 10 studies in five countries in South Asia and Sub‐Saharan Africa that evaluated the effect of asset transfers. We examined effects on the following outcomes: Women have increased access and ownership to assets, credit and income, women and girls have equitable access to livelihood support services, Initiatives supported that facilitate women to access decent work (formal and informal employment), including people with disabilities, Increased participation in decision making by women at the household or community level, including during crisis response and the Empowerment/Equality Index. Our included studies reported against all three secondary outcomes (Resources, Agency and Achievement) and seven of the nine immediate outcomes for our review.

The ten linked qualitative studies reported on four different themes that highlighted contextual and natural factors that impeded or promoted the success of the intervention. General climate barriers, including water access and arid climates can impede the success of an asset transfer, especially in an agricultural setting. Traditional gender roles in a community may also hinder or support a women's ability to andomized on her assets. Lastly, multicomponent asset transfer interventions that combined the transfer with training or an additional component often had the most positive outcomes. The IGVGD programme in Bangladesh, for example, was successful by combining microfinance facilities and other linked components. Overall, the GRADE assessments generally indicate a range of very low to high certainty in this body of evidence.

From the quantitative and qualitative evidence base, we find that asset transfer was statistically significant for women accessing and owning assets, credit and income, which is buttressed by the above qualitative findings. The summary of quantitative findings along with the GRADE certainty of evidence assessments are presented in Table 18 (Table 13).

**Table 13:** GRADE summary of findings and certainty of evidence on asset transfers

Certainty assessment							Sample size	Effect	Certainty	Importance
No. of studies	Study design	Risk of bias	Inconsistency	Indirectness	Imprecision	Other considerations		Absolute (95% CI)		
*(AB1) Increased capacity of women to understand and use financial, banking and business services effectively*										
1	RCT	Not serious	Serious^a^	Not serious	Not serious	Strong association	1053	SMD 0.62SD higher (0.5 higher to 0.74 higher)	⊕⊕⊕⊕ HIGH	Important, but not critical
*(AB2) Women have increased access and ownership to assets, credit and income*										
10	RCT‐7 QED‐3	Serious^b^	Very serious^c^	Not serious	Serious	None	20,864	SMD 0.34 SD higher (0.22 higher to 0.46 higher)	⊕◯◯◯ VERY LOW	Important, but not critical
*(AC1) More women engaged in other micro, small and medium‐sized enterprises*										
1	RCT	Very serious^d^	Serious^a^	Not serious	Not serious	None	1146	SMD 0.24 SD higher (0.13 higher to 0.36 higher)	⊕◯◯◯ VERY LOW	Important, but not critical
*(AC2) Initiatives supported that facilitate women to access decent work (formal and informal employment), including people with disabilities*										
5	RCT‐4 QED‐1	Serious^e^	Very serious^f^	Not serious	Serious^g^	None	16,178	SMD 0.1 SD higher (0 to 0.2 higher)	⊕◯◯◯ VERY LOW	Important, but not critical
*(AC3) Improved capacity of women entrepreneurs*										
1	RCT	Not Serious	Serious^a^	Not Serious	Serious^h^	None	1140	Two positive effect estimates with a 95% CI range of 0.04 to 0.58	⊕⊕◯◯ LOW	Important, but not critical
*(BA1) Women have improved success in the workplace*										
1	RCT	Serious^i^	Serious^a^	Not serious	Serious^j^	None	18,387	One positive and one negative effect estimate with a 95% CI range of −0.08 to 0.14	⊕◯◯◯ VERY LOW	Important, but not critical
*(BA2) Women have more and better control over their bodies and sexual health*										
1	QED	Serious^k^	Serious^a^	Not serious	Serious^l^	None	589	Five positive and one negative effect estimate with a 95% CI range of −0.22 to 0.38	⊕◯◯◯ VERY LOW	Critical
*(BA3) Women have increased freedom of movement and association*										
1	QED	Serious^m^	Serious^a^	Not serious^n^	Very serious^o^	None	589	Seven negative and three positive effect estimates with a 95% CI range of −0.46 to 0.28	⊕◯◯◯ VERY LOW	Limited importance
*(BA8) Reduced instances of child or forced marriage*										
1	RCT	Not serious	Serious^a^	Not serious	Not serious	None	543	SMD 0.14 SD lower (0.31 lower to 0.02 higher)	⊕⊕⊕◯ MODERATE	Critical
*(BB1) Increased participation in decision making by women at the household or community level, including during crisis response*										
5	RCT‐4 QED‐1	Serious^p^	Not serious	Not serious	Not serious	None	14,638	SMD 0.07 SD higher (0.04 higher to 0.11 higher)	⊕⊕⊕◯ MODERATE	Limited importance
*(BB2) Women participate more in their community*										
1	RCT	Very serious^q^	Serious^a^	Not serious	Not serious	None	1105	SMD 0.27 SD higher (0.16 higher to 0.39 higher)	⊕◯◯◯ VERY LOW	Limited importance
*(CA2) Increased representation of women in local and subnational civil and political processes, including during peacebuilding and post conflict restoration*										
1	RCT	Serious^r^	Serious^a^	Not serious	Serious^s^	None	1147	Four positive effect estimates with a 95% CI range of −0.09 to 0.60.	⊕◯◯◯ VERY LOW	Limited importance
*(CB3) Women have improved attitudes, self‐image and confidence*										
2	RCT‐2	Serious^w^	Serious^x^	Not serious	Serious^y^	None	20,384	SMD 0.24 SD higher (0.16 lower to 0.64 higher)	⊕◯◯◯ VERY LOW	Limited importance
*(CB4) Improved attitudes and increased support for women's economic, social and human rights by men, household and family members and community members*										
1	RCT	Very serious^z^	Serious^a^	Not serious	Not serious	None	900	SMD 0.01 SD lower (0.14 lower to 0.12 higher)	⊕◯◯◯ VERY LOW	Limited importance
*(CB7) Reduced frequency and distribution of types of violence by an intimate partner*										
3	RCT‐2 QED‐1	Serious^t^	Not serious	Not serious	Not serious	None	3256	SMD 0.00 SD (0.07 lower to 0.07 higher)	⊕⊕⊕◯ MODERATE	Critical
*(CB8) Improved quality of relationships between women and their household and community members*										
3	RCT‐2 QED‐1	Very serious^u^	Serious^v^	Not serious	Not serious	None	2804	SMD 0.05 SD higher (0.03 lower to 0.13 higher)	⊕◯◯◯ VERY LOW	Limited importance
*(CC1) Empowerment/Equality Index*										
3	RCT‐3	Serious^aa^	Serious^ab^	Not serious	Serious^ac^	None	8981	SMD 0.22 SD higher (0.03 lower to 0.47 higher)	⊕◯◯◯ VERY LOW	Important, but not critical

Abbreviations: CI, confidence interval; GRADE, Grading of Recommendations, Assessment, Development and Evaluations; QED, quasi‐experimental design; RCT, andomized controlled trial; RoB, risk of bias; SMD, andomizedi mean difference.

^a^ All single studies downgraded once for inconsistency.

^b^ Downgraded once because two studies present high risk of bias, and others present some concern for bias. That said, when the high risk of bias‐studies were removed, the effect estimate only changed by 0.02.

^c^ Downgraded twice for (1) variation of points estimates: points estimates on the same side of the threshold, (2) overlaps of CI: no systematic overlaps of CI, (3) $I^{2}=92.85\%$, (4) test of stat: the true outcomes appear to be heterogeneous.

^d^ Based on a single study with a final risk of bias score rated as high. Deviations from intended intervention was a plausible risk.

^e^ Downgraded once since all of the studies but one was an RCT and none of them were high risk of bias. Three studies have some concerns related to risk of bias.

^f^ Downgraded twice as (1) all studies but one are on the same side of the threshold but the C is do not systematically overlap, (2) $I^{2}=87.92\%$ and the *Q*‐test concludes that the true outcomes appear to be heterogeneous.

^g^ Downgraded once as the CI is quite large although *p *=* *0.05 and sample size >400.

^h^ Downgraded due to relatively wide variance of point estimates across the two effect sizes: one is large, the other small (b) No overlap of CI.

^i^ Based on a single study with a final risk of bias score rated as medium for concerns with deviation from intended intervention.

^j^ Downgraded due to wide variance of point estimates across the two effect sizes on both sides of the 0.00 threshold: −0.025 versus 0.086 (b) No overlap of CI.

^k^ Downgraded because four of seven RoB criteria have been assessed as unclear: Randomness of assignment, performance bias, outcome measurement bias and reporting bias.

^l^ Downgraded due to very wide range of Cis across the point estimates.

^m^ Based on a single study with a final risk of bias score rated as medium. Four of seven RoB criteria have been assessed as unclear: Randomness of assignment, performance bias, outcome measurement bias and reporting bias.

^n^ Downgraded due to a wide range of point estimates across both sides of the threshold and Cis that do not overlap.

^o^ Downgraded due to a wide range of point estimates across both sides of the threshold and Cis that do not overlap.

^p^ Downgraded once because two studies are of high risk of bias. That said, it should be noted that those two studies only contribute a combined 15% of the weight of the meta‐analysis. Other studies are either of uncertain or low risk.

^q^ Based on a single study with high risk of bias due to not addressing selection bias to a satisfactory extent.

^r^ Downgraded once as although the three studies are RCTs and two score low risk of bias one has a high risk of bias.

^s^ Downgraded because, despite being all positive, the effect estimates vary widely and have nonoverlapping Cis.

^t^ Downgraded because one study presents high risk of bias. Additionally, a study of uncertain risk of bias is weighted to contribute 73% to the meta‐analysis.

^u^ Downgraded twice because >80% of the meta‐analysis is coming from high risk of bias studies. If those studies are removed, the group is left with one QED of uncertain bias.

^v^ Downgraded to account for the wide range of Cis across the three studies, crossing both sides of the threshold.

^w^ Downgraded once since out of the two studies both of them are RCTs but one is medium and the other one is low risk of bias.

^x^ Downgraded once (1) both studies are on the same side of the threshold but the CI are not overlapping (2) $I^{2}=97.55\%$ but there was no significant amount of heterogeneity in true outcomes.

^y^ Downgraded once since *p *=* *0.24 although sample size >400.

^z^ Based on a single study with a final risk of bias score rated as high. Deviations from intended intervention is a plausible risk.

^aa^ Downgraded once as although the three studies are RCT and 2 score low risk of bias one has a high risk of bias.

^ab^ Downgraded twice as (1) all the studies are on the same side of the threshold but the CI are not systematically overlapping, (2) $I^{2}=95.468\%$ and *Q*‐test concludes that the true outcomes appear to be heterogenous.

^ac^ Downgraded once as the CI is very large and *p *=* *0.08 although sample size >400.

#### 5.3.2 Cash transfers

Cash transfers are a form of social protection intervention that provide cash to vulnerable households or individuals to reduce vulnerability, poverty and risk and to improve the human capital of the poor. They allow beneficiary communities, households and/or individuals to protect themselves from economic, demographic and seasonal shocks, improve nutrition and food security, and increase asset ownership and expenditure on basic necessities (Miller et al., 2008). They are interventions that either require beneficiaries to comply with specific conditions to be eligible for a money or cash transfer that will allow them to respond to specific needs of resources, or do not require any specific actions to be undertaken by targeted beneficiaries to be eligible for a money or cash transfer that will allow them to respond to specific needs of resources (Hemsteede, 2018).

##### 5.3.2.1 How do cash transfers affect gender equality, women's empowerment and Peace outcomes?

**Figure 33:**
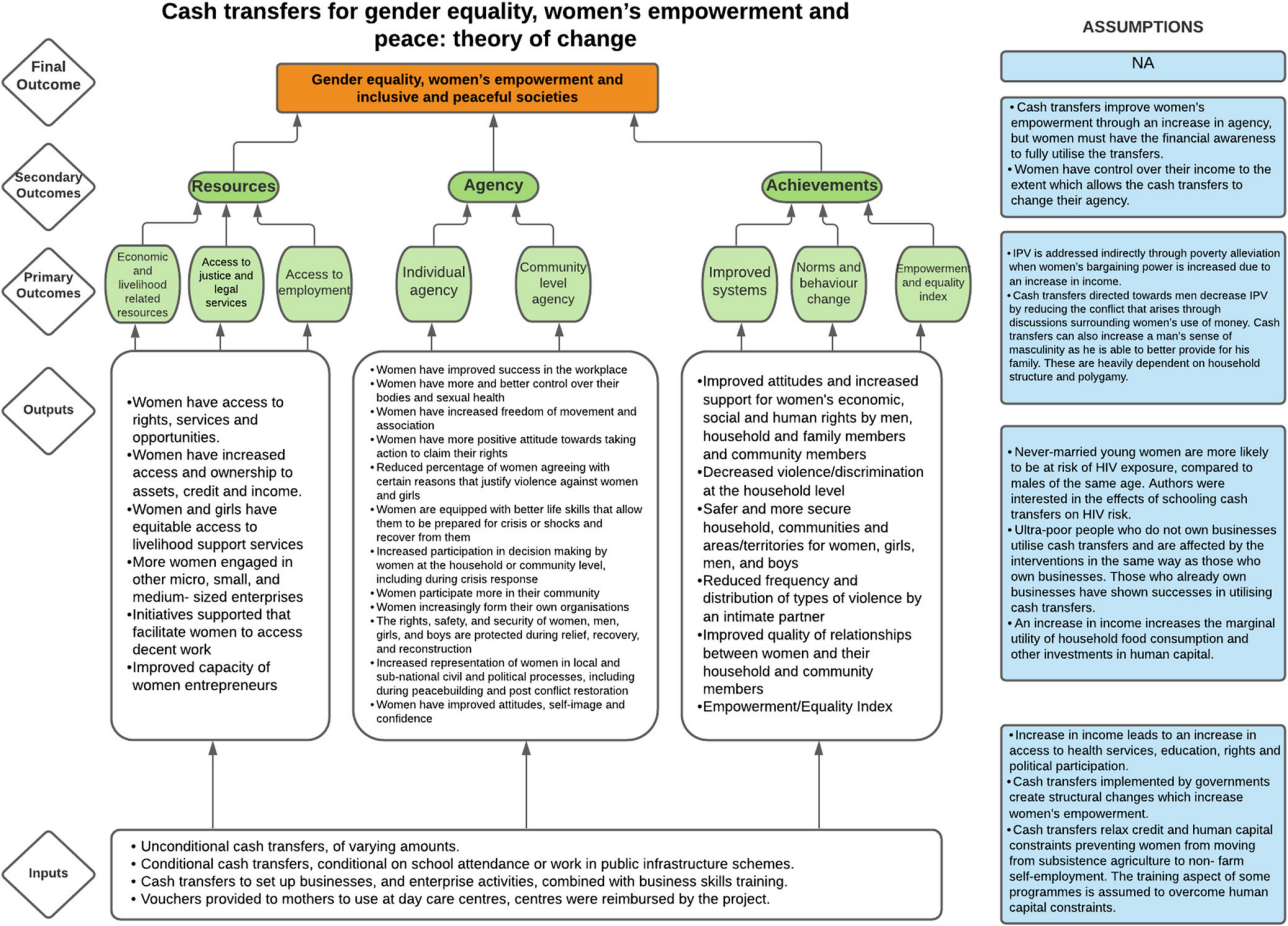
Cash transfers for gender equality, women's empowerment and peace: Theory of change

Figure 33 maps out the causal chain of how cash transfers may improve gender equality, women's empowerment and peace outcomes.

Cash transfers can work under two separate ToCs, depending on whether the transfer is directed towards the man or woman of the household. When cash transfers, conditional or unconditional, are directed towards the woman within the household, this can lead to two changes. First, the increase in income can empower women by increasing their agency and reducing their reliance on their husbands. An increase in their bargaining power within the household leads to greater agency. Secondly, cash transfer programmes lift two constraints faced by women. Firstly, it lifts the financial constraint which prevents women from moving from subsistence agriculture to nonfarm self‐employment. Secondly, programmes which incorporate business skills training lift the human capital constraints faced by women where they do not have the required knowledge to andomi the transfers to set up a business.

Cash transfers directed towards men work on a different ToC. The increase of income can lead to a decrease in conflict between the man and woman within the house regarding the discussion and use of money, specifically when it comes to women asking for money. The increase of income can also increase the man's dominance as he is able to more easily provide for his household. The decrease in household conflict can lead to greater empowerment for women.

##### 5.3.2.2 Description of included studies

We included 21 studies reported in 29 different papers evaluating the effect of 12 programmes. We included more than one paper that evaluated the same programme if the author(s) reported different outcomes over several papers.

##### 5.3.2.3 Population

As previously mentioned, cash transfers can be targeted towards men or women as the main beneficiaries. The Benazir Income Support Programme (BISP) specifically targeted women in households which scored under a certain level on a proxy means test. Conditional cash transfers, conditional on school attendance target households with children, while in general, all transfers target the poor and ultra‐poor.

The included studies evaluated 12 different programmes and trials in MENA, South Asia and Sub‐Saharan Africa, a total of 11 countries including: Burkina Faso, Cameroon, Egypt, Ethiopia, Kenya, Malawi, Mali, Pakistan, South Sudan, Uganda and Zambia.

##### 5.3.2.4 Intervention, inputs and activities

The included studies evaluated a range of different cash transfer activities and inputs including:

- Unconditional cash transfers (*n* = 13): Any programme that provided an unconditional cash transfer. These transfers are a fixed, but varying, amount and are not conditional on any requirements.
- Conditional cash transfers (*n* = 5): Any programme that provided a conditional cash transfer. This is a sum of money in exchange for meeting certain requirements such as a child's school attendance or participation in a public infrastructure work scheme.
- Day care vouchers (*n* = 1): Day care vouchers were provided to mothers who were able to use them to send their child to day care. The day care centres were reimbursed for the vouchers (Table 14).

**Table 14:** Features of included cash transfer studies

Study	Activity/input	Length of treatment	Frequency of cash transfer
Ambler and De Brauw (2017)	Cash transfer of 1000 PKR per month, paid in quarterly instalments of 3000 PKR	Unclear	Quarterly
Baird et al. (2010)	Conditional cash transfer of varying amounts, conditional on school attendance, as well as an unconditional cash transfer of varying amounts	24 months	Monthly
Baird et al. (2015)	Conditional cash transfer of varying amounts, conditional on school attendance, as well as an unconditional cash transfer of varying amounts	24 months	Monthly
Baird et al. (2009)	Conditional cash transfer of varying amounts, conditional on school attendance, as well as an unconditional cash transfer of varying amounts	24 months	Monthly
Baird et al. (2013)	Conditional cash transfer of varying amounts, conditional on school attendance, as well as an unconditional cash transfer of varying amounts	24 months	Monthly
Blattman et al. (2013)	$150 USD grants, as well as business skills training and planning, and on‐going supervision to implement the business plan	24 months	One time transfer
Blattman et al. (2016)	$150 USD grants, as well as business skills training and planning, and on‐going supervision to implement the business plan	24 months	One time transfer
Bonilla et al. (2017)	$24 USD bi‐monthly cash transfers	48 months	Bi‐monthly
Breisinger et al. (2018)	Cash transfers, the amount of which is dependent on the number of children and their schooling level	Unclear	Monthly
Brooks et al. (2020)	One‐time cash transfer of 5000 KES	½ month	One time transfer
Clark et al. (2019)	Mothers were offered vouchers to be used at day care centres, day care centres were also offered improvements	14 months	Monthly
Eze (2019)	Distribution of cash transfers which totalled 654.1 billion FCFA	96 months	Not reported
Gelagay and Lecoutere (2019)	Provision of a conditional cash transfer based on work in a public infrastructure scheme	Unclear	Monthly
Gobin et al. (2017)	$100 USD cash transfer to set up a microenterprise, alongside business skills training. An additional $50 USD transfer for support conditional on the microenterprise having been set up	14 months	One time transfer with one additional transfer for qualifying participants
Green et al. (2015)	Business start‐up grant of $150 USD, as well as business skill training	Unclear	One time transfer
Green et al. (2016)	Business start‐up grant of $150 USD, as well as business skill training	Unclear	One time transfer
Handa et al. (2015)	Unconditional cash transfer of 1500 Kenyan shillings per household	48 months	Monthly
Haushofer et al. (2019)	Unconditional cash transfer of $709 USD per beneficiary	24 months	Monthly
Haushofer and Shapiro (2013)	Unconditional cash transfer of $709 USD per beneficiary	24 months	Monthly
Haushofer and Shapiro (2016)	Unconditional cash transfer of $709 USD per beneficiary	24 months	Monthly
Haushofer and Shapiro (2018)	Unconditional cash transfer of $709 USD per beneficiary	24 months	Monthly
Heath et al. (2020)	Cash transfers, group training sessions on different themes and nutrition packages	36 months	Quarterly
Iqbal et al. (2020)	Conditional cash transfers, the pilot also consisted of provision of training, microfinance and health insurance	132 months	Quarterly
Iqbal et al. (2020)	Conditional cash transfers, the pilot also consisted of provision of training, microfinance and health insurance	132 months	Quarterly
Karimli et al. (2019)	Four components: (1) savings, (2) technical skills training, (3) cash transfers with livelihood selection and (4) ongoing support.	24 months	One time transfer
Karimli et al. (2021)	Four components: (1) savings, (2) technical skills training, (3) cash transfers with livelihood selection and (4) ongoing support	24 months	One time transfer
Malaeb and Uzor (2017)	Business start‐up grant of $150 USD, as well as business skill training	Unclear	One time transfer
Mekonnen (2017)	Cash transfers to implement innovative agricultural production systems	24 months	Unclear
Müller et al. (2019)	Unconditional cash grant with business skill training	14 months	Unclear
Natali et al. (2016)	Unconditional cash transfer of $12 USD per month	34 months	Monthly

Abbreviations: FCFA, Central African CFA franc; KES, Kenyan shilling; PKR, Pakistani rupee; USD, United States dollar.

##### 5.3.2.5 Comparison

All included studies compared treated groups to comparison groups receiving no intervention. Nine studies included multiple treatment arms.

##### 5.3.2.6 Outcomes

The included studies reported on several relevant outcomes:

- Women have increased access and ownership to assets, credit and income (*n* = 13): Women can apply for, receive and manage assets/credit and income and have support to manage, claim and execute their assets without pressure or influence from external actors, including male family members, husbands and cultural leaders.
- Initiatives that facilitate women to access decent work (formal and informal employment), including people with disabilities (*n* = 7): Women are able to apply for, receive and work in jobs (and have support for the above), without discrimination for sex, gender or other identifying factors, including development of skills for improved access.
- Women have increased freedom of movement and association (*n* = 5): Women and girls can move freely and meet within their household, community, and area/territory without fear of attack or discrimination.
- Increased participation in decision making by women at the household or community level, including during crisis response (*n* = 11): Women take part in all or any step of the decision‐making process at the household community and district level, but also are able to meaningfully take part and have influence on the final decision, including in crisis response.
- Women participate more in their community (*n* = 3): Women increase their influence and representation in the life of their community. This can include increased participation in local/district government, cultural institutions and representation in various sectors and having a greater influence and involvement in the activities and pursuits of the community, district, and so on.

The majority of studies in this category (*n* = 14) measured economic and livelihood related resources through women having increased access and ownership to assets, credit and income and women and girls having equitable access to livelihood support services.

The majority of studies (*n* = 12) measured community level agency through increased participation in decision making by women at the household or community level, including during crisis response; women participate more in their community; women increasingly form their own organisations, and the rights, safety, and security of women, men, girls, and boys are protected during relief, recovery, and reconstruction.

The majority of studies (*n* = 11) measured individual agency through women have improved success in the workplace; women have more and better control over their bodies and sexual health; women have increased freedom of movement and association; women have more positive attitude towards taking action to claim their rights; reduced percentage of women agreeing with certain reasons that justify violence against women and girls and women are equipped with better life skills that allow them to be prepared for crisis or shocks and recover from them.

The majority of studies (*n* = 11) also measured norms and behaviour change through women have improved attitudes, self‐image and confidence; improved attitudes and increased support for women's economic, social and human rights by men, household and family members and community members; decreased violence/discrimination at the household level; safer and more secure household, communities and areas/territories for women, girls, men, and boys; reduced frequency and distribution of types of violence by an intimate partner and improved quality of relationships between women and their household and community members.

The majority of studies (*n* = 7) measured access to employment through more women engaged in other micro, small, and medium‐sized enterprises; initiatives supported that facilitate women to access decent work (formal and informal employment), including people with disabilities and improved capacity of women entrepreneurs.

Studies (*n* = 5) also measured Empowerment/Equality Index through Empowerment/Equality Index.

Other outcomes measured included: (*n* = 1) measured improved systems through increased representation of women in local and subnational civil and political processes, including during peacebuilding and post conflict restoration; (*n* = 1) measured access to justice and legal services through women have access to rights, services and opportunities.

Our list of 44 outcomes reported in our included studies was grouped into nine immediate outcomes directly contributing to the three secondary outcomes as per Kabeer's dimensions of women's empowerment and gender equality (Resources, Agency and Achievement). The division of the immediate and secondary outcomes is reported in Table 20 (Table 15).

**Table 15:** Summary of secondary and immediate outcomes for cash transfers

Secondary outcome category	Immediate outcome	Number of studies
Resources material, human and social resources which serve to enhance the ability to exercise choice	Access to justice and legal services	0
	Economic and livelihood related resources	13
	Access to employment	7
Agency ability to define one's goals and act upon them and andomizedized decision‐making;	Individual agency	12
	Community level agency	13
	Institutions supporting agency	1
Achievement ways of being and doing which can be andomiz by different individuals	Improved systems	3
	Norms and behaviour change	11
	Empowerment index	4

##### 5.3.2.7 Study design

**Figure 34:**
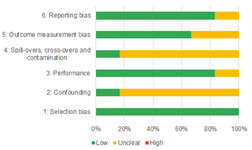
Cash transfer studies quasi‐experimental design risk of bias assessment

Four of our cash transfer studies used a QED (Ambler & De Brauw, 2017; Brooks et al., 2020; Eze, 2019; Iqbal et al., 2020). All four studies were assessed as having some concerns related to risk of bias. As detailed in Figure 34, high risk of bias was not identified in any RoB domains. However, the quality assessment of the studies was limited by the lack of discussion by the authors regarding potential issues with the study design that could have resulted in confounding of the results; not enough information was provided about data collection and treatment implementation processes, so that issues about implementation, such as spill‐overs and crossovers, and about outcome measurement could not be discarded. This was the case in particular of Gelagay and Lecoutere (2019) and Karimli et al. (2019). We could not identify any limitation related to the assignment of the intervention and selection of the comparison group in any of the included studies, so that all included studies were assessed as low risk of selection bias.

Twenty of the included cash transfer studies used an experimental design. This included Aker (2016), Baird et al. (2009, 2010, 2013), Blattman et al. (2013, 2016), Bonilla et al. (2017), Clark et al. (2019), Gobin et al. (2017), Green et al. (2015), Haushofer and Shapiro (2013, 2016, 2018), Haushofer et al. (2019), Heath et al. (2020), Karimli et al. (2021), Malaeb and Uzor (2017), Mekonnen (2017), Müller et al. (2019), Natali et al. (2016) and Sarah et al. (2017).

**Figure 35:**
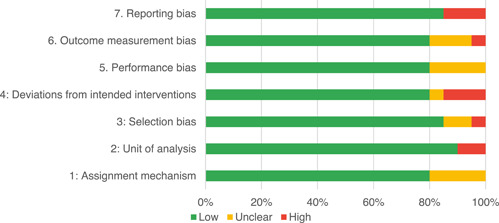
Cash transfer studies andomized controlled trial risk of bias assessment

Approximately 25% of the papers (*n* = 5) were assessed as having a high risk of bias and 30% (*n* = 6) as having some concerns, but we did not identify any major limitation for 45% of the included RCT papers evaluating cash transfers (*n* = 9). As detailed in Figure 35, the limitations that were associated with high risk of bias were identified in five RoB domains (unit of analysis, selection, deviation from intended intervention, outcome measurement and reporting), yet in all categories the number of papers presenting high risk or being unclear were four or less.

The most common issues in the RCT studies evaluating cash transfers were due to deviations from intended interventions and reporting bias. Deviations from intended interventions can occur when there are implementation issues that might lead the control participants to receive the treatment, as when vouchers delivered can be traded or transferred (Clark et al., 2019) or when there is a risk of contamination because participants could not be isolated from other programs (Karimli et al., 2021). Reporting bias is observed when authors of RCT studies do not discuss baseline balance between treatment and control groups (e.g., Karimli et al., 2021; Malaeb & Uzor, 2017), or when multiple treatment arms are not differentiated in the analysis (Clark et al., 2019).

Other limitations that we identified in RCT studies evaluating cash transfers were the fact that a few had assigned treatment at the cluster level (e.g., village) but had analysed data at the individual level without adjusting for clustering (Clark et al., 2019; Malaeb & Uzor, 2017); another study shows a large difference in attrition rates between treatment and control groups, which poses a risk of selection bias given that authors could not rule out that it was related to the intervention assignment (Clark et al., 2019); further, one study was scored as high risk of bias because of measurement issues related to the use of self‐report outcome data which is susceptible to recall and reporting bias (Blattman et al., 2013).

The most common issues in the RCT studies evaluating cash transfers were due to deviations from intended interventions and reporting bias. Deviations from intended interventions can occur when there are implementation issues that might lead the control participants to receive the treatment, as when vouchers delivered can be traded or transferred (Clark et al., 2019) or when there is a risk of contamination because participants could not be isolated from other programs (Karimli et al., 2021). Reporting bias is observed when authors of RCT studies do not discuss baseline balance between treatment and control groups (e.g., Karimli et al., 2021; Malaeb & Uzor, 2017), or when multiple treatment arms are not differentiated in the analysis (Clark et al., 2019).

##### 5.3.2.8 Qualitative studies, process evaluations and project documents

We identified 25 additional documents related to nine programmes covered by the cash transfer studies.

- Child Grant Programme (Zambia): two process evaluations and one descriptive quantitative document
- Productive Safety Net Programme (PSNP) (Ethiopia): one qualitative and two descriptive documents
- Zomba Cash Transfer Programme (ZCTP) (Malawi): two descriptive quantitative documents
- Takaful and Karama programme (Egypt): three qualitative documents
- BISP (Pakistan): two descriptive quantitative and one qualitative document
- Unnamed Cash Transfer Programme (Kenya): one qualitative document
- Women's Income Generating Support (WINGS) (Uganda): four descriptive quantitative documents
- Rural Entrepreneur Access Program (Kenya): four descriptive quantitative documents
- Trickle Up (Burkina Faso): two descriptive quantitative documents

The majority (*n* = 14) of studies under this intervention group were of moderate empirical quality, followed by high quality (*n* = 6) and low quality (*n* = 5).

##### 5.3.2.9 Synthesis of findings

The following subsection presents the results of effectiveness of cash transfers on gender equality, women's empowerment and peace outcomes.

##### 5.3.2.10 Quantitative findings

*Effects of cash transfers on women's access to and ownership of assets, credit and income*

**Figure 36:**
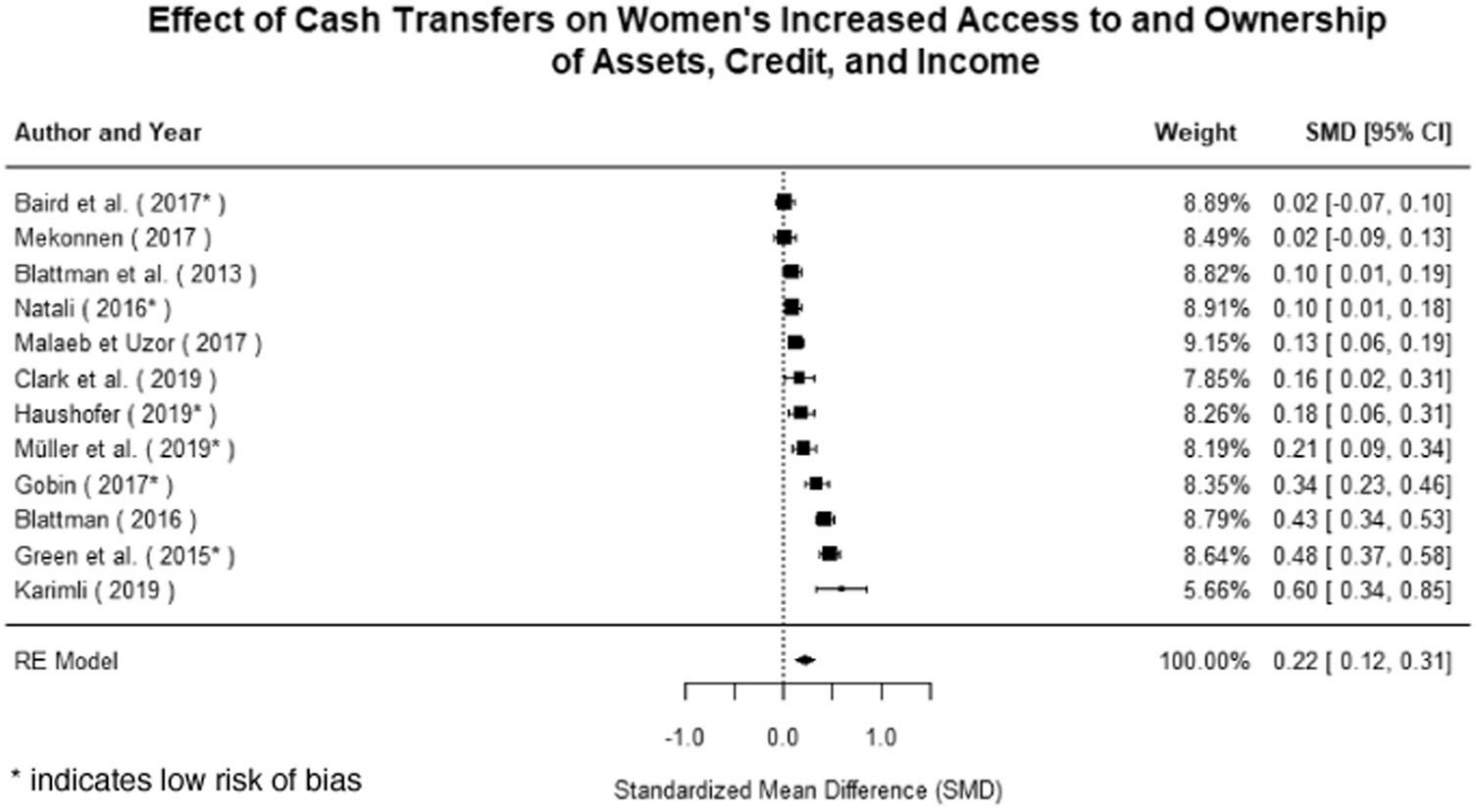
CTAB2a: Forest plot showing the observed outcomes and the estimate of the random‐effects model. CI, confidence interval

We included a total of k=12 studies in the analysis. We assessed six studies as low risk of bias, three as some concerns, and three as high risk of bias. The observed outcomes ranged from 0.02 to 0.60. The estimated average outcome based on the random‐effects model was $\hat{\mu }=0.22$ (95% CI: 0.12 to 0.31). Therefore, the average outcome differed significantly from zero (z=4.50, p<0.01). A forest plot showing the observed outcomes and the estimate based on the random‐effects model is shown in Figure 36 (CTAB2a).

According to the Q‐test, the true outcomes appear to be heterogeneous (Q(11)=109.31, p<0.01, ${\hat{\tau }}^{2}=0.02$, $I^{2}=89.94$%). An examination of the tudentized residuals revealed that none of the studies had a value larger than ±2.87 and hence there was no indication of outliers in the context of this model. According to the Cook's distances, none of the studies could be considered to be overly influential.

We were able to test several moderators for this outcome category. First, we tested whether effects from studies using conditional cash transfers differed from those using unconditional cash transfers. We found no difference between the two intervention conditions ($\hat{B}=0.009,p=0.94$ [95% CI: −0.20 to 0.22]). Studies with a high risk of bias were not significantly different than studies with some concerns or low risk of bias ($\hat{B}=-0.12,p=0.29$ [95% CI: −0.35 to 0.10]). In addition, there was no difference in the size of effects between programmes that used conditional versus unconditional cash transfers ($\hat{B}=-0.02,p=0.80$ [95% CI: −0.24 to 0.19]). There was also no moderation by the length of exposure to the intervention ($\hat{B}=-0.003,p=0.32$ [95% CI: −0.01 to 0.003]), nor the length of the evaluation follow‐up period ($\hat{B}=-0.004,p=0.32$ [95% CI: −0.01 to 0.004]). Finally, publication year was also not a significant moderator ($\hat{B}=0.006,p=0.83$ [95% CI: −0.05 to 0.07]). Study design could not be tested as a moderator because all of the studies were experimental designs, and we also could not test for differences between adjusted and unadjusted models because only one study presented an unadjusted effect.

**Figure 37:**
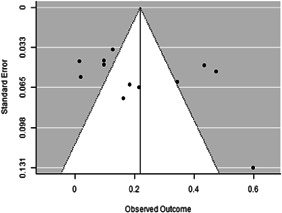
CTAB2b: Funnel plot

A funnel plot of the estimates is shown in Figure 37 (CTAB2b). Neither the rank correlation nor the regression test indicated any funnel plot asymmetry (p=0.25 and p=0.05, respectively).

*Effects of cash transfers on women and girls having equitable access to livelihood support services*

**Figure 38:**
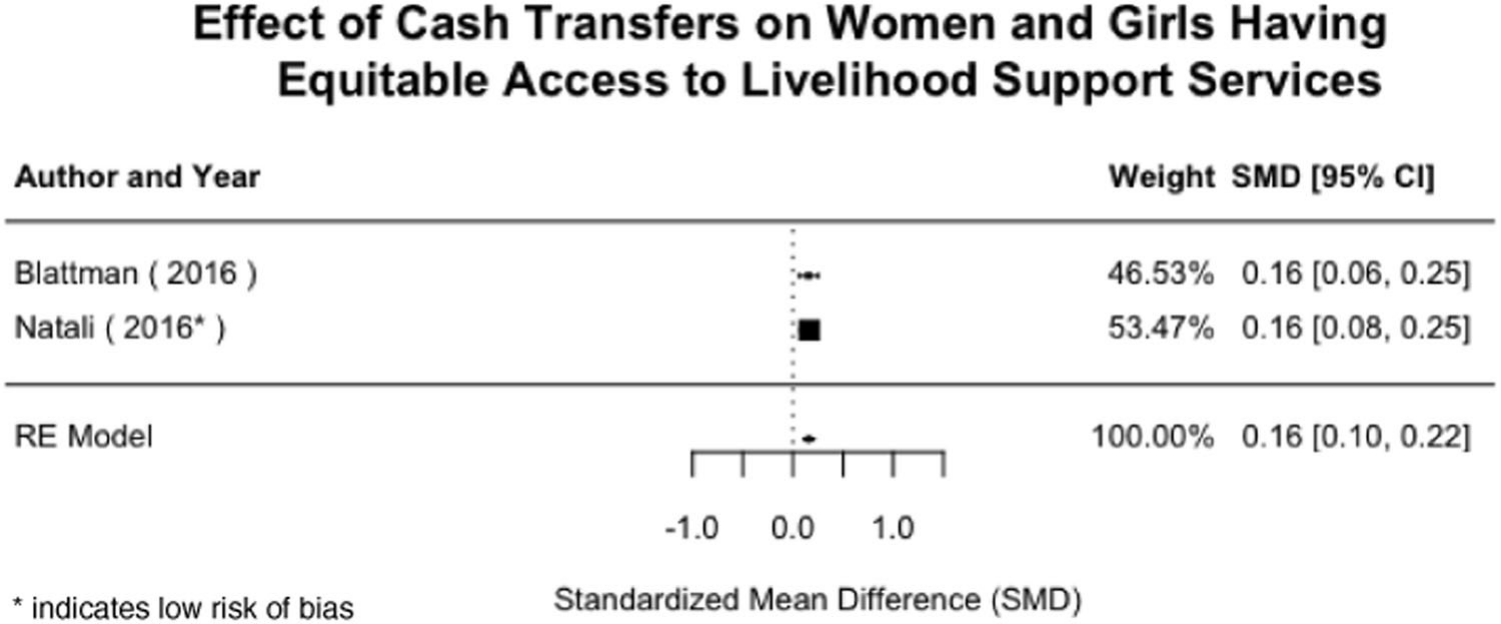
CTAB3. Forest plot showing the observed outcomes and the estimate of the random‐effects model

Only two studies reported the impact of cash transfers on equitable access to livelihood support services, thus we included *k *=* *2 studies in the analysis. We assessed one of the studies as low risk of bias and the other as some concerns. The estimated average outcome based on the random‐effects model was $\hat{\mu }=0.16$ (95% CI: 0.10to0.22). This positive effect differed significantly from zero (z=4.96, p<0.01). A forest plot showing the observed outcomes and the estimate based on the random‐effects model is shown in Figure 38 (CTAB3). Given the small number of studies, this result should be interpreted with caution. According to the Q‐test, there was no significant amount of heterogeneity in the true outcomes (Q(1)=0.00, p=0.94, ${\hat{\tau }}^{2}=0.00$, $I^{2}=0.00$%). With only two studies, moderator analyses were not possible and tests of publication bias are not valid.

*Effects of cash transfers on more women engaged in other micro, small and medium‐sized enterprises*

Only two studies reported the impact of cash transfers on more women engaged in other micro, small, and medium‐sized enterprises. These studies were linked, both examining the impact of WINGS in Uganda. Both studies reported large positive and significant impacts after both 6 months of exposure to the intervention g=0.91 (95% CI:0.71 to 0.92) and 16 months of exposure to the intervention g=0.81(95%CI:0.82to1.01). We assessed one study as having low risk of bias and the other as some concerns.

*Effects of cash transfers on initiatives that help women access decent work (formal and informal employment)*

**Figure 39:**
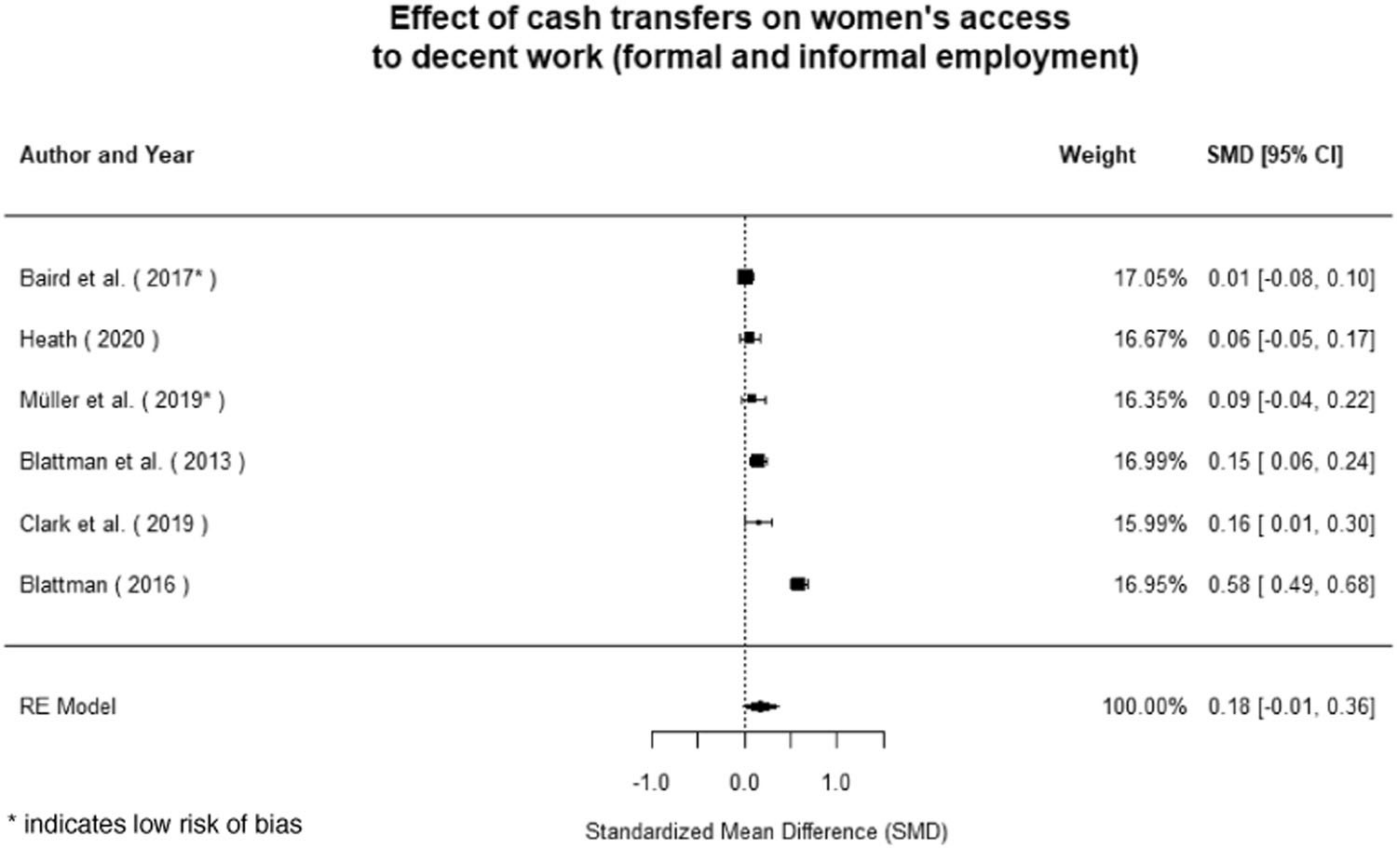
CTAC2: Forest plot showing the observed outcomes and the estimate of the random‐effects model. CI, confidence interval

We included a total of k=6 studies in the analysis. We assessed two studies as low risk of bias, two as some concerns, and two as high risk of bias. The observed outcomes ranged from 0.01 to 0.58. The estimated average outcome based on the random‐effects model was $\hat{\mu }=0.18$ (95% CI: −0.01 to 0.36). Therefore, the average outcome did not differ significantly from zero (z=1.86, p=0.06). A forest plot showing the observed outcomes and the estimate based on the random‐effects model is shown in Figure 39 (CTAC2).

According to the Q‐test, the true outcomes appear to be heterogeneous (Q(5)=93.61, p<0.01, ${\hat{\tau }}^{2}=0.05$, $I^{2}=94.66$%). An examination of the studentised residuals revealed that one study (Blattman et al., 2016) had a value larger than ±2.69 and may be a potential outlier in the context of this model. According to the Cook's distances, one study (Blattman et al., 2016) could be considered to be overly influential. Indeed, sensitivity analyses leaving each study out indicated that removing Blattman et al. (2016) would reduce the overall average effect ($\hat{\mu }$=0.08 (95% CI: 0.03 to 0.15), but the effect would still be positive and would become statistically significant (*z *=* *2.86, *p *=* *0.004).

Effect from studies with high risk of bias were not different than effects from studies with some concerns or low risk of bias ($\hat{B}=-0.03,p=0.88$ [95% CI: −0.43 to 0.37]). There was also no moderation by exposure to intervention ($\hat{B}=-0.01,p=0.41$ [95% CI: −0.03 to 0.01]) nor by evaluation period ($\hat{B}=-0.002,p=0.71$ [95% CI: −0.01 to 0.01]). Publication year was also not a significant source of variation among effects ($\hat{B}=-0.03,p=0.49$ [95% CI: −0.10 to 0.05]). We were not able to test for moderation by study design (all RCTs), post‐intervention versus change from baseline, or whether the model was adjusted for covariates (all were adjusted). In addition, only one effect was conditional, so we were unable to test for differences between conditional and unconditional transfers.

*Effects of cash transfers on improved capacity of women entrepreneurs*

Blattman and colleagues' (2013) experimental study in Uganda was the only study evaluating the impact of cash transfers on improved capacity of women entrepreneurs. There was a large, and statistically significant, effect (*g *=* *0.40, [95% CI:0.32 to 0.49]), but we assessed the study as having high risk of bias.

*Effects of cash transfers on women have more and better control over their bodies and sexual health*

**Figure 40:**
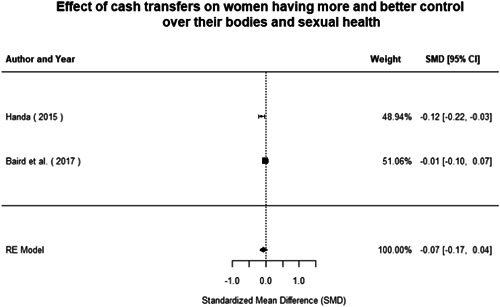
CTBA2: Forest plot showing the observed outcomes and the estimate of the random‐effects model. CI, confidence interval

Only two studies reported the impact of cash transfers on improved life skills for women, thus we included *k *=* *2 studies in the analysis. We assessed one study as having low risk of bias (Sarah et al., 2017) and the other as some concerns related to risk of bias (Handa et al., 2015). The estimated average outcome based on the random‐effects model was $\hat{\mu }=-0.07$ (95% CI: −0.17to0.04). Therefore, the average outcome did not differ significantly from zero (z=−1.22, p=0.22). A forest plot showing the observed outcomes and the estimate based on the random‐effects model is shown in Figure 40 (CTBA2). Given the small number of studies, this result should be interpreted with caution. According to the Q‐test, there was no significant amount of heterogeneity in the true outcomes (Q(1)=2.90, p=0.09, ${\hat{\tau }}^{2}=0.004$, $I^{2}=65.56$%). With only two studies, moderator analyses were not possible and tests of publication bias are not valid.

*Effects of cash transfers on women have increased freedom of movement and association*

**Figure 41:**
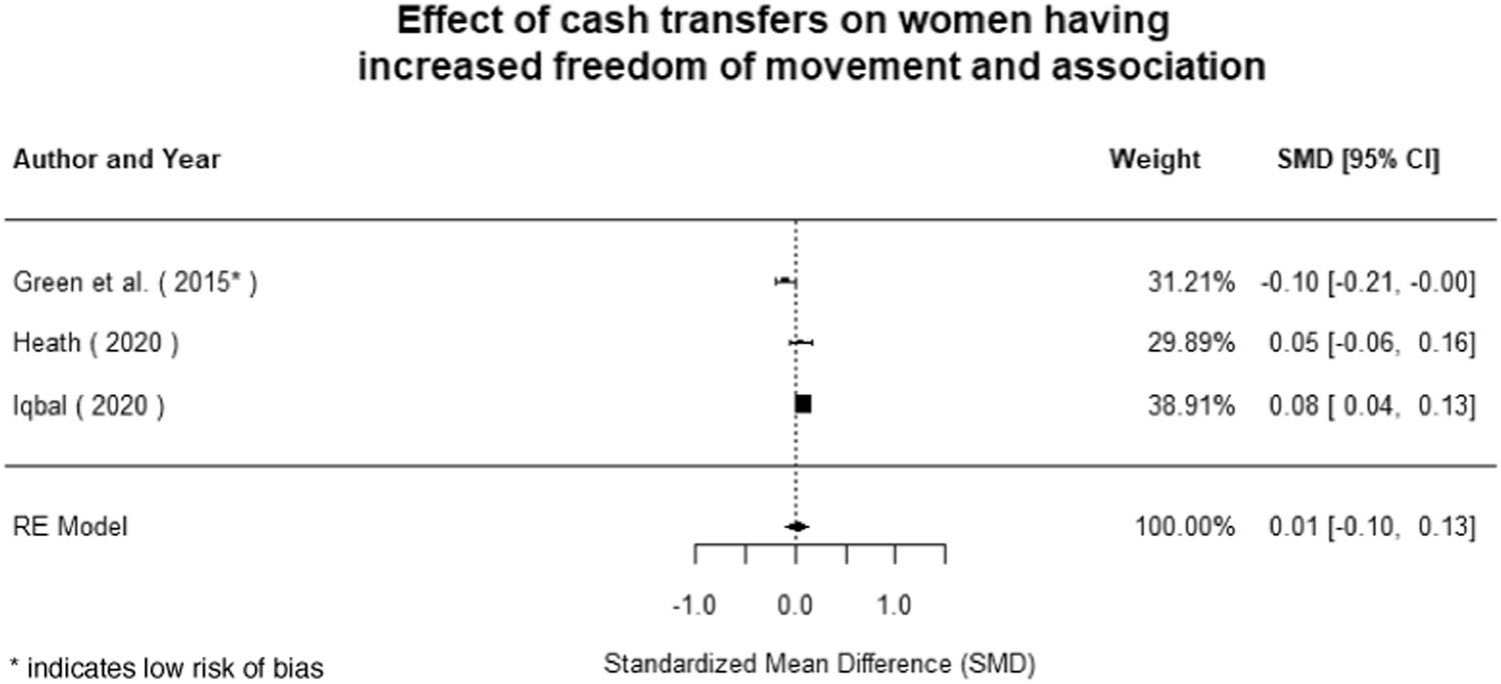
CTBA3: Forest plot showing the observed outcomes and the estimate of the random‐effects model. CI, confidence interval

We included a total of k=3 studies in the analysis. We assessed one study as low risk of bias, two as some concerns, and none as high risk of bias. The observed outcomes ranged from −0.10 to 0.08. The estimated average outcome based on the random‐effects model was $\hat{\mu }=0.01$ (95% CI: −0.10 to 0.13). Therefore, the average outcome did not differ significantly from zero (z=0.25, p=0.80). A forest plot showing the observed outcomes and the estimate based on the random‐effects model is shown in Figure 41 (CTBA3).

According to the Q‐test, the true outcomes appear to be heterogeneous (Q(2)=11.02, p<0.01, ${\hat{\tau }}^{2}=0.01$, $I^{2}=81.85$%). An examination of the studentised residuals revealed that one study (Green et al., 2015) had a value larger than ±2.39 and may be a potential outlier in the context of this model. Indeed, sensitivity analyses leaving each study out indicated that removing Green and colleagues (2015) would increase the overall average effect ($\hat{\mu }=0.08$ [95% CI: 0.04 to 0.12]), and the effect would still be positive, but would be significant (*z *=* *3.72, *p *<* *0.001). According to the Cook's distances, none of the studies could be considered to be overly influential. With only three studies, moderator analyses were not appropriate.

*Effects of cash transfers on effect of cash transfers on reduced percentage of women agreeing with certain reasons that justify violence against women and girls*

**Figure 42:**
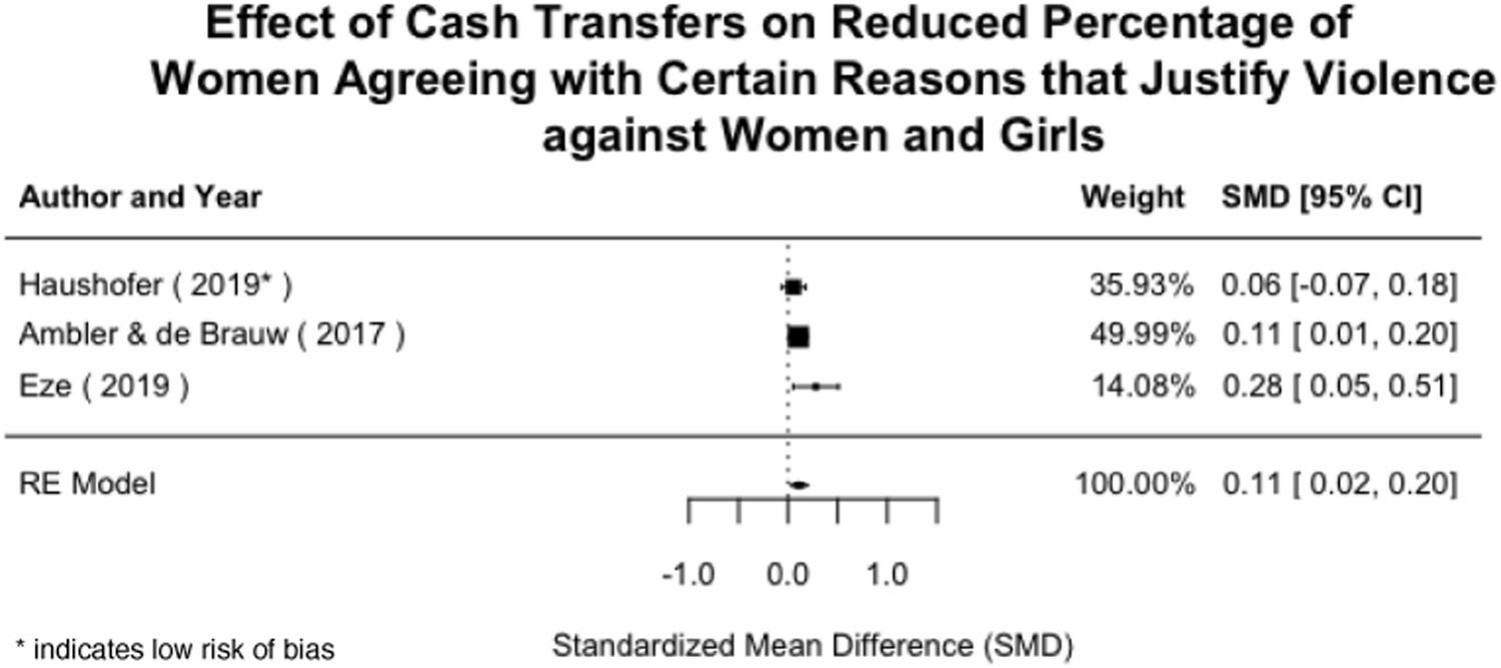
CTBA6: Forest plot showing the observed outcomes and the estimate of the random‐effects model. CI, confidence interval

We included a total of k=3 studies in the analysis. We assessed one study as low risk of bias, two as some concerns, and none as high risk of bias. The observed outcomes ranged from 0.06 to 0.28, with all estimates being positive. The estimated average outcome based on the random‐effects model was $\hat{\mu }=0.11$ (95% CI: 0.02 to 0.20). Therefore, the average outcome differed significantly from zero (z=2.38, p=0.02). A forest plot showing the observed outcomes and the estimate based on the random‐effects model is shown in Figure 42 (CTBA6).

According to the Q‐test, there was no significant amount of heterogeneity in the true outcomes (Q(2)=2.93, p=0.23, ${\hat{\tau }}^{2}=0.00$, $I^{2}=31.64$%). An examination of the studentised residuals revealed that none of the studies had a value larger than ±2.39 and hence there was no indication of outliers in the context of this model. According to the Cook's distances, none of the studies could be considered to be overly influential. With only three studies, moderator analyses were not appropriate.

*Effects of cash transfers on improved life skills for women*

**Figure 43:**
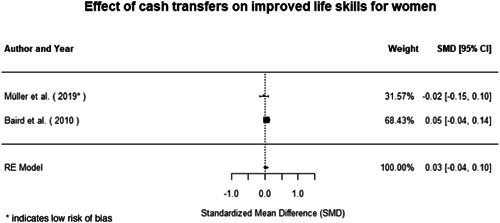
CTBA7: Forest plot showing the observed outcomes and the estimate of the random‐effects model. CI, confidence interval

Only two studies reported the impact of cash transfers on improved life skills for women, thus we included *k *=* *2 studies in the analysis. One study was assessed as having low risk of bias, the other as having high risk of bias. The estimated average outcome based on the random‐effects model was $\hat{\mu }=0.03$ (95% CI: −0.04to0.10). Therefore, the average outcome did not differ significantly from zero (z=0.74, p=0.46). A forest plot showing the observed outcomes and the estimate based on the random‐effects model is shown in Figure 43 (CTBA7). Given the small number of studies, this result should be interpreted with caution. According to the Q‐test, there was no significant amount of heterogeneity in the true outcomes (Q(1)=0.89, p=0.46, ${\hat{\tau }}^{2}=0.00$, $I^{2}=0.00$%). With only two studies, moderator analyses were not possible and tests of publication bias are not valid.

*Effects of cash transfers on reduced support for, or instances of, child or forced marriage*

**Figure 44:**
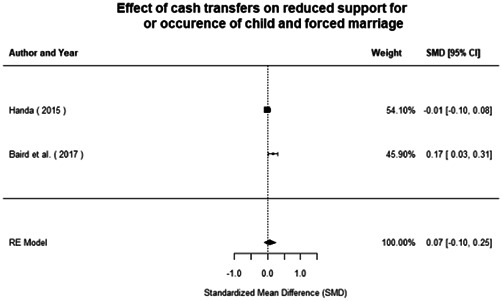
CTBA8: Forest plot showing the observed outcomes and the estimate of the random‐effects model. CI, confidence interval

Only two studies reported the impact of cash transfers on reduced support for, or instances of, child or forced marriage, thus we included *k *=* *2 studies in the analysis. We assessed one study as having low risk of bias (Sarah et al., 2017) and the other as some concerns (Handa et al., 2015). The estimated average outcome based on the random‐effects model was $\hat{\mu }=0.07$ (95% CI: −0.10to0.25). Therefore, the average outcome did not differ significantly from zero (z=0.80, p=0.42). A forest plot showing the observed outcomes and the estimate based on the random‐effects model is shown in Figure 44 (CTBA8). Given the small number of studies, this result should be interpreted with caution. According to the Q‐test, there was no significant amount of heterogeneity in the true outcomes (Q(1)=4.59, p=0.03, ${\hat{\tau }}^{2}=0.01$, $I^{2}=78.22$%). With only two studies, moderator analyses were not possible and tests of publication bias are not valid.

*Effects of cash transfers on increased participation of women in decision making at the household or community level*

**Figure 45:**
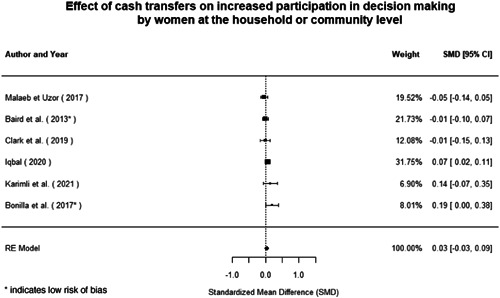
CTBB1: Forest plot showing the observed outcomes and the estimate of the random‐effects model. CI, confidence interval

We included a total of k=6 studies in the analysis. We assessed two studies as low risk of bias, one as some concerns, and three as high risk of bias. The observed outcomes ranged from −0.05 to 0.19. The estimated average outcome based on the random‐effects model was $\hat{\mu }=0.03$ (95% CI: −0.03 to 0.09). Therefore, the average outcome did not differ significantly from zero (z=1.11, p=0.27). A forest plot showing the observed outcomes and the estimate based on the random‐effects model is shown in Figure 45 (CTBB1).

The Q‐test for heterogeneity was not significant, but some heterogeneity may still be present in the true outcomes (Q(5)=9.79, p=0.08, ${\hat{\tau }}^{2}=0.00$, $I^{2}=48.92$%). An examination of the studentised residuals revealed that none of the studies had a value larger than ±2.77 and hence there was no indication of outliers in the context of this model. According to the Cook's distances, none of the studies could be considered to be overly influential. If the high risk of bias studies were removed, the average effect increases slightly and is still significantly different from zero.

For decision making outcomes, effects from conditional cash transfers were smaller than effects from unconditional cash transfers by 0.10 standard deviation units, a small but significant difference ($\hat{B}=-0.10,p=0.01$ [95% CI: −0.18 to −0.03]). Effect from studies with high risk of bias were not different than effect form studies with some concerns or low risk of bias ($\hat{B}=-0.06,p=0.35$ [95% CI: −0.18 to 0.06]). There was also no moderation by exposure to intervention ($\hat{B}=0.001,p=0.44$ [95% CI: −0.001 to 0.004]). Publication year was also not a significant source of variation among effects ($\hat{B}=0.01,p=0.20$ [95% CI: −0.01 to 0.03]). Effects did not differ by whether the study was examining post intervention changes versus changes from baseline ($\hat{B}=0.07,p=0.30$ [95% CI: −0.06 to 0.20]) nor by whether or not the model was adjusted for covariates ($\hat{B}=-0.06,p=0.28$ [95% CI: −0.16 to 0.04]). We were not able to test for moderation by study design (only one study used a QED), nor by evaluation period (all studies examined effects immediately after exposure to intervention).

*Effects of cash transfers on increased on women participate more in their community*

**Figure 46:**
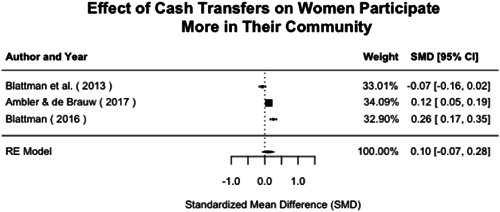
CTBB2: Forest plot showing the observed outcomes and the estimate of the random‐effects model. CI, confidence interval

We included a total of k=3 studies in the analysis. None of the studies were appraised as low risk of bias, and so these findings should be applied cautiously. We assessed two studies as having some concerns of risk of bias and one as high risk of bias. The observed outcomes ranged from −0.07 to 0.26. The estimated average outcome based on the random‐effects model was $\hat{\mu }=0.10$ (95% CI: −0.07 to 0.28). Therefore, the average outcome did not differ significantly from zero (z=1.17, p=0.24). A forest plot showing the observed outcomes and the estimate based on the random‐effects model is shown in Figure 46 (CTBB2).

According to the Q‐test, the true outcomes appear to be heterogeneous (Q(2)=25.55, p<0.01, ${\hat{\tau }}^{2}=0.02$, $I^{2}=92.17$%). An examination of the studentised residuals revealed that none of the studies had a value larger than ±2.39 and hence there was no indication of outliers in the context of this model. According to the Cook's distances, none of the studies could be considered to be overly influential. With only three studies, moderator analyses were not appropriate.

*Effects of cash transfers on the rights, safety and security of women, men, girls and boys being protected during relief, recovery and reconstruction*

Brooks and colleagues' (2020) experimental study in Kenya was the only study evaluating the impact of cash transfers on the rights, safety and security of women, men, girls and boys being protected during relief, recovery and reconstruction. Their report included two effects that fell into this outcome category (Personal protective equipment spending and protective measures). The effects were medium size and positive for both PPE spending (*g *=* *0.29, 95% CI [0.04, 0.53]) and protective measures (*g *=* *0.36, 95% CI [0.12 to 0.61]. We assessed the study as having high risk of bias.

*Effects of cash transfers on increased representation of women in local and subnational civil and political processes, including during peacebuilding and post conflict restoration*

We included a total of three studies in Pakistan reporting on the impact of cash transfers on the representation of women in political processes (Ambler & De Brauw, 2017; Iqbal et al., 2020). All three were studying the BISP after different periods of exposure to the intervention (20, 72, and 90 months). We assessed all three studies as having some concerns of risk of bias. The observed outcomes ranged from g=0.07 (95% CI:0.02 to 0.11]) to g=0.22(95%CI:0.13to0.30), with the largest impact reported after 90 months of exposure to the intervention. All three reports were appraised as having some concerns related to risk of bias.

*Effects of cash transfers on communities' attitudes towards women/marginalised groups*

Blattman et al.'s (2016) experimental study in Uganda was the only study evaluating the impact of cash transfers on communities' attitudes towards women/marginalised groups. Without group training, there was a small, negative and statistically significant effect (*g *=* *−0.11, 95% CI [−0.20, −0.01]), and with group training there was a very small, positive but not statistically significant effect (*g *=* *0.02, [95% CI: −0.08 to 0.11]). We assessed the study as having some risk of bias concerns.

*Effects of cash transfers on improved attitudes and increased support for women's social, economic and human rights by men, family member, household members and the community*

**Figure 47:**
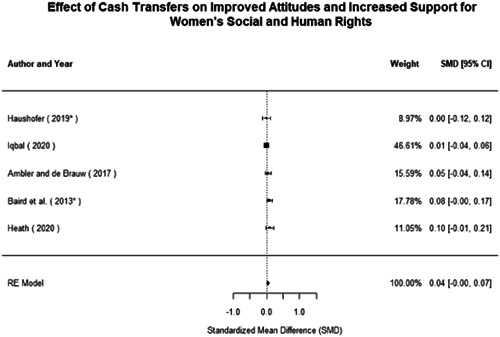
CTCB4: Forest plot showing the observed outcomes and the estimate of the random‐effects model. CI, confidence interval

We included a total of k=5 studies that examined the impact of cash transfer on improved attitudes towards women. We assessed two studies as low risk of bias, three as some concerns, and none as high risk of bias. The observed outcomes ranged from 0.00 to 0.10. The estimated average outcome based on the random‐effects model was $\hat{\mu }=0.04$ (95% CI: −0.00 to 0.07). Therefore, the average outcome did not differ significantly from zero (z=1.88, p=0.06). A forest plot showing the observed outcomes and the estimate based on the random‐effects model is shown in Figure 47 (CTCB4).

According to the Q‐test, there was no significant amount of heterogeneity in the true outcomes (Q(4)=4.27, p=0.37, ${\hat{\tau }}^{2}=0.00$, $I^{2}=6.43$%). An examination of the studentised residuals revealed that none of the studies had a value larger than ±2.58 and hence there was no indication of outliers in the context of this model. According to the Cook's distances, one study (Iqbal et al., 2020) could be considered to be overly influential. Indeed, sensitivity analyses leaving each study out indicated that removing Iqbal et al. (2020) would increase the overall average effect ($\hat{\mu }\;=\;$0.06, (95% CI: 0.01 to 0.11)), and the effect would become statistically significant (*z *=* *2.47, *p *=* *0.01).

For decision making outcomes, effects from conditional cash transfers were no different than effects from unconditional cash transfers ($\hat{B}=0.05,p=0.21$ [95% CI: −0.03 to −.13]). There was also no moderation by exposure to intervention ($\hat{B}=-0.001,p=0.14$ [95% CI: −0.002 to 0.0003]). Effects from experimental studies were no different than effects from quasi‐experimental studies ($\hat{B}=-0.05,p=0.16$ [95% CI: −0.13 to 0.02]). Publication year was also not a significant source of variation among effects ($\hat{B}=-0.01,p=0.19$ [95% CI: −0.02 to 0.004]). Effects did not differ by whether the study was examining post intervention changes versus changes from baseline ($\hat{B}=-0.05,p=0.16$ [95% CI: −0.13 to 0.02]) nor by whether or not the model was adjusted for covariates ($\hat{B}=0.07,p=0.052$ [95% CI: −0.001 to 0.14]). Finally, we found no moderation by scores on neither the GII ($\hat{B}=1.26,p=0.06$ [95% CI: −0.08 to 2.60]), nor the FSI ($\hat{B}=-0.01,p=0.08$ [95% CI: −0.01 to 0.001]). We were not able to test for moderation by study design (only one study used a QED), and there were no high risk of bias studies in this outcome group, so we did not test moderation by high risk of bias. We also could not test by evaluation period because all studies evaluated effects immediately after the end of the intervention.

*Effects of cash transfers on more women engaged in other micro, small and medium‐sized enterprises*

**Figure 48:**
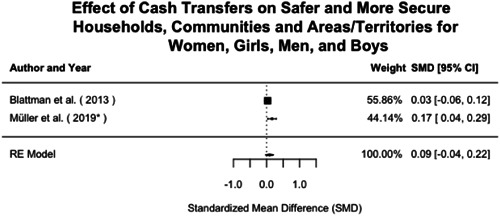
CTCB6: Forest plot showing the observed outcomes and the estimate of the random‐effects model. CI, confidence interval

Only two studies reported the impact of cash transfers on more women engaged in other micro, small, and medium‐sized enterprises, thus we included *k *=* *2 studies in the analysis. We assessed one of the studies as low risk of bias and the other as high risk of bias. The estimated average outcome based on the random‐effects model was $\hat{\mu }=0.09$ (95% CI: −0.04to0.22). This effect was not significantly from zero (z=1.38, =<0.17). A forest plot showing the observed outcomes and the estimate based on the random‐effects model is shown in Figure 48 (CTCB6). Given the small number of studies, this result should be interpreted with caution. According to the Q‐test, there was no significant amount of heterogeneity in the true outcomes (Q(1)=2.78, p=0.09, ${\hat{\tau }}^{2}=0.01$, $I^{2}=64.10$%). With only two studies, moderator analyses were not possible and tests of publication bias are not valid.

*Effects of cash transfers on frequency and distribution of types of violence by an intimate partner*

**Figure 49:**
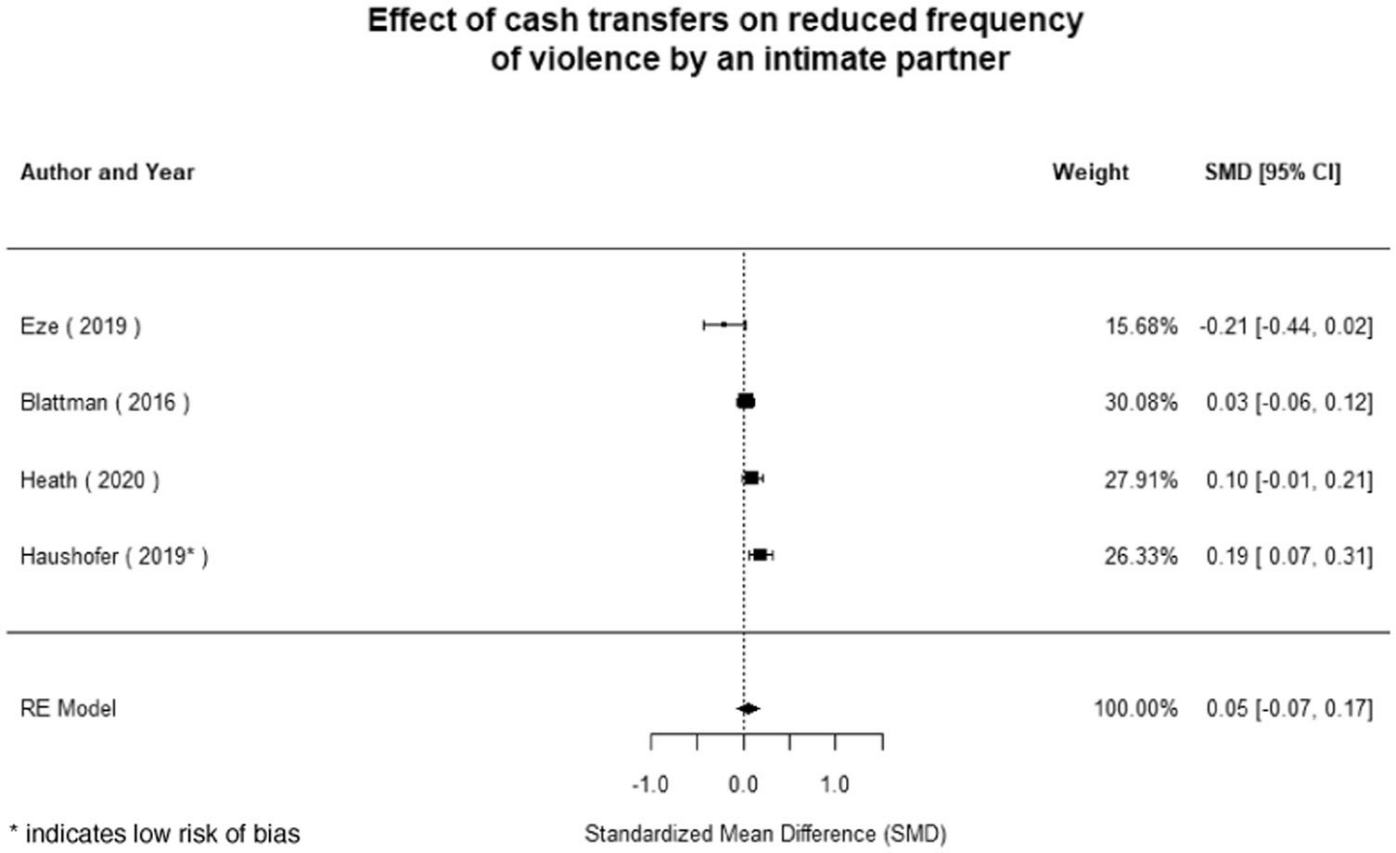
CTCB7: Forest plot showing the observed outcomes and the estimate of the random‐effects model. CI, confidence interval

We included a total of k=4 studies in the analysis. We assessed one study as low risk of bias, three as some concerns, and none as high risk of bias. The observed outcomes ranged from −0.21 to 0.19. The estimated average outcome based on the random‐effects model was $\hat{\mu }=0.05$ (95% CI: −0.07 to 0.17). Therefore, the average outcome did not differ significantly from zero (z=0.88, p=0.38). A forest plot showing the observed outcomes and the estimate based on the random‐effects mode l is shown in Figure 49 (CTCB7).

According to the Q‐test, the true outcomes appear to be heterogeneous (Q(3)=10.52, p=0.01, ${\hat{\tau }}^{2}=0.01$, $I^{2}=71.49$%). An examination of the studentised residuals revealed that none of the studies had a value larger than ±2.58 and hence there was no indication of outliers in the context of this model. According to the Cook's distances, none of the studies could be considered to be overly influential.

There was also no moderation by exposure to intervention ($\hat{B}=0.003,p=0.79$ [95% CI: −0.02 to 0.03]), and publication year was also not a significant source of variation among effects ($\hat{B}=0.01,p=0.86$ [95% CI: −0.11 to 0.13]). Effects did not differ by whether or not the model was adjusted for covariates ($\hat{B}=0.05,p=0.79$ [95% CI: −0.32 to 0.42]). We were not able to test for moderation by study design (only one study used a QED), and there were no high risk of bias studies in this outcome group, so we did not test moderation by high risk of bias. We also could not test by evaluation period because only one study did not evaluate effects immediately after the end of the intervention, nor for moderation by whether the study was examining post‐intervention changes or changes from baseline. Finally, we could not examine differences between conditional and unconditional transfers because only one used a conditional transfer.

*Effects of cash transfers on improved quality of relationships between women and their household and community members*

**Figure 50:**
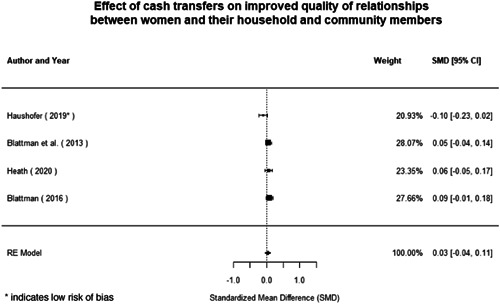
CTCB8: Forest plot showing the observed outcomes and the estimate of the random‐effects model. CI, confidence interval

We included a total of k=4 studies in the analysis. We assessed one study as low risk of bias, two as some concerns, and one as high risk of bias. The observed outcomes ranged from −0.10 to 0.09. The estimated average outcome based on the random‐effects model was $\hat{\mu }=0.03$ (95% CI: −0.04 to 0.11). Therefore, the average outcome did not differ significantly from zero (z=0.81, p=0.42). A forest plot showing the observed outcomes and the estimate based on the random‐effects model is shown in Figure 50 (CTCB8).

The Q‐test for heterogeneity was not significant, but some heterogeneity may still be present in the true outcomes (Q(3)=6.45, p=0.09, ${\hat{\tau }}^{2}=0.00$, $I^{2}=53.50$%). An examination of the tudentized residuals revealed that one study (2) had a value larger than ±2.58 and may be a potential outlier in the context of this model. According to the Cook's distances, none of the studies could be considered to be overly influential.

Most moderators could not be tested because of a lack of variation among the studies, but of the two that could be tested, effects did not differ by publication year ($\hat{B}=-0.001,p=0.89$ [95% CI: −0.02 to 0.01]), nor by length of exposure to intervention ($\hat{B}=-0.01,p=0.53$ [95% CI: −0.04 to 0.02]).

*Effects of cash transfers on women's empowerment index*

**Figure 51:**
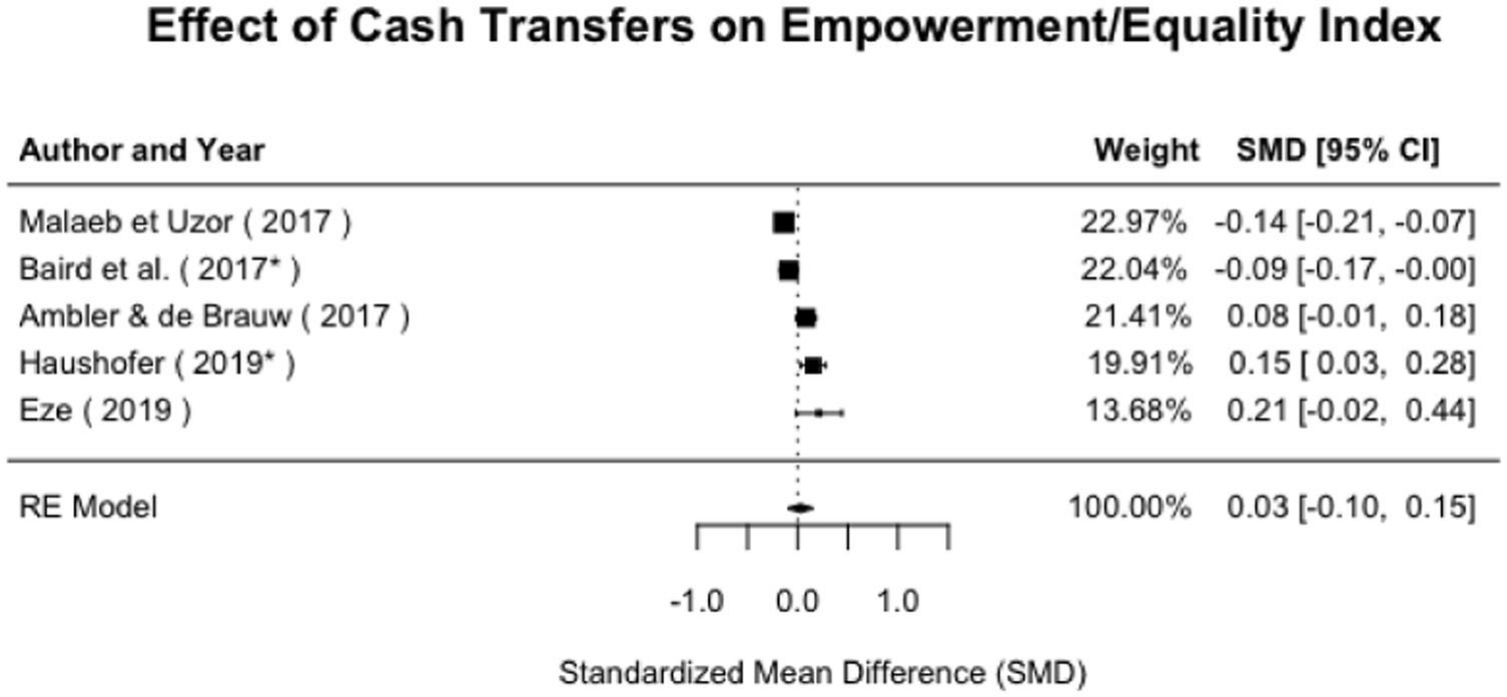
CTCC1: Forest plot showing the observed outcomes and the estimate of the random‐effects model. CI, confidence interval

We included a total of k=5 studies in the analysis. We assessed two of the studies as low risk of bias, two as some concerns and one as high risk of bias. The observed outcomes ranged from −0.14 to 0.21. The estimated average outcome based on the random‐effects model was $\hat{\mu }=0.03$ (95% CI: −0.10 to 0.15). Therefore, the average outcome did not differ significantly from zero (z=0.41, p=0.68). A forest plot showing the observed outcomes and the estimate based on the random‐effects model is shown in Figure 51 (CTCC1).

According to the Q‐test, the true outcomes appear to be heterogeneous (Q(4)=29.32, p<0.01, ${\hat{\tau }}^{2}=0.02$, $I^{2}=86.36$%). An examination of the andomized residuals revealed that none of the studies had a value larger than ±2.58 and hence there was no indication of outliers in the context of this model. According to the Cook's distances, none of the studies could be considered to be overly influential.

Effects from experimental studies were not different than effects from quasi‐experimental studies ($\hat{B}=0.16,p=0.21$ [95% CI: −0.09 to 0.42]). They were also no different between studies using conditional versus unconditional cash transfers ($\hat{B}=0.004,p=0.98$ [95% CI: −0.30 to 0.31]). However, effects did differ by length of exposure to intervention ($\hat{B}=-0.07,p=0.045$ [95% CI: −0.01 to −0.001]) such that each additional month decreased the effect by 0.07 standard deviation units. Finally, effect from studies whose models were adjusted for covariates were larger than those from studies using unadjusted models by 0.23 standard deviation units ($\hat{B}=-0.07,p=-0.23$ [95% CI: −0.43 to −0.02]).

##### 5.3.2.11 Qualitative findings

We conducted a thematic synthesis on 25 linked qualitative studies to the included cash transfer interventions. As indicated above, this thematic synthesis aims to identify themes related to the interplay of intervention design, intervention implementation, target population and contextual variables with intervention outcomes and effects. In total, we identified 18 descriptive themes, which we configured into five analytical themes (Supporting Information Appendix SN). These five analytical themes present the synthesis results and are discussed in more detail below.

*Theme 1: Gender norms, culture and religious beliefs can preserve men's dominance in the household which affects programme uptake and participation and may result in undesirable outcomes in poor households*.

Cash transfer programmes can support joint decision‐making processes within beneficiary households and enhance the autonomy of women when making financial decisions. However, religion and traditional beliefs regarding gender roles were often used to justify men's superior status which leads to the perpetuation of control and inequality within poorer households. Cash transfers to married women may lead to men's dissatisfaction with women earning money and could cause household conflicts. In some communities, women are not allowed to overshadow their husbands as the provider (ElDidi et al., 2018). The identified themes suggest that these cultural nuances of household power dynamics can undermine positive changes for women's empowerment outcomes.

Gender norms and cultural and religious beliefs thus featured heavily in cash transfer programmes and their uptake. For instance, qualitative findings from the WINGS programme in Uganda suggested that the role of supportive partners enhanced the well‐being of target women and other benefits from the programme (Blattman et al., 2013). Notwithstanding household heterogeneity, WINGS’ women beneficiaries reported modest improvements in decision‐making power within the household, even in the face of rigid traditional beliefs (Bonilla et al., 2017). Furthermore, an improvement in autonomy may mean that women felt more confident to make decisions without their partner's approval.

Household relationships are a more complex, yet influential, factor in how cash transfers administered were perceived and andomiz. Gendered norms were seen to influence the control and use of cash within the households, often in favour of men. The participants’ views were mixed, as secondary perspectives suggested that disagreements on how to use the money were often divisive and conflict‐inducing in the Zambian child grant scheme (Bonilla et al., 2017). In Egypt, one Takaful and Karama beneficiary expressed improved relationship quality with her partner: ‘We are able to afford a shared meal of meat more often. We became closer to each other’ (ElDidi et al., 2018). In Ethiopia, women participating in the PSNP felt more empowered but were similarly restricted by existing household duties and mandatory public works participation (Gelagay & Lecoutere, 2019).

Smaller cash benefits and those designated for women may only exacerbate tensions within beneficiary households. In the Takaful and Karama programme, a woman beneficiary, when asked if any joint decisions were taken between her and her husband, remarked: ‘None. The money is with my husband to barely cover our food’. The woman felt no improvement in her decision‐making contribution within the household. Another noted that relations between her and her partner had deteriorated slightly adding that: ‘Yes, [the transfers] can make some problems because a man can get mad… Some men can get annoyed because the money is coming to the woman, so he would think she'll spend it on herself’. Moreover, men's dominance in traditional societies seemed to stifle programme impact when it came to household relationships and empowerment.

*Theme 2: Targeting presented a key tool to generate benefits for the most vulnerable households and individuals*.

Cash transfer programmes typically targeted the most vulnerable households and women who struggle to meet their basic needs and those who do not have access to credit or capital to start businesses. Targeting procedures aimed to andomiz beneficiary selection errors, maximise trust in administrative processes and focus on low‐income groups. Accurate targeting requires consideration of social and economic contexts so that it works to empower the historically andomizedi and improve their well‐being. Women felt more empowered when targeted as beneficiaries than if men were. As one programme officer of the Takaful and Karama programme observed: ‘We cannot conclude whether these effects would have been different if the transfers were targeted to men, but it seems unlikely that doing so would have improved women's control over household decisions' (ElDidi et al., 2018). There was additional support by both men and women of the choice to prioritise women (El‐Enbaby et al., 2019).

An inclusive targeting approach is important to help policymakers reach those in socioeconomic need and families as a unit rather than individuals (Berlie, 2018; ElDidi et al., 2018). Participants in the Takaful and Karama Programme reflected that for severely poor households, the cash injection was largely spent on basic needs such as food, clothing and medical expenses (ElDidi et al., 2018). Most of these beneficiaries had high levels of financial debt from an array of sources. Rural Egyptian communities with rigid social norms were difficult to access and to target women, who were blocked from participating in work (El‐Enbaby et al., 2019).

Beneficiaries of the WINGS programme in Uganda suggested that targeted participants should be of a determined eligible age to enhance the way cash is used in the household. Indeed, some perceptions suggested that younger cohorts of the beneficiary pool were undeserving of the cash because they had an income to rely on Baird et al. (2013). There was evidence on meeting of conditional requirements to qualify for cash injections; participants in the Takaful and Karama programme suggested that it was a justified strategy to ensure that the needy are andomized and that cash is not misused (Zaki, 2018). Poorer households were also often indifferent on meeting the requirement of minimum school attendance and clinic visit targets for pre‐schoolers which was stipulated by the Takaful and Karama programme (El‐Enbaby et al., 2019).

Qualitative results from the PSNP urged that the graduation model for beneficiaries was perceived to prematurely drop qualifying beneficiaries (Gelagay & Lecoutere, 2019). Furthermore, strict criteria on graduation requirements might be a problem. As one beneficiary adds: ‘At present, I graduated from safety nets programme but I could not feed my family for 12 months from my own production’ (Berlie, 2018). Some of the key issues alluded to the absence of a full family targeting strategy as well as low asset formation, as cash was largely used for basic needs. Due to these two factors, the graduation eligibility criteria in some instances excluded qualifying beneficiaries prematurely (Berlie, 2018).

*Theme 3: Mentorship and support from either programme mentors or family members encourages beneficiaries to engage in programme activities*.

Mentorship, training and support from family members encourages adherence and compliance to programme requirements, especially in conditional cash transfer programmes. Mentorships encourage beneficiaries to meet their financial obligations and for parents to enrol their children in schools, for instance. Family coaching is perceived as useful for the achievement of financial autonomy and improved marital relations for women. Stringent requirements for beneficiary compliance can stifle programme implementation. To maintain eligibility status, cash transfer programmes targeted at younger women often require parents to adhere to conditions. The feasibility of these conditions requires careful consideration of the extent to which they may curtail positive outcomes.

From the qualitative evidence, an integral part of cash transfer programmes was the role of interactions between participants, programme staff and third‐party support structures. The Rural Entrepreneur Access Project (REAP) in Kenya revealed some crucial insights. For both monitoring and evaluation (MandE) and programme support purposes, BOMA Project mentors supported REAP participants in ensuring programme adherence and coaching for 2 years to enhance school attendance, financial literacy and beneficiaries’ knowledge (BOMA, 2018).

Additional results from Karimli et al. (2020) indicate that the mentoring and coaching component embedded within the Trickle Up cash transfer programme in Burkina Faso made various positive contributions, largely related to resource access and control. Evidence from the Takaful and Karama scheme suggested that women preferred being the main beneficiaries of cash programmes with one female beneficiary expressing: ‘It's good this way (that the transfers are going to women) […] the state's caring for me. In other words, it's given me dignity’ (El‐Enbaby et al., 2019).

*Theme 4: Awareness‐raising initiatives support programme coverage and sensitise target beneficiaries to programme objectives to encourage participation*.

Cash transfer programmes design factors included a sufficient range of awareness‐raising strategies and prior support to understand the objectives and properties of the programme. Sensitising target populations is important to consider as a design factor that can support the andomized of desired impacts. This is convenient in trying to influence perceptions of prospective beneficiaries, so that they fully understand issues of eligibility, conditionality and full compliance. Prior knowledge of the exact size of cash transfers is a relevant factor for programme design. When cash transfers and programme coverage were perceived to be insufficient, beneficiaries were less satisfied. The role of inflation in diminishing purchasing power and limiting beneficiary options for cash use presents a further design consideration.

In the ZCTP in Malawi, slow uptake and lack of awareness in communities of interest motivated the implementation team to design and execute a formal andomizediz process. Participation rates improved and were reported by programme staff as successful in helping prospective participants understand and accept the intervention. However, the additional time and resources spent on this was costly to the overall programme (Baird et al., 2013). For the ZCTP, the investigation team andomiz the village structure to ensure that willing participants were successfully identified. Another key barrier to implementation was that potential participants were seemingly only aware of the study but not the specification that a cash incentive might be offered to chosen beneficiaries.

In the case of Trickle Up in Burkina Faso, Karimli et al. (2020) found that because prior andomizediz was not feasible due to lack of resources, the implementation team resolved to limit their intended programme reach. The evidence suggested that there might be value in raising awareness and educating target populations before full implementation. Takaful and Karama continued to struggle with raising awareness as a key member of the government implementation team expressed: ‘No booklets or brochures have been given to the applicants’, apart from printed posters, which included the eligibility criteria for prospective beneficiaries (Zaki, 2018).

*Theme 5: Adverse programme operations, untimely delivery of cash and negative perceptions of administrative challenges are bottlenecks to the successful implementation of cash transfer programmes*.

The timely and predictable delivery of cash and the low cost of access to beneficiaries are key design contributors to enhance cash transfer programme success. Obstacles to access the transfer and perceptions of administrative difficulties may temper programme satisfaction. Adherence and participation rely heavily on beneficiary knowledge of and confidence in cash transfer programmes.

Zambia's cash transfer scheme was logistically satisfactory for beneficiaries. However, most study participants suggested a more intimate, door‐to‐door delivery model for payments (Seidenfeld et al., 2013). In addition to this, beneficiaries felt that the cash amount received was insufficient to adequately cover their basic household needs. A Takaful and Karama beneficiary expressed that: ‘What the government gives with the right hand, it takes with the left one’, while another added that: ‘This isn't the sum of money that would let the man of the household sleep’ (Zaki, 2018). Moreover, nearly all surveyed recipients of the cash transfer were satisfied with the timely availability of their benefits and had confidence that their next payments would also be available on time (Handa et al., 2019). Another important factor from the qualitative evidence is related to how beneficiaries perceived the administration and dissemination of cash benefits. Negative perceptions on programme staff were an additional barrier to implementation.

Additional qualitative findings from the Takaful and Karama cash transfer programme revealed that many beneficiaries were unhappy with the selection process; those who did not qualify were reported to often have been erroneously included. Eligibility criteria and ineffective adherence monitoring were reportedly often manipulated by beneficiaries who were able to underreport their earnings without consequence (ElDidi et al., 2018). Furthermore, perceived corruption in the provision of cash benefits and staff mistreatment of beneficiaries were cited as common issues in the Egyptian context. One Takaful and Karama beneficiary complained that: ‘They were pushing us and dealing with the people in a very bad way’ while another shared a different sentiment on staff treatment: ‘…they were good to us and dealt with us in a good way’. Ultimately, hostility from government staff was met with hostility from the beneficiaries (Zaki, 2018). In some cases, cash transfer programmes may not be adequately marketed to intended beneficiaries.

Lack of knowledge of programme operations and systems presents an impediment to uptake and adherence because participants often have little understanding on how to maintain their benefits and involvement in the cash transfer programmes. Beneficiaries in the Takaful and Karama programme expressed scant knowledge of the programme rules and actual value of the benefits while implementing partners confessed that there was no reliable system in place to monitor such failures or limitations (Zaki, 2018). Other recipients of the transfer knew the programme by a different name which curbed their interest in learning the rules and qualification criteria (‘Maash El‐Sisi’ was the name that many participants used). Some of the monitoring failures were captured when one Takaful and Karama informant complained that: ‘Someone called me and asked me a number of questions and then told me that if anyone asked you if a researcher visited you say yes’. To ensure that beneficiaries were compliant, another beneficiary was of the view that: ‘The government has the right to enter the homes of the applicants’, to assess applicant living conditions and confirm eligibility as a monitoring technique (Zaki, 2018).

##### 5.3.2.12 Discussion

Across 20 total studies (*n* = 20), quantitative meta‐analysis findings from cash transfers tested a wide range of outcomes covering domains such as empowerment, well‐being and relationship quality. While several outcomes such as those related to access to assets, credit and income as well as engagement in small, medium and micro enterprises are positive, there are more qualitative insights to discuss the empowerment‐related outcomes. Summarily, there are smaller positive quantitative effects on empowerment‐related outcomes such women's decision‐making power in communities or households and gender equality (albeit statistically significant) outcomes. A theme that emerges quite commonly from the qualitative evidence base (*n* = 25) is the centrality of gender norms and roles when designing programmes. Below are a few possible explanations from the qualitative evidence:

- Social norms, existing gender roles and rigid traditional beliefs can preserve men's dominance regardless of the cash injection in a typical household. While the cash transfer can accrue to women and/or girls within the household, men can control the use of these benefits or even discourage the uptake of these programmes by women and girls within the household.
- The design of cash transfer programmes that consider the harmful role of structural norms in society can aid improved outcomes to empowerment. Cash benefits should not be assumed to directly spill over to addressing structural norms.
- Programmes can be enhanced by adding components such as a couple life, social and livelihood skills and capacity building component that aims to improve household relationships and conflict resolution skills. Additionally, mentoring components can deepen peer‐to‐peer exchanges and collective building to cast the net for learning and interaction wider.
- Cash transfers can advance the financial standing of women in society as asset ownership, access to credit and income improve over time if benefits are used well.
- Women's improved access to livelihood support services can be attributed to their improved well‐being and financial position as they can access key amenities and services that are central to their livelihood.
- There are various points of focus that present opportunities for programme designers to refine cash transfer programmes:
  o More accurate targeting efforts and clear awareness‐raising tactics can boost uptake and the identification of the most relevant target group.
  o Instead of targeting beneficiaries individually, there can be better consideration for family unit targeting to mitigate conflict about benefits.
  o According to the qualitative data, there is evidence that beneficiaries would prefer more cash as a benefit due to inflationary pressures and household size.
  o Programme staff andomized that this cash amount should not replace salaries and wages from work.

##### 5.3.2.13 Summary of findings and discussion

We included 20 studies in 12 countries in MENA, South Asia and Sub‐Saharan Africa that evaluated the effect of cash transfers. We were able to examine effects on the following outcomes: Women have increased access and ownership to assets, credit and income, Initiatives supported that facilitate women to access decent work (formal and informal employment), including people with disabilities, women have increased freedom of movement and association, Increased participation in decision making by women at the household or community level, including during crisis response, women participate more in their community. Our included studies report against all three of the secondary outcomes (Resources, Agency and Achievement) and eight of the nine immediate outcomes for our review. Overall, the GRADE assessments generally indicate a very low to high certainty in this body of evidence.

For 25 linked qualitative studies, five analytical themes emerged that buttress the quantitative findings and give a contextual lens to the outcomes of cash transfer programmes in FCAS. Gender norms were a significant barrier to success for women or individuals receiving cash transfers due to roles and permissions related to the management of money in the household. Targeting specific vulnerable populations supported progress against specific outcomes. However, project management and implementation could hinder the ability of cash transfers to positively impact a target population. Lastly, awareness raising and mentorship initiatives complement cash transfers by andomized beneficiaries and their household and community members on the timeline and use of cash transfers.

Table 21 presents the GRADE review of our findings (Table 16).

**Table 16:** GRADE summary of findings and certainty of evidence on cash transfers

Certainty assessment							Sample size	Effect	Certainty	Importance
No. of studies	Study design	Risk of bias	Inconsistency	Indirectness	Imprecision	Other considerations		Absolute (95% CI)		
*(AB2) Women's access to assets, income and savings (assessed with: self‐reports)*										
12	RCT‐11 QED‐1	Serious^a^	Serious^b^	Not serious	Not serious	Strong association	22,126	SMD 0.22SD higher (0.12 higher to 0.31 higher)	⊕⊕⊕◯ MODERATE	Important, but not critical
*(AB3) Women and girls have equitable access to livelihood support services*										
2	RCT‐2	Not serious	Not serious	Not serious	Not serious	None	3869	SMD 0.16SD higher (0.1 higher to 0.22 higher)	⊕⊕⊕⊕ HIGH	Important, but not critical
*(AC1) More women engaged in other micro, small and medium sized enterprises*										
2 (linked)	RCT	Not serious	Serious^c^	Not serious	Not serious	None	3288	Two linked studies reporting effect estimates of 0.81 and 0.91	⊕⊕⊕◯ MODERATE	Important, but not critical
*(AC2) Initiatives supported to facilitate women's access to decent work*										
6	RCT‐6	Serious^d^	Serious^e^	Not serious	Serious^f^	None	8651	SMD 0.18 SD higher (0.01 lower to 0.36 higher)	⊕◯◯◯ VERY LOW	Important, but not critical
*(AC3) Improved capacity of women entrepreneurs*										
1	RCT	Very serious^g^	Serious^c^	Not serious	Not serious	Strong association	1997	SMD 0.4 SD higher (0.32 higher to 0.49 higher)	⊕⊕◯◯ LOW	Important, but not critical
*(BA2) Women have more and better control over their bodies and sexual health*										
2	RCT	Serious^h^	Serious	Not serious	Not serious	None	6888	SMD 0.07 SD lower (−0.17 lower to 0.04 higher)	⊕⊕◯◯ LOW	Critical
*(BA3) Women have increased freedom of movement and association*										
3	RCT‐2 QED‐1	Serious^i^	Serious^j^	Not serious	Serious^k^	None	4021	SMD 0.01SD higher (−0.10 lower to 0.13 higher)	⊕◯◯◯ VERY LOW	Limited importance
*(BA6) Reduced percentage of women agreeing with certain reasons that justify violence against women and girls*										
3	RCT‐1 QED‐2	Serious^l^	Not serious	Not serious	Not serious	None	3098	SMD 0.11 SD higher (0.02 higher to 0.2 higher)	⊕⊕⊕◯ MODERATE	Critical
*(BA7) Women are equipped with better life skills that allow them to be prepared for crisis or shocks and recover from them*										
3	RCT‐3	Not serious	Not serious	Not serious	Serious^m^	None	5054	SMD 0.03 SD higher (0.04 lower to 0.1 higher)	⊕⊕⊕◯ MODERATE	Important, but not critical
*(BA8) Reduced instances of child or forced marriage*										
2	RCT	Not serious	Not serious	Not serious	Serious^n^	None	2109	SMD 0.07 SD higher (0.1 lower to 0.25 higher)	⊕⊕⊕◯ MODERATE	Critical
*(BB1) Women's decision‐making at the household and community levels (assessed with: self‐reports)*										
6	RCT‐5 QED‐1	Very serious^o^	Very serious^p^	Not serious	Serious	None	31,619	SMD 0.03 SD higher (0.03 lower to 0.9 higher)	⊕◯◯◯ VERY LOW	Limited importance
*(BB2) Women participate more in their communities*										
3	RCT‐2 QED‐1	Very serious^q^	Very serious^r^	Not serious	Serious^s^	None	6571	SMD 0.1 SD higher (0.07 lower to 0.28 higher)	⊕◯◯◯ VERY LOW	Limited importance
*(BC1) The rights, safety, and security of women, men, girls and boys are protected during relief, recovery and reconstruction*										
1	RCT	Serious^t^	Serious^c^	Not serious	Not serious	None	4112	Two positive effect estimates with a 95% CI range of 0.04 to 0.61	⊕⊕◯◯ LOW	Critical
*(CA2) Increased representation of women in local and subnational civil and political processes, including during peacebuilding and post conflict restoration*										
3 (Linked)	QED‐3	Serious^u^	Very serious^v^	Not serious	Not serious	Undetected	12,557	Three positive effect estimates with a 95% CI range of 0.02 to 0.3	⊕◯◯◯ VERY LOW	Limited importance
*(CB2) Communities have a more positive attitude towards women/andomizedi groups*										
1	RCT	Very serious^aa^	Serious^c^	Not serious^ab^	Serious^ac^	None	1800	One positive and one negative effect estimate with a 95% CI range of −0.20 to 0.11	⊕◯◯◯ VERY LOW	Limited importance
*(CB3) Women have improved attitudes, self‐image and confidence*										
3	RCT‐2 QED‐1	Not serious	Serious^ad^	Not serious	Serious^ae^	None	4863	SMD 0.09 SD higher (0.01 lower to 0.18 higher)	⊕⊕◯◯ LOW	Limited importance
*(CB4) Improved attitudes and increased support for women's economic, social and human rights by men, household and family members and community members*										
5	RCT‐3 QED‐2	Serious^af^	Serious^ag^	Not serious	Not serious	None	11,360	SMD 0.04SD higher (0 to 0.07 higher)	⊕⊕◯◯ LOW	Limited importance
*(CB6) Safer and more secure household, communities and areas/territories for women, girls, men and boys*										
2	RCT‐2	Serious^ah^	Serious^ai^	Not serious	Serious^aj^	None	2813	SMD 0.09SD higher (0.04 lower to 0.22 higher)	⊕◯◯◯ VERY LOW	Critical
*(CB7) Reduced frequency and distribution of types of violence by an intimate partner*										
4	RCT‐3 QED‐1	Serious^ak^	Very serious^al^	Not serious	Not serious	None	4368	SMD 0.05 SD higher (0.05 lower to 0.17 higher)	⊕◯◯◯ VERY LOW	Critical
*(CB8) Improved quality of relationships between women and their household and community members*										
4	RCT‐5	Serious^am^	Very serious^an^	Not serious	Serious^ao^	None	7405	SMD 0.03 SD higher (0.04 lower to 0.11 higher)	⊕◯◯◯ VERY LOW	Limited importance
*(CC1) Empowerment/Equality Index*										
5	RCT‐3 QED‐2	Very serious^ap^	Very serious^aq^	Not serious	Serious^ar^	Publication bias strongly suspected	8315	SMD 0.03 SD higher (0.1 lower to 0.15 higher)	⊕◯◯◯ VERY LOW	Important, but not critical

Abbreviations: CI, confidence interval; GRADE, Grading of Recommendations, Assessment, Development and Evaluations; QED, quasi‐experimental design; RCT, andomized controlled trial; SMD, andomizedi mean difference.

^a^ This group has been downgraded because three of twelve studies present high risk of bias. That said, when the highest risk of bias study is removed, the effect size is only very marginally impacted.

^b^ One downgrade has been applied here because there is inconsistency insomuch as there are estimate points whose 95% CI do not overlap at all. That said, this is not ‘very serious’ because there is overlap across eight of twelve studies, and those which do not are still presenting positive effect sizes (i.e., the results vary, but are all positive).

^c^ All single study groups downgraded once for inconsistency.

^d^ Downgraded once because, while four of six studies are of low or some concerns related to risk of bias, two present high risk. There is particular risk and uncertainty surrounding outcome measurement bias.

^e^ Downgraded once to capture the wide range of point estimates, including across both sides of the threshold, and the Cis of several studies that do not overlap.

^f^ Downgraded once because of wide Cis that cross both sides of the threshold.

^g^ Downgraded to very serious because this study presents significant risk of outcome measurement bias as well as a lack of clarity across other criteria.

^h^ This analysis has been downgraded once due to one study having a high risk of bias and the other presenting some concerns related to deviation from intended intervention although they are all RCTs.

^i^ This analysis has been downgraded once due to a mix of RCT and QED with only one study with low risk and the rest with some concerns related to risk of bias.

^j^ Downgraded to reflect Cis widely on both sides of the threshold and a lack of systematic overlap of Cis across different effect sizes.

^k^ Downgraded once as *p*‐value of 0.14 and sample size >400.

^l^ This analysis has been downgraded once due to a mix of RCT and QED with RCT with low risk and the rest with some concerns related to risk of bias.

^m^ Downgraded once as *p*‐value of 0.46 and sample size >400.

^n^ Downgraded because the CI's of the effect estimate cross the threshold considerably.

^o^ This group has been downgraded on risk of bias because three studies present high risk of bias across a number of domains (reporting bias, selection bias). That said, when high risk of bias studies were removed from analysis, the finding only marginally changes and remains positive.

^p^ Downgraded twice because (1) there are effect sizes whose confidence intervals do not systematically overlap, and who present both positive and negative impacts, and (2) *Q*‐score and $I^{2}$ indicate significant heterogeneity which cannot be explained by moderators.

^q^ Downgraded twice: (1) mix of QED and RCT, (2) two studies are low risk and one RCT is high risk of bias (3) removing either study would have an impact on the meta‐analysis.

^r^ Downgraded twice: (1) variation of points estimates: data on both side of the threshold, (2) overlaps of CI: no systematic overlap, (3) $I^{2}=92.17\%$, (4) test of stat: According to the *Q*‐test, the true outcomes appear to be heterogeneous.

^s^ Downgrade once because of a *p*‐value of 0.26 and sample size >400.

^t^ BC1 The rights, safety and security of women, men, girls and boys are protected during relief, recovery and reconstruction.

^u^ Downgraded once because the body of evidence is comprised three QED studies two of three of them raised some concerns related to risk of bias.

^v^ Downgraded once to reflect the range of point estimates.

^aa^ Downgraded because of risk of bias relating to the unit of analysis and a lack of clarity across other criteria.

^ab^ Downgraded once because the point estimates of this study cross both sides of the threshold and have a wide 95% CI.

^ac^ Downgraded for imprecision because the CI of the meta‐analytic effect size crosses the threshold while also crossing a non‐trivially beneficial level.

^ad^ Downgraded once: (1) variation of points estimates: data on same side of the threshold, (2) overlaps of CI: systematic overlap, (3) $I^{2}=59.86\%$, (4) test of stat: According to the *Q*‐test, some heterogeneity may still be present in the true outcomes.

^ae^ Downgraded once as *p*‐value of 0.07 and sample size >400.

^af^ Downgraded once because some studies presented unclear risk of bias, and less than half of the studies included are of low risk of bias.

^ag^ Downgraded once because although there is overlap in the confidence intervals of effect sizes, some are positive while others are negative. Additionally, $I^{2}=6.43\%$.

^ah^ Downgraded once because half of the studies are rated as having high risk of bias with particular concern for outcome measurement bias.

^ai^ Downgraded once because of widely varying point estimates that do not fall with the Cis of the other study.

^aj^ Downgraded once for wide Cis around the point estimate that cross both sides of the threshold.

^ak^ This group has been downgraded once because several studies are ‘unclear’ in a number of risks of bias categories (namely in performance bias and outcome measurement bias). That said, no studies in this group present high risk of bias.

^al^ Two downgrades have been applied for inconsistency because (1) There are effect sizes whose confidence intervals do not overlap, and who present both positive and negative impacts, and (2) The $I^{2}=68.34\%$ suggests that some inconsistency can only be interpreted as due to chance, and not heterogeneity.

^am^ This group has been downgraded once (1) all studies are RCTs but one of them has a high risk of bias, two with some concerns related to risk of bias and two low risk. (2) The study with high risk of bias represents a high proportion of results of the meta‐analysis.

^an^ Downgraded twice: (1) variation of points estimates: data on both side of the threshold, (2) overlaps of CI: no systematic overlap, (3) $I^{2}=56.28\%$, (4) test of stat: According to the *Q*‐test, some heterogeneity may still be present in the true outcomes.

^ao^ Downgraded once as *p*‐value of 0.15 and sample size >400.

^ap^ This group has been downgraded twice: (1) mix of RCT and QED, (2) one study is high risk, two are medium and two are low risk, (3) removing the high risk would affect the results of the meta‐analysis.

^aq^ Downgraded twice: (1) variation of points estimates: data on both side of the threshold, (2) overlaps of CI: no systematic overlap, (3) $I^{2}=86.36\%$, (4) test of stat: According to the *Q*‐test, the true outcomes appear to be heterogeneous.

^ar^ Downgraded once as *p*‐value of 0.68 and sample size >400.

#### 5.3.3 Community‐based services

Community‐based services are the improved public provision of services and support to families and individuals at the local and/or community level. This can include health, education, economic service provision and/or counselling and networking support.

##### 5.3.3.1 How do community‐based services affect gender equality, women's empowerment and Peace outcomes?

**Figure 52:**
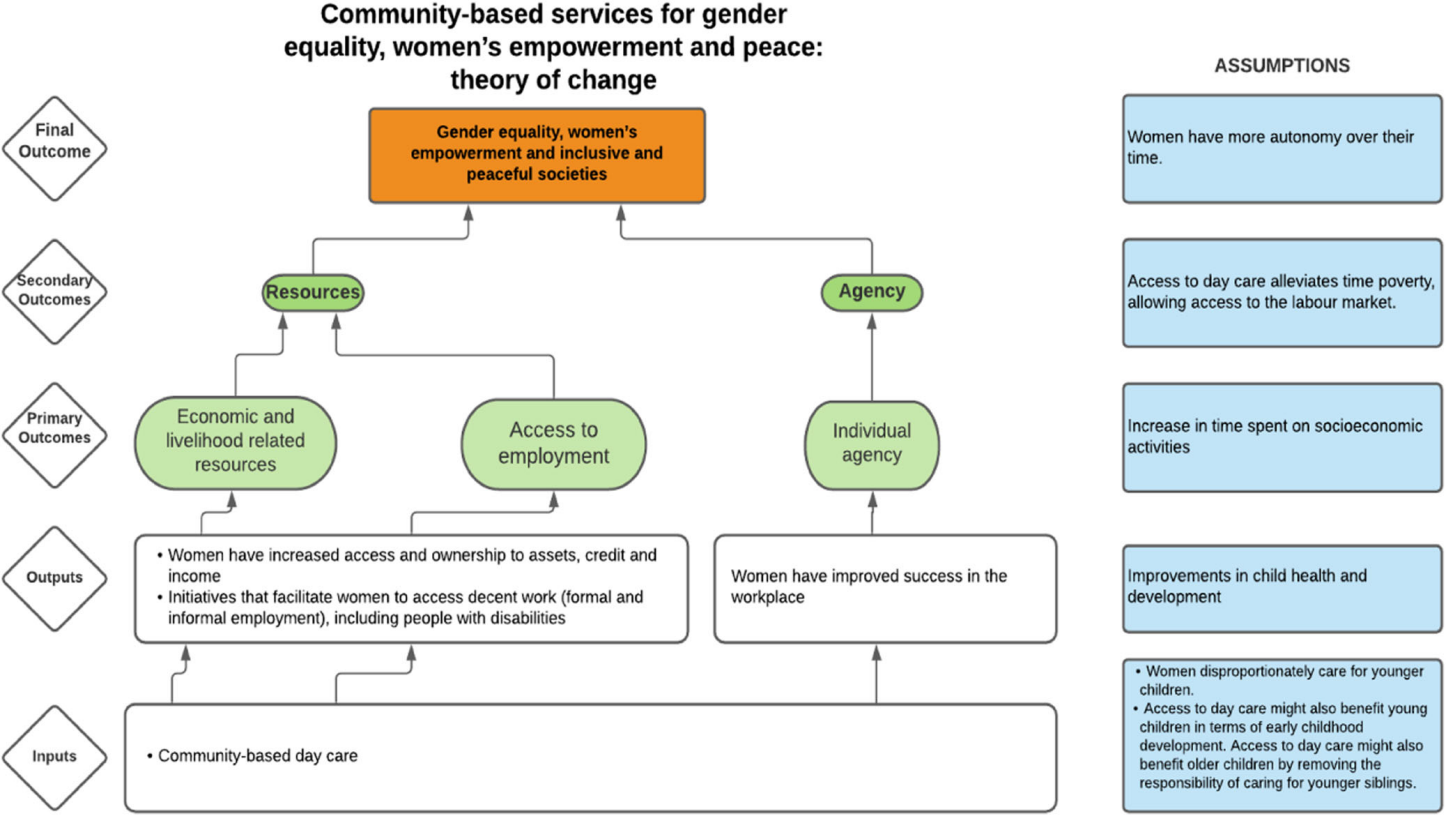
Community‐based services for gender equality, women's empowerment and peace: Theory of change

Figure 52 maps out the causal chain of how community‐based services may improve gender equality, women's empowerment and peace outcomes.

As illustrated in Figure 52, community‐based service initiatives that are inclusive (such as community‐based day care services to reduce the burden of childcare) can help societies to achieve high levels of societal resources and agency, which are essential to the overall empowerment of women and the fulfilment of an inclusive and peaceful society. These resources and individual agency rely on access to economic and livelihood related resources, access to employment, and individual agency, and are fostered by immediate outcomes that are generated from inclusive community‐based service programmes. Immediate outcomes of such programmes include women having increased access and ownership to assets, credit and income, facilitating women to access decent work (formal and informal employment) and improving their success in the workplace.

##### 5.3.3.2 Description of included studies

We included only one study that evaluated the effect of community‐based services (Table 22).

##### 5.3.3.3 Population

The included study evaluated one programme in South Asia, in one country: India.

##### 5.3.3.4 Intervention, inputs and activities

The included study evaluated the following community‐based service activity:

- Community‐based day care (*n* = 1): Programme implementation was structured around one major intervention at the village level, the provision of childcare, nutritious food and supplements, basic medicines and preschool education to children 1–6 years old (Table 17).

**Table 17:** Community‐based services features of included studies

Study	Activity/input	Length of treatment	Intervention frequency
Nandi et al. (2020)	Community‐based day care services	24 months	Daily

##### 5.3.3.5 Comparison

The included study compared treated groups to comparison groups receiving no intervention. No study included multiple treatment arms.

##### 5.3.3.6 Outcomes

The included study reported on a number of relevant outcomes:

- Women have increased access and ownership to assets, credit and income (*n* = 1): Women can apply for, receive and manage assets/credit and income and have support to manage, claim and execute their assets without pressure or influence from external actors, including male family members, husbands and cultural leaders.
- Initiatives that facilitate women to access decent work (formal and informal employment), including people with disabilities (*n* = 1): Women are able to apply for, receive and work in jobs (and have support for the above), without discrimination for sex, gender or other identifying factors, including development of skills for improved access.

The division of the immediate and secondary outcomes is reported in Table 23 (Table 18).

**Table 18:** Summary of secondary and immediate outcomes for community‐based services

Secondary outcome category	Immediate outcome	Number of studies
Resources material, human and social resources which serve to enhance the ability to exercise choice	Access to justice and legal services	0
	Economic and livelihood related resources	1
	Access to employment	1
Agency ability to define one's goals and act upon them and andomizedized decision‐making	Individual agency	1
	Community level agency	0
	Institutions supporting agency	0
Achievement ways of being and doing which can be andomiz by different individuals	Improved systems	0
	Norms and behaviour change	0
	Empowerment index	0

##### 5.3.3.7 Study design

**Figure 53:**
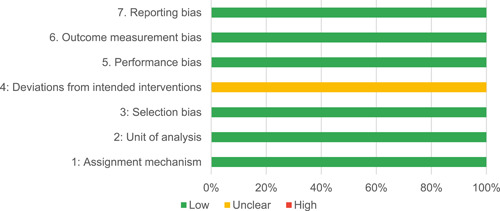
Randomised controlled trial community based services risk of bias assessment

Our only community‐based services study was Nandi et al. (2020). It used an experimental design with no major concern but potential contamination issues where children from control villages attended day‐care services in neighbouring treatment villages. We did not identify any other limitation related to any of the risk of bias domains (assignment mechanism, unit of analysis, selection, performance, outcome measurement and reporting), attrition was relatively low and similar between treatment and control groups, baseline characteristics were balanced for both groups, and allocation was concealed until after the baseline survey to andomiz opportunities for bias in recruitment of participants. Figure 53 presents the results of the RoB assessment.

##### 5.3.3.8 Qualitative studies, process evaluations and project documents

We identified no linked qualitative studies and were unable to conduct a qualitative evidence synthesis related to the community‐based services intervention.

##### 5.3.3.9 Synthesis of findings

The following subsection presents the results of effectiveness of community‐based services on gender equality, women's empowerment and peace outcomes.

##### 5.3.3.10 Quantitative findings

*Effects of community‐based services on women accessing decent work (formal and informal employment)*

Nandi and colleagues' (2020) experimental study in India also evaluated the impact of community‐based services on women to access decent work (formal and informal employment). Their report included three effects that fell into this outcome category (e.g., worked in past 7 days). The effects ranged from very small, negative estimates (*g *=* *−0.02, 95% CI [−0.09, 0.05]) to very small, positive estimates (*g *=* *0.02, [95% CI: −0.05 to 0.09]), but none were statistically significant. We assessed the study as having some risk of bias concerns.

##### 5.3.3.11 Summary of findings and discussion

We included only one study in India that evaluated the effect of community‐based services. We examined effects on two outcomes: women have increased access and ownership to assets, credit and income and women's access decent work (formal and informal employment). Our included study reported against one of the three secondary outcomes (Resources, Agency and Achievement) and two of the nine immediate outcomes for our review. We found no evidence of the community day care service being effect for increasing outcomes related to women's empowerment and gender equity. Overall, the GRADE assessments (Table 24) generally indicate a low certainty in this body of evidence.

We identified no linked qualitative studies and were unable to conduct a qualitative evidence synthesis related to the community‐based services intervention (Table 19).

**Table 19:** GRADE summary of findings and certainty of evidence on community‐based services

Certainty assessment								Effect	Certainty	Importance
No of studies	Study design	Risk of bias	Inconsistency	Indirectness	Imprecision	Other considerations	Sample size	Absolute (95% CI)		
*(AB2) Women have increased access and ownership to assets, credit and income*										
1	RCT	Serious^a^	Serious^b^	Not serious	Not serious	None	2859	Two (identical) positive effect estimates of 0.02 (−0.05, 0.09)	⊕⊕◯◯ LOW	Important, but not critical
*(AC2) Initiatives supported that facilitate women to access decent work (formal and informal employment), including people with disabilities*										
1	RCT	Serious^a^	Serious^b^	Not serious	Not serious	None	2859	Two negative and one positive effect estimate with a 95% CI range of −0.09 to 0.09	⊕⊕◯◯ LOW	Important, but not critical

Abbreviations: CI, confidence interval; GRADE, Grading of Recommendations, Assessment, Development and Evaluations; RCT, andomized controlled trial.

^a^ Downgraded once due to issues of bias relating to deviation from intended intervention.

^b^ All single studies downgraded once for inconsistency.

#### 5.3.4 Inclusive community‐driven development

We define inclusive community‐driven development as development initiatives that put control of the development process, resources and decision‐making authority directly towards groups in a community. Community‐driven development (CDD) programmes operate on principles of transparency, participation, local empowerment, demand‐responsiveness, greater downward accountability and enhanced local capacity (World Bank, 2021a, 2021b). This enhances participatory involvement at the local level (White et al., 2018). In our case, CDD can include a andomizedi group in the decision‐making process or as beneficiaries. It is a bottom‐up model of development that aims to increase participation among the beneficiaries of development.

##### 5.3.4.1 How does inclusive community‐driven development affect gender equality, women's empowerment and Peace outcomes?

**Figure 54:**
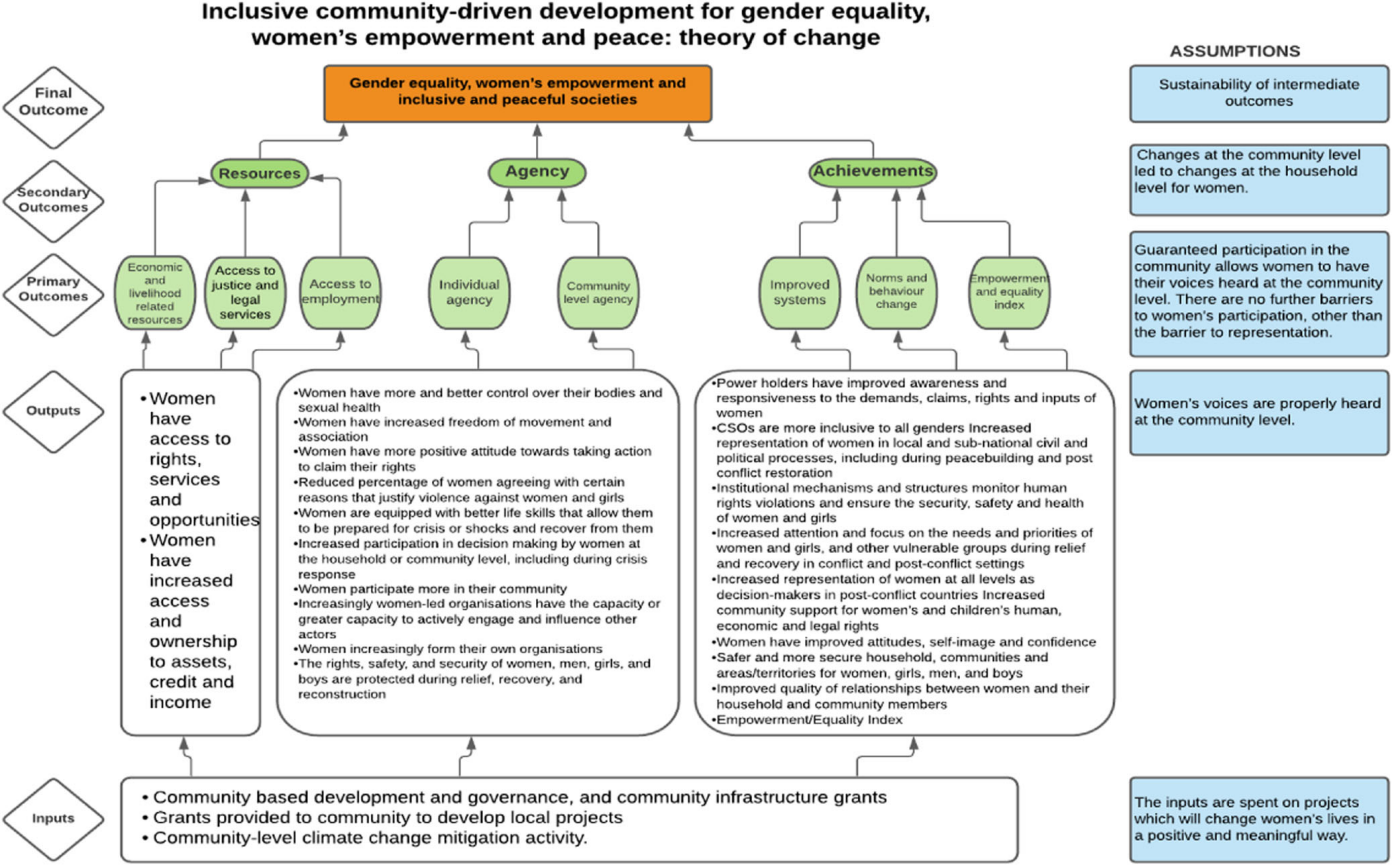
Inclusive community‐driven development for gender equality, women's empowerment and peace: Theory of change

Figure 54 maps out the causal chain of how inclusive community‐driven development may improve gender equality, women's empowerment and peace outcomes.

As illustrated in Figure 54, community‐driven development initiatives that are inclusive (such as community‐based governance as well as provision of resources to tackle community challenges) can help societies to achieve high‐level resources, agency and achievements, which are essential to the overall empowerment of women and inclusive and peaceful society. These resources, agency and achievements rest on access to justice and legal services, livelihood related resources, individual and community agency, norms and behaviour change, among others, and are fostered by immediate outcomes that are generated from inclusive community‐driven development programmes. Immediate outcomes of such programmes include women's access to rights and services, improved participation in decision making, improved freedom, among many others.

##### 5.3.4.2 Description of included studies

We included three programme studies reported in eight different papers that evaluated the effect of inclusive community‐driven development programmes. We included more than one paper that evaluated the same programme if the author(s) reported different outcomes over several papers.

##### 5.3.4.3 Population

The included studies andomi on individuals as well as entire communities, with special attention given in many cases to opinion leaders such as village chiefs. The included studies evaluated three different programmes in Latin America, East Asia and the Pacific, South Asia and Sub‐Saharan Africa in eight countries: Brazil, Cameroon, Indonesia, Peru, Tanzania, Vietnam, Afghanistan and DRC.

##### 5.3.4.4 Intervention, inputs and activities

The included studies evaluated a range of different inclusive community‐driven development activities and inputs including the followings:

- Community‐based development and governance and community infrastructure grants (*n* = 3): Programme implementation was structured around two major interventions at the village level: (i) the election of a gender‐balanced community development council (CDC) through a secret‐ballot, universal suffrage election centred on democratic processes and women's participation; and (ii) the provision of ‘block grants’—valued at $200 USD per household, up to a village maximum of $60,000 USD and averaging $33,000 USD—to fund village‐level projects designed and selected by CDCs in consultation with villagers. Projects generally fall into one of six categories: transport; water and sanitation; irrigation; power; literacy and vocational training; and others.
- Community‐level climate change mitigation activity (*n* = 1): REDD+ initiatives vary across countries but typically involve work to prevent deforestation by working with community‐level NGOs.
- Grant provided to community to develop local projects (*n* = 4): Tuungane 1 had two components: (i) an intervention at the village development committee level that involved $3,000 USD grants, and subsequently; (ii) an intervention at the CDC level that involved $50,000–$70,000 USD sub‐grants to undertake infrastructure projects such as the construction of schools and hospitals. A core element of Tuungane 1 was a focus on women's empowerment and on championing the rights and roles of women in collective decision‐making (Table 20).

**Table 20:** Inclusive community‐driven development activities design features of included studies

Study	Activity/input	Length of treatment	Intervention frequency
Beath (2013b)	Community‐based development and governance and community infrastructure grants	50 months	N/A
Beath et al. (2015)	Community‐based development and governance and community infrastructure grants	50 months	N/A
Beath et al. (2013a, 2013b)	Community‐based development and governance and community infrastructure grants	50 months	N/A
Larson et al. (2018)	Community‐level climate change mitigation activity	48 months	Annually
Laudati et al. (2018)	Grant provided to community to develop local projects	48 months	N/A
Mvukiyehe and van der Windt (2020)	Grant provided to community to develop local projects	48 months	N/A
Van der Windt et al. (2018)	Grant provided to community to develop local projects	48 months	N/A
Van der Windt et al. (2018) (linked paper)	Grant provided to community to develop local projects	48 months	N/A

##### 5.3.4.5 Comparison

All included studies compared treated groups to comparison groups receiving no intervention. One study included multiple treatment arms.

##### 5.3.4.6 Outcomes

The included studies reported on a number of relevant outcomes, including the following:

- Women have increased freedom of movement and association (*n* = 5): Women and girls are able to move freely and meet within their household, community, and area/territory without fear of attack or discrimination.
- Increased representation of women in local and subnational civil and political processes, including during peacebuilding and post conflict restoration (*n* = 6): Women are represented in the institutions, organisations and other decision‐making processes and have capacities to influence the decision and direction of these processes. Women's specific needs and requests are taken into account and are reflected in the final decision, giving a full role to women in civil and political processes.
- Women have improved attitudes, self‐image and confidence (*n* = 2): Women feel more entitled to claim their rights and needs in community and change social norms and behaviours. They are aware of the importance of their status in society and are empowered to take this role and make use of the available resources to guarantee their rights.
- Safer and more secure household, communities and areas/territories for women, girls, men, and boys (*n* = 4): We report the division of the immediate and secondary outcomes in Table 26 (Table 21).

**Table 21:** Inclusive community‐driven development summary of secondary and immediate outcomes

Secondary outcome category	Immediate outcome	Number of studies
Resources material, human and social resources which serve to enhance the ability to exercise choice	Access to justice and legal services	1
	Economic and livelihood related resources	2
	Access to employment	0
Agency ability to define one's goals and act upon them and andomizedized decision‐making;	Individual agency	6
	Community level agency	4
	Institutions supporting agency	0
Achievement ways of being and doing which can be andomiz by different individuals	Improved systems	6
	Norms and behaviour change	5
	Empowerment index	0

##### 5.3.4.7 Study design

One of our inclusive community‐driven development studies used a QED (Larson et al., 2018). It was assessed as having high risk of bias because of reporting issues related to a lack of details provided on the matching methods used for identifying a valid comparison group. The extent to which matching methods were able to control for a comprehensive set of time‐varying characteristics that might confound the impact of the program was unclear, tests for hidden bias were not reported, nor were sensitivity analyses presented, and balance after matching was not tested. Further, no reasons were discussed for eliminating 25 villages from the data set. Therefore, we assessed the study as having high risk of confounding, outcome measurement and reporting bias. Yet, we did not identify any implementation issues or limitations because of spill‐overs, crossovers or contamination.

**Figure 55:**
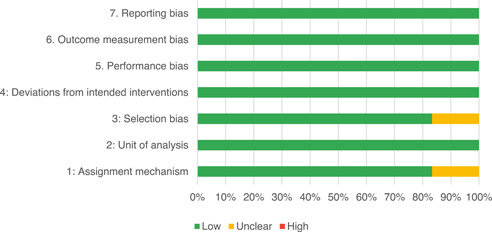
Inclusive community‐driven development studies andomized controlled trial risk of bias assessment

Six of our inclusive community‐driven development studies used an experimental design. These include Beath et al. (2013a, 2013b, 2015), Laudati et al. (2018), Mvukiyehe and van der Windt (2020) and Van der Windt et al. (2018). Overall, no major limitation was identified for this set of studies where two thirds were assessed as having a low risk of bias. As detailed in Figure 55, high risk of bias was not identified in any RoB domains of the sample of included studies. The only concerns observed in RCT studies evaluating inclusive community‐driven development interventions were the lack of a balance table in the report (Beath et al., 2015) and risk of selection bias because of a large proportion of villages were not surveyed during the follow‐up due to security conditions (Beath et al., 2013a, 2013b). Other than that, no limitations were identified with regard to unit of analysis, deviations from intended intervention, performance, outcome measurement and reporting.

##### 5.3.4.8 Qualitative studies, process evaluations and project documents

We identified six additional documents related to two programmes covered by the inclusive CDD group.

- National Solidarity Programme (NSP) (Afghanistan): four qualitative documents
- Tuungane 1 (DRC one qualitative and one descriptive quantitative documents

Most (*n* = 5) of the included qualitative studies were marked as high empirical quality, with the rest (*n* = 1) marked as moderate.

##### 5.3.4.9 Synthesis of findings

*Quantitative findings*

*Effects of inclusive community‐driven development on women's access to rights, services and opportunities*

Beath and colleagues’ (2013a, 2013b) experimental study in Afghanistan was the only study evaluating the impact of inclusive community‐driven development on women's access to rights, services and opportunities. Their report included five effects that fell into this outcome category (e.g., girl aspires to work in professional occupation). The effects ranged from very small, negative estimates (*g *=* *−0.004, [95% CI: −0.07 to 0.06]) to very small, estimates (*g *=* *0.09, [95% CI: −0.02 to 0.20]). We assessed the study was assessed as having low risk of bias.

*Effects of inclusive community‐driven development on women's access and ownership to assets, credit and income*

Beath and colleagues’ (2013a, 2013b) experimental study in Afghanistan was the only one evaluating the impact of inclusive community‐driven development on women's access and ownership to assets, credit and income. Their reports included four effects that fell into this outcome category (e.g., generated income in past year). The effects ranged from very small, negative estimates (*g *=* *−0.01, [95% CI: −0.05 to 0.04]) to small, positive effects (*g *=* *0.11, [95% CI: 0.05 to 0.17]). We assessed the studies as having low risk of bias and some concerns, respectively.

*Effects of inclusive community‐driven development on women having more and better control over their bodies and sexual health*

Beath and colleagues' (2013a) experimental study in Afghanistan was the only study evaluating the impact of inclusive community‐driven development on women having more and better control over their bodies and sexual health. There was a very small, but not statistically significant, estimate (*g *=* *−0.02, [95% CI: −0.06 to 0.03]) and we assessed the study as having low risk of bias.

*Effects of inclusive community‐driven development on women having increased freedom of movement and association*

**Figure 56:**
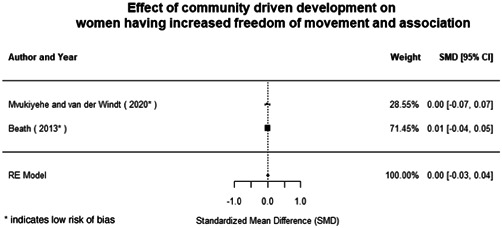
CDDBA3: Forest plot showing the observed outcomes and the estimate of the random‐effects model. CI, confidence interval

For increased freedom of movement and association, two studies reported disaggregated data for women, thus we included a total of k=2 studies in the analysis. We assessed both studies as having low risk of bias. The estimated average outcome based on the random‐effects model was $\hat{\mu }=0.004$ (95% CI: −0.03 to 0.04). Therefore, the average outcome did not differ significantly from zero (z=0.21, p=0.83). A forest plot showing the observed outcomes and the estimate based on the random‐effects model is shown in Figure 56 (CDDBA3). According to the Q‐test, there was no significant amount of heterogeneity in the true outcomes (Q(2)=0.02, p=0.89, ${\hat{\tau }}^{2}=0.00$, $I^{2}=0.00$%). Given the small number of studies and that there was no heterogeneity among the effects, moderator analyses were not possible.

*Effects of inclusive community‐driven development on women having more positive attitude towards taking action to claim their rights*

**Figure 57:**
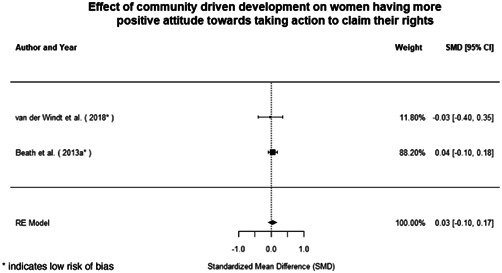
CDDBA5: Forest plot showing the observed outcomes and the estimate of the random‐effects model. CI, confidence interval

For women having more positive attitude towards taking action to claim their rights, two studies reported disaggregated data, thus we included *k *=* *2 studies in the analysis. We assessed both studies as having low risk of bias. The estimated average outcome based on the random‐effects model was $\hat{\mu }=0.03$ (95% CI: −0.10to0.17). Therefore, the average outcome did not differ significantly from zero (z=0.52, p=0.60). A forest plot showing the observed outcomes and the estimate based on the random‐effects model is shown in Figure 57 (CDDBA5). Given the small number of studies, this result should be interpreted with caution. According to the Q‐test, there was no significant amount of heterogeneity in the true outcomes (Q(1)=0.11, p=0.74, ${\hat{\tau }}^{2}=0.00$, $I^{2}=0.00$%). With only two studies, and given there was no heterogeneity among the effects, moderator analyses were not possible, and tests of publication bias are not valid.

*Effects of inclusive community‐driven development on women agreeing with certain reasons that justify violence against women and girls*

**Figure 58:**
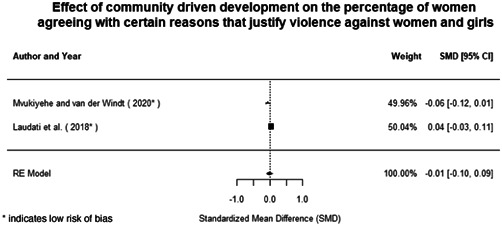
CDDBA6: Forest plot showing the observed outcomes and the estimate of the random‐effects model. CI, confidence interval

For women agreeing with certain reasons that justify violence against women and girls, two studies reported disaggregated data, thus we included *k *=* *2 studies in the analysis. We assessed both studies as having low risk of bias. The estimated average outcome based on the random‐effects model was $\hat{\mu }=-0.01$ (95% CI: −0.10to0.08). Therefore, the average outcome did not differ significantly from zero (z=−0.1512, p=0.88). A forest plot showing the observed outcomes and the estimate based on the random‐effects model is shown in Figure 58 (CDDBA6). Given the small number of studies, this result should be interpreted with caution. According to the Q‐test, there was some amount of heterogeneity in the true outcomes (Q(1)=3.99, p=0.05, ${\hat{\tau }}^{2}=0.06$, $I^{2}=74.95$%). However, with only two studies moderator analyses were not possible, and tests of publication bias are not valid.

*Effects of inclusive community‐driven development on women being equipped with better life skills that allow them to be prepared for crisis or shocks and recover from them*

Beath and colleagues' (2013a) experimental study in Afghanistan was the only study evaluating the impact of inclusive community‐driven development on women being equipped with better life skills that allow them to be prepared for crisis or shocks and recover from them. Their report included two effects that fell into this outcome category (days girl attended school in past week and girl correctly completed basic calculation). The effects were very small and positive (*g *=* *0.07, [95% CI: 0.01 to 0.12], and *g *=* *0.10, [95% CI: −0.01 to 0.21], respectively). We assessed the study as having low risk of bias.

*Effects of inclusive community‐driven development on participation in decision making by women at the household or community level, including during crisis response*

Beath and colleagues' (2013a) experimental study in Afghanistan was the only study evaluating the impact of inclusive community‐driven development on increased participation in decision making by women at the household or community level, including during crisis response. Their report included four effects that fell into this outcome category (e.g., exerts control over income earned). The effects ranged from very small, negative effects (*g *=* *−0.05, 95% CI [−0.11, 0.01]) to very small, positive effects (*g *=* *0.05, [95% CI: −0.01 to 0.11]). We assessed the study as having low risk of bias.

*Effects of inclusive community‐driven development on women's participation in their community*

Van der Windt and colleagues' (2018) experimental study in Democratic Republic of Congo was the only study evaluating the impact of inclusive community‐driven development on women participating more in their community. There was a very small, but not statistically significant, effect (*g *=* *−0.05, 95% CI [−0.43, 0.33]), and we assessed the study as having low risk of bias.

*Effects of inclusive community‐driven development on women forming their own organisations*

Beath and colleagues's (2013b) experimental study in Afghanistan was the only study evaluating the impact of inclusive community‐driven development on women forming their own organisations. Their report included three effects that fell into this outcome category (e.g., there exists a village or pan‐village women's council). The effects ranged from medium, positive effects (*g *=* *0.33, 95% CI [0.14, 0.52]) to large, positive effects (*g *=* *0.88, 95% CI [0.69, 1.08]). We assessed the study as having some risk of bias concerns.

*Effects of inclusive community‐driven development on power holders' awareness and responsiveness to the demands, claims, rights and inputs of women*

Beath and colleagues' (2013a) experimental study in Afghanistan was the only study evaluating the impact of inclusive community‐driven development on power holders' awareness and responsiveness to the demands, claims, rights and inputs of women. There was a very small, but not statistically significant, effect (*g *=* *0.03, 95% CI [−0.01, 0.08]), and we assessed the study as having low risk of bias.

*Effects of inclusive community‐driven development on representation of women in local and subnational civil and political processes, including during peacebuilding and post conflict restoration*

**Figure 59:**
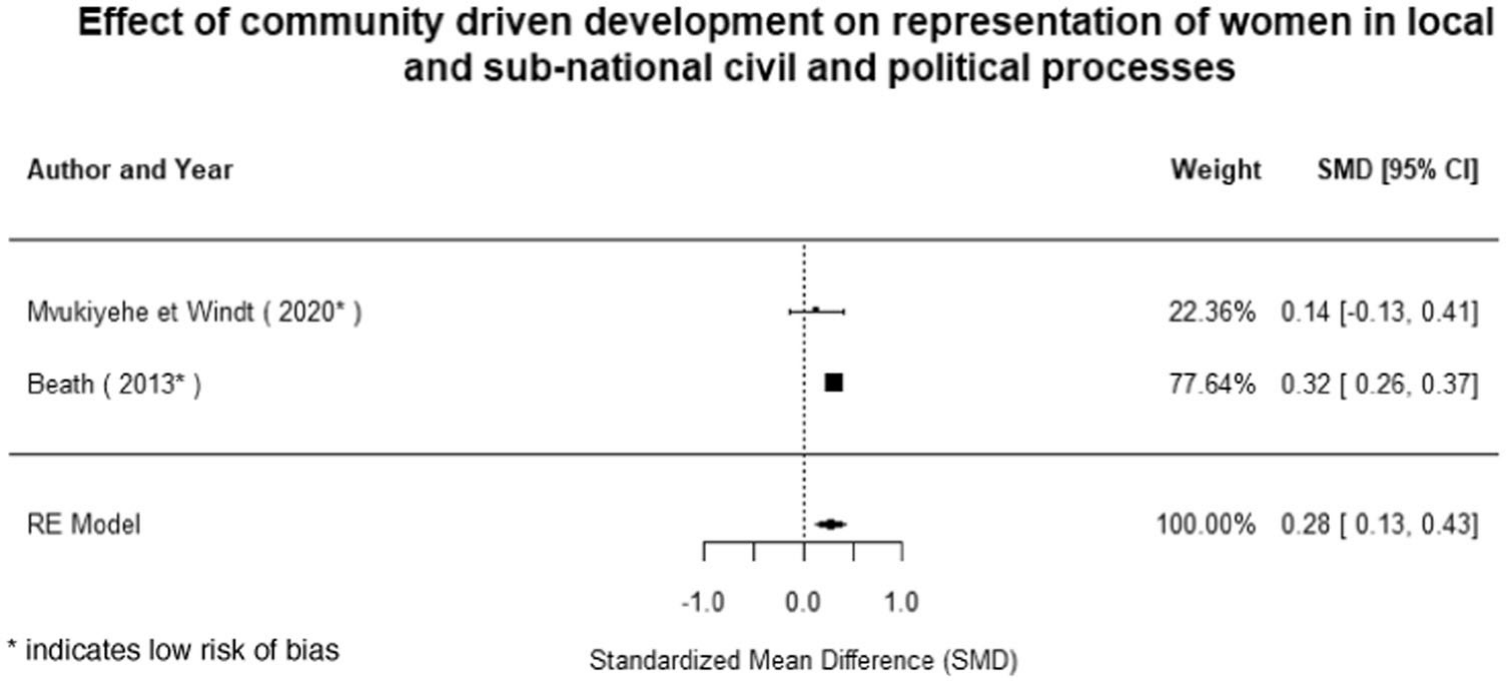
CDDCA2: Forest plot showing the observed outcomes and the estimate of the random‐effects model. CI, confidence interval

Two studies reported disaggregated data for representation of women in local ans subnational civil and political processes, including during peace building and post conflict restoration, thus we included a total of k=2 studies in the analysis We assessed both studies as having low risk of bias. The estimated average outcome based on the random‐effects model was $\hat{\mu }=0.28$ (95% CI: 0.13 to 0.43). Therefore, the average outcome differed significantly from zero (z=3.67, p<0.001). A forest plot showing the observed outcomes and the estimate based on the random‐effects model is shown in Figure 59 (CDDCA2). According to the Q‐test, there was no significant amount of heterogeneity in the true outcomes (Q(1)=1.66, p=0.20, ${\hat{\tau }}^{2}=0.01$, $I^{2}=39.79$%). With only two studies moderator analyses were not possible and tests of publication bias are not valid.

*Effects of inclusive community‐driven development on institutional mechanisms and structures to monitor human rights violations and ensure the security, safety and health of women and girls*

Beath and colleagues' (2013a) experimental study in Afghanistan was the only study evaluating the impact of inclusive community‐driven development on institutional mechanisms and structures monitor human rights violations and ensure the security, safety and health of women and girls. There was a very small, but not statistically significant, effect (*g *=* *0.01, 95% CI [−0.03, 0.05]), and we assessed the study as having low risk of bias.

*Effects of inclusive community‐driven development on attention and focus on the needs and priorities of women and girls, and other vulnerable groups during relief and recovery in conflict and post‐conflict settings*

Beath and colleagues' (2013a) experimental study in Afghanistan was also the only study evaluating the impact of inclusive community‐driven development on increased attention and focus on the needs and priorities of women and girls, and other vulnerable groups during relief and recovery in conflict and post‐conflict settings. Their report included two effects that fell into this outcome category (women's views considered in aid allocation and males' response on women's views considered in aid allocation). The effects were positive and statistically significant, very small for males' response (*g *=* *0.08, 95% CI [0.02, 0.14]) and small for women's views being considered for aid allocation (*g *=* *0.12, 95% CI [0.05, 0.19]). We assessed the study as having low risk of bias.

*Effects of inclusive community‐driven development on increased representation of women at all levels as decision‐makers in post‐conflict countries*

Beath and colleagues' (2013a) experimental study in Afghanistan was also the only study evaluating the impact of inclusive community‐driven development on increased representation of women at all levels as decision‐makers in post‐conflict countries. Their report included four effects that fell into this outcome category (e.g., women helped mediate most recent dispute). The effects ranged from very small, positive effects (*g *=* *0.05, 95% CI [−0.02, 0.12]) to small, positive effects (*g *=* *0.10, 95% CI [0.04, 0.16]), with all of the effects being positive. We assessed the study as having low risk of bias.

*Effects of inclusive community‐driven development on increased community support for women's and children's human, economic and legal rights*

**Figure 60:**
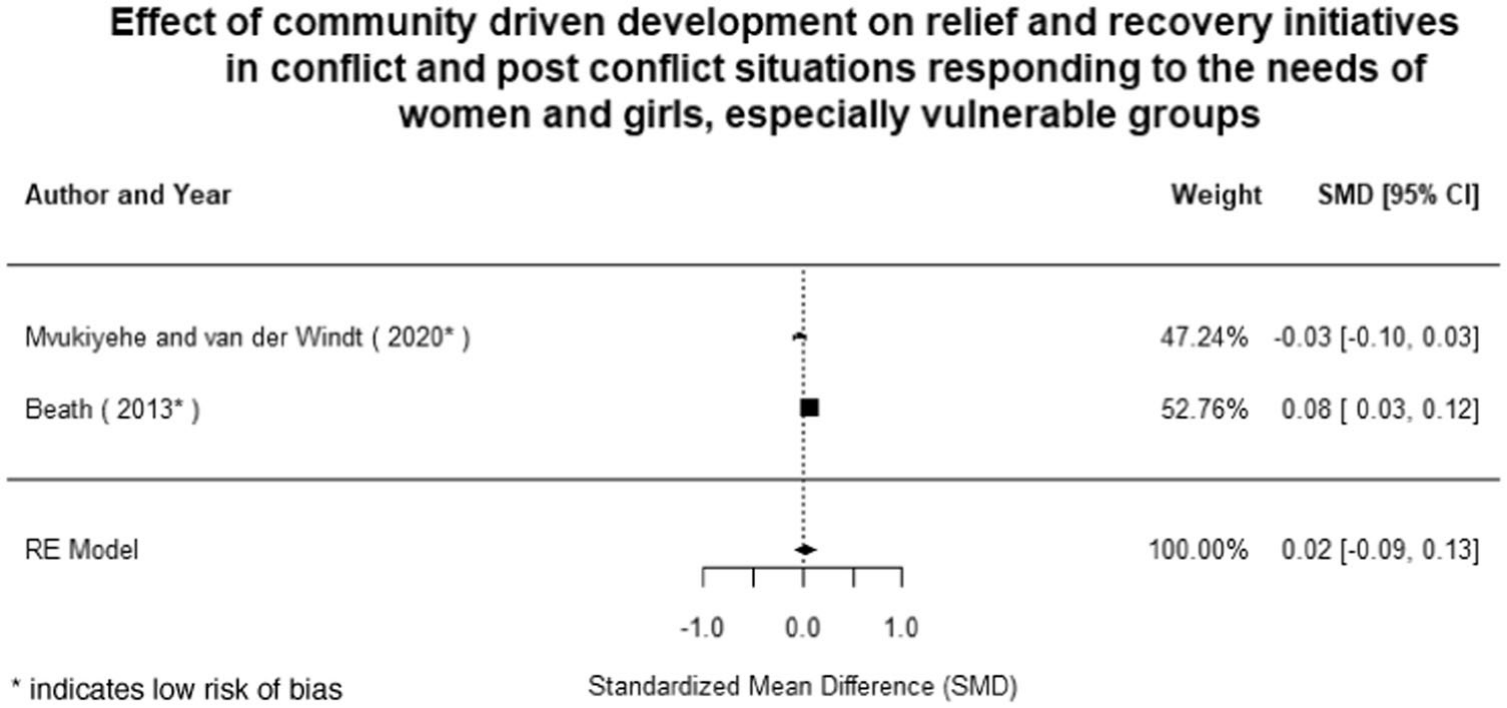
CDDCA8: Forest plot showing the observed outcomes and the estimate of the random‐effects model. CI, confidence interval

Two studies reported disaggregated data for community support for women's and children's human, economic and legal rights, thus we included a total of k=2 studies in the analysis. We assessed both studies as having low risk of bias. The estimated average outcome based on the random‐effects model was $\hat{\mu }=0.02$ (95% CI: −0.09 to 0.13). Therefore, the average outcome did not differ significantly from zero (z=0.44, p=0.66). A forest plot showing the observed outcomes and the estimate based on the random‐effects model is shown in Figure 60 (CDDCA8). According to the Q‐test, the true outcomes appear to be heterogeneous (Q(3)=7.43, p<0.01, ${\hat{\tau }}^{2}=0.01$, $I^{2}=86.53$%). With only two studies, moderator analyses were not possible.

*Effects of inclusive community‐driven development on CSOs being more inclusive to all genders*

Beath and colleagues' (2013b) experimental study in Afghanistan was the only study evaluating the impact of inclusive community‐driven development on CSOs being more inclusive to all genders. Their report included two effects that fell into this outcome category (male and female respondent believes it is appropriate for women to work with NGOs). The effects were very small and positive, but not statistically significant for male respondents (*g *=* *0.03, 95% CI [−0.03, 0.09]) whereas statistically significant for female respondents (*g *=* *0.08, 95% CI [0.02, 0.14]). We assessed the study as having some risk of bias concerns.

*Effects of inclusive community‐driven development on women having improved attitudes, self‐image and confidence*

**Figure 61:**
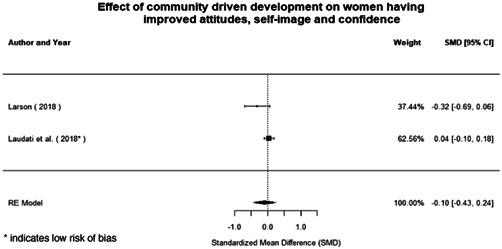
CDDCB3: Forest plot showing the observed outcomes and the estimate of the random‐effects model. CI, confidence interval

For women having improved attitudes, self‐image and confidence, two studies reported disaggregated data, thus we included *k *=* *2 studies in the analysis. We assessed one study as having low risk of bias, none as some concerns, and one as high risk of bias. The estimated average outcome based on the random‐effects model was $\hat{\mu }=-0.0958$ (95% CI: −0.4289to0.2374). Therefore, the average outcome did not differ significantly from zero (z=−0.5634, p=0.5732). A forest plot showing the observed outcomes and the estimate based on the random‐effects model is shown in Figure 61 (CDDCB3). Given the small number of studies, this result should be interpreted with caution. According to the Q‐test, there was some amount of heterogeneity in the true outcomes (Q(1)=2.9941, p=0.0836, ${\hat{\tau }}^{2}=0.0411$, $I^{2}=66.6009$%). However, with only two studies moderator analyses were not possible, and tests of publication bias are not valid.

*Effects of inclusive community‐driven development on attitudes and support for women's economic, social and human rights by men, household and family members and community members*

Beath and colleagues' (2013a, 2013b) experimental study in Afghanistan was the only study evaluating the impact of inclusive community‐driven development on attitudes and support for women's economic, social and human rights by men, household and family members and community members. Their report included four effects that fell into this outcome category (e.g., men accept women seeking national office). The effects ranged from very small, negative point estimates (*g *=* *−0.00, [95% CI: −0.08 to 0.07]) to very small, positive point estimates (*g *=* *0.03, [95% CI: −0.04 to 0.10]) We assessed the study as having low risk of bias.

*Effects of inclusive community‐driven development on safer and more secure households, communities and areas/territories for women, girls, men and boys*

**Figure 62:**
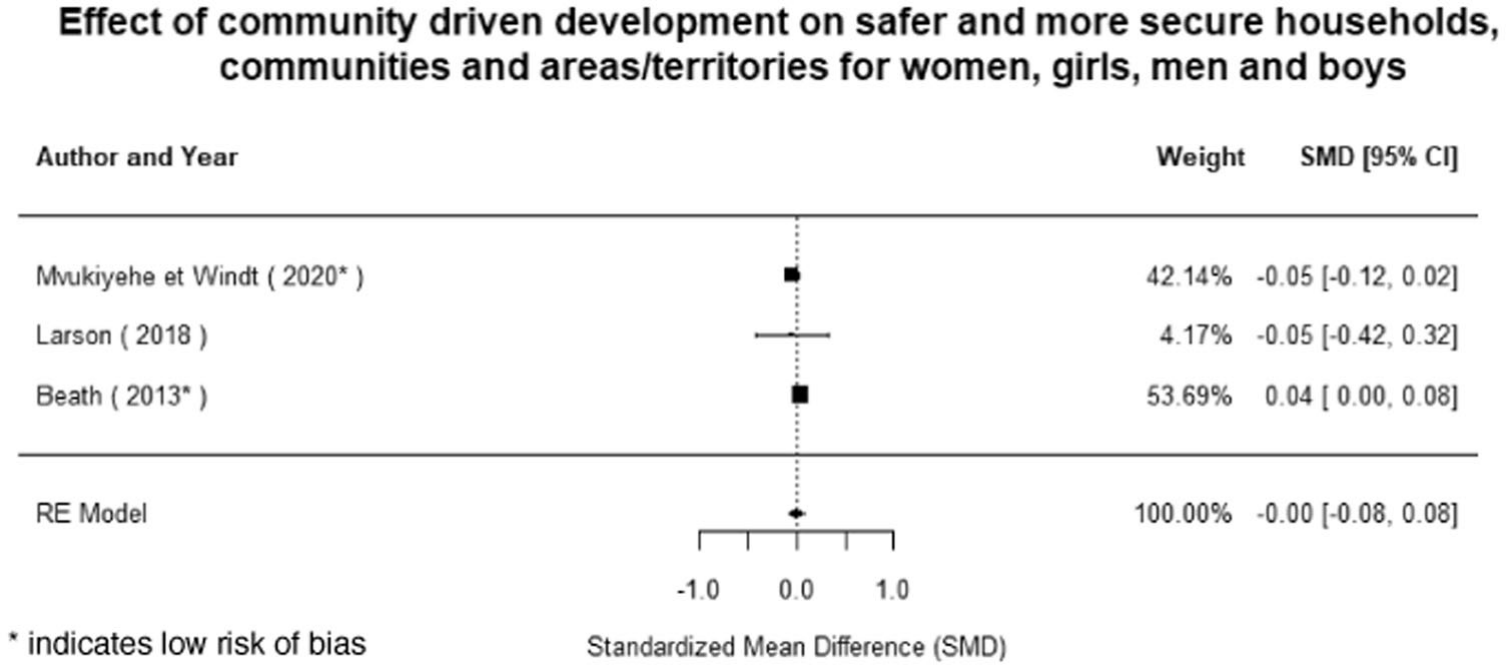
CDDCB6: Forest plot showing the observed outcomes and the estimate of the random‐effects model. CI, confidence interval

We included a total of k=3 studies in the analysis. We assessed two studies as low risk of bias, none as some concerns, and one as high risk of bias. The observed outcomes ranged from −0.05 to 0.04. The estimated average outcome based on the random‐effects model was $\hat{\mu }=-0.0003$ (95% CI: −0.08 to 0.08). Therefore, the average outcome did not differ significantly from zero (z=−0.01, p=0.99). A forest plot showing the observed outcomes and the estimate based on the random‐effects model is shown in Figure 62 (CDDCB6).

According to the Q‐test, there was no significant amount of heterogeneity in the true outcomes (Q(3)=5.08, p=0.08, ${\hat{\tau }}^{2}=0.00$, $I^{2}=60.62$%). An examination of the andomized residuals revealed that none of the studies had a value larger than ±2.50 and hence there was no indication of outliers in the context of this model. According to the Cook's distances, none of the studies could be considered to be overly influential. With only three studies, moderator analyses were not appropriate.

*Effects of inclusive community‐driven development on quality of relationships between women and their household and community members*

Beath and colleagues’ (2013a, 2013b) experimental study in Afghanistan and Mvukiyehe and van der Windt (2020 experimental study in Democratic Republic of Congo were the only studies evaluating the impact of inclusive community‐driven development on improved quality of relationships between women and their household and community members. While there are enough studies here, the outcomes do not appear to be similar enough to combine in a meta‐analysis.

Beath and colleagues (2013a, 2013b) included three effects that fell into this outcome category (e.g., dispute last year). The effects ranged from small, negative effects (*g *=* *−0.17, 95% CI [−0.37, 0.03]) to very small, positive effects (*g *=* *0.08, 95% CI [−0.06, 0.21]). We assessed the study as having low risk of bias.

Mvukiyehe and van der Windt (2020 included two effects that fell into this outcome category (intra‐village trust in village member and intra‐village trust in chief). Both effects were very small, negative and not statistically significant (*g *=* *−0.01, 95% CI [−0.11, 0.10]), and (*g *=* *−0.06, 95% CI [−0.17, 0.05]), and we assessed the study as having low risk of bias.

*Qualitative findings*

We conducted a thematic synthesis on the six linked qualitative studies to the included inclusive community‐driven development interventions. As indicated above, this thematic synthesis aims to identify themes related to the interplay of intervention design, intervention implementation, target population, and contextual variables with intervention outcomes and effects. In total, we identified 27 descriptive themes, which we configured into six analytical themes (Supporting Information Appendix SN). These six analytical themes present the synthesis results and are discussed in more detail below.

*Theme 1: Socioeconomic characteristics affect participation whilst gender‐balanced participation in committees, particularly women's representation, can determine the potential benefits derived from CDD projects*.

Women's education and literacy level, farmland ownership and residential proximity to the project (e.g., road networks) affected participation and benefits from CDD projects. Involvement and/or representation of women in project implementation committees may not only heighten community members’ feeling of ownership, self‐value and respect, and reliability on government, but also support changes in cultural and gender norms (e.g., involvement in girls' school development projects brought about positive changes in community members' attitude towards girls' education [Komorowska, 2016]).

Successful implementation and sustainability of CDD projects can be facilitated by men and women's cooperation from the ground up. Bottom‐up initiatives can be successful even when existing cultural norms are sustained. Mobilisation and awareness raising among community members can enhance ownership of community projects. Women's participation in decision making processes was central to ensuring that women's development needs were captured and protected as a woman reflected: ‘In the past, we fetched water from the river but now there is no water in the river, we go in the night to wells far away for water, we take turns and sometimes wait for three or four hours before we can get the water' (Boesen, 2004).

Equal opportunity, access, contribution, participation and representation of men and women were also important for smooth implementation. Humphreys et al. (2012) reported men's dominance in access to information, contribution to and use of the projects. Projects and committees that are developed without constructive involvement of the community members and formed just for the sake of the intervention, may have a negative impact on the community members as they may see it as forcefully imposed. Hence, mobilising and involving community members meaningfully and in a timely manner was crucial for CDD projects.

*Theme 2: Capacity and legitimacy of the project committee members are crucial to the delivery of the projects*.

Members of the project committees can play vital roles throughout the life cycle of inclusive CDD projects. To represent and empower women through these interventions, it was found to be crucial that the composition of the project committees ensure gender parity. In many cases, gender parity was mandated by design. Although Humphreys and colleagues (2012) found a certain level of women's representation in the committees even without such mandated parity,, they explained that committees designed with mandated gender parity tend to have a large number of women. Essentially, committee members’ knowledge of project design, resource management, operational and logistics management for implementation, and monitoring and evaluation were important for sound implementation of the projects. Barakat and Strand (2006) reported that members of the majority of the project committees that they assessed in their study required training.

Legitimacy of the committee reflected its authority, peoples’ trustworthiness and cooperation with relevant government and nongovernmental agencies. Boesen (2004) reported that: ‘In many districts, the CDC was already perceived as an established and legitimate institution in a wider context than NSP. This was due to two reasons. One reason was that the CDC is a structure which is elected by the whole community on a democratic basis without direct deference to traditional local powerholders. Furthermore, the formal andomizediz of the CDC by the government through (the) Ministry for Rural Rehabilitation and Development (MRRD), and the official stamp and registration in the provincial MRRD office, was perceived as essential with regard to the legitimacy of the CDC in the communities’.

*Theme 3: Timely disbursements of funds and proper human resource management system of government personnel is essential to the successful completion of CDD projects*.

Successful completion and sustainability of inclusive CDD projects critically hinge on timely implementation, regular payment and funds release, management of human resources and maintenance of physical resources. Delayed and faulty implementation and approval processes were often identified as major challenges. Barakat and Strand (2006) noticed delays in payment and fund release which resulted in delay in procurement of critical goods required to implement the projects. Such delays often cause tension between community/committee members and corresponding funding bodies.

Timely implementation of critical project components was also mentioned as a crucial factor for smooth delivery of planned projects. For instance, community andomizedi and awareness raising were reported to be critical for encouraging participation and it was essential that these activities were carried out at the onset of the project. Emphasising on the importance of timing, Barakat and Strand (2006) reported that: ‘Most seriously, the delays were blamed for damage caused to the quality of projects; for example, where the building of infrastructure could not be completed before winter’.

Timing of intervention targeting women was also key to its delivery and success. When interventions coincide with annual harvesting seasons, participation of the community and especially of women were reportedly near impossible (Laudati et al., 2018). These kinds of interventions must be well planned and timed to ensure their effectiveness in reaching the target populations.

Bhatia et al. (2018) also pointed out that it is important to ensure that all sections of the community can equally benefit from the project. When project funds are restricted to cover only a segment of the affected community, rivalry and grievances arise that may challenge the delivery and success of the projects.

Proper human resource management, defined responsibilities and workload distribution were reported to be essential for smooth operation. Overburdening staff with workload and engaging project staff in out‐of‐project activities because of their expertise can hinder implementation. Adequate allocation and maintenance of physical resources were also important for smooth implementation and sustainability of the projects.

*Theme 4: Distrust, power dynamics and frictions, corruption and nepotism can undermine communities’ involvement and support for the project*.

Communities demonstrated overwhelming preference for funded community projects to be managed by external bodies rather than local authorities or traditional systems. Corruption and nepotism were major factors fuelling peoples’ lack of trust. For instance, a participant complained that: ‘We now have less qualified teachers because those who were qualified were chased by the current schoolmaster; instead, he hired the ones according to his affinity/kinship relations with them and some students left the school for other schools that are far from here. As a result, the overall quality of the teaching has reduced’. Another participant reported that, ‘Rather than this [reluctance to use] being a result of a badly built centre, it was found that villagers had stopped attending the Tuungane 1 facility because the head of the health care centre was reportedly charging patients as much as five times more than neighbouring clinics’ (Laudati et al., 2018).

Against their reputation for corruption and nepotism, the qualitative findings suggested that external management was perceived to better empower the populations, improve efficiency and reduce corruption. The experience of the community with failed government promises and corrupt practices showed in form of reluctance towards the new programme or intervention. Sometimes, directors of newly provided facilities and services exhibited the same level of corruption and nepotism, which denied beneficiary communities the intended quality, access, use and control of the facilities/services. However, good governance and project management can also improve public trust and reliability towards the government. A staff member expressed that: ‘The prestige projects, the ones that are better built, have a huge psychological effect on the community. Having a proper school. A proper health centre. To me it has an impact on how the infrastructure is maintained after the programme leaves, and on how the programme is evaluated by the communities’ (Laudati et al., 2018).

Power dynamics within the community can affect the participation of different stakeholders especially if they affect traditional governance structures. Laudati and colleagues (2018) reported that power dynamics is ‘important for the level of overall participation and engagement in the project by different actors, inclusion in the decision‐making process regarding project identification, and as discussed here, the ability of different beneficiaries to access and have control over facility services’. Rivalry among different religious groups (e.g., Protestant and Catholic Churches), as well as intervillage tensions—resulting from their placement in relation to the intervention—can hamper the delivery and success of the intervention (Humphreys et al., 2012).

Some qualitative findings highlighted the challenges arising from weak institutional infrastructure linked to rivalry among different government ministries. Bureaucracy and administrative bottlenecks (e.g., the problem of high staff turnover; the existence of too many forms; constant changes to the operational manual or rules of business) and rigid mindset regarding how things should be done also pose serious challenges (Barakat & Strand, 2006). Collaboration, cooperation and coordination between relevant public and private agencies are crucial as well.

Programme planners and implementers need to take the issue of strategic involvement of the established community system into critical consideration. It was reported that if inclusive CDD projects potentially affect vested interests of local influential individuals or groups, they may position themselves within the project committees and thereby reorient the projects in their favour (Komorowska, 2016). Nevertheless, the study also mentioned that recently a positive change could be noticed where influential elders were giving up powers to the committee or acting as watchdogs to oversee the implementation process.

Financial irregularities, leakage or incidences of larceny such as robbery of theft can also limit success of the projects. For the Tuungane 1 project in DRC, a chief who mentioned: ‘the project hasn't been well executed because of robbery and mismanagement’ (Laudati et al., 2018).

*Theme 5: Insecurity and vested influence of powerful individuals can hamper project implementation*.

Several external factors pose serious threats to ICDD projects. Security is crucial for the success of development interventions. Security issues range from low‐intensity conflicts to civil war, sensitivity around poppy industries (Barakat & Strand, 2006), resistance from insurgent groups and violence against women (Bhatia et al., 2018). Where insecurity prevails, intervention staff can be endangered, the community is scared to participate, provided facilities and services are hijacked by the insurgents, and the use and sustainability of the project/facility/services provided is at risk. Reported that respondents mentioned: ‘CDCs were specifically targeted by insurgent groups as part of their campaign against the government. CDC members reported that they were warned to stop their work by the Taliban, and that they were afraid of being associated with the CDC’, while another participant said, ‘A more commonly mentioned reason in other communities was that, as insecurity rose, government agencies and NGOs withdrew from the area, and there were no projects to implement and therefore no reason for CDCs to continue with meetings’ (Bhatia et al., 2018).

In many cases, the government was unable to tackle these insurgencies. Some participants reported that despite being occupied by insurgents, some communities were able to continue the project by actively collaborating with them. In addition, projects can undermine vested interests of local influential persons who can cause hindrance to implementation. External influence on committee elections can also have negative impacts. Technical issues external to project design and implementation (e.g., seasonality, depth of groundwater level, geographic isolation, drought or other environmental stressors) can affect implementation (Laudati et al., 2018).

*Theme 6: Gender and social norms deeply rooted in cultural beliefs and practices are rigid. This applies particularly if changes in such norms and beliefs undermine the authority of influential individuals or groups and hinder implementation*.

Even when women are afforded BCC, safe spaces, and income‐generating initiatives, their level of participation and therefore their potential benefit may be constrained by such norms. In Afghanistan, qualitative findings from the NSP identified conservative religious beliefs and core values or norms about women's mobility and participation in community affairs as key obstacles limiting women's voting rights, membership in committees, and holding decision making positions (Bhatia et al., 2018). Furthermore, programme evaluators acknowledge that positive changes in norms are usually protracted. One NSP woman complained that: ‘Men stop you if you start talking about women's problems, they say that women should be quiet’ (Boesen, 2004).

NSP activities typically involved the participation of women in income generating activities of home‐based products (for instance, tailoring, handicrafts, carpet weaving). Due to the low marketing potential of these products and women's limited access to markets due to cultural restrictions, women could not make enough profit from such activities (Boesen, 2004). The involvement of women and girls in projects aimed to improve women's or girls’ access to education or health services and could activate changes in traditional norms and beliefs (Komorowska, 2016). In the context of Afghanistan's NSP, in communities facing the shared post‐war realities, men tend to andomize the importance of women to community reconstruction and development (Boesen, 2004).

The involvement of women in community activities rooted within ICDD interventions may pose a threat to established customary governance systems, vested interests and even religious strongholds. Such activities include the building of important communal structures, water and power supply infrastructure. In particular, leadership opportunities for women in the Tuungane 1 for community‐related development. In the Tuungane 1 programme, traditional norms and beliefs remained an impediment to this possibility in some instances as one woman responded: ‘only men have positions of power’. Another woman felt that: ‘Tuungane did well [for women] as it created a space for a woman to be involved; Tuungane is democratic, it doesn't discriminate and it called for men to understand that women are equally able to make positive change in society’.

In response to rebuttals on such customs and vested interests, it might not be unlikely that influential individuals or groups would try to infiltrate the committees and influence the voting of their elected officials to ensure they retain control (Bhatia et al., 2018). A Tuungane 1 community member advised that: ‘Tuungane has to monitor the work on the ground constantly to avoid corruption’ while another expressed uncertainty on the status of a building: ‘The CDC project was good but the one we have needed an engineer who let us down; right now, we don't know whether the money has been used or not or why the building is not yet finished’ (Humphreys et al., 2012). Furthermore, communities tend to fear that programme implementers are pretentiously introducing Westernised cultures, intending to erode traditional culture (Boesen, 2004).

##### 5.3.4.10 Discussion

Quantitative meta‐analysis revealed that ICDD interventions had positive significant effects on representation of women in local and subnational civil and political processes. However, effects on community and household level outcomes such as safer and more secure households and communities were nonsignificant. The intervention group was covered by three programmes and the associated GRADE analysis showed high confidence in the finding related representation of women in civil and political processes and low confidence in the community and household findings.

A complementary qualitative synthesis was carried out to put forward a set of recommendations that may help design more effective ICDD interventions that can support the translation of political representation effects on community and household level outcomes targeting broader empowerment objectives.

- From the qualitative evidence, a number of implementation themes emerged which shed some light on the challenges of translating representation outcomes into more effective project government and implementation. Even when interventions succeeded in enhancing the representation of women on project committees and other decision‐making structures, capacity gaps across governance structures impeded project implementation and delivery.
- In addition, the composition of committees and representatives on decision‐makers structures mattered largely for the legitimacies of these bodies within the communities targeted by the intervention. While interventions reported progress in ensuring a greater representation of women on these bodies, qualitative evidence referenced challenges in ensuring background diversity of these women. That is, socioeconomic characteristics such as class, education level, profession emerged as important for the perceived legitimacy of governance bodies and decision‐makers within the communities. Projects often struggled to ensure this additional level of diversity.
- Taken together, governance bodies’ relative lack of capacity and legitimacy emerged as two possible explanations as to why ICDD interventions achieved less success at the household and community level.
- Furthermore, qualitative evidence pointed to a complex navigation of competing governance systems within communities as an additional explanatory lens why intervention effects were not observed equally at household and community level. That is, in most target communities, governance structures introduced by ICDD coexist with traditional governance authorities (e.g., local chiefs) as well as local government representatives (e.g., elected district officials). Navigating this coexistence required a careful balancing of politics and power relations, which limited the space for project implementation and reach in some instances.
- All of the themes above, that is, governance legitimacy, capacity, and coexistence, presented entry points for powerful elites and vested interests to undermine project implementation and impact; with an often‐observed alignment and intersection of these actors’ agenda with restrictive social norms and preserving existing power imbalances between men and women.
- In terms of intervention design and implementation recommendation, the above qualitative findings and explanatory lenses suggest that investment in ‘good governance’ for ICDD projects is important, not overlooking more granular project management capacities. Likewise, intervention design should anticipate conflicts to emerge where multiple governance systems coexist and to outline proactively mitigation strategies and a space and contribution of the new ICDD‐introduced structures within the communities.

##### 5.3.4.11 Summary of findings and discussion

We included three programmes in eight countries in Latin America, East Asia and Pacific, South Asia and Sub‐Saharan Africa that evaluated the effect of inclusive community‐driven development in LMICs. We were able to examine effects on the following: women have increased freedom of movement and association, increased representation of women in local and subnational civil and political processes, including during peacebuilding and post conflict restoration, women have improved attitudes, self‐image and confidence, safer and more secure household, communities and areas/territories for women, girls, men and boys. Our included studies report against all three secondary outcomes (Resources, Agency and Achievement) and six of the nine immediate outcomes. Overall, the GRADE assessments (Table 27) generally indicate a range of very low to high certainty in this body of evidence.

Six qualitative analytical themes buttressed the quantitative findings by illuminating the importance of andomize capacity building, andomized power dynamics and ensuring particular benefits from projects by balancing representing in committees and potential buy‐in by powerholders. With positive and significant impact on women's representation outcomes, ICDD interventions may be able to promote a greater role for women in decision‐making as well as deciding key outcomes for their communities. While other outcomes relating to secure households were not significant, the emphasis on women's participation shows the importance of planning ICDD interventions that allow women to have their voices and opinions heard (Table 22).

**Table 22:** GRADE summary of findings and certainty of evidence on inclusive community‐driven development

Certainty assessment										Sample size	Effect		Certainty	Importance
No. of studies	Study design	Risk of bias	Inconsistency		Indirectness		Imprecision	Other considerations			Absolute (95% CI)			
*(AA1) Women have access to rights, services and opportunities*														
1	RCT	Not serious	Serious^a^		Not serious		Not serious	None		4307	One negative and four positive effect estimates with a 95% CI range of −0.09 to 0.19		⊕⊕⊕◯ MODERATE	Limited importance
*(AB2) Women have increased access to and ownership of assets, credit and income*														
1	RCT	Not serious	Serious^a^		Not serious		Not serious	None		8032	Two very small positive and two very small negative effect sizes with a 95% CI range of −0.07 to 0.20		⊕⊕⊕◯ MODERATE	Limited importance
*(BA2) Women have more and better control over their bodies and sexual health*														
1	RCT	Not serious	Serious		Not serious		Not serious	None		8032	One negative and one zero effect estimates with a 95% CI range of −0.06 to 0.06		⊕⊕⊕◯ MODERATE	Critical
*(BA3) Women have increased freedom of movement and association*														
2	RCT‐2	Not serious	Not serious		Not serious		Serious^b^	None		9134	SMD 0.00 SD (−0.03 lower to 0.04 higher)		⊕⊕⊕◯ MODERATE	Limited importance
*(BA5) women have more positive attitude towards taking action to claim their rights*														
2	RCT‐2	Not serious	Not serious		Not serious		Serious^c^	None		900	SMD 0.03 SD higher (0.1 lower to 0.17 higher)		⊕⊕⊕◯ MODERATE	Limited importance
*(BA6) Reduced percentage of women agreeing with certain reasons that justify violence against women and girls*														
2	RCT‐2	Not serious	Serious^d^		Not serious		Serious^e^	None		6751	SMD 0.01 SD lower (0.1 lower to 0.09 higher)		⊕⊕◯◯ LOW	Critical
*(BA7) Women are equipped with better life skills that allow them to be prepared for crisis or shocks and recover from them*														
1	RCT	Not serious	Serious^a^		Not serious		Serious^f^	None		4931	One negative and two positive effect estimates with a 95% CI range of −0.06 to 0.20		⊕⊕◯◯ LOW	Important, but not critical
*(BB1) Women participate more in their community*														
1	RCT	Not serious	Serious^a^		Not serious		Not serious	None		916	Six negative and two positive effect estimates with a 95% CI range of −0.11 to 0.11		⊕⊕⊕◯ MODERATE	Limited importance
*(BB2) Women participate more in their community*														
1	RCT	Not serious	Serious^a^		Not serious		Not serious	None		916	Two negative effect estimates of −0.05 with a 95% CI range of −0.43 to 0.33		⊕⊕⊕◯MODERATE	Limited importance
*(BB4) Women increasingly form their own organisations*														
1	RCT	Serious^g^	Serious^a^		Not serious		Serious^h^	Strong association		424	Three positive (two large) effect estimates with a 95% CI range of 0.13 to 1.08		⊕⊕◯◯LOW	Limited importance
*(CA1) Power holders have improved awareness and responsiveness to the demands, claims, rights and inputs of women*														
1	RCT	Not serious	Serious^a^		Not serious		Not serious	None		8021	SMD 0.03 SD higher (0.01 lower to 0.08 higher)		⊕⊕⊕◯MODERATE	Limited importance
*(CA2) Representation of women in local and subnational civil and political processes (assessed with: self‐reports)*														
2	RCT	Not serious	Not serious		Not serious		Not serious	Strong association		2319	SMD 0.28 SD higher (0.13 higher to 0.43 higher)		⊕⊕⊕⊕HIGH	Limited importance
*(CA3) Institutional mechanisms and structures monitor human rights violations and ensure the security, safety and health of women and girls*														
1	RCT	Not serious	Serious^a^		Not serious		Not serious	None		8032	SMD 0.01 SD higher (0.03 lower to 0.05 higher)		⊕⊕⊕◯MODERATE	Critical
*(CA4) Increased attention and focus on the needs and priorities of women and girls, and other vulnerable groups during relief and recovery in conflict and post‐conflict settings*														
1	RCT	Not serious	Serious^a^		Not serious		Not serious	None		4095	Two positive effect estimates with a 95% CI range of 0.02 to 0.19		⊕⊕⊕◯MODERATE	Limited importance
*(CA5) Increased representation of women at all levels as decision‐makers in post‐conflict countries*														
1	RCT	Not serious	Serious^a^		Not serious		Not serious	None		4308	Four positive effect estimates with a 95% CI range of −0.02 to 0.16		⊕⊕⊕◯MODERATE	Limited importance
*(CA8) Community support for women's and children's human, economic and legal rights*														
4	RCT‐4	Not serious	Not serious		Not serious		Not serious	None		15,674	SMD 0.01 SD lower (0.1 lower to 0.08 higher)		⊕⊕⊕⊕HIGH	Important, but not critical
*(CA11) CSOs are more inclusive to all genders*														
1	RCT	Serious^i^	Serious^a^		Not serious		Not serious	None		4643	Two positive effect estimates with a 95% CI range of 0.03 to 0.14		⊕⊕◯◯LOW	Limited importance
*(CB3) Women have improved attitudes, self‐image and confidence*														
2	RCT‐1 QED‐1	Very serious^j^	Serious^k^		Not serious		Serious^l^	None		891	SMD 0.1 SD lower (0.43 lower to 0.24 higher)		⊕◯◯◯VERY LOW	Limited importance
*(CB4) Improved attitudes and increased support for women's economic, social and human rights by men, household and family members and community members*														
1	RCT	Not serious	Serious^a^		Not serious		Not serious	None		8968	One null and three positive effect estimates with a 95% CI range of −0.08 to 0.10		⊕⊕⊕◯MODERATE	Limited importance
*(CB6) Safer and more secure household, communities and areas/territories for women, girls, men and boys*														
3	RCT‐2QED‐1	Serious^m^	Serious^n^		Not serious		Not serious	None		13,020	SMD −0.00 SD higher (0.08 lower to 0.08 higher)		⊕⊕◯◯ LOW	Critical
*(CB8) Improved quality of relationships between women and their households and communities*														
1	RCT	Not serious	Serious^a^	Serious		Not serious		None	916			Two negative effect estimates with a 95% CI range of −0.33 to 0.21	⊕⊕⊕◯MODERATE	Limited importance

Abbreviations: CI, confidence interval; GRADE, Grading of Recommendations, Assessment, Development and Evaluations; QED, quasi‐experimental design; RCT, andomized controlled trial; RoB, risk of bias; SMD, andomizedi mean difference.

^a^ All single studies downgraded once for inconsistency.

^b^ (a) Rate down if confidence intervals of RE model cross the threshold: Yes. (b) Rate down if one study: No, three studies. (c) Sample size: Large, not rated down.

^c^ (a) Rate down if confidence intervals of RE model cross the threshold: Yes. (b) Rate down if one study: No, two studies. (c) Sample size: Large enough, not rated down.

^d^ (a) Wide variance of point estimates across studies. (b) Minimal overlap of Cis, which suggests variation may be more than what one would expect by chance alone. (c) Statistical criteria, including tests of heterogeneity which test the null hypothesis that all studies have the same underlying magnitude of effect, have a low *p*‐value (*p *<* *0.05), indicating to reject the null hypothesis.

^e^ (a) Rate down if confidence intervals of RE model cross the threshold: Yes. (b) Rate down if one study: No, two studies. (c) Sample size: Large, not rated down.

^f^ Downgraded once because of a wide range of Cis that cross both sides of the threshold of interest.

^g^ Downgraded once due to uncertainty related to selection bias, and some risk related to other criteria.

^h^ While all effect estimates are quite large, there is a discrepancy in one estimate against the other two of more than 100%.

^i^ Downgraded due to issues related to deviation from intended intervention and uncertainty with respect to selection bias.

^j^ Downgraded twice because 37.44% of weight (or half of the studies) is coming from a high RoB QED, and when the high RoB study is removed, there is only one study remaining and the effect estimate would change considerably.

^k^ Downgraded because the point estimates are on either side of the threshold. While the Cis do overlap, there is a considerable difference between the actual point estimates.

^l^ Downgraded once on imprecision to reflect the large CI on the RE model point estimate.

^m^ This analysis has been downgraded because one of the four studies presents a high RoB due to reporting bias, outcome measurement bias, and selection bias. All other studies are low risk of bias.

^n^ Downgraded because some studies present positive, while others negative effect sizes. $I^{2}=41.62\%$.

*Effects of community‐based services on women's access and ownership to assets, credit and income*

Nandi and colleagues’ (2020) experimental study in India was the only study evaluating the impact of community‐based services on women's access and ownership to assets, credit and income. Their report included two effects that fell into this outcome category (paid in cash and any time on paid work). Both point estimates were very small, positive and not statistically significant (*g *=* *0.02, [95% CI: −0.05 to 0.09]. We assessed the study as having some risk of bias concerns.

#### 5.3.5 Institutional provision of loans and savings

Institutional provision of loans and savings includes the provision of loans, credit and savings by a formal financial institution or microcredit programme (World Bank, 2021a, 2021b).

##### 5.3.5.1 How do institutional provision of loans and savings affect gender equality, women's empowerment and Peace outcomes?

**Figure 63:**
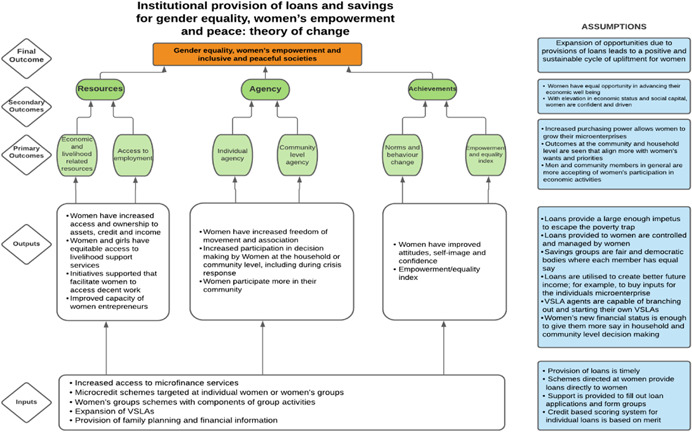
Institutional provision of loans and savings for gender equality, women's empowerment and peace: Theory of change

Figure 63 maps out the causal chain of how the institutional provision of loans and savings may improve gender equality, women's empowerment and peace outcomes. Loans and savings schemes (primarily microcredit schemes) improve women's access to assets, credit and income; hence, expanding the economic resources available to women. At the same time, many schemes offer financial or business training that increase their capacity to interact with these resources. Consequently, women's economic status is improved which simultaneously leads to increased agency, more decision‐making ability, improved autonomy and higher empowerment. Moreover, schemes that employ group models give women a *de facto* network of peers thereby increasing their social capital and hence, their empowerment. Therefore, there is a shift towards a more equitable and inclusive society.

##### 5.3.5.2 Description of included studies

We included seven studies from nine different papers that evaluated the effect of three programmes.

*Population*

Studies included andomi on low‐income women. Some studies extended loans to men as well; however, there was a particular emphasis on enhancing poor women's access to cash.

The included studies evaluated two different programmes and trials in South Asia and Sub‐Saharan Africa in five countries: Ethiopia, India, Liberia, Nigeria and Pakistan.

*Intervention, inputs and activities*

The included studies evaluated a range of different loans and savings activities and inputs including:

- Business loans (*n* = 1): Offers microfinance loans to businesswomen with higher loans to those at higher loan cycles.
- Microfinance lending (*n* = 1): Lends microcredit to women entrepreneurs.
- Microfinance scheme for individuals (*n* = 2): Provide microcredit loans directly to individuals based on some merit‐based credit scoring system.
- Group‐based lending (*n* = 3): Microfinance scheme that is contingent on women forming groups that share the liability of the loans.
- Training and activities (*n* = 2): Provision of financial training alongside loans. Additionally, some programmes provide information on family planning and some conduct activities within loan groups (Table 23).

**Table 23:** Institutional ision of loans and savings activities features of included studies

Study	Activity/input	Length of treatment	Intervention frequency
Banerjee et al. (2015)	Spandana is a microfinance product targeted towards low‐income women that form or participate in loan groups	18 months	Weekly
Duflo et al. (2013)	Spandana is a microfinance product targeted towards low‐income women that form or participate in loan groups	12 months	‐
Ifelunini and Wosowei (2012)	Increased access to microfinance lending	‐	‐
Olajide et al., (2016)	Microcredit scheme targeting women with no requirement of group formation/participation	4 months	‐
Johnson (2018)	Microcredit loan offered by BRAC to women who participate in groups. Group activities also conducted by BRAC	Varies among participants. Average engagement being 10 years	‐
Tarozzi et al. (2013)	Provision of information on family planning and financial self‐help groups	36 months	‐
Tarozzi et al. (2015)	Provision of information on family planning and financial self‐help groups	36 months	‐
Weber and Ahmad (2014)	Increased access to microfinance for women	‐	‐

Abbreviation: BRAC, Bangladesh Rural Advancement Committee.

*Comparison*

All our included studies compared treated groups to comparison groups receiving no intervention. One study included multiple treatment arms.

*Outcomes*

The included studies reported on a number of relevant outcomes, including:

- Women have increased access and ownership to assets, credit and income (*n* = 4): Women are able to apply for, receive and manage assets/credit and income and have support to manage, claim and execute their assets without pressure or influence from external actors, including male family members, husbands and cultural leaders.
- Improved capacity of women entrepreneurs (*n* = 2): Capacity can include both technical abilities and resources to achieve a goal. Through skills training, microgrants, and linkage of services, women entrepreneurs are able to pursue their businesses and other pursuits.
- Empowerment/Equality Index (*n* = 2): Indices using multiple outcomes of the list to aggregate them into a gender equality and/or women's empowerment score

The division of the immediate and secondary outcomes is reported in Table 29 (Table 24).

**Table 24:** Institutional provision of loans: Secondary and immediate outcomes

Secondary outcome category	Immediate outcome	Number of studies
Resources material, human and social resources which serve to enhance the ability to exercise choice	Access to justice and legal services	0
	Economic and livelihood related resources	4
	Access to employment	2
Agency ability to define one's goals and act upon them and andomizedized decision‐making	Individual agency	2
	Community level agency	2
	Institutions supporting agency	0
Achievement ways of being and doing which can be andomiz by different individuals	Improved systems	1
	Norms and behaviour change	1
	Empowerment index	2

*Study design*

**Figure 64:**
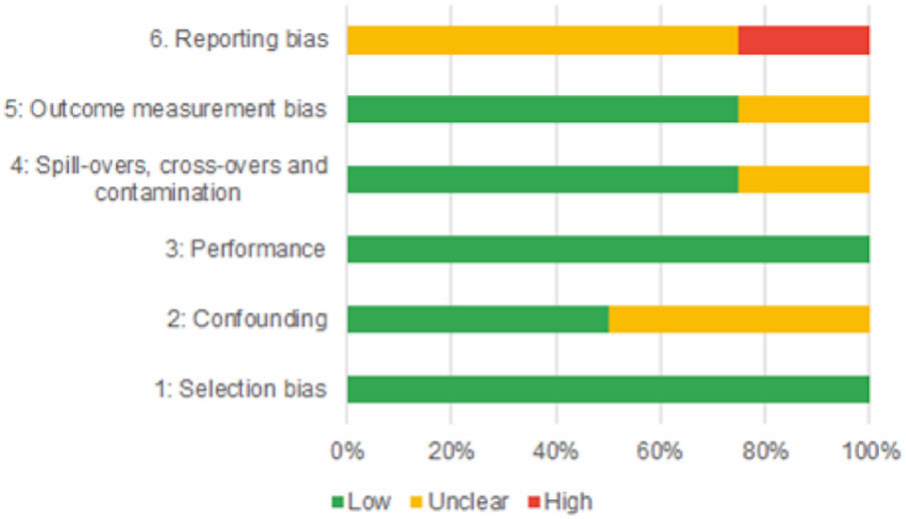
Institutional loans and savings studies quasi‐experimental design risk of bias assessment

Four institutional provision of loans and savings studies used a QED (Ifelunini & Wosowei, 2012; Olajide et al., 2016; Johnson, 2018; Weber & Ahmad, 2014). Only one of these studies was assessed as having a high risk of bias, the rest had some concerns related to risk of bias. As detailed in Figure 64, the domain in which the high risk was identified was reporting bias, this was because the authors did not provide enough information on the matching process and did not report the results for one of the outcomes because of not finding a significant impact (Weber & Ahmad, 2014). A common issue to the rest of QED studies evaluating institutional provision of loans and savings interventions was that authors did not report robustness checks, for which we assessed their reporting bias as unclear. Further, we identified the insufficiency of details provided on characteristics and cluster controls as being unclear whether confounding was an issue or not for two studies (Johnson, 2018; Weber & Ahmad, 2014). We did not identify any limitation related to performance or selection bias.

**Figure 65:**
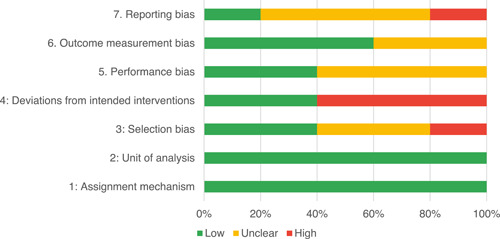
Institutional loans and savings andomized controlled trial risk of bias assessment

Five of the studies evaluating provision of loans and savings used an experimental design (Duflo et al., 2013; Tarozzi et al., 2013). One of the studies had a high risk of bias, and the other presented some concerns related to risk of bias. As detailed in Figure 65, the high risk of bias was identified in the deviations from intended interventions domain. The issue in this regard was a potential contamination from other microfinance institutions starting operations in treatment and control groups (Duflo et al., 2013). It was also unclear whether both studies were free from reporting bias since authors do not provide a pre‐analysis plan and not enough information was provided to verify that all intended analysis and outcome variables were reported. Finally, Tarozzi and colleagues (2013) did not provide enough information regarding attrition, so we assessed selection bias as unclear. We did not identify any other limitation related to assignment mechanism, unit of analysis, performance and outcome measurement.

*Qualitative studies, process evaluations and project documents*

We identified no linked qualitative studies and were unable to conduct a qualitative evidence synthesis related to savings and loans interventions.

##### 5.3.5.3 Synthesis of findings

The following subsection presents the results of effectiveness of institutional loans and savings on gender equality, women's empowerment and peace outcomes.

*Quantitative findings*

*Effects of institutional provision of loans and savings on increased capacity of women to understand and use financial, banking and business services effectively*

Olajide et al., (2016) quasi‐experimental study in Nigeria was the only study evaluating the impact of institutional provision of loans and savings on increased capacity of women to understand and use financial, banking, and business services effectively. There was a large positive and statistically significant effect (*g *=* *0.49, [95% CI: 0.41 to 0.57]). We assessed the study as having some risk of bias concerns.

*Effects of institutional provision of loans and savings on women having increased access and ownership to assets, credit and income*

**Figure 66:**
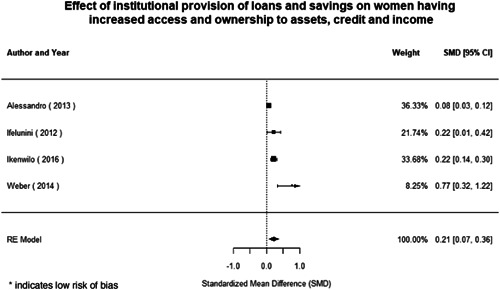
LSAB2: Forest plot showing the observed outcomes and the estimate of the random‐effects model. CI, confidence interval

We included a total of k=4 studies in the analysis. We assessed none of the studies as low risk of bias, three as some concerns and one as high risk of bias. The observed outcomes ranged from 0.08 to 0.77. The estimated average outcome based on the random‐effects model was $\hat{\mu }=0.21$ (95% CI: 0.07 to 0.36). Therefore, the average outcome differed significantly from zero (z=2.84, p<0.01). A forest plot showing the observed outcomes and the estimate based on the random‐effects model is shown in Figure 66 (LSAB2).

According to the Q‐test, the true outcomes appear to be heterogeneous (Q(3)=17.45, p<0.01, ${\hat{\tau }}^{2}=0.01$, $I^{2}=82.80$%).

An examination of the studentised residuals revealed that none of the studies had a value larger than ±2.50 and hence there was no indication of outliers in the context of this model. According to the Cook's distances, none of the studies could be considered to be overly influential.

We attempted to test all potential moderators, but several could not be tested because all but one study belonged to a single moderator group (i.e., risk of bias, study design, post‐intervention vs. change from baseline). Of the remaining moderators (publication year, whether the model was adjusted for covariates, exposure to intervention, evaluation period), none were significant predictors of heterogeneity.

*Effects of institutional provision of loans and savings on women's engagement in other micro, small and medium‐sized enterprises*

Banerjee et al. (2015), an experimental study in India, was the only study evaluating the impact of institutional provision of loans and savings on women's engagement in other micro, small and medium‐sized enterprises. The effect was very small and but positive and statistically significant (*g *=* *0.05, [95% CI: 0.02 to 0.08]). We assessed the study as having high risk of bias concerns.

*Effects of institutional provision of loans and savings on initiatives supported that facilitate women to access decent work (formal and informal employment)*

Tarozzi et al.'s (2013) experimental study in Ethiopia was the only study evaluating the impact of institutional provision of loans and savings on initiatives supported that facilitate women to access decent work (formal and informal employment), including people with disabilities. Their report included two effects that fell into this outcome category (hours of work per week in self‐employment and outside employment). The point estimates were both very small and not statistically significant, negative for self‐employment (*g *=* *−0.02, [95% CI: −0.07 to 0.02]) and positive for outside employment (*g *=* *0.02, [95% CI: −0.02 to 0.06]). We assessed the study as having some risk of bias concerns.

*Effects of institutional provision of loans and savings on improved capacity of women entrepreneurs*

**Figure 67:**
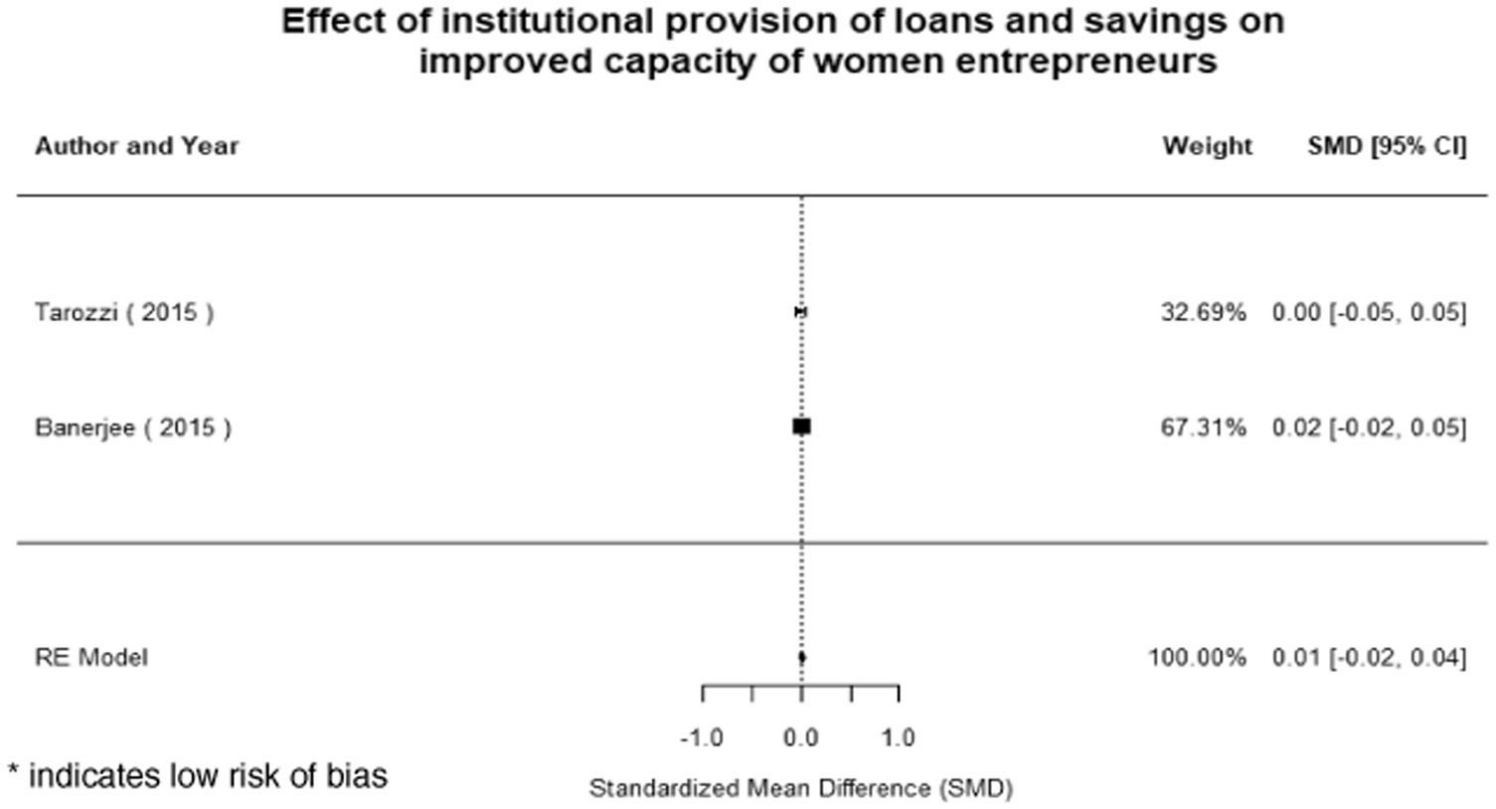
LSAC3: Forest plot showing the observed outcomes and the estimate of the random‐effects model. CI, confidence interval

For capacity of women entrepreneurs, two studies reported disaggregated data, thus we included *k *=* *2 studies in the analysis. We assessed one of the studies as some concerns of risk of bias and the other as high risk of bias. The estimated average outcome based on the random‐effects model was $\hat{\mu }=0.01$ (95% CI: −0.02to0.04). Therefore, the average outcome did not differ significantly from zero (z=0.77, p=0.44). A forest plot showing the observed outcomes and the estimate based on the random‐effects model is shown in Figure 67 (LSAC3). Given the small number of studies, this result should be interpreted with caution. According to the Q‐test, there was no significant amount of heterogeneity in the true outcomes (Q(1)=0.29, p=0.59, ${\hat{\tau }}^{2}=0.00$, $I^{2}=0.00$%). With only two studies, moderator analyses were not possible, and tests of publication bias are not valid.

*Effects of institutional provision of loans and savings on women's freedom of movement and association*

Weber and Ahmad's (2014) quasi‐experimental study in Pakistan was the only study evaluating the impact of institutional provision of loans and savings on women having increased freedom of movement and association. There was a large, and statistically significant, effect (*g *=* *0.53, [95% CI: 0.09 to 0.96]), however we assessed the study as having high risk of bias.

*Effects of institutional provision of loans and savings on women's attitude towards taking action to claim their rights*

Johnson's (2018) quasi‐experimental study in Liberia was the only study evaluating the impact of institutional provision of loans and savings on women having more positive attitude towards taking action to claim their rights. Their report included effects for both individual and group lending technologies. The point estimates were medium, negative but not statistically significant, for individual lending technology (*g *=* *−0.35, [95% CI: −0.85 to 0.16]), and for group lending technology (*g *=* *−0.32, [95% CI: −0.72 to 0.09]), and we assessed the study as having some risk of bias concerns.

*Effects of institutional provision of loans and savings on increased participation in decision making by women at the household or community level, including during crisis response*

**Figure 68:**
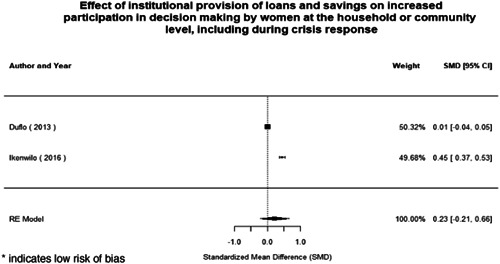
LSBB1: Forest plot showing the observed outcomes and the estimate of the random‐effects model. CI, confidence interval

Two studies reported disaggregated data for increased participation in decision making by women at the household or community level, including during crisis response, thus we included *k *=* *2 studies in the analysis. We assessed one of the studies as some concerns of risk of bias and the other as high risk of bias. The estimated average outcome based on the random‐effects model was $\hat{\mu }=0.23$ (95% CI: −0.21to0.66). Therefore, the average outcome did not differ significantly from zero (z=1.02, p=0.31). A forest plot showing the observed outcomes and the estimate based on the random‐effects model is shown in Figure 68 (LSBB1). Given the small number of studies, this result should be interpreted with caution. According to the Q‐test, there was significant amount of heterogeneity in the true outcomes (Q(1)=81.05, p<0.0001, ${\hat{\tau }}^{2}=0.10$, $I^{2}=98.77$%). With only two studies, moderator analyses were not possible, and tests of publication bias are not valid.

*Effects of institutional provision of loans and savings on representation of women in local and subnational civil and political processes, including during peacebuilding and post conflict restoration*

Johnson's (2018) quasi‐experimental study in Liberia was the only study evaluating the impact of institutional provision of loans and savings on representation of women in local and subnational civil and political processes, including during peacebuilding and post conflict restoration. Their report included effects for both individual and group lending technologies. The effect of individual lending technology intervention was large, negative and statistically significant (*g *=* *−0.70, [95% CI: −1.21 to −0.20]), and the point estimate of group lending technology was also negative but medium, and not statistically significant (*g *=* *−0.36, [95% CI: −0.77 to 0.04]). We assessed the study as having some risk of bias concerns.

*Effects of institutional provision of loans and savings on women's attitudes, self‐image and confidence*

Olajide et al., (2016) quasi‐experimental study in Nigeria was the only study evaluating the impact of institutional provision of loans and savings on women having improved attitudes, self‐image and confidence. There was a large, and statistically significant, effect (*g *=* *0.58, 95% CI [0.50, 0.67]), and we assessed the study as having some risk of bias concerns.

*Effects of institutional provision of loans and savings on women's empowerment index*

**Figure 69:**
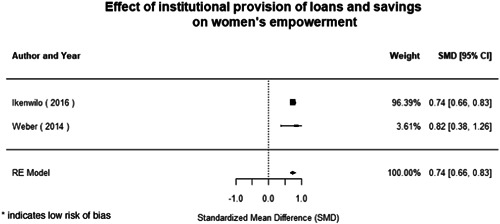
LSCC1: Forest plot showing the observed outcomes and the estimate of the random‐effects model. CI, confidence interval

Two studies reported disaggregated data for women's empowerment index, thus we included k=2 studies in the analysis. We assessed one of the studies as some concerns of risk of bias and the other as high risk of bias. The estimated average outcome based on the random‐effects model was $\hat{\mu }=0.74$ (95% CI: 0.66 to 0.83). Therefore, the average outcome differed significantly from zero (z=17.40, p<0.001). A forest plot showing the observed outcomes and the estimate based on the random‐effects model is shown in Figure 69 (LSCC1). According to the Q‐test, the true outcomes appear to be homogeneous (Q(1)=0.11, p=0.73, ${\hat{\tau }}^{2}=0.00$, $I^{2}=0.00$%). With only two studies, moderator analyses were not possible, and tests of publication bias are not valid.

##### 5.3.5.4 Summary of findings and discussion

We included six studies across five countries in South Asia and Sub‐Saharan Africa that evaluated the effect of institutional provision of loans and savings in LMICs. We found positive effects on women having increased access and ownership to assets, credit and income as well as on women's empowerment. However, the number of studies is small, so the results must be viewed with caution. We were also able to examine improved capacity of women entrepreneurs and increased participation in decision making by women at the household or community level, including during crisis response, but there were no significant impacts. Our included studies reported against all secondary outcomes (Resources, Agency and Achievement) and seven of the nine immediate outcomes. Overall, the GRADE assessments generally indicate a very low to low (with one moderate) certainty in this body of evidence.

We identified no linked qualitative studies and were unable to conduct a qualitative evidence synthesis related to savings and loans. The summary of quantitative evidence along with the GRADE certainly ratings are presented in Table 30 (Table 25).

**Table 25:** GRADE summary of findings and certainty of evidence on institutional provision of loans and savings

Certainty assessment							Sample size	Effect	Certainty	Importance
No. of studies	Study design	Risk of bias	Inconsistency	Indirectness	Imprecision	Other considerations		Absolute (95% CI)		
*(AB1) Increased capacity of women to understand and use financial, banking, and business services effectively*										
1	RCT‐1 QED‐1	Serious^a^	Not serious	Not serious	Serious^b^	None	15,250	One large, positive effect size of 0.49 with a 95% CI of 0.41 to 0.57	⊕⊕◯◯ LOW	Important, but not critical
*(AB2) Women have increased access and ownership to assets, credit and income*										
4	RCT‐1 QED‐3	Very serious^c^	Serious^d^	Not serious	Not serious	Publication bias strongly suspected^e^	15,314	SMD 0.21 SD higher (0.07 higher to 0.36 higher)	⊕◯◯◯ VERY LOW	Important, but not critical
*(AC1) More women engaged in micro, small and medium sized enterprises*										
1	RCT	Very serious^w^	Serious^g^	Not serious	Not serious	None		One positive effect estimate of 0.05 with a 95% CI range of 0.02 to 0.08	⊕◯◯◯ VERY LOW	Limited importance
*(AC3) Improved capacity of women entrepreneurs*										
2	RCT‐2	Very serious^h^	Not serious	Not serious	Not serious	None	12,687	SMD 0.01 SD higher (0.02 lower to 0.04 higher)	⊕⊕◯◯ LOW	Important, but not critical
*(BA3) Women have increased freedom of movement and association*										
1	QED	Very serious^i^	Serious^g^	Not serious	Serious^j^	Strong association	81	SMD 0.53 SD higher (0.09 higher to 0.96 higher)	⊕◯◯◯ VERY LOW	Limited importance
*(BA5) Women have more positive attitude towards taking action to claim their rights*										
1	QED	Serious^k^	Serious^g^	Not serious	Very serious^l^	None	127	Two negative and one positive effect estimate with a 95% CI range of −0.72 to 0.49	⊕◯◯◯ VERY LOW	Limited importance
*(BB1) Increased participation in decision making by Women at the household or community level, including during crisis response*										
2	RCT‐1 QED‐1	Very serious^m^	Very serious^n^	Not serious	Very serious^o^	None	9039	SMD 0.23 SD higher (0.21 lower to 0.66 higher)	⊕◯◯◯ VERY LOW	Limited importance
*(CA2) Increased representation of women in local and subnational civil and political processes, including during peacebuilding and post conflict restoration*										
1	QED	Serious^q^	Serious^g^	Not serious	Very serious^r^	None	127	Two negative and one positive effect estimate with a 95% CI range of −0.77 to 0.49	⊕◯◯◯ VERY LOW	Limited importance
*(CB3) Women have improved attitudes, self‐image and confidence*										
1	QED	Serious^s^	Serious^g^	Not serious	Not serious	Strong association	2184	SMD 0.58 SD higher (0.5 higher to 0.67 higher)	⊕⊕⊕◯ MODERATE	Limited importance
*(CC1) Empowerment/Equality Index*										
2	QED‐2	Very serious^t^	Serious^u^	Not serious	Serious^v^	Strong association	15,323	SMD 0.74 SD higher (0.66 lower to 0.83 higher)	⊕◯◯◯ VERY LOW	Important, but not critical

Abbreviations: CI, confidence interval; GRADE, Grading of Recommendations, Assessment, Development and Evaluations; QED, quasi‐experimental design; RCT, ealizeda controlled trial; SMD, ealizedali mean difference.

^a^ Downgraded once because both studies are of some concern for bias (though neither is high risk). In particular, there is uncertainty around reporting bias and performance bias.

^b^ The range of the CI's on the RE model estimate are very wide, ranging from nearly zero to a very large estimate of 0.63.

^c^ Downgraded because three of four of the studies are of some concern for bias, and the other one is of high risk. Additionally, three of four of the studies are QEDs. There is uncertainty around the reporting bias ratings. When the high risk of bias study is removed, the effect estimates decreases slightly.

^d^ Downgraded because the Weber study is very inconsistent with the others in terms of magnitude of effect. That said, the studies all report positive effect sizes.

^e^ The regression test indicated funnel plot asymmetry (*p* < .01) but not the rank correlation test (*p* = .33).

^f^ Downgraded because the reporting bias and the selection bias criteria are unclear.

^g^ All single studies downgraded once for inconsistency.

^h^ Downgraded twice because both studies present a high risk of bias with significant likelihood of deviations from the intended intervention.

^i^ Downgraded twice due to uncertainty surrounding several criteria and a high risk of reporting bias.

^j^ Downgraded due to very wide confidence intervals.

^k^ Downgraded due to uncertainty with respect to selection and reporting biases.

^l^ Downgraded twice because point estimates vary widely and cross both sides of the threshold, including to nearly large effect levels.

^m^ Downgraded twice because: (1) both studies present at least concern for bias, and one is high risk of bias, and (2) when the high risk of bias study is removed, we are left with only a QED, and uncertainty and risk remains.

^n^ The effect estimates are very different, including CI's that do not overlap and who cross into both sides of the threshold despite one study indicating a very large effect estimate.

^o^ The RE model effect estimate has very large CI's which reach far across both sides of the threshold spanning a range of −0.21 to 0.66.

^p^ Downgraded due to uncertainty with respect to performance and reporting bias.

^q^ Downgraded due to uncertainty with respect to selection and reporting biases.

^r^ Downgraded twice because point estimates vary widely and cross both sides of the threshold, including to nearly large effect levels.

^s^ Downgraded due to uncertainty with respect to reporting bias.

^t^ Downgraded twice to reflect the three studies’ medium and high risk of bias. Particularly there is considerable risk and uncertainty surrounding reporting bias.

^u^ Downgraded because the Karlan study is very inconsistent with the others in the group, and that study provides 35.39% of the weight of the meta‐analysis.

^v^ Downgraded twice because the CI's are very large, crossing into both sides of the threshold despite a very large and positive effect estimate.

^w^ This single study was rated as having high risk of bias related to imbalanced attrition.

#### 5.3.6 Quotas

We define quotas as the allocation of positions of power on decision‐making bodies in government, organisations, or community groups to a particular demographic (often oppressed or minority groups) (IDEA, 2021).

##### 5.3.6.1 How do quotas affect gender equality, women's empowerment and Peace outcomes?

**Figure 70:**
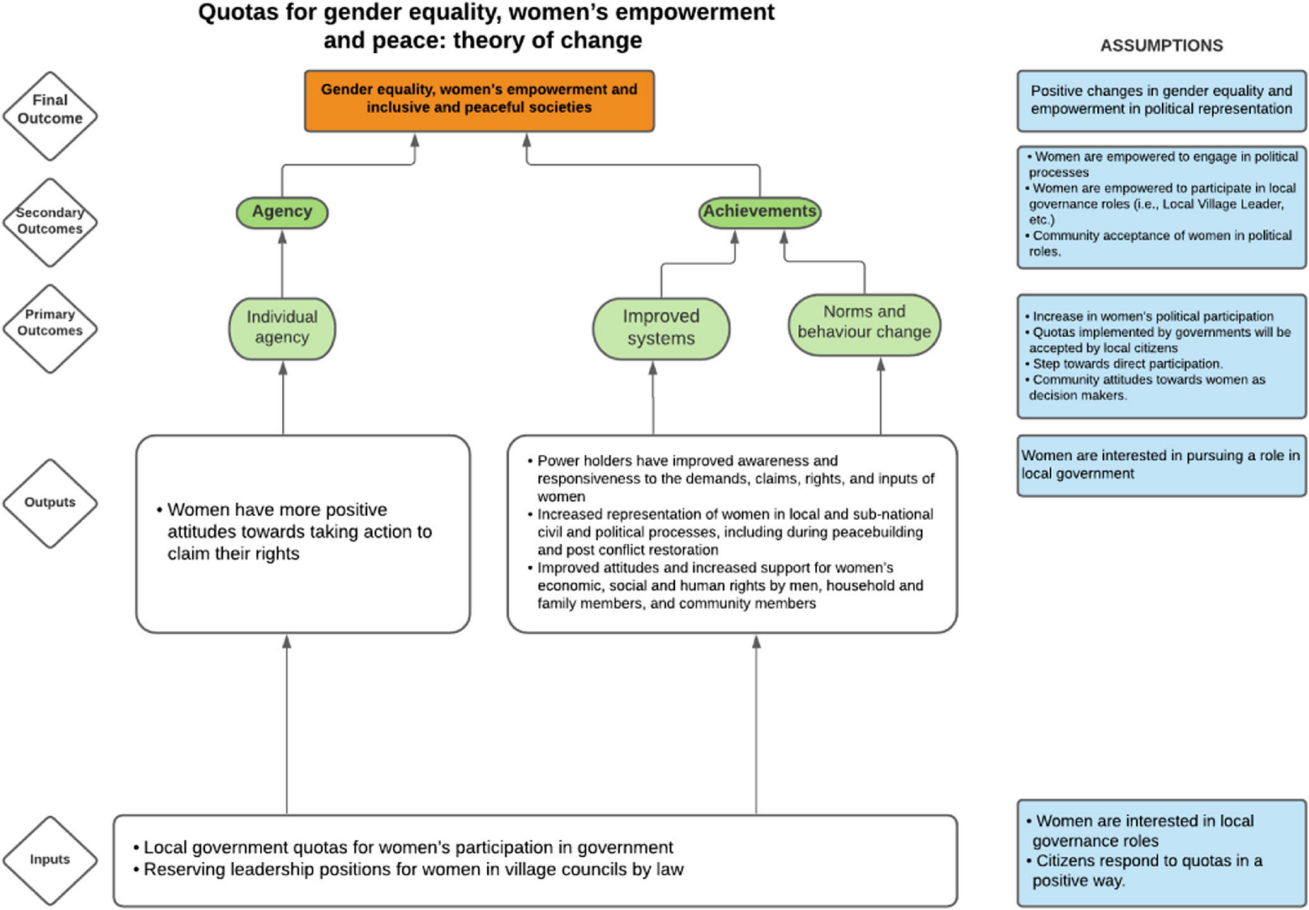
Quotas for gender equality, women's empowerment and peace: Theory of change

Figure 70 maps out the causal chain of how quotas may improve gender equality, women's empowerment and peace outcomes.

As illustrated in the figure below, quotas that are inclusive (such as local government quotas for women's participation in government, or legally reserving leadership positions for women in village councils) can help societies achieve high levels of agency and achievements, which are essential to the overall empowerment of women and inclusive and peaceful society. Agency and achievements rest on individual agency, improved systems, and norms and behaviour change, and are fostered by immediate outcomes that are generated from quota programmes. Immediate outcomes of such programmes include women have more positive attitudes towards taking action to claim their rights, power holders have improved awareness and responsiveness to the demands, claims, rights, and inputs of women, increased representation of women in local and subnational civil and political processes, including during peacebuilding and post conflict restoration, and improved attitudes and increased support for women's economic, social and human rights by men, household and family members and community members. Additionally, women may have different preferences for public goods than men, and thus may invest in different public goods, such as education and healthcare.

##### 5.3.6.2 Description of included studies

We included two studies reported in two different papers that evaluated the effect of quotas.

*Population*

The included studies focused at the community level, with special attention given in many cases to women. The included studies evaluated programmes in both South Asia and Sub‐Saharan Africa in two countries including India and Lesotho.

*Intervention, inputs and activities*

The included studies (Table 31) evaluated a range of different quota‐related activities and inputs including:

- Reserving leadership positions for women in village councils (*n* = 1): A 1993 law reserved leadership positions for women to participate in local village councils.
- Local government act enacted quotas for women's participation in local government positions (*n* = 1): The 2005 Local Government Elections Act required that 30% of all single‐member electoral divisions (Eds) (distributed evenly across the newly created councils) be reserved for only women councillors. Women still competed with other women in these Eds, but men were not allowed to compete (Table 26).

**Table 26:** Quota studies design features

Study	Activity/input	Length of treatment	Intervention frequency
Beaman et al. (2012)	Reserving leadership positions for women in village councils	N/A	N/A
Clayton (2015)	Local Government Act enacted quotas for women's participation in local government position	77 months	N/A

*Comparison*

All our included studies compared treated groups to comparison groups receiving no intervention. No study included multiple treatment arms.

*Outcomes*

The included studies reported on a number of relevant outcomes, including the following:

- Women have a more positive attitude towards taking action to claim their rights (*n* = 2): Women feel entitled to be engaged and given the leadership capacity and knowledge to claim their rights and take action on relevant issues. Increased self‐efficacy and autonomy. Increased opportunities for women to claim their rights including as a result of education and ealizedaliz. Women are engaged and given the leadership capacity and knowledge to claim their rights and take action on relevant issues.
- Power holders have improved awareness and responsiveness to the demands, claims, rights and inputs of women (*n* = 1): Power holders are able to ealized and support women/girls/ealizedali groups through their services. They both have knowledge and understanding of these specific rights and how to act accordingly. More opportunities for dialogue and feedback mechanisms are put in place, particularly relating to gender issues. The specific needs of ealizedali groups are taken into account in policies and interventions. Power holders take action towards supporting those groups.
- Increased representation of women in local and subnational civil and political processes, including during peacebuilding and post conflict restoration (*n* = 1): Women are represented in the institutions, organisations and other decision‐making processes and have capacities to influence the decision and direction of these processes. Women's specific needs and requests are taken into account and are reflected in the final decision, giving a full role to women in the civil and political processes.
- Improved attitudes and increased support for women's economic, social and human rights by men, household and family members and community members (*n* = 1): These positive attitudes can be shaped by ealizedaliz and education to shift social norms, particularly relating to ealizedali groups. As a result, they are aware of the specific needs of these groups and take an active part in reducing inequalities.

We report the distinction between the immediate and secondary outcomes in Table 32 (Table 27).

**Table 27:** Summary of secondary and immediate outcomes for quota studies

Secondary outcome category	Immediate outcome	Number of studies
Resources material, human and social resources which serve to enhance the ability to exercise choice	Access to justice and legal services	0
	Economic and livelihood related resources	0
	Access to employment	0
Agency ability to define one's goals and act upon them and ealizedalized decision‐making	Individual agency	2
	Community level agency	0
	Institutions supporting agency	0
Achievement ways of being and doing which can be ealized by different individuals	Improved systems	1
	Norms and behaviour change	1
	Empowerment index	0

*Study design*

**Figure 71:**
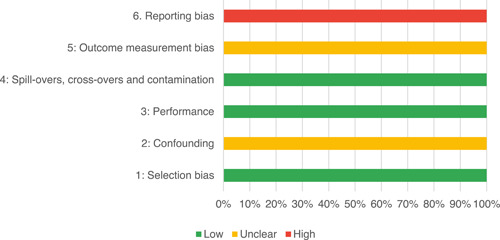
Quasi‐experimental design quotas risk of bias assessment

One of our quota‐focused studies implemented a QED. The only study was Clayton (2015), assessed as having a high risk of bias because matching methods are not clearly reported and there was no information provided on sensitivity analysis (e.g., using different matching methods or other robustness tests). We assessed outcome measurement as unclear since authors used administrative data and did not provide enough information about data collection processes and outcome measurement methods. We also assessed confounding as unclear because the authors did not report tests for hidden bias nor results from different matching methods varying sample sizes. We did not identify any limitations related to spill‐overs, crossovers, contamination, survey effects, performance or selection bias. Figure 66 presents the RoB assessment for the QED study (Figure 71).

**Figure 72:**
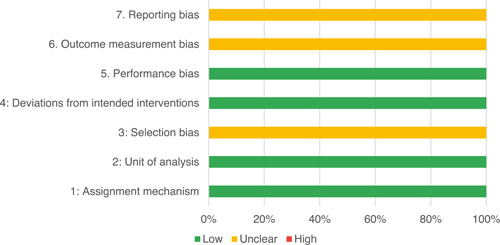
Randomised controlled trial quotas risk of bias assessment

The other quota study used an experimental design (Beaman et al., 2012). This study was assess as having some concerns related to risk of bias because there was not enough information provided to assess limitations. With regard to selection bias, authors used cross‐sectional data collected post‐intervention, while baseline balance was tested using census data, yet it was unclear whether results may suffer from sampling issues. We also assessed as unclear whether the study suffered from outcome measurement bias because the authors did not discuss blinding procedures or potential limitations with recall or self‐reported outcomes. Finally, it was also unclear whether there are reporting issues because there is not enough information to determine that there is an analysis missing. Figure 72 presents the RoB assessment for the RCT study:

*Qualitative studies, process evaluations and project documents*

We identified no linked qualitative studies and were unable to conduct a qualitative evidence synthesis related to quotas.

##### 5.3.6.3 Synthesis of findings

The following subsection presents the results of effectiveness of quotas on gender equality, women's empowerment and peace outcomes.

*Quantitative findings*

*Effects of quotas on women having more positive attitudes towards taking action to claim their rights*

**Figure 73:**
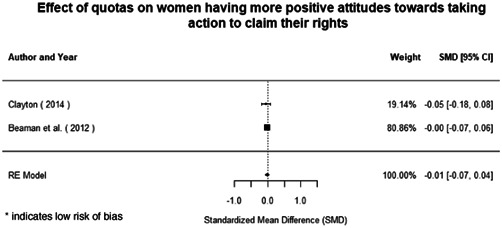
QuoAttitudes. Forest plot showing the observed outcomes and the estimate of the random‐effects model. CI, confidence interval

Only two studies using a quota intervention reported outcomes related to women having more positive attitudes towards taking action to claim their rights, thus we included *k *=* *2 studies in the analysis. We assessed one study as having some concerns of risk of bias and the other as high risk of bias. The estimated average outcome based on the random‐effects model was $\hat{\mu }=-0.01$ (95% CI: −0.07to0.04). Therefore, the average outcome did not differ significantly from zero (z=−0.46, p=0.64). A forest plot showing the observed outcomes and the estimate based on the random‐effects model is shown in Figure 73 (QuoAttitudes). Given the small number of studies, this result should be interpreted with caution. According to the Q‐test, there was no significant amount of heterogeneity in the true outcomes (Q(1)=0.36, p=0.55, ${\hat{\tau }}^{2}=0.00$, $I^{2}=0.00$%). With only two studies, moderator analyses were not possible and tests of publication bias are not valid.

*Effects of quotas on reduced instances of child or forced marriage*.

Beaman and colleagues' (2012) experimental study in India was the only study evaluating the impact of quota on reduced instances of child or forced marriage. There was a very small, but not statistically significant, point estimate (*g *=* *0.06, [95% CI: −0.01 to 0.12]), and we assessed the study as having some risk of bias concerns.

*Effects of quotas on power holders having improved awareness and responsiveness to the demands, claims, rights and inputs of women*

Clayton's (2015) quasi‐experimental study in Lesotho was the only study evaluating the impact of quota on power holders having improved awareness and responsiveness to the demands, claims, rights and inputs of women. Their report included two effects that fell into this outcome category (whether respondent can make councillor listen and whether her councillors try to listen). The point estimates were small, negative and not statistically significant (*g *=* *−0.15, [95% CI: −0.28 to −0.01] and *g *=* *−0.13, [95% CI: −0.27 to 0.00]). We assessed the study as having high risk of bias.

*Effects of quotas on representation of women in local and subnational civil and political processes, including during peacebuilding and post conflict restoration*

Clayton's (2015) quasi‐experimental study in Lesotho was the only study evaluating the impact of quota on increased representation of women in local and subnational civil and political processes, including during peacebuilding and post conflict restoration. Their report included two effects that fell into this outcome category (interest in politics and discuss politics). The effect was negative, medium and statistically significant for interest in politics (*g *=* *−0.26, [95% CI: −0.40 to −0.13]) and the point estimate was very small, not significant for whether the respondent discusses politics (*g *=* *−0.04, [95% CI: −0.18 to 0.09]). We assessed the study as having high risk of bias.

*Effects of quotas on attitudes and support for women's economic, social and human rights by men, household and family members and community members*

Beaman and colleagues' (2012) experimental study in India was the only study evaluating the impact of quota on improved attitudes and increased support for women's economic, social and human rights by men, household and family members and community members. Their report included 12 effects that fell into this outcome category (e.g., gap in parents' aspirations for girls vs. boys: the parent does not wish child to be housewife or whatever in‐laws prefer). The effects ranged from very small, negative point estimates (*g *=* *−0.03, [95% CI: −0.08 to 0.02]) to very small, positive effects (*g *=* *0.09, 95% CI [0.02 to 0.15]). We assessed the study as having some risk of bias concerns.

##### 5.3.6.4 Summary of findings and discussion

We included two studies in two countries in South Asia and Sub‐Saharan Africa that evaluated the effect of quotas. We were able to quantitatively examine effects on women having more positive attitude towards taking action to claim their rights, however, there was no significant impact. Our included studies report against two of the three secondary outcomes (Resources, Agency and Achievement) and three of the nine immediate outcomes for our review, the GRADE assessments generally indicate a low certainty in this body of evidence.

We identified no linked qualitative studies and were unable to conduct a qualitative evidence synthesis related to quotas. Table 33 presents the GRADE review of our findings (Table 28).

**Table 28:** GRADE summary of findings and certainty of evidence on quotas

Certainty assessment							Sample size	Effect	Certainty	Importance
No. of studies	Study design	Risk of bias	Inconsistency	Indirectness	Imprecision	Other considerations		Absolute (95% CI)		
*(BA5) Women have more positive attitude towards taking action to claim their rights*										
2	RCT‐1 QED‐1	Very serious^a^	Not serious	Not serious	Not serious	None	4550	SMD 0.01 SD lower (0.07 lower to 0.04 higher)	⊕⊕◯◯ LOW	Limited importance
*(BA8) Reduced instances of child or forced marriage*										
1	RCT	Serious^b^	Serious^c^	Not serious	Not serious	None	3680	SMD 0.06 SD higher (0.01 lower to 0.12 higher)	⊕⊕◯◯ LOW	Critical
*(CA1) Power holders have improved awareness and responsiveness to the demands, claims, rights and inputs of women*										
1	QED	Serious^d^	Serious^c^	Not serious	Not serious	None	870	Four negative effect estimates with a 95% CI range of −0.28 to 0.07	⊕⊕◯◯ LOW	Limited importance
*(CA2) Increased representation of women in local and subnational civil and political processes, including during peacebuilding and post conflict restoration*										
1	QED	Serious^e^	Serious^c^	Not serious	Not serious	None	870	Two negative effect estimates with a 95% CI range of −0.20 to 0.08	⊕⊕◯◯ LOW	Limited importance
*(CB4) Improved attitudes and increased support for women's economic, social and human rights by men, household and family members and community members*										
1	RCT	Serious^f^	Serious^c^	Not serious	Not serious	None	3680	One positive and one negative effect estimate (both near null) with a 95% CI range of −0.07 to 0.14	⊕⊕◯◯ LOW	Limited importance

Abbreviations: CI, confidence interval; GRADE, Grading of Recommendations, Assessment, Development and Evaluations; QED, quasi‐experimental design; RCT, randomised controlled trial; SMD, standardised mean difference.

^a^ Downgraded because half of the evidence base is coming from a high risk of bias QED, and the other half from a some concerns related to risk of bias.

^b^ Downgraded due to uncertainty and some risk of bias across numerous criteria.

^c^ All single studies downgraded once for inconsistency.

^d^ Downgraded due to bias across a number of criteria, most notably outcome measurement bias.

^e^ Downgraded due to bias across a number of criteria, most notably outcome measurement bias.

^f^ Downgraded due to uncertainty and some risk of bias across numerous criteria.

#### 5.3.7 Self‐help groups and village savings and loan associations

Self‐help groups (SHGs) and Village Savings and Loan Associations (VSLAs) are bringing people together informally or through associations to work collectively towards mutual goals, often related to savings and the provision of small loans (Khasnabis et al., 2010). This category includes what is commonly referred to in existing literature as VSLAs, which is a group of people who meet regularly to save together and take small loans from those savings (VSL Associates, 2021).

##### 5.3.7.1 How do self‐help groups and VSLAs affect gender equality, women's empowerment and Peace outcomes?

**Figure 74:**
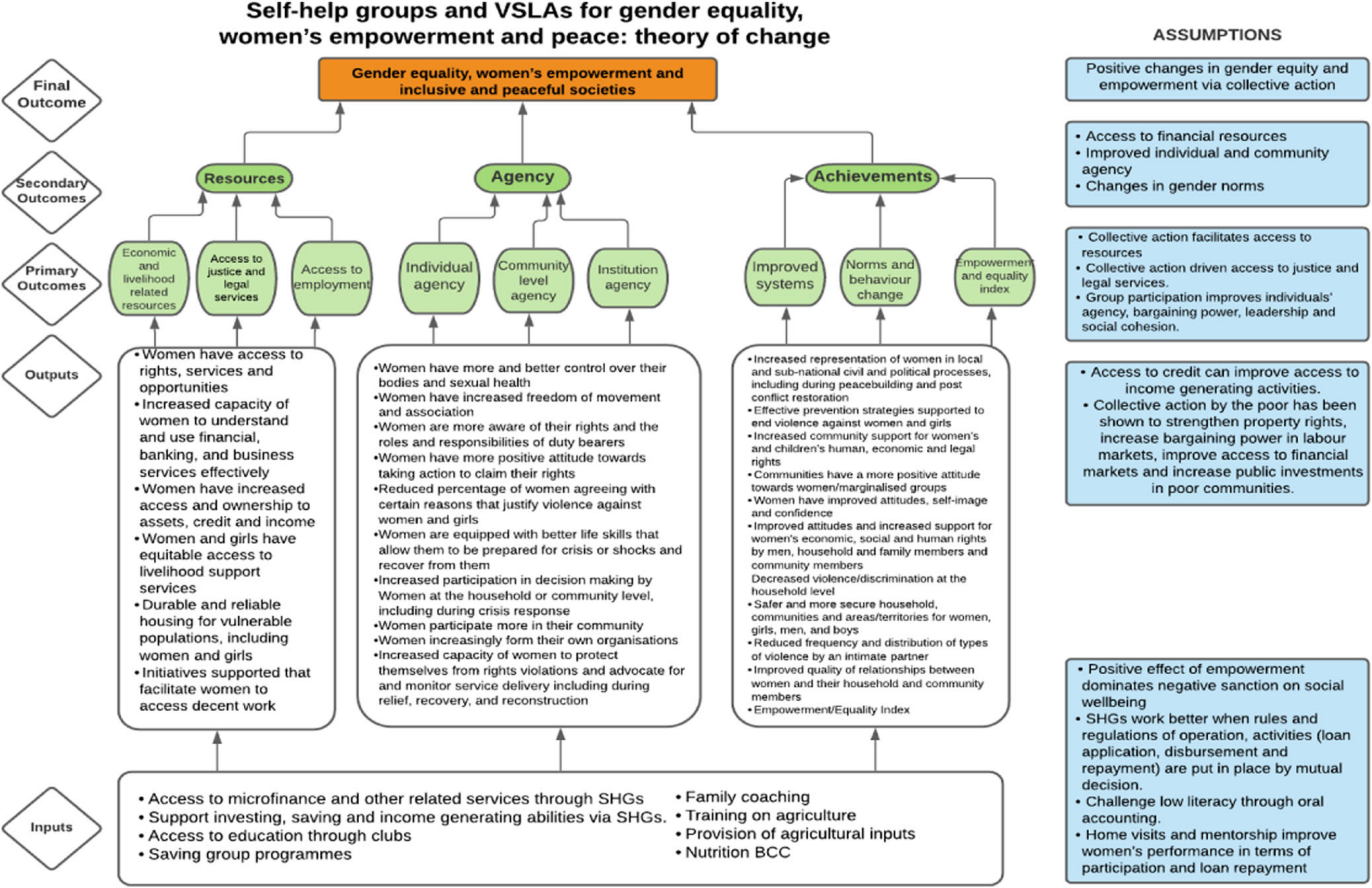
Self‐help groups and Village Savings and Loan Associations (VSLAs) for gender equality, women's empowerment and peace: Theory of change

Figure 74 maps out the causal chain of how self‐help groups and VSLAs may improve gender equality, women's empowerment and peace outcomes. SHGs and VSLA interventions particularly bring together women (and men) with common needs into self‐operating groups. The ToC implies that poor women, who lack agency and opportunity to access financial and livelihood resources, can do so via collective action. By forming groups and setting rules to govern the groups, women can collectively save and borrow money. Groups can organise and deliver skills to access livelihood activities, provide access to formal financial institutions and markets, and develop soft life‐skills. It is expected that such collective actions can improve women's financial sovereignty and families' well‐being, boost their self‐esteem and confidence, enhance their decision‐making power and widen their social network and support system. This builds on the direct benefits to credit and assets that participating in these groups often brings.

##### 5.3.7.2 Description of included studies

We included 12 studies reported in 11 different papers that evaluated the effect of six different programmes.

*Population*

Self‐help groups and VSLAs primarily operate at the community level. Individual women or more than one member from the same household can participate in a group. Out of the 11 papers evaluated in this category, 10 of them exclusively looked at groups for women and girls. Only one study evaluated SHGs that also allowed men to participate.

The included studies evaluated seven different programmes and trials in South Asia and Sub‐Saharan Africa and seven countries: Burkina Faso, DRC, Ethiopia, Ghana, India, Malawi, Mali, Uganda and Zambia.

*Intervention, inputs and activities*

The included studies evaluated a range of different SHG and VSLA activities and inputs including:

- Access to microfinance for women (*n* = 1): Implemented by Rojiroti Microfinance, a microfinance institution which serves people who are poorer and more marginalised than those served by other microfinance programmes. The Rojiroti approach consisted of the creation of women's self‐help groups, rotated loans from savings, and subsequent credit from CPSI, a Bihar based NGO.
- Formation of SHGs—once SHGs are formed, then access to credit and other services is facilitated (*n* = 1): Sponsored by the Indian government, the Swarnajayanti Gram Swarozgar Yojana was a credit scheme where self‐help groups are setup for members to mobilise, save credit and facilitate loans.
- Girls' and women's clubs for supporting girls and women's empowerment through access to education (*n* = 1): Implemented by OXFAM, this programme consisted of setting up mothers' associations to conduct training and sensitisation activities, setting up girls' club to deliver sensitisation training, setting up training, identifying role‐model women to organise exchanges on topics.
- Savings group programme for women (*n* = 1): Implemented by the International Rescue Committee and targeted towards women survivors of sexual violence, VSLAs are economic programmes in which individuals join with people they know to regularly save funds and build capital to generate small loans to other members.
- Self‐help groups (*n* = 2): Implemented by the Government of Ethiopia, this programme aimed to create self‐help groups for women working in apple production. SHGs are groups of 10–20 women initiated by a development agency, which are usually involved in savings and credit programmes and/or advancing group members' rights.
- Microfinance SHG scheme (*n* = 1): The Self‐Employed Women's Association (SEWA) set up SHGs in the Dungarpur district of Rajasthan. These SHGs set their own savings targets for their members. The funds were put in a savings account with a linked bank and were lent out to members of the SHG.
- Multicomponent intervention involving training and group creation, with the aim of increase investing, saving, income generating abilities (*n* = 1): Following the Gender Action Learning System approach, the project established women's groups to promote savings and investment opportunities and be trained on knowledge for running small enterprises. The project also supported women's groups in gaining access to loans to start new business.
- VLSA model, organising women into groups and providing seed capital for initiatives + family coaching (*n* = 1): The experiment consisted of (1) a package of economic and livelihood interventions for women caregivers that included formation and training using the Village Savings and Loan Association (VSLA), seed capital grants to jump‐start or expand livelihood activities and one‐on‐one mentoring; and (2) a gender sensitive family coaching component.
- VSLAs (*n* = 1): Begun by CARE International and implemented by the International Rescue Committee and other INGOs, VSLAs are economic programmes in which individuals join with people they know to regularly save funds and build capital to generate small loans to other members.
- Women's groups for agricultural training, provision of initial inputs and nutrition BCC (*n* = 1): This Concern Worldwide‐supported programme entailed group sessions on agriculture, gender equality and women's empowerment, (Ag‐G group), nutrition targeted behavioural change communication interventions and provision of standard government services such as maize‐focused agricultural extension targeted predominantly to male farmers, and antenatal care visits and growth monitoring for under 5‐year‐old children (Table 29).

**Table 29:** SHGs and VSLAs design features of included studies

Study	Activity/input	Length of treatment	Intervention frequency
Alemu et al. (2018)	SHGs	36 months	Unclear
Bass et al. (2016)	VSLAs	12 months	Weekly
Beaman et al. (2014)	Saving group programme for women	36 months	Weekly
De Hoop et al. (2010)	SHGs	Unclear	Unclear
De Hoop et al. (2014)	SHGs	36 months	Unclear
Deininger and Liu (2013)	SHGs	Unclear	Weekly
Desai and Joshic (2013)	Microfinance SHG scheme through SEWA	24 months	Monthly
Ismayilova et al. (2018)	VLSA model, organising women into groups and providing seed capital for initiatives + family coaching	24 months	Weekly
Karlan et al. (2017)	VSLA model with agents whose objective was to informally expand the VSLA model to other communities	30 months in Ghana. 22 months in Malawi and Uganda	Weekly
Kundu and Mukherjee (2011)	Formation of SHGs and access to credit and other services was facilitated	48 months	Unclear
Lombardini and Yoshikawa (2015)	Multicomponent intervention involving training and group creation, with the aim of increase investing, saving, income generating abilities	33 months	Monthly
Vigneri and Lombardini (2017)	Girls' and women's clubs for supporting girls and women's empowerment through access to education	50 months	Weekly
Yaron et al. (2018)	Access to microfinance for women	18 months	Unclear

Abbreviations: SEWA, Self‐Employed Women's Association; SHG, self‐help group; VSLA, Village Savings and Loan Association.

*Comparison*

All our included studies are comparing treated groups to comparison groups receiving no intervention. Seven studies included multiple treatment arms.

*Outcomes*

The included studies reported on a number of relevant outcomes, including:

- Women have increased access and ownership to assets, credit and income (*n* = 8): Women are able to apply for, receive and manage assets/credit and income and have support to manage, claim and execute their assets without pressure or influence from external actors, including male family members, husbands and cultural leaders.
- Women are equipped with better life skills that allow them to be prepared for crisis or shocks and recover from them (*n* = 4): Women develop skills, have access to resources, and make use of these skills and resources to increase their level of resilience and be equipped to face life challenges (health, education, finance, social relations, work, etc.).
- Increased participation in decision making by women at the household or community level, including during crisis response (*n* = 7): Women take part in all or any step of the decision‐making process at the household, community and district level, but also are able to meaningfully take part and have influence on the final decision, including in crisis response.
- Decreased violence and discrimination at the household level (*n* = 3): The occurrence of these acts decreases over time as a result of interventions focusing on prevention and response.

The division of the immediate and secondary outcomes is reported in Table 35 (Table 30).

**Table 30:** Self‐help groups and Village Savings and Loan Associations summary of secondary and immediate outcomes

Secondary outcome category	Immediate outcome	Number of studies
Resources material, human and social resources which serve to enhance the ability to exercise choice	Access to justice and legal services	1
	Economic and livelihood related resources	9
	Access to employment	2
Agency ability to define one's goals and act upon them and operationalised decision‐making	Individual agency	9
	Community level agency	9
	Institutions supporting agency	1
Achievement ways of being and doing which can be realised by different individuals	Improved systems	4
	Norms and behaviour change	8
	Empowerment index	4

*Study design*

**Figure 75:**
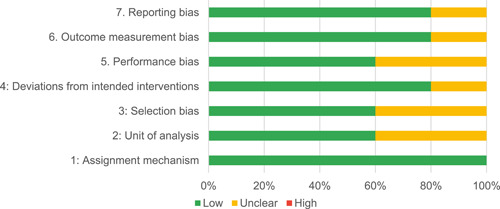
Self‐help groups and Village Savings and Loan Association studies quasi‐experimental design risk of bias assessment

Seven of our SHG and VSLA studies used a QED. This included Alemu et al. (2018), De Hoop et al. (2010), Kundu and Mukherjee (2011), Lombardini and Yoshikawa (2015), Vigneri and Lombardini (2017), Yaron et al. (2018). We assessed half of the QED studies evaluation SHG and VSLA as having high risk of bias and the other lf as having some concerns, none was assessed as having low risk or bias. As detailed in Figure 75, high risk of bias was identified in four RoB domains (selection bias, confounding bias, spill‐overs and reporting bias). Potential issues with selection bias were observed when it was not clear that all relevant characteristics had been accounted for (Vigneri & Lombardini, 2017; Kundu & Mukherjee, 2011). Confounding was a risk when authors did not control for relevant characteristics that may be correlated with both participation and outcomes of interest (Ibid.). Issues with reporting bias included a lack of robustness checks (Lombardini & Yoshikawa, 2015) and uncommon methods used for the analysis such as no adjustment for covariates on a matched sample (Vigneri & Lombardini, 2017). Finally, the presence of similar initiatives in the intervention areas was considered a risk of contamination (Ibid.). Outcome measurement bias was assessed as unclear for all but one of the studies because the authors had not provided enough information or discussion about recall or self‐reported bias.

**Figure 76:**
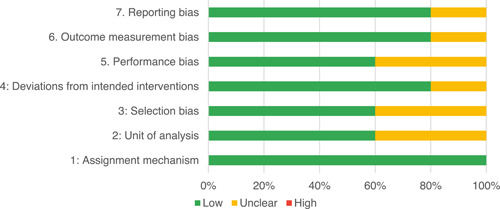
Self‐help groups and Village Savings and Loan Association studies randomised controlled trial risk of bias assessment

Five SHG and VSLA studies used an experimental design. This included Bass et al. (2016), Beaman et al. (2014), Desai and Joshic (2013), Ismayilova et al. (2018), Karlan et al. (2017) and Kumar et al. (2018). All six studies had some concerns related to risk of bias. As detailed in Figure 76, high risk of bias was not identified in any RoB domains of the sample of included RCT studies. No major concerns were observed, however one or two studies in each category did not provide enough information to discard issues related to deviations from intended interventions, selection bias, performance bias, reporting bias, assignment mechanism or unit of analysis. The only domain for which all studies were assessed as having low risk of bias was the outcome measurement one.

##### 5.3.7.3 Qualitative studies, process evaluations and project documents

We identified 10 additional documents related to five programmes covered by the SHG and VSLA group of studies.

- Saving for Change (SfC) (Mali): two qualitative documents, two process evaluations and two descriptive quantitative documents
- SEWA programme (India): one qualitative document
- Village Savings and Loans Associations (VSLAs) (DRC): two descriptive quantitative documents
- Girls Can: Promoting secondary education for girls in West Africa (Mali): one descriptive quantitative document

The highest number of studies were ranked as high quality (*n* = 5), followed by low quality (*n* = 4). Only one study from this intervention group was marked as moderate quality.

##### 5.3.7.4 Synthesis of findings

*Quantitative findings*

*Effect of SHGs on women's access to rights, services and opportunities*

Lombardini and Yoshikawa's (2015) quasi‐experimental study in Uganda was the only study evaluating the impact of self‐help groups and VSLA on women have access to rights, services and opportunities. There was a small, and statistically significant, effect (*g *=* *0.18, 95% CI [0.03, 0.32]), however we assessed the study as having high risk of bias.

*Effect of SHGs on increased capacity of women to understand and use financial, banking and business services effectively*

**Figure 77:**
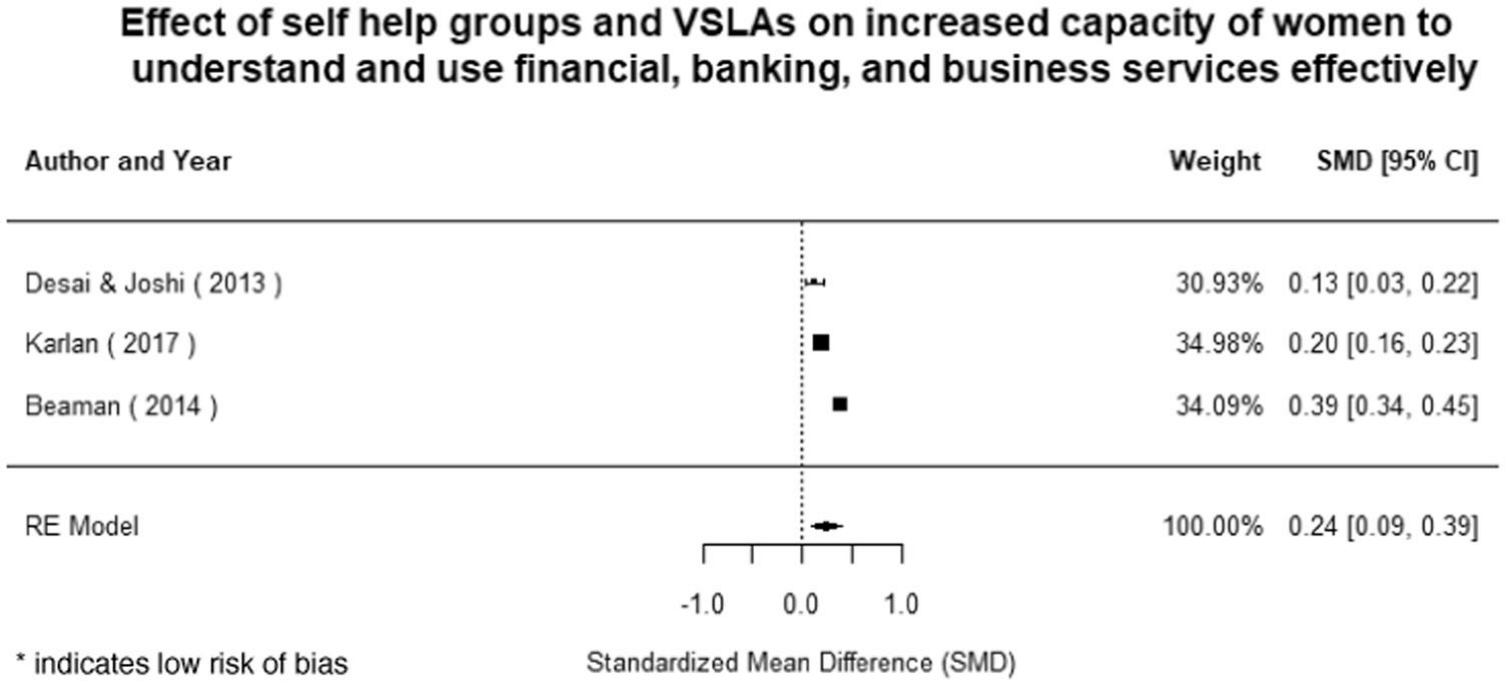
SHGAB1: Forest plot showing the observed outcomes and the estimate of the random‐effects model. CI, confidence interval; VSLA, Village Savings and Loan Association

We included a total of k=3 studies in the analysis. We assessed all three studies as having some concerns of risk of bias. The observed outcomes ranged from 0.13 to 0.39. The estimated average outcome based on the random‐effects model was $\hat{\mu }=0.24$ (95% CI: 0.09 to 0.39). Therefore, the average outcome differed significantly from zero (z=3.15, p<0.01). A forest plot showing the observed outcomes and the estimate based on the random‐effects model is shown in Figure 77 (SHGAB1). Given the small number of studies, this result should be interpreted with caution. According to the Q‐test, the true outcomes appear to be heterogeneous (Q(2)=42.94, p<0.01, ${\hat{\tau }}^{2}=0.02$, $I^{2}=95.34$%). An examination of the studentized residuals revealed that one study (Beaman et al., 2014) had a value larger than ±2.39 and may be a potential outlier in the context of this model. According to the Cook's distances, none of the studies could be considered to be overly influential. Indeed, sensitivity analyses leaving each study out indicated that removing Beaman et al. (2014) would reduce the overall average effect ($\hat{\mu }$ = 0.18 [95% CI: 0.12 to 0.24]), but the effect would still be positive and nonsignificant (*z *=* *5.61, *p *<* *0.001). With only three studies, moderator analyses were not appropriate.

*Effect of SHGs on women having increased access and ownership to assets, credit and income*

**Figure 78:**
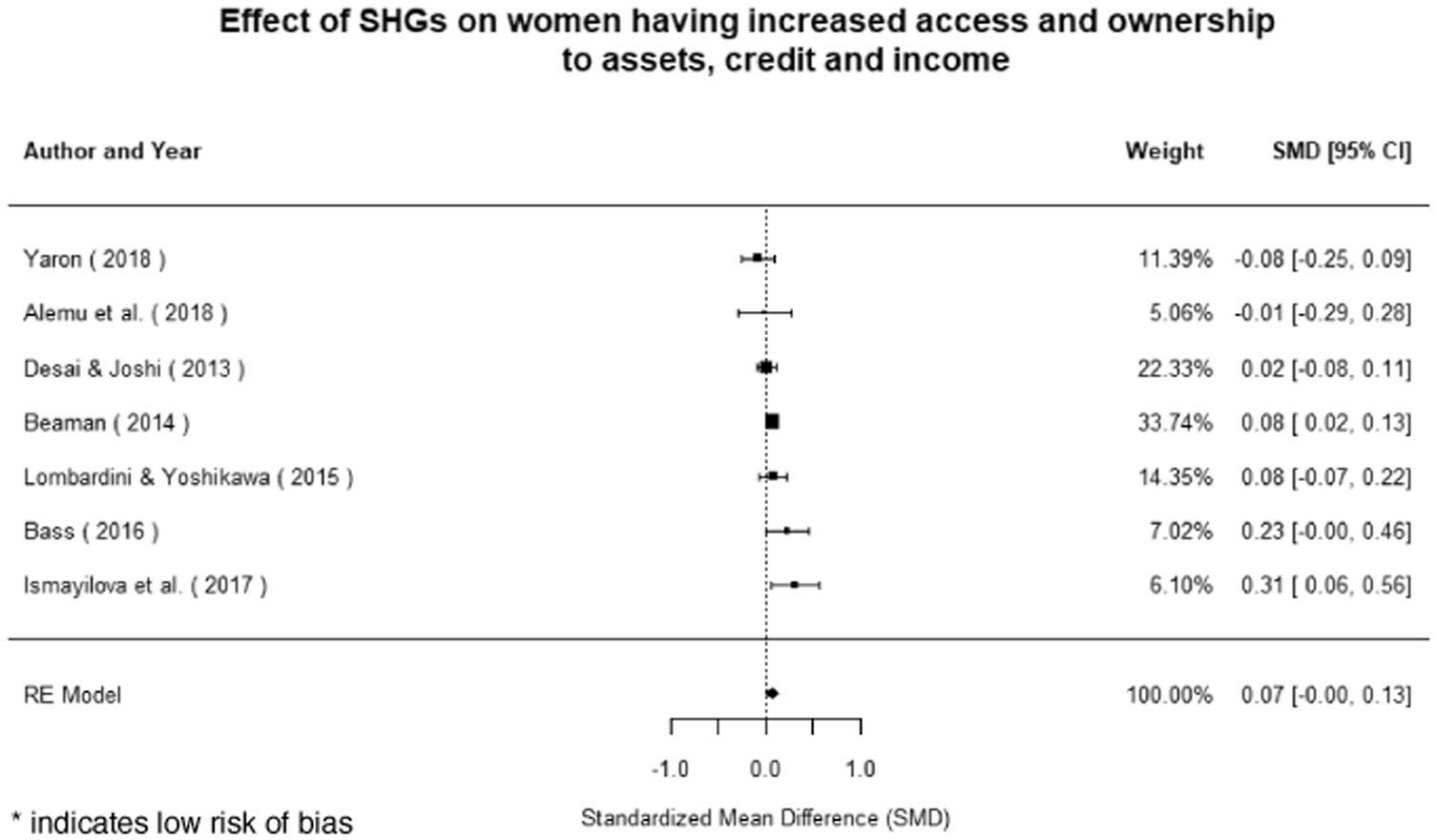
SHGAB2: Forest plot showing the observed outcomes and the estimate of the random‐effects model. CI, confidence interval; SHGs, self‐help groups

We included a total of k=7 studies in the analysis. We assessed none of the studies as low risk of bias, six as some concerns, and one as high risk of bias. The observed outcomes ranged from −0.08 to 0.31. The estimated average outcome based on the random‐effects model was $\hat{\mu }=0.07$ (95% CI: −0.00 to 0.13). Therefore, the average outcome did not differ significantly from zero (z=1.90, p=0.06). A forest plot showing the observed outcomes and the estimate based on the random‐effects model is shown in Figure 78 (SHGAB2).

According to the Q‐test, there was no significant amount of heterogeneity in the true outcomes (Q(6)=9.69, p=0.14, ${\hat{\tau }}^{2}=0.00$, $I^{2}=38.08$%). An examination of the studentised residuals revealed that none of the studies had a value larger than ±2.69 and hence there was no indication of outliers in the context of this model. According to the Cook's distances, none of the studies could be considered to be overly influential. We tested all moderators, but there were no significant sources of heterogeneity for this outcome.

*Effect of SHGs on women and girls' access to livelihood support services*

Alemu and colleagues' (2018) quasi‐experimental study in Ethiopia was the only study evaluating the impact of self‐help groups and VSLA on women and girls having equitable access to livelihood support services. There was a medium, and statistically significant, effect (*g *=* *0.31, [95% CI: 0.03 to 0.60]), and we assessed the study as having some risk of bias concerns.

*Effect of SHGs on durable and reliable housing for vulnerable populations, including women and girls*

Yaron et al.'s (2018) quasi‐experimental study in India was the only study evaluating the impact of self‐help groups and VSLA on durable and reliable housing for vulnerable populations, including women and girls. There was a large, and statistically significant, effect (*g *=* *0.40, [95% CI: 0.22 to 0.57]), and we assessed the study as having some risk of bias concerns.

*Effect of SHGs on women's engagement in other micro, small, and medium‐sized enterprises*

Beaman et al.'s (2014) experimental study in Mali was the only study evaluating the impact of self‐help groups and VSLA on more women being engaged in other micro, small, and medium‐sized enterprises. There was a very small, but not statistically significant, point estimate (*g *=* *0.03, [95% CI: −0.01 to 0.07]), and we assessed the study as having some risk of bias concerns.

*Effect of SHGs on initiatives supported that facilitate women to access decent work (formal and informal employment), including people with disabilities*

**Figure 79:**
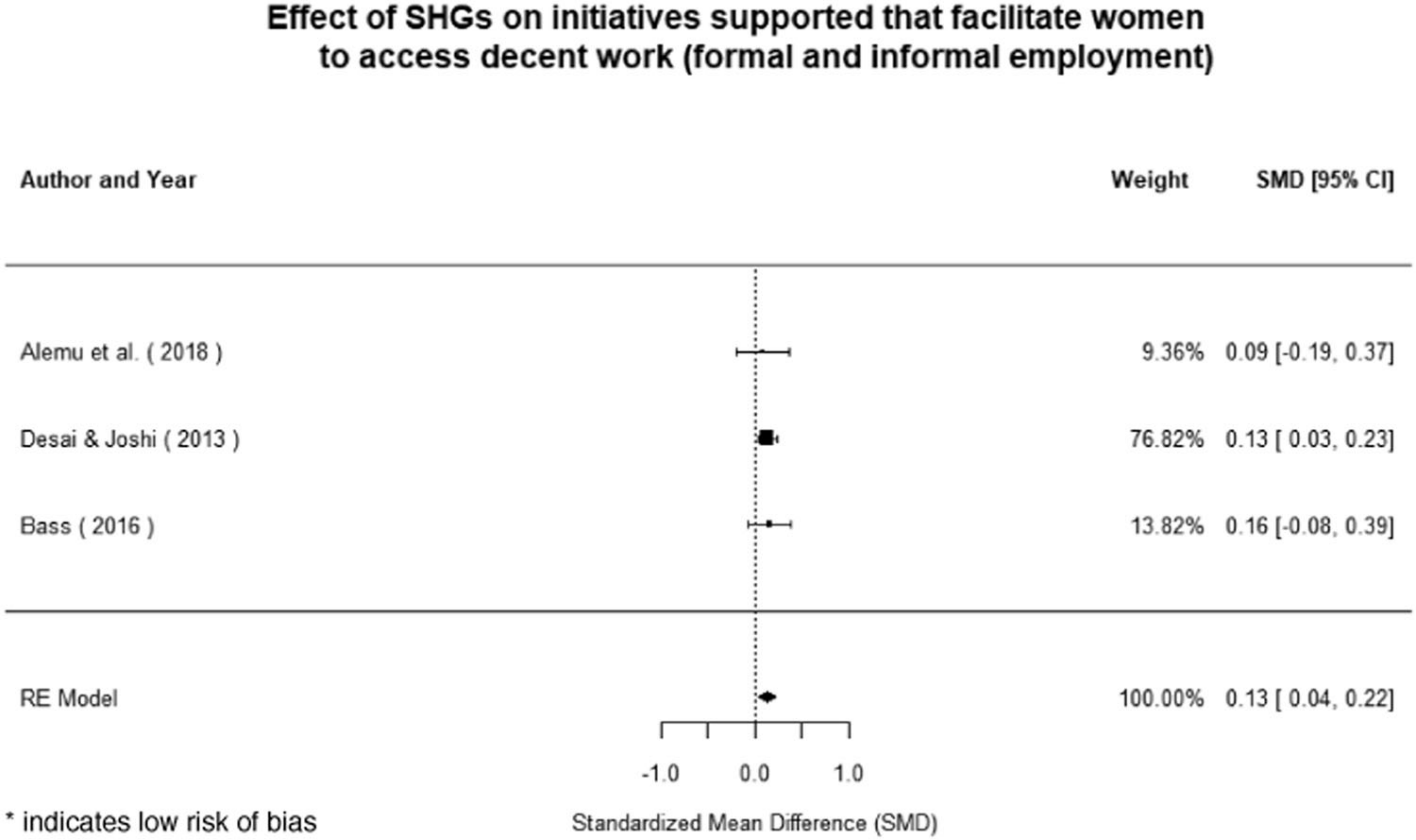
SHGAC2: Forest plot showing the observed outcomes and the estimate of the random‐effects model. CI, confidence interval; SHGs, self‐help groups

We included a total of k=3 studies in the analysis. We assessed all three studies as having some concerns of risk of bias. The observed outcomes ranged from 0.09 to 0.16. The estimated average outcome based on the random‐effects model was $\hat{\mu }=0.13$ (95% CI: 0.04 to 0.22). Therefore, the average outcome differed significantly from zero (z=2.97, p<0.01). A forest plot showing the observed outcomes and the estimate based on the random‐effects model is shown in Figure 79 (SHGAC2).

According to the Q‐test, there was no significant amount of heterogeneity in the true outcomes (Q(2)=0.12, p=0.94, ${\hat{\tau }}^{2}=0.00$, $I^{2}=0.00$%). An examination of the studentised residuals revealed that none of the studies had a value larger than ±2.39 and hence there was no indication of outliers in the context of this model. According to the Cook's distances, none of the studies could be considered to be overly influential. With only three studies and no heterogeneity among the effects, moderator analyses were not possible.

*Effect of SHGs on improved capacity of women entrepreneurs*

Lombardini and Yoshikawa's (2015) quasi‐experimental study in Uganda was the only study evaluating the impact of self‐help groups and VSLA on improved capacity of women entrepreneurs. There was a small, and statistically significant, effect (*g *=* *0.19, [95% CI: 0.05 to 0.34]), and we assessed the study as having high risk of bias.

*Effect of SHGs on women having more and better control over their bodies and sexual health*

Lombardini and Yoshikawa's (2015) quasi‐experimental study in Uganda was the only study evaluating the impact of self‐help groups and VSLA on women having more and better control over their bodies and sexual health. There was a very small, but not statistically significant, point estimate (*g *=* *−0.01, [95% CI: −0.16 to 0.13]), and we assessed the study as having high risk of bias.

*Effect of SHGs on women having increased freedom of movement and association*

**Figure 80:**
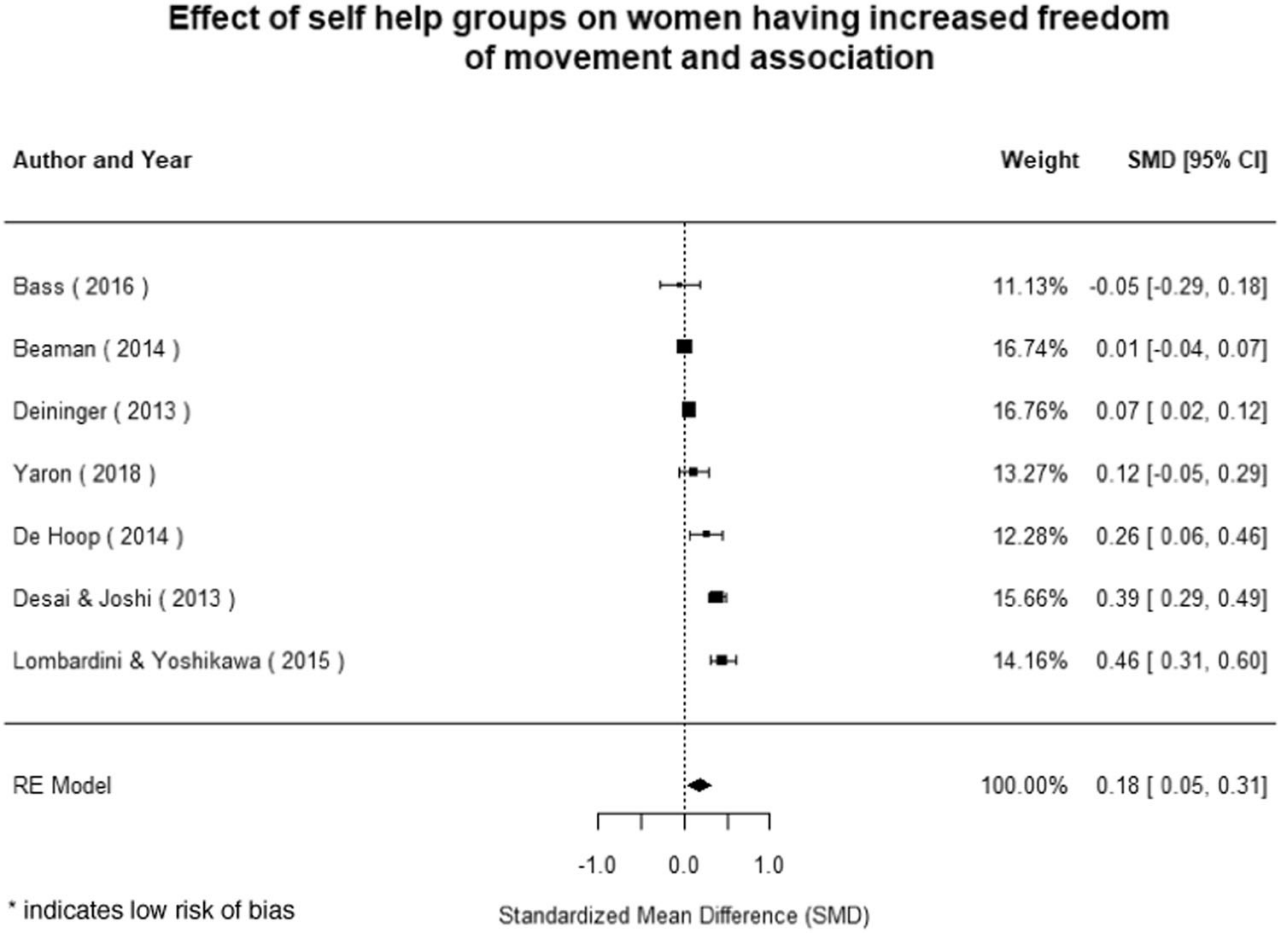
SHGBA3: Forest plot showing the observed outcomes and the estimate of the random‐effects model. CI, confidence interval

We included a total of k=7 studies in the analysis. We assessed none of the studies as low risk of bias, six as some concerns and one as high risk of bias. The observed outcomes ranged from −0.05 to 0.46. The estimated average outcome based on the random‐effects model was $\hat{\mu }=0.18$ (95% CI: 0.05 to 0.31). Therefore, the average outcome did not differ significantly from zero (z=2.72, p<0.01). A forest plot showing the observed outcomes and the estimate based on the random‐effects model is shown in Figure 80 (SHGBA3).

According to the Q‐test, the true outcomes appear to be heterogeneous (Q(6)=71.20, p<0.01, ${\hat{\tau }}^{2}=0.03$, $I^{2}=91.57$%). An examination of the studentised residuals revealed that none of the studies had a value larger than ±2.69 and hence there was no indication of outliers in the context of this model. According to the Cook's distances, none of the studies could be considered to be overly influential. We found no significant sources of heterogeneity through our moderator analyses.

*Effect of SHGs on women being more aware of their rights and the roles and responsibilities of duty bearers*

**Figure 81:**
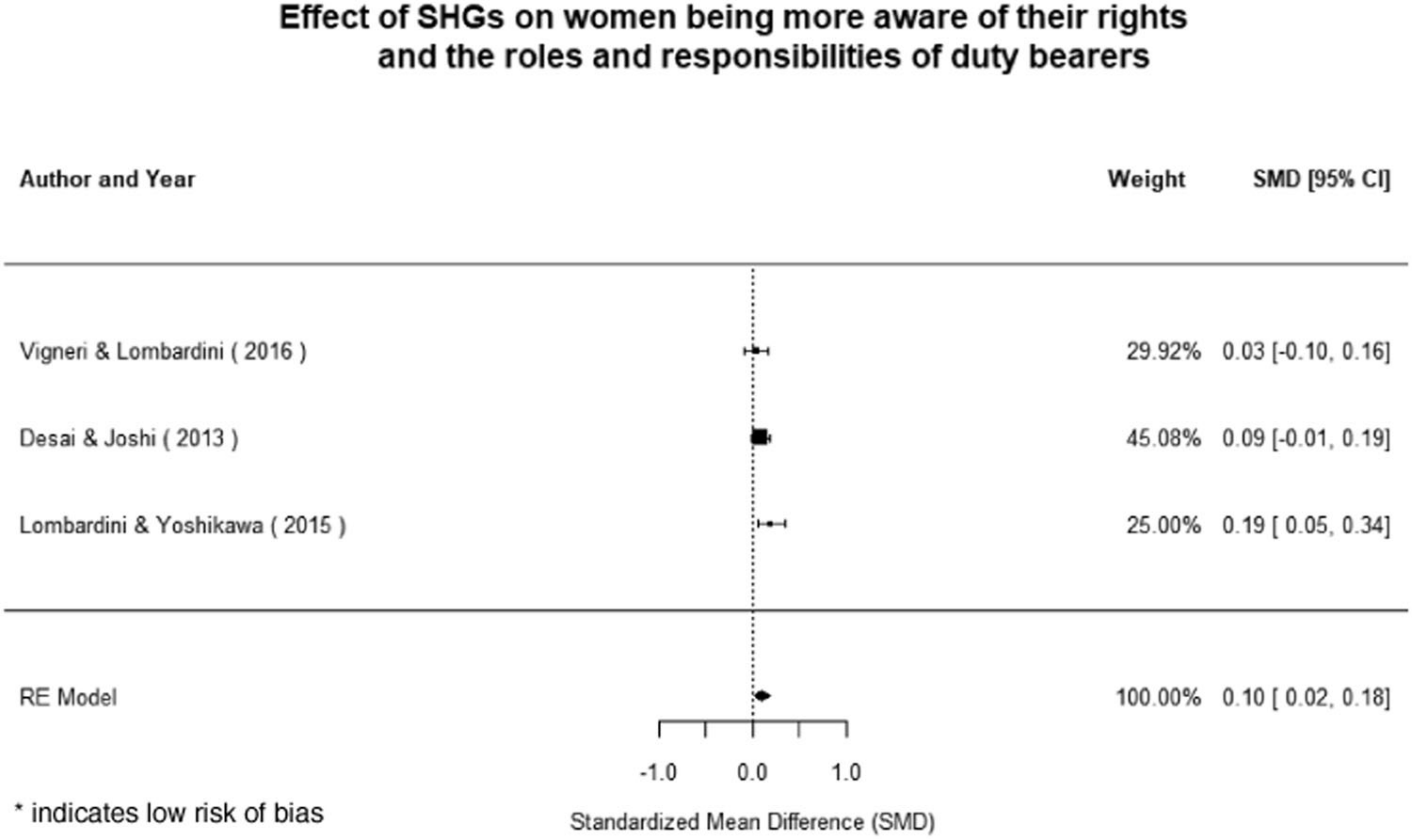
SHGBA4: Forest plot showing the observed outcomes and the estimate of the random‐effects model. CI, confidence interval; SHGs, self‐help groups

We included a total of k=3 studies in the analysis. We assessed none of the studies as low risk of bias, one as having some concerns, and two as low risk of bias. The observed outcomes ranged from 0.03 to 0.19. The estimated average outcome based on the random‐effects model was $\hat{\mu }=0.10$ (95% CI: 0.02 to 0.18). Therefore, the average outcome differed significantly from zero (z=2.39, p=0.02). A forest plot showing the observed outcomes and the estimate based on the random‐effects model is shown in Figure 81 (SHGBA4).

According to the Q‐test, there was no significant amount of heterogeneity in the true outcomes (Q(2)=2.59, p=0.27, ${\hat{\tau }}^{2}=0.00$, $I^{2}=22.64$%). An examination of the studentised residuals revealed that none of the studies had a value larger than ±2.39 and hence there was no indication of outliers in the context of this model. According to the Cook's distances, none of the studies could be considered to be overly influential. With only three studies, moderator analyses were not appropriate.

*Effect of SHGs on women having more positive attitude towards taking action to claim their rights*

**Figure 82:**
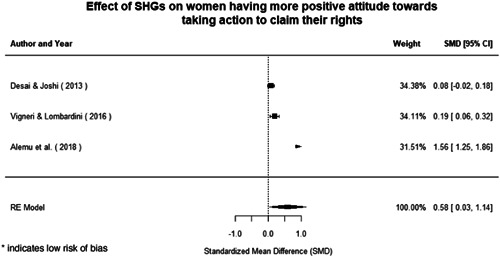
SHGBA5: Forest plot showing the observed outcomes and the estimate of the random‐effects model. CI, confidence interval; SHGs, self‐help groups

We included a total of k=3 studies in the analysis. We assessed none of the studies as low risk of bias, two as having some concerns, and one as low risk of bias. The observed outcomes ranged from 0.08 to 1.56. The estimated average outcome based on the random‐effects model was $\hat{\mu }=0.58$ (95% CI: 0.03 to 1.14). Therefore, the average outcome differed significantly from zero (z=2.06, p=0.04). A forest plot showing the observed outcomes and the estimate based on the random‐effects model is shown in Figure 82 (SHGBA5).

According to the Q‐test, the true outcomes appear to be heterogeneous (Q(2)=82.66, p<0.01, ${\hat{\tau }}^{2}=0.23$, $I^{2}=97.58$%). An examination of the studentised residuals revealed that one study (Alemu et al., 2018) had a value larger than ±2.39 and may be a potential outlier in the context of this model. According to the Cook's distances, none of the studies could be considered to be overly influential. Sensitivity analyses leaving each study out indicated that removing Alemu and colleagues (2018) would reduce the overall average effect ($\hat{\mu }=0.13$; 95% CI: 0.02 to 0.24), but the effect would still be positive and significant (*z *=* *2.31, p=0.02). With only three studies, moderator analyses were not appropriate.

*Effect of SHGs on reduced percentage of women agreeing with certain reasons that justify violence against women and girls*

**Figure 83:**
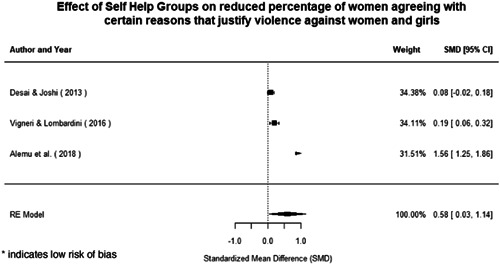
SHGBA6: Forest plot showing the observed outcomes and the estimate of the random‐effects model. CI, confidence interval

We included a total of k=3 studies in the analysis. We assessed none of the studies as low risk of bias, one as having some concerns, and two as low risk of bias. The observed outcomes ranged from 0.08 to 1.56. The estimated average outcome based on the random‐effects model was $\hat{\mu }=0.58$ (95% CI: 0.03 to 1.14). Therefore, the average outcome differed significantly from zero (z=2.06, p=0.04). A forest plot showing the observed outcomes and the estimate based on the random‐effects model is shown in Figure 83 (SHGBA6).

According to the Q‐test, the true outcomes appear to be heterogeneous (Q(2)=82.66, p<0.01, ${\hat{\tau }}^{2}=0.23$, $I^{2}=97.58$%). An examination of the studentised residuals revealed that one study (Alemu et al., 2018) had a value larger than ±2.39 and may be a potential outlier in the context of this model. According to the Cook's distances, none of the studies could be considered to be overly influential. sensitivity analyses leaving each study out indicated that removing Alemu et al. (2018) would reduce the overall average effect ($\hat{\mu }$= 0.13 [95% CI: 0.02 to 0.24]), but the effect would still be positive and significant (*z *=* *2.31, p= 0.02). With only three studies, moderator analyses were not appropriate.

*Effect of SHGs on women being equipped with better life skills that allow them to be prepared for crisis or shocks and recover from them*

**Figure 84:**
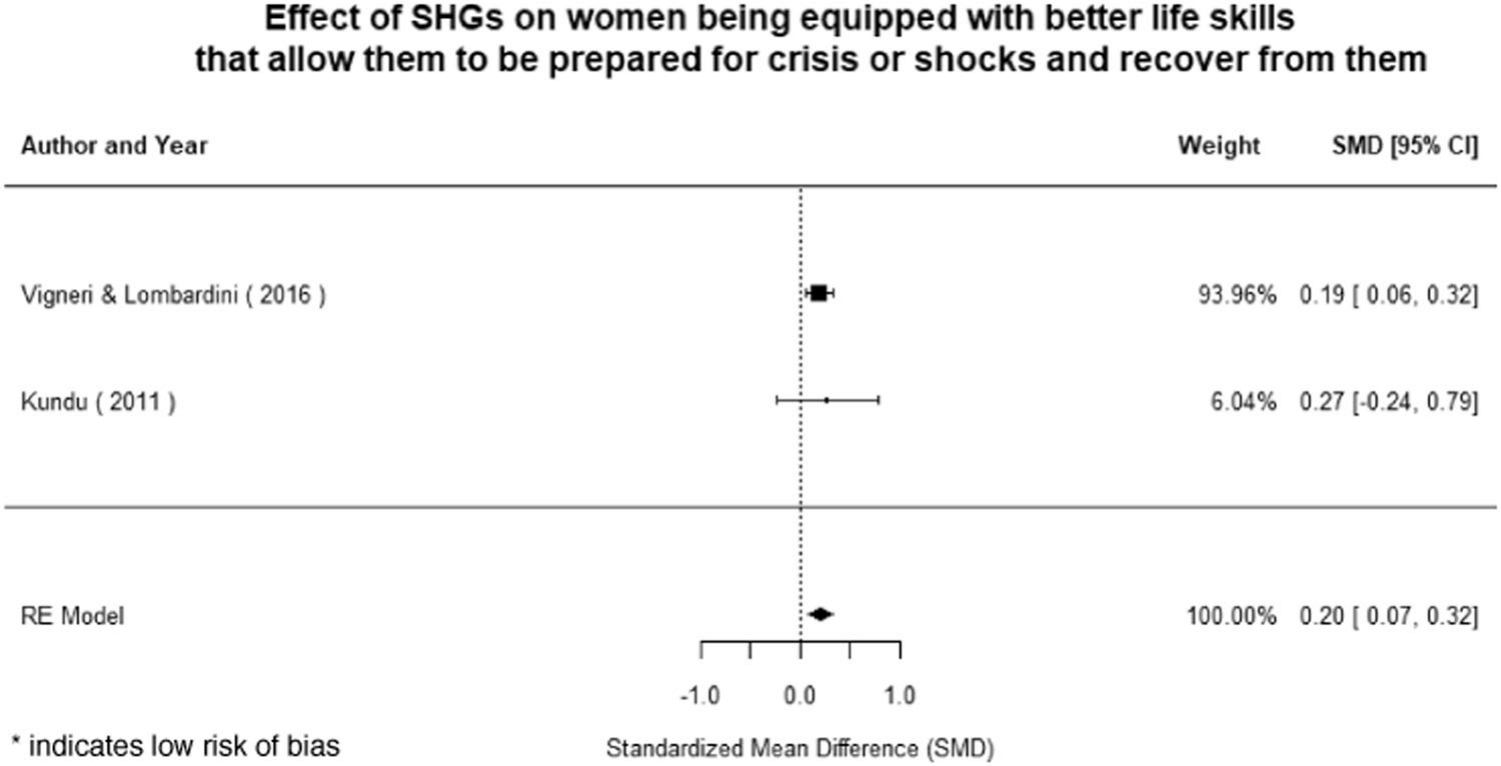
SHGBA7: Forest plot showing the observed outcomes and the estimate of the random‐effects model. CI, confidence interval; SHGs, self‐help groups

Two studies reported disaggregated data for women being equipped with better life skills that allow them to be prepared for crisis or shocks and recover from them, thus we included *k *=* *2 studies in the analysis. We assessed both of the studies as low risk of bias. The estimated average outcome based on the random‐effects model was $\hat{\mu }=0.1987$ (95% CI: 0.07to0.33). Therefore, the average outcome differed significantly from zero (z=3.0862, p=0.002). A forest plot showing the observed outcomes and the estimate based on the random‐effects model is shown in Figure 84 (SHG BA7). Given the small number of studies, this result should be interpreted with caution. According to the Q‐test, there was no significant amount of heterogeneity in the true outcomes (Q(1)=0.0893, p=0.77, ${\hat{\tau }}^{2}=0.00$, $I^{2}=0.00$%). With only two studies, and given there was no heterogeneity among the effects, moderator analyses were not possible, and tests of publication bias are not valid.

*Effect of SHGs on reduced instances of child or forced marriage*

Vigneri and Lombardini's (2017) quasi‐experimental study in Mali was the only study evaluating the impact of self‐help groups and VSLA on reduced instances of child or forced marriage. There was a very small, and not statistically significant, point estimate (*g *=* *0.03, [95% CI: −0.10 to 0.16]), and we assessed the study as having high risk of bias.

*Effect of SHGs on increased participation in decision making by women at the household or community level, including during crisis response*

**Figure 85:**
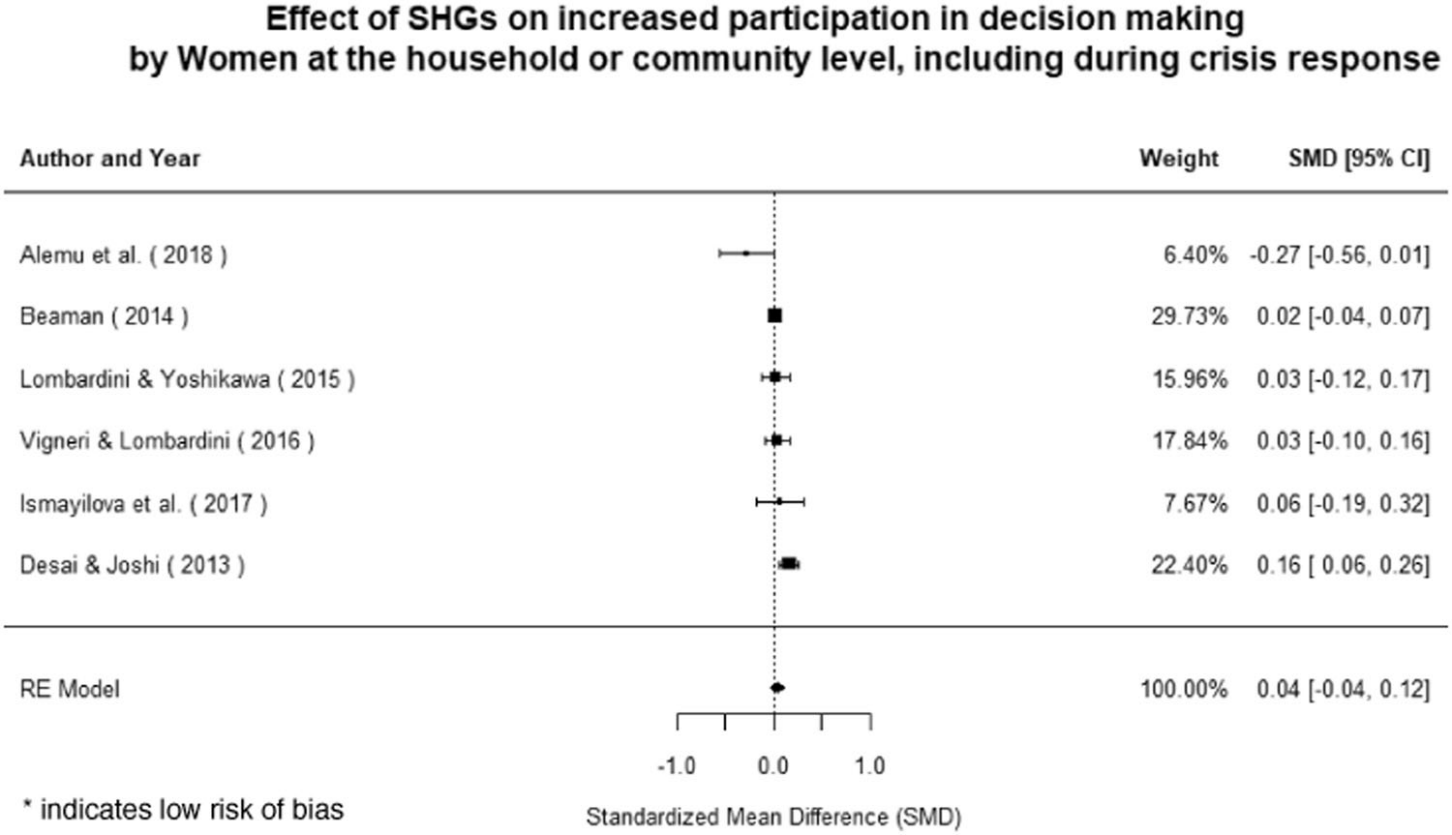
SHGBB1: Forest plot showing the observed outcomes and the estimate of the random‐effects model. CI, confidence interval; SHGs, self‐help groups

We included a total of k=6 studies in the analysis. We assessed none of the studies as low risk of bias, four as having some concerns, and two as low risk of bias. The observed outcomes ranged from −0.27 to 0.16. The estimated average outcome based on the random‐effects model was $\hat{\mu }=0.0$ 4 (95% CI: −0.04 to 0.12). Therefore, the average outcome did not differ significantly from zero (z=0.98, p=0.33). A forest plot showing the observed outcomes and the estimate based on the random‐effects model is shown in Figure 85 (SHGBB1).

According to the Q‐test, the true outcomes appear to be heterogeneous (Q(5)=11.43, p=0.04, ${\hat{\tau }}^{2}=0.00$, $I^{2}=56.24$%). An examination of the studentised residuals revealed that one study (Desai & Joshic, 2013) had a value larger than ±2.64 and may be a potential outlier in the context of this model. According to the Cook's distances, none of the studies could be considered to be overly influential. Sensitivity analyses leaving each study out indicated that removing Desai and Joshic (2013) would reduce the overall average effect ($\hat{\mu }\;=\;$0.01 [95% CI: −0.04 to 0.07]), but the effect would still be insignificant (*z *=* *0.51, p=0.61). No sources of heterogeneity were found to be significant through our moderator analyses.

*Effect of SHGs on women participating more in their community*

**Figure 86:**
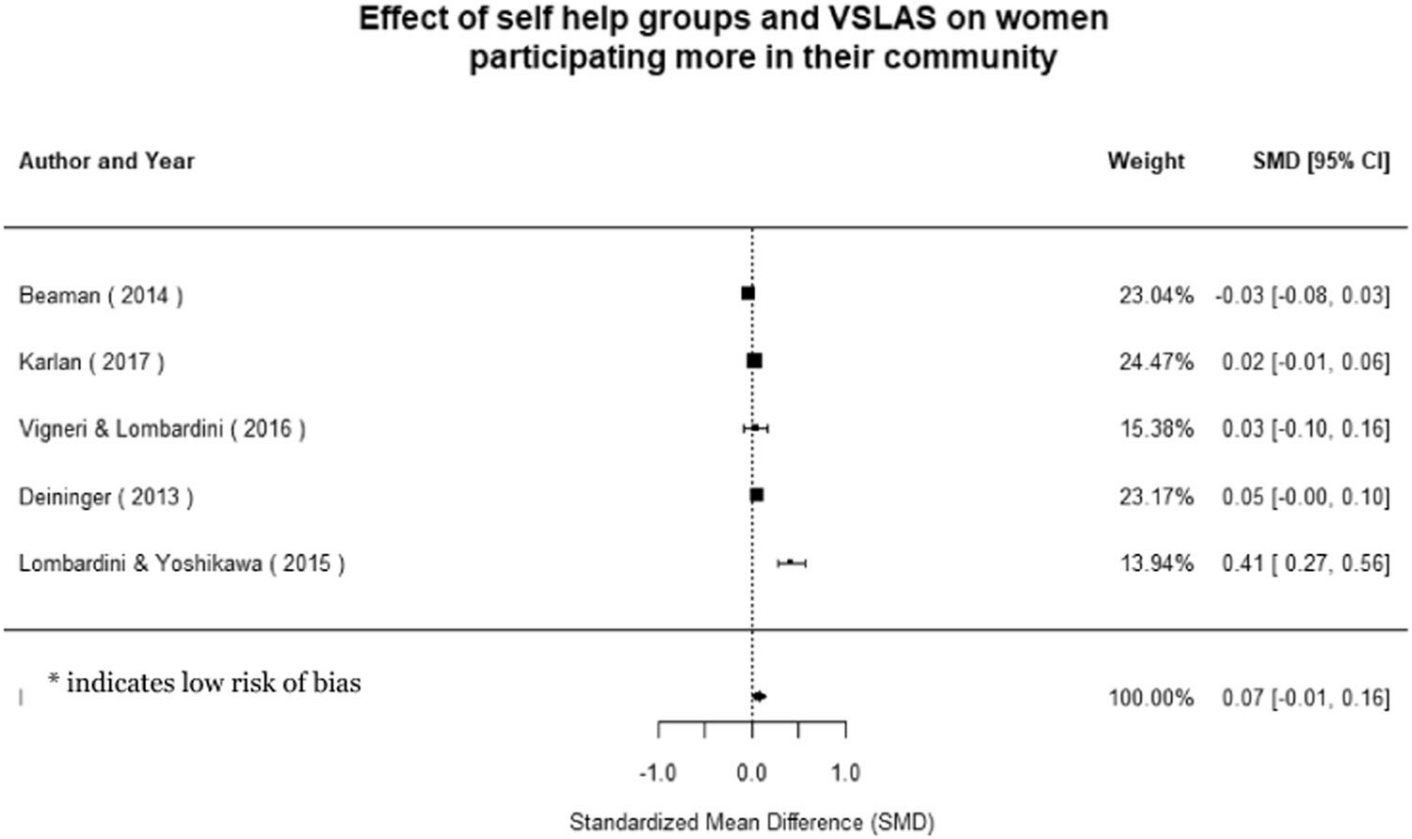
SHGBB2: Forest plot showing the observed outcomes and the estimate of the random‐effects model. CI, confidence interval; VSLAs, Village Savings and Loan Associations

We included a total of k=5 studies in the analysis. We assessed none of the studies as low risk of bias, three as having some concerns, and two as low risk of bias. The observed outcomes ranged from −0.03 to 0.41. The estimated average outcome based on the random‐effects model was $\hat{\mu }=0.0$ 7 (95% CI: −0.01 to 0.16). Therefore, the average outcome did not differ significantly from zero (z=1.81, p=0.07). A forest plot showing the observed outcomes and the estimate based on the random‐effects model is shown in Figure 86 (SHGBB2).

According to the Q‐test, the true outcomes appear to be heterogeneous (Q(4)=31.85, p<0.01, ${\hat{\tau }}^{2}$ = 0.01, $I^{2}=87.44$%). An examination of the studentised residuals revealed that one study (Lombardini & Yoshikawa, 2015) had a value larger than ±2.58 and may be a potential outlier in the context of this model. According to the Cook's distances, none of the studies could be considered to be overly influential. Sensitivity analyses leaving each study out indicated that removing Lombardini and Yoshikawa (2015) would reduce the average effect ($\hat{\mu }=0.0187$ [95% CI: −0.01 to 0.05]), but the effect would still be insignificant (*z *=** 1.12, p=0.26).

We were able to test several moderators in the context of this model. Studies with a high risk of bias were significantly different than studies with some concerns related to risk of bias ($\hat{B}=0.20,p=0.01$ [95% CI: 0.04 to 0.35]), such that high risk of bias studies had higher effects than studies with some concerns by 0.20 standard deviation units. Exposure to intervention, publication year and post‐intervention versus change from baseline were not significant moderators. There was not sufficient data to test evaluation period or whether the model was adjusted for covariates.

*Effect of SHGs on power holders having improved awareness and responsiveness to the demands, claims, rights and inputs of women*

Lombardini and Yoshikawa's (2015) quasi‐experimental study in Uganda was the only study evaluating the impact of self‐help groups and VSLA on power holders having improved awareness and responsiveness to the demands, claims, rights and inputs of women. There was a very small, and not statistically significant, point estimate (*g *=* *0.09, [95% CI: −0.06, 0.23]), and we assessed the study as having high risk of bias.

*Effect of SHGs on representation of women in local and subnational civil and political processes, including during peacebuilding and post conflict restoration*

**Figure 87:**
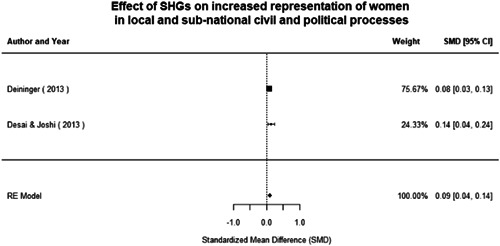
SHGCA2: Forest plot showing the observed outcomes and the estimate of the random‐effects model. CI, confidence interval; SHGs, self‐help groups

Two studies reported disaggregated data for representation of women in local and subnational civil and political processes, including during peacebuilding and post conflict restoration, thus we included *k *=* *2 studies in the analysis. We assessed both studies as having some concerns of risk of bias. The estimated average outcome based on the random‐effects model was $\hat{\mu }=0.09$ (95% CI: 0.04to0.14). Therefore, the average outcome differed significantly from zero (z=3.5987, p=0.0003). A forest plot showing the observed outcomes and the estimate based on the random‐effects model is shown in Figure 87 (SHGCA2). Given the small number of studies, this result should be interpreted with caution. According to the Q‐test, there was no significant amount of heterogeneity in the true outcomes (Q(1)=1.1089, p=0.2923, ${\hat{\tau }}^{2}=0.00$, $I^{2}=9.82$%). With only two studies, and given there was no heterogeneity among the effects, moderator analyses were not possible, and tests of publication bias are not valid.

*Effect of SHGs on effective prevention strategies supported to end violence against women and girls*

**Figure 88:**
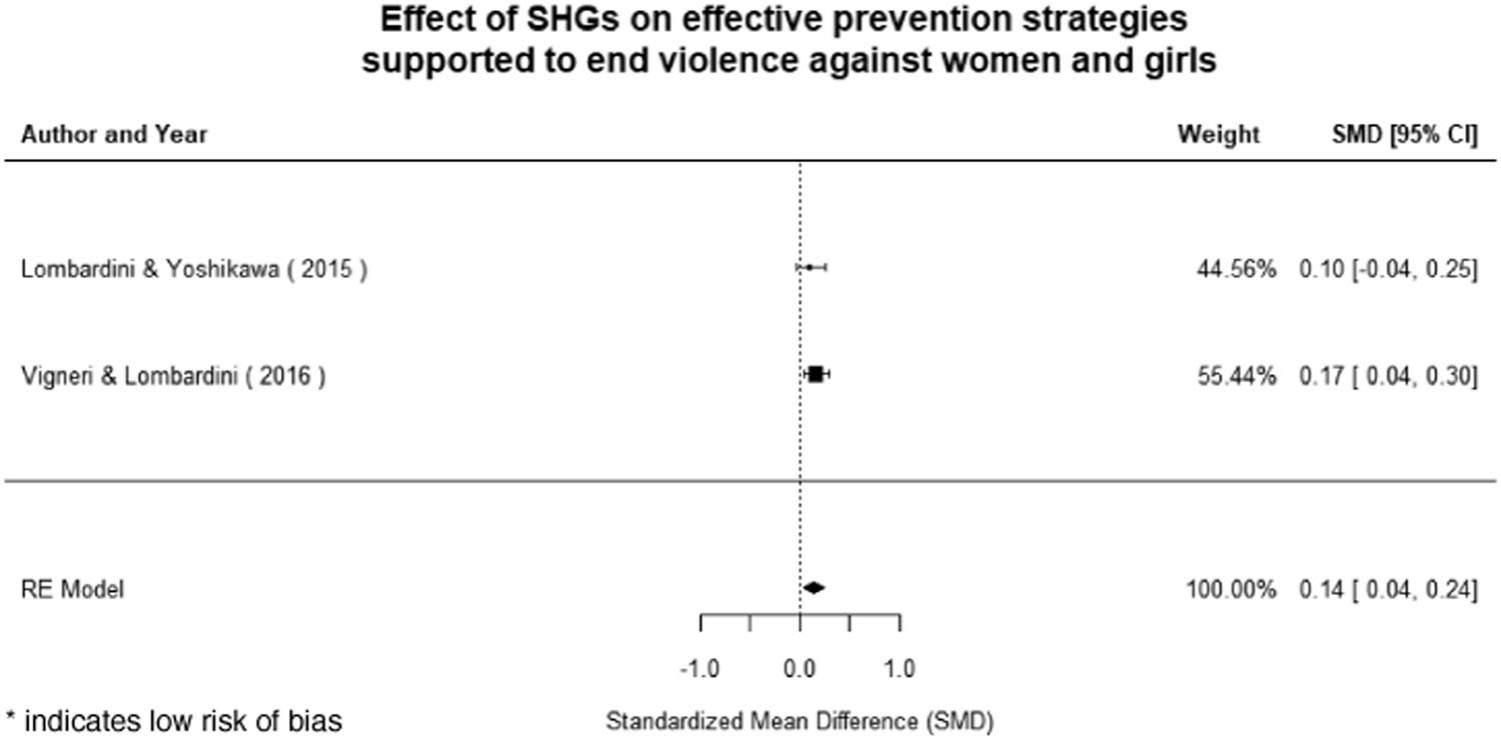
SHGCA6: Forest plot showing the observed outcomes and the estimate of the random‐effects model. CI, confidence interval; SHGs, self‐help groups

Two studies reported disaggregated data for effective prevention strategies supported to end violence against women and girls, thus we included *k *=* *2 studies in the analysis. We assessed both studies as high risk of bias. The estimated average outcome based on the random‐effects model was $\hat{\mu }=0.1389$ (95% CI: 0.04to0.24). Therefore, the average outcome differed significantly from zero (z=2.8084, p=0.005). A forest plot showing the observed outcomes and the estimate based on the random‐effects model is shown in Figure 88 (SHGCA6). Given the small number of studies, this result should be interpreted with caution. According to the Q‐test, there was no significant amount of heterogeneity in the true outcomes (Q(1)=0.3792, p=0.538, ${\hat{\tau }}^{2}=0.00$, $I^{2}=0.00$%). With only two studies, and given there was no heterogeneity among the effects, moderator analyses were not possible, and tests of publication bias are not valid.

*Effect of SHGs on relief and recovery initiatives in conflict and post conflict situations respond to the needs of women and girls, especially vulnerable groups*

Vigneri and Lombardini's (2017) quasi‐experimental study in Mali was the only study evaluating the impact of self‐help groups and VSLA on relief and recovery initiatives in conflict and post conflict situations respond to the needs of women and girls, especially vulnerable groups. There was a small, and statistically significant, effect (*g *=* *0.19, [95% CI: 0.06 to 0.32]), and we assessed the study as having high risk of bias.

*Effect of SHGs on communities having a more positive attitude towards women/marginalised groups*

**Figure 89:**
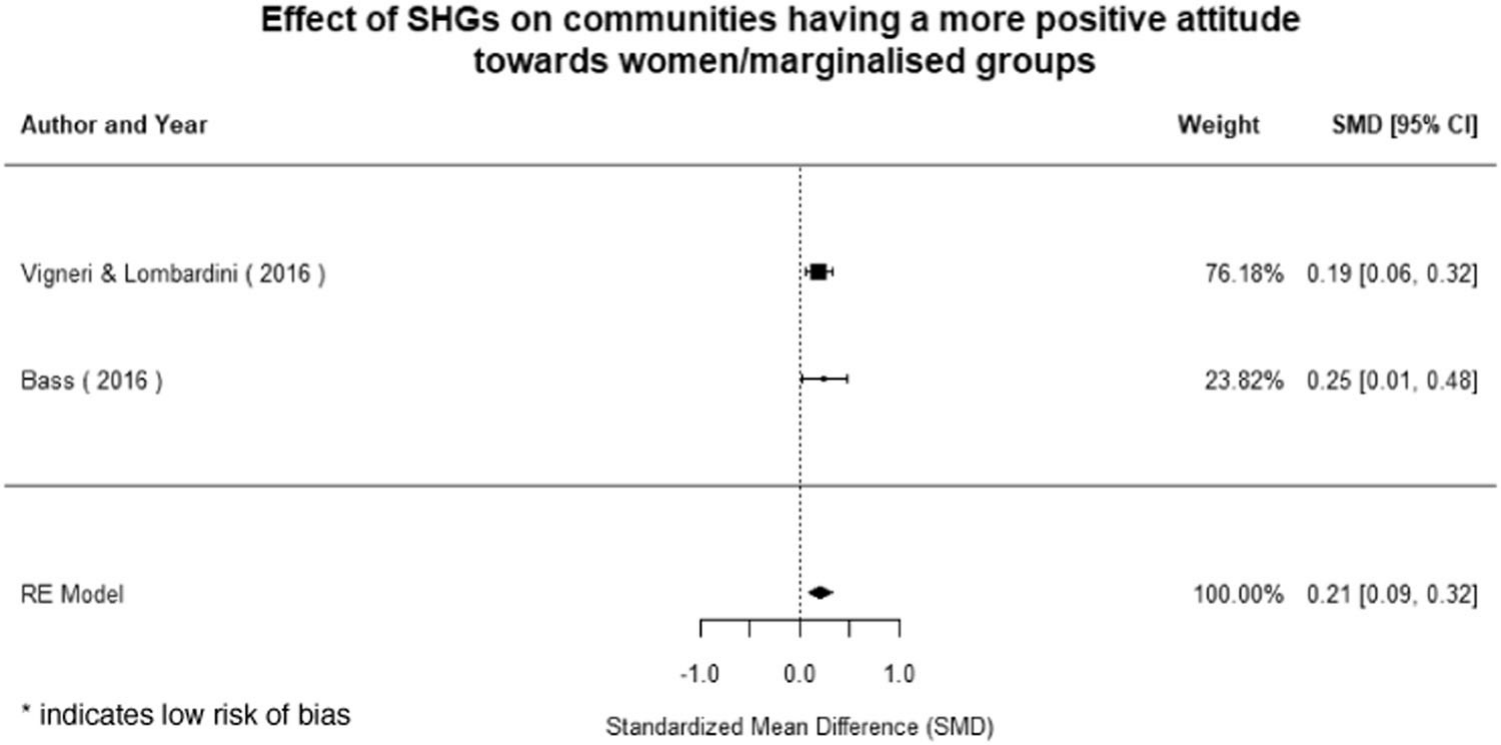
SHGCB2: Forest plot showing the observed outcomes and the estimate of the random‐effects model. CI, confidence interval; SHGs, self‐help groups

Two studies reported disaggregated data for having a more positive attitude towards women/marginalised groups, thus we included *k *=* *2 studies in the analysis. We assessed one of the studies as having some concerns of risk of bias and the other as high risk of bias. The estimated average outcome based on the random‐effects model was $\hat{\mu }=0.2066$ (95% CI: 0.09to0.32). Therefore, the average outcome differed significantly from zero (z=3.5625, p=0.0004). A forest plot showing the observed outcomes and the estimate based on the random‐effects model is shown in Figure 89 (SHGCB2). Given the small number of studies, this result should be interpreted with caution. According to the Q‐test, there was no significant amount of heterogeneity in the true outcomes (Q(1)=0.1534, p=0.6953, ${\hat{\tau }}^{2}=0.00$, $I^{2}=0.00$%). With only two studies, and given there was no heterogeneity among the effects, moderator analyses were not possible, and tests of publication bias are not valid.

*Effect of SHGs on women having improved attitudes, self‐image and confidence*

**Figure 90:**
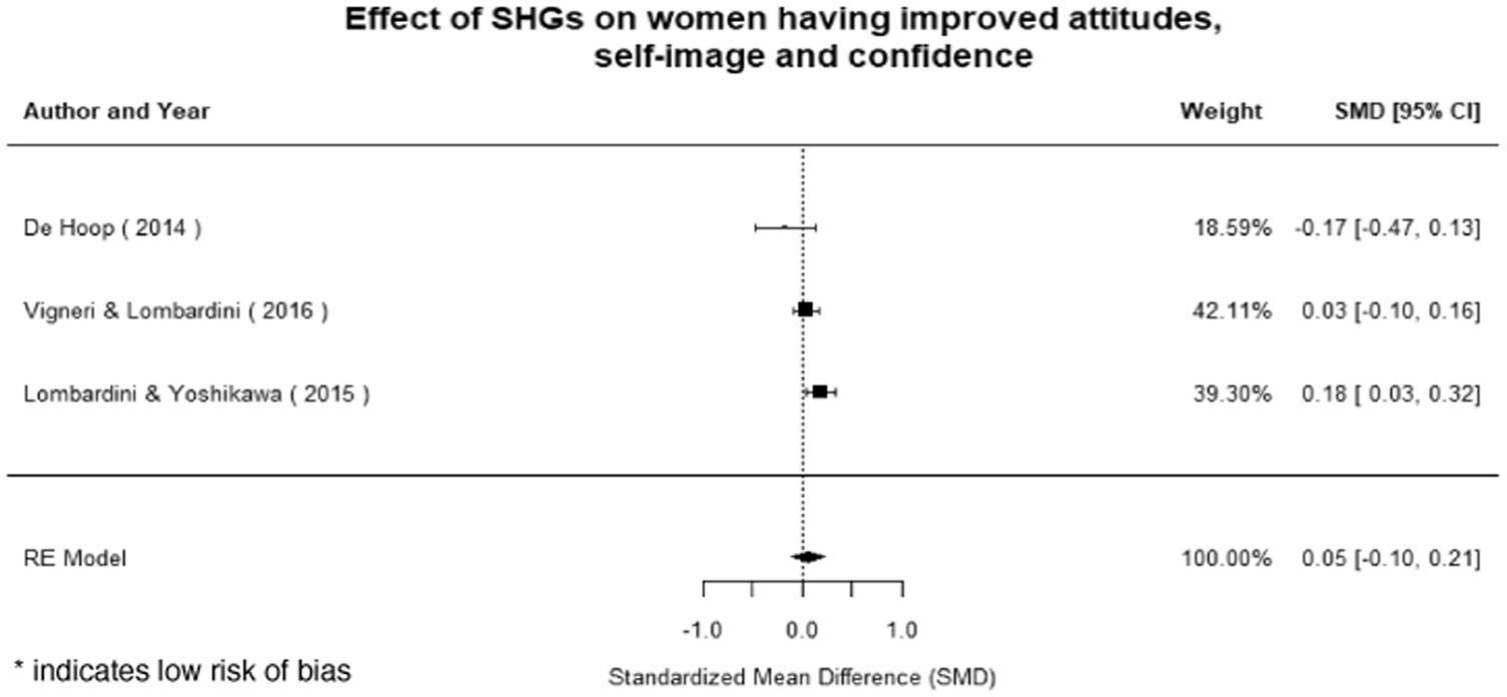
SHGCB3: Forest plot showing the observed outcomes and the estimate of the random‐effects model. CI, confidence interval; SHGs, self‐help groups

We included a total of k=3 studies in the analysis. We assessed none of the studies as low risk of bias, one as some concerns, and two as high risk of bias. The observed outcomes ranged from −0.17 to 0.18, with the majority of estimates being positive (67%). The estimated average outcome based on the random‐effects model was $\hat{\mu }=0.05$ (95% CI: −0.10 to 0.21). Therefore, the average outcome did not differ significantly from zero (z=0.66, p=0.51). A forest plot showing the observed outcomes and the estimate based on the random‐effects model is shown in Figure 90 (SHGCB3).

According to the Q‐test, there was no significant amount of heterogeneity in the true outcomes (Q(2)=4.83, p=0.09, ${\hat{\tau }}^{2}=0.01$, $I^{2}=58.60$%). An examination of the studentised residuals revealed that none of the studies had a value larger than ±2.39 and hence there was no indication of outliers in the context of this model. According to the Cook's distances, none of the studies could be considered to be overly influential. With only three studies, moderator analyses were not appropriate.

*Effect of SHGs on attitudes and support for women's economic, social and human rights by men, household and family members and community members*

Alemu and colleagues' (2018) quasi‐experimental study in Ethiopia was the only study evaluating the impact of self‐help groups and VSLA on improved attitudes and increased support for women's economic, social and human rights by men, household and family members and community members. Their report included 12 effects that fell into this outcome category (e.g., women should be more involved in politics). The effects ranged from medium, negative point estimates (*g *=* *−0.25, [95% CI: −0.53 to 0.04]) to large, positive effects (*g *=* *2.01, [95% CI: 1.70 to 2.33]). We assessed the study as having some risk of bias concerns.

*Effect of SHGs on decreased violence/discrimination at the household level*

**Figure 91:**
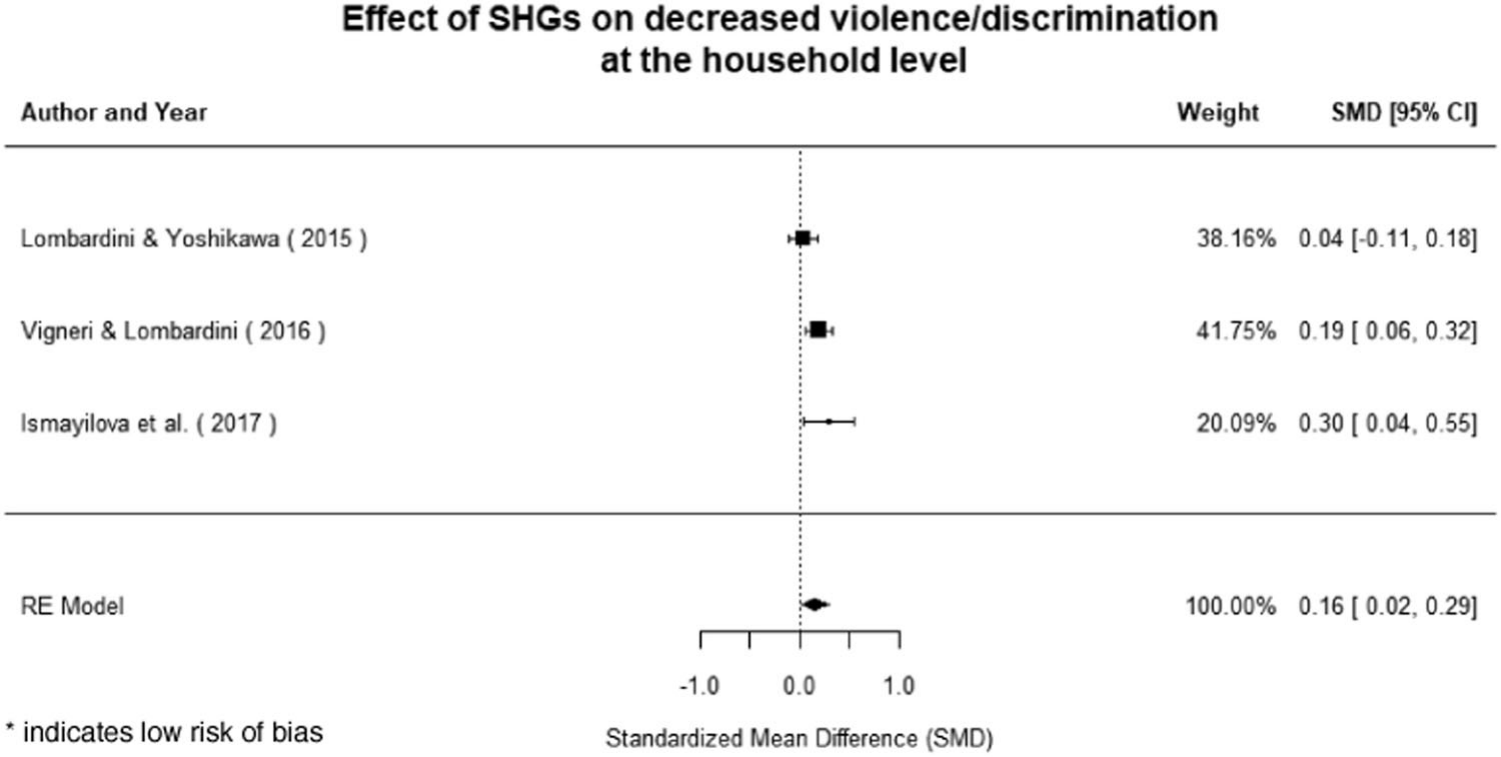
SHGCB5: Forest plot showing the observed outcomes and the estimate of the random‐effects model. CI, confidence interval; SHGs, self‐help groups

We included a total of k=3 studies in the analysis. We assessed none of the studies as low risk of bias, one as some concerns, and two as high risk of bias. The observed outcomes ranged from 0.04 to 0.30. The estimated average outcome based on the random‐effects model was $\hat{\mu }=0.16$ (95% CI: 0.02 to 0.29). Therefore, the average outcome differed significantly from zero (z=2.25, p=0.02). A forest plot showing the observed outcomes and the estimate based on the random‐effects model is shown in Figure 91 (SHGCB5).

According to the Q‐test, there was no significant amount of heterogeneity in the true outcomes (Q(2)=3.95, p=0.14, ${\hat{\tau }}^{2}=0.01$, $I^{2}=49.36$%). An examination of the studentised residuals revealed that none of the studies had a value larger than ±2.39 and hence there was no indication of outliers in the context of this model. According to the Cook's distances, none of the studies could be considered to be overly influential. With only three studies, moderator analyses were not appropriate.

*Effect of self‐help groups on safer and more secure household, communities and areas/territories for women, girls, men and boys*

Bass et al.'s (2016) experimental study in the Democratic Republic of Congo was the only study evaluating the impact of self‐help groups and VSLA on safer and more secure household, communities and areas/territories for women, girls, men and boys. There was a small, but not statistically significant, point estimate (*g *=* *0.19, [95% CI: −0.05 to 0.42]), and we assessed the study as having some risk of bias concerns.

*Effect of SHGs on improved quality of relationships between women and their household and community members*

**Figure 92:**
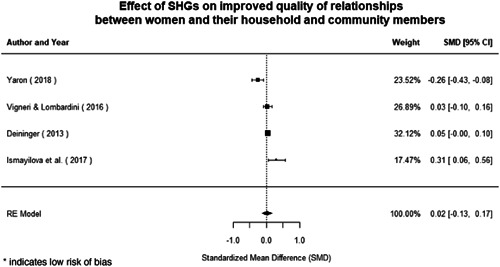
SHGCB8: Forest plot showing the observed outcomes and the estimate of the random‐effects model. CI, confidence interval; SHGs, self‐help groups

We included a total of k=4 studies in the analysis. We assessed none of the studies as low risk of bias, three as some concerns, and one as high risk of bias. The observed outcomes ranged from −0.26 to 0.31 (75%). The estimated average outcome based on the random‐effects model was $\hat{\mu }=0.02$ (95% CI: −0.13 to 0.17). Therefore, the average outcome did not differ significantly from zero (z=0.24, p=0.81). A forest plot showing the observed outcomes and the estimate based on the random‐effects model is shown in Figure 92 (SHGCB8).

According to the Q‐test, the true outcomes appear to be heterogeneous (Q(3)=15.89, p<0.01, ${\hat{\tau }}^{2}=0.02$, $I^{2}=81.12$%). An examination of the studentised residuals revealed that one study (Yaron et al., 2018) had a value larger than ±2.50 and may be a potential outlier in the context of this model. According to the Cook's distances, none of the studies could be considered to be overly influential. Sensitivity analyses leaving each study out indicated that removing Yaron et al., (2018) would increase the average effect ($\hat{\mu }=0.08$ [95% CI: −0.02 to 0.18]), but the effect would still be insignificant (*z *=** 1.52, p=0.13).

We were able to examine moderation by exposure to intervention, evaluation period, study design, publication year, adjustment for covariates, GII and FSI, but none were significant predictors of improved quality of relationships between women and their household and community members.

*Effect of SHGs on women's empowerment index*

**Figure 93:**
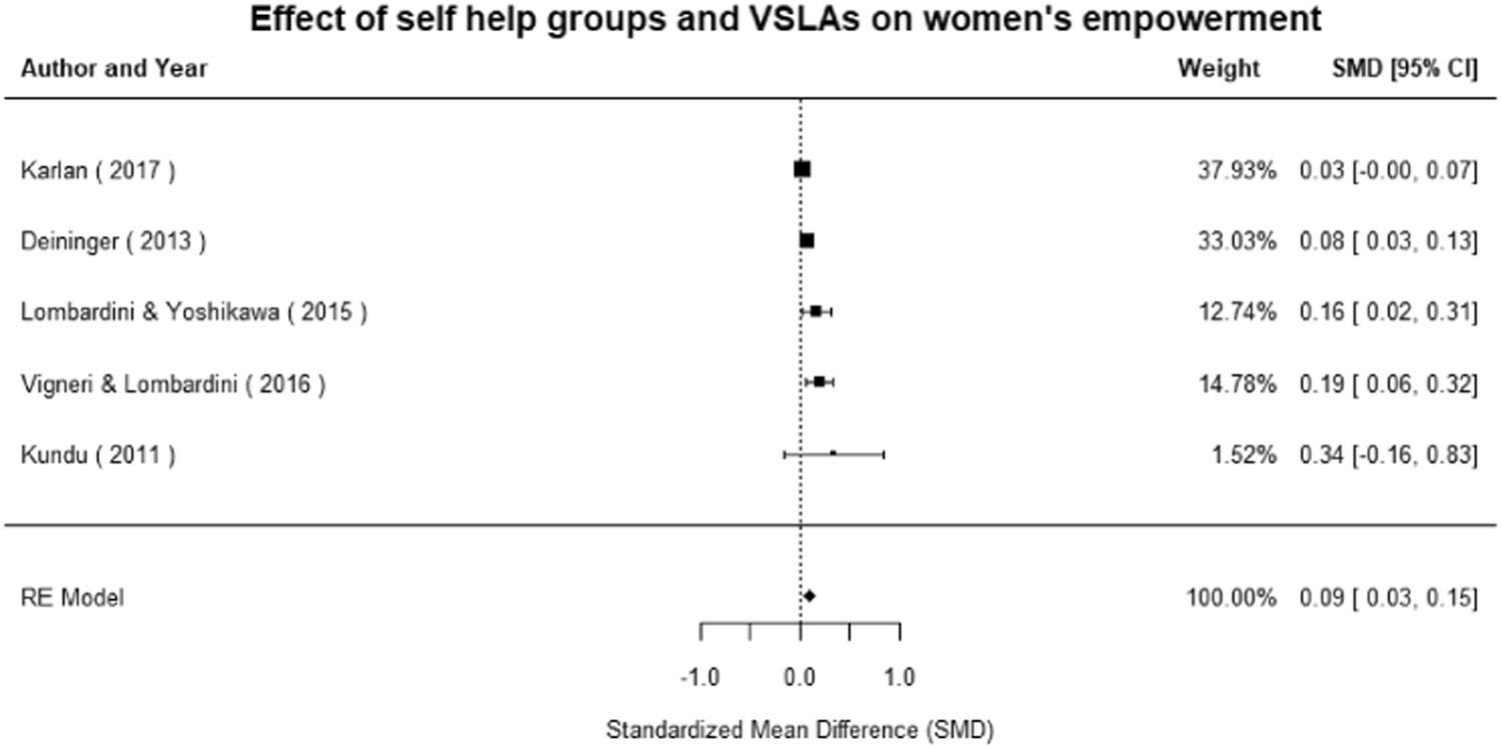
SHGCC1: Forest plot showing the observed outcomes and the estimate of the random‐effects model. CI, confidence interval; VSLAs, Village Savings and Loan Associations

We included a total of k=5 studies in the analysis. We assessed none of the studies as low risk of bias, two as some concerns, and three as high risk of bias. The observed outcomes ranged from 0.03 to 0.34. The estimated average outcome based on the random‐effects model was $\hat{\mu }=0.09$ (95% CI: 0.03 to 0.15). Therefore, the average outcome differed significantly from zero (z=2.95, p<0.01). A forest plot showing the observed outcomes and the estimate based on the random‐effects model is shown in Figure 93 (SHGCC1).

According to the Q‐test, the true outcomes appear to be heterogeneous (Q(4)=10.092, p=0.04, ${\hat{\tau }}^{2}$= 0.01, $I^{2}=60.35$%). An examination of the studentized residuals revealed that none of the studies had a value larger than ±2.58 and hence there was no indication of outliers in the context of this model. According to the Cook's distances, none of the studies could be considered to be overly influential.

We were able to test several moderators. Studies with a high risk of bias were significantly different than studies with some concerns related to risk of bias ($\hat{B}=0.14,p=0.01$ [95% CI: 0.04 to 0.24]), such that high risk of bias studies had higher effects than studies with some concerns by 0.14 standard deviation units. Exposure to intervention in months ($\hat{B}=0.004,p=0.022$ [95% CI: 0.0006 to 0.0079]) was significant, such that each additional month of exposure to the intervention increased the size of the effect by 0.004 standard deviation units. There were also several moderators that could not be tested in the context of this model. These included study design, evaluation period and whether the model was adjusted for covariates.

*Qualitative findings*

We conducted a thematic synthesis on the 12 linked qualitative studies to the included SHGs and VSLA interventions. As indicated above, this thematic synthesis aims to identify themes related to the interplay of intervention design, intervention implementation, target population, and contextual variables with intervention outcomes and effects. In total, we identified 17 descriptive themes, which we configured into four analytical themes (Supporting Information Appendix SN). These four analytical themes present the synthesis results and are discussed in more detail below.

*Theme 1: Training of leaders and members of self‐help groups and VSLAs is crucial to encourage uptake and the support of other groups and agents is valuable for conflict management*.

Self‐help groups and VSLAs can benefit from training of leadership and members in conjunction with agent and group support. Training activities within these groups was perceived to enhance confidence and trust in the leadership and capability among beneficiaries. For example, in the Saving for Change (SfC) programme in Mali, the participants accepted the programme because replicating agents were considered legitimate; this legitimacy was derived from the formal training and the received certificate d (IPA, 2013). The case of Mali's SfC programme clearly shows the importance of training replicating agents in building confidence among members as well as propelling the status and perception by women in the community. As a clear expression of this, one replicating agent in N'Gorosso Peul succeeded in forming a group but faced challenges in forming other groups in other villages because the agent had not received formal training. For people to believe and trust her, she stated that she needed a technical agent as an escort (IPA, 2013). According to the replicating agent's comments,

‘Everything depends on the TA’, she explained. ‘Women listen to the TA because he comes from outside the village and has more knowledge and has been to school. The women respect me but they do not always have confidence that my solutions to problems are the right ones. When an outsider speaks to them, they listen more’.

The replication agent also cited the technical agent's higher level of education and training as the major difference between them, as the community members were more likely to respect the authority of an educated ‘outsider’ more than a local. Another replicating agent from Nekeroro who had received the structured replication training remarked:

‘*Now that I can show women that I left the village and had training, they understand that I am better able to answer their questions when the TA is not here*’.

A CAEB coordinator in the SfC programme acknowledged that one of the biggest advantages of being part of the association is the advice from other groups on the handling of internal problems and pooling of resources for large‐scale projects. Group support is also seen to promote participation through trust building in the system of self‐help groups and VSLAs. In the SfC programme in Mali, there were groups that refused to join the association due to lock‐in costs. To garner interest, the agent brought women from different groups to visit and observe association meetings and report back to their respective groups (Bureau of Applied Research in Anthropology and IPA, 2008). These traditional modes of raising awareness, namely word‐of‐mouth and workshops, attracted non‐beneficiaries as stories of positive programme outcomes are shared in communities where saving activity improved, and borrowing was used positively (IPA, 2013).

Though there were mixed feelings by women from the SfC programme regarding the continued visits of agents, they appreciated the support, stating that they provided valuable help (Bureau of Applied Research in Anthropology and IPA, 2008). To ensure sustainability, there is a need to maintain continuous presence in the form of NGO agents, for instance, in areas where the programme is relatively new. These stakeholders can be important for resolving issues related to financial management of funds (Bureau of Applied Research in Anthropology and IPA, 2008). For instance, Mali, a president of an SfC group commented, ‘We need Tonus agents here. Everyone agrees on this point. Sometimes there are important misunderstandings about budget management or loan reimbursement that only they can resolve. An outside eye is needed. If there's a misunderstanding, or people don't fulfil their obligations, they need someone to come and explain. Most women rely on collecting and selling shea nuts to get income to pay their contribution or loans. This year there were not enough fruits and people didn't respect the repayment date. This is when someone from the outside is needed for arbitration’.

*Theme 2: Self‐help groups and VSLAs are an attractive and convenient option for women who are often excluded from meaningful economic participation but factors related to market access and market conditions can minimise the gains from these programmes*.

Due to the inclusive nature of self‐help groups and VSLAs, women who could not engage in prior saving and borrowing activities can be granted better opportunities for economic participation. Women from the SfC programme, for instance, reported appreciating the SfC loans as they had less stringent requirements (no need for collateral) than formal credit programmes (IPA, 2013). As noted in the Bureau of Applied Research in Anthropology and IPA (2008) study, poorer households who have fears of taking loans tend to use these programmes as a means of saving. Further evidence for the need to be inclusive to the vulnerable groups is found in this study, which notes that in some villages there are artisanal caste groups that are concentrated in the outlets but are excluded due to distances from the villages. Married women were perceived to be more influential because they enjoy greater social capital and labour access than unmarried women and widows. The former were able to take more risks and/or repay loans, which influenced the nature and extent of borrowing and saving activities (Bureau of Applied Research in Anthropology and IPA, 2008).

Access issues and environmental risk were a major concern for the reported success of self‐help groups and VSLAs, particularly in the African context. In Mali, for example, villages were constrained by access to markets and centres which increased transportation costs. The prices of produce and goods purchased also varied due to changes in local, national and international market forces. The potential consequence is the negative impact on profits from goods produced and the increase in cost of transporting purchased goods. This prevented women from engaging in some activities, such as diversifying their crops (Bureau of Applied Research in Anthropology and IPA, 2008). The absence of formal financial institutions in villages and the lack of trust in such organisations in some cases pushed rural households to have a general preference for investing their savings in animals and commerce rather than banking systems (IPA, 2013).

Lack of water for domestic use presents itself as a major observed challenge for women who then spend a substantial amount of time looking for water, especially in the dry season when many sources of water run dry (Bureau of Applied Research in Anthropology and IPA, 2008). In many of the villages, droughts severely affected crop yields whilst incidences of malaria and water borne diseases put a serious strain on savings and labour resources (Bureau of Applied Research in Anthropology and IPA, 2008; IPA, 2013).

*Theme 3: Male dominance in household decision making, cultural attitudes towards women's roles and mobility, religion, time constraints exert negative pressure towards gains associated with self‐help groups and VSLAs*.

Qualitative findings show that because of their greater financial contributions to the household through participating in self‐help groups and VSLAs, women can realise increases in household decision‐making power and sense of independence (Bermudez & Matuszeski, 2010; Concern Worldwide, 2017; Deubel & Boyer, 2020; Nair, 2012), reduced household‐level tension and marital conflicts (Bermudez & Matuszeski, 2010; Deubel & Boyer, 2020); IPA, 2013). In an Indian study by Nair (2012), for example, one respondent highlighted, ‘After I started working with SEWA, I was consulted for big financial decisions in my family. However, I make the small financial decisions of the family’—Staff F. In Mali, an animator (replication agent) summarised the increased independence trend, noting that ‘SfC has changed women. They can say yes or no now. Before women would always agree with what men said’ (Bermudez & Matuszeski, 2010).

Due to gender and cultural norms, men may dominate household decisions, including participation in savings groups, financial decisions and spousal mobility (Bureau of Applied Research in Anthropology and IPA, 2008; Deubel & Boyer, 2020) which impedes programme uptake. Consequently, women's empowerment is constrained within social bounds. For example, in Mali, some women from Kalifabougou highlighted that they had to ask permission from their husbands to join the savings groups (Bureau of Applied Research in Anthropology and IPA, 2008). In Zambia, findings from Concern Worldwide (2017) showed that some men were not supportive of women participating in community groups, which they saw as a waste of time, especially when women came back from training ‘empty handed’ (Concern Worldwide, 2017). Results from Nair (2012) capture the influence of in‐laws in making financial decisions in rural India, with one woman stating that:

‘Every family decision is made by the father‐in‐law and we have to follow. I save Rs. 500 ($9 USD) in my account and he asks me, what is the need. Any investment, saving or expenditure, we have to ask him’—Staff G.

Lack of support and time constraints resulting from gender skewed domestic workload prevented women from either participating or fully participating in self‐help groups and VSLAs (IPA, 2013; Nair, 2012). In rural Gujarat, India, women's primary role and responsibilities were household chores and taking care of family members. According to a respondent Staff S. from the Nair (2012) study, ‘Even if a woman is working and earning for the family, she is expected to keep up with the chores, or any small business by a family member’. Staff S. continues: ‘No, my husband nor any male family members do not contribute to domestic chores. It is completely my responsibility. I also have to contribute to his work and my contribution is hardly ever acknowledged’.

Another important factor that deters uptake of self‐help groups and VSLAs was religious beliefs and stigma regarding loans. For example, in Mali, strong religious interpretations effectively prevented women and households from taking loans outright (IPA, 2013). Traditionally, in some Malian cultures, getting loans from non‐relatives is considered shameful, though not forbidden (Bermudez & Matuszeski, 2010).

*Theme 4: Support from family members and partners can enhance programme participation and association rules improves group management. Domination of elites can stifle development of new leaders in the community whereas legislation can inhibit expansion*.

Family and partner support emerged as one of the critical factors in determining participation in self‐help groups and VSLAs g (Bermudez & Matuszeski, 2010; Bureau of Applied Research in Anthropology and IPA, 2008; Concern Worldwide, 2017; Nair, 2012). There is some evidence that, in India, lack of family support is one of the most difficult barriers to overcome in enhancing participation in self‐help groups and VSLAs. According to Nair (2012), women pointed out that one should not push their parents or other family members to support them too much: ‘It happens slowly, you have to give them time. You have to walk with all of them, you can't leave them behind. You have to take them all along with you. If we don't do so, SEWA's reputation will also be spoilt’. The support includes being allowed to move freely and conduct daily life activities to balance paid and domestic work. Partner support also becomes key even in the absence of general family support. Sentiments from one woman of the SEWA programme in India adequately captures this barrier: ‘I am married into a Muslim family and it is difficult for girls to progress in the community. If you are going out, you have to tell every member of the family. My husband is very supportive. My extended family and relatives dislike that I work’—Staff Y. In Mali, husbands of members assisted members with loan repayments in certain situations which had disempowered members from repaying loans, such as illness and business closure. In one household interview in Kalifabougou, an SfC member indicated her doubts about repaying her loan and expected her husband to assist with any outstanding balance to which the husband agreed (Bureau of Applied Research in Anthropology and IPA 2008).

Setting rules of participation by group members provided some comfort, enhanced inclusiveness and minimised chances of default by members. As captured in IPA (2013) regarding the SfC programme, one replicating agent Werekela explained that: ‘In our group, we set the rules about how much money will be contributed, how loans are taken and what will happen if someone does not repay. Therefore, we understand how to manage the group and there is less fear that if a woman borrows money, she will not understand the terms of how to repay it and will become indebted. This is what can happen when villagers use the caisse d'épargne. There are many rules and a woman who cannot read may also be afraid of participating when the rules are set by people she does not know’.

Three contextual factors in Mali's self‐help groups and VSLA groups affected the success of the SfC programme. First, the domination of elites. The advancement of existing leaders could potentially block the development of new female leaders in the community. There are cases where women in the villages who held prominent positions and generally wealthier assumed leadership of the SfC programme and exerted undue influence over other women from the village, such as in Bouugola (Bermudez & Matuszeski, 2010; Bureau of Applied Research in Anthropology and IPA, 2008). To avoid domination by village elites over time, instituting mandatory leadership rotation yearly or biannually would create a more democratic process of leadership change and increase the capacity‐building component of the programme in general (Bureau of Applied Research in Anthropology and IPA 2008). Second, group conflicts can emanate from disagreements on the application of rules or general failure to meet obligations. Such conflicts resulted in women exiting the SfC programme (Bermudez & Matuszeski, 2010). Lastly, the legislative framework was restrictive. The government of Mali generally forbids informal microfinance programmes though it allowed the SfC programme under the close watch of government officials. This legal grey area can have limited programme expansion and thereby curtailed programme reach (Bureau of Applied Research in Anthropology and IPA 2008).

*Discussion*

A total of 11 studies were included in the quantitative analysis (*n* = 11). Quantitative meta‐analysis showed positive results for self‐help groups and VSLAs across a number of outcomes including: (1) women's capacity to understand and use financial, banking and business services effectively, (2) women being more aware of their rights and the roles and responsibilities of duty bearers, (3) women having more positive attitude towards taking action to claim their rights and (4) reduced percentage of women agreeing with certain reasons that justify violence against women and girls.

Some possible pathways from the qualitative evidence base that may explain these quantitative observations are:

- Training self‐help group and VSLA members can enhance financial literacy and business skills. Successfully sharing of adequate and relevant knowledge and skills between group members can be beneficial to group replication as well.
- Self‐help groups and VSLAs can adopt traditional methods of raising awareness such as allowing prospective members to listen in on meetings, word‐of‐mouth and workshops to motivate responsible and informed participation and use of financial instruments.
- Social capital gains and the acquisition of skills from self‐help groups and VSLAs seem to drive economic participation (including the effective use of loans and saving and labour participation).

*So what is so special about self‐help groups and SHGs?*

Here are some potentially important characteristics and their justification:

- Consolidating results from both our quantitative and qualitative analyses, self‐help groups and VSLAs seem to foster more participatory methods of engagement that can increase the confidence of women.
- Activities of self‐help groups and VSLAs often challenge existing gender roles and norms by design and include women in activities that they were traditionally excluded from—this can provide a pathway for positive empowerment gains.
- Empowering women to lead can improve their views on self‐worth and motivate them to take ownership of their rights.
- Clear rules of participation and communication of programme activities possibly increase adherence (repayments) and willingness to participate for beneficiaries.
- Factors that could limit these positive effects:
  o Legislation and compliance obligations as well as domination by smaller groups.
  o Intergroup conflict and control by a few elite leaders within the beneficiary pool.
  o Partner reluctance to support programme women on their daily activities due to pervasive gender norms and roles.

##### 5.3.7.5 Summary of findings and discussion

We included 12 studies across seven countries in South Asia and Sub‐Saharan Africa that evaluated the effect of SHGs and VSLAs. The story told by the quantitative evidence on the effect of SHGs and VSLAs is an overwhelmingly positive one. We found moderate positive and significant impacts of SHGs and VSLAs on increased capacity of women to understand and use financial, banking and business services effectively. We found additional small but significant positive impacts on women's increased access to and ownership of assets, credit and income, women's access to decent work, women being more aware of their rights and the roles and responsibilities of duty bearers, better life skills for women, effective prevention strategies against violence being supported, decreased violence/discrimination at the household level, and increased women's empowerment. There were also positive but nonsignificant effects on increased freedom of movement and association, women having more positive attitudes towards taking action to claim their rights, increased participation in decision making by women at the household or community level, women participating more in their communities, communities having more positive attitudes towards women, and women having improved attitudes/self‐image/confidence. There were two negative but nonsignificant summary effects, which were on reduced percentages of women agreeing with reason justifying violence against women, as well as improved quality of relationships between women and their household/community members.

The body of evidence in this intervention category also allowed for us to test several moderators. While there were no ubiquitous patterns among these moderators, we did find lower effects on assets, credit and income in India than we did in other countries. For impacts on freedom of movement and association, there were larger effects for RCTs than there were for QEDs, and smaller effects in more gender inequitable places. Women's decision making was moderated by exposure to intervention (in months) such that longer interventions achieved larger effects. Effects on decision making by women were also smaller in more recent studies. Women's participation in their communities was moderated by study quality such that studies with high risk of bias reported larger effects than those with some concerns related to risk of bias. Finally, longer interventions also achieved larger effects on women's empowerment, and there were smaller effects reported for women's empowerment in more recent studies. Overall, the GRADE assessments generally indicate a very low to low (with one moderate) certainty in this body of evidence.

Between the 12 quantitative studies and the 10 linked qualitative studies, we have a sufficient evidence base to support the outcomes observed. Through four different analytical themes, several contextual and implementation themes emerged when looking at SHGs and VSLAs. Prevailing gender norms and household structures can impede the ability of women to participate and actively challenge these norms that have prevented them from economic and social mobility. SHGs and VSLAs can exist outside or in conjunction with. The summary of our quantitative findings along with the GRADE certainty of evidence ratings are presented in Table 36 (Table 31).

**Table 31:** GRADE summary of findings and certainty of evidence on SHGs and VSLAs

Certainty assessment							Sample size	Effect	Certainty	Importance
No. of studies	Study design	Risk of bias	Inconsistency	Indirectness	Imprecision	Other considerations		Absolute (95% CI)		
*(AA1) Women have access to rights, services and opportunities*										
1	QED	Very serious^a^	Serious^b^	Not serious	Not serious	None	728	SMD 0.18 SD higher (0.03 higher to 0.32 higher)	⊕◯◯◯ VERY LOW	Limited importance
*(AB1) Capacity of women to understand and use financial, banking, and business services effectively*										
3	RCT‐3	Serious^c^	Serious^d^	Not serious	Not serious	None	8642	SMD 0.24 SD higher (0.09 to 0.39 higher)	⊕⊕◯◯ LOW	Important, but not critical
*(AB2) Women have increased access and ownership to assets, credit and income*										
7	RCT‐4 QED‐3	Very serious^e^	Serious^f^	Not serious	Not serious	None	12,521	SMD 0.07 SD higher (0.00 higher to 0.13 higher)	⊕◯◯◯ VERY LOW	Important, but not critical
*(AB3) Women and girls have equitable access to livelihood support services*										
1	QED	Serious^g^	Serious^b^	Not serious	Serious^h^	None	192	SMD 0.31 SD higher (0.03 higher to 0.6 higher)	⊕◯◯◯ VERY LOW	Important, but not critical
*(AB5) Durable and reliable housing for vulnerable populations, including women and girls*										
1	QED	Serious^i^	Serious^b^	Not serious	Not serious	Strong association	1046	SMD 0.39 SD higher (0.28 higher to 0.56 higher)	⊕⊕⊕◯ MODERATE	Important, but not critical
*(AC1) More women engaged in other micro, small, and medium‐sized enterprises*										
1	RCT	Serious^j^	Serious^b^	Not serious	Not serious	None	8595	SMD 0.03 SD higher (0.07 lower to 0.07 higher)	⊕⊕◯◯ LOW	Important, but not critical
*(AC2) Initiatives supported that facilitate women to access decent work (formal and informal employment), including people with disabilities*										
3	RCT‐2 QED‐1	Serious^k^	Not serious	Not serious	Not serious	None	3632	SMD 0.13 SD higher (0.04 higher to 0.22 higher)	⊕⊕⊕◯ MODERATE	Important, but not critical
*(AC3) Improved capacity of women entrepreneurs*										
1	QED	Very serious^l^	Serious^b^	Not serious	Not serious	None	728	SMD 0.19 SD higher (0.05 higher to 0.33 higher)	⊕◯◯◯ VERY LOW	Important, but not critical
*(BA2) Women have more and better control over their bodies and sexual health*										
1	QED	Very serious^m^	Serious^b^	Not serious	Serious^n^	None	728	SMD 0.01 SD lower (0.16 lower to 0.13 higher)	⊕◯◯◯ VERY LOW	Critical
*(BA3) Women have increased freedom of movement and association*										
7	RCT‐4 QED‐3	Very serious^o^	Serious^p^	Not serious	Serious^q^	None	14,316	SMD 0.18 SD higher (0.05 lower to 0.31 higher)	⊕◯◯◯ VERY LOW	Limited importance
*(BA4) Women are more aware of their rights and the roles and responsibilities of duty bearers*										
3	RCT‐1 QED‐2	Very serious^r^	Not serious	Not serious	Not serious	None	4793	SMD 0.1 SD higher (0.02 higher to 0.18 higher)	⊕⊕◯◯ LOW	Limited importance
*(BA5) Women have more positive attitude towards taking action to claim their rights*										
3	RCT‐1 QED‐2	Very serious^s^	Very serious^t^	Not serious	Not serious	Strong association	4257	SMD 0.58 SD higher (0.03 higher to 1.14 higher)	⊕◯◯◯ VERY LOW	Limited importance
*(BA6) Reduced percentage of women agreeing with certain reasons that justify violence against women and girls*										
3	QED‐3	Very serious	very serious	Not serious	Serious	None	1827	SMD 0.23 lower (0.57 lower to 0.12 lower)	⊕◯◯◯ VERY LOW	Critical
*(BA7) Women are equipped with better life skills that allow them to be prepared for crisis or shocks and recover from them*										
2	QED‐2	Very serious^u^	Serious^w^	Not serious	Not serious	None	5192	SMD 0.16 SD higher (0.08 higher to 0.23 higher)	⊕◯◯◯ VERY LOW	Important, but not critical
*(BA8) Reduced instances of child or forced marriage*										
1	QED	Very serious^v^	Serious^b^	Not serious	Not serious	None	907	SMD 0.03 SD higher (0.1 lower to 0.16 higher)	⊕◯◯◯ VERY LOW	Critical
*(BB1) Increased participation in decision making by Women at the household or community level, including during crisis response*										
6	RCT‐3 QED‐3	Very serious^w^	Very serious^x^	Not serious	Serious^y^	None	14,186	SMD 0.04 SD higher (0.04 lower to 0.12 higher)	⊕◯◯◯ VERY LOW	Limited importance
*(BB2) Women participate more in their community*										
4	RCT‐2 QED‐2	Very serious^z^	Very serious^aa^	Not serious	Not serious	None	9132	SMD 0.07 SD higher (0.01 lower to 0.16 higher)	⊕◯◯◯ VERY LOW	Limited importance
*(CA1) Power holders have improved awareness and responsiveness to the demands, claims, rights and inputs of women*										
1	QED	Very serious^ab^	Serious^b^	Not serious	Not serious	None	728	SMD 0.09 SD higher (0.02 lower to 0.21 higher)	⊕◯◯◯ VERY LOW	Limited importance
*(CA2) Increased representation of women in local and subnational civil and political processes, including during peacebuilding and post conflict restoration*										
2	RCT‐1 QED‐1	Serious^ac^	Not serious	Not serious	Not serious	None	3205	SMD 0.09 SD higher (0.04 higher to 0.14 higher)	⊕⊕⊕◯ MODERATE	Limited importance
*(CA6) Effective prevention strategies supported to end violence against women and girls*										
2	QED‐2	Very serious^ad^	Not serious	Not serious	Not serious	None	1635	SMD 0.14 SD higher (0.04 higher to 0.24 higher)	⊕⊕◯◯ LOW	Critical
*(CA8) Increased community support for women's and children's human, economic and legal rights*										
1	QED	Serious^ae^	Serious^b^	Not serious	Not serious	None	907	SMD 0.19 SD higher (0.06 higher to 0.32 higher)	⊕⊕◯◯ LOW	Important, but not critical
*(CB2) Communities have a more positive attitude towards women/marginalised groups*										
2	RCT‐1 QED‐1	Very serious^af^	Not serious	Not serious	Serious^ag^	None	4726	SMD 0.21 SD higher (0.09 lower to 0.32 higher)	⊕◯◯◯ VERY LOW	Limited importance
*(CB3) Women have improved attitudes, self‐image and confidence*										
3	QED‐3	Very serious^ah^	Serious^ai^	Not serious	Serious^aj^	None	1635	SMD 0.1 SD higher (0.04 lower to 0.24 higher)	⊕◯◯◯ VERY LOW	Limited importance
*(CB4) Improved attitudes and increased support for women's economic, social and human rights by men, household and family members and community members*										
1	QED	Serious^ak^	Serious^b^	Not serious	Very serious^al^	None	192	Seven positive and five negative effect estimates, which vary widely	⊕◯◯◯ VERY LOW	Limited importance
*(CB5) Decreased violence/discrimination at the household level*										
3	RCT‐1 QED‐2	Very serious^am^	Serious^an^	Not serious	Not serious	None	1875	SMD 0.16 SD higher (0.02 higher to 0.29 higher)	⊕◯◯◯ VERY LOW	Critical
*(CB6) Safer and more secure household, communities and areas/territories for women, girls, men and boys*										
1	RCT	Serious^ao^	Serious^b^	Not serious	Serious^ap^	None	283	SMD 0.19 SD higher (0.05 lower to 0.42 higher)	⊕◯◯◯ VERY LOW	Critical
*(CB8) Improved quality of relationships between women and their household and community members*										
4	RCT‐2 QED‐2	Serious^aq^	Very serious^ar^	Not serious	Serious^as^	None	5729	SMD 0.02 SD (0.13 lower to 0.17 higher)	⊕◯◯◯ VERY LOW	Limited importance
(CC1) Empowerment/Equality Index										
5	RCT‐2 QED‐3	Very serious^at^	Not serious	Not serious	Not serious	None	2481	SMD 0.09 SD higher (0.01 higher to 0.24 higher)	⊕⊕◯◯ LOW	Important, but not critical

Abbreviations: CI, confidence interval; GRADE, Grading of Recommendations, Assessment, Development and Evaluations; QED, quasi‐experimental design; RCT, randomised controlled trial; SHG, self‐help group; SMD, standardised mean difference; VSLA, Village Savings and Loan Association.

^a^ One study assessed as having a high risk of bias. Reporting bias is likely. The deviations from intended interventions and the outcome measurement bias are unclear.

^b^ All single studies downgraded once for inconsistency.

^c^ This analysis has been downgraded once due to some concerns related to risk of bias for both studies although there is no major concern.

^d^ Downgraded once: (1) variation of points estimates: no overlaps, (2) overlaps of CI: do not cross no effect line, (3) $I^{2}=95.46\%$, (4) test of stat: According to the *Q*‐test, there was a significant amount of heterogeneity in the true outcomes.

^e^ This analysis has been downgraded twice due to one study having a high risk of bias and all other studies with some concerns related to risk of bias.

^f^ Downgraded to reflect widely varying point estimates and CIs that do not overlap.

^g^ Downgraded as the deviations from intended interventions and the outcome measurement bias were unclear.

^h^ Downgraded once because of very wide confidence intervals.

^i^ Assessed as having some concerns related to risk of bias, due to all the criteria being unclear except outcome measurement bias.

^j^ Downgraded because of risk of reporting bias.

^k^ This analysis has been downgraded once due to some concerns related to risk of bias for both studies although there is no major concern.

^l^ Assessed as having a high risk of bias. Reporting bias is likely. The deviations from intended interventions and the outcome measurement bias are unclear.

^m^ Assessed as having a high risk of bias. Reporting bias is likely. The deviations from intended interventions and the outcome measurement bias are unclear.

^n^ Downgraded once because of very wide confidence intervals.

^o^ This analysis has been downgraded twice due to two studies having a high risk of bias and all other studies with some concerns related to risk of bias.

^p^ Downgraded once: (1) variation of points estimates: points estimates on both side of the threshold, (2) overlaps of CI: not systematic overlap of CI.

^q^ Downgraded once to reflect the effect estimates wide confidence interval.

^r^ Downgraded twice because all studies present risk of bias, and two‐thirds of the evidence comes from high risk of bias QEDs.

^s^ This analysis has been downgraded twice due to one study having a high risk of bias and all other studies with some concerns related to risk of bias and a mix of RCT and QED.

^t^ (1) Variation of points estimates: points estimates on the same side of the threshold. (2) Overlaps of CI: no systematic overlaps of CI. (3) $I^{2}=97.58\%$. (4) Test of stat: the true outcomes appear to be heterogeneous downgrade twice.

^u^ Downgraded twice because both studies are high risk of bias QEDs.

^v^ Assessed as having a high risk of bias. All the criteria are assessed as being likely biased, except the deviations from intended interventions being likely not biased and the outcome measurement bias being unclear.

^w^ Downgraded twice times: (1) the RCTs have some concerns related to risk of bias, (2) two studies have a high risk of bias, (3) removing either high risk study would have an impact on the meta‐analysis.

^x^ Downgrade twice: (1) variation of points estimates: points estimates on both side of the threshold, (2) overlaps of CI: no systematic overlaps of CI, (3) $I^{2}=53.69\%$, (4) test of stat: the true outcomes appear to be heterogeneous.

^y^ Downgraded once to reflect a very large *p*‐value and wide‐ranging CI of the meta‐analytic effect estimate.

^z^ Downgraded twice because all studies present risk of bias, and 2/4 of the evidence comes from high risk of bias QEDs.

^aa^ Downgraded twice because one study is very inconsistent with the others, including CI's that do not overlap and a point estimate that varies considerably from all others. When this study is removed, the meta‐analytic results change.

^ab^ Assessed as having a high risk of bias. Reporting bias is likely. The deviations from intended interventions and the outcome measurement bias are unclear.

^ac^ Downgraded due to risk of reporting bias.

^ad^ Downgraded to very serious because all of the evidence in this group comes from high risk of bias studies.

^ae^ Downgraded due to risk of reporting bias.

^af^ This group has been downgraded twice because both included studies present at least some concern, and one of two studies present high risk. In particular, there was uncertainty or high risk of bias for all studies with respect to deviation from intended intervention.

^ag^ Downgraded because the CI of the RE effect estimate are large, and cross over both sides of the threshold of interest.

^ah^ Downgraded twice because: (1) all three studies included are high risk of bias QEDs, and (2) removing any study would significantly impact the effect estimate and leave the group with one high risk of bias study.

^ai^ Downgraded once to reflect very different effect sizes and CIs that cross both sides of the threshold.

^aj^ Downgraded because the RE effect estimate cross the threshold of interest and has CI that are larger than the effect estimate itself.

^ak^ Downgraded as the deviations from intended interventions and the outcome measurement bias were unclear.

^al^ Downgraded twice due to widely varying point estimates far across both sides of the threshold of interest.

^am^ Downgraded twice because: (1) 79.91% of the weight of the meta‐analysis comes from high risk of bias QEDs, and (2) The only non‐high risk of bias study is unclear across several domain criteria like performance bias and outcome measurement bias.

^an^ Downgraded because there are point estimates on either side of the threshold and a very wide CI range across the studies.

^ao^ Downgraded as having some concerns related to risk of bias. The unit of analysis criteria has a likely bias and the deviations from intended interventions criteria is unclear.

^ap^ Downgraded due to very wide confidence intervals.

^aq^ 1/4 of the studies in this group was high risk of bias, and several others were unclear.

^ar^ This group includes studies that are both significantly positive and significantly negative, including studies whose CIs are entirely on one side of the other of the threshold of interest.

^as^ Downgraded because the RE model effect estimate has wide CIs that reach widely onto both side of the threshold.

^at^ Downgraded twice because 3/5 studies in this group comes from high risk of bias QEDs. There is concern or uncertainty with respect to selection bias, outcome measurement, performance bias (and other criteria) across all studies in the group.

#### 5.3.8 Technical and vocational education and training (TVET)

TVET are educational interventions primarily designed to lead participants to acquire practical skills, know‐how and understanding necessary for employment in a particular occupation, trade or group of occupations or trades (Pompa, 2014).

##### 5.3.8.1 How does TVET affect gender equality, women's empowerment and Peace outcomes?

**Figure 94:**
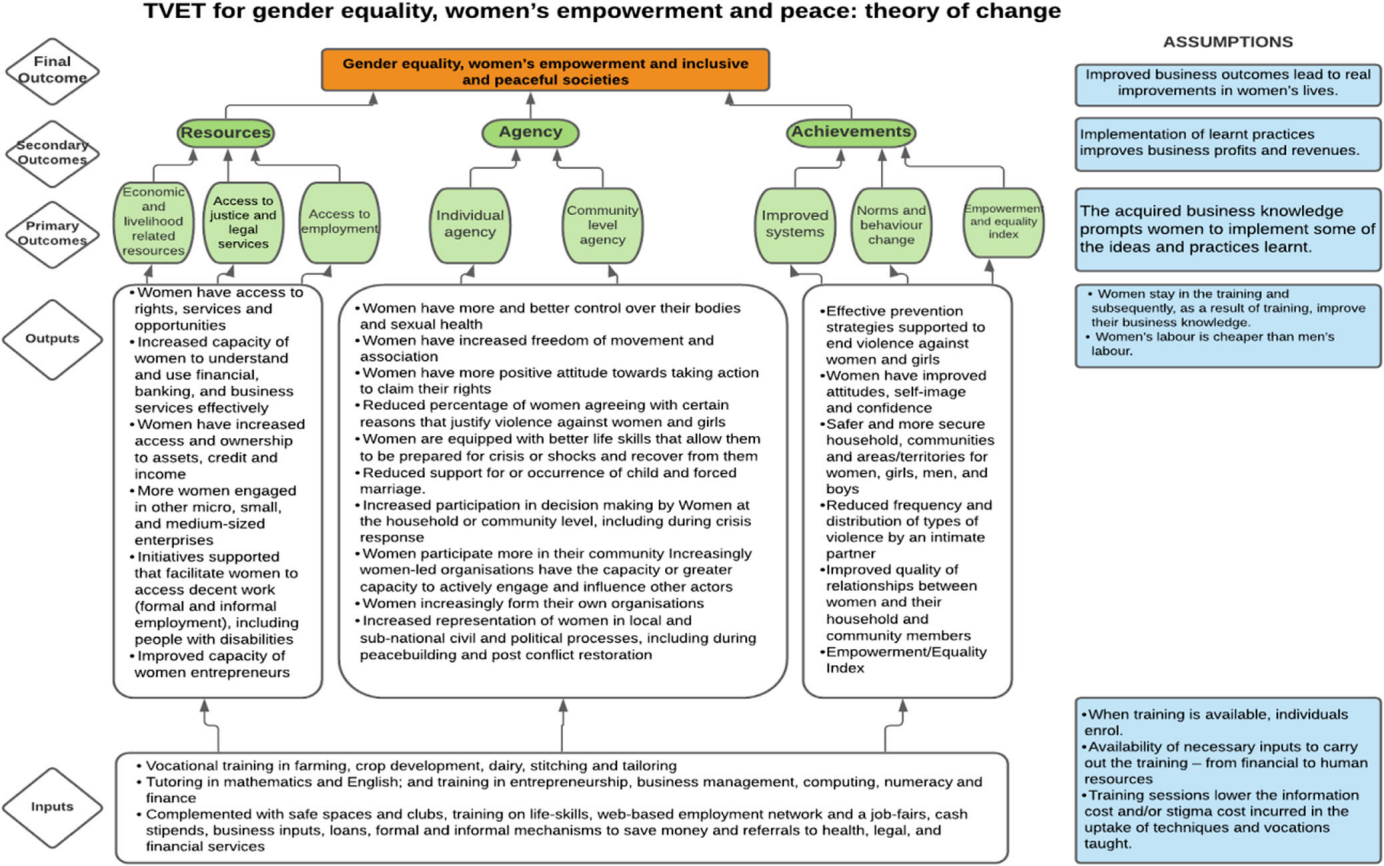
Technical and vocational education and training for gender equality, women's empowerment and peace: Theory of change

Figure 94 maps out the causal chain of how TVET may improve gender equality, women's empowerment and peace outcomes.

Vocational education and training in entrepreneurship, business management, computing, numeracy and finance, is expected to empower women through the development of skills that improve their access to financial services, employment, assets, and resources in general. These new skills help women participate more in decision making processes and in their communities with improved attitude, self‐image and confidence to take action and claim their rights. This is based on the assumption that, when available, individuals take up training, stay in it and, subsequently, improve their vocational or business knowledge so that the implementation of learnt practices can increase business profits and revenues.

##### 5.3.8.2 Description of included studies

We included 10 studies reported in 12 different papers that evaluated the effect of eight identified programmes. We included more than one paper that evaluated the same programme if the author reported different outcomes over several papers.

*Population*

The interventions evaluated by included studies targeted adolescent girls aged 12 years or older, young women, college graduates and women farmers.

The included studies looked at eight different programmes and trials in South Asia and Sub‐Saharan Africa and ten countries including: Afghanistan, Bangladesh, India, Kenya, Liberia, Nigeria, Pakistan, Sierra Leone, Uganda and Zambia.

*Intervention, inputs and activities*

The included studies evaluated a range of different TVET activities and inputs including the following:

- Vocational training plus resource provision (*n* = 3): Skill‐building in numeracy and chosen vocational skill (such as sewing) together with foundational training in modules that include: the value of women's work, financial literacy, basic business skills and inputs, ways to improve health and well‐being, women's rights and prevention of SGBV, strategies to make decisions and negotiate, civic action and advocacy, social networks and safety nets. Resources provision in the form of loans or cash stipend, mechanisms to save money, and referrals to health, legal and financial services.
- Tutoring and training (*n* = 7): Classroom‐based technical and life skills training, with a focus on skills with high market demand such as stitching and tailoring, or farmer skills training and crop development.
- Entrepreneurship skills‐building (*n* = 1): Training on gender and entrepreneurship, business woman and her environment, the business project, and people, organisation and management.
- Community groups training (*n* = 1): training on the dairy sector plus establishment of collection centres for collecting milk from small farm producers (Table 32).

**Table 32:** Activities and design features of included technical and vocational education and training studies

Study	Activity/input	Length of treatment	Intervention frequency
Carney and Carney (2018)	Farmer skills training and crop development	NA	Unclear
Gibbs et al. (2020)	Foundational training, skill‐building in numeracy and a chosen vocational skill, cash stipend, formal and informal mechanisms to save money, and referrals to health, legal and financial services, combined with safe spaces	Unclear	Weekly
Gibbs et al. (2018)	Foundational training, skill‐building in numeracy and a chosen vocational skill, cash stipend, formal and informal mechanisms to save money, and referrals to health, legal and financial services, combined with safe spaces	Unclear	Weekly
Mckenzie et al. (2019)	Training on gender and entrepreneurship, businesswoman and her environment, the business project, and people, organisation and management	30	Daily
Lombardini and Yoshikawa (2015)	Community groups who received training on the dairy sector combined with collection centres and an established enterprise with a formal agreement with a major company in the dairy sector for selling the milk from collected sectors	24	Unclear
Croke et al. (2017)	Classroom‐based training, web‐based employment network and a job‐fair	13	Weekly
Bandiera et al. (2019)	Peer education in life skills, safe spaces, vocational skills training, financial literacy, business inputs, loan	19	Daily
Adoho et al. (2014)	Classroom‐based technical and life skills training, with a focus on skills with high market demand, followed by placement and follow‐up support in their transition to self or wage employment	12	Unclear
Bandiera et al. (2018)	Safe spaces, life skills training, vocational skills training, recreational activities, financial literacy courses	22	Daily
Maitra and Mani (2017)	Stitching and tailoring vocational training	6	Daily
Bandiera et al. (2012)	Safe spaces, life skills training, vocational skills training, recreational activities, financial literacy courses	22	Daily
Amin et al. (2016)	Tutoring in mathematics and English (in‐school girls), and computing or financial training (out‐of‐school girls); life skills training on gender rights and negotiation, critical thinking, and decision‐making; and training in computers, entrepreneurship, mobile phone servicing, photography and basic first aid	18	Weekly

*Comparison*

All our included studies compared treated groups to comparison groups receiving no intervention. Three studies included multiple treatment arms.

*Outcomes*

The included studies reported on a number of relevant outcomes, including the following:

- Women have increased access and ownership to assets, credit and income (*n* = 10): Women are able to apply for, receive and manage assets/credit and income and have support to manage, claim and execute their assets without pressure or influence from external actors, including male family members, husbands and cultural leaders.
- Women can access decent work (formal and informal employment) (*n* = 6): Women are able to apply for, receive and work in jobs (and have support for the above), without discrimination by sex, gender or other identifying factors, including development of skills for improved access.
- Improved capacity of women entrepreneurs (*n* = 5): The capacity can both cover technical abilities and resources to achieve a goal. Through skills training, microgrants, and linkage of services, women entrepreneurs are able to pursue their businesses and other pursuits.
- Increased participation in decision making by women at the household or community level, including during crisis response (*n* = 4): Women take part in all or any step of the decision‐making process at the household community and district level, but also are able to meaningfully take part and have influence on the final decision, including in crisis response.
- Women have improved attitudes, self‐image and confidence (*n* = 6): Women feel more entitled to claim their rights and needs in community and change social norms and behaviours. They are aware of the importance of their status in society and are empowered to take this role and make use of the available resources to guarantee their rights.

The division of the immediate and secondary outcomes is reported in Table 38 (Table 33).

**Table 33:** Technical and vocational education and training summary of secondary and immediate outcomes

Secondary outcome category	Immediate outcome	Number of studies
Resources material, human and social resources which serve to enhance the ability to exercise choice	Access to justice and legal services	0
	Economic and livelihood related resources	10
	Access to employment	8
Agency ability to define one's goals and act upon them and operationalised decision‐making	Individual agency	9
	Community level agency	6
	Institutions supporting agency	0
Achievement ways of being and doing which can be realised by different individuals	Improved systems	3
	Norms and behaviour change	8
	Empowerment index	5

*Study design*

**Figure 95:**
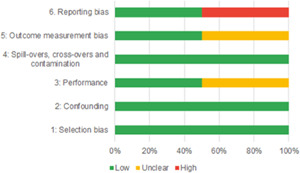
Technical and vocational education and training studies quasi‐experimental design risk of bias assessment

Two of the TVET studies used a QED (Carney & Carney, 2018; Lombardini & Yoshikawa, 2015). We assessed Carney and Carney (2018) as having a low risk of bias and Lombardini and Yoshikawa (2015) as having high risk of bias. As detailed in Figure 95, high risk of bias was identified on the reporting bias domain because the authors do not provide any robustness checks (Lombardini & Yoshikawa, 2015). The same study did not provide enough information to discard issues related to outcome measurement and performance bias. No limitations related to reporting bias, nor issues with confounding or selection bias, were detected.

**Figure 96:**
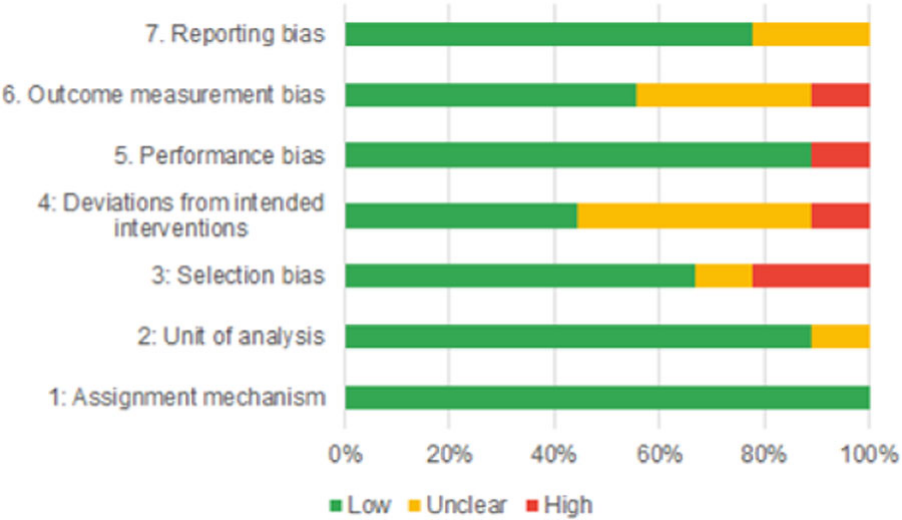
Technical and vocational education and training studies randomised controlled trial risk of bias assessment

Nine of the TVET papers used an experimental design (Figure 96). This included Adoho et al. (2014), Amin et al. (2016), Bandiera et al. (2018), Bandiera et al. (2012, 2019), Gibbs et al. (2018, 2020), Maitra and Mani (2017) and Mckenzie et al. (2019). Only one study was assessed as having a low risk of bias and four were assessed as having a high risk of bias, the rest (*n* = 4) were assed as having some concerns related to risk of bias. Some of the issues with RCTs evaluating TVET interventions were differences in attrition rates between treatment and control groups that could lead to selection bias (Amin et al., 2016; Gibbs et al., 2020); we also identified high risk of deviations from intended intervention in one study that reported noncompliance of the assignment to treatment (Bandiera et al., 2012), and a risk of performance bias in a study that reported a possible change in behaviour in response to the presence of the program (Adoho et al., 2014). The only category for which all studies were assessed as having low risk of bias was the assignment mechanism, while for unit of analysis and reporting bias, there was one and two studies that did not provide enough information to discard any issues.

*Qualitative studies, process evaluations and project documents*

We identified five additional documents related to three programmes covered by the TVET group of studies.

- GET Ahead for Women in Enterprise (Kenya): one qualitative study and one descriptive quantitative study
- Economic Empowerment of Adolescent Girls and Young Women (EPAG) (Liberia): one process evaluation and one descriptive quantitative study
- Empowerment and Livelihood for Adolescents (ELA) programme (Sierra Leone): one descriptive quantitative study

All but one (*n* = 4) were ranked as moderate quality, with the remaining study marked as low quality.

##### 5.3.8.3 Synthesis of findings

The following subsection presents the results of effectiveness of (intervention group) on Gender Equality, Women's Empowerment and Peace outcomes.

*Quantitative findings*

*Effect of TVET on women having increased capacity to understand and use financial, banking and business services*

Bandiera's et al. (2018), experimental study in Sierra Leone was the only study evaluating the impact of TVET on women having increased capacity to understand and use financial, banking and business services. Their report included one effect for four groups that fell into this outcome category (e.g., financial literacy for 12–17 year olds in high and low disruption villages and 18–25 year olds in high and low disruption villages). The effects ranged from small, negative point estimates (*g *=* *−0.05, [95% CI: −0.16 to 0.06]) to small, positive point estimates (*g *=* *0.10, [95% CI: −0.01 to 0.22]). The effects in the high disruption (Ebola) villages were larger than those in the low disruption villages, however none of the effect sizes were statistically significant at *p *<* *0.05. We assessed the study as having some risk of bias concerns.

*Effect of TVET on women having increased access to and ownership of assets, credit and income*

**Figure 97:**
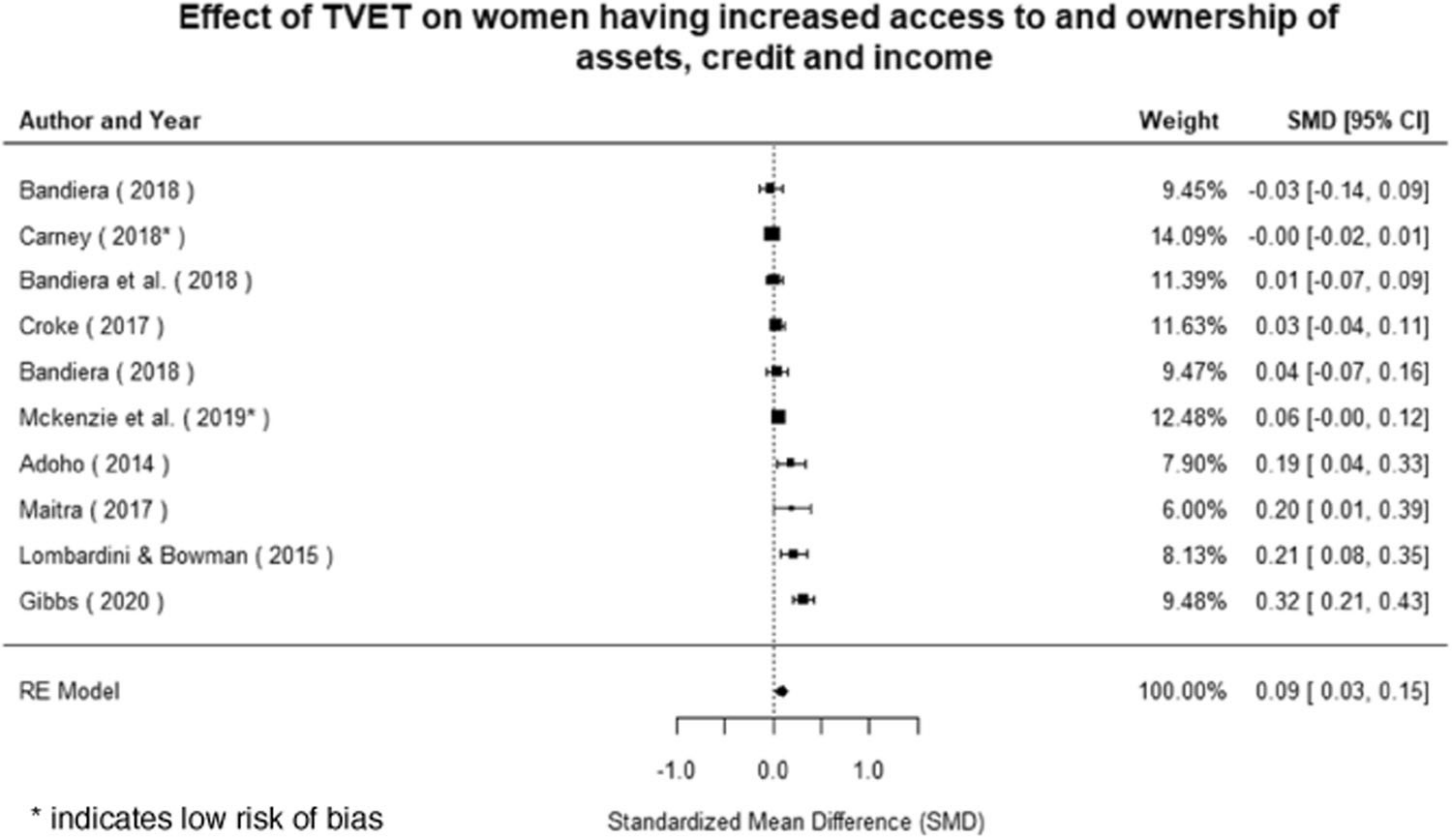
TVETAB2: Forest plot showing the observed outcomes and the estimate of the random‐effects model. CI, confidence interval; TVET, technical and vocational education and training

We included a total of k=10 studies in the analysis. We assessed two studies as low risk of bias, three as some concerns, and five as high risk of bias. The observed outcomes ranged from −0.03 to 0.32. The estimated average outcome based on the random‐effects model was $\hat{\mu }=0.09$ (95% CI: 0.03 to 0.15). Therefore, the average outcome differed significantly from zero (z=2.85, p<0.01). Two of the Bandiera et al. (2018), effects reflect separate effect for 12–17 year olds (*g *=* *−0.03) and 18–25 year olds (*g *=* *0.04) from Sierra Leone, while Bandiera et al. (2018) is the effect from Uganda. A forest plot showing the observed outcomes and the estimate based on the random‐effects model is shown in Figure 97 (TVETAB2).

According to the Q‐test, the true outcomes appear to be heterogeneous (Q(9)=52.67, p<0.01, ${\hat{\tau }}^{2}=0.01$, $I^{2}=82.91$%). An examination of the studentised residuals revealed that one study (Gibbs et al., 2020) had a value larger than ±2.84 and may be a potential outlier in the context of this model. According to the Cook's distances, one study (Gibbs et al., 2020) could be considered to be overly influential. Indeed, sensitivity analyses leaving each study out indicated that removing Gibbs et al. (2020) would reduce the overall average effect (μ=0.04 [95% CI: 0.004 to 0.08]), but the effect would still be positive and significant (*z *=* *2.15, *p *=* *0.03).

We were able to test several moderators in the context of this model. Exposure to intervention in months ($\hat{B}=0.001,p=0.76$ [95% CI: −0.005 to 0.01]) and evaluation period in months ($\hat{B}=0.002,p=0.64$ [95% CI: −0.01 to 0.01]) were also not significant. Effects from studies using experimental designs were similar to effects found in studies with QEDs ($\hat{B}=0.03,p=0.70$ [95% CI: −0.13 to 0.20]). Studies examining change from baseline had similar effects to those examining post‐intervention changes ($\hat{B}=-0.12,p=0.07$ [95% CI: −0.26 to 0.11]). Effects from models that adjusted for covariates did not differ from models with no covariate adjustments ($\hat{B}=-0.13,p=0.29$ [95% CI: −0.38 to 0.11]). Effects also did not differ by scores on the GII ($\hat{B}=-0.50,p=0.60$ [95% CI: −2.40 to 1.39]) or the FSI ($\hat{B}=0.002,p=0.50$ [95% CI: −0.004 to 0.01]). Finally, the qualitative evidence suggested that there might be a potential difference between programmes that targeted women only versus those that targeted both men and women. Thus, we tested this as another source of potential heterogeneity specifically for TVET programmes. We found that effects were not different between programmes exclusively targeting women versus mixed target programmes ($\hat{B}=0.02,p=0.79$ [95% CI: −0.16 to 0.21]).

The only significant moderator was the risk of bias assessment. Studies with a high risk of bias were significantly different than studies with some concerns or low risk of bias ($\hat{B}=0.13,p=0.03$ [95% CI: 0.01 to 0.26]), such that studies assessed as high risk of bias had larger effects by 0.13 standard deviation units.

**Figure 98:**
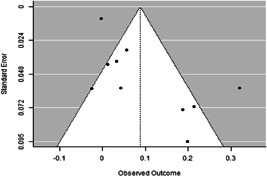
TVETAB2b: Funnel plot

A funnel plot of the estimates is shown in Figure 98. The regression test indicated funnel plot asymmetry (p=0.01) but not the rank correlation test (p=0.38).

*Effect of TVET on women being more engaged in micro, small and medium‐sized enterprises*

Mckenzie and colleague's (2019) experimental study in Kenya was the only study evaluating the impact of TVET on women being more engaged in micro, small and medium‐sized enterprises. Their report examined how many women were talking about their businesses, providing impact estimates at both the 12‐ and 36‐month follow‐up periods. The point estimate was small and positive for the 12‐month follow‐up (*g *=* *0.04, [95% CI: −0.05 to 0.13]) while there was a small and significant positive effect at the 36‐month follow‐up (*g *=* *0.10, [95% CI: 0.01 to 0.19]). We assessed the study as having a low risk of bias.

*Effect of TVET on women having access to decent work*

**Figure 99:**
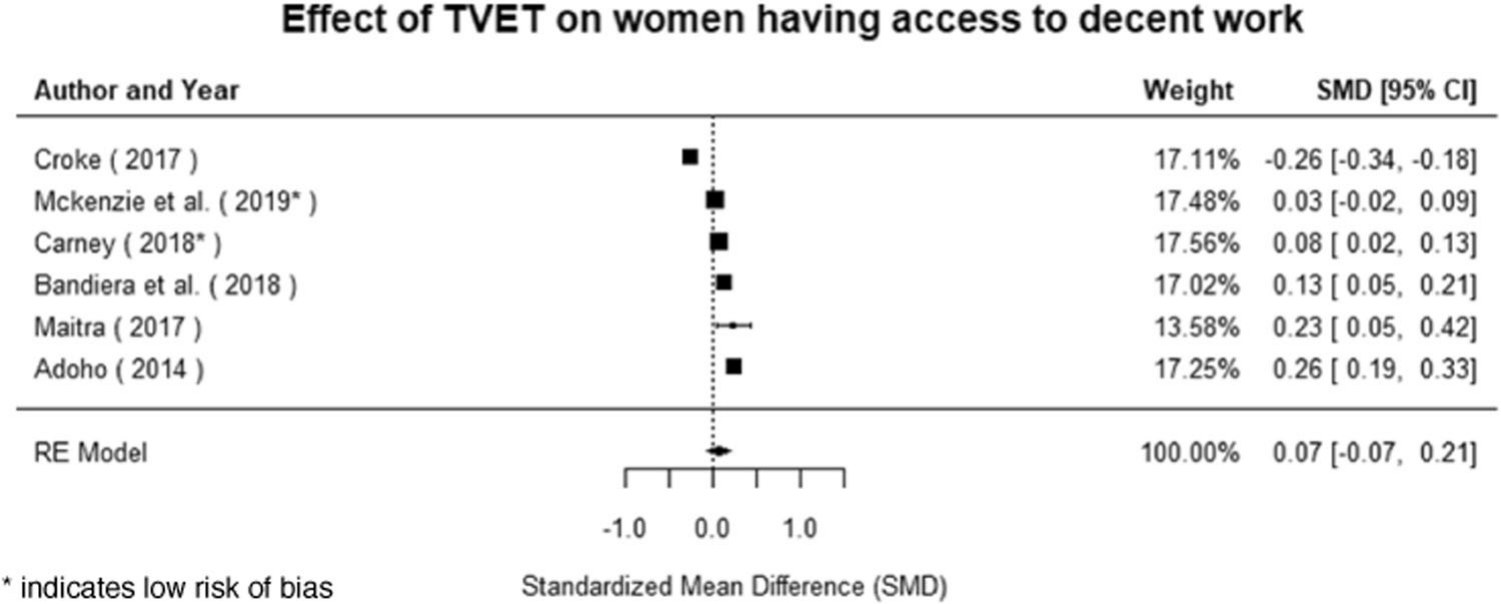
TVETAC2: Forest plot showing the observed outcomes and the estimate of the random‐effects model. CI, confidence interval; TVET, technical and vocational education and training

We included a total of k=6 studies in the analysis. We assessed two studies as low risk of bias, two as some concerns, and two as high risk of bias. The observed outcomes ranged from −0.26 to 0.26. The estimated average outcome based on the random‐effects model was $\hat{\mu }=0.07$ (95% CI: −0.07 to 0.21). Therefore, the average outcome did not differ significantly from zero (z=1.03, p=0.30). A forest plot showing the observed outcomes and the estimate based on the random‐effects model is shown in Figure 99 (TVETAC2).

According to the Q‐test, the true outcomes appear to be heterogeneous (Q(5)=106.30, p<0.01, ${\hat{\tau }}^{2}=0.03$, $I^{2}=95.30$%). An examination of the studentised residuals revealed that one study (Croke et al., 2017) had a value larger than ±2.64 and may be a potential outlier in the context of this model. According to the Cook's distances, none of the studies could be considered to be overly influential. Indeed, sensitivity analyses leaving each study out indicated that removing Croke et al. (2017) would increase the overall average effect ($\hat{\mu }=$0.14 [95% CI: 0.05 to 0.22]), and the resulting effect would be positive and significant (*z *=* *3.05, *p *=* *0.002).

We were able to test several moderators in the context of this model. Studies with a high risk of bias were not significantly different than studies with some concerns or low risk of bias ($\hat{B}=0.18,p=0.17$ [95% CI: −0.08 to 0.45]). Exposure to intervention in months ($\hat{B}=0.01,p=0.25$ [95% CI: −0.01 to 0.04]) and evaluation period in months ($\hat{B}=-0.01,p=0.07$ [95% CI: −0.02 to 0.001]). were also not significant. Studies using and experimental design had effects similar to those of studies using a QED ($\hat{B}=-0.14,p=0.38$ [95% CI: −0.18 to 0.46]). Effects also did not differ by scores on the fragile states index ($\hat{B}=-0.01,p=0.20$ [95% CI: −0.03 to 0.01]).

There were several moderators that could not be tested for this outcome group. First, only one study was looking at post‐intervention changes, while the rest were examining change from baseline, thus we could not test this as a moderator. We were also unable to the for differences based on the gender equality index score as there was not sufficient variation. Finally, while we wanted to test for a potential difference between programmes that targeted women only versus those that targeted both men and women, only one study in this outcome group used a mixed‐target approach, so we could not explore this as moderator.

*Effect of TVET on approved capacity of women entrepreneurs*

**Figure 100:**
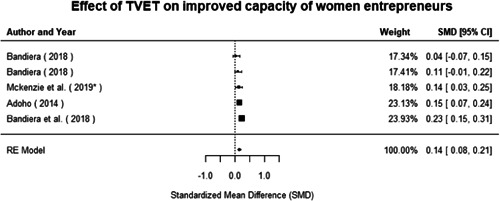
TVETAC3: Forest plot showing the observed outcomes and the estimate of the random‐effects model. CI, confidence interval; TVET, technical and vocational education and training

We included a total of k=5 studies in the analysis. We assessed one of the studies as low risk of bias, two as some concerns, and three as high risk of bias. The observed outcomes ranged from 0.04 to 0.23. The estimated average outcome based on the random‐effects model was $\hat{\mu }=0.14$ (95% CI: 0.08 to 0.21). Therefore, the average outcome differed significantly from zero (z=4.35, p<0.01). Two of the Bandiera et al. (2018), effects reflect separate effect for 12–17 year olds (*g *=* *0.04) and 18–25 year olds (*g *=* *0.11) from Sierra Leone, while Bandiera et al. (2018) is the effect from Uganda. A forest plot showing the observed outcomes and the estimate based on the random‐effects model is shown in Figure 100 (TVETAC3).

The Q‐test for heterogeneity was not significant, but some heterogeneity may still be present in the true outcomes (Q(4)=8.46, p=0.08, ${\hat{\tau }}^{2}=0.00$, $I^{2}=52.74$%). An examination of the studentised residuals revealed that none of the studies had a value larger than ±2.58 and hence there was no indication of outliers in the context of this model. According to the Cook's distances, none of the studies could be considered to be overly influential.

We were able to test several moderators in the context of this model. Studies with a high risk of bias were significantly different than studies with some concerns or low risk of bias ($\hat{B}=0.10,p=0.04$ [95% CI: 0.003 to 0.20]) such that studies with a high risk of bias had larger effects by 0.10 standard deviation units. Exposure to intervention in months ($\hat{B}=0.0000,p=0.99$[95% CI: −0.01 to 0.01])and evaluation period in months ($\hat{B}=-0.0001,p=0.99$ [95% CI: −0.02 to 0.01]) were also not significant. Finally, effects did not differ by scores on the GII ($\hat{B}=-0.89,=0.31$ [95% CI: −2.61 to 0.82]) or the FSI ($\hat{B}=0.01,p=0.23$ [95% CI: −0.01 to 0.03]).

All but one study was an experimental design, and all examined change from baseline. In addition, all studies only targeted women and all models adjusted for covariates. Thus, none of these could be tested as moderators.

*Effect of TVET on women having increased success in the workplace*

Adoho et al.'s (2014) quasi‐experimental study was the only study evaluating the impact of TVET on women having increased success in the workplace. Their report examined women's satisfaction with their job, finding moderate positive and significant impact their programme (*g *=* *0.23, 95% CI [0.16, 0.31]). We assessed the study as having a high risk of bias.

*Effect of TVET on women having more control over their bodies and sexual health*

**Figure 101:**
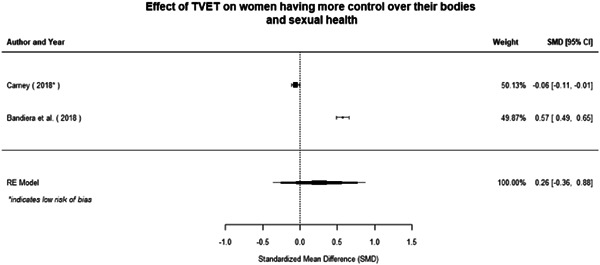
TVETBA2: Forest plot showing the observed outcomes and the estimate of the random‐effects model. CI, confidence interval; TVET, technical and vocational education and training

Only two studies reported disaggregated data for women having more control over their bodies and sexual health, thus we included k=2 studies in the analysis. We assessed one of the studies as low risk of bias and the other as high risk of bias. The observed outcomes ranged from −0.06 to 0.57. The estimated average outcome based on the random‐effects model was $\hat{\mu }=0.26$ (95% CI: −0.36 to 0.88). Therefore, the average outcome did not differ significantly from zero (z=0.81, p=0.42). A forest plot showing the observed outcomes and the estimate based on the random‐effects model is shown in Figure 101 (TVETBA2).

The Q‐test for heterogeneity was significant, thus the true outcomes appear to be heterogeneous (Q(4)=171.96, p<0.001, ${\hat{\tau }}^{2}=0.20$, $I^{2}=99.42$%). With only two studies, moderator analyses are not appropriate and tests of publication bias are not valid.

*Effect of TVET on women having increased freedom of movement and association*

**Figure 102:**
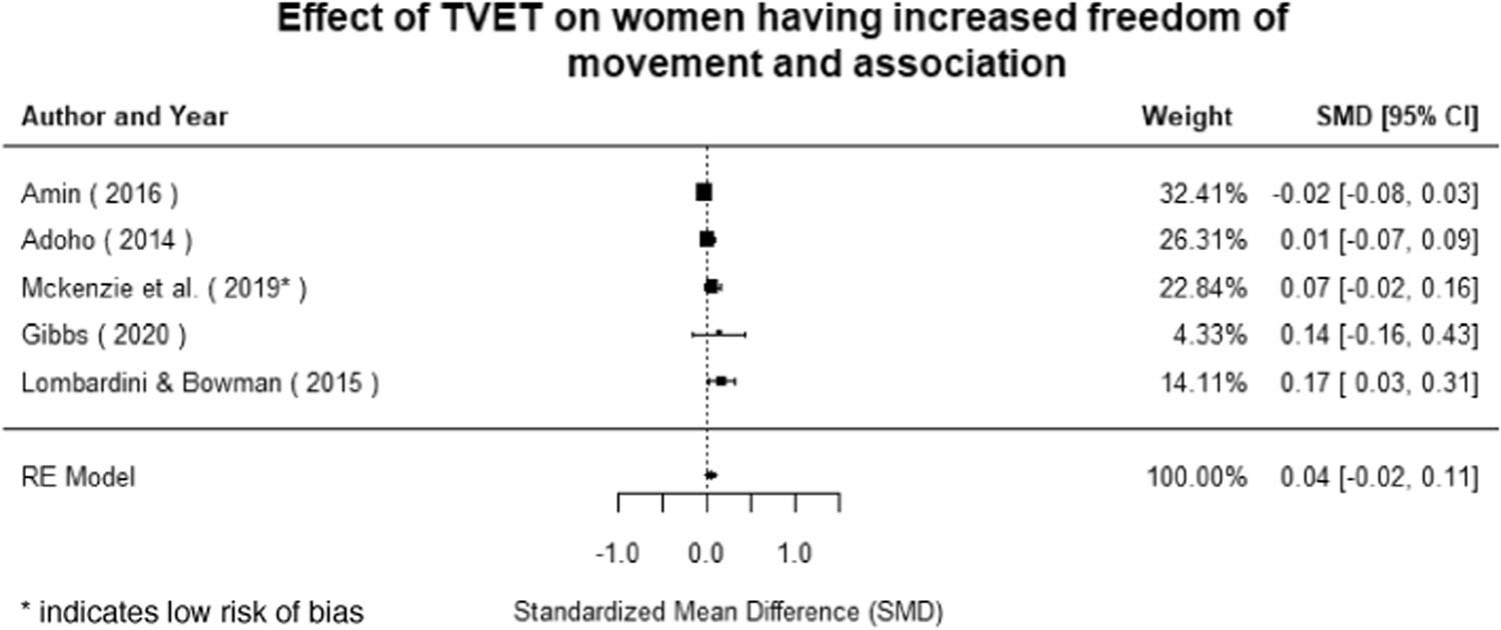
TVETBA3: Forest plot showing the observed outcomes and the estimate of the random‐effects model. CI, confidence interval; TVET, technical and vocational education and training

We included a total of k=5 studies in the analysis. We assessed one of the studies as low risk of bias, four as some concerns, and none as high risk of bias. The observed outcomes ranged from −0.02 to 0.17. The estimated average outcome based on the random‐effects model was $\hat{\mu }=0.04$ (95% CI: −0.02 to 0.11). Therefore, the average outcome did not differ significantly from zero (z=1.24, p=0.21). A forest plot showing the observed outcomes and the estimate based on the random‐effects model is shown in Figure 102 (TVETBA3).

The Q‐test for heterogeneity was not significant, but some heterogeneity may still be present in the true outcomes (Q(4)=8.43, p=0.08, ${\hat{\tau }}^{2}=0.00$, $I^{2}=52.57$%). An examination of the studentised residuals revealed that none of the studies had a value larger than ±2.58 and hence there was no indication of outliers in the context of this model. According to the Cook's distances, none of the studies could be considered to be overly influential.

We were able to test several moderators in the context of this model. Studies with a high risk of bias were not significantly different than studies with some concerns or low risk of bias ($\hat{B}=0.18,p=0.17$ [95% CI: −0.08 to 0.45]). Exposure to intervention in months ($\hat{B}=0.003,p=0.29$ [95% CI: −0.002 to 0.01]) and evaluation period in months ($\hat{B}=0.004,p=0.60$ [95% CI: −0.01 to 0.02]) were also not significant. Studies using an experimental design had effects similar to those of studies using a QED ($\hat{B}=0.04,p=0.58$ [95% CI: −0.11 to 0.20]). Effects also did not differ by scores on the GII ($\hat{B}=-0.40,p=0.74$ [95% CI: −2.73 to 1.93]), but they did differ by their scores on the FSI ($\hat{B}=0.01,p=0.01$ [95% CI: 0.003 to 0.02]) such that each 1 point increase on the fragile states index (indicating an increase in fragility) increased the effect by 0.01 standard deviation units. In other words, the more fragile states saw a larger benefit than the less fragile states.

Only one study was examining post‐intervention changes, while the rest were exploring change from baseline. There was also only one study targeting both men and women, while the remainder targeted women only. Finally, one study was not adjusted for covariates while all other studies were. These moderators could not be tested.

*Effect of TVET on women's attitude towards taking action to claim their rights*

Gibbs et al.'s (2020) experimental study in Afghanistan was the only study evaluating the impact of TVET on women having more positive attitude towards taking action to claim their rights. There was a very small, but not statistically significant, point estimate (*g *=* *0.03, [95% CI: −0.08 to 0.14]), and we assessed the study as having high risk of bias.

*Effect of TVET on reduced percentage of women agreeing with certain reasons that justify violence against women*

**Figure 103:**
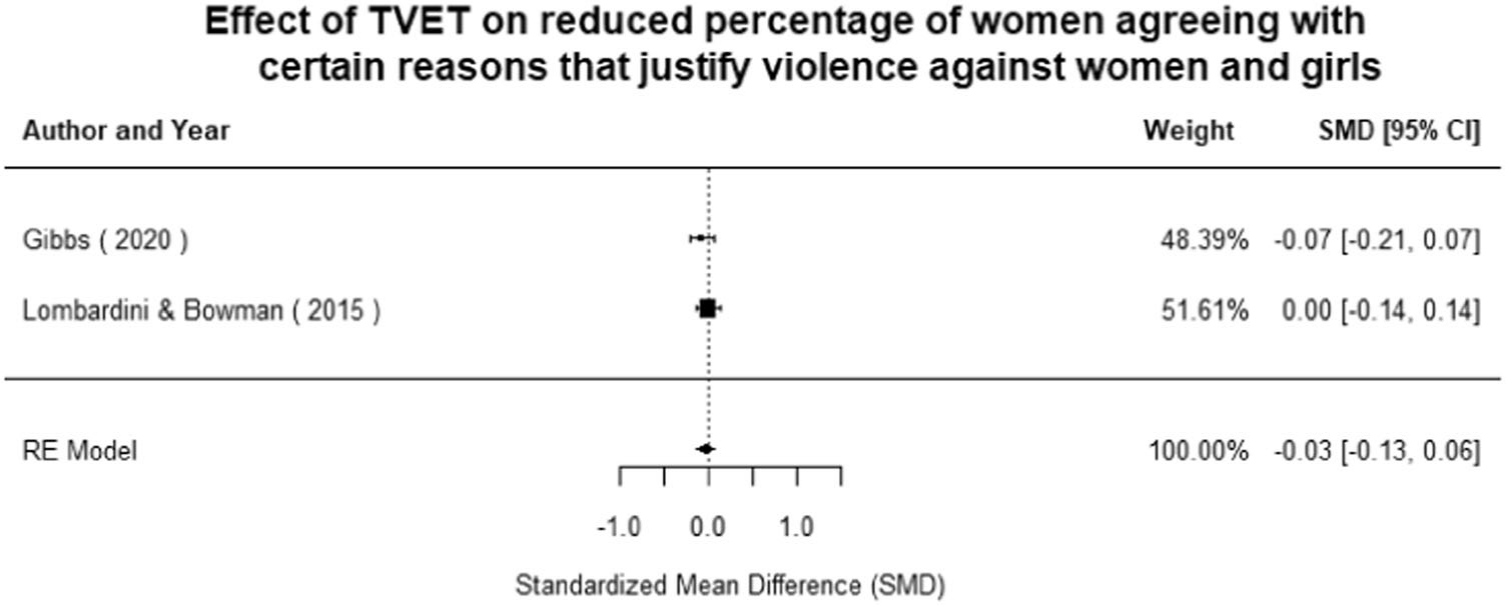
TVETBA6: Forest plot showing the observed outcomes and the estimate of the random‐effects model. CI, confidence interval; TVET, technical and vocational education and training

Only two studies reported the impact of TVET on a reduced percentage of women agreeing with certain reasons that justify violence against women, thus we included *k *=* *2 studies in the analysis. We assessed both of the studies as high risk of bias. The estimated average outcome based on the random‐effects model was $\hat{\mu }=-0.3$ (95% CI: −0.13to0.06). This positive effect did not differ significantly from zero (z=−0.69, p=0.49). A forest plot showing the observed outcomes and the estimate based on the random‐effects model is shown in Figure 103 (TVETBA6). Given the small number of studies, as well as the fact that both studies were assessed as high risk of bias, this result should be interpreted with caution. According to the Q‐test, there was no significant amount of heterogeneity in the true outcomes (Q(1)=0.51, p=0.49, ${\hat{\tau }}^{2}=0.00$, $I^{2}=0.00$%). With only two studies, moderator analyses were not possible, and tests of publication bias were not valid.

*Effect of TVET on women being equipped with better life skills*

**Figure 104:**
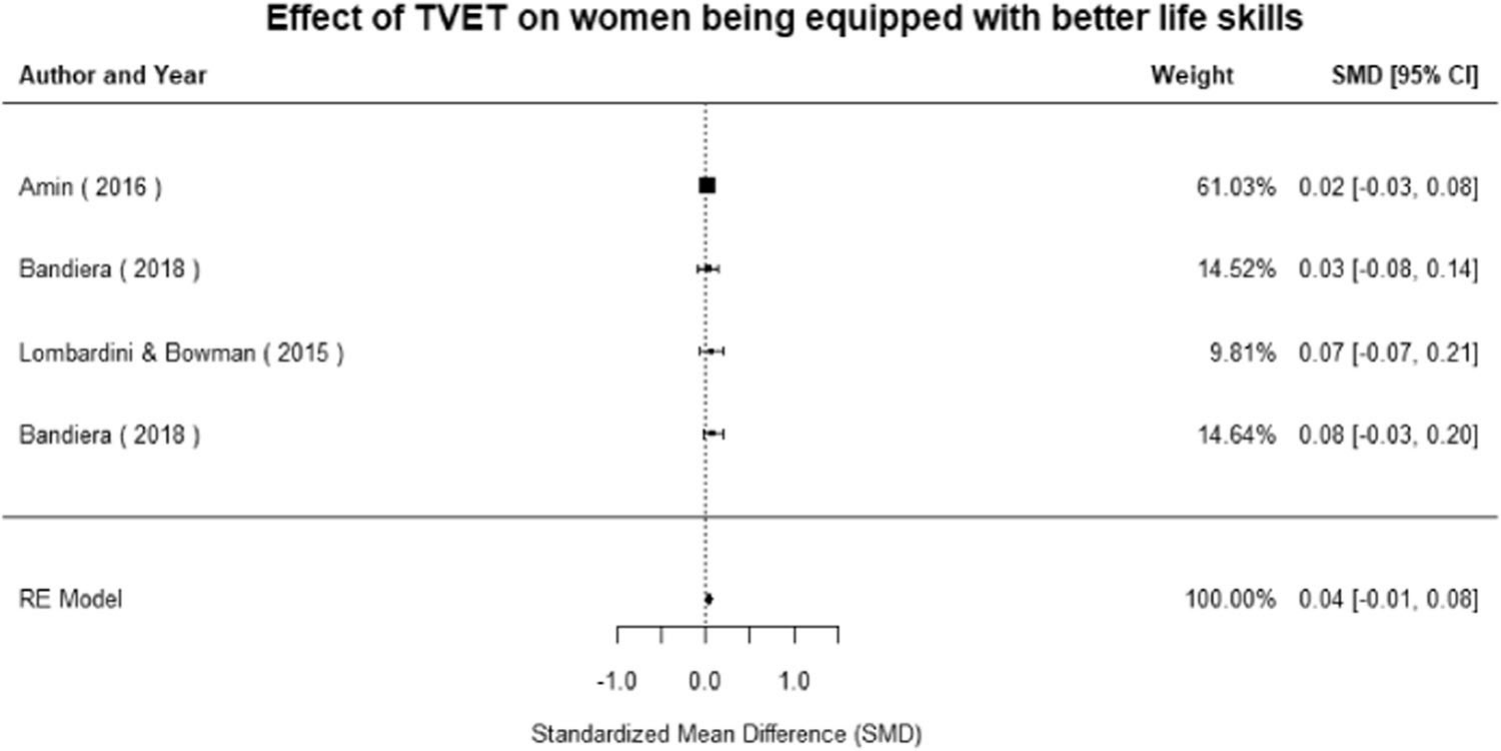
TVETBA7: Forest plot showing the observed outcomes and the estimate of the random‐effects model. CI, confidence interval; TVET, technical and vocational education and training

We included a total of *k *=* *4 studies in the analysis. We assessed none of the studies as low risk of bias, two as some concerns, and two as high risk of bias. The observed outcomes ranged from 0.02 to 0.08. The estimated average outcome based on the random‐effects model was $\hat{\mu }\;=\;$0.04 (95% CI: −0.01 to 0.08). Therefore, the average outcome did not differ significantly from zero (*z *=* *1.70, *p *=* *0.09). A forest plot showing the observed outcomes and the estimate based on the random‐effects model is shown in Figure 104 (TVETBA7).

According to the *Q*‐test, there was no significant amount of heterogeneity in the true outcomes Q(3)= 1.11, p= 0.77, ${\hat{\tau }}^{2}=$ 0.00, $I^{2}$ *=*0.00%). An examination of the studentised residuals revealed that none of the studies had a value larger than ±2.50 and hence there was no indication of outliers in the context of this model. According to the Cook's distances, none of the studies could be considered to be overly influential. With no heterogeneity, moderator analyses were not appropriate.

*Effect of TVET on reduced support for or instance of child and forced marriage*

**Figure 105:**
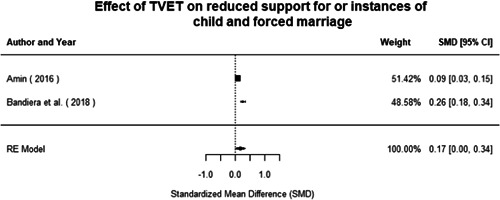
TVETBA8: Forest plot showing the observed outcomes and the estimate of the random‐effects model. CI, confidence interval; TVET, technical and vocational education and training

Only two studies reported the impact of TVET on a reduced support for or instance of child and forced marriage, thus we included *k *=* *2 studies in the analysis. We assessed one of the studies as having some concerns of risk of bias and the other as high risk of bias. The estimated average outcome based on the random‐effects model was $\hat{\mu }=0.17$ (95% CI: 0.004to0.34). This positive effect differed significantly from zero (z=2.01, p=0.04). A forest plot showing the observed outcomes and the estimate based on the random‐effects model is shown in Figure 105 (TVETBA8). Given the small number of studies, as well as the one study was assessed as high risk of bias, and the other was assessed as some concerns, this result should be interpreted with caution. According to the Q‐test, there was significant heterogeneity in the true outcomes (Q(1)=11.65, p<0.001, ${\hat{\tau }}^{2}=0.01$, $I^{2}=91.41$%). With only two studies, moderator analyses were not possible, and tests of publication bias are not valid.

*Effect of TVET on increased participation in decision making by women at the household and community level*

**Figure 106:**
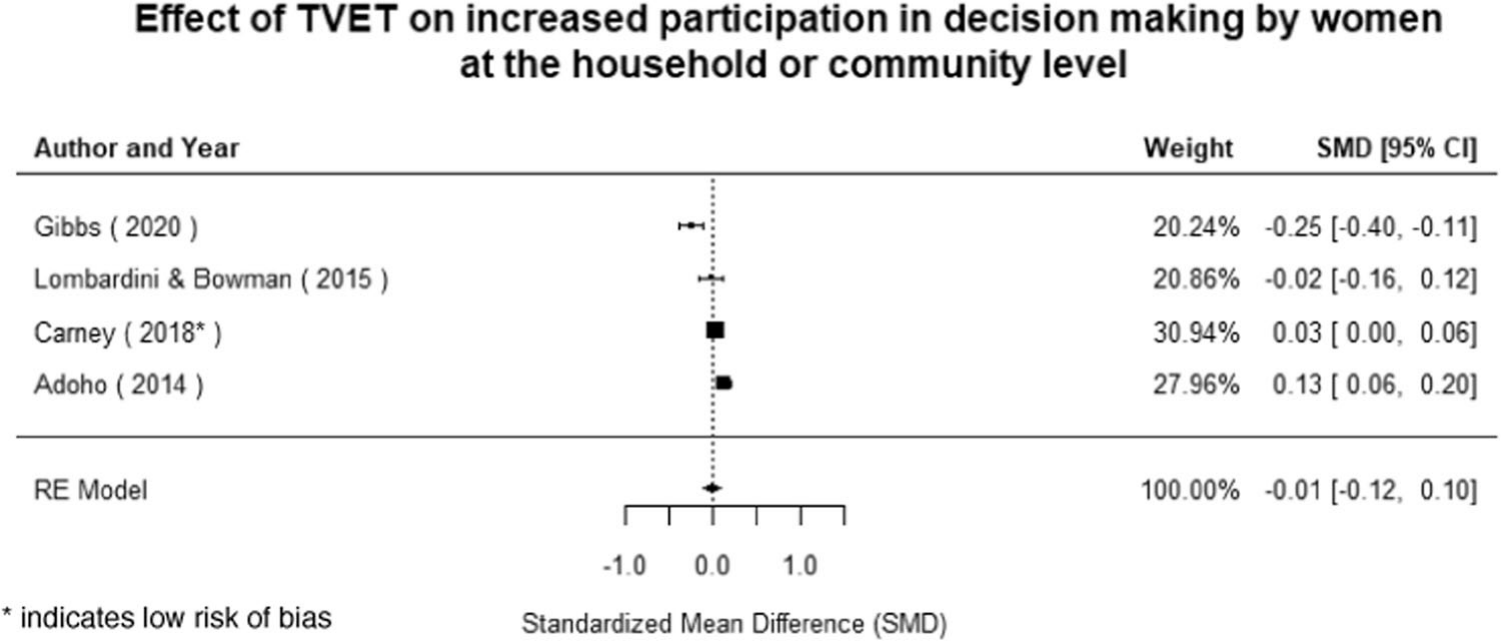
TVETBB1: Forest plot showing the observed outcomes and the estimate of the random‐effects model. CI, confidence interval; TVET, technical and vocational education and training

We included a total of k=4 studies in the analysis. We assessed one of the studies as low risk of bias, none as some concerns, and three as high risk of bias. The observed outcomes ranged from −0.25 to 0.13. The estimated average outcome based on the random‐effects model was $\hat{\mu }=-0.01$ (95% CI: −0.12 to 0.10). Therefore, the average outcome did not differ significantly from zero (z=−0.21, p=0.84). A forest plot showing the observed outcomes and the estimate based on the random‐effects model is shown in Figure 106 (TVETBB1).

According to the Q‐test, the true outcomes appear to be heterogeneous (Q(3)=22.60, p<0.01, ${\hat{\tau }}^{2}=0.01$, $I^{2}=86.72$%). An examination of the studentised residuals revealed that one study (Gibbs et al., 2020) had a value larger than ±2.50 and may be a potential outlier in the context of this model. According to the Cook's distances, none of the studies could be considered to be overly influential.

Exposure to intervention in months ($\hat{B}=0.001,p=0.81$ [95% CI: −0.01 to 0.01]) was not a significant moderator, but evaluation period in months was significant ($\hat{B}=-0.03,p=0.02$ [95% CI: −0.05 to 0.01]) such that each additional month between the end of the evaluation and the data collection, the size of the effect decreased by 0.03 standard deviation units.

Several moderators could not be tested, including whether the model was adjusted for covariates, whether the programmed targeted women or a mix of women and men, whether the study was examining post‐intervention changes or changes from baseline, GII score, FSI score and study design. We also could not examine risk of bias because three of the four studies were assessed as having a high risk of bias.

*Effect of TVET on women participating more in their communities*

Lombardini and Bowman (2015) quasi‐experimental study in Pakistan was the only study evaluating the impact of TVET on women participating more in their communities. There was a significant large positive effect (*g *=* *1.11, [95% CI: 0.97 to 1.25]), but we assessed the study as having high risk of bias.

*Effect of TVET on women forming their own organisations*

Mckenzie and colleagues' (2019) experimental study in Kenya was the only study evaluating the impact of TVET on women forming their own organisations, reporting on women's associations at the 12‐ and 36‐month follow‐up. While there was a very small positive but nonsignificant estimate at the 12‐month follow‐up (*g *=* *0.01, [95% CI: −0.08 to 0.10]), and there was a moderate positive and significant effect noted at the 36‐month follow‐up (*g *=* *0.15, [95% CI: 0.06 to 0.24]). We assessed the study as having high risk of bias.

*Effect of TVET on increased representation of women in local and subnational civil and political processes*

**Figure 107:**
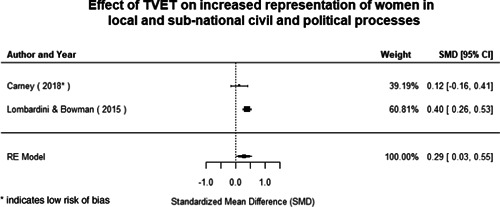
TVETCA2. Effect of technical and vocational education and training (TVET) on women having improved attitudes, self‐image and confidence. CI, confidence interval

Only two studies reported the impact of TVET on increased representation of women in local and subnational civil and political processes, thus we included *k *=* *2 studies in the analysis. We assessed one of the studies as low risk of bias and the other as high risk of bias. The estimated average outcome based on the random‐effects model was $\hat{\mu }=0.249$ (95% CI: 0.03to0.56). This positive effect did not differ significantly from zero (z=2.16, p=0.03). A forest plot showing the observed outcomes and the estimate based on the random‐effects model is shown in Figure 107 (TVETCA2). Given the small number of studies, this result should be interpreted with caution. According to the Q‐test, there was no significant amount of heterogeneity in the true outcomes (Q(1)=2.85, p=0.09, ${\hat{\tau }}^{2}=0.02$, $I^{2}=64.93$%). With only two studies, moderator analyses were not possible and tests of publication bias are not valid.

*Effect of TVET on women having improved attitudes, self‐image and confidence*

**Figure 108:**
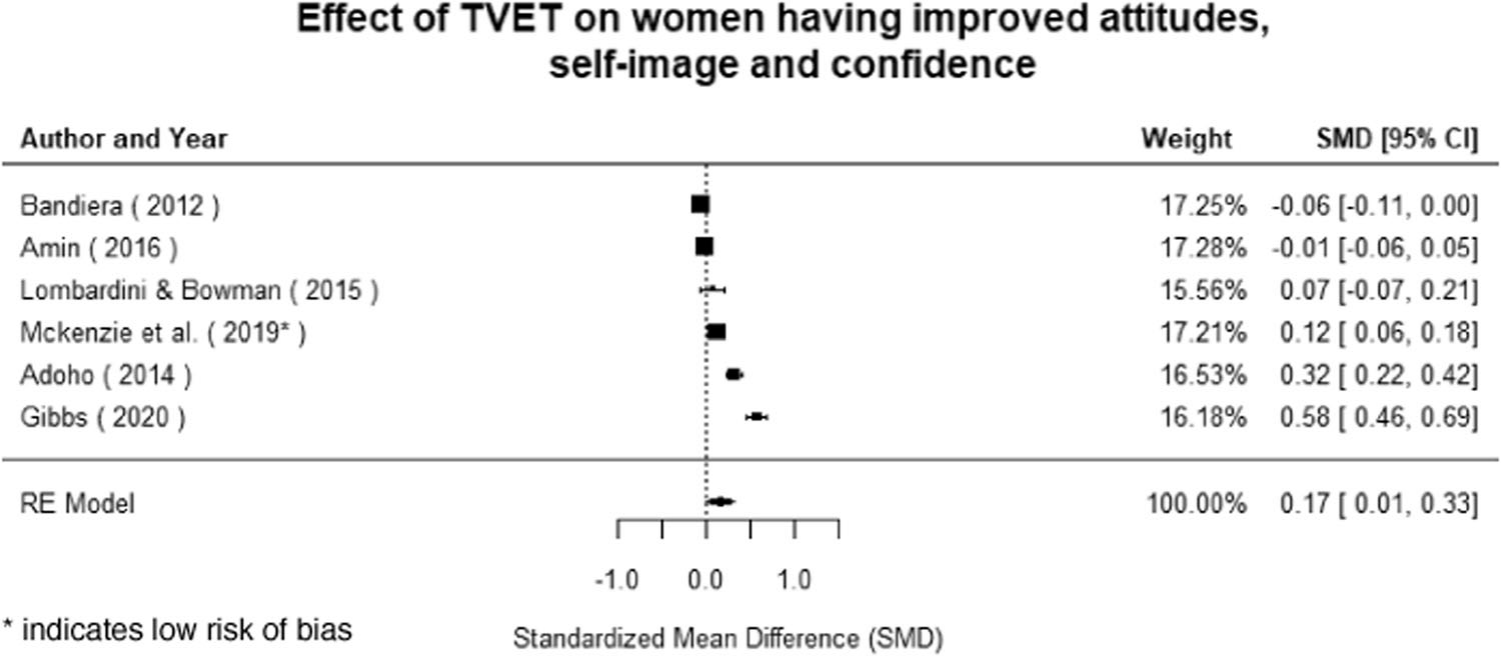
TVETCB3: Forest plot showing the observed outcomes and the estimate of the random‐effects model. CI, confidence interval; TVET, technical and vocational education and training

We included a total of k=6 studies in the analysis. We assessed one of the studies as low risk of bias, one as some concerns, and four as high risk of bias. The observed outcomes ranged from −0.06 to 0.58. The estimated average outcome based on the random‐effects model was $\hat{\mu }=0.17$ (95% CI: 0.01 to 0.33). Therefore, the average outcome differed significantly from zero (z=2.07, p=0.04). A forest plot showing the observed outcomes and the estimate based on the random‐effects model is shown in Figure 108 (TVETCB3).

According to the Q‐test, the true outcomes appear to be heterogeneous (Q(5)=129.22, p<0.01, ${\hat{\tau }}^{2}=0.04$, $I^{2}=96.13$%). An examination of the studentised residuals revealed that one study (Gibbs et al., 2020) had a value larger than ±2.64 and may be a potential outlier in the context of this model. According to the Cook's distances, none of the studies could be considered to be overly influential.

We examined several moderators in the context of this model. Effects from high risk of bias studies were not different than effects from low or some concerns related to risk of bias ($\hat{B}=0.17,p=0.43$ [95% CI: −0.25 to 0.54]). Exposure to intervention in months ($\hat{B}=-0.01,p=0.40$ [95% CI: −0.02 to 0.01]) was not a significant moderator, nor was evaluation period in months ($\hat{B}=0.02,p=0.20$ [95% CI: −0.01 to 0.06]). Effects from experimental studies were not different than effects from quasi‐experimental studies ($\hat{B}=0.04,p=0.85$ [95% CI: −0.40 to 0.49]). Effects also did not differ by scores on the gender equality index ($\hat{B}=2.8,p=0.08$ [95% CI: −0.29 to 0.5.9]), or the fragile states index ($\hat{B}=-0.01,p=0.70$ [95% CI: −0.04 to 0.03]).

Several moderators could not be tested, including whether the model was adjusted for covariates, whether the programmed targeted women or a mix of women and men and whether the study was examining post‐intervention changes or changes from baseline.

*Effect of TVET on decreased violence and discrimination at the household level*

Lombardini and Bowman (2015) quasi‐experimental study in Pakistan was the only study evaluating the impact of TVET on decreased violence and discrimination at the household level. There was a small positive point estimate (*g *=* *0.09, [95% CI: −0.05 to 0.22]) that was nonsignificant, and we assessed the study as having high risk of bias.

*Effect of TVET on safer and more secure households, communities and territories for women and girls*

Amin et al.'s (2016) experimental study in Bangladesh was the only study evaluating the impact of TVET on safer and more secure households, communities and territories for women and girls. They included nine effects, three for teach treatment arm, that looked at harassment at home, outside the home, or in school/class. The point estimates ranged from very small, negative and nonsignificant (*g *=* *−0.002, [95% CI: −0.06, 0.05]) to small, positive and significant effects (*g *=* *0.11, [95% CI: 0.06 to 0.17]). We assessed the study as having some concerns related to risk of bias.

*Effect of TVET on reduced frequency of intimate partner violence*

**Figure 109:**
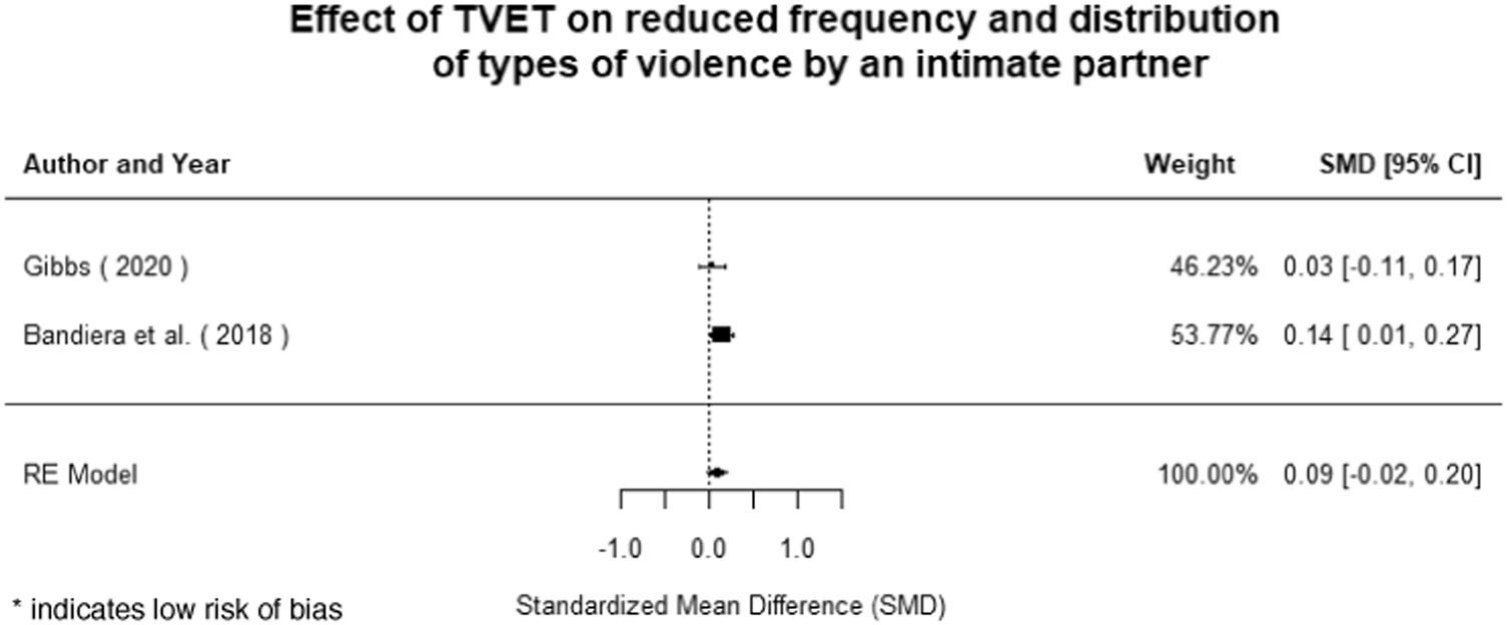
TVETCB7: Forest plot showing the observed outcomes and the estimate of the random‐effects model. CI, confidence interval; TVET, technical and vocational education and training

Only two studies reported the impact of TVET on the reduced frequency of intimate partner violence, thus we included *k *=* *2 studies in the analysis. We assessed both of the studies as high risk of bias. The estimated average outcome based on the random‐effects model was $\hat{\mu }=0.09$ (95% CI: −0.02to0.20). This positive effect did not differ significantly from zero (z=1.62, p=0.10). A forest plot showing the observed outcomes and the estimate based on the random‐effects model is shown in Figure 109 (TVETCB7). Given the small number of studies, this result should be interpreted with caution. According to the Q‐test, there was no significant amount of heterogeneity in the true outcomes (Q(1)=1.32, p=0.25, ${\hat{\tau }}^{2}=0.002$, $I^{2}=24.26$%). With only two studies, moderator analyses were not possible and tests of publication bias are not valid.

*Effect of TVET on improved quality of relationships between women and their household and community members*

**Figure 110:**
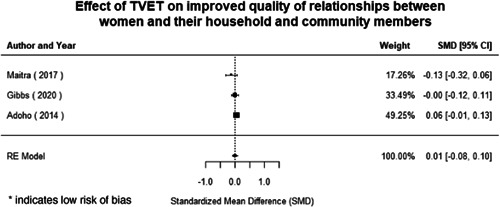
TVETCB8: Forest plot showing the observed outcomes and the estimate of the random‐effects model. CI, confidence interval; TVET, technical and vocational education and training

We included a total of k=3 studies in the analysis. We assessed none of the studies as low risk of bias, one as some concerns, and two as high risk of bias. The observed outcomes ranged from −0.13 to 0.06. The estimated average outcome based on the random‐effects model was $\hat{\mu }=0.01$ (95% CI: −0.08 to 0.10). Therefore, the average outcome did not differ significantly from zero (z=0.14, p=0.89). A forest plot showing the observed outcomes and the estimate based on the random‐effects model is shown in Figure 110 (TVETCB8).

According to the Q‐test, there was no significant amount of heterogeneity in the true outcomes (Q(2)=3.68, p=0.16, ${\hat{\tau }}^{2}=0.00$, $I^{2}=45.70$%). An examination of the studentised residuals revealed that none of the studies had a value larger than ±2.39 and hence there was no indication of outliers in the context of this model. According to the Cook's distances, none of the studies could be considered to be overly influential. With only three studies, moderator analyses were not appropriate.

*Effect of TVET on women's empowerment index*

**Figure 111:**
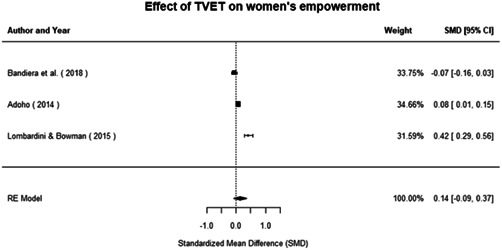
TVETCC1: Forest plot showing the observed outcomes and the estimate of the random‐effects model. CI, confidence interval; TVET, technical and vocational education and training

We included a total of k=3 studies in the analysis. We assessed all of the studies as high risk of bias. The observed outcomes ranged from −0.07 to 0.42. The estimated average outcome based on the random‐effects model was $\hat{\mu }=0.14$ (95% CI: −0.09 to 0.37). Therefore, the average outcome did not differ significantly from zero (z=1.19, p=0.24). A forest plot showing the observed outcomes and the estimate based on the random‐effects model is shown in Figure 111 (TVETCC1).

According to the Q‐test, the true outcomes appear to be heterogeneous (Q(2)=33.11, p<0.01, ${\hat{\tau }}^{2}=0.04$, $I^{2}=93.96$%). An examination of the studentised residuals revealed that one study (Lombardini & Bowman, 2015) had a value larger than ±2.50 and may be a potential outlier in the context of this model. Indeed sensitivity analyses leaving each study out indicated that removing Lombardini and Bowman (2015) would reduce the overall average effect ($\hat{\mu }\;=\;$0.01 [95% CI: −0.13 to 0.15]), but the effect would still be positive and nonsignificant (*z *=* *0.11, *p *=* *.91). According to the Cook's distances, none of the studies could be considered to be overly influential. With only three studies, moderator analyses were not appropriate.

*Qualitative findings*

We conducted a thematic synthesis on the five linked qualitative studies to the included TVET interventions. As indicated above, this thematic synthesis aims to identify themes related to the interplay of intervention design, intervention implementation, target population, and contextual variables with intervention outcomes and effects. In total, we identified 18 descriptive themes, which we configured into four analytical themes (Supporting Information Appendix SN). These four analytical themes present the synthesis results and are discussed in more detail below.

*Theme 1: Women‐only TVET programmes are preferred by participants*.

The qualitative evidence indicated a consistent theme of women preferring single‐sex TVET programme approaches (Bandiera et al., 2018; Mckenzie et al., 2019; Stangl et al., 2015; Subah‐Belleh Associates, 2010). All three included interventions—the Economic Empowerment of Adolescent Girls and Young Women (EPAG), the Empowerment and Livelihood for Adolescents (ELA) programme in Sierra Leone, and the GET Ahead for Women in Enterprise in Kenya were designed to only include women participants and men were not eligible for participation. In light of this, all qualitative data is based on women's experiences in single‐sex programmes. Reasons expressed for this preference by women for single‐sex TVET programmes included a lack of space for open engagement, safety concerns, and need for peer‐to‐peer exchanges and collective‐building. Demand was expressed for ‘men to see the benefits’ of the programmes, but not for men to be eligible to join them.

Women in the Kenyan GET Ahead for Women in Enterprise programme felt that the inclusion of men would reduce the space for open engagement within the training with participants expressing that ‘I am not of the opinion of including men because some of the women like myself will not be free to share their ideas in the presence of the men. (…) It's better to be women alone because if we were mixed the men would have sold out what the women converse in the training outside and hence, they would be viewed differently’ (Stangl et al., 2015). This fear of negative repercussions against women was reported in the programme evaluation which further recorded incidents of men and community members challenging women about their late return home and status gained from attending the training. Women expressed an explicit fear that should men be included ‘women entrepreneurs would again be forced into domestic responsibilities instead of being able to focus on growing their businesses’ (Stangl et al., 2015).

In addition, a further theme in favour of women‐only TVET programmes focused on the role of peer‐to‐peer interaction and the building of a collective. Kenyan entrepreneurs in particular valued the ‘camaraderie, support and mentorship they received from one another’ (in addition to the freedom to express themselves without the censure of men) (Stangl et al., 2015). The ELA programme in Sierra Leone focused on a similar pathway to change aiming to create women‐only clubs that offer a space where young women can gather and socialise, and thus provide an alternative to spending time with men or other leisure activities (Bandiera et al., 2018). The essence of this peer‐to‐peer focus and emphasis on a safe collective was perhaps best captured in a Kenyan's entrepreneurs' expression that ‘Having the programme with women only helps since we put our thoughts together. For men, the only goal is to get money. As for women, we help one another move to a better status’ (Stangl et al., 2015).

However, there was also dissenting evidence on this theme. In both Liberia and Kenya, trainers and implementers advocated for a mixed‐sex programme approach arguing that the involvement of women's partners is required to change household dynamics and that the role of men in changing structural gender norms cannot be overlooked. A small minority of women in the Kenyan GET Ahead for Women in Enterprise programme agreed with this rationale but the overall opinion of the included women was summarised in the evaluation as the expression of ‘men will spoil it’ (Stangl et al., 2015).

*Theme 2: Enhancing social capital and peer‐to‐peer exchange is a contributing factor, if designed for within TVET*.

A second set of themes identified in the qualitative evidence base expands on the above reported potential of women's peer‐to‐peer exchange and collective building. In addition to the benefits of gendered exchanges aiming to build support structures against restrictive social environments and norms, the qualitative evidence also points to the potential of peer‐to‐peer design elements within TVET programmes more broadly. TVET programme designs that emphasised ‘collective‐building’ and ‘peer‐to‐peer’ exchanges such as mentoring and the embedding within social clubs or women's self‐help groups reported an enhanced social capital and networks of women. This social capital and networks in return were seen as contributing to creating a space for the application of skills in practice (e.g., opening a business, finding a new job) which enhanced the potential and design of the TVET programmes.

Liberia's EPAG programme deliberately designed a pairing/small group approach between girls aiming to improve girls' skill development as well as to promote the creation of social capital, such as friendships, mutual trust and support networks among the girls (Subah‐Belleh Associates, 2010). These groups were paired deliberately consisting of 3–4 girls to encourage after school interaction and relationship building considering location and a mix of skill levels. The programme evaluation found this design element to be appreciated by the girl participants who reported helping each other inside and outside of the classroom activities. As one participant observed ‘There were girls that could not write and felt ashamed to take part … these girls were assisted by the others who could write … and urged them to come to school regularly. With this persuasion and encouragement, we were able to overcome this challenge’. The evaluation report further links these generated networks of girls to exit polls results describing the girl's newfound optimism and belief in themselves, with one trainer noting, ‘now they look like sisters’ (Subah‐Belleh Associates, 2010).

Similar reports on the value and benefit of mentoring for business skills were found in the Kenyan GET Ahead for Women in Enterprise programme. In this context, mentoring was seen as a relevant design element to enhance business skill transfer and development by contextualising these skills in the experience of an established entrepreneur. Finally, in the ELA programme in Sierra Leone embedding TVET components within existing women groups and collectives proved a feasible design strategy. This design strategy provided a direct link to the Theme 1 discussed above.

*Theme 3: TVET and its design benefits from the inclusion of and coupling with LSCB programmes*.

The qualitative evidence on programme design suggests an integration of TVET and LSCB capacity building programmes to be of promise. The assessments of stand‐alone TVET programmes and TVET components within LSCB programmes emphasise the interconnectedness of both types of skill sets; and programme participants, too, express a demand and need for both types of skills to enhance their life capabilities and opportunities. Within TVET programmes participants cited obstacles to the application of these skills to further their life being constrained by personal and structural issues that LSCB programmes focus on; vice‐versa, within LSCB programmes, participants expressed a need for practical applications as well as a pathway for how new skills could lead to economic opportunities.

In Liberia's EGAP programmes, qualitative data and feedback from trainers and implementers highlighted the need to enhance the training's ‘social aspect’ targeting outcomes such as the girls' self‐esteem and social competencies, which fall in the design realm of LSCB programmes rather (Subah‐Belleh Associates, 2010). This sentiment was echoed by the programme's exit poll data which confirmed girls' desire and appreciation for more of the life skills‐related topics. Similar feedback emerged from the TVET programmes in Kenya and Sierra Leone with participants emphasising the need for practical opportunities to apply skills in real world situations, including life skills competencies rather than only technical skills (Bandiera et al., 2018; Stangl et al., 2015).

This more general theme of wanting to apply gained skills in daily contexts seemed to reflect a desire for practical tools and applications to further one's capabilities, personal development and growth. In combination with the qualitative data on LSCB programmes, which mirror this theme, this points to the direction that capacity building design can benefit from intervention packages with multiple components aimed at structural change. Within the qualitative data on TVET programmes, there is indication that this is indeed feasible from a design perspective and promising in practice. All three included TVET programmes referenced a deliberate multicomponent or multi‐faceted design approach to support women's empowerment beyond the mere skill building component:

- The EGAP in Liberia reported four implemented performance enhancing strategies—the pairing/small group approach, mentoring, childcare services and transport/meal allowance—which are recommended as a design package for future interventions.
- The ELA programme in Sierra Leone referred to its multifaceted approach to simultaneously tackle multiple disadvantages young women face, related to having agency over their bodies and barriers to accumulating human capital. This approach combines the provision of life skills, vocational skills and microfinance, delivered within a social club and safe space for women.
- The GET Ahead for Women in Enterprise programme in Kenya described itself as a management training programme with a strong gender component; that is, the programme is designed specifically with a gender lens throughout and aims to address both the practical and strategic needs of low‐income women in enterprise by strengthening their basic business and people management skill as well as supporting networking and relationship building.

*Theme 4: A bundle of key implementation considerations apply across contexts*.

Across the TVET programmes a number of implementation considerations were reported repeatedly as contributing factors to programme success. These implementation considerations are fairly programmatic and granular. They are reported below as a bundle of considerations suggested across individual studies along the implementation cycle:

- Design and implement a tailored and sensitive awareness‐raising and outreach campaign to attract participants into the programme. For example, female entrepreneurs in the GET Ahead for Women in Enterprise mentioned the sensitive and respectful approach of recruiters that gave them a positive first impression of the programme including that they were approached directly at their own place of business. As part of this programme, implementers further ran an invitation choice experiment to test the design of three different intervention types. The results of this trial were, however, inconclusive.
- Identify and select a central and safe location that is easily accessible (e.g., via public transport, within walking distance of participants). In Kenya, some women part of the GET Ahead for Women in Enterprise had to leave their homes as early as 3:00am to reach the training venue via public transport; a fact which undermined participation and satisfaction. Programmes in Sierra Leone and Liberia mitigated this issue by identifying and renting facilities within villages and central transport hubs.
- Provision of an allowance to cover transport and possibly offset opportunity costs of attending. This implementation criterion was regarded as the most important aspect by women participants in the GET Ahead for Women in Enterprise in Kenya. In Liberia, too, female trainees rated the provided allowance as a key incentive and enabler for attendance in the EPAG programme.
- Provision of childcare services at the training venue. This implementation criterion featured prominently within the Kenyan and Liberian TVET programmes and was equally regarded as a strong enabler of attendance by female participants
- Training curricula and materials require careful adaptation to local context and translation into local languages. Participants of both the Liberian EGAP and Kenyan GET Ahead for Women in Enterprise complained about the use of foreign languages and irrelevant examples and contexts in the provided materials.
- The skilfulness and approach of programme trainers was reported as a key implementation element and driver of programme satisfaction across the included studies.

The above present a list of programmatic implementation factors reported in the qualitative evidence base. Within these the Liberian EGAP and Kenyan GET Ahead for Women in Enterprise made deliberate attempts to mitigate the gendered nature of some of these factors. This included the use of gender‐sensitive outreach and communication materials in both programmes; the provision of childcare in both programmes; the selection of the programme location for women's access in Liberia; and the adaptation and design of a gender‐specific curriculum in Kenya. Arguable, it should be feasible to apply a gendered lens to all identified implementation considerations in future programme design.

##### 5.3.8.4 Discussion

The quantitative meta‐analysis of TVET programmes revealed positive significant effects on the capacity of women entrepreneurs; women's increased access to and ownership of assets, credit, and income; and women having improved attitudes, self‐image, and confidence. The remainder of outcomes were nonsignificant. This included empowerment related outcomes such as women having more control over their bodies; women's freedom of movement and association; women's participation in decision‐making at the household and community level and direct measures of women's empowerment, which were all found to be nonsignificant.

A complementary qualitative synthesis was carried out to explore explanations and recommendations that may help design more effective TVET interventions that can go beyond narrow outcomes on skills development and positive attitudes targeting broader empowerment objectives:

- The qualitative evidence base indicated a need to design additional programmes components within TVET to target outcomes beyond individual skill development and improved attitudes. In particular, TVET programmes were designed as women‐only spaces—a design feature strongly preferred by women but flagged by implementers and decision‐makers as a potential barrier for programmes to translate into boarder empowerment outcomes. Not involving men by design in programmes might exclude a pathway for change and negate the translation and application of skills development into progress on household power relations and women's empowerment within communities.
- A design feature suggested by the qualitative synthesis which might support a translation of skills development outcomes refers to the use of mentoring and ‘collective‐building’ formats such as women's self‐help groups and clubs. These programme components can allow women to engage in peer‐to‐peer exchanges and to form relationships and networks that allow support to each other beyond the technical skills development. These components thereby add a programme dimension more aligned with targeting broader empowerment outcomes and creating multiple pathways for change.
- Overall, the qualitative evidence contextualised with the quantitative results does indicate that, unless designed for specifically within the programme composition and implementation, outcomes beyond the direct objective of TVET interventions (i.e., skills) are difficult to achieve; in particular if they depend on changing entrenched social norms and practices.

##### 5.3.8.5 Summary of findings and discussion

We included 10 studies in eight countries in South Asia and Sub‐Saharan Africa. We were able to examine effects on the following outcomes: Women have increased access and ownership to assets, credit and income, women can access decent work (formal and informal employment), improved capacity of women entrepreneurs, increased participation in decision making by women at the household or community level, including during crisis response, women have improved attitudes, self‐image and confidence. Our included studies report against all secondary outcomes (Resources, Agency and Achievement) and seven immediate outcomes for our review. Overall, the GRADE assessments generally indicate a range of very low to high certainty in this body of evidence.

The 10 quantitative studies were further detailed by five linked qualitative studies on the impact of TVET programmes. The meta‐analysis results align with the qualitative results; both suggest that women‐only TVET programmes have a higher positive and significant impact. Additionally, qualitative findings suggest that bundling components together, such as the training and sensitisation, can result in more positive and long‐lasting outcomes. Designing programmes with a peer exchange or social skills component also holds potential to bolster positive outcomes; girls or women are able to apply skills in practice and with the support of their peers. Table 39 presents the GRADE review of our findings (Table 34).

**Table 34:** GRADE summary of findings and certainty of evidence on TVET

Certainty assessment							Sample size	Effect	Certainty	Importance
No. of studies	Study design	Risk of bias	Inconsistency	Indirectness	Imprecision	Other considerations		Absolute (95% CI)		
*(AB1) Increased capacity of women to understand and use financial, banking and business services effectively*										
1	RCT	Not serious	Serious^a^	Not serious	Not serious	None	2400	SMD 0.09 SD higher (0.03 lower to 0.15 higher)	⊕⊕⊕◯ MODERATE	Important, but not critical
*(AB2) Women have increased access and ownership to assets, credit and income*										
10	RCT‐10	Very serious^b^	Very serious^c^	Not serious	Not serious	Publication bias strongly suspected	7181	SMD 0.06 SD higher (0.01 higher to 0.11 higher)	⊕◯◯◯ VERY LOW	Important, but not critical
*(AC1) More women engaged in other micro, small and medium‐sized enterprises*										
1	RCT	Not serious	Serious^a^	Not serious^d^	Serious^e^	None	5398	Two small, positive effect estimates with a 95% CI range of −0.05 to 0.19	⊕⊕◯◯ LOW	Important, but not critical
*(AC2) Initiatives supported that facilitate women to access decent work (formal and informal employment), including people with disabilities*										
7	RCT‐6 QED‐1	Serious^f^	Very serious^g^	Not serious	Serious^h^	None	91,564	SMD 0.07 SD higher (0.07 lower to 0.21 higher)	⊕◯◯◯ VERY LOW	Important, but not critical
*(AC3) Improved capacity of women entrepreneurs*										
5	RCT‐5	Very serious^i^	Not serious	Not serious	Not serious	None	34,761	SMD 0.14 SD higher (0.09 higher to 0.19 higher)	⊕⊕◯◯ LOW	Important, but not critical
*(BA1) Women have improved success in the workplace*										
1	RCT	Very serious^l^	Serious^a^	Not serious	Not serious	None	2474	SMD 0.23 SD higher (0.15 higher to 0.31 higher)	⊕◯◯◯ VERY LOW	Important, but not critical
*(BA2) Women have more and better control over their bodies and sexual health*										
2	RCT‐1 QED‐1	Serious^p^	Very serious^q^	Not serious	Serious^r^	None	21,941	SMD 0.26 SD higher (0.36 lower to 0.88 higher)	⊕◯◯◯ VERY LOW	Critical
*(BA3) Women have increased freedom of movement and association*										
5	RCT‐4 QED‐1	Very serious^s^	Not serious	Not serious	Not serious	Publication bias strongly suspected	12,015	SMD 0.04 SD higher (0.02 lower to 0.11 higher)	⊕◯◯◯ VERY LOW	Limited importance
*(BA5) Women have more positive attitude towards taking action to claim their rights*										
1	RCT	Very serious^w^	Serious^a^	Not serious	Not serious	None	1210	SMD 0.03 SD higher (0.08 lower to 0.14 higher)	⊕◯◯◯ VERY LOW	Limited importance
*(BA6) Reduced percentage of women agreeing with certain reasons that justify violence against women and girls*										
2	RCT‐1 QED‐1	Very serious^x^	Not serious	Not serious	Not serious	None	13,870	SMD 0.03 SD lower (0.13 lower to 0.06 higher)	⊕⊕◯◯ LOW	Critical
*(BA7) Women are equipped with better life skills that allow them to be prepared for crisis or shocks and recover from them*										
3	RCT‐2 QED‐1	Very serious^y^	Not serious	Not serious	Not serious	None	1558	SMD 0.04 SD higher (0.01 lower to 0.08 higher)	⊕⊕◯◯ LOW	Important, but not critical
*(BA8) Reduced instances of child or forced marriage*										
2	RCT‐2	Very serious^y^	Not serious	Not serious	Not serious	None	15,424	SMD 0.17 SD higher (0 to 0.34 higher)	⊕⊕◯◯ LOW	Critical
*(BB1) Increased participation in decision making by Women at the household or community level, including during crisis response*										
4	RCT‐2 QED‐2	Very serious^z^	Very serious^aa^	Not serious	Serious^ab^	None	9763	SMD 0.01 SD lower (0.12 lower to 0.1 higher)	⊕◯◯◯ VERY LOW	Limited importance
*(BB2) Women participate more in their community*										
1	QED	Serious^ac^	Serious^a^	Not serious	Not serious	Very strong association	804	SMD 1.11 SD higher (0.97 higher to 1.25 higher)	⊕⊕⊕⊕ HIGH	Limited importance
*(BB4) Women increasingly form their own organisations*										
1	RCT	Not serious	Serious^a^	Not serious	Serious^ad^	None	5400	0 (0 to 0)	⊕⊕◯◯ LOW	Limited importance
*(CA2) Increased representation of women in local and subnational civil and political processes, including during peacebuilding and post conflict restoration*										
2	QED‐2	Very serious^ae^	Serious^af^	Not serious	Not serious	None	24,735	SMD 0.29 SD higher (0.03 higher to 0.55 higher)	⊕◯◯◯ VERY LOW	Limited importance
*(CA8) Increased community support for women's and children's human, economic and legal rights*										
2	RCT‐1 QED‐1	Very serious^x^	Serious^ag^	Not serious	Not serious	None	992	SMD 0.07 SD higher (0.04 higher to 0.18 higher)	⊕◯◯◯ VERY LOW	Important, but not critical
*(CB3) Women have improved attitudes, self‐image and confidence*										
6	RCT‐5 QED‐1	Very serious^ah^	Very serious^ai^	Not serious	Not serious	None	3993	SMD 0.17 SD higher (0.01 higher to 0.33 higher)	⊕◯◯◯ VERY LOW	Limited importance
*(CB5) Decreased violence/discrimination at the household level*										
1	QED	Serious^aj^	Serious^a^	Not serious	Serious^ak^	None	804	SMD 0.09 SD higher (0.05 lower to 0.22 higher)	⊕◯◯◯ VERY LOW	Critical
*(CB6) Safer and more secure household, communities and areas/territories for women, girls, men and boys*										
1	RCT	Serious^al^	Serious^a^	Not serious	Not serious	None	5046	0 (0 to 0)	⊕⊕◯◯ LOW	Critical
*(CB7) Reduced frequency and distribution of types of violence by an intimate partner*										
2	RCT‐2	Very serious^j^	Not serious	Not serious	Serious^k^	None	25,864	SMD 0.09 SD higher (0.02 lower to 0.2 higher)	⊕◯◯◯ VERY LOW	Critical
*(CB8) Improved quality of relationships between women and their household and community members*										
3	RCT‐3	Very serious^m^	Serious^n^	Not serious	Serious^o^	None	2602	SMD 0.01 SD higher (0.08 lower to 0.1 higher)	⊕◯◯◯ VERY LOW	Limited importance
*(CC1) Empowerment/Equality Index*										
4	RCT‐3 QED‐1	Very serious^t^	Very serious^u^	Not serious	Serious^v^	None	17,030	SMD 0.13 SD higher (0.01 lower to 0.27 higher)	⊕◯◯◯ VERY LOW	Important, but not critical

Abbreviations: CI, confidence interval; GRADE, Grading of Recommendations, Assessment, Development and Evaluations; QED, quasi‐experimental design; RCT, randomised controlled trial; RoB, risk of bias; SMD, standardised mean difference; TVET, technical and vocational education and training.

^a^ All single studies downgraded once for inconsistency.

^b^ Downgraded twice since although only one of 12 studies is not a RCT 5 of them are high risk of bias and 3 raise some concerns related to risk of bias.

^c^ Downgraded twice because studies are on both side of the threshold and CI are not overlapping. The $I^{2}=80.04\%$ and the *Q*‐test concludes that the true outcomes appear to be heterogeneous.

^d^ Downgraded because there are point estimates on either side of the threshold with a wide berth of CIs.

^e^ Downgraded because there are point estimates on either side of the threshold with a wide berth of CIs.

^f^ Downgraded because three of seven included studies present high risk of bias cross various domain criteria, and an additional two are of some concerns related to risk of bias. Deviation from intended intervention is of particular concern.

^g^ Downgraded twice because this group of studies estimates a wide range of effect sizes, with limited CI overlap and both positive and negative point estimates.

^h^ While the CI are reasonably small, the CI cross the threshold, and the small point estimate is not representative of the range of estimates included in the meta‐analysis.

^i^ Downgraded twice because half the body of evidence comes from high risk of bias studies, and an additional two of six are from medium or uncertain risk of bias.

^j^ Downgraded three times. (1) Only two studies and both of them are high risk of bias. (2) High risk on three RoB domains. (3) Removing either study would impact meta‐analysis result.

^k^ Downgraded once because although sample size >400, *p* = .10.

^l^ A high risk of bias was found in the unit of analysis criterion, and some concerns related to risk of bias in the selection and performance criteria.

^m^ Downgraded two times. (1) The three studies are RCTs but two of them are high risk and one raises some concerns related to risk of bias. (2) Removing either of the high‐risk studies would impact meta‐analysis.

^n^ Downgraded once. (1) Effects sizes are on both side of the threshold and CI range is wide and not systematically overlapping. (2) $I^{2}=45.70\%$ but according to *Q*‐test there was no significant amount of heterogeneity.

^o^ Downgraded once because although sample size >400, *p* = .89.

^p^ Downgraded to reflect the high and some concerns related to risk of bias studies in this group. There is particular concern with regard to deviation from intended intervention. The high risk of bias study strongly influences the final effect estimate.

^q^ There is considerable variation in the effect estimates on both sides of the threshold. The difference is so large that the wide CIs do not cross.

^r^ Downgraded due to wide CI that cross the threshold of interest widely on both sides.

^s^ Downgraded twice because three of five studies in this group are of high risk for bias, and another is of some concerns related to risk of bias There is particular concern for selection bias and performance bias.

^t^ Downgraded three times. (1) Mix of RCTs and QED. (2) All studies are high risk of bias covering five RoB domains. (3) Removing either study would impact the meta‐analysis.

^u^ Downgraded twice. (1) We have effect sizes on both side of the threshold and a wide range of CI not systematically overlapping. (2) $I^{2}=91.17\%$ and according to *Q*‐test the true outcomes appear to be heterogenous.

^v^ Downgraded once because although sample size >400, *p* = .07.

^w^ High risk biases were found in the unit of analysis and selection bias criteria. The deviations from intended interventions and outcome measurement bias criteria were unclear.

^x^ Downgraded twice because all evidence in this group comes from high risk of bias studies.

^y^ Downgraded because half of the evidence comes from high risk of bias studies while the other half is uncertain.

^z^ Downgraded 3 times. (1) Mix of QED and RCTs. (2) Three of the four studies are high risk of bias covering three different RoB domains. (3) Removing either study would impact the results of the meta‐analysis.

^aa^ Downgraded twice. (1) We have effect sizes on both side of the threshold and a wide range of CI not systematically overlapping. (2) $I^{2}=86.72\%$ and according to *Q*‐test the true outcomes appear to be heterogenous.

^ab^ Downgraded once because *p* = .84 although sample size >400.

^ac^ BB2 Women participate more in their community.

^ad^ Downgraded because point estimates vary considerably, and Cis cross widely into both sides of the threshold.

^ae^ Downgraded three times. (1) The two studies are QED. (2) One of the studies is high risk of bias and the other one is low risk. (3) Removing either study would affect the meta‐analysis.

^af^ Downgraded once as the effect sizes are on the same side of the threshold and the CI overlap but $I^{2}=64.93\%$ indicates risk of heterogeneity.

^ag^ Downgraded because of varied point estimates and CI that cross both sides of the threshold.

^ah^ Downgraded three times. (1) Although most of the studies are RCTs only one of them is low risk of bias and one raises some concerns related to risk of bias. (2) Four studies out of six are high risk of bias covering three RoB domains. (3) Removing any of the high risk would impact the results of the meta‐analysis.

^ai^ Downgraded twice. (1) Effects sizes are on both side of the threshold and CI range is wide and not systematically overlapping. (2) $I^{2}=96.13\%$ and *Q*‐test concludes that the true outcomes appear to be heterogeneous.

^aj^ Some concerns related to risk of bias of reporting bias, and unclear deviations from intended interventions and outcome measurement bias.

^ak^ Downgraded due to wide range of CIs.

^al^ Some concerns related to risk of bias was found in the unit of analysis criterion. The deviations from intended interventions and outcome measurement bias criteria were unclear.

### 5.4 Effects of interventions under the UNSCR protection pillar

The protection pillar includes a set of interventions that create, facilitate access to or build awareness of and support for legal or social protections for women's and girls' rights. This also includes behavioural, legal and environmental interventions that aim to reduce women and girls' risk of experiencing SGBV.

In our SR, this pillar gathered the following types of interventions covering 8306 beneficiaries:

- AWPSs
- Sensitisation campaigns

**Figure 112:**
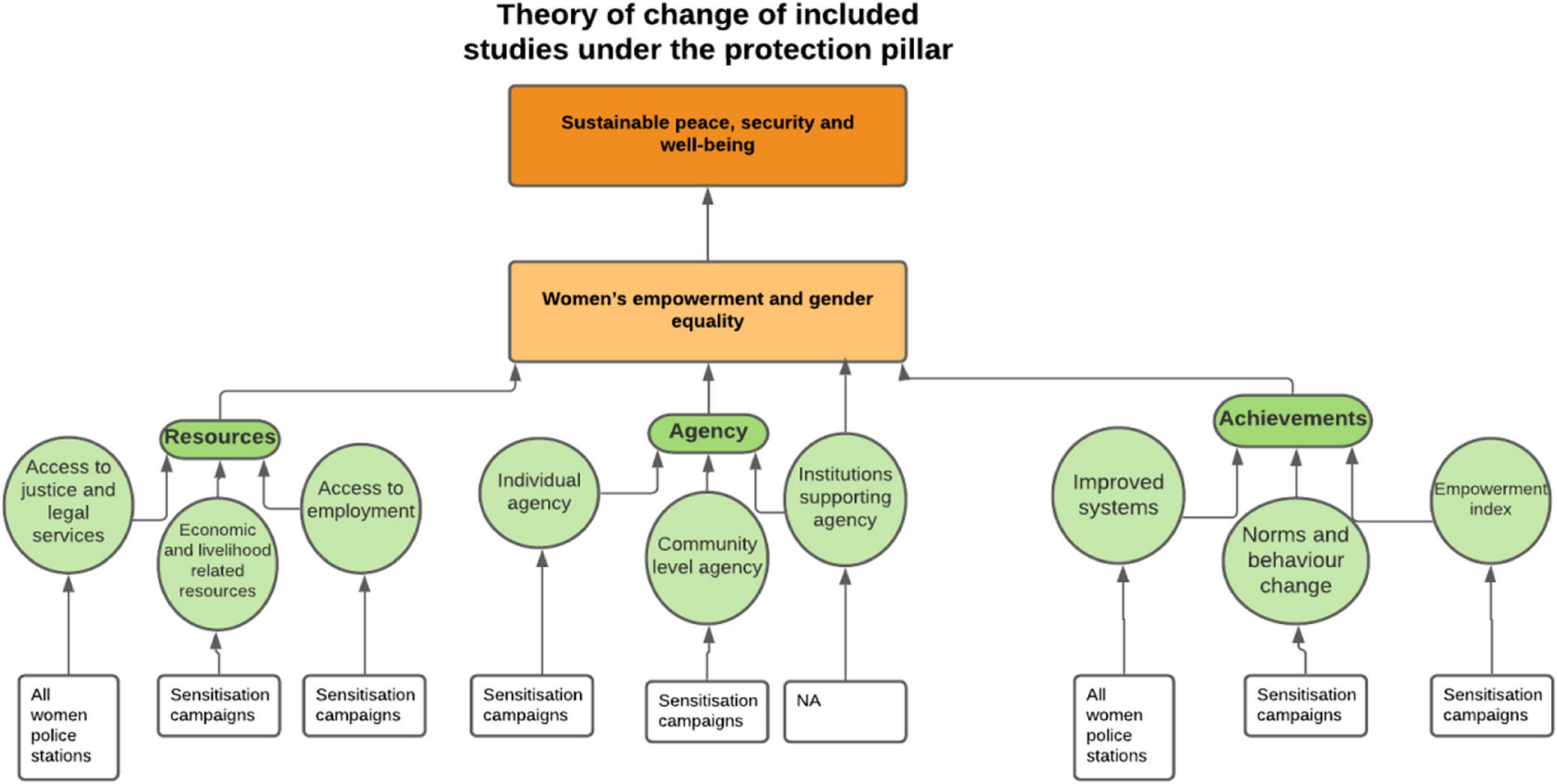
Theory of change for included studies under the protection pillar

This section provides the findings of our synthesis of the 10 included studies evaluating the effect of these interventions on gender equality, women's empowerment and peaceful and inclusive societies. The section is organised by each intervention group. Each sub‐section begins with a description of the intervention groups, their activities and ToC, followed by descriptive results and the findings addressing our research questions. Figure 112 provides a summary of the outcomes and targeted effects of the interventions included under the protection pillar:

#### 5.4.1 AWPSs

AWPSs are when policewomen can be posted at a standard station or all women station, the main distinction being that the latter is run entirely by women staff. All women stations are additive; a victim may register a crime in the standard police station near where the crime occurred or to the all‐women station in the district headquarters. This form of policing provides safe spaces for women willing to use formal support services. They typically employ women officers purposely trained to handle gender‐based violence crimes (Amaral et al., 2019). Other services such as psychological, social and legal advice, education and prevention or mediation can be offered in those stations (Perova & Reynolds, 2017). Table 40 presents the characteristics of the included studies.

##### 5.4.1.1 How do AWPSs affect gender equality, women's empowerment and Peace outcomes?

**Figure 113:**
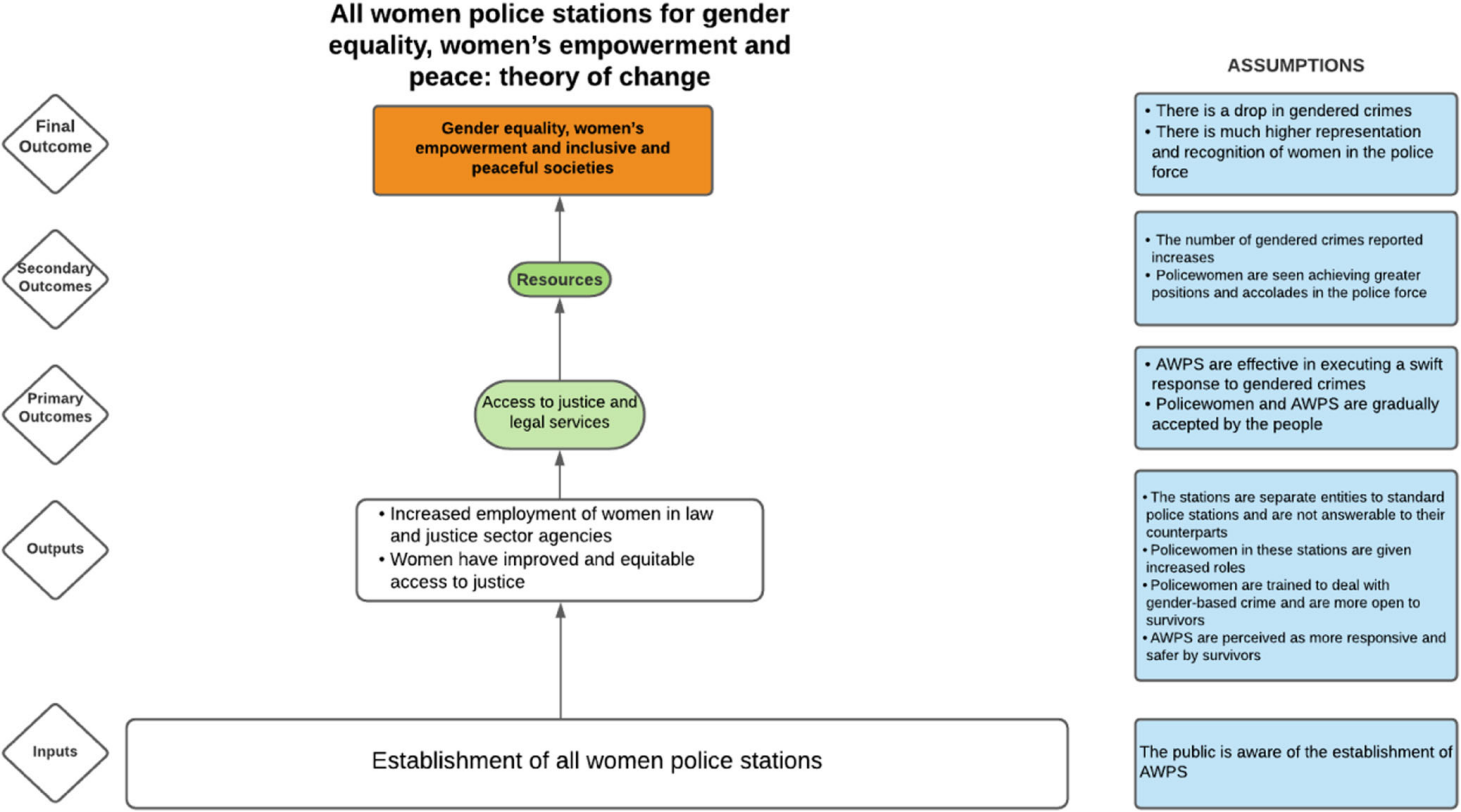
All women police stations for gender equality, women's empowerment and peace: Theory of change

Figure 113 maps out the causal chain of how AWPSs may improve gender equality, women's empowerment and peace outcomes.

AWPSs create gender equitable societies through two major pathways. (1) Safe spaces for women: AWPSs lower the cost of reporting crimes for women as they offer a more hospitable environment with women officers that are typically trained to handle gendered crimes and are separate from traditional police stations that are generally corrupt, dominated by men and are perceived to have a greater stigma against gendered crimes. (2) Representation: AWPSs promise to increase responsibilities of women in the police force and gradually remove previously held prejudices that may be hindering their advancement.

Our review only included one study (Jassal, 2020) that explored the causal effects of AWPS. The study refuted the idea of AWPS leading to gender inclusive outcomes and argued for the opposite. They distinguish *inclusive* mechanisms of representation (e.g., quotas) from representation mechanisms that employ *separation*, such as the AWPS. They posit that AWPS offer segregation rather than integration and hence, hinder women's advancement in the police force and allow standard police stations to pass on gender‐based crimes to women police stations that are relatively less widespread, increasing the cost of reporting for the victim.

##### 5.4.1.2 Description of included studies

We included one study reported in one paper that evaluated one programme.

*Population*

AWPSs, by virtue of the concept, targeted women and girls. They comprise an all women staff and specialise in gender‐based crimes.

The included studies evaluated one programme and trial in India. The low number of studies including a focus on AWPSs is mainly because this is a type of intervention primarily implemented in Latin America, where less countries met the inclusion criteria for our review.

*Intervention, inputs and activities*

The included study (Table 40) evaluated the following activity:

- AWPSs (*n* = 1): Establishment of AWPSs in Haryana, India (Table 35).

**Table 35:** All‐women police station features of included studies

Study	Activity/input	Length of treatment	Intervention frequency
Jassal (2020)	Establishment of all women police stations in the state of Haryana	‐	‐

*Comparison*

The included study compared treated groups to comparison groups receiving no intervention. The study did not include multiple treatment arms.

*Outcomes*

The included study reported on a number of relevant outcomes, including the following:

- Increased employment of women in law and justice sector agencies (*n* = 1): Increased recruitment practices for women, nondiscrimination, more women police officers, judges, lawyers, community legal volunteers and so on. Through this employment, Women have an increased influence in this sector.
- Women have improved and equitable access to justice (*n* = 1): Women are able to file cases of injustice and raise cases, receive information, have judicial support and address offenses. Women's cases are not dropped, meaning that cases (especially relating to SGBV or women's rights, economic or otherwise) are followed up at all levels. This could mean the creation or editing of a referral pathway or improved communication mechanisms between actors and service providers.

The division of the immediate and secondary outcomes in asset transfer is reported in Table 41 (Table 36).

**Table 36:** Summary of secondary and immediate outcome

Secondary outcome category	Immediate outcome	Number of studies
Resources material, human and social resources which serve to enhance the ability to exercise choice	Access to justice and legal services	1
	Economic and livelihood related resources	0
	Access to employment	0
Agency ability to define one's goals and act upon them and operationalised decision‐making	Individual agency	0
	Community level agency	0
	Institutions supporting agency	0
Achievement ways of being and doing which can be realised by different individuals	Improved systems	1
	Norms and behaviour change	0
	Empowerment index	0

*Study design*

**Figure 114:**
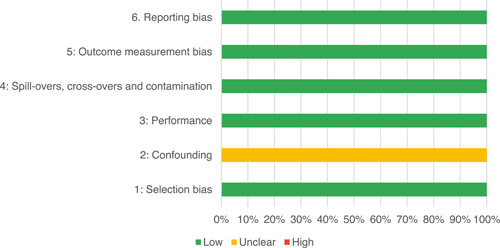
Quasi‐experimental design all‐women police station risk of bias assessment

The only study in this intervention group used a QED (Jassal, 2020) which had no major concerns of risk of bias. We only identified one domain for which there may be some issues, this was the confounding domain given that authors did not report a balance test for observations at both sides of the cut‐off point used for the regression discontinuity estimated to evaluate the impact of the AWPS intervention (Figure 114).

*Qualitative studies, process evaluations and project documents*

We have identified one additional document related to one programme covered by the AWPS group of studies. This single qualitative study was marked as moderate empirical quality. Given this insufficient number of linked qualitative studies (*n* = 1) related to the AWPS interventions, we were unable to conduct a qualitative evidence synthesis for this intervention group.

##### 5.4.1.3 Synthesis of findings

The following subsection presents the results of effectiveness of AWPSs on gender equality, women's empowerment and peace outcomes.

*Quantitative findings*

*Effects of* AWPS*s on increased employment of women in law and justice sector agencies*

Jassal's (2020) quasi‐experimental study in India was the only study evaluating the impact of AWPSs on increased employment of women in law and justice sector agencies. They examined the daily proportion of cases assigned to women. There was a large, and statistically significant negative effect (*g *=* *−0.45, [95% CI: −0.58 to −0.32]), and we assessed the study as having some risk of bias concerns.

*Effects of* AWPS*s on women having improved and equitable access to justice*

Jassal's (2020) quasi‐experimental study in India was also the only study evaluating the impact of AWPSs on women having improved and equitable access to justice. There was a very small, and not statistically significant impact (*g *=* *0.07, [95% CI: −0.06 to 0.19]), and we assessed the study as having some risk of bias concerns.

##### 5.4.1.4 Summary of findings and discussion

We included one study in South Asia that evaluated the effect of AWPSs in India. We were able to examine effects on the following outcomes: Increased employment of women in law and justice sector agencies and women have improved and equitable access to justice. Our included studies report against two of the three secondary outcomes (Resources, Agency and Achievement) and two of the nine immediate outcomes in our review. Overall, the GRADE assessments generally indicate a very low to low certainty in this body of evidence. We identified only one linked qualitative study and were unable to conduct a qualitative evidence synthesis related to AWPS interventions. Table 42 presents the GRADE review of our findings (Table 37).

**Table 37:** GRADE summary of findings and certainty of evidence on all‐women police stations

Certainty assessment							Sample size	Effect	Certainty	Importance
No. of studies	Study design	Risk of bias	Inconsistency	Indirectness	Imprecision	Other considerations		Absolute (95% CI)		
*(AA2) Increased employment of women in law and justice sector agencies*										
1	QED	Serious^a^	Serious^b^	Not serious	Serious^c^	Strong association	949	SMD 0.44 SD lower (0.58 lower to 0.32 lower)	⊕⊕◯◯ LOW	Limited importance
*(CA7) Women have improved and equitable access to justice*										
1	QED	Serious^a^	Serious^b^	Not serious	Serious^c^	None	1898	SMD 0.06 SD higher (0.05 lower to 0.19 higher)	⊕◯◯◯ VERY LOW	Important, but not critical

Abbreviations: CI, confidence interval; GRADE, Grading of Recommendations, Assessment, Development and Evaluations; QED, quasi‐experimental design; SMD, standardised mean difference.

^a^ Downgraded once due to uncertainty surrounding confounding.

^b^ All single studies downgraded once for inconsistency.

^c^ Downgraded once because of very wide confidence intervals.

#### 5.4.2 Sensitisation campaigns

Sensitisation campaigns include the provision of access to information and messaging and/or raised awareness on specific topics through multimedia outlets like radio, posters and speakers (Gholami et al., 2017). Separate from discussion groups and community dialogues, sensitisation campaigns focus purely on the dissemination of messages. The interventions themselves offer no space for dialogue.

##### 5.4.2.1 How do sensitisation campaigns affect gender equality, women's empowerment and Peace outcomes?

**Figure 115:**
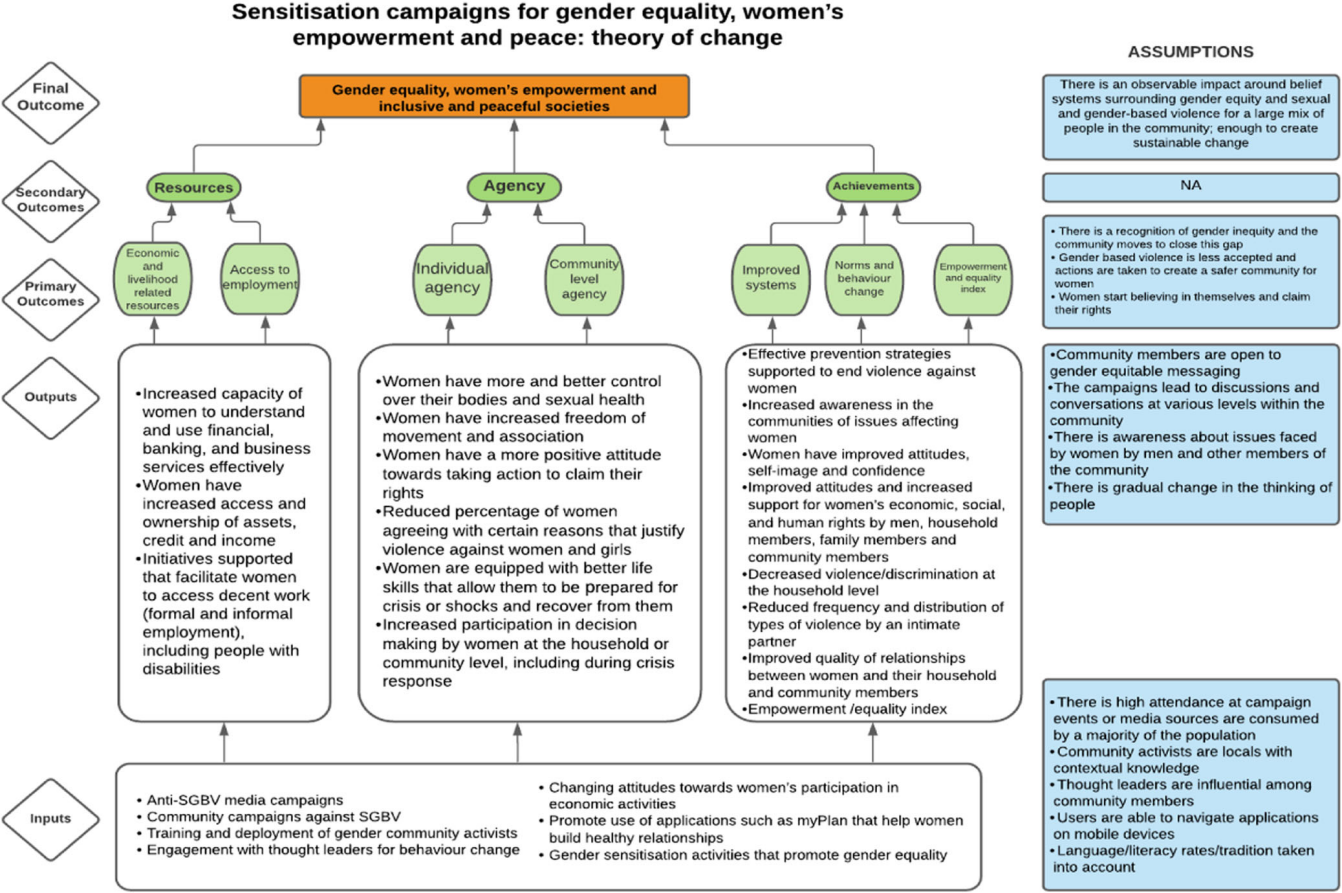
Sensitisation campaigns for gender equality, women's empowerment and peace: Theory of change

Figure 115 maps out the causal chain of how sensitisation campaigns may improve gender equality, women's empowerment and peace outcomes.

Sensitisation campaigns gradually uproot traditional norms and misconceptions surrounding SGBV and concepts of gender equity. Media campaigns, community campaigns, deployment of community activists (CAs) and engagement with community leaders all sought to gradually change predispositions surrounding concepts of gender by initiating conversations and spreading awareness about such topics at different ecological levels. Changing beliefs lead to more economic initiatives for women, women having more control over their bodies, women having increased freedom of movement, increased decision making by women, support for effective prevention strategies to stop SGBV, decreased violence and discrimination at the household level and improved quality of relationships for women. Consequently, women have increased agency, and, at the community level, improved systems are employed and sustainable change in norms and behaviours is achieved. As a result, there is a more equitable and inclusive society.

##### 5.4.2.2 Description of included studies

We included seven studies in nine different papers that evaluated the effect of six identified programmes. We included more than one paper that evaluated the same programme if the author(s) undertook different analyses or reported different outcomes over several papers.

*Population*

Sensitisation campaigns in our review, in general, targeted the collective consciousness of the members of a community to create a more inclusive community. Two interventions aimed to create change at the household level by targeting couples. Men's Action to Stop Violence Against Women focussed only on men. The included studies evaluated six different programmes and trials in South Asia and Sub‐Saharan Africa: Bangladesh, India, Kenya, Rwanda and Uganda.

*Intervention, inputs and activities*

The included studies evaluated a range of sensitisation activities and inputs including:

- Anti‐SGBV media campaign (*n* = 2): Utilised radio, television and other instruments of mass media to spread messages against SGBV and for gender equity.
- CAs (*n* = 2): Trained and deployed CAs that are responsible for sensitising community members on violence against women and girls and promoting gender equitable norms.
- Community campaigns (*n* = 1): Inclusive community campaigns spreading gender equitable messaging.
- Engagement with thought leaders (*n* = 1): Created change by spreading awareness through community leaders that are well respected by people.
- Applications for SGBV preventions (*n* = 1): Used the myPlan app to sensitise women about healthy relationships and strategies to mitigate IPV.
- Gender sensitisation activities (*n* = 1): Series of activities that led to the improvement in the status/empowerment of women and gender parity between women and men.
- Savings groups and safe spaces (*n* = 1): Specific use of such tools with the overall aim to promote women's partners acceptance of their participating in economic development activity, thus focusing on norm change at the household level (Table 38).

**Table 38:** Activities and design features of included sensitisation studies

Study	Activity/Input	Length of treatment	Intervention frequency
Abramsky et al. (2014)	Training and deployment of gender community activists	33.5 months	Weekly
Das et al. (2012)	Community campaigns against SGBV	‐	‐
Decker et al. (2020)	Promote use of applications such as myPlan that help women build healthy relationships and flag unhealthy practices in their relationships	7 months	‐
Dunkle et al. (2020)	Changing attitudes towards women's participation in economic activities	36 months	‐
Green et al. (2020)	Anti‐SGBV media campaigns	2 months	Weekly
Green et al. (2018)	Anti‐SGBV media campaigns	2 months	Weekly
Quisumbing et al. (2020)	Gender sensitisation activities to promote gender equality	29 months	Monthly
Schensul et al. (2015)	Engagement with thought leaders for behaviour change	84 months	Weekly
Watts et al. (2015)	Training and deployment of gender community activists	33.5 months	Weekly

Abbreviation: SGBV, sexual and gender‐based violence.

*Comparison*

All our included studies compared treated groups to comparison groups receiving no intervention. Four studies included multiple treatment arms.

*Outcomes*

The included studies reported on a number of relevant outcomes, including the following:

- Women have a more positive attitude towards taking action to claim their rights (*n* = 3): Women feel entitled to be engaged and given the leadership capacity and knowledge to claim their rights and take action on relevant issues. Increased self‐efficacy and autonomy. Increased opportunities for women to claim their rights including as a result of education and sensitisation. Women are engaged and given the leadership capacity and knowledge to claim their rights and take action on relevant issues.

- Reduced percentage of women agreeing with certain reasons that justify violence against women and girls (*n* = 2): Women are sensitised and empowered to recognise harmful social norms, particularly relating to intimate partner violence (IPV). As a consequence of this sensitisation, women are less keen to agree with reasons justifying wife‐beating and are equipped with resources to claim their right and prosecute perpetrators.
- Increased awareness in communities of the issues affecting women (*n* = 2): Community members are easily able to recognise and support women, by having knowledge and understanding of these particular issues.
- Improved attitudes and increased support for women's economic, social and human rights by men, household and family (*n* = 4): These positive attitudes can be shaped by sensitisation and education to shift social norms, particularly relating to marginalised groups. As a result, they are aware of the specific needs of these groups and take an active part in reducing inequalities.
- Reduced frequency and distribution of types of violence by an intimate partner (*n* = 4): Types of violence in this case include four types of IPV: physical violence, sexual violence, stalking and psychological aggression. These incidences are reduced over time due to a bundle of interventions that focus on both support for survivors and sanctioning perpetrators.

The division of the immediate and secondary outcomes is reported in Table 44 (Table 39).

**Table 39:** Summary of secondary and immediate outcomes

Secondary outcome category	Immediate outcome	Number of studies
Resources material, human and social resources which serve to enhance the ability to exercise choice	Access to justice and legal services	0
	Economic and livelihood related resources	1
	Access to employment	1
Agency ability to define one's goals and act upon them and operationalised decision‐making	Individual agency	4
	Community level agency	1
	Institutions supporting agency	0
Achievement ways of being and doing which can be realised by different individuals	Improved systems	2
	Norms and behaviour change	6
	Empowerment index	2

*Study design*

**Figure 116:**
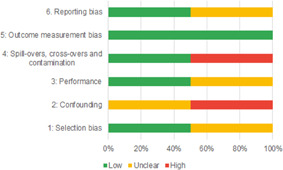
Sensitisation campaign studies quasi‐experimental design risk of bias assessment

Two sensitisation campaigns studies used a QED (Das et al., 2012; Schensul et al., 2015). Both studies were assessed as having a high risk of bias. Reasons for this assessment included not reporting a Hausman test to suggest there is no evidence of endogeneity of the instrument (Das et al., 2012), and potential contamination of other activities run by NGOs present in the area of intervention (Schensul et al., 2015). We did not identify any other major issues, however in each of the following categories at least one study did not provide enough information to justify the absence of selection, performance or reporting bias (Figure 116).

**Figure 117:**
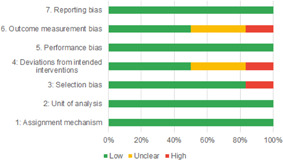
Sensitisation campaigns randomised controlled trial risk of bias assessment

Six studies used an experimental design. This included Abramsky et al. (2014), Decker et al. (2020), Dunkle et al. (2020), Green et al. (2020), Quisumbing et al. (2020) and Watts et al. (2015). We assessed two of these studies as having a low risk of bias, another two as having a high risk of bias, and the rest had some concerns. As detailed in Figure 117, high risk of bias was observed in selection, deviations from intended intervention, and outcome measurement domains. Selection bias could have occurred because of differential attrition between treatment and control groups (Dunkle et al., 2020), deviations from interventions were observed because part of the control group in a waitlist received the intervention at the same time as the treatment group (Abramsky et al., 2014), and measurement error was an issue because of potential reporting bias in self‐reported outcomes (Abramsky et al., 2014). However, these limitations were not common to more than one study and we did not identify any issues with the assignment mechanism of the included studies, the unit of analysis or the reporting of analysis and outcomes.

*Qualitative studies, process evaluations and project documents*

We identified 10 additional documents related to four programmes covered by the sensitisation campaign studies:

- myPlan Kenya (Kenya): one descriptive quantitative document
- Indashyikirwa—‘Agents of Change' (Rwanda): three qualitative studies and two process evaluations
- Men's Action to Stop Violence Against Women (MASVAW) (India): one qualitative study
- SASA! (Uganda): two qualitative studies and one descriptive quantitative study

Four of the 10 studies were marked as high quality, with the remainder of studies each falling into the moderate (*n* = 3) and low (*n* = 3) categories.

##### 5.4.2.3 Synthesis of findings

The following subsection presents the results of effectiveness of sensitisation groups.

*Quantitative findings*

*Effects of sensitisation campaigns on women having increased access and ownership to assets, credit and income*

Dunkle and colleagues' (2020) experimental study in Rwanda was the only study evaluating the impact of sensitisation campaigns on women having increased access and ownership to assets, credit and income. Their report included two effects measured at 12 and 24 months that fell into this outcome category (any earned income and any household debt payments). The effects ranged from very small, positive point estimates at 12 months (*g *=* *0.03, [95% CI: −0.08 to 0.13]) to small, positive effects at 24 months (*g *=* *0.17, [95% CI: 0.05 to 0.30]). We assessed the study as having high risk of bias.

*Effects of sensitisation campaigns on initiatives supported that facilitate women to access decent work (formal and informal employment), including people with disabilities*

Quisumbing et al.'s (2020) experimental study in Bangladesh was the only study evaluating the impact of sensitisation campaigns on initiatives supported that facilitate women to access decent work (formal and informal employment), including people with disabilities. Their report included six effects that fell into this outcome category for each of two treatment arms (nutrition and agricultural). The effects measured were, the number of hours spent on work, work balance and time spent working. The effects ranged from very small and not significant point estimates (*g *=* *0.00, [95% CI: −0.11 to 0.11]) when looking at first differences, to large, positive and statistically significant effects (*g *=* *0.58, [95% CI: 0.47 to 0.69]) when comparing means. We assessed the study as having some risk of bias concerns.

*Effects of sensitisation campaigns on women having more and better control over their bodies and sexual health*

Decker and colleagues' (2020) experimental study in Malawi was the only study evaluating the impact of sensitisation campaigns on women having more and better control over their bodies and sexual health. There was a very small, not statistically significant, point estimate (*g *=* *0.01, [95% CI: −0.21 to 0.23]), and we assessed the study as having some risk of bias concerns.

*Effects of sensitisation campaigns on women having increased freedom of movement and association*

Quisumbing et al.'s (2020) experimental study in Bangladesh was the only study evaluating the impact of sensitisation campaigns on women having increased freedom of movement and association. Their report included six effects that fell into this outcome category for each of the two treatment arms (e.g., visiting important locations). The effects ranged from large, negative effects (*g *=* *−0.67, [95% CI: −0.78 to −0.56]) to large, positive effects (*g *=* *2.32, [95% CI: 2.19 to 2.45]). We assessed the study as having some risk of bias concerns.

*Effects of sensitisation campaigns on women having more positive attitudes towards taking action to claim their rights*

**Figure 118:**
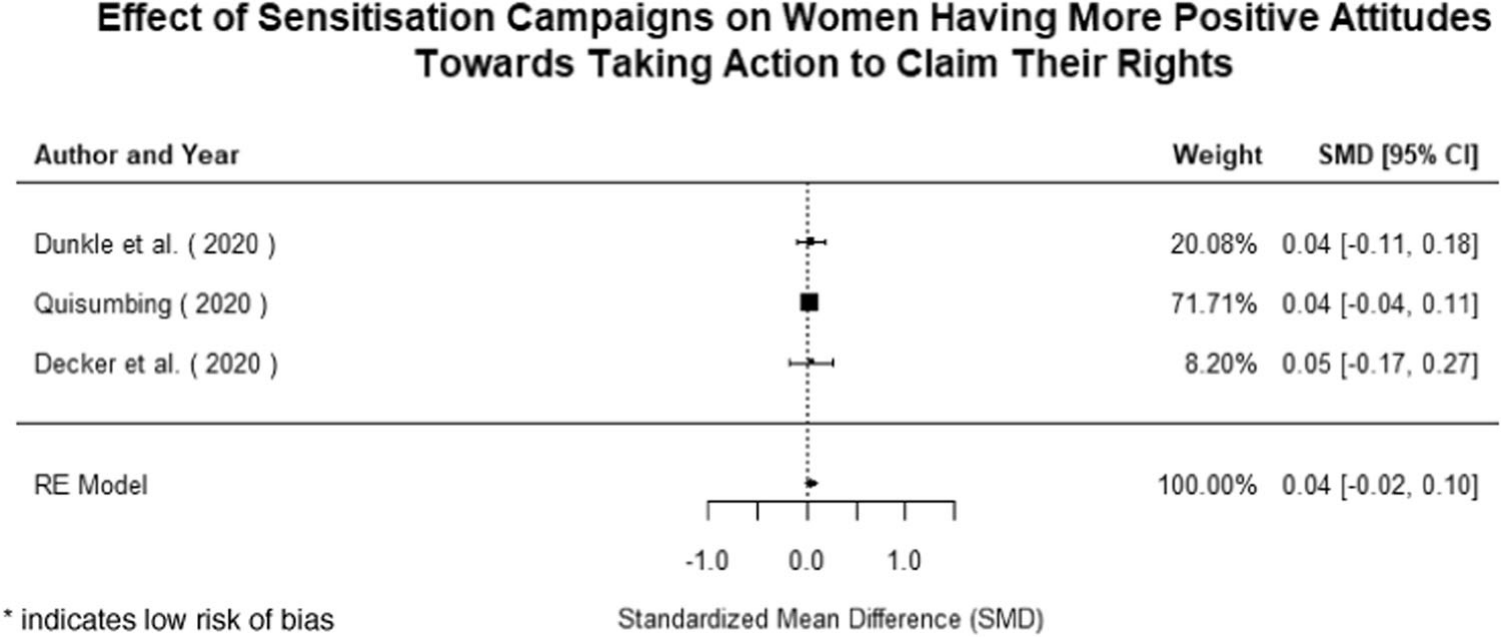
SCBA5: Forest plot showing the observed outcomes and the estimate of the random‐effects model. CI, confidence interval

We included a total of k=3 studies in the analysis. We assessed none of the studies as low risk of bias, two as some concerns, and one as high risk of bias. The observed outcomes ranged from 0.04 to 0.05. The estimated average outcome based on the random‐effects model was $\hat{\mu }=0.04$ (95% CI: −0.02 to 0.10). Therefore, the average outcome did not differ significantly from zero (z=1.19, p=0.23). A forest plot showing the observed outcomes and the estimate based on the random‐effects model is shown in Figure 118 (SCBA5).

According to the Q‐test, there was no significant amount of heterogeneity in the true outcomes (Q(2)=0.01, p=1.00, ${\hat{\tau }}^{2}=0.00$, $I^{2}=0.00$%). An examination of the studentised residuals revealed that none of the studies had a value larger than ±2.39 and hence there was no indication of outliers in the context of this model. According to the Cook's distances, none of the studies could be considered to be overly influential. With only three studies and no heterogeneity, moderator analyses were not appropriate.

*Effects of sensitisation campaigns on reducing the percentage of women agreeing with certain reasons that justify violence against women and girls*

**Figure 119:**
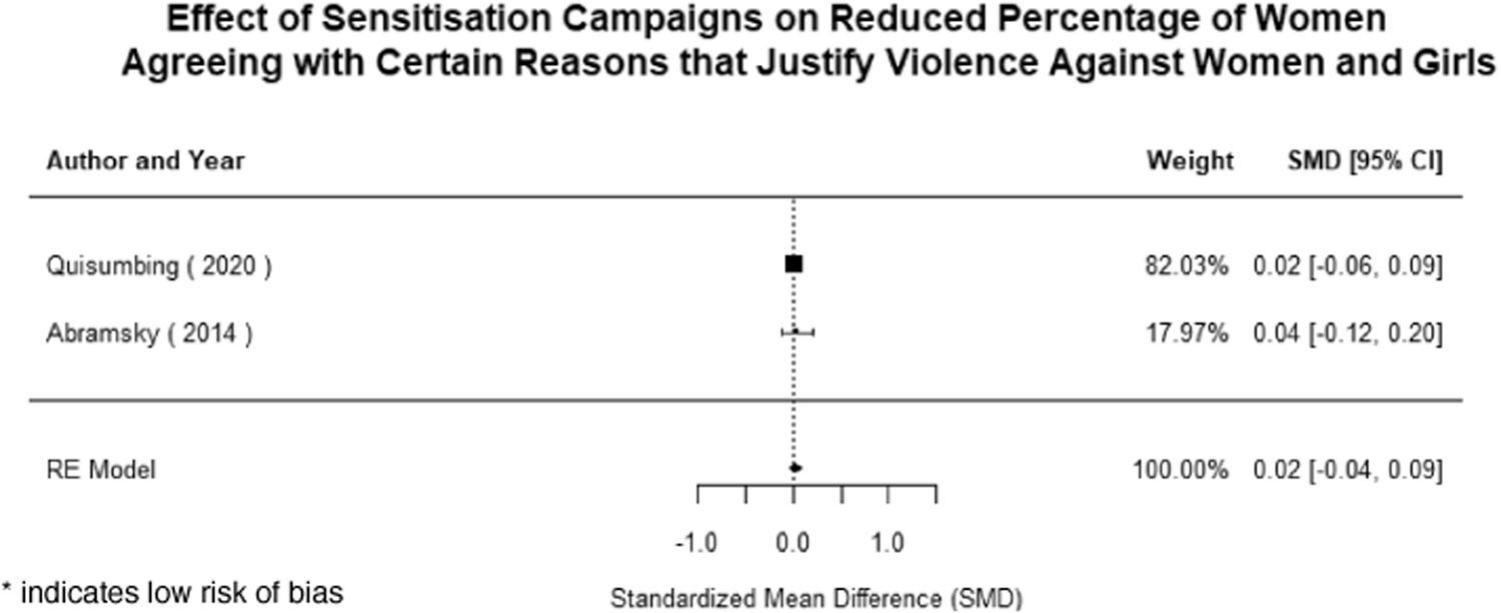
SCBA6: Forest plot showing the observed outcomes and the estimate of the random‐effects model. CI, confidence interval

Two studies examined the impacts of sensitisation campaigns on reducing the percentage of women agreeing with certain reasons that justify violence against women and girls, thus we included *k *=* *2 studies in the analysis. We assessed one of the studies as having some concerns of risk of bias and the other as high risk of bias. The estimated average outcome based on the random‐effects model was $\hat{\mu }=$ 0.02 (95% CI: −0.04 to 0.09). Therefore, the average outcome did not differ significantly from zero (*z *=* *0.66, *p *=* *0.51). A forest plot showing the observed outcomes and the estimate based on the random‐effects model is shown in Figure 119 (SCBA6). Given the small number of studies, this result should be interpreted with caution. According to the *Q*‐test, there was no significant amount of heterogeneity in the true outcomes (*Q* (1)* *=* *0.06, *p *=* *0.81, *τ* ^2^ **=* *0.00, *I* ^2^ **=* *0.00%). With only two studies, moderator analyses were not possible and tests of publication bias are not valid. None of the studies was appraised as having a low risk of bias.

*Effects of sensitisation campaigns on women being equipped with better life skills that allow them to be prepared for crisis or shocks and recover from them*

Decker and colleagues' (2020) experimental study in Malawi was the only study evaluating the impact of sensitisation campaigns on women being equipped with better life skills that allow them to be prepared for crisis or shocks and recover from them. There was a medium, but not statistically significant, point estimate (*g *=* *0.21, [95% CI: −0.01 to 0.43]), and we assessed the study as having some risk of bias concerns.

*Effects of sensitisation campaigns on increased participation in decision making by women at the household or community level*

Quisumbing et al.'s (2020) experimental study in Bangladesh was the only study evaluating the impact of sensitisation campaigns on increased participation in decision making by women at the household or community level, including during crisis response. For each of two treatment arms (nutrition and agricultural training), their report included 12 effects that fell into this outcome category (e.g., Access to and decisions on financial service). The effects ranged from very small, positive, but not statistically significant point estimates (*g *=* *0.01, [95% CI: −0.07 to 0.08]) to large, positive effects (*g *=* *17.70, [95% CI: 17.19 to 18.21]). We assessed the study as having some risk of bias concerns.

*Effects of sensitisation campaigns on supporting effective prevention strategies to end violence against women and girls*

**Figure 120:**
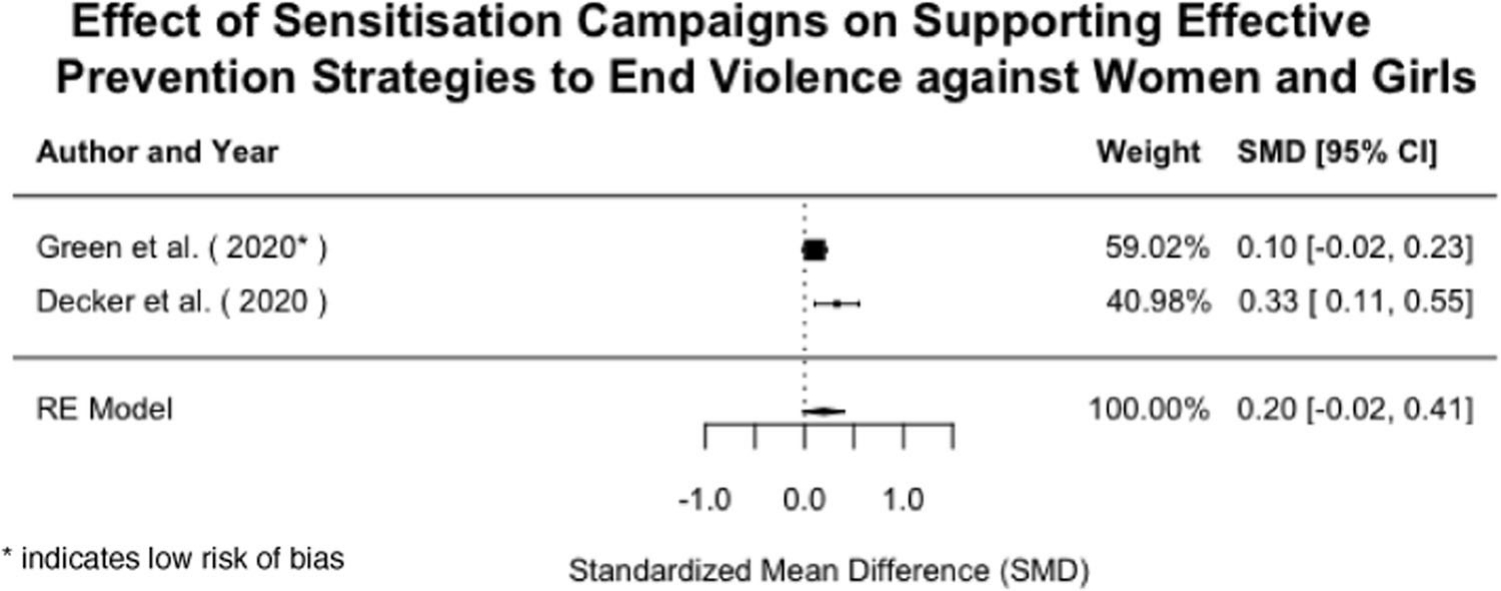
SCCA6: Forest plot showing the observed outcomes and the estimate of the random‐effects model. CI, confidence interval

Two studies examined the impacts of sensitisation campaigns on supporting effective prevention strategies to end violence against women and girls, thus we included *k *=* *2 studies in the analysis. We assessed one of the studies as low risk of bias and the other as some concerns. The estimated average outcome based on the random‐effects model was $\hat{\mu }=$0.1958 (95% CI: −0.0193 to 0.4109). Therefore, the average outcome did not differ significantly from zero (*z *=* *1.7845, *p *=* *0.0743). A forest plot showing the observed outcomes and the estimate based on the random‐effects model is shown in Figure 120 (SCCA6). Given the small number of studies, this result should be interpreted with caution. According to the *Q*‐test, there was no significant amount of heterogeneity in the true outcomes (*Q* (1) * *=* *2.19814, *p *=* *0.0842, *τ* ^2 = ^0.0165, *I* ^2^ **=* *66.4600%). With only two studies, moderator analyses were not possible and tests of publication bias are not valid. One of the studies was appraised as having a low risk of bias.

*Effects of effect of sensitisation campaigns on increased community awareness of the issues affecting women*

**Figure 121:**
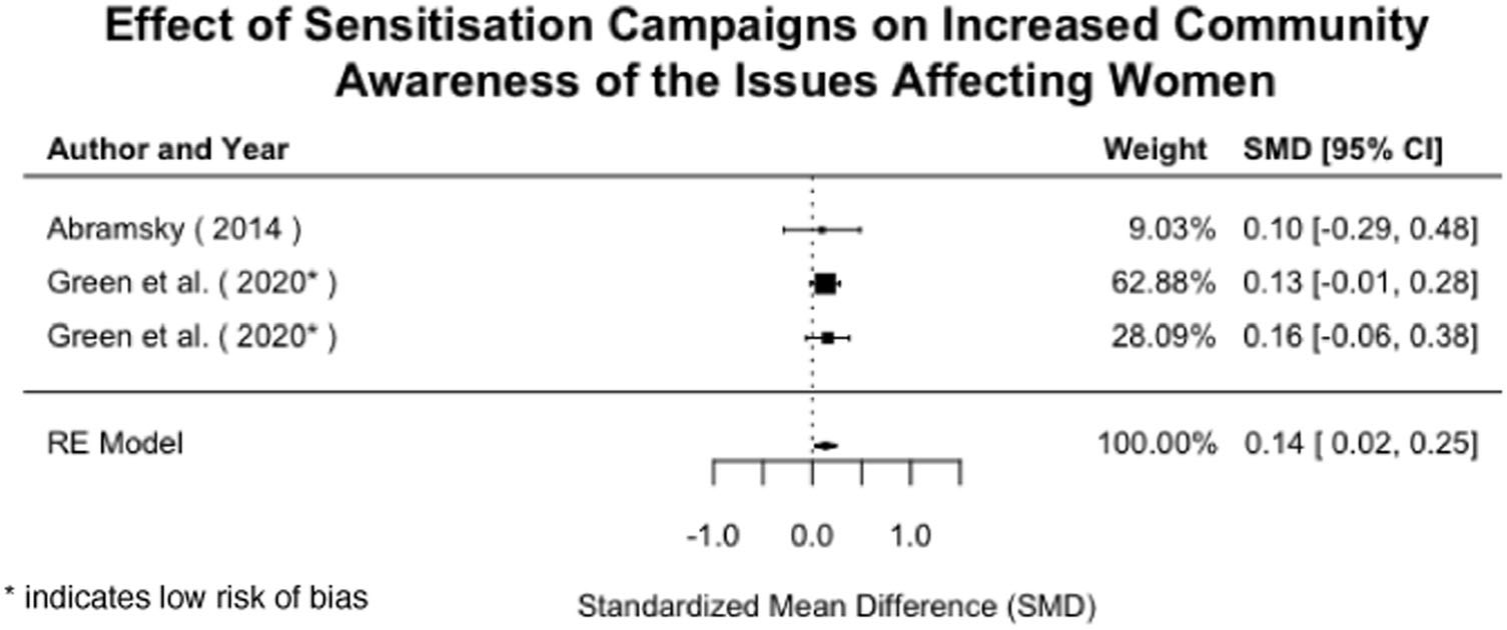
SCCB1: Forest plot showing the observed outcomes and the estimate of the random‐effects model. CI, confidence interval

We included a total of k=3 studies in the analysis. We assessed one of the studies (two of the effects) as low risk of bias and the other as high risk of bias. The observed outcomes ranged from 0.10 to 0.16. The estimated average outcome based on the random‐effects model was $\hat{\mu }=0.14$ (95% CI: 0.02 to 0.25). Therefore, the average outcome differed significantly from zero (z=2.32, p=0.02). Here we include two independent effects from Green and colleagues (2020), one for men (*g *=* *0.13) and one for women (*g *=* *0.16). A forest plot showing the observed outcomes and the estimate based on the random‐effects model is shown in Figure 121 (SCCB1). Two of the three studies were appraised as low risk of bias.

According to the Q‐test, there was no significant amount of heterogeneity in the true outcomes (Q(2)=0.08, p=0.96, ${\hat{\tau }}^{2}=0.00$, $I^{2}=0.00$%). An examination of the studentised residuals revealed that none of the studies had a value larger than ±2.39 and hence there was no indication of outliers in the context of this model. According to the Cook's distances, none of the studies could be overly influential. With only three effects and no heterogeneity, moderator analyses were not appropriate.

*Effects of sensitisation campaigns on women having improved self‐image and confidence*

**Figure 122:**
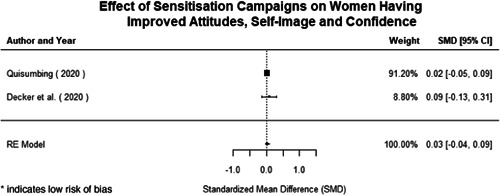
SCCB3: Forest plot showing the observed outcomes and the estimate of the random‐effects model. CI, confidence interval

Two studies examined the impacts of sensitisation campaigns on reducing the percentage of women agreeing with certain reasons that justify violence against women and girls, thus we included *k *=* *2 studies in the analysis. We assessed both of the studies as having some concerns of risk of bias. The estimated average outcome based on the random‐effects model was $\hat{\mu }=$ 0.03 (95% CI: −0.04 to 0.09). Therefore, the average outcome did not differ significantly from zero (*z *=* *0.86, *p *=* *0.39). A forest plot showing the observed outcomes and the estimate based on the random‐effects model is shown in Figure 122 (SCCB3). Given the small number of studies, this result should be interpreted with caution. According to the *Q*‐test, there was no significant amount of heterogeneity in the true outcomes (*Q* (1) * *=* *0.29, *p *=* *0.59, *τ* ^2^ **=* *0.00, *I* ^2^ **=* *0.00%). With only two studies, moderator analyses were not possible and tests of publication bias are not valid. Neither of the studies were appraised as having a low risk of bias.

*Effects of sensitisation campaigns on improved attitudes and increased support for women's economic, social and human rights*

**Figure 123:**
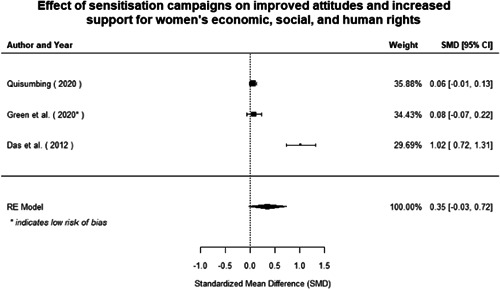
SCCB4: Forest plot showing the observed outcomes and the estimate of the random‐effects model. CI, confidence interval

We included a total of k=3 studies in the analysis. We assessed one of the studies as low risk of bias, one as some concerns, and one as high risk of bias. The observed outcomes ranged from 0.06 to 1.02. The estimated average outcome based on the random‐effects model was $\hat{\mu }=0.35$ (95% CI: −0.03 to 0.72). Therefore, the average outcome did not differ significantly from zero (z=1.83, p=0.07). A forest plot showing the observed outcomes and the estimate based on the random‐effects model is shown in Figure 123 (SCCB4).

According to the Q‐test, the true outcomes appear to be heterogeneous (Q(2)=39.03, p<0.01, ${\hat{\tau }}^{2}=0.10$, $I^{2}=94.88$%). An examination of the studentised residuals revealed that one study (Das et al., 2012) had a value larger than ±2.39 and may be a potential outlier in the context of this model. According to the Cook's distances, none of the studies could be considered to be overly influential. With only three studies, moderator analyses were not appropriate.

*Effects of effect of sensitisation campaigns on decreased violence/discrimination at the household level*

Dunkle and colleagues' (2020) experimental study in Rwanda was the only study evaluating the impact of sensitisation campaigns on decreased violence/discrimination at the household level. Their report included two effects that fell into this outcome category: children in household witnessing IPV at 12 and 24 months of exposure. For both waves the effect was medium, negative and significant (*g *=* *−0.22, [95% CI: −0.37 to −0.07]). However, we assessed the study as having high risk of bias.

*Effect of sensitisation campaigns on quality of relationships between women and their household and community members*

**Figure 124:**
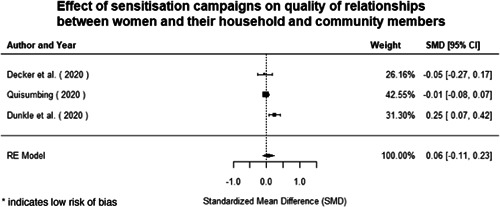
SCCB8: Forest plot showing the observed outcomes and the estimate of the random‐effects model. CI, confidence interval

We included a total of k=3 studies in the analysis. We assessed none of the studies as low risk of bias, two as some concerns, and one as high risk of bias. The observed outcomes ranged from −0.05 to 0.25. The estimated average outcome based on the random‐effects model was $\hat{\mu }=0.06$ (95% CI: −0.11 to 0.23). Therefore, the average outcome did not differ significantly from zero (z=0.70, p=0.49). A forest plot showing the observed outcomes and the estimate based on the random‐effects model is shown in Figure 124 (SCCB8). None of the studies were appraised as low risk of bias. Accordingly, these results should be applied cautiously.

According to the Q‐test, the true outcomes appear to be heterogeneous (Q(3)=8.51, p=0.04, ${\hat{\tau }}^{2}=0.01$, $I^{2}=64.74$%). An examination of the studentised residuals revealed that one study (Dunkle et al., 2020) had a value larger than ±2.50 and may be a potential outlier in the context of this model. Indeed, sensitivity analyses leaving each study out indicated that removing Dunkle and colleagues (2020) would reduce the overall average effect ($\hat{\mu }\;=\;$−0.01 (95% CI: −0.15 to 0.36), making the effect negative but still nonsignificant (*z *=* *0.83, *p *=* *0.40). According to the Cook's distances, none of the studies could be overly influential. With only three studies, moderator analyses were not appropriate.

*Effects of sensitisation campaigns on women's empowerment index*

**Figure 125:**
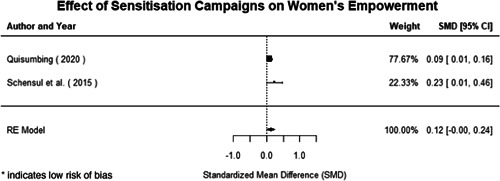
SCCC1: Forest plot showing the observed outcomes and the estimate of the random‐effects model. CI, confidence interval

Two studies examined the impacts of sensitisation campaigns on women's empowerment index, thus we included *k *=* *2 studies in the analysis. We assessed one of the studies as having some concerns of risk of bias and the other as high risk of bias. The estimated average outcome based on the random‐effects model was $\hat{\mu }=$ 0.12 (95% CI: −0.001 to 0.24). Therefore, the average outcome did not differ significantly from zero (*z *=* *1.94, *p *=* *0.05). A forest plot showing the observed outcomes and the estimate based on the random‐effects model is shown in Figure 125 (SCCC1). Given the small number of studies, this result should be interpreted with caution. According to the *Q*‐test, there was no significant amount of heterogeneity in the true outcomes (*Q* (1) * *=* *1.45, *p *=* *0.23, *τ* ^2^ **=* *0.003, *I* ^2^ **=* *31.23%). With only two studies, moderator analyses were not possible, and tests of publication bias are not valid.

*Qualitative findings*

We conducted a thematic synthesis on the 10 linked qualitative studies to the included sensitisation campaign interventions. As indicated above, this thematic synthesis aims to identify themes related to the interplay of intervention design, intervention implementation, target population, and contextual variables with intervention outcomes and effects. In total, we identified 17 descriptive themes, which we configured into five analytical themes (Supporting Information Appendix SN). These five analytical themes present the synthesis results and are discussed in more detail below.

*Theme 1: Patriarchal attitudes and gendered norms hinder sensitisation campaigns whilst religious messages can facilitate the process of attaining gender equality sensitisation*.

Constructive religious messages and principles can encourage sensitisation and changing attitudes towards gender equality. More specifically, while rigid gender norms and attitudes act as a potential barrier to gender sensitisation, religious messages can act as a useful channel. Due to gendered norms, women tend to refrain from allowing their husbands to participate in domestic work. Further, women may not be comfortable discussing intimate issues in public and confiding in others. Based on findings from the qualitative evidence base, men who were more actively and visibly involved in domestic work were considered outliers. Men may be reluctant to loosen control over their wives and allow them to work outside the home (Mogford et al., 2015).

Traditional family structures and rigid patriarchal attitudes were reported to curtail sensitisation across communities. For instance, Men's Action for Stopping Violence Against Women (MASVAW) respondents in India reported the difficulty of bypassing existing norms and roles in the context of extended families (Mogford et al., 2015). In this way, ‘…when a MASVAW man living in an extended family’ confronted the status quo, he was perceived as being an opponent of family members' lifestyles. Gender norms made it difficult for women to adjust to the sensitisation of men into roles that were traditionally reserved for women. In fact, some spouses were not comfortable with the increased involvement of their husbands in household chores due to the family pressure they faced as a result of allowing this (Mogford et al., 2015).

Curriculums designed to help adjust power dynamics in relationships, identify the causes of IPV and improve knowledge of the rights of women seemed to be successful in discouraging IPV and enhancing sensitisation to the IPV problem in a Rwandan context (Stern & Nyibizi, 2018). The Indashyikirwa Intimate Partner Violence Prevention Programme (Indashyikirwa) was observed to aid men's understanding of the perpetration of sexual IPV and sensitised women to their own rights to refusal. One Indashyikirwa man stated: ‘I thought, my wife is mine, I married her; so, there is nothing which can prevent me from having sexual intercourse with her. But then, after receiving the lesson on sexual intercourse, we found out that she can sometimes be feeling unwell when you force her to have sexual intercourse, in that case you are committing violence’. Women enrolled in the programme also reported improved spousal relations and knowledge owing to better understanding their own rights to sexual consent (Stern, Heise, & Cislaghi, 2020). Indashyikirwa couples described better‐quality relationships due to a better understanding of IPV as they adjusted their behaviour and focus on their caregiving responsibilities within the relationship (Stern & Nyiratunga, 2017).

The role of reflection through religious messages and principles supported participants to understand and internalise that the genders were all equal. Essentially, religion acted as a conduit for participants to better understand how power imbalances between genders could be improved. One religious leader expressed: ‘The Indashyikirwa training has been very useful to us even though it is hard in the beginning because there are some who didn't understand. There has been a challenge of mentality but because we are used to learning the word of God, we know everyone should have the equal right regardless of their gender. That has been very helpful to us’ (Stern, Heise, & Cislaghi, 2020). The training component of Indashyikirwa was a valuable tool because it provided couples with a participatory environment and problem‐solving skills platform.

*Theme 2: Culture of openness and community engagement supports sensitisation to gender issues*.

Open dialogue forums and communication between and within communities can aid the resolution of gender‐related conflict as people collaborate to understand gender issues and reduce IPV. Third parties such as other community members realise that there are greater incentives to intervene during conflict as it may help build respect and trust in relationships. Women are additionally encouraged to discuss their experiences and sensitise their communities to violence against women and other gender‐related issues.

By using participatory training and community‐based activism, the Indashyikirwa programme encouraged a culture of community engagement to activate learning and third‐party intervention. In this way, participants supported those that suffered from IPV. One community leader expressed that: ‘We studied laws protecting women. That has made us more confident because if I see a victim of violence, it would not cause me any problems to look for a leader and tell him there is a person who is a victim of violence there. After receiving that training, I felt I also have to play a role’. An opinion leader offered that: ‘We used to face cases of violence in our work but ignored how to classify the types of violence. I and my colleagues who had the training, we came to understand this…. we immediately understand what it is about and how to solve it quickly’ (Stern, Heise, Dunkle, et al., 2020). While Indashyikirwa respondents from Chatterji and colleagues (2020) reported that more intimate settings were conducive to thorough engagement, others from Stern, Heise, Dunkle and colleagues (2020) voiced greater confidence in raising awareness and openly discussing IPV: ‘Now I openly speak out and I use the power that I have in me and I feel there is something that I can do to make my family developed. That is a very big thing’. The Indashyikirwa also found value in using community forums such as parent's evenings to sensitise communities on the negative consequences of perpetrating IPV (Stern & Niyibizi, 2018). Ultimately, community‐wide coordination and planning may have ensured that implementation was relatively easy as one activist noted: ‘When I prepare a discussion, I ask for help from the village leader. The village leader tells the community members to come for a meeting; comes to start a meeting for you, then you continue with the discussion and he closes for you. These are thanks to the quarterly meeting you had together, about taking measures and solutions’.

SASA! in Uganda used community mobilisation to mitigate the scourge of IPV and t develop quality of relationships between couples. The engagement of couples by SASA! staff equipped them with problem‐solving skills that encouraged greater mutual respect and more open communication channels (Starmann et al., 2017). By helping participants understand the main tenets of a healthy relationship through community engagement, some couples reported positive responses to the intervention as two men observed in their relationships respectively: ‘[W]e started smiling, we started talking and discussing issues well together’, and ‘What I am most happy about is the agreeing and understanding each other…it shows love in the relationship’. Engagement with SASA! staff seemed to work well to support greater consideration of equality and choice between partners, openness around sensitive issues and shifts in gender norms and roles. Regarding household finances, a SASA! man in Uganda observed: ‘I don't hide anything from her … it was SASA! that taught us all that… They tell us that it is good for each one of you to inform the other about your income. You have to let them know how much you earn and together you decide how to use that money. You discuss what to buy, how much to save and you don't use another's money without their consent because you have power. You have to agree and if one of the parties does not want [to] then you don't force her’ (Kyegombe et al., 2014).

*Theme 3: Awareness, conflict resolution and communication skills training contribute to greater group sensitisation and awareness to IPV*.

Trainings to build awareness of IPV and conflict resolution communication skills are observed to be important in the sensitisation of men and boys given the lack of understanding of various forms of IPV among men. Training presents an opportunity for men to challenge traditional gender boundaries. Partner involvement in this training enhances openness about personal experience and combined decision‐making, particularly regarding finances.

Post‐intervention, many participants of the Indashyikirwa programme expressed greater awareness in understanding IPV in its various forms after being sensitised to the violation behind it (Stern & Niyibizi, 2018). Men were sensitised to the perverse nature of the act of violence on both women and children; as one woman remarked on the behaviour of her partner: ‘He has many regrets and said I wish I could get back the time that I wasted and said this will not happen again. Sometimes the child even tells him, ‘do you remember the way you used to beat me?’ And my husband tells him, ‘I promise I will not beat you again’ (Stern, Heise, & Cislaghi, 2020; Stern, Heise, Dunkle, et al., 2020). Analogous to this were the qualitative findings that BCC influenced their conflict resolution skills. The curriculum provided in the Indashyikirwa allowed participants to self‐reflect and discover the underlying causes for IPV as one man in the programme recounted: ‘After drinking I felt, I am a man and I would commit violence against my wife but after having studied about alcohol, I realised it prevents me from reaching any household development. Now I am no longer a drunkard. I am now a person who builds his family and my family has noticed that I have changed. We became Indashyikirwa’.

Training men to understand the value of women's roles better was an important exercise for MASVAW participants (Mogford et al., 2015). In particular, allowing men to imagine ‘the average day in a woman's life’ was instrumental in helping to provoke reflection around gender roles and norms. In the SASA! programme, a former perpetrator of physical IPV reflected that: ‘They told me, ‘what you are doing is not good. You will kill your wife, and after killing her, you won't find another wife like her’. So, I understood that it is a shame and until now it has never happened again’. Essentially, the qualitative evidence proposed that equipping men with conflict resolution skills was helpful in addressing attitudes to IPV (Stern & Niyibizi, 2018). Additionally, financial skills were reported to be a useful addition to the training as it allowed better communication between couples regarding finances. As one man pointed out: ‘I used to feel I had to be the one to say how to spend money and that my wife didn't have a say because she had not worked for that money. But after having seen that when I go to work my wife does other housework, we put together what we have both done, we put the money at the bank or at the VSLAs since we are members of the group. We learned to save and how to spend the rest of the money. That has helped us’ (Stern & Nyiratunga, 2017). In this way, sensitising couples to find the source of spousal frictions was used to smooothen the quality of their relationships.

*Theme 4: Greater contact frequency and programme length aids a positive shift in perceptions about gender norms and equality while regular and sustained interaction are key requirements for enhanced programme implementation*.

Greater time exposure to programme activity can improve programme effectiveness by allowing participants to adjust their beliefs and adopt better social norms over time. But sensitisation programme donors need to consider that such intervention types can be costly and protracted when undertaken. The qualitative evidence suggests that programme outcomes are more favourable when dissemination delays are minimised, targeting rigour is ensured, and activity sequencing is carefully planned. To maximise sensitisation campaigns' influence, consistent interaction and discussions with implementation staff and peers is a required design feature. Joint engagement by relevant stakeholders and concerted activism enhanced implementation. In particular, sensitisation campaigns designed to aid and improve interpersonal relationships and change couple processes were observed to be of promise. The role of colleagues or wives in providing inspiration and motivation is crucial for sensitisation campaigns aimed at transforming male attitudes and behaviours. Moreover, beneficiaries report notable reduction in anxiety levels and greater motivation to participate under such conditions.

The longer and more frequent the programme activities, the better received the intervention was by participants as this can allow enough time to learn about and apply new skills. Qualitative responses from SASA! participants in Rwanda cited the length and frequency of exposure to intervention activities on couples as key factors for participant follow through (Starmann et al., 2017). Responses from SASA! implementation staff and CAs suggested that programme impact was likely curtailed by insufficient time for in‐depth activism across communities (Chatterji et al., 2020). Similarly, Indashyikirwa programme partners indicated that the activism component of this intervention was fairly truncated and thus insufficient to convincingly alter gender norms that are embedded within society. Furthermore, from a funding perspective, the duration of an intervention designed to shift societal norms has to be carefully considered to effect change (Chatterji et al., 2020).

Effective programme implementation relies heavily on sustained interaction with programme staff and designed material. The formation of sustainable relationships with CAs in the SASA! programme reportedly contributed to positive changes in personal and couple relationships (Abramsky et al., 2014). SASA! participants observed that the continued guidance and support of CAs empowered couples to broaden communication avenues for quarrelsome topics to be dealt with jointly. The proximity, availability and consistency of CAs allowed SASA! participants to trust and use their advice on the resolution of relationship conflict. In the case of Indashyikirwa, the cursory use of CAs hinderd their contribution as their ability to mobilise the community was curtailed by hasty implementation and poor readiness. An additional constraint in this case was the tailoring of interventions to successfully embed them in an Indian setting (Chatterji et al., 2020).

The regularity of interaction with programme staff is reported to gradually sensitise programme participants. The frequency of activities by SASA! participants was highlighted by various respondent couples as a motivating factor to continue with adherence and participation (Starmann et al., 2017). One woman responded: ‘[T]hey warned him, I think they all scared him…he realised that he had to change’ when asked about how regular interaction with CAs had enhanced her relationship with her partner. It was noteworthy that the benefits of steady interaction were realised by couples even when only one partner adhered to this aspect of the SASA! intervention. Moreover, allowing both partners to interact with CAs was repoted to yield greater success as couples were able to build healthier relationships with constant support from programme staff.

*Theme 5: Community leaders including men and opinion leaders are key actors in developing and using sensitisation strategies*.

Opinion leaders and men are key actors in the success of sensitisation programmes in communities. When opinion leaders realise their influence over societal outcomes and citizen behaviours, sensitisation programmes can help in focusing their efforts in a more responsible and positively influential manner geared towards gender equality. Sensitisation programmes offer men the opportunity to learn and self‐correct negative behaviours that are grounded in beliefs around masculinity. Personal histories and relationships can be a barrier in improving gender equality, but concerted sensitisation efforts can improve this.

To achieve greater sensitisation, community leaders and target groups are frequently appointed as agents of change to lead programme activities and positively influence other participants. As a field member of the implementation staff in the Indashyikirwa programme in Rwanda observed: ‘Several opinion leaders shared testimonies of change in their relationships by the end line interviews, including men more actively supporting domestic and caregiving roles, more equitable decision‐making, and improved conflict resolution’ (Stern, Heise, Dunkle, et al., 2020). The qualitative findings indicated that opinion leaders were excited to engage actively with other participants and support programme staff in identifying and embedding knowledge of gender inequalities in their communities. Their involvement encouraged them to prioritise IPV prevention and wider engagement on gender issues with the community. Similarly, the involvement of men helped sensitisation campaigns gain traction in their localities as they were equipped with the knowledge to be champions for change on gender and equity issues (Mogford et al., 2015).

Participants of the MASVAW intervention in India communicated that they were empowered to assess their own complicity in the perpetuation of gender stereotypes and inequalities in their own societies. Some of the reportedprominent behavioural changes amongst these men were greater acceptance of women's physical autonomy, more informed views on sexual intercourse with their partners and their domestic involvement in activities that were traditionally set aside as being strictly for women. Furthermore, all participants in the MASVAW programme the reported greater satisfaction in their marriages through greater knowledge of gender issues.

A useful dynamic in the MASVAW model of interaction was selecting men to engage with other men (Mogford et al., 2015). Participants viewed this as successful because in addition to broadening their understanding of gender equality and empathy with women, they could reflect on the role of their own masculinity in peddling misinformation and unyielding attitudes to women. Moreover, opinion leaders and men were seen as successful agents of change in the sensitisation of their communities. As another opinion leader selected in the Indashyikirwa programme expressed: ‘A leader must be a role model in his own family, a role model in his extended family, a role model where you live, where you work because you cannot be a bad example while you are a leader. After being trained, I gained a lot; starting from my family, even if we did not have conflicts, but it helps me be humble, and to spread a good message’.

##### 5.4.2.4 Discussion

Quantitative meta‐analysis showed positive results for sensitisation campaigns across a number of outcomes. While there are cases of statistical insignificance at the 95% confidence level (all outcomes except community awareness), various insights from the qualitative findings can supplement the quantitative results. Positive quantitative effects from sensitisation campaigns cover outcomes such as: (1) women having more positive attitudes towards taking action to claim their rights (*n* = 3), (2) reducing the percentage of women agreeing with certain reasons that justify violence against women and girls (*n* = 2), (3) supporting effective prevention strategies to end violence against women and girls (*n* = 2), (4) women having improved self‐image and confidence (*n* = 2), (5) improved attitudes and increased support for women's economic, social and human rights (*n* = 3), (6) increased community awareness of the issues affecting women (*n* = 3), (7) reduced frequency and distribution of types of violence by an intimate partner (*n* = 5), (8) quality of relationships between women and their household and community members (*n* = 4), (9) women's empowerment index (*n* = 2). Some qualitative findings that can explain these findings are as follows:

- Based on the qualitative findings, sensitisation campaigns can be limited and restricted by social factors such as gender norms and roles. Changes that are related to altering human behaviour through learning and communication commonly can take time to realise.
- Culturally developed patriarchal attitudes can stem the effectiveness of sensitisation campaigns because they are more difficult to change.
- To enhance effectiveness, sensitisation campaigns can adopt strategies that expose participants to longer and more frequent programme activities such as training and counselling. More concerted efforts to sensitise communities on a larger scale can lead to more empathetic interventions by community members and support staff in spousal conflicts.
- Hurried and disorganised implementation of sensitisation processes can hinder programme participation by communities.
- Issues of personal image, knowledge of rights and empowerment can be addressed gradually as they depend on the nature of close relationships and personal confidence from successful programme impact.
- Opinion leaders can be used as agents of change to teach about IPV and instil useful skills for couples to rely on.
- Men who are respected community leaders can act as mentors for IPV perpetrators and help to facilitate the gradual breakdown of societal norms.

*Is there a more effective form of media for sensitisation campaigns?*

While the qualitative evidence base assessed does not provide a clear recommendation on the most reliable medium for the dissemination sensitisation campaigns, there are some notable observations:

- Media used for sensitisation campaigns are largely in‐person platforms and intimate sessions with a large focus on couples and men as perpetrators.
- Respected leaders in the community are used to counsel and sensitise couples to IPV, teach on the tenets of healthy relationships, impart conflict resolution skills and knowledge and mediate on conflicts within relationships.
- Using communal and public platforms is feasible to reach more participants at the same time while intimate settings with trusted leaders can help to more actively address obstacles to healthy personal relationships.
- A medium that is easily accessible and conscious to socioeconomic conditions related to access (to phones, places, etc.) can lead to greater participation.

##### 5.4.2.5 Summary of findings and discussion

We included seven studies in five countries in South Asia and Sub‐Saharan Africa that evaluated the effect of sensitisation campaigns. The largest positive and significant impact in the category of sensitisation campaigns was on women having more positive attitudes towards taking action to claim their rights, though the magnitude of the effect was still small. There was a similar small positive and significant impact on increased awareness in communities of the issues affecting women. There were also many outcomes for which there were positive but nonsignificant summary effects. These included reduced percentage of women agreeing with reasons that justify violence against women and girls, increased support for prevention strategies to end violence, women having improved attitudes, self‐image and confidence, improved attitudes and increased support for women's economic, social and human rights by men/household/community members, improved quality of relationships between women and their household/community members, and women's empowerment. In addition, there were two negative but nonsignificant effects for reduced frequency of both physical and sexual IPV. Overall, the GRADE assessments generally indicate a range of very low to moderate certainty in this body of evidence.

The ten linked qualitative studies and their resulting five analytical themes also offered a key look into what features of sensitisation campaigns can aid or hinder outcomes. The engagement of community leaders is a promising facilitator of sensitisation and engagement by the community. However, entrenched patriarchal norms will have to result in increased buy‐in by these stakeholders for appropriate uptake at the community level. Open dialogue forums may support this. Similarly, this aspect combined with regular and sustained interaction can also enhance the potential of programmes to achieve their intended outcomes. Overall, rich and in‐depth qualitative data can sustain the claims made by the meta‐analysis by pointing to recurring as well as contextual themes that have occurred across interventions. Table 45 presents the GRADE review of our findings (Table 40).

**Table 40:** GRADE summary of findings and certainty of evidence on sensitisation campaigns

Certainty assessment							Sample size	Effect	Certainty	Importance
No. of studies	Study design	Risk of bias	Inconsistency	Indirectness	Imprecision	Other considerations		Absolute (95% CI)		
*(AB2) Women have increased access and ownership to assets, credit and income*										
1	RCT	Very serious^a^	Serious^b^	Not serious	Not serious	None	1417	Four positive effect sizes with a 95% CI range of −0.08 to 0.30	⊕◯◯◯ VERY LOW	Important, but not critical
*(AC2) Initiatives supported that facilitate women to access decent work (formal and informal employment), including people with disabilities*										
1	RCT	Serious^c^	Serious^b^	Not serious	Serious^d^	None	3245	Two zero and six positive effect sizes, all near zero with one significant outlier	⊕◯◯◯ VERY LOW	Important, but not critical
*(BA2) Women have more and better control over their bodies and sexual health*										
1	RCT	Not serious	Serious^b^	Not serious	Not serious	None	312	SMD 0.01 SD higher (0.21 lower to 0.23 higher)	⊕⊕⊕◯ MODERATE	Critical
*(BA3) Women have increased freedom of movement and association*										
1	RCT	Serious^e^	Serious^b^	Not serious	Very serious^f^	None	2739	Two negative, two zero, and seven positive effect sizes with a 95% CI range of −0.78 to 2.45	⊕◯◯◯ VERY LOW	Limited importance
*(BA5) Women have more positive attitude towards taking action to claim their rights*										
3	RCT‐3	Serious^g^	Not serious	Not serious	Not serious	None	3817	SMD 0.18 SD higher (0.06 lower to 0.30 higher)	⊕⊕⊕◯ MODERATE	Limited importance
*(BA6) Reduced percentage of women agreeing with certain reasons that justify violence against women and girls*										
2	RCT‐2	Serious^h^	Serious	Not serious	Not serious	None	3338	SMD 0.02SD higher (0.04 lower to 0.09 higher)	⊕⊕◯◯ LOW	Critical
*(BA7) Women are equipped with better life skills that allow them to be prepared for crisis or shocks and recover from them*										
1	RCT	Not serious	Serious^b^	Not serious^i^	Serious^i^	None	312	SMD 0.21 SD higher (0.01 lower to 0.44 higher)	⊕⊕◯◯ LOW	Important, but not critical
*(BB1) Increased participation in decision making by Women at the household or community level, including during crisis response*										
1	RCT	Serious^j^	Serious^b^	Not serious	Very serious^k^	None	2739	29 positive effect estimates with widely varying CIs	⊕◯◯◯ VERY LOW	Limited importance
*(CA6) Effective prevention strategies supported to end violence against women and girls*										
2	RCT‐2	Not serious	Serious^l^	Not serious	Serious^m^	None	1348	SMD 0.2 SD higher (0.02 lower to 0.41 higher)	⊕⊕◯◯ LOW	Critical
*(CB1) Increased awareness in communities of the issues affecting women*										
2	RCT‐2	Serious^n^	Not serious	Not serious	Not serious	None	1143	SMD 0.14 SD higher (0.02 higher to 0.25 higher)	⊕⊕⊕◯ MODERATE	Limited importance
*(CB3) Women have improved attitudes, self‐image and confidence*										
2	RCT‐2	Not serious	Serious^o^	Not serious	Not serious	None	3557	SMD 0.03 SD higher (0.04 lower to 0.09 higher)	⊕⊕⊕◯ MODERATE	Limited importance
*(CB4) Improved attitudes and increased support for women's economic, social and human rights by men, household and family members and community members*										
3	RCT‐2 QED‐1	Serious^p^	Very serious^q^	Not serious	Serious^r^	none	4154	SMD 0.35 SD higher (0.03 lower to 0.72 higher)	⊕◯◯◯ VERY LOW	Limited importance
*(CB5) Decreased violence/discrimination at the household level*										
1	RCT	Serious^s^	Serious^b^	Not serious	Not serious	None	674	Two negative effect estimates with a 95% CI range of −0.37 to −0.07	⊕⊕◯◯ LOW	Critical
*(CB7) Reduced frequency and distribution of types of violence by an intimate partner—physical violence (PV)*										
4	RCT‐4	Very serious^t^	Serious^u^	Not serious	Serious^v^	None	4018	SMD 0.06 SD lower (0.18 lower to 0.06 higher)	⊕◯◯◯ VERY LOW	Critical
(CB7) Reduced frequency and distribution of types of violence by an intimate partner—sexual violence (SV)										
3	RCT‐3	Very serious^a^	Serious^b^	Not serious	Serious^v^	None	1380	SMD 0.01 SD lower (0.07 lower to 0.13 higher)	⊕◯◯◯ VERY LOW	Critical
*(CB8) Improved of relationships between women and their household and community members*										
3	RCT‐3	Serious^w^	Serious^x^	Not serious	Serious^y^	None	3555	SMD 0.06 SD higher (0.011 lower to 0.23 higher)	⊕◯◯◯ VERY LOW	Limited importance
*(CC1) Empowerment/Equality Index*										
2	RCT‐1 QED‐1	Very serious^z^	Not serious	Not serious	Not serious	None	3035	SMD 0.12 SD higher (0 to 0.24 higher)	⊕⊕◯◯ LOW	Important, but not critical

Abbreviations: CI, confidence interval; GRADE, Grading of Recommendations, Assessment, Development and Evaluations; QED, quasi‐experimental design; RCT, randomised controlled trial; RoB, risk of bias; SMD, standardised mean difference; TVET, technical and vocational education and training.

^a^ Downgraded because of a high overall risk of bias. Selection bias is likely. Outcome measurement bias unclear.

^b^ All single studies downgraded once for inconsistency.

^c^ Downgraded once because of a medium overall risk of bias. Deviation from intended intervention and outcome measurement bias are assessed as unclear.

^d^ For seven out of eight effect sizes there is large overlap of CIs and low variance of point estimates, centered around the median of −0.07. There is however one outlier with an estimate of (−)0.58 and a confidence interval not overlap with any of the seven studies.

^e^ Downgraded once because of a medium overall risk of bias. Deviation from intended intervention and outcome measurement bias are assessed as unclear.

^f^ Very wide variance of point estimates across the twelve effect sizes at both sides of the 0.00 threshold, ranging from −0.67 to 2.3. (b) Very limited overlap of CIs.

^g^ Downgraded because one study presents high risk of bias and the others are unclear. There is risk across all three studies of deviation from intended intervention.

^h^ Downgraded because half of the evidence comes from a high risk of bias study with issues relating to reporting bias and deviations from intended outcomes.

^i^ The 95% confidence interval for the only effect size spans from no effect (−0.01) to large effect (0.43).

^j^ Based on only one study assessed as having a medium overall risk of bias. Deviation from intended intervention and outcome measurement bias are assessed as unclear.

^k^ Very wide variance of point estimates across the 27 effect sizes, ranging from 0 to 17. (b) Very limited overlap of CIs.

^l^ Downgraded to reflect large CIs and one study's point estimate not falling within the CI of the other.

^m^ Downgraded because of the RE model's large CI's which cross both sides of the threshold.

^n^ Downgraded once to reflect that one‐third of the included studies presents high risk of bias due to outcome measurement and deviation from intended intervention.

^o^ Both studies' estimates are near to the threshold, but a wide CI in the Decker study, taken in cognizance of there only being two total studies, warrants a downgrade.

^p^ Downgraded because this groups has a high risk of bias QED whose point estimate varies hugely from the other two (lower risk of bias) RCTs.

^q^ (a) Wide variance of point estimates and no overlap of CI between the one QED study, and the other two RCTs. (b) The *Q*‐test for heterogeneity which tests the null hypothesis that all studies have the same underlying magnitude of effect, has a very low *p*‐value (*p *<* *0.01), significant at a 99% confidence level, which may indicate a problem with heterogeneity. $I^{2}$ indicates that 95% of the variability in effect estimates may be due to heterogeneity rather than sampling error (chance).

^r^ (a) Rate down if confidence intervals of RE Model cross the threshold: Yes. (B) Rate down if one study: No, three studies. (C) Sample size: Large, not rated down.

^s^ Based on only one study assessed as having a high overall risk of bias. Selection bias is likely. Outcome measurement bias unclear.

^t^ Out of four studies: two have high RoB, two have some concerns related to risk of bias. RoB assessments indicate that three out of four studies have been rated as (probably) not free from outcome measurement bias, and two have been marked as likely to have deviations from intended interventions.

^u^ (a) Relatively wide variance of point estimates across studies. (b) Minimal overlap of CIs, which suggests variation may be more than what one would expect by chance alone. (c) The *Q*‐test for heterogeneity which tests the null hypothesis that all studies have the same underlying magnitude of effect, has a relatively low *p*‐value (*p *<* *0.06), significant at a 90% confidence level, which may indicate a problem with heterogeneity. $I^{2}$ indicates that 59% of the variability in effect estimates may be due to heterogeneity rather than sampling error (chance).

^v^ (a) Rate down if confidence intervals of RE Model cross the threshold: Y. (B) Rate down if one study: No four studies. (C) Sample size: large, not rated down.

^w^ Out of three studies: one has a high RoB, two have some concerns related to risk of bias. For one study selection bias has been identified as a likely risk. Outcome measurement bias has been marked as unclear for two studies, while deviations from intended interventions are marked as unclear for one. I consider this as crucial limitation for one criterion and some limitations more criteria sufficient to lower confidence in the estimate of effect—by one level.

^x^ (a) Relatively wide variance of point estimates across studies. (b) Minimal overlap of CIs, which suggests variation may be more than what one would expect by chance alone. (c) The *Q*‐test for heterogeneity which tests the null hypothesis that all studies have the same underlying magnitude of effect, has a relatively low *p*‐value (*p *<* *0.04), significant at a 95% confidence level, which may indicate a problem with heterogeneity. $I^{2}$ indicates that 65% of the variability in effect estimates may be due to heterogeneity rather than sampling error (chance).

^y^ (a) Rate down if confidence intervals of RE Model cross the threshold: Yes. (B) Rate down if one study: No, three studies. (C) Sample size: >400, so not rated down.

^z^ Downgraded twice because both studies present some risk of bias, and one is a high risk of bias QED. When that study is removed, the point estimate of the RE model changes significantly.

### 5.5 Effects of interventions under the UNSCR prevention pillar

The prevention pillar is a set of interventions that build capacities and systems to support the gender responsiveness and inclusivity of violence prevention and conflict transformation processes. This also includes efforts to hold perpetrators of violence accountable through formal or informal means.

In our SR, this pillar bundled the following type of interventions covering 3872 beneficiaries:

- Community dialogues and reconciliations

This section provides the findings of our synthesis of the two included studies evaluating the effect of these interventions on gender equality, women's empowerment and peaceful and inclusive societies. The section is organised by each intervention group. Each sub‐section starts with a description of the inStervention groups, their activities and ToC, followed by descriptive results and the findings addressing our research questions.

**Figure 126:**
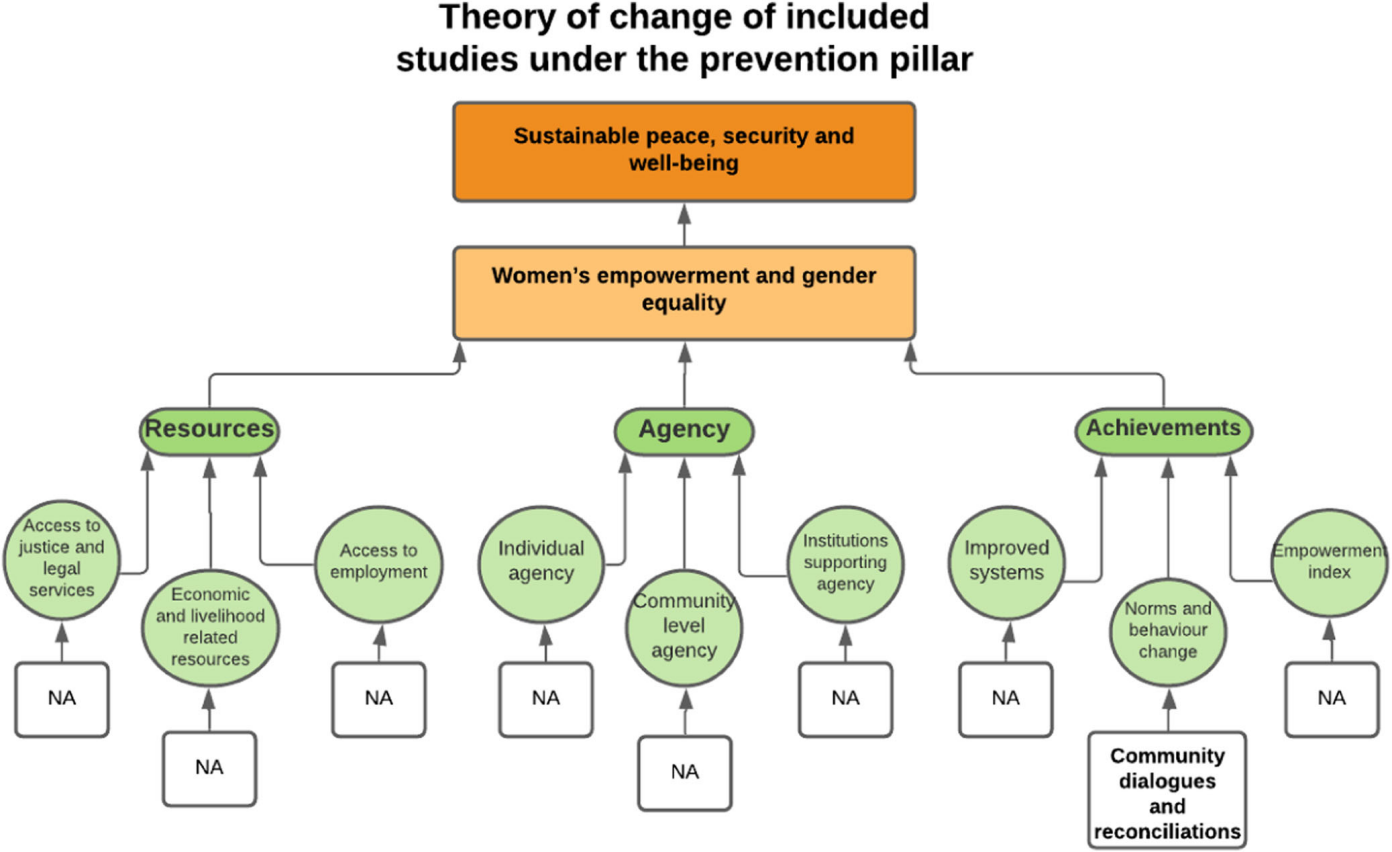
Theory of change of the included studies under the prevention pillar

Figure 126 provides a summary of the outcomes and targeted effects of the interventions included under the prevention pillar:

#### 5.5.1 Community dialogues and reconciliations

Community dialogues and reconciliations draw participants from different sections of a community and create opportunities for exchanging information and perspectives, clarifying viewpoints, and developing solutions to issues of interest to the community (Search for Common Ground, 2016). They differentiate from discussion groups because they can involve groups with conflicting interests while discussion groups involve members with shared interests. They often, but not exclusively, occur in conflict/post‐conflict settings where survivors, perpetrators and community members are brought together to increase intergroup trust. Communal dialogue projects are influenced by the contact theory and encompass expected changes at both personal level (empathy, trust) and intergroup levels (positive behaviours, intergroup respect and trust) (CDA Collaborative Learning, 2019).

##### 5.5.1.1 How do community dialogues and reconciliations affect gender equality, women's empowerment and Peace outcomes?

**Figure 127:**
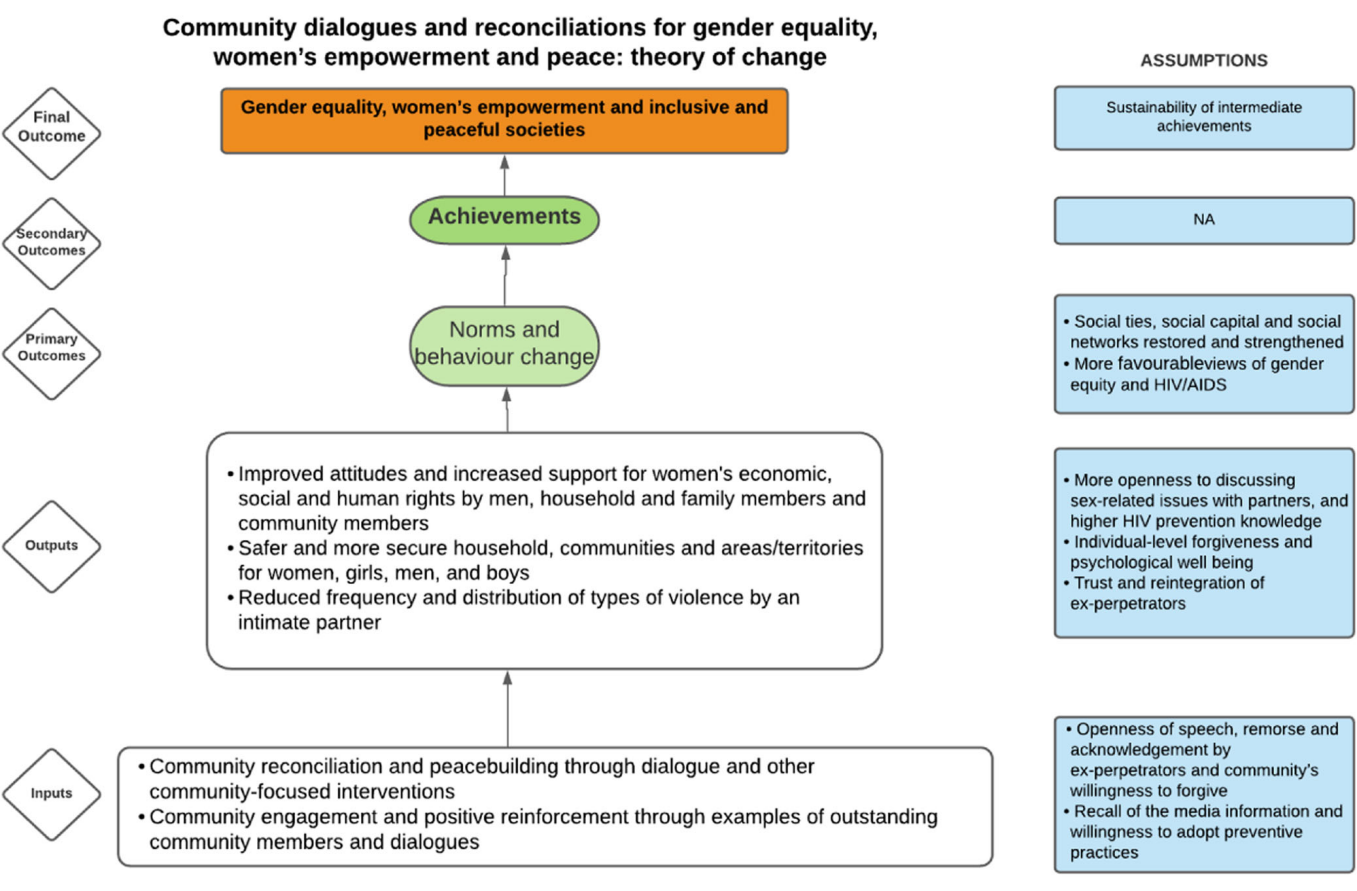
Community dialogues and reconciliations for gender equality, women's empowerment and peace: Theory of chang

Figure 127 maps out a causal chain of how community dialogues and reconciliations may improve gender equality, women's empowerment and peace outcomes. Cultural norms are hard to break because pressures to conform with the acceptable, and the avoidance of social isolation prevent actors from expressing divergent/deviant views (Scheufele & Moy, 2000). New social norms can emerge by a process of normative influence through communication networks (Kincaid, 2004). The opportunity for exchange of information on a certain topic accelerates convergence, a new view of the world, a critical consciousness among participants. This consciousness is a powerful agent of change for a new way of thinking that can evolve into new social norms (Kincaid, 2000). Dialogue groups and the media are means by which exposure to such communication can take place.

As shown in the figure below, achieving required changes in gender‐related behaviour and norms is a high‐level achievement for affected communities, and it can be made possible through community dialogues that foster an improved attitude and increased support for women's rights, reduce violence by intimate partners and create safer households and communities, when the necessary community‐ and individual‐level acceptance and networks are in place.

##### 5.5.1.2 Description of included studies

We included two studies reported in two different papers that evaluated the effects of two programmes. Each of the papers evaluated a different programme carried out in a different country.

*Population*

Both programmes evaluated involved men and women, boys and girls at the community level. The included studies evaluated two different programmes and trials in Sub‐Saharan Africa, including Mozambique and Sierra Leone.

*Intervention, inputs and activities*

The included studies evaluated a range of different community dialogues and reconciliation activities including:

- Community reconciliation and peacebuilding (*n* = 1): This intervention occurred at the level of sections, which are clusters of up to 10 contiguous villages. Two groups are established: a reconciliation committee consisting of village chiefs, religious and youth leaders, as well as some war survivors and former combatants; and an outreach committee, consisting mostly of youths. The reconciliation forum consists of a 2‐day bonfire ceremony, at which victims share their experiences and perpetrators ask for forgiveness for their war crimes. It is capped by a ‘cleansing' ceremony conducted by Sierra Leone's ‘secret societies’, where perpetrators who have expressed remorse are cleansed of their sins. After the ceremony, Fambul Tok (FT) also establishes a series of local institutional structures to further heal the community. It sets up a Peace Tree, as a symbolic gesture, which provides a focal point for resolving disputes. In some treated areas, it also creates communal farms on land set aside as a pledge towards reconciliation. Finally, it helps establish a Peace Mothers group to promote women's economic activities and discuss gender‐targeted atrocities perpetrated during the war.
- Community engagement and positive reinforcement through examples of outstanding community members and dialogues (*n* = 1): The programme had two main components: facilitated community dialogues (Tchova Tchova Histórias de Vida: Diálogos Comunitários [TTHV] sessions) using the African Transformation gender tool adapted to HIV/AIDS prevention and the TT radio magazine. The tool included nine video and written profiles of real Mozambican trendsetters. In the profiles, the men, women, and couples tell their stories of how they overcame gender, cultural, and social barriers, such as domestic violence, alcohol abuse, and the subordination of women, to make positive changes in their lives that impacted HIV treatment and prevention. To expand the reach of TTHV activities, the TT radio magazine provided reinforcing messages and featured testimonies of TTHV participants who had made changes in their lives and modelled positive behaviours. The radio magazine included 34 programmes of 12 min each, in local languages, covering topics such as condom use, sexual networks, domestic violence, dialogue on HIV between partners, HIV stigma, and gender and HIV, among others (Table 41).

**Table 41:** Community dialogues and reconciliation activities design features

Study	Activity/input	Length of treatment	Intervention frequency
Cilliers et al. (2018)	Community reconciliation and peacebuilding through dialogue and other community‐focused interventions	5 months	Weekly
Figueroa et al. (2016)	Community engagement and positive reinforcement through examples of outstanding community members and dialogues	18 months	Weekly

*Comparison*

All included studies compared treated groups to comparison groups receiving no intervention. One study included multiple treatment arms.

*Outcomes*

The included studies reported on a number of relevant outcomes, including the following:

- Improved attitudes and increased support for women's economic, social and human rights by men, household and family members and community members (*n* = 2): These positive attitudes can be shaped by sensitisation and education to shift social norms, particularly relating to marginalised groups. As a result, they are aware of the specific needs of these groups and take an active part in reducing inequalities.
- Communities have a more positive attitude towards women/marginalised groups (*n* = 1): Communities are informed and more aware of issues, characteristics and actions for women/marginalised groups and are able to act on these issues. This includes media actors (traditional and internet) publish more pieces (multimedia inclusive) focusing on these issues through gender‐transformative and gender‐specific lens.

Both of the studies (*n* = 2) measured norms and behaviour through improved attitudes and increased support for women's economic, social and human rights by men, household and family members and community members.

One of the studies (*n* = 1) also measured norms and behaviour through safer and more secure households, communities and areas/territories for women, girls, men, and boys; as well as through reduced frequency and distribution of types of violence by an intimate partner.

The division of the immediate and secondary outcomes in asset transfer is reported in Table 47 (Table 42).

**Table 42:** Summary of secondary and immediate outcomes for community dialogues and reconciliations

Secondary outcome category	Immediate outcome	Number of studies
Resources material, human and social resources which serve to enhance the ability to exercise choice	Access to justice and legal services	0
	Economic and livelihood related resources	0
	Access to employment	0
Agency ability to define one's goals and act upon them and operationalised decision‐making	Individual agency	0
	Community level agency	0
	Institutions supporting agency	0
Achievement ways of being and doing which can be realised by different individuals	Improved systems	0
	Norms and behaviour change	2
	Empowerment index	0

*Study design*

One of the community dialogues and reconciliation studies used a QED (Figueroa et al., 2016) which had some concerns related to risk of bias. The authors do not test if the results are sensitive to the existence of hidden bias, nor do they use different matching methods or report sensitivity or robustness checks, so that we could not justify the absence of confounding bias or reporting bias. However, we did not identify any major limitation and we assessed the study as having a low risk of selection bias, performance bias and outcome measurement issues.

The other community dialogues and reconciliation study used an experimental design (Cilliers et al. 2018) which had a high risk of selection bias because of issues with the implementation that led to spill‐overs. We did not identify any other issue or limitation related to any of the other domains of the RoB (assignment mechanism, unit of analysis, confounding, performance, outcome measurement and analysis reporting). Random assignment was based on a public lottery and baseline characteristics were balanced between treatment and control and the authors report low rates of attrition.

*Qualitative studies, process evaluations and project documents*

We identified only one additional document related to one programme.

- Fambul Tok's reconciliation programme (Sierra Leone): one descriptive quantitative study

This single document was ranked as high empirical quality during the appraisal process. We identified only one linked qualitative study and were unable to conduct a qualitative evidence synthesis related to community dialogues.

##### 5.5.1.3 Synthesis of findings

The following subsection presents the results of effectiveness of dialogue groups and reconciliations on gender equality, women's empowerment and peace outcomes.

*Quantitative findings*

*Effects of community dialogues and reconciliation on communities having a more positive attitude towards women/marginalised groups*

Cilliers and colleagues' (2018) experimental study in Sierra Leone was the only study evaluating the impact of community dialogues and reconciliations on communities having a more positive attitude towards women/marginalised groups. Their report included two effects that fell into this outcome category (i.e., index of attitudes towards women and attitudes towards wife beating). The effects were both very small, positive and not statistically significant point estimates (*g *=* *0.09, [95% CI: −0.01 to 0.19]) and (*g *=* *0.04, [95% CI: −0.07 to 0.14]). We assessed the study as having high risk of bias.

*Effects of community dialogues and reconciliation on improved attitudes and increased support for women's economic, social and human rights by men, household and family members and community members*

**Figure 128:**
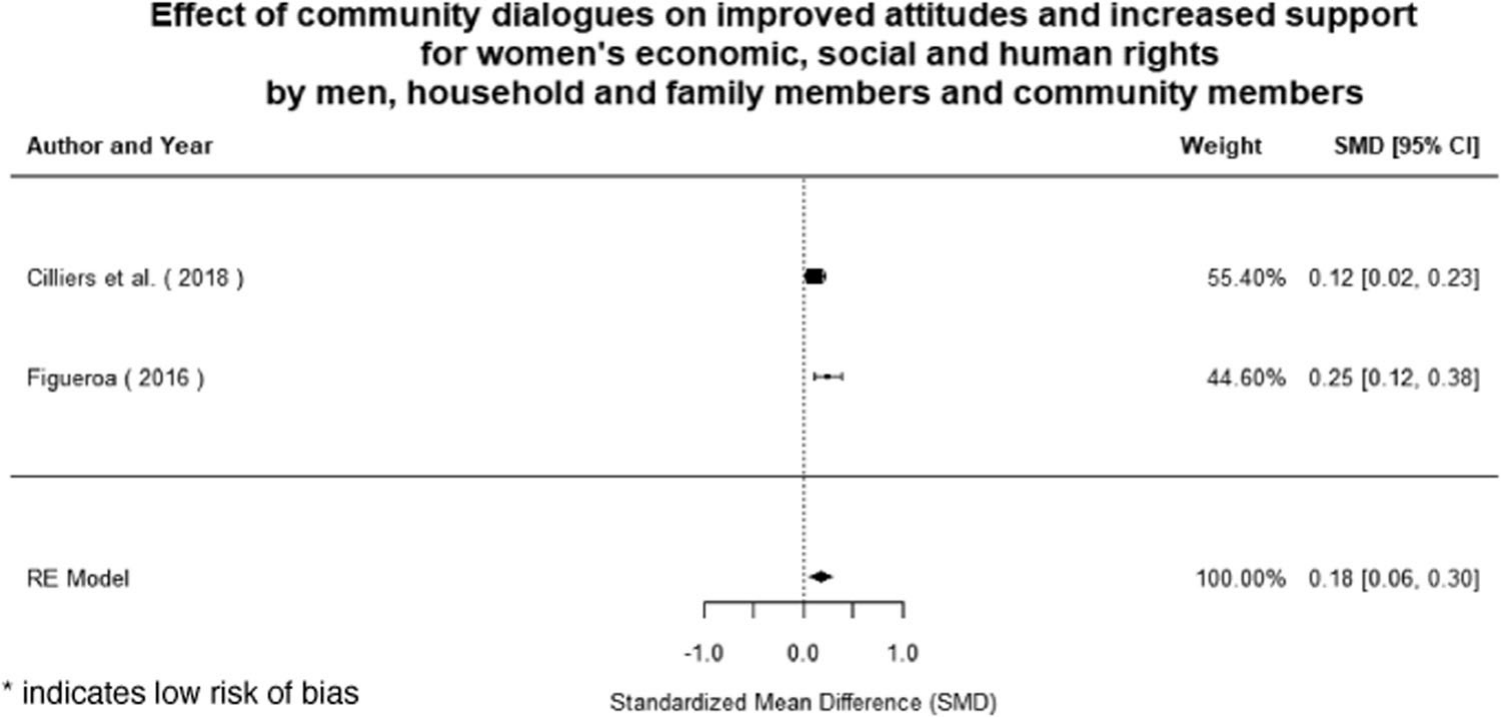
CDCB4. Forest plot showing the observed outcomes and the estimate of the random‐effects model. CI, confidence interval

Two studies reported disaggregated data for improved attitudes and increased support for women's economic, social and human rights by men, household and family members and community members, thus we included *k* = 2 studies in the analysis. We assessed one of the studies as having some concerns of risk of bias and the other as high risk of bias. The estimated average outcome based on the random‐effects model was $\hat{\mu }=0.18$ (95% CI: 0.06to0.30). Therefore, the average outcome was significantly different from zero (z=2.89, p=0.004). A forest plot showing the observed outcomes and the estimate based on the random‐effects model is shown in Figure 128 (CDCB4). Given the small number of studies, this result should be interpreted with caution. According to the Q‐test, there was no significant amount of heterogeneity in the true outcomes (Q(1)=2.19, p=0.139, ${\hat{\tau }}^{2}=0.0042$, $I^{2}=54.33$%). With only two studies, moderator analyses were not possible and tests of publication bias are not valid.

##### 5.5.1.4 Summary of findings and discussion

We included two studies in two Sub‐Saharan African countries that evaluated the effect of community dialogues and reconciliations. The was a paucity of evidence for this intervention category, thus we were only able to complete a quantitative synthesis for one outcome. We found a small positive but significant effect on improved attitudes and increased support for women's economic, social and human rights by men/households/community members. Overall, the GRADE assessments generally indicate a very low to low certainty in this body of evidence.

We identified only one linked qualitative study for community dialogues and were unable to conduct a qualitative evidence synthesis related to this intervention. Table 48 presents the summary of our quantitative findings along with the GRADE certainty of evidence ratings (Table 43).

**Table 43:** GRADE summary of findings and certainty of evidence on community dialogues and reconciliations

Certainty assessment							Sample size	Effect	Certainty	Importance
No. of studies	Study design	Risk of bias	Inconsistency	Indirectness	Imprecision	Other considerations		Absolute (95% CI)		
*(CB2) Communities have a more positive attitude towards women/marginalised groups*										
1	QED	Very serious^c^	Serious^d^	Not serious	Serious	None	2982	Two positive effect estimates with a 95% CI range of −0.01 to 0.19	⊕◯◯◯ VERY LOW	Limited importance
*(CB4) Improved attitudes and increased support for women's economic, social and human rights by men, household and family members and community members*										
2	RCT‐1 QED‐1	Very serious^a^	Not serious	Not serious	Not serious^b^	None	3872	SMD 0.18 SD higher (0.06 higher to 0.3 higher)	⊕⊕◯◯ LOW	Limited importance

Abbreviations: CI, confidence interval; GRADE, Grading of Recommendations, Assessment, Development and Evaluations; QED, quasi‐experimental design; RCT, randomised controlled trial; SMD, standardised mean difference.

^a^ This analysis has been downgraded twice due to high risk of bias in both studies. This includes one study rated as very high risk that selection bias and deviations from intended interventions.

^b^ Downgraded once to account for a *p*‐value of 0.139.

^c^ Downgraded to very serious because of significant issues related to deviation from intended intervention and other criteria.

^d^ All single studies downgraded once for inconsistency.

### 5.6 Effects of interventions under multiple UNSCR pillars: Participation and protection

Multi‐pillar interventions intersect across multiple pillars. In our study, multi‐pillar interventions both targeted the participation and protection pillars:

- The participation pillar includes interventions that create opportunities for, build acceptance of, or strengthen capacities for the equal participation and full involvement of women and girls in political, economic and social institutions and decision‐making processes.
- The protection pillar includes interventions that create, facilitate access to, or build awareness of and support for legal or social protections for women's and girls' rights. This also includes behavioural, legal and environmental interventions that aim to reduce women and girls' risk of experiencing SGBV.

In our SR, this pillar gathered the following interventions covering 47,232 beneficiaries:

- Discussion groups
- Safe spaces
- Life, social and livelihood skills and capacity building

**Figure 129:**
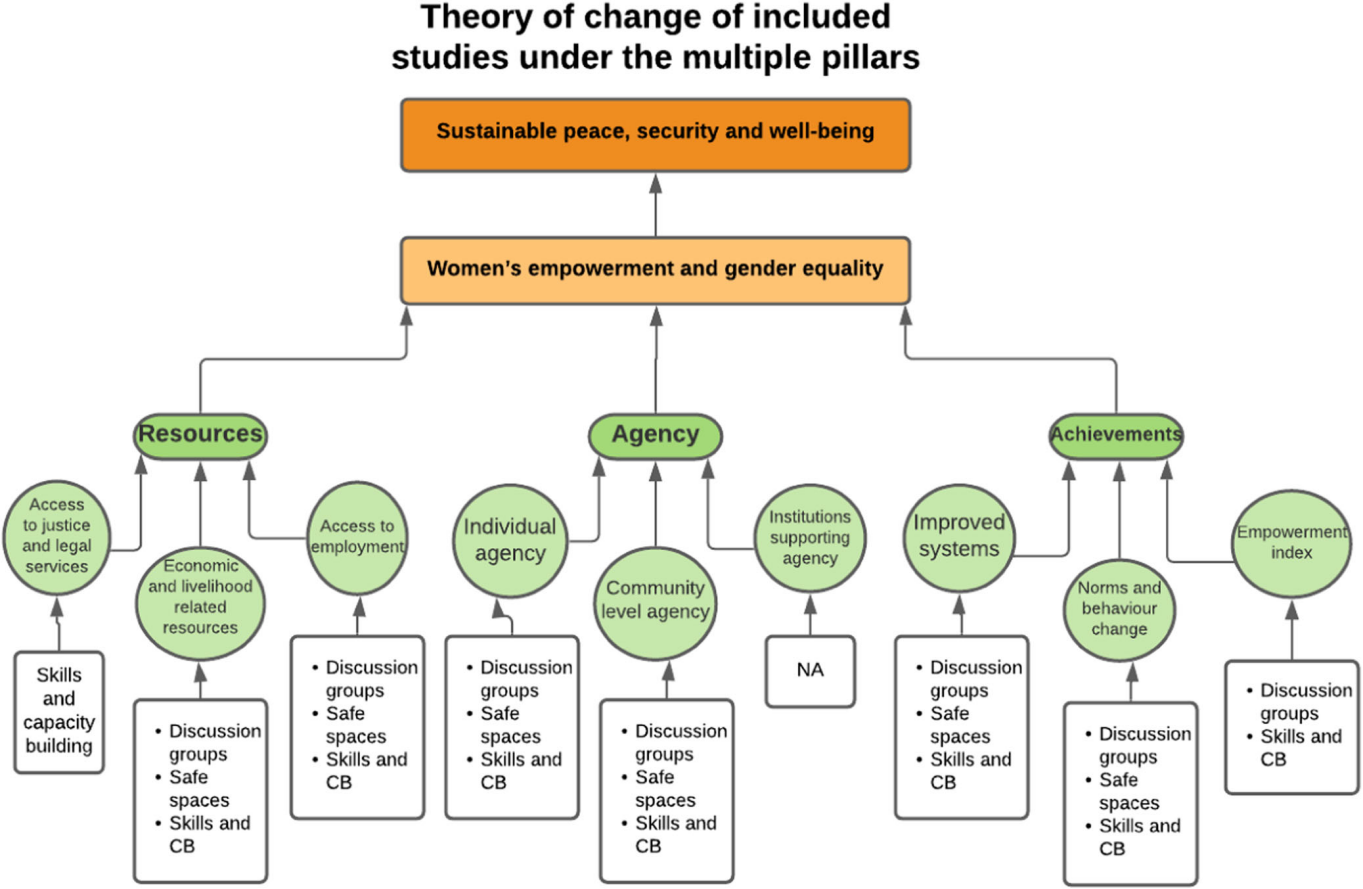
Theory of change of the included studies under multiple pillars

This section provides the findings of our synthesis of the 30 included studies evaluating the effect of these interventions on gender equality, women's empowerment and peaceful and inclusive societies. The section is organised by each intervention group. Each sub‐section starts with a description of the intervention groups, their activities and ToC, followed by descriptive results and the findings addressing our research questions. Figure 129 provides a summary of the outcomes and targeted effects of the interventions included under the multiple pillars.

#### 5.6.1 Discussion groups

Discussion groups are meetings, facilitated by a leader, amongst community members with common interests or characteristics which aim to expand understanding, improve attitudes, or change behaviours through conversation. The emphasis is on people with social capital, authority and influence, whose views and behaviours, if directed at reconciliation, can result in positive change for the wider community (CDA Collaborative Learning, 2019).

##### 5.6.1.1 How do discussion groups affect gender equality, women's empowerment and Peace outcomes?

**Figure 130:**
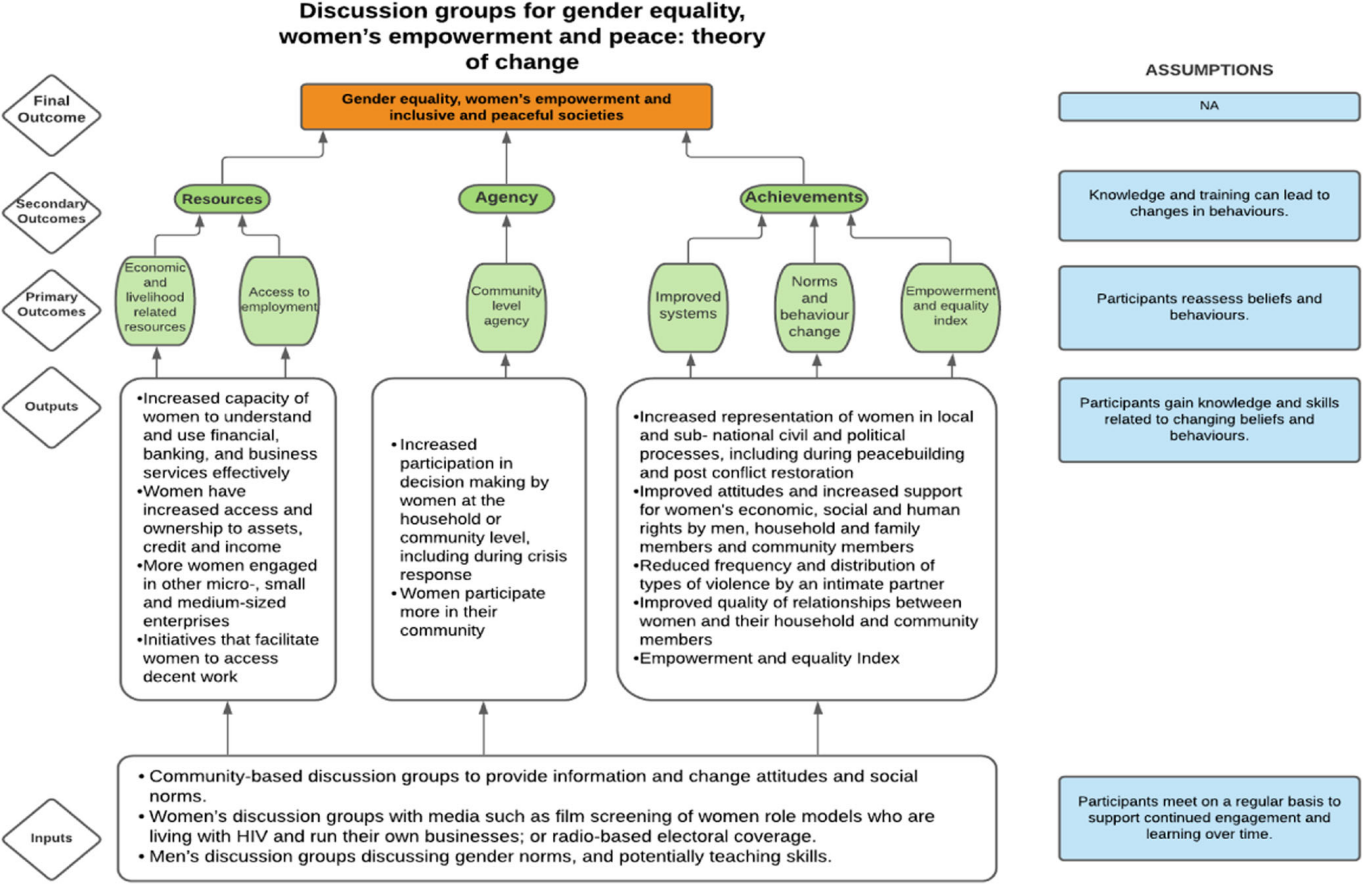
Discussion groups for gender equality, women's empowerment and peace: Theory of change

Figure 130 maps out the causal chain of how discussion groups may improve gender equality, women's empowerment and peace outcomes.

Engaging women and men in discussion groups that convey information through a curriculum, media, or other means would aim to improve participants' knowledge and skills. Gaining knowledge would help participants reassess their beliefs and behaviours in favour of more equitable social norms and relationships. Participants' reflection would lead to gradual and overall change in their beliefs and behaviours, and contribute to advancing gender equality, women's empowerment, and inclusive and peaceful societies. Assumptions associated with this ToC include participants meeting on a regular basis, such as weekly or monthly, to build dialogue and engagement with the topics over time. While knowledge and training are assumed to lead to changes in behaviours at the individual level and potentially at the household and community levels, longer timeframes may be needed to capture community‐level change, and interventions may need to focus beyond individual participants to change community norms. For example, a discussion group that regularly engages men about gender norms and teaches conflict management skills would aim to help participants reflect on their beliefs and identify ways that they could change their behaviours, such as improving the quality of their relationships with women in their households and/or at the community level. Changes in attitudes and beliefs about gender relations could lead to changes in behaviour and reduce incidents of SGBV, IPV and related consequences of harmful social norms, though the latter would emerge gradually. If achieved, reductions in violence could contribute to a more peaceful and equitable society.

##### 5.6.1.2 Description of included studies

We included four studies from five different papers that evaluated the effect of two programmes in LMICs. We included more than one paper that evaluated the same programme if the author(s) reported different outcomes over several papers.

*Population*

The targeted populations for discussion group interventions included women only, men only, women and men, or women and/or men who had undergone testing for HIV or who had tested positive for HIV. Discussion group interventions generally targeted changes in beliefs, attitudes and behaviours at the individual level, though some interventions, such as those focusing on political participation or gender relations, also targeted change at the household or community levels.

The included studies evaluated two different programmes in four countries in Sub‐Saharan Africa: Côte d'Ivoire, DRC, Liberia and Uganda.

*Intervention, inputs and activities*

The included studies evaluated a range of different discussion group activities and inputs including the following:

- Discussion groups related to SGBV (*n* = 3): Groups generally separated by gender convened to discuss attitudes and behaviours about SGBV and IPV, gender relations and gender rights. Some groups also received skills training.
- Discussion groups with radios (*n* = 1): Women were provided radios and listened to electoral coverage.
- Short films at HIV/AIDS clinics (*n* = 1): Women at these clinics participated in discussion groups and watched short films featuring women entrepreneurs living with HIV (Table 44).

**Table 44:** Discussion group activities design features of included studies

Study	Activity/input	Length of treatment	Intervention frequency
Wagman et al. (2015)	Discussion groups related to SGBV	35 months	NA
Vaillant et al. (2020)	Discussion groups related to SGBV	NA	Weekly
Vaillant (2016)	Discussion groups related to SGBV	NA	NA
Hossain et al. (2014)	Discussion groups related to SGBV	5 months	Weekly
Mvukiyehe (2017)	Discussion groups with radios	NA	Weekly
Lubega et al. (2017)	Short films at HIV clinics	10 months	Monthly

Abbreviation: SGBV, sexual and gender‐based violence.

*Comparison*

All the included studies compared treated groups to comparison groups receiving no intervention.

*Outcomes*

The included studies reported on a number of relevant outcomes, including:

- Women have increased access and ownership to assets, credit and income (*n* = 2): Women are able to apply for, receive and manage assets/credit and income and have support to manage, claim and execute their assets without pressure or influence from external actors, including male family members, husbands and cultural leaders.
- Increased participation in decision making by women at the household or community level, including during crisis (*n* = 2): Women take part in all or any step of the decision‐making process at the household community and district level, but also are able to meaningfully take part and have influence on the final decision, including in crisis response.
- Improved attitudes and increased support for women's economic, social and human rights by men, household and family (*n* = 2): These positive attitudes can be shaped by arginalized and education to shift social norms, particularly relating to arginalized groups. As a result, they are aware of the specific needs of these groups and take an active part in reducing inequalities.
- Reduced frequency and distribution of types of violence by an intimate partner (*n* = 1): Types of violence in this case include four types of IPV: physical violence, sexual violence, stalking, and psychological aggression. These incidences are reduced over time due to a bundle of interventions that focus on both support for survivors and sanctioning perpetrators.

The separation of the immediate and secondary outcomes is reported in Table 50 (Table 45).

**Table 45:** Summary of secondary and immediate outcomes for discussion groups

Secondary outcome category	Immediate outcome	Number of studies
Resources material, human and social resources which serve to enhance the ability to exercise choice	Access to justice and legal services	0
	Economic and livelihood related resources	2
	Access to employment	1
Agency ability to define one's goals and act upon them and operationalised decision‐making	Individual agency	1
	Community‐level agency	2
	Institutions supporting agency	0
Achievement ways of being and doing which can be realised by different individuals	Improved systems	1
	Norms and behaviour change	2
	Empowerment index	1

*Study design*

**Figure 131:**
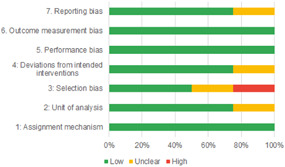
Discussion groups studies randomised controlled trial risk of bias assessment

The four included studies evaluating discussion groups used an experimental design (Hossain et al., 2014; Lubega et al., 2017; Mvukiyehe, 2017; Vaillant et al., 2020). One of the four studies was assessed as having a high risk of bias. As detailed in Figure 131, high risk of bias was identified in the selection domain because of a large difference in attrition rates between the treatment and control groups (Hossain et al., 2014). Two studies were assessed as having some concerns of risk of bias. The first did not provide enough information for us to justify low risk of deviations from intended interventions, no mention of spill‐overs, crossovers or contamination was observed, and no information of geographical dispersion that may have dissuaded implementation issues (Vaillant et al., 2020). We assessed another study as unclear on selection and reporting bias because the authors do not discuss attrition and do not provide enough information to justify the absence of selective reporting of the analysis and the results. We did not identify any limitations related to the assignment mechanics, performance and behavioural changes in participants and not participants relate to the knowledge of being observed and compared, or outcome measurement.

*Qualitative studies, process evaluations and project documents*

We identified two additional documents related to one programme covered by the discussion group section of included studies.

- Engaging Men through Accountable Practice (EMAP) (DRC): one descriptive quantitative and one qualitative study

One study was marked as high empirical quality and the other was marked as moderate. We identified only two qualitative studies and were unable to conduct a qualitative evidence synthesis related to the discussion group intervention.

##### 5.6.1.3 Synthesis of findings

The following subsection presents the results of effectiveness of discussion groups on gender equality, women's empowerment and peace outcomes.

*Quantitative findings*

*Effects of discussion groups on women having increased access to and ownership of assets, credit and income*

**Figure 132:**
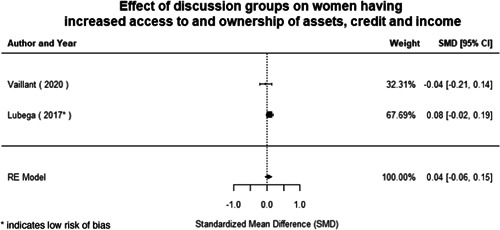
DGAB2. Forest plot showing the observed outcomes and the estimate of the random‐effects model. CI, confidence interval

Two studies examined the effects of discussion groups on women having increased access to and ownership of assets, credit, and income, thus we included *k *=* *2 studies in the analysis. We assessed one of the studies as low risk of bias and the other as some concerns. The estimated average outcome based on the random‐effects model was $\hat{\mu }=0.04$ (95% CI: −0.06to0.15). Therefore, the average outcome did not differ significantly from zero (z=0.80, p=0.42). A forest plot showing the observed outcomes and the estimate based on the random‐effects model is shown in Figure 132 (DGAB2). Given the small number of studies, this result should be interpreted with caution. According to the Q‐test, there was no significant amount of heterogeneity in the true outcomes (Q(1)=1.37, p=0.24, ${\hat{\tau }}^{2}=0.002$, $I^{2}=26.92$%). One study was appraised as having *some concerns* with risk of bias (Vaillant et al., 2020) and the other (Lubega et al., 2017) was assessed as having a low risk of bias. With only two studies, moderator analyses were not possible and tests of publication bias are not valid.

*Effects of discussion groups on engaging more women in other micro, small and medium‐sized enterprises*

Lubega et al.'s (2017) experimental study in Uganda was the only study evaluating the impact of discussion groups on increasing women's engagement in other micro, small, and medium‐sized enterprises. Their report included three effects that fell into this outcome category (e.g., whether a woman operates an enterprise or open a retail shop). The effects ranged from a null point estimate (*g *=* *0.00, [95% CI: −0.10 to 0.10]) for whether a woman operates an enterprise to a small, positive point estimate (*g *=* *0.13, [95% CI: −0.13 to 0.40]) for whether women open a retain shop among the subgroup of women who start a business. We assessed the study as having low risk of bias.

*Effects of discussion groups on whether women have more positive attitude towards taking action to claim their rights*.

Mvukiyehe's (2017) experimental study in Liberia was the only study evaluating the impact of discussion groups on whether women have more positive attitudes towards taking action to claim their rights. Their report included 19 effects that fell into this outcome category (e.g., such as attending strategic meetings or strategic voting). The effects ranged from a large negative effect (*g *=* *−0.52, [95% CI: −0.74 to −0.30]) for an overall index measuring political behaviour to a small, positive point estimate (*g *=* *0.15, [95% CI: −0.07 to 0.36]) for whether women had their ballot marked by someone else. We assessed the study as having some risk of bias concerns.

*Effects of discussion groups on increased representation of women in local and subnational civil and political processes, including during peacebuilding and post conflict restoration*.

Mvukiyehe's (2017) experimental study in Liberia was the only study evaluating the impact of discussion groups on increased representation of women in local and subnational civil and political processes, including during peacebuilding and post conflict restoration. There was a large, and statistically significant, negative effect (*g *=* *−0.47, [95% CI: −0.76 to −0.18]), and we assessed the study as having some risk of bias concerns.

*Effects of discussion groups on improved attitudes and increased support for women's rights by men, household and family members and community members*

**Figure 133:**
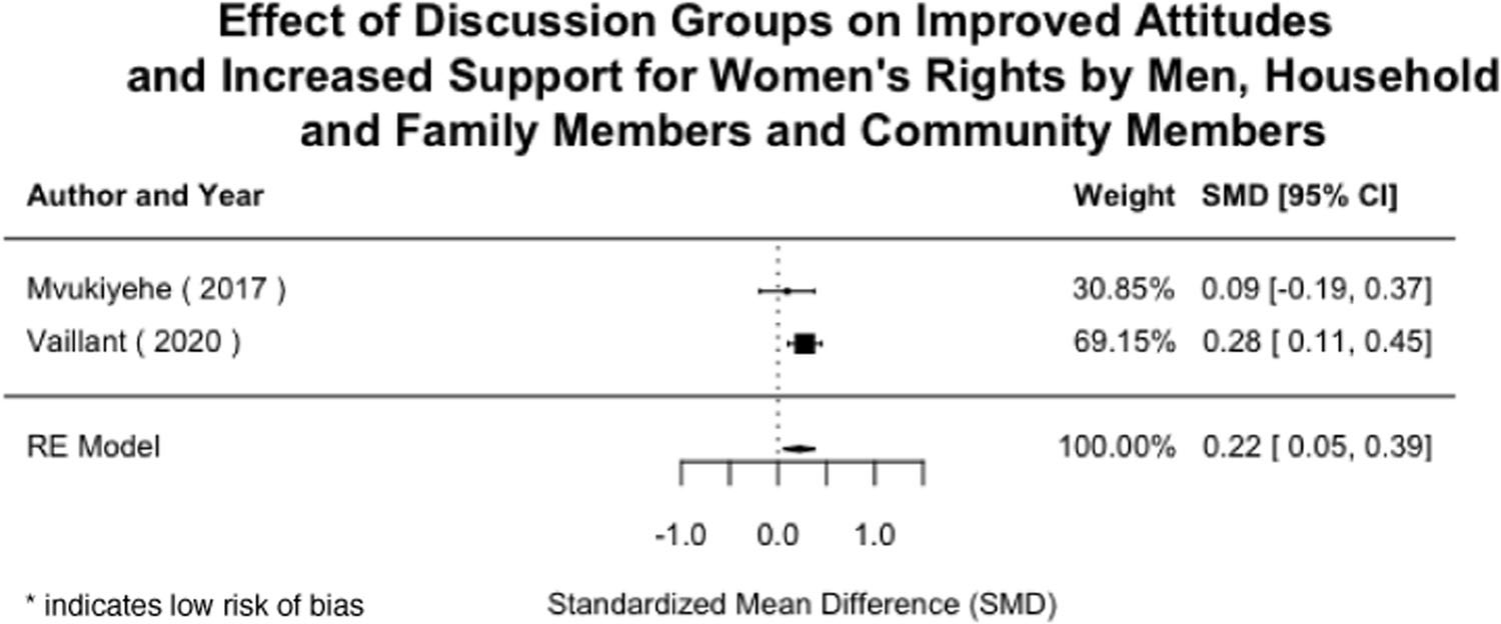
DGCB4. Forest plot showing the observed outcomes and the estimate of the random‐effects model. CI, confidence interval

Two studies examined the effects of discussion groups on improved attitudes and increased support for women's rights by men, household and family members and community members, thus we included −*k *=* *2 studies in the analysis (see Table 51). We assessed both of the studies as having some concerns of risk of bias. The estimated average outcome based on the random‐effects model was $\hat{\mu }=0.2199$ (95% CI: 0.0542to0.3857). Therefore, the average outcome differed significantly from zero (z=2.6003, p=0.0093). A forest plot showing the observed outcomes and the estimate based on the random‐effects model is shown in Figure 133 (DGCB4). Given the small number of studies, this result should be interpreted with caution. According to the Q‐test, there was no significant amount of heterogeneity in the true outcomes (Q(1)=1.19, p=0.27, ${\hat{\tau }}^{2}=0.0027$, $I^{2}=16.20$%). Both studies were appraised as having *some concerns* with risk of bias and these results should be applied cautiously. With only two studies, moderator analyses were not possible and tests of publication bias are not valid.

*Effects of discussion groups on an improved quality of relationships between women and their household and community members*

Vaillant et al.'s (2020) experimental study in Democratic Republic of Congo was the only study evaluating the impact of discussion groups on improved quality of relationships between women and their household and community members. There was a small, but not statistically significant, point estimate (*g *=* *0.15, [95% CI: −0.02 to 0.32]), and we assessed the study as having some risk of bias concerns.

*Effects of discussion groups on women's empowerment index*

Mvukiyehe's (2017) experimental study in Liberia was the only study evaluating the impact of discussion groups on women's empowerment index. There was a small, but not statistically significant, point estimate (*g *=* *−0.11, [95% CI: −0.39 to 0.17]), and we assessed the study as having some risk of bias concerns.

##### 5.6.1.4 Summary of findings and discussion

We included four studies in four Sub‐Saharan African countries that evaluated the effect of discussion groups. Only two outcomes had sufficient evidence to conduct a quantitative synthesis. There was a significant positive effect of discussion groups on improved attitudes and increased support for women's economic, social and human rights by men/household/community members. There was a positive but nonsignificant point estimate for of discussion groups on women having increased access to and ownership of assets, credit and income. Overall, the GRADE assessments generally indicate a very low to moderate certainty in this body of evidence.

We identified no linked qualitative studies and were unable to conduct a qualitative evidence synthesis related to the discussion groups intervention. The summary of our quantitative findings along with the GRADE certainty of evidence ratings are presented in Table 51 (Table 46).

**Table 46:** GRADE summary of findings and certainty of evidence on discussion groups

Certainty assessment							Sample size	Effect	Certainty	Importance
No. of studies	Study design	Risk of bias	Inconsistency	Indirectness	Imprecision	Other considerations	Discussion groups	Absolute (95% CI)		
*(AB2) Women have increased access and ownership to assets, credit and income*										
2	RCT‐2	Not serious	Serious^a^	Not serious	Not serious	None	2515	SMD 0.04 SD higher (0.06 lower to 0.15 higher)	⊕⊕⊕◯ MODERATE	Important, but not critical
*(AC1) More women engaged in other micro, small and medium‐sized enterprises*										
1	RCT	Not serious	Serious^b^	Not serious	Not serious	None	1502	One null effect and two positive effect estimates with a 95% CI range of −0.12 to 0.39	⊕⊕⊕◯ MODERATE	Important, but not critical
*(BA5) Women have more positive attitude towards taking action to claim their rights*										
1	RCT	Serious^c^	Serious^b^	Not serious	Not serious	None	190	SMD 0.19 SD higher (0.09 lower to 0.47 higher)	⊕⊕◯◯ LOW	Limited importance
*(CA2) Increased representation of women in local and subnational civil and political processes, including during peacebuilding and post conflict restoration*										
1	RCT	Serious^d^	Serious^b^	Not serious	Serious^e^	None	190	One null, 4 negative and 15 positive effect sizes with a 95% range of −0.76 to 0.37	⊕◯◯◯ VERY LOW	Limited importance
*(CB4) Improved attitudes and increased support for women's economic, social and human rights by men, household and family members and community members*										
2	RCT‐2	Not serious	Serious^f^	Not serious	Serious^g^	None	1245	SMD 0.22 SD higher (0.05 higher to 0.39 higher)	⊕⊕◯◯ LOW	Limited importance
*(CB8) Improved quality of relationships between women and their household and community members*										
1	RCT	Serious^h^	Serious^b^	Not serious	Serious^i^	None	1051	SMD 0.15 SD higher (0.02 lower to 0.32 higher)	⊕◯◯◯ VERY LOW	Limited importance
*(CC1) Empowerment/Equality Index*										
1	RCT	Serious^j^	Serious^b^	Not serious	Serious^k^	None	194	SMD 0.11 SD lower (0.39 lower to 0.17 higher)	⊕◯◯◯ VERY LOW	Important, but not critical

Abbreviations: CI, confidence interval; GRADE, Grading of Recommendations, Assessment, Development and Evaluations; RCT, randomised controlled trial; SMD, standardised mean difference.

^a^ Although the point estimates are quite close to null, this has been downgraded because of wide confidence intervals suggesting inconsistency.

^b^ Downgraded once for one study in the group.

^c^ Downgraded because the study presents uncertain risk of bias with regard to selection bias and reporting bias.

^d^ Downgraded because the study presents uncertain risk of bias with regard to selection bias and reporting bias.

^e^ Downgraded because this single study reports a wide range of effect sizes measuring this outcome, including on both sides of the threshold.

^f^ Downgraded to reflect significantly different point estimates whose CIs, despite overlapping, are very wide.

^g^ Downgraded because the point estimate's CIs are wide and vary considerably relative to one of the studies in the analysis.

^h^ Downgraded because the study presents uncertain risk of bias due to deviation from the intended intervention.

^i^ Downgraded to reflect the very wide range of CIs surrounding the point estimates.

^j^ Downgraded because the study presents uncertain risk of bias with regard to selection bias and reporting bias.

^k^ Downgraded to reflect the very wide range of CIs surrounding the point estimates.

#### 5.6.2 Safe spaces

Safe spaces are formal or informal gathering places where women and girls feel physically and emotionally safe, access services, participate in training and skills development activities, join peer support networks, mentorship and so on. Safe spaces are group‐centred mechanisms to articulate and accommodate the preferences of women and the girl‐child. Safe spaces provide participants with a welcoming atmosphere that can enhance their opening up on sensitive issues as particular to them, individually and/or collectively. Safe spaces shoulder the responsibility of knowledge dissemination through developmental activities and mentoring (UNFPA, 2015).

##### 5.6.2.1 How do safe spaces affect gender equality, women's empowerment and Peace outcomes?

**Figure 134:**
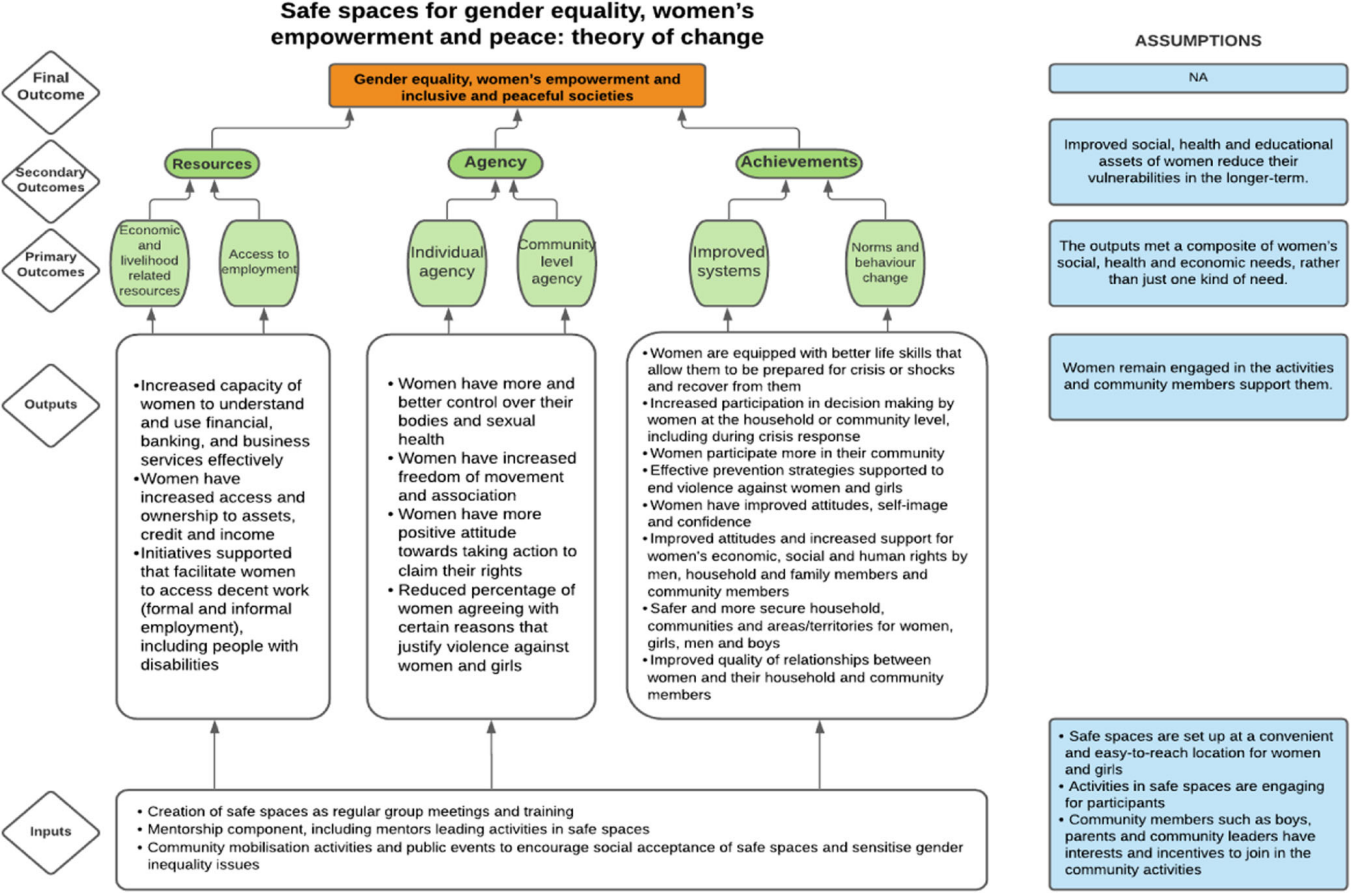
Safe spaces for gender equality, women's empowerment and peace: Theory of change

Figure 134 maps out the causal chain of how safe spaces may improve gender equality, women's empowerment and peace outcomes.

The main inputs are safe space programmes that involve regular group sessions for education, discussions and peer networking activities. Curriculums mainly focus on literacy, sexual and reproductive health, financial education and other life skills. The activities are typically led by mentors from local communities, who support and build trust with programme participants within and outside of safe spaces. In some cases, programme components include activities for community members such as boys, parents and community leaders to sensitise gender inequality issues and encourage their support and acceptance of safe spaces for women. The assumptions are that locations of safe spaces are easily accessible by women, and both safe space participants and community members have sustained interest and incentives to participate in the activities.

The outputs of these activities include women's improved knowledge of a range of topics such as life skills, greater access to assets and more decent work, better control over their bodies and sexual health, self‐efficacy, and freedom of movement and participation in their households and communities. The community members will improve their attitudes towards women's economic, social and human rights. Relationships between women and their household and community members will be enhanced. It is crucial that participants stay engaged in the safe space programmes, and community members keep supporting them.

These will result in immediate outcomes, such as improved access to economic and livelihood related resources, access to employment, individual and community level agency, improved systems, and norms and behaviour change. It is assumed that the outputs meet a combination of women's social, health, and economic assets rather than one kind of asset only. For example, it was claimed that only improving girls' knowledge of sexual and reproductive health itself is insufficient to yield any expected outcomes, as their vulnerable economic situation overshadows their knowledge (Austrian & Muthengi, 2014). Finally, these immediate outcomes will catalyse women's improved resources, agency and achievements, which will then lead to better gender equality, empowerment and inclusive and peaceful societies.

##### 5.6.2.2 Description of included studies

We included six studies reported in six different papers that evaluated the effect of five programmes. We included more than one paper that evaluated the same programme if the author(s) reported different outcomes over several papers.

*Population*

All programmes targeted adolescent girls aged between 10 and 19, except the *Powering up Biruh Tesfa* programme in Ethiopia where girls under 10 years old were included (Erulkar & Medhin, 2017). Around half of the programmes explicitly targeted girls in low‐income areas (*n* = 3). Two programmes in Ethiopia and Egypt targeted out‐of‐school girls only (Erulkar & Medhin, 2017; Sieverding & Elbadawy, 2016). One programme in Zambia targeted only girls who had never married at baseline (Austrian et al., 2020).

The included studies evaluated five different programmes and trials in MENA and Sub‐Saharan Africa, covering six countries including: Egypt, Ethiopia, Niger, Tanzania, Uganda and Zambia.

*Intervention, inputs and activities*

The included studies evaluated a range of different safe space activities and inputs including:

- Safe spaces with access to economic and/or health assets (*n* = 4): Safe space programmes that involve regular group sessions and training led by mentors, with the provision of vouchers for free or subsidised health services, and/or assistance to open savings accounts for girls.

Safe spaces with community activities (*n* = 2): Safe space programmes that involve regular group sessions and training led by mentors, with community activities (e.g., meetings, seminars or public events) to encourage acceptance of safe spaces and sensitisation of gender inequality issues within communities (Table 47).

**Table 47:** Safe spaces activities and design features of included studies

Study	Activity/input	Length of treatment	Intervention frequency
Austrian and Muthengi (2014)	Safe spaces with regular group meetings, training, mentoring, provision of savings accounts	12 months	Weekly
Austrian et al. (2020)	Safe spaces with regular group meetings, training, mentoring, provision of savings accounts and vouchers for free health services	24 months	Weekly
Buehren, Goldstein, et al. (2017)	Safe spaces with recreational activities and education, mentoring, community activities (e.g., periodic meetings with village members)	24 months	Daily
Erulkar and Medhin (2017)	Safe spaces with education, mentoring, provision of vouchers for free or subsidised health services	6 months	Daily
Mercy Corps (2015)	Safe spaces with regular group meetings and education, mentoring, provision of savings account and conditional lentil ration	8–20 months	Weekly
Sieverding and Elbadawy (2016)	Safe spaces with education, mentoring, community activities (e.g., seminars, public events), provision of conditional food ration	36 months	Daily

*Comparison*

All included studies are comparing treated groups to comparison groups receiving no intervention. Four studies included multiple treatment arms.

*Outcomes*

The included studies reported on a number of relevant outcomes, including the following:

- Women have increased access and ownership to assets, credit and income (*n* = 4): Women are able to apply for, receive and manage assets/credit and income and have support to manage, claim and execute their assets without pressure or influence from external actors, including male family members, husbands and cultural leaders.
- Women have more and better control over their bodies and sexual health (*n* = 2): Women are able to make free and educated decisions about their health (not included in this study), sexuality and procreation.
- Women have a more positive attitude towards taking action to claim their rights (*n* = 1): Women feel entitled to be engaged and given the leadership capacity and knowledge to claim their rights and take action on relevant issues. Increased self‐efficacy and autonomy. Increased opportunities for women to claim their rights including as a result of education and sensitisation. Women are engaged and given the leadership capacity and knowledge to claim their rights and take action on relevant issues.
- Women are equipped with better life skills that allow them to be prepared for crisis or shocks and recover from them (*n* = 4): Women develop skills, have access to resources, and make use of these skills and resources to increase their level of resilience and be equipped to face life challenges (health, education, finance, social relations, work, etc.).
- Women have improved attitudes, self‐image and confidence (*n* = 4): Women feel more entitled to claim their rights and needs in community and change social norms and behaviours. They are aware of the importance of their status in society and are empowered to take this role and make use of the available resources to guarantee their rights.

We report the division of the immediate and secondary outcomes in Table 53 (Table 48).

**Table 48:** Summary of secondary and immediate outcomes

Secondary outcome category	Immediate outcome	Number of studies
Resources material, human and social resources which serve to enhance the ability to exercise choice	Access to justice and legal services	0
	Economic and livelihood related resources	4
	Access to employment	1
Agency ability to define one's goals and act upon them and operationalised decision‐making;	Individual agency	5
	Community level agency	4
	Institutions supporting agency	0
Achievement ways of being and doing which can be realised by different individuals	Improved systems	2
	Norms and behaviour change	6
	Empowerment index	0

*Study design*

**Figure 135:**
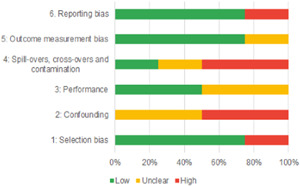
Safe spaces studies quasi‐experimental design risk of bias assessment

Four safe spaces studies used a QED. This included Austrian and Muthengi (2014), Erulkar and Medhin (2017), Mercy Corps (2015), Sieverding and Elbadawy (2016). We assessed three of these as having a high risk of bias. As detailed in Figure 135, high risk of bias was identified in four RoB domains (selection, confounding, spill‐overs and reporting). Selection bias was an issue in a study using difference in differences without adjusting for any covariates (Sieverding & Elbadawy, 2016), differential attrition posed risks of confounding (Austrian & Muthengi, 2014; Sieverding & Elbadawy, 2016), spill‐overs were an issue in interventions where participants assigned to the control group received the intervention (Mercy Corps, 2015), and no robustness tests were reported in one of the studies that used the difference in differences but did not match the treatment and comparison groups based on the observable characteristics, so that self‐selection was not accounted for (Sieverding & Elbadawy, 2016).

**Figure 136:**
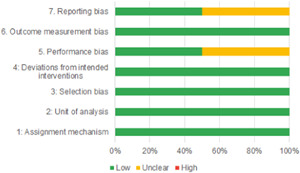
Safe spaces studies quasi‐experimental design risk of bias assessment

Two safe spaces studies used an experimental design (Austrian et al., 2020; Buehren, Goldstein, et al., 2017). Both studies scored some concerns related to risk of performance and reporting bias, yet we did not identify any major issue. As detailed in Figure 136, high risk of bias was not identified in any RoB domain of the sample of included studies, but the authors did not report enough information to justify the absence of performance bias in one study (Austrian et al., 2020) and reporting bias in the other (Buehren, Goldstein, et al., 2017).

*Qualitative studies, process evaluations and project documents*

We identified four additional documents related to three programmes covered by the safe spaces studies. Programmes are as follows:

- Ishraq (Egypt): one qualitative study and one process evaluation
- Adolescent Girls Empowerment Programme (AGEP) (Zambia): one qualitative study
- Sawki ‘Safe Space’ and ‘Safe Space + Livelihoods’ (Niger): one descriptive quantitative study

Half (*n* = 2) were marked as moderate quality and the remainder (*n* = 2) marked as low‐quality documents.

##### 5.6.2.3 Synthesis of findings

The following subsection presents the results of effectiveness of safe spaces on gender equality, women's empowerment and peace outcomes.

*Quantitative findings*

*Effects of safe spaces on women's increased capacity to understand and use financial, banking, and business services effectively*

**Figure 137:**
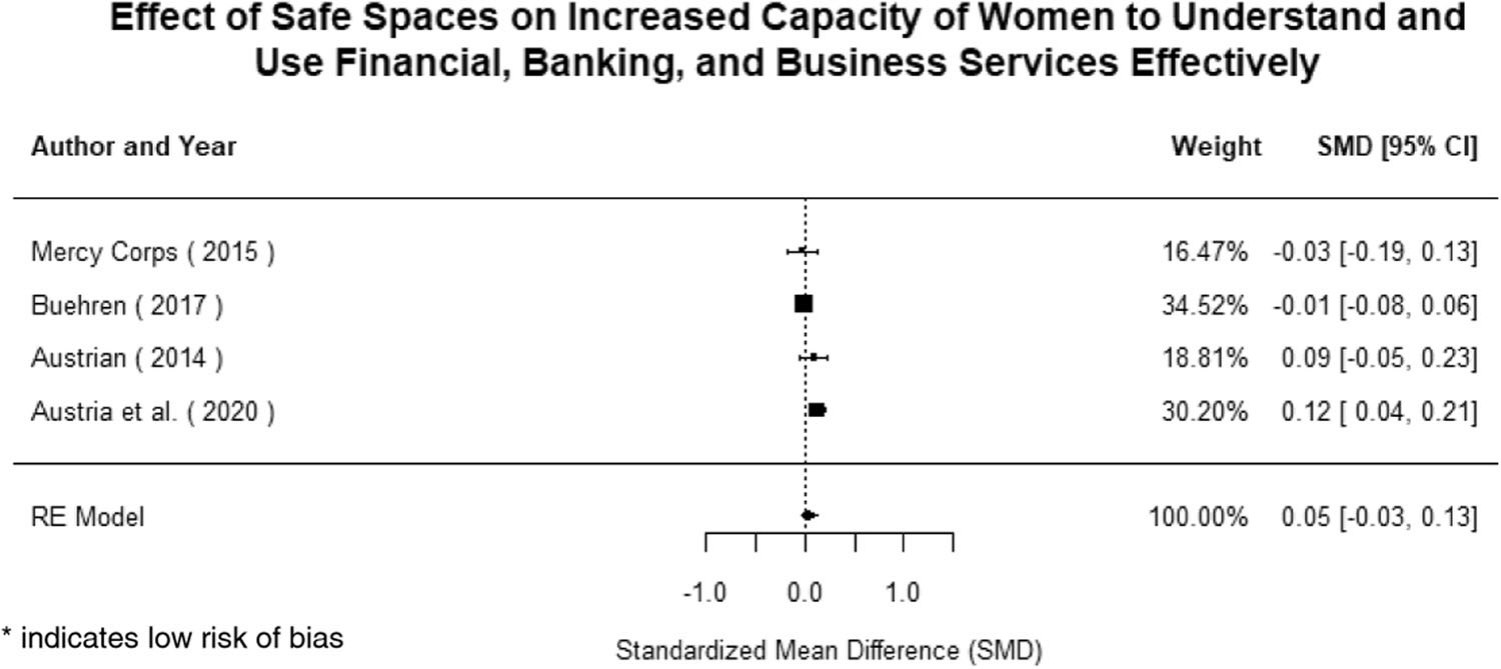
SSAB1: Forest plot showing the observed outcomes and the estimate of the random‐effects model. CI, confidence interval

We included a total of k=4 studies in the analysis. The observed outcomes ranged from −0.03 to 0.12. We assessed none of the studies as low risk of bias, two as some concerns, and two as high risk of bias. The estimated average outcome based on the random‐effects model was $\hat{\mu }=0.05$ (95% CI: −0.03 to 0.13). Therefore, the average outcome did not differ significantly from zero (z=1.13, p=0.26). A forest plot showing the observed outcomes and the estimate based on the random‐effects model is shown in Figure 137 (SSAB1).

The Q‐test for heterogeneity was not significant, but some heterogeneity may still be present in the true outcomes (Q(3)=6.78, p=0.08, ${\hat{\tau }}^{2}=0.00$, $I^{2}=55.77$%). An examination of the studentised residuals revealed that none of the studies had a value larger than ±2.50 and hence there was no sign of outliers in the context of this model. According to the Cook's distances, none of the studies could be overly influential. Given the nonsignificant test for heterogeneity, we did not proceed with moderator analyses.

*Effects of safe spaces on women's increased access to and ownership of assets, credit and income*

**Figure 138:**
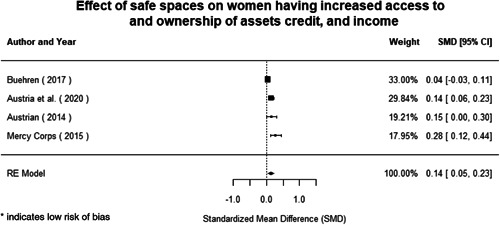
SSAB2: Forest plot showing the observed outcomes and the estimate of the random‐effects model. CI, confidence interval

We included a total of k=4 studies in the analysis. We assessed none of the studies as low risk of bias, two as some concerns, and two as high risk of bias. The observed outcomes ranged from 0.04 to 0.28. The estimated average outcome based on the random‐effects model was $\hat{\mu }=0.14$ (95% CI: 0.05 to 0.23). Therefore, the average outcome differed significantly from zero (z=2.95, p<0.01). A forest plot showing the observed outcomes and the estimate based on the random‐effects model is shown in Figure 138 (SSAB2).

According to the Q‐test, the true outcomes appear to be heterogeneous (Q(3)=8.45, p=0.04, ${\hat{\tau }}^{2}=0.01$, $I^{2}=64.48$%). An examination of the studentised residuals revealed that none of the studies had a value larger than ±2.50 and hence there was no indication of outliers in the context of this model. According to the Cook's distances, none of the studies could be overly influential.

We found significant moderation by exposure to intervention ($\hat{B}=-0.007,p=0.006$ [95% CI: −0.01 to −0.002]) such that each additional month of exposure decreased the impact by 0.007 standard deviation units. Effects did not differ by risk of bias, study design, or publication year. We were unable to test evaluation period (all were evaluated immediately after the end of the intervention), whether the model was adjusted for covariates, or change from baseline versus post‐intervention changes.

*Effects of safe spaces on improved capacity of women entrepreneurs*

Buehren, Goldstein, et al.'s (2017) experimental study in Tanzania was the only study evaluating the impact of safe spaces on improved capacity of women entrepreneurs. Their report included two effects that fell into this outcome category (e.g., whether the respondent indicated that she was planning to start a new income‐generating activity). The effects are reported separately for each treatment village. For Treatment Village A, the effect was a very small, positive point estimate (*g *=* *0.02, [95% CI: −0.05 to 0.09]), and for Treatment Village B the effect was a very small, positive point estimate (*g *=* *0.02, [95% CI: −0.05 to 0.09]). We assessed the study as having some risk of bias concerns.

*Effects of safe spaces on women's control over their bodies and sexual health*

**Figure 139:**
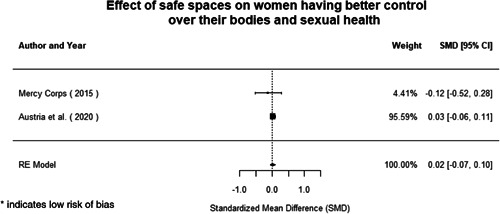
SSBA2: Forest plot showing the observed outcomes and the estimate of the random‐effects model. CI, confidence interval

Only two studies reported the impact of safe spaces on women's control over their bodies and sexual health, thus we included *k *=* *2 studies in the analysis. We assessed both studies as high risk of bias. The estimated average outcome based on the random‐effects model was $\hat{\mu }=0.02$ (95% CI: −0.07to0.10). This positive effect did not differ significantly from zero (z=0.44, p=0.66). A forest plot showing the observed outcomes and the estimate based on the random‐effects model is shown in Figure 139 (SSBA2). Given the small number of studies, this result should be interpreted with caution. According to the Q‐test, there was no significant amount of heterogeneity in the true outcomes (Q(1)=0.48, p=0.49, ${\hat{\tau }}^{2}=0.00$, $I^{2}=0.00$%). With only two studies, moderator analyses were not possible and tests of publication bias are not valid. Neither of the studies was appraised as low risk of bias.

*Effects of safe spaces on women's increased freedom of movement and association*

**Figure 140:**
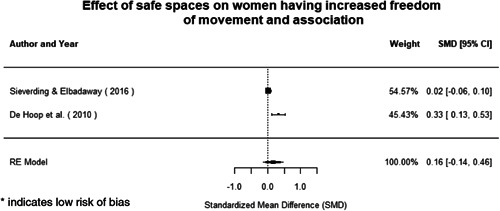
SSBA3: Forest plot showing the observed outcomes and the estimate of the random‐effects model. CI, confidence interval

Only two studies reported the impact of safe spaces on women's increased freedom of movement and association, thus we included *k *=* *2 studies in the analysis. We assessed one of the studies as having some concerns of risk of bias and the other as high risk of bias. The estimated average outcome based on the random‐effects model was $\hat{\mu }=0.16$ (95% CI: −0.14to0.29). This positive effect did not differ significantly from zero (z=1.05, p=0.29). A forest plot showing the observed outcomes and the estimate based on the random‐effects model is shown in Figure 140 (SSBA3). Given the small number of studies, this result should be interpreted with caution. According to the Q‐test, there was significant amount of heterogeneity in the true outcomes (Q(1)=7.73, p=0.01, ${\hat{\tau }}^{2}=0.04$, $I^{2}=87.07$%). With only two studies, moderator analyses were not possible and tests of publication bias are not valid. Neither of the studies was appraised as low risk of bias.

*Effects of safe spaces on women's attitudes toward taking action to claim their rights*

Mercy Corps' (2015) quasi‐experimental study in Niger was the only study evaluating the impact of safe spaces on women having more positive attitude towards taking action to claim their rights. Their report included two effects that fell into this outcome category (e.g., whether women feel in control). The effects were for slightly different interventions. For the Sawki program (safe spaces), there was a very small, positive point estimate (*g *=* *0.06, [95% CI: −0.10 to 0.22]), while for the Sawki program (safe spaces* *+* *livelihoods) the point estimate was a small and positive (*g *=* *0.12, [95% CI: −0.04 to 0.28]). We assessed the study as having high risk of bias.

*Effects of safe spaces on reduced percentage of women agreeing with certain reasons that justify violence against women and girls*

Sieverding and Elbadawy's (2016) quasi‐experimental study in Egypt was the only study evaluating the impact of safe spaces on reduced percentage of women agreeing with certain reasons that justify violence against women and girls. Their report included three effects that fell into this outcome category (e.g., an index asking when it is justified to beat a girl across different situations). The effects measured two outcomes in this category and at different periods after the intervention ended. The point estimates ranged from very small and negative (*g *=* *−0.03, [95% CI: −0.11 to 0.06]) to very small and positive (*g *=* *0.03, [95% CI: −0.07 to 0.13]). We assessed the study as having high risk of bias.

*Effects of safe spaces on women having better life skills*

**Figure 141:**
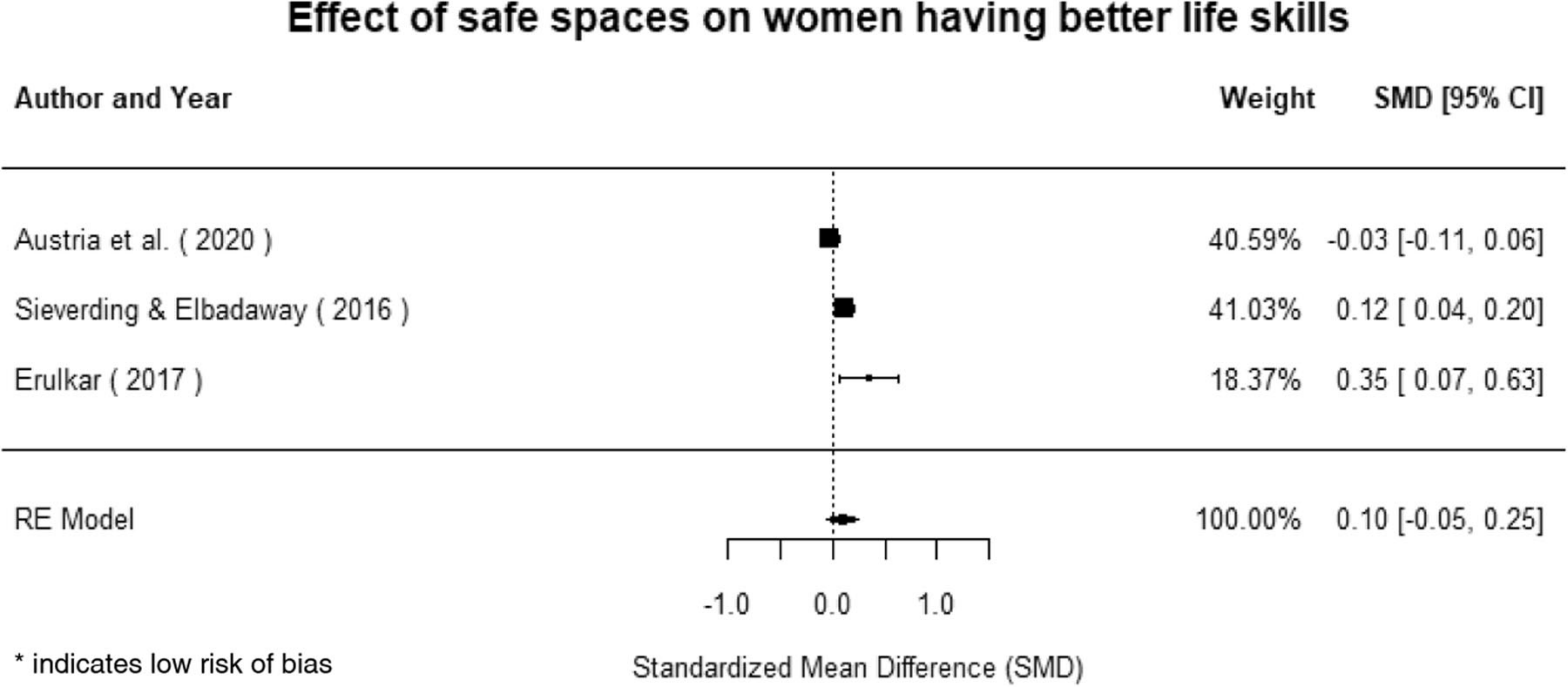
SSBA7: Forest plot showing the observed outcomes and the estimate of the random‐effects model. CI, confidence interval

We included a total of k=3 studies in the analysis. We assessed none of the studies as low risk of bias, two as some concerns, and one as high risk of bias. The observed outcomes ranged from −0.03 to 0.35. The estimated average outcome based on the random‐effects model was $\hat{\mu }=0.10$ (95% CI: −0.05 to 0.25). Therefore, the average outcome did not differ significantly from zero (z=1.32, p=0.19). A forest plot showing the observed outcomes and the estimate based on the random‐effects model is shown in Figure 141 (SSBA7).

According to the Q‐test, the true outcomes appear to be heterogeneous (Q(2)=9.87, p<0.01, ${\hat{\tau }}^{2}=0.01$, $I^{2}=79.74$%). An examination of the studentised residuals revealed that none of the studies had a value larger than ±2.39 and hence there was no indication of outliers in the context of this model. According to the Cook's distances, none of the studies could be considered to be overly influential. With only three studies, moderator analyses were not appropriate.

*Effects of safe spaces on reduced support for or occurrence of child and forced marriage*

**Figure 142:**
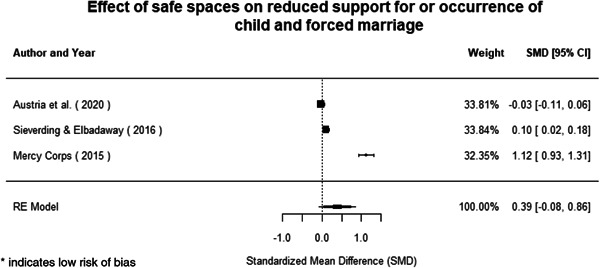
SSBA8: Forest plot showing the observed outcomes and the estimate of the random‐effects model. CI, confidence interval

We included a total of k=3 studies that examined the impact of safe spaces interventions on reduced support for or occurrence of child and forced marriage. We assessed none of the studies as low risk of bias, one as some concerns, and two as high risk of bias. The observed outcomes ranged from −0.03 to $1.12\hat{\mu }=0.39$. The estimated average outcome based on the random‐effects model was $\hat{\mu }=0.39\;$(95% CI: −0.08to0.86). Therefore, the average outcome did not differ significantly from zero (z=1.62, p=0.10). A forest plot showing the observed outcomes and the estimate based on the random‐effects model is shown in Figure 142 (SSBA8).

According to the Q‐test, the true outcomes appear to be heterogeneous (Q(2)=115.54, p<0.01, ${\hat{\tau }}^{2}=0.17$, $I^{2}=98.27$%). An examination of the studentised residuals revealed that one study (Mercy Corps, 2015) had a value larger than ±2.39 and may be a potential outlier in the context of this model. Indeed sensitivity analyses leaving each study out indicated that removing Mercy Corps (2015) would reduce the overall average effect (*μ*^ = 0.04 (95% CI: −0.09 to 0.16), but the effect would still be positive and nonsignificant (*z *=* *0.61, *p *=* *0.54). According to the Cook's distances, none of the studies could be overly influential. With only three studies, moderator analyses were not appropriate.

*Effects of safe spaces on increased participation in decision making by women at the household or community level*

**Figure 143:**
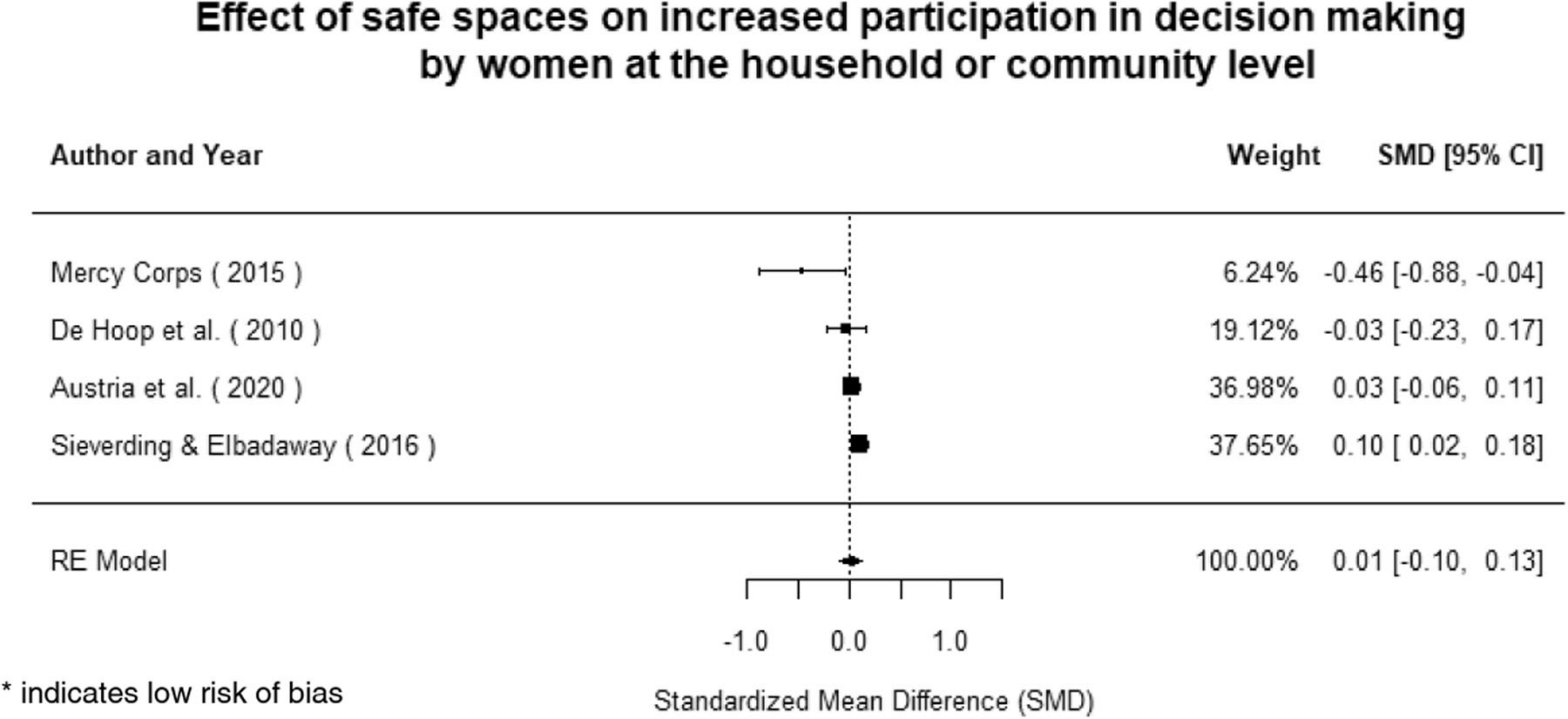
SSBB1: Forest plot showing the observed outcomes and the estimate of the random‐effects model. CI, confidence interval

We included a total of k=4 studies in the analysis. We assessed none of the studies as low risk of bias, two as some concerns, and two as high risk of bias. The observed outcomes ranged from −0.46 to 0.10. The estimated average outcome based on the random‐effects model was $\hat{\mu }=0.01$ (95% CI: −0.10 to 0.13). Therefore, the average outcome did not differ significantly from zero (z=0.24, p=0.81). A forest plot showing the observed outcomes and the estimate based on the random‐effects model is shown in Figure 143 (SSBB1).

According to the Q‐test, the true outcomes appear to be heterogeneous (Q(3)=7.96, p=0.047, ${\hat{\tau }}^{2}=0.01$, $I^{2}=62.29$%). An examination of the studentised residuals revealed that none of the studies had a value larger than ±2.50 and hence there was no indication of outliers in the context of this model. According to the Cook's distances, none of the studies could be overly influential.

Effects did not differ by risk of bias, publication year, or whether the study was examining post‐intervention changes or changes from baseline. We were unable to examine study design, exposure to intervention, evaluation period or whether the model was adjusted for covariates as potential sources of heterogeneity.

*Effects of safe spaces on women's participation in their community*

Sieverding and Elbadawy's (2016) quasi‐experimental study in Egypt was the only study evaluating the impact of safe spaces on women participating more in their community. There was small, and statistically significant, effect (*g *=* *0.12, [95% CI: 0.04 to 0.20]), but we assessed the study as having high risk of bias.

*Effects of safe spaces on prevention strategies to end violence against women and girls*

Sieverding and Elbadawy's (2016) quasi‐experimental study in Egypt was also the only study evaluating the impact of safe spaces on effective prevention strategies to end violence against women and girls. There was a small, and statistically significant, effect (*g *=* *0.12, [95% CI: 0.04 to 0.20]), and we assessed the study as having high risk of bias.

*Effects of safe spaces on whether relief and recovery initiatives in conflict and post conflict situations respond to the needs of women and girls*

Mercy Corps' (2015) quasi‐experimental study in Niger was the only study evaluating the impact of safe spaces on whether relief and recovery initiatives in conflict and post conflict situations respond to the needs of women and girls, especially vulnerable groups. There was a small, but not statistically significant, point estimate (*g *=* *−0.11, [95% CI: −0.27 to 0.05]), and we assessed the study as having high risk of bias.

*Effects of safe spaces on whether communities have a more positive attitude towards women/marginalised groups*

Sieverding and Elbadawy's (2016) quasi‐experimental study in Egypt was the only study evaluating the impact of safe spaces on whether communities have a more positive attitude towards women/marginalised groups. There was a very small, but not statistically significant, point estimate (*g *=* *−0.02, [95% CI: −0.10 to 0.06]), and we assessed the study as having high risk of bias.

*Effects of safe spaces on women having improved attitudes, self‐image and confidence*

**Figure 144:**
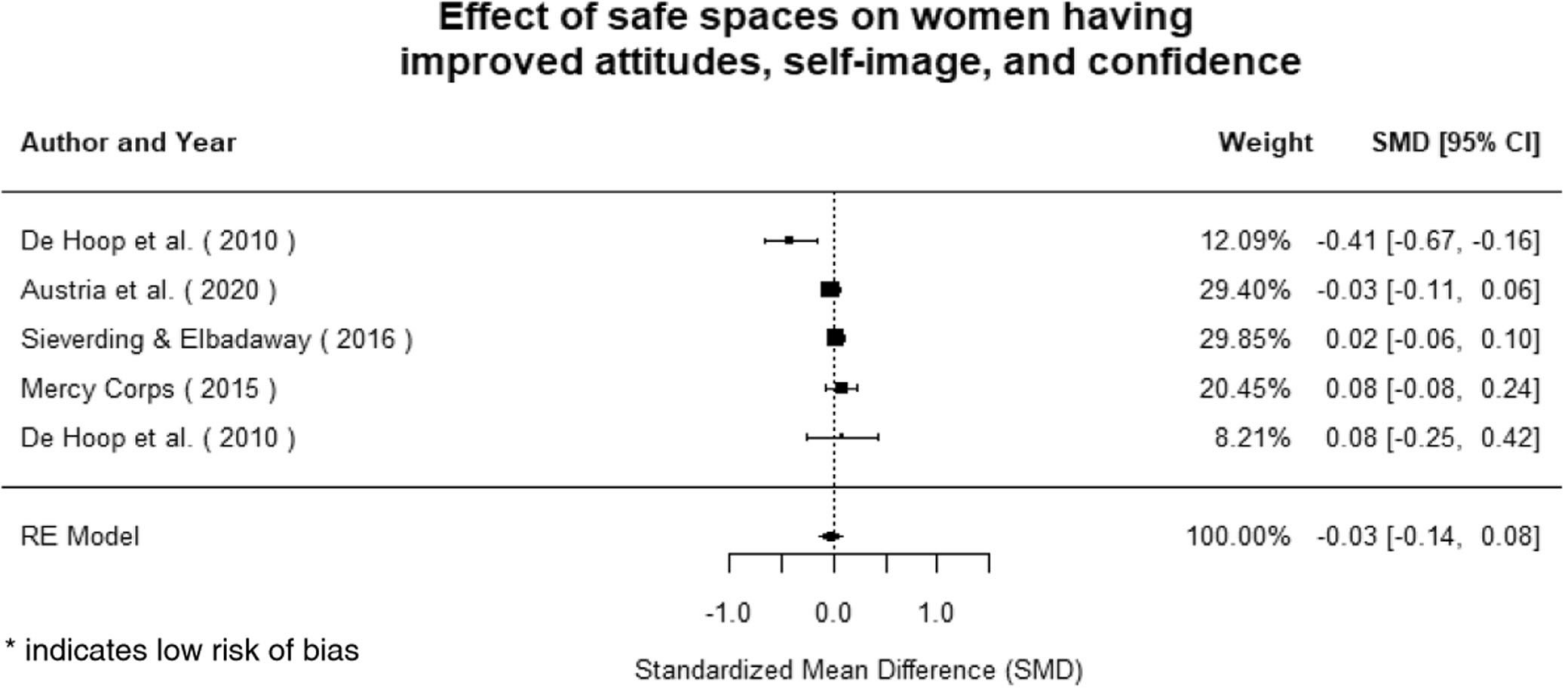
SSCB3: Forest plot showing the observed outcomes and the estimate of the random‐effects model. CI, confidence interval

We included a total of k=5 studies in the analysis. We assessed none of the studies as low risk of bias, two as some concerns, and two as high risk of bias. De Hoop and colleagues (2010) contribute two effects from two independent samples, one sample of participants from villages with conservative social norms (*g *=* *0.41), and the other from participants in villages with liberal social norms (*g *=* *0.08). The observed outcomes ranged from −0.41 to 0.08. The estimated average outcome based on the random‐effects model was $\hat{\mu }=-0.03$ (95% CI: −0.14 to 0.08). Therefore, the average outcome did not differ significantly from zero (z=−0.52, p=0.61). A forest plot showing the observed outcomes and the estimate based on the random‐effects model is shown in Figure 144 (SSCB3).

According to the Q‐test, the true outcomes appear to be heterogeneous (Q(4)=11.52, p=0.02, ${\hat{\tau }}^{2}=0.01$, $I^{2}=65.27$%). An examination of the studentised residuals revealed that one study (De Hoop et al., 2010) had a value larger than ±2.58 and may be a potential outlier in the context of this model. According to the Cook's distances, none of the studies could be overly influential.

Effects were not moderated by risk of bias, publication year, or whether the study was examining post‐intervention changes versus changes from baseline. We were unable to test moderation by study design, exposure to the intervention, evaluation period, whether the model was adjusted for covariates.

*Effects of safe spaces on attitudes and support for women's economic, social and human rights*

Sieverding and Elbadawy's (2016) quasi‐experimental study in Egypt was the only study evaluating the impact of safe spaces on improved attitudes and increased support for women's economic, social and human rights by men, household and family members and community members. Their report included two effects that fell into this outcome category (e.g., whether the mother was pro‐sports for girls). The effects ranged from very small, positive point estimates (*g *=* *0.02, 95% CI [−0.06, 0.10]) to very small, positive point estimates (*g *=* *0.02, 95% CI [−0.06, 0.10]). We assessed the study as having high risk of bias.

*Effects of safe spaces on safer and more secure household, communities and areas/territories for women, girls, men and boys*

Mercy Corps' (2015) quasi‐experimental study in Niger was the only study evaluating the impact of safe spaces on safer and more secure household, communities and areas/territories for women, girls, men, and boys. Their report included six effects that fell into this outcome category (e.g., someone to rely on when you have sensitive problems). The effects examined three outcomes over different time periods and slightly different versions of the intervention (savings plus vs savings only). The effects ranged from very small, negative point estimates (*g *=* *−0.02, [95% CI: −0.18 to 0.15]) to medium, positive effects (*g *=* *0.27, [95% CI: 0.12 to 0.43]). We assessed the study as having high risk of bias.

*Effects of safe spaces on reduced frequency and distribution of types of violence by an intimate partner*

Buehren, Goldstein, et al.'s (2017) experimental study in Tanzania was the only study evaluating the impact of safe spaces on reduced frequency and distribution of types of violence by an intimate partner. Their report included 2 effects that fell into this outcome category (e.g., ever had sex unwillingly). The study reported effects for slightly different versions of the intervention. The point estimate for the Club Only intervention was very small and negative (*g *=* *−0.06, [95% CI: −0.13 to 0.01]) and the point estimate for the Club + Microfinance intervention was very small and positive (*g *=* *0.00, [95% CI: −0.07 to 0.07]). We assessed the study as having some risk of bias concerns.

*Effects of safe spaces on improved quality of relationships between women and their household and community members*

**Figure 145:**
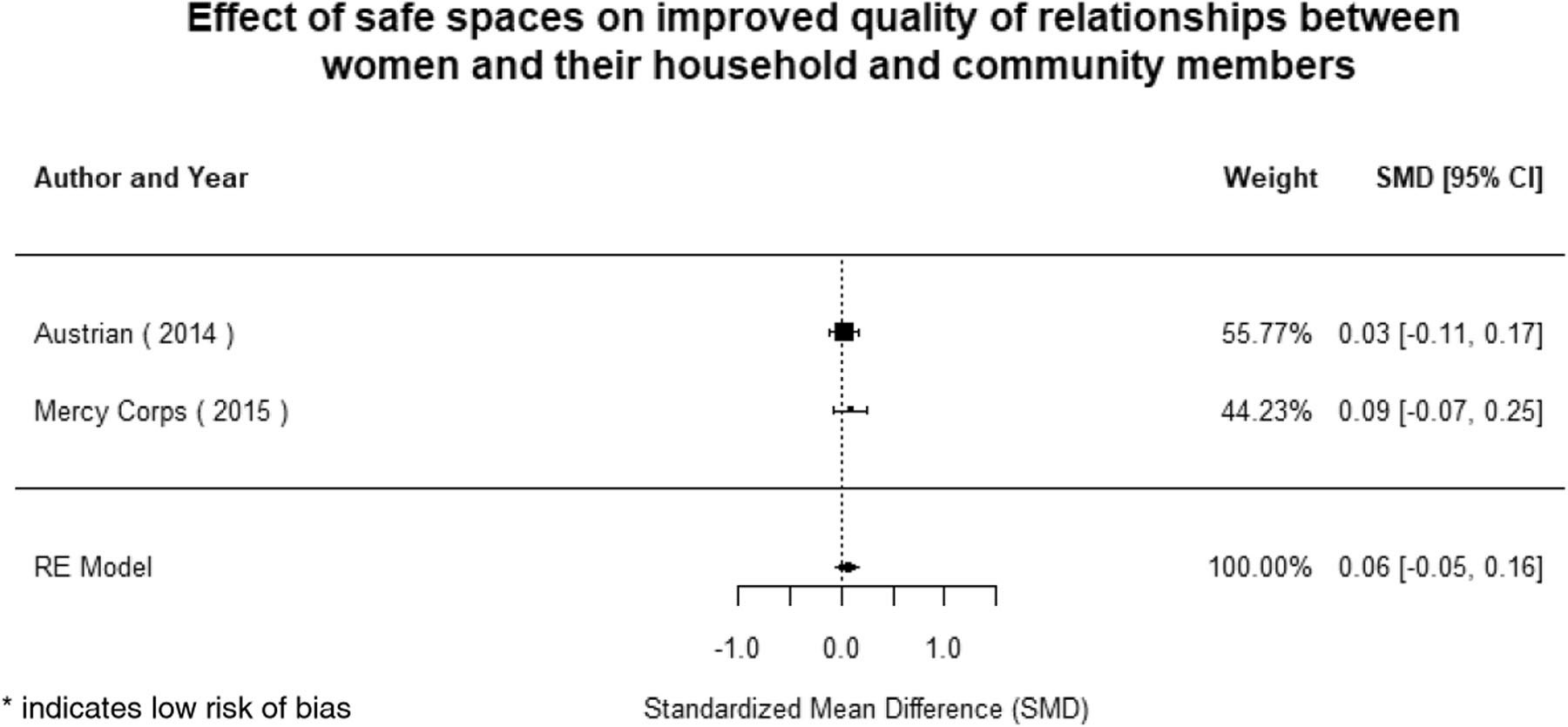
SSCB8. Forest plot showing the observed outcomes and the estimate of the random‐effects model. CI, confidence interval

Only two studies reported the impact of safe spaces on improved quality of relationships between women and their household and community members, thus we included *k *=* *2 studies in the analysis. We assessed both studies as high risk of bias. The estimated average outcome based on the random‐effects model was $\hat{\mu }=0.06$ (95% CI: −0.05to0.16). This positive effect did not differ significantly from zero (z=1.02, p=0.31). A forest plot showing the observed outcomes and the estimate based on the random‐effects model is shown in Figure 145 (SSCB8). Given the small number of studies, this result should be interpreted with caution. According to the Q‐test, there was no significant amount of heterogeneity in the true outcomes (Q(1)=0.28, p=0.59, ${\hat{\tau }}^{2}=0.00$, $I^{2}=0.00$%). With only two studies, moderator analyses were not possible and tests of publication bias are not valid. Neither of the studies was appraised as low risk of bias.

*Qualitative findings*

We conducted a thematic synthesis on the four linked qualitative studies to the included safe spaces interventions. As indicated above, this thematic synthesis aims to identify themes related to the interplay of intervention design, intervention implementation, target population, and contextual variables with intervention outcomes and effects. In total, we identified 11 descriptive themes, which we configured into three analytical themes (Supporting Information Appendix SN). These three analytical themes present the synthesis results and are discussed in more detail below.

*Theme 1: Safe space intervention design benefits from targeting girls before reaching the contextually eligible age for marriage and parental awareness raising initiatives to encourage uptake*.

Cultural and gender practices can predispose girls to early forced marriages and teenage pregnancy. The qualitative evidence suggests to target girls before they reach their teens or ages that are culturally considered eligible for marriage. The Ishraq programme in Egypt targeted girls 13–15 years of age, which was reported as a factor that may have contributed to programme impacts (Ringler, 2009) in a community where any girl above 15 years of age was considered for marriage (e.g., through traditional rites ceremonies to prepare teenagers). One community leader from Zambia's (AGEP) claimed: ‘They do not value education and especially girls, so girls are married off at a very tender age… we have heard that within the circles, a girl can be married at actually 3 years. 4 years, and the man keeps waiting for that girl, and then when they reach puberty, they just get that girl. So, it is very, very common in this community… Community challenges… religious beliefs, where elderly men marry off young girls… we really need to find some interventions to get to them’ (Duby et al., 2016).

Negative perceptions about programme intentions and debates around childrens’ rights can impede programme uptake, andomized the need for programme design to incorporate appropriate awareness and communication strategies. For example, a mentor in the AGEP programme in Zambia discussed religious sentiments preventing participation: ‘The first thing is the parents, since inception of the programme it was difficult even the time, we were distributing letters some parents used to refuse, for those that understood what we were explaining are the ones that came but some didn't and they never showed up, because they had already told their minds that we are Satanists. There was a lot of influence going on among the members of the community that led to most of the girls dropping out. Due to the rumour of us being Satanists’. A parent in the same context in an interview complained about human rights for their children: ‘Human rights has to be removed because a parent has to reinforce discipline in the children and sometimes parents are blamed of which it's not their fault but because of this human rights thing’ (Duby et al., 2016).

*Theme 2: Mentorship appears to be a key component of the intervention design and delivery*.

Safe spaces interventions are a forum where interactions geared towards changing participants’ beliefs and attitudes take place. Mentors are seen to play a crucial role in encouraging collaboration, interaction, learning from each other and bringing about positive attitudes and knowledge, which often cover issues beyond what is learnt through formal education. Safe spaces intervention and relevant group‐based activities benefit from the role mentors play in this process. Mentors’ personality, passion and purpose for the cause of the girls, as well as their capacity development in the areas of advocacy, outreach, communication and networking skills, support winning the confidence of parents to permit their children to participate.

The case of the Ishraq programme in Egypt, andomized the role that mentors play in building trusted relations to support programme uptake: ‘One of the most effective recruitment strategies was the promoters’ home visits. Promoters met with families of eligible girls to describe the programme, what was expected from girls and their families and the valuable opportunity it represented for their daughters. Because of Ishraq's novelty and the community's entrenched social norms, more than one home visit often was needed to secure a girl's participation. By understanding more about who the promoters were and gaining confidence and trust in them, parents began to feel increasingly comfortable with the programme’ (Brady et al., 2007).

Through mentorship, girls may open up on matters they may not be able to share, even with their own parents. One of the participants of the AGEP in Zambia said: ‘It has definitely changed my confidence… at the Safe Spaces we are instilled with so much self‐esteem, even if someone annoyed you at home when you get to the Safe Spaces you feel better… when you get there your mentor will start to give you different lessons in the process, she will make you laugh about something or put you on the right path’ (Duby et al., 2016). And about the safe spaces programme in Niger, this was reported, ‘…*mentors, usually an iya (traditional mentor), are central to the success of safe space objectives. While this finding needs to be further investigated, it also tells us that programs andomize safe spaces must invest in training and coaching their mentors to support girls and effectively deliver content*’ (Mercy Corps, 2015).

*Theme 3: Provision of a suitable, accessible and safe location for meetings and as well as sufficient incentives encourage participation*.

While safe spaces provide a forum where girls and women can openly discuss private issues that may feel unsafe to discuss publicly, it is important that meeting places also be physically safe. Meeting places require deliberate selection andomized safe accessibility. When meeting places are either too far from the reach of participants or located in dangerous locations, they are not suitable undermining the willingness of participants to attend and focus on programme activities, te. A mentor in the AGEP in Zambia reflected on this issue: ‘We had a challenge with where we were meeting, we were not safe. At first, we were just meeting outside so there was no concentration, they (group members) were not participating, they were just looking around and doing other things. When you are in an enclosure, they concentrate…’ (Duby et al., 2016).

Incentives and rewards are a promising strategy to encourage participation of the targeted population in programmes. However, the design of both needs to take into account a number of reported factors that can undermine their implementation: (i) where incentives are not provided as and when due; (ii) when the conditions tied to the award of rewards are too stringent for participants to fulfil; and (iii) when incentives become the sole reason for participation. These factors can lead to complaints from participants, resulting in an additional burden for mentors or implementers of the programme. One of the mentors on the AGEP in Zambia commented about the challenge that incentives constituted to the programme this way: ‘What used to discourage me is the issue to do with the prizes, they used to give me some trouble and they caused a lot of children to stop coming because they used to say a child can only get a prize if they have been attending the meetings continuously for ten weeks but you would find that some of them would miss some meetings and then when they come they find that their friends are being given prizes you find that they go back and never come back and secondly instead of giving the children the prizes at the time that they promised they would give them, they would delay, it used to take a long time and they would even start asking to say madam, when will we get the prizes, madam, we have been attending the meetings for the past so many weeks, when will we get our things? That used to discourage us, it used to be a challenge for us mentors… Most of them stopped because some of the children were coming for the meetings to get the prizes not necessarily to learn’ (Duby et al., 2016).

*Discussion*

Quantitative meta‐analysis revealed that safe spaces interventions had positive significant effects on women's increased access to and ownership of assets, credit, and income. Although small positive effects were found in terms of increases capacity to understand financial services, increases mobility, control over body, marriage and sexual health, better life skills, participation in household/community level decision making and quality of relationship with household or community members, none of these were statistically significant. The intervention group was only covered by [4] studies and the associated GRADE analysis showed low confidence about the quality of the evidence. A complementary qualitative analysis was carried out to put forward a set of recommendations that may help design more effective safe spaces interventions.

An explanation to the limited effects might be the fact that many of the outcomes involved changes in long‐standing social and cultural norms and attitudes towards women or girls, and it may need a longer period to observe visible changes. In view of this, awareness building, at both household and community levels, were found to be a key design element. Working only with target participants may limit the potential for such changes if the household/community members are not ready to change their attitudes. This supports the idea of pre‐intervention research to design the best intervention mechanism to suit the settings.

Targeting the appropriate demographic groups was found to be a further crucial factor. With regard to bringing about positive changes in outcomes related to marriage and maternal health, control over body and sexual health, it is important to work with unmarried young girls and women. Awareness building among participants as well as their household members and the wider community at an early stage of the intervention plays a critical role as many of such outcomes were compounded by social and cultural norms and traditions.

As mentioned earlier, quantitative analysis showed small positive yet insignificant effects of safe spaces interventions on women's/girls’ control over their bodies, knowledge about sexual health, better life skills and freedom of movement and association. These outcomes are often conveyed through collaborative learning and participation in group activities and mentorship provided via safe spaces interventions was found as an important driver in view of this. Mentors play a crucial role in encouraging collaboration, interaction, learning from each other and bringing about positive attitudes and knowledge. Mentors served as trustworthy source to whom participants could turn when in need. Parents would often feel comfortable to let their daughters participate in outdoor activities if accompanied by mentors.

Positive changes in women's and girls’ lives expected from safe spaces interventions critically hinges on participation in activities such as meetings, trainings, and group learning/activities. The qualitative synthesis revealed several concerns hindering women's/girls’ participation which might have resulted in the statistically insignificant effects found in the quantitative analysis. Qualitative evidence found that distance to the safe spaces meeting location, timing of the meetings, availability of the meeting venues during the scheduled meeting times were among key challenges limiting participation. The physical facility where safe spaces meetings and activities would be carried out needs to be safe and suitable for participants. These operational issues need to be considered during design.

##### 5.6.2.4 Summary of findings and discussion

We included six studies in six countries in MENA and Sub‐Saharan Africa that evaluated the effect of safe spaces. While many outcome categories had sufficient evidence for quantitative synthesis, only one outcome showed a significant impact of safe spaces. Safe spaces interventions had positive significant effects on women's increased access to and ownership of assets, credit, and income. Though small positive effects were found for increased capacity to understand financial services, increased control over body and sexual health, increased freedom of movement and association, better life skills, reduced support for or instance of child and forced marriage, increased participation in household/community level decision making and quality of relationship with household or community members, none of these were statistically significant. There was also a negative but nonsignificant impact on improved attitudes/self‐image/confidence. While the general body of evidence did not allow for testing of moderation in most instances, we did find that exposure to intervention was a source of heterogeneity for women's access to and ownership of assets, credit and income such that longer exposure periods were related to smaller impacts. This is likely due to the one study with the longest exposure period having the smallest effect. Overall, the GRADE assessments indicate a very low to low certainty in this body of evidence.

The quantitative findings are buttressed by three qualitative themes from the four qualitative linked studies. Overall, targeting is crucial for such interventions to improve the uptake and potential for long‐lasting effects. By focusing specific safe spaces interventions for adolescent girls, forced marriage could potentially be reduced by the package of activities offered in such a space. Physical space as well as planned activities plays a crucial role in maintaining uptake and the perceived safety of participants and implementing partners. Lastly, awareness raising with parents (if adolescent girls are participating) or household members can prove beneficial to participation and uptake of the interventions offered in safe spaces. The summary of our quantitative findings along with the GRADE certainty of evidence ratings are presented in Table 54 (Table 49).

**Table 49:** GRADE summary of findings and certainty of evidence on safe spaces

Certainty assessment							Sample size	Effect	Certainty	Importance
No. of studies	Study design	Risk of bias	Inconsistency	Indirectness	Imprecision	Other considerations		Absolute (95% CI)		
*(AB1) Increased capacity of women to understand and use financial, banking, and business services effectively*										
4	RCT‐2 QED‐2	Very serious^a^	Not serious	Not serious	Not serious	None	8679	SMD 0.05 SD higher (0.03 lower to 0.13 higher)	⊕⊕◯◯ LOW	Important, but not critical
*(AB2) Women have increased access and ownership to assets, credit and income*										
4	RCT‐2 QED‐2	Very serious^b^	Serious^c^	Not serious	Not serious	Publication bias strongly suspected	8742	SMD 0.14 SD higher (0.05 higher to 0.23 higher)	⊕◯◯◯ VERY LOW	Important, but not critical
*(AC3) Improved capacity of women entrepreneurs*										
1	RCT	Serious^d^	Serious^e^	Not serious	Not serious	None	3179	Two positive effect estimates with a 95% CI of −0.05 to 0.09	⊕⊕◯◯ LOW	Important, but not critical
*(BA2) Women have more and better control over their bodies and sexual health*										
2	RCT‐1 QED‐1	Very serious^f^	Serious^g^	Not serious	Serious^h^	None	4226	SMD 0.1 SD higher (0.05 lower to 0.25 higher)	⊕◯◯◯ VERY LOW	Critical
*(BA3) Women have increased freedom of movement and association*										
2	QED‐2	Very serious^i^	Very serious^j^	Not serious	Serious^k^	None	2635	SMD 0.02 SD higher (0.07 lower to 0.1 higher)	⊕◯◯◯ VERY LOW	Limited importance
*(BA5) Women have more positive attitude towards taking action to claim their rights*										
1	QED	Very serious^l^	Serious^e^	Not serious	Not serious	None	615	Two positive effect estimates with a 95% CI of −0.10 to 0.28	⊕◯◯◯ VERY LOW	Limited importance
*(BA6) Reduced percentage of women agreeing with certain reasons that justify violence against women and girls*										
1	RCT	Very serious^m^	Serious^e^	Not serious	Not serious	None	732	Two positive and one negative effect estimate with a 95% CI range of −0.11 to 0.13	⊕◯◯◯ VERY LOW	Critical
*(BA7) Women are equipped with better life skills that allow them to be prepared for crisis or shocks and recover from them*										
3	RCT‐1 QED‐2	Very serious^n^	Very serious^o^	Not serious	Serious^p^	None	6752	SMD 0.1 SD higher (0.05 lower to 0.25 higher)	⊕◯◯◯ VERY LOW	Important, but not critical
*(BA8) Reduced instances of child or forced marriage*										
3	RCT‐1 QED‐2	Very serious^q^	Very serious^r^	Not serious	Serious^s^	Publication bias strongly suspected strong association	6823	SMD 0.39 SD higher (0.08 lower to 0.86 higher)	⊕◯◯◯ VERY LOW	Critical
*(BB1) Increased participation in decision making by Women at the household or community level, including during crisis response*										
4	RCT‐1 QED‐3	Very serious^t^	Very serious^u^	Not serious	Serious^v^	Publication bias strongly suspected	6842	SMD 0.01 SD higher (0.1 lower to 0.13 higher)	⊕◯◯◯ VERY LOW	Limited importance
*(BB2) Women participate more in their community*										
1	QED	Very serious^w^	Serious^e^	Not serious	Not serious	None	2248	SMD 0.12 SD higher (0.04 higher to 0.2 higher)	⊕◯◯◯ VERY LOW	Limited importance
*(CA6) Effective prevention strategies supported to end violence against women and girls*										
1	QED	Very serious^x^	Serious^e^	Not serious	Not serious	None	2248	SMD 0.12 SD higher (0.04 higher to 0.2 higher)	⊕◯◯◯ VERY LOW	Critical
*(CA8) Increased community support for women's and children's human, economic and legal rights*										
1	QED	Very serious^y^	Serious^e^	Not serious	Serious^z^	None	594	SMD 0.11 SD lower (0.27 lower to 0.05 higher)	⊕◯◯◯ VERY LOW	Important, but not critical
*(CB2) Communities have a more positive attitude towards women/andomizedi groups*										
1	QED	Very serious^aa^	Serious^e^	Not serious	Not serious	None	2248	SMD 0.02 SD lower (0.1 lower to 0.06 higher)	⊕◯◯◯ VERY LOW	Limited importance
*(CB3) Women have improved attitudes, self‐image and confidence*										
4	RCT‐1 QED‐3	Very serious^ab^	Very serious^ac^	Not serious	Serious^ad^	None	7567	SMD 0.03 SD lower (0.14 lower to 0.08 higher)	⊕◯◯◯ VERY LOW	Limited importance
*(CB4) Improved attitudes and increased support for women's economic, social and human rights by men, household and family members and community members*										
1	QED	Very serious^ae^	Serious^e^	Not serious	Not serious	None	2248	Two positive studies with identical Cohen's *d*: 0.02 (−0.06, 0.10)	⊕◯◯◯ VERY LOW	Limited importance
*(CB6) Safer and more secure household, communities and areas/territories for women, girls, men and boys (54,258,398)*										
1	QED	Very serious^af^	Serious^e^	Not serious	Serious^ag^	None	615	One negative and one positive effect estimate with a 95% CI range of −0.18 to 0.21	⊕◯◯◯ VERY LOW	Critical
*(CB6) Safer and more secure household, communities and areas/territories for women, girls, men and boys (54,977,131)*										
1	QED	Very serious^ah^	Serious^e^	Not serious	Not serious	None	762	Four positive effect estimates with a 95% CI range of −0.07 to 0.43	⊕◯◯◯ VERY LOW	Critical
*(CB7) Reduced frequency and distribution of types of violence by an intimate partner*										
1	RCT	Serious^ai^	Serious^e^	Not serious	Not serious	None	3179	One positive and one negative effect estimate with a 95% CI range of −0.05 to 0.00	⊕⊕◯◯ LOW	Critical
*(CB8) Improved quality of relationships between women and their household and community members*										
2	QED‐2	Very serious^aj^	Not serious	Not serious	Not serious	None	1993	SMD 0.06 SD higher (0.05 lower to 0.16 higher)	⊕⊕◯◯ LOW	Limited importance

Abbreviations: CI, confidence interval; GRADE, Grading of Recommendations, Assessment, Development and Evaluations; QED, quasi‐experimental design; RCT, andomized controlled trial; SMD, andomizedi mean difference.

^a^ Downgraded twice because all studies present risk of bias and two are of high risk of bias, including selection bias and deviation from intended intervention.

^b^ Downgraded to very serious because none of the studies are rated as low bias. When high ROB studies are removed, summary effect changes from 0.14 and significant to 0.09 and nonsignificant.

^c^ Downgraded once because point estimates vary considerably and there are Cis that do not overlap.

^d^ Downgraded because of uncertainty related to outcome reporting bias.

^e^ All single studies downgraded once for inconsistency.

^f^ Downgraded two times because: (1) both studies present at least some concerns related to risk of bias and one is of high risk, and (2) removing either study would have an impact on the meta‐analysis.

^g^ Downgraded once: (1) variation of points estimates: data on both side of the threshold, (2) overlaps of CI: systematic overlap, (3) $I^{2}=0\%$, (4) test of stat: According to the *Q*‐test, there was no significant amount of heterogeneity in the true outcomes.

^h^ Downgrade once as *p*‐value of 0.66 and sample size >400.

^i^ Downgraded twice because: (1) both studies present at least some concerns related to risk of bias and one is of high risk, and (2) removing either study would have an impact on the meta‐analysis.

^j^ Downgraded twice: (1) variation of points estimates: data on both side of the threshold, (2) overlaps of CI: no systematic overlap, (3) $I^{2}=87.07\%$, (4) test of stat: According to the *Q*‐test, there was significant amount of heterogeneity in the true outcomes.

^k^ Downgrade once as *p*‐value of 0.29 and sample size >400.

^l^ Downgraded because of uncertainty across several criteria and a high risk of spill‐over/contamination.

^m^ Downgraded because of uncertainty across several criteria and a high risk of spill‐over/contamination and reporting bias.

^n^ Downgraded twice because (1) all studies in this group present risk of bias, and one study is of high risk, and (2) the high risk of bias studies make up >30% of the weight of the meta‐analysis.

^o^ Downgraded twice: (1) variation of points estimates: data on both side of the threshold, (2) overlaps of CI: no systematic overlap, (3) $I^{2}=79.74\%$, (4) Test of stat: According to the *Q*‐test, the true outcomes appear to be heterogeneous.

^p^ downgrade once as *p*‐value of 0.19 and sample size >400.

^q^ Downgraded twice because: (1) One raises some concerns related to risk of bias and (2) high risk including high risk on 3 domains, (3) removing either study would have an impact on the meta‐analysis.

^r^ Downgraded twice: (1) Variation of points estimates: data on both side of the threshold, (2) overlaps of CI: no systematic overlap with one study CI very far from the two others, (3) $I^{2}=98.27\%$, (4) test of stat: According to the *Q*‐test, the true outcomes appear to be heterogeneous.

^s^ downgrade once as *p*‐value of 0.10 and sample size >400.

^t^ Downgraded twice because half of the body of evidence come from high risk of bias studies, and the rest from studies of medium or uncertain bias. Additionally, three of four of the studies are QEDs, including two high risk.

^u^ Downgraded twice: (1) variation of points estimates: data on both side of the threshold, (2) overlaps of CI: no systematic overlap with one study CI very far from the two others, (3) $I^{2}=62.29\%$, (4) test of stat: According to the *Q*‐test, the true outcomes appear to be heterogeneous.

^v^ downgrade once as *p*‐value of 0.81 and sample size >400.

^w^ Downgraded because of uncertainty across several criteria and a high risk of spill‐over/contamination and reporting bias.

^x^ Downgraded because of uncertainty across several criteria and a high risk of spill‐over/contamination and reporting bias.

^y^ Downgraded because of uncertainty across several criteria and a high risk of spill‐over/contamination.

^z^ Downgraded once because of wide Cis crossing the threshold of interest.

^aa^ Downgraded because of uncertainty across several criteria and a high risk of spill‐over/contamination and reporting bias.

^ab^ Downgraded twice because half the body of evidence comes from high risk of bias QEDs. Furthermore, removing either high RoB studies impacts the results of the meta‐analysis

^ac^ Downgraded twice: (1) variation of points estimates: data on both side of the threshold, (2) overlaps of CI: no systematic overlap with one study CI very far from the two others, (3) $I^{2}=65.27\%$, (4) test of stat: According to the *Q*‐test, the true outcomes appear to be heterogeneous.

^ad^ downgrade once as *p*‐value of 0.61 and sample size >400.

^ae^ Downgraded because of uncertainty across several criteria and a high risk of spill‐over/contamination and reporting bias.

^af^ Downgraded because of uncertainty across several criteria and a high risk of spill‐over/contamination.

^ag^ Downgraded because the point estimates are quite broad and the range of CI is widely across both sides of the threshold.

^ah^ Downgraded due to uncertainty across several criteria including performance bias and outcome measurement bias.

^ai^ Downgraded because of uncertainty related to outcome reporting bias.

^aj^ Downgraded to very serious because both included studies are of high risk of bias.

#### 5.6.3 Life, social and livelihood skills and capacity building

Life, social and livelihood skills and capacity building include the process of changing attitudes and behaviours by imparting knowledge and developing skills while andomized the benefits of participation, knowledge exchange and ownership (Di Pierro, 2021). Training and capacity development encompass vocation and technical skills, cognitive and noncognitive skills, life skills and so on.

##### 5.6.3.1 How do life, social and livelihood skills and capacity building affect gender equality, women's empowerment and Peace outcomes?

**Figure 146:**
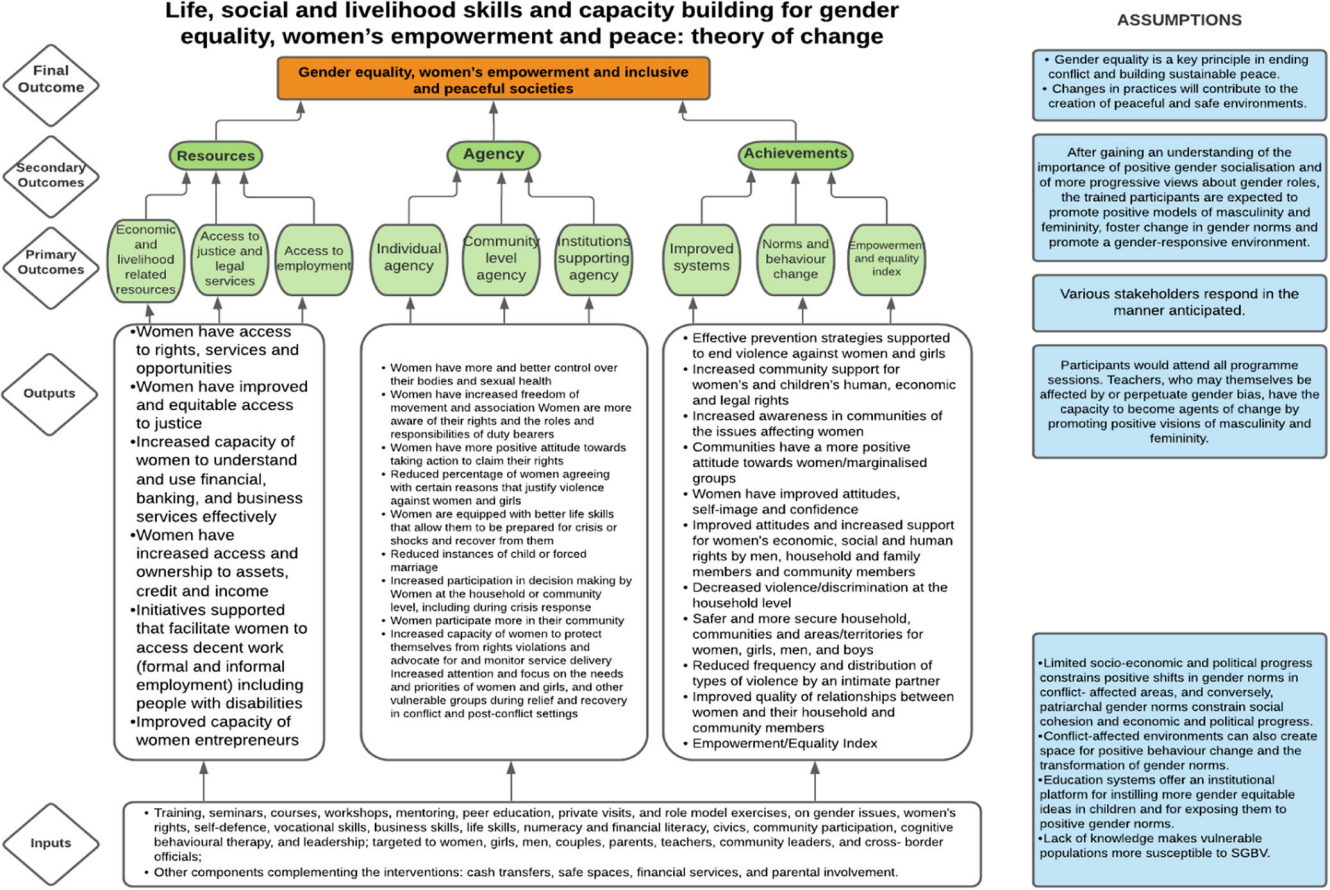
Life, social and livelihood skills and capacity building for gender equality, women's empowerment and peace: Theory of change

Figure 146 maps the causal chain of how life, social and livelihood skills and capacity building may improve gender equality, women's empowerment and peace outcomes.

Capacity building interventions and training in life, social and livelihood skills programmes aim to empower women through increasing their access to income generating activities through decent work and self‐employment, assets, and services; the control over their bodies and sexual health; and their freedom of movement and association. Equipping women with better life skills should be translated into more participation in decision making in their households and in their communities, reduced violence against women, and improved attitudes, self‐image and confidence to take action and claim their rights. To achieve this, both formal and informal platforms, seminars, courses, workshops, mentoring, peer education and other forms of training can be complemented with cash transfers, safe spaces, financial services and parental involvement. Assumptions supporting this causal path include the idea that lack of knowledge is a barrier for women to have more resources, agency and achievements, where training and education interventions may be effective in overcoming this barrier. Further, the educational system is assumed to be a platform where not only more equitable gender norms can be installed, but also where teachers can promote and set an example of gender equality. Equality is supposed to promote the end of conflict and build sustainable peace and safe environments.

##### 5.6.3.2 Description of included studies

We included 20 studies reported in 26 different papers that evaluated the effect of 12 identified programmes. We included more than one paper that evaluated the same programme if the author(s) undertook different analyses or reported different outcomes over several papers.

*Population*

Most studies targeted adolescent girls aged between 10 and 19 years old; most were interventions implemented alongside safe spaces and including parental involvement. Interventions working with adults, primarily aimed to promote egalitarian decision making within households, targeted married or cohabiting couples. Other populations targeted were teachers, small‐scale coffee farmers, bank clients, micro entrepreneurs, and community members.

The included studies evaluated 12 different programmes and trials covered countries in South Asia and Sub‐Saharan Africa and 13 countries including: Afghanistan, Bangladesh, India, DRC, Ethiopia, Liberia, Malawi, Mali, Sierra Leone, South Sudan, Tanzania, Uganda, Zambia.

*Intervention, inputs and activities*

The included studies evaluated a range of different activities and inputs including:

- Life skills sessions, mentoring, parental involvement and safe spaces (*n* = 8): Life skills sessions covered content ranging from decision‐making and disagreement resolution, to reproductive health, puberty, gender norms, cultural pressures, healthy relationships, life goals and violence prevention, while parent/caregiver discussion groups andomi on positive relationship building, empathetic communication, non‐violent discipline methods and specific developmental and cultural issues experienced by adolescent girls.
- Skills building courses (*n* = 7): Using peer education, participatory courses, or afterschool programmes, to train girls on topics ranging from informal negotiation, budgeting, financial literacy, business planning, and savings, to social skills, cognitive behavioural therapy skills, self‐defence, leadership and advocacy skills.
- Vocational training plus resource provision (*n* = 1): Skill building in numeracy and chosen vocational skill (e.g., sewing) together with foundational training in modules that included: the value of women's work, ways to save money, ways to earn income and improve income‐generating activities, basic business skills, ways to improve health and well‐being, women's rights and prevention of SGBV, strategies to make decisions and negotiate, civic action and advocacy, social networks and safety nets. Resources provision in the form of monthly cash stipend, mechanisms to save money, and referrals to health, legal and financial services.
- Men's and couples’ seminars and courses (*n* = 5): Delivered either at a local established ceremony for community discussion, or through workshops, covering topics such as division of roles, responsibilities, decision‐making power and access to resources in their households, attitudes towards and treatment of women. With or without complementary activities such as gender dialogues with community leaders and women's self‐help groups.
- Training teachers as agents of change for promoting gender norms and equality (*n* = 1): three training sessions plus reinforcing text messages on gender, conflict and peacebuilding.
- Training for cross‐border officials on andomized against SGBV (*n* = 1): andomizedizedion of officials to reduce harassment of traders and improve conditions for small‐scale cross‐border trade; empowerment of small‐scale traders; and support for policy dialogue and improved coordination among local actors, with a set of joint workshops held between traders and officials.
- Civics course on community participation in governance (*n* = 1): A civics course providing information about the rights and responsibility of citizens in the democratic process (Table 50).

**Table 50:** Life skills and capacity building activities design features of included studies

Study	Activity/input	Length of treatment	Intervention frequency
Tanner and O'Connor (2017a, 2017b)	Life skills sessions, mentoring, parental involvement and safe spaces	9	Weekly
Tanner and O'Connor (2017a, 2017b)	Life skills sessions, mentoring, parental involvement and safe spaces	9	Weekly
Tanner and O'Connor (2017a, 2017b)	Life skills sessions, mentoring, parental involvement and safe spaces	9	Weekly
Buehren, Chakravarty, et al. (2017)	Life skills and livelihoods training combined with small capital to help turn training into an income generating activity and courses on financial literacy and budgeting skills	24	Unclear
Stark, Asghar, et al. (2018); Stark, Seff, et al. (2018)	Life skills sessions, mentoring, parental involvement and safe spaces	16	Weekly
Scales et al. (2013)	Peer education in social skills, literacy, and school learning, combined with study circles to support in‐school girls, education on budgeting, financial and business planning, and savings, and an incentive to delay marriage	48	Daily
Leight et al. (2021)	Participatory skill building courses that covered a very broad range of topics and are facilitated amongst women, men, and couples and coupled with a local ceremony	7	Daily
Lecoutere and Wuyts (2021)	Couple seminars and coaching through workshops and private visits, in participatory intra‐household decision making, plus women's leadership training	17	Monthly
Field et al. (2010)	Financial literacy skills for SHG members to improve access to financial services including a training module	8	Daily
Sharma et al. (2020)	Participatory skill building courses that cover a very broad range of topics and are facilitated amongst women, men, and couples and coupled with a local ceremony	7	Daily
Murray et al. (2020)	Teaching cognitive behavioural therapy skills (engagement, introduction/psychoeducation, safety, substance use reduction, cognitive coping and restructuring, problem solving, behavioural activation, relaxation, and exposure)	2	Weekly
Stark, Asghar, et al. (2018); Stark, Seff, et al. (2018)	Life skills sessions, mentoring, parental involvement and safe spaces	10	Weekly
Chinen and Elmeski (2016)	Training teachers as agents of change for promoting gender norms and equality	9	Unclear
Özler et al. (2020)	Girl Empower life skills curriculum, caregiver discussion groups, individual savings start‐up for the girls, and capacity building for local health and psychosocial service providers, combined with participation incentive payment	10	Weekly
Noble et al. (2019)	Foundational training, skill building in numeracy and a chosen vocational skill, cash stipend, formal and informal mechanisms to save money, and referrals to health, legal, and financial services, combined with safe spaces	Unclear	Weekly
Lecoutere (2018)	Couple seminars and coaching through workshops and private visits, in participatory intra household decision making, plus women's leadership training	17	Monthly
Ashraf et al. (2018)	Informal negotiation skills taught to girls along with role model exercises	5	Daily
Stark, Asghar, et al. (2018); Stark, Seff, et al. (2018)	Life skills sessions, mentoring, parental involvement and safe spaces	10	Weekly
Decker et al. (2018)	Empowerment self‐defence training in early recognition of boundary testing, negotiation, diffusion and distraction tactics, verbal assertiveness, and physical self‐defence	1.5	Weekly
Bastian et al. (2018)	Training on a financial product and business skills intervention	22	Weekly
Fuller (2014)	Training on women's rights issues, leadership skills and advocacy skills, combined with awareness‐raising and advocacy on women's property and literacy rights within their communities	60	Weekly
Halim et al. (2019)	Savings groups for women plus peer‐groups for men addressing gender relations and IPV prevention, with and without gender dialogues for community leaders.	8	Daily
Croke et al. (2020)	Training for cross‐border officials on andomized against SGBV	16	Daily
Gottlieb (2016)	Civics course on community participation in governance	2	Weekly
Kumar et al. (2018)	Women's groups for agricultural training, provision of initial inputs and nutrition BCC	48 months	Unclear

Abbreviations: BCC, behaviour change communication; SGBV, sexual and gender‐based violence.

*Comparison*

All included studies compared treated groups to comparison groups receiving no intervention. Twelve studies included multiple treatment arms.

*Outcomes*

The included studies reported on a number of relevant outcomes, including the following:

- Increased capacity of women to understand and use financial, banking, and business services effectively (*n* = 3): Women both benefit from enhanced skills and capacities in the use of financial, banking and business services. This can include training programmes, establishment of bank accounts, entrepreneurship training, business skills seminars and so on any activity aimed to increase women's independence in managing these assets/services.
- Women have increased access and ownership to assets, credit and income (*n* = 8): Women are able to apply for, receive and manage assets/credit and income and have support to manage, claim and execute their assets without pressure or influence from external actors, including male family members, husbands and cultural leaders.
- Women have more and better control over their bodies and sexual health (*n* = 7): Women are able to make free and educated decisions about their health (not included in this study), sexuality and procreation.
- Women are equipped with better life skills that allow them to be prepared for crisis or shocks and recover from them (*n* = 4): Women develop skills, have access to resources, and make use of these skills and resources to increase their level of resilience and be equipped to face life challenges (health, education, finance, social relations, work, etc.) These resources can take the form of Safe spaces, community dialogues and informal peer/community educators and increased ability of women to address life challenges (access to education, literacy, jobs, services, etc.). This would also cover activities supporting the development of self‐confidence in the capacity of women to face life challenges. During conflict, Women are equipped with increased skills and knowledge for resilience to recover and adapt to shocks, including learning how to have savings, adapt employment skills, without resorting to negative coping mechanisms and the related resources.
- Women have improved attitudes, self‐image and confidence (*n* = 6): Women feel more entitled to claim their rights and needs in community and change social norms and behaviours. They are aware of the importance of their status in society and are empowered to take this role and make use of the available resources to guarantee their rights.

The division of the immediate and secondary outcomes is reported in Table 56 (Table 51).

**Table 51:** Summary of secondary and immediate outcomes for LSCB and capacity building

Secondary outcome category	Immediate outcome	Number of studies
Resources material, human and social resources which serve to enhance the ability to exercise choice	Access to justice and legal services	1
	Economic and livelihood related resources	9
	Access to employment	1
Agency ability to define one's goals and act upon them and andomizedized decision‐making	Individual agency	15
	Community level agency	10
	Institutions supporting agency	0
Achievement ways of being and doing which can be andomiz by different individuals	Improved systems	1
	Norms and behaviour change	16
	Empowerment index	4

*Study design*

**Figure 147:**
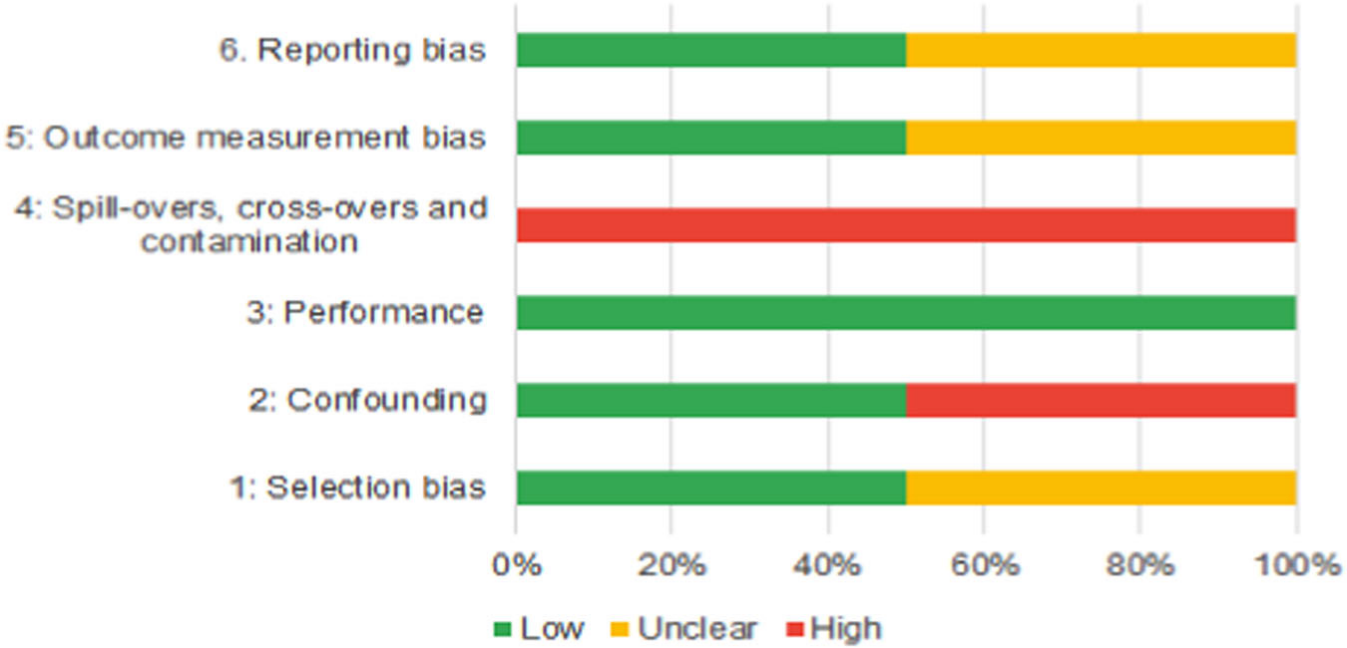
Life, social and livelihood skills and capacity building studies quasi‐experimental design risk of bias assessment

Two of our life, social and livelihood skills and capacity building studies used a QED. This included Lecoutere and Wuyts (2021) and Fuller (2014). We assessed both studies as having a high risk of bias. As detailed in Figure 147, high risk of bias was identified in two RoB domains (confounding and spill overs). Confounding was assessed as a risk of bias in one of the studies that did not account for cluster controls and could not reject unobserved bias (Fuller, 2014). Spill‐overs were an issue when participants in comparison areas reported having participated in similar activities than those delivered by the program (Lecoutere & Wuyts, 2021; Fuller, 2014). For other domains, we did not have enough information to justify the absence of bias in at least one study, this was the case for selection bias, outcome measurement and reporting bias. The only domain we did not identify any issue was performance, where there was no obvious issue with monitoring in any of the studies.

Twenty‐four of our life, social and livelihood skills and capacity building studies use an experimental design, including (among others) Ashraf et al. (2018), Bastian et al. (2018), Buehren, Chakravarty, et al. (2017); Croke et al. (2020), Chinen and Elmeski (2016), Decker et al. (2018) and Field et al. (2010).

**Figure 148:**
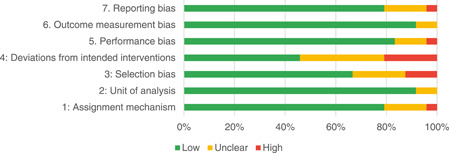
Life, social and livelihood skills and capacity building studies andomized controlled trial risk of bias assessment

We assessed about one fourth of the studies as having a low risk of bias, however, 30% had a high risk of bias and the rest had some concerns. As detailed in Figure 148, high risk of bias was identified in five RoB domains (assignment mechanism, selection, deviation, performance and reporting). The most common issue in the RCT studies in the capacity building interventions was deviations from the intended interventions. For example, two studies suffered from survey effects because of including information in the data collection tools that could have given the control participants an idea of what the intervention group received (Murray et al., 2020; Scales et al., 2013), while other studies reported risks of noncompliance of the assignment to treatment and control groups (Buehren, Chakravarty, et al., 2017; Chinen & Elmeski, 2016; Croke et al., 2020).

We identified a high risk of selection bias in studies that suffered from differential attrition (Halim et al., 2019; Murray et al., 2020) or very high attrition even if not correlated to the intervention (Buehren, Chakravarty, et al., 2017; Croke et al., 2020). We also assessed one study as having a high risk of bias on the assignment mechanism because the authors do not repot balance at baseline between treatment and control groups, so that not enough information was available to confirm that randomization had worked (Gottlieb, 2016).

Other limitations were related to performance and reporting biases. We assessed one study as having a high risk of motivation bias among participants, introduced by the process of monitoring, and a high risk of reporting bias because the authors do not provide separate analysis for all treatment arms (Scales et al., 2013). The same study does not consider clustering in the analysis of results and do not provide enough information to rule out that attrition is related to treatment, or to confirm that balance is achieved at baseline.

*Qualitative studies, process evaluations and project documents*

We identified 16 additional documents related to six (06) programmes covered by the life, social and livelihood skills and capacity building included studies.

- Creating opportunities through mentoring, parental involvement and safe spaces (COMPASS) programme (DRC): one qualitative and two descriptive quantitative documents
- Kishoree Kontha (‘Adolescent Girls’ Voices’) (Bangladesh): one process evaluation
- Hanns R. Neumann Stiftung ‘Gender Household Approach’ (Uganda): one descriptive quantitative document
- Women for Women International's (WfWI) What Works to Prevent Violence Programme (Afghanistan): four descriptive quantitative documents and one qualitative document
- Unite for a Better Life (UBL) (Ethiopia): one process evaluation, two descriptive quantitative document
- Together to End Violence Against Women (TEVAW) (Tanzania): one descriptive quantitative document
- Realigning Agriculture for Improved Nutrition (RAIN) (Zambia): two descriptive quantitative documents

The majority of these studies were marked as moderate (*n* = 6) or low (*n* = 6) empirical quality. The remainder (*n* = 4) were marked as high quality.

##### 5.6.3.3 Synthesis of findings

The following subsection presents the results of effectiveness of life, social and livelihood skills and capacity building on gender equality, women's empowerment and peace outcomes.

*Quantitative findings*

*Effects of capacity building on women's access to rights, services and opportunities*

Stark, Asghar and colleagues’ (2018) experimental study in Ethiopia was the only study evaluating the impact of life, social and livelihood skills and capacity building on women's access to rights, services and opportunities. There was a very small, but not statistically significant, positive point estimate (*g *=* *0.02, [95% CI: −0.12 to 0.16]), and we assessed the study as having some risk of bias concerns.

*Effects of capacity building on increased capacity of women to understand and use financial, banking and business services effectively*

**Figure 149:**
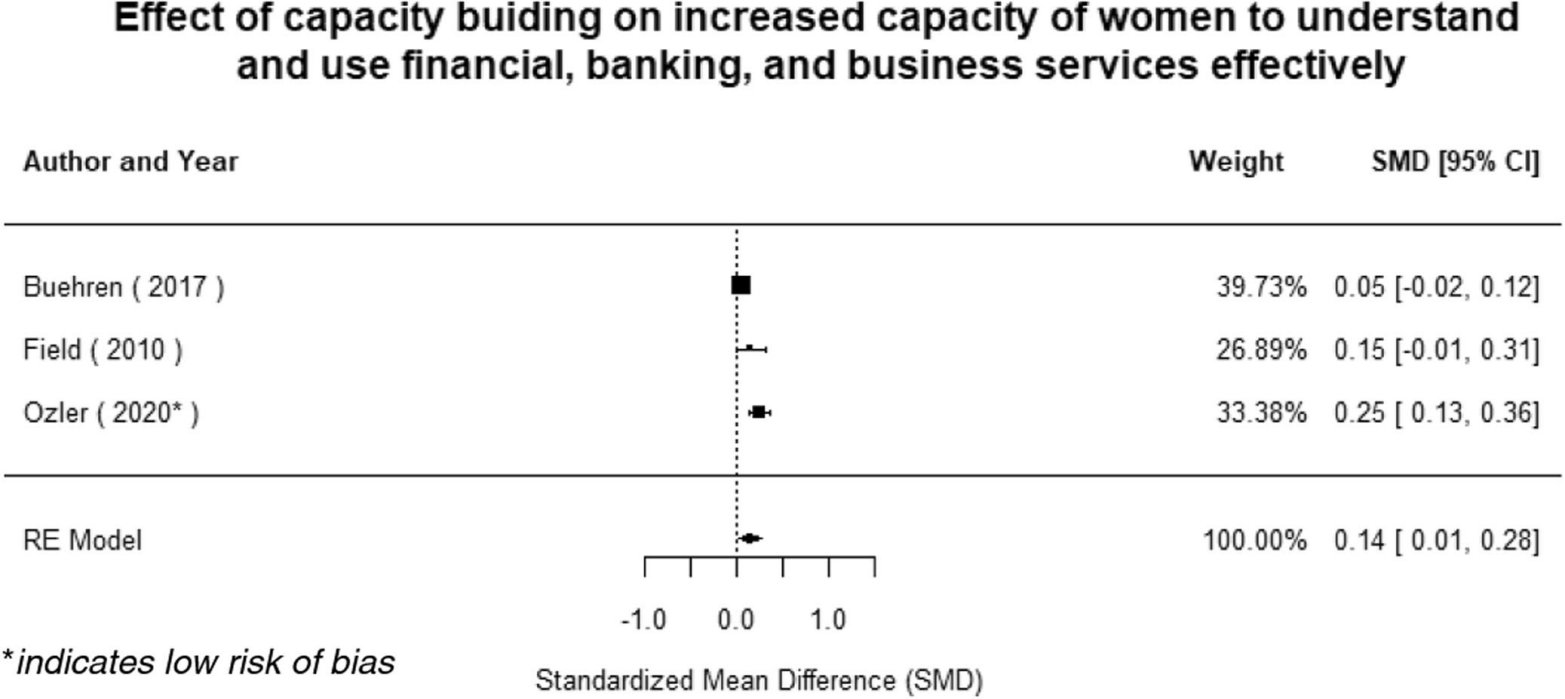
CBAB1: Forest plot showing the observed outcomes and the estimate of the random‐effects model. CI, confidence interval

We included a total of k=3 studies in the analysis. We assessed one study as low risk of bias, one as some concerns, and one as high risk of bias. The observed outcomes ranged from 0.05 to 0.25. The estimated average outcome based on the random‐effects model was $\hat{\mu }=0.14$ (95% CI: 0.01 to 0.28). Therefore, the average outcome differed significantly from zero (z=2.14, p=0.03). A forest plot showing the observed outcomes and the estimate based on the random‐effects model is shown in Figure 149 (CBAB1).

According to the Q‐test, the true outcomes appear to be heterogeneous (Q(2)=8.53, p=0.01, ${\hat{\tau }}^{2}=0.01$, $I^{2}=76.54$%). An examination of the studentised residuals revealed that one study (Buehren, Chakravarty, et al., 2017) had a value larger than ±2.39 and may be a potential outlier in the context of this model. According to the Cook's distances, none of the studies could be considered to be overly influential. With only three studies, moderator analyses were not appropriate.

*Effects of capacity building on increased access to and ownership of assets, credit and income*

**Figure 150:**
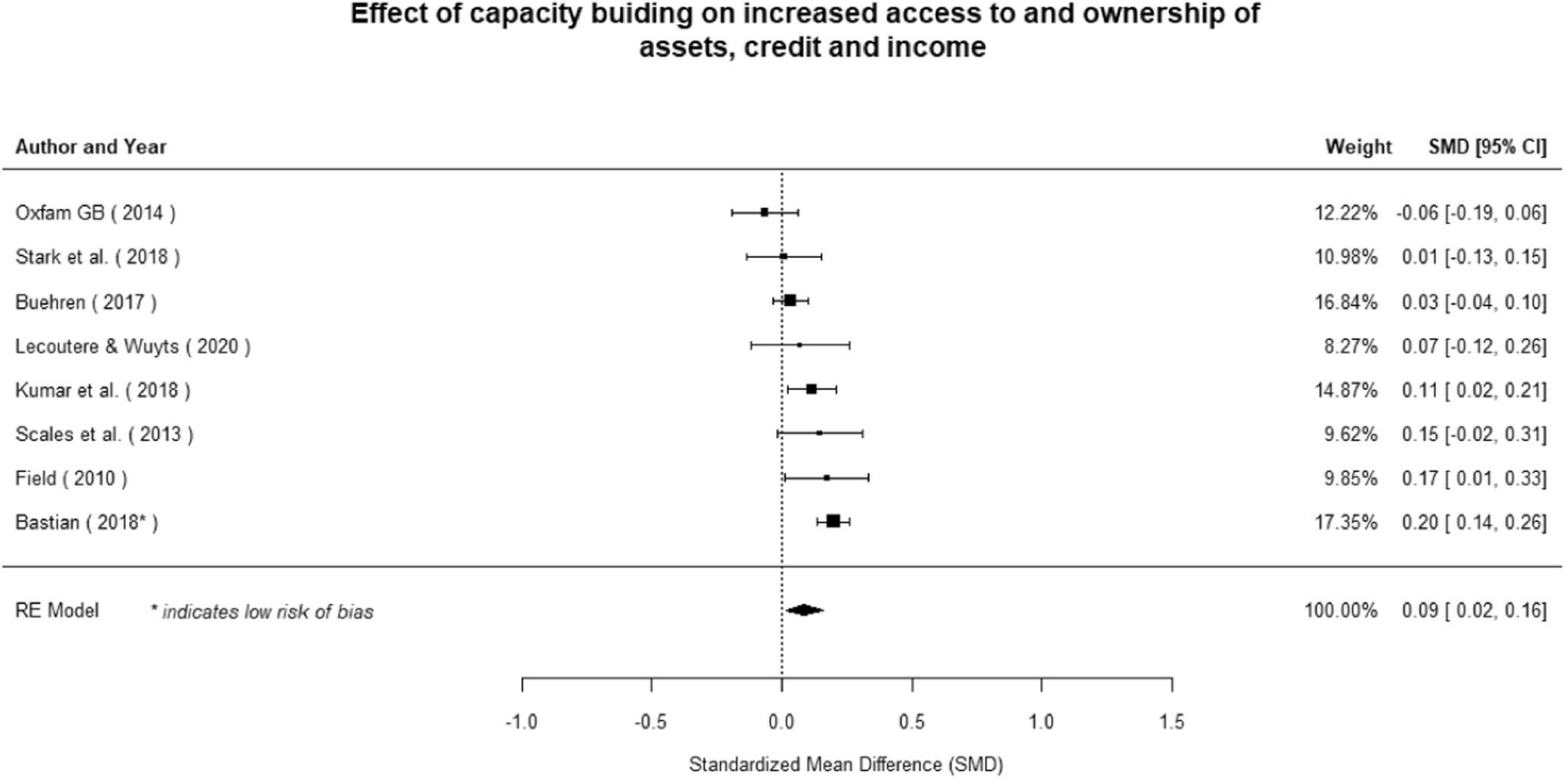
CBAB2: Forest plot showing the observed outcomes and the estimate of the random‐effects model. CI, confidence interval

We included a total of k=8 studies in the analysis. The observed outcomes ranged from −0.06 to 0.20. We assessed one study as low risk of bias, four as some concerns, and four as high risk of bias. The estimated average outcome based on the random‐effects model was $\hat{\mu }=0.09$ (95% CI: 0.02 to 0.16). Therefore, the average outcome differed significantly from zero (z=2.42, p=0.02). A forest plot showing the observed outcomes and the estimate based on the random‐effects model is shown in Figure 150 (CBAB2).

According to the Q‐test, the true outcomes appear to be heterogeneous (Q(7)=22.55, p<0.01, ${\hat{\tau }}^{2}=0.01$, $I^{2}=68.96$%). An examination of the studentized residuals revealed that none of the studies had a value larger than ±2.73 and hence there was no indication of outliers in the context of this model. According to the Cook's distances, none of the studies could be considered to be overly influential.

We again tested all moderators. High risk of bias studies were not significantly different from studies assessed as low or some concerns ($\hat{B}=-0.10,p=0.07$ [95% CI: −0.21 to 0.01]). Length of exposure to the intervention ($\hat{B}=-0.003,p=0.08$ [95% CI: −0.01 to 0.001]) and evaluation period ($\hat{B}=0.001,p=0.91$ [95% CI: −0.01 to 0.01]) we also not significant sources of variation. Effects from experimental studies were not different than effects from quasi‐experimental studies ($\hat{B}=-0.13,p=0.11$ [95% CI: −0.28 to 0.03]). Effects also did not differ by publication year ($\hat{B}=-0.002,p=0.88$ [95% CI: −0.03 to 0.02]), by gender inequality score ($\hat{B}=-0.37,p=0.68$ [95% CI: −2.14 to 1.40]), nor by FSI score ($\hat{B}=-0.01,p=0.17$ [95% CI: −0.01 to 0.003]). Finally, effects were no different between studies that target women only versus studies targeting mixed genders ($\hat{B}=-0.06,p=0.58$ [95% CI: −0.25 to 0.14]). There were not sufficient numbers in each group to test by country and we could not test for adjustment by covariates as only one model was unadjusted.

*Effects of capacity building on initiatives that facilitate women's access to decent work*

Buehren, Chakravarty, et al.'s (2017) experimental study in South Sudan was the only study evaluating the impact of life, social and livelihood skills and capacity building on initiatives that facilitate women's, including people with disabilities, access to decent work (formal and informal employment). Their report included five effects that fell into this outcome category (e.g., self‐employment and farmer's self‐employment). The effects ranged from very small, negative point estimates (*g *=* *−0.01, [95% CI: −0.08 to 0.06]) for hours worked per month to very small, positive effects (*g *=* *0.09, 95% CI [0.02, 0.16]) for nonfarm self‐employment. We assessed the study as having high risk of bias.

*Effects of capacity building on improved capacity of women entrepreneurs*

Buehren, Chakravarty, et al.'s (2017) experimental study in South Sudan was also the only study evaluating the impact of life, social and livelihood skills and capacity building on improved capacity of women entrepreneurs. There was a very small, but not statistically significant, negative point estimate (*g *=* *−0.02, [95% CI: −0.09 to 0.05]), and we assessed the study as having high risk of bias.

*Effects of capacity building on women's success in the workplace*

Bastian et al.'s (2018) experimental study in Tanzania was the only study evaluating the impact of life, social and livelihood skills and capacity building on women having improved success in the workplace. Their report included five effects that fell into this outcome category (e.g., marketing score, which is the proportion of seven business marketing practices used by the firm.). The effects ranged from very small, negative point estimates (*g *=* *0.06, [95% CI: −0.00 to 0.13]) to medium, positive effects (*g *=* *0.35, 95% CI [0.28, 0.41]). We assessed the study as having low risk of bias.

*Effects of capacity building on women having increased freedom of movement and association*

**Figure 151:**
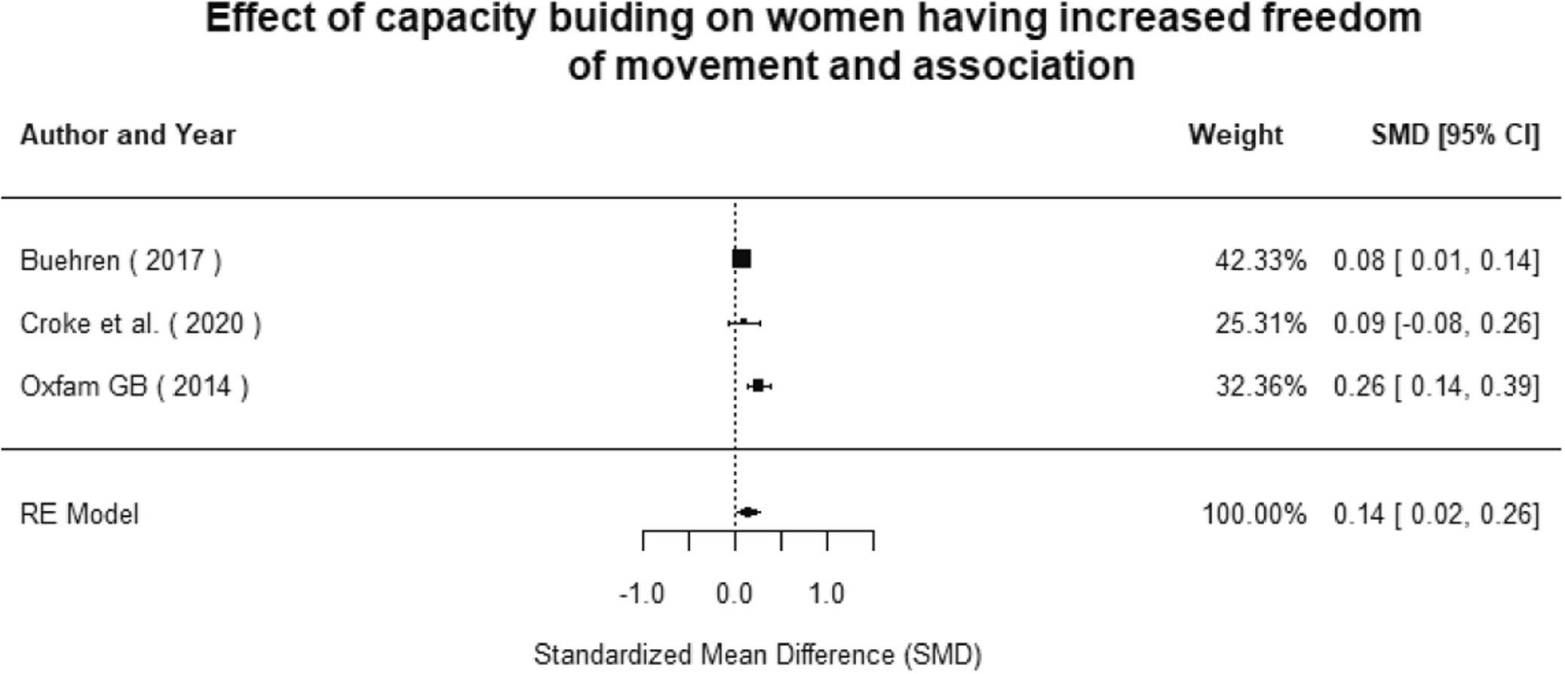
CBBA3: Forest plot showing the observed outcomes and the estimate of the random‐effects model. CI, confidence interval

We included a total of k=3 studies in the analysis. We assessed all three studies as high risk of bias. The observed outcomes ranged from 0.08 to 0.26. The estimated average outcome based on the random‐effects model was $\hat{\mu }=0.14$ (95% CI: 0.02 to 0.26). Therefore, the average outcome differed significantly from zero (z=2.22, p=0.03). A forest plot showing the observed outcomes and the estimate based on the random‐effects model is shown in Figure 151 (CBBA3).

According to the Q‐test, the true outcomes appear to be heterogeneous (Q(2)=6.63, p=0.04, ${\hat{\tau }}^{2}=0.01$, $I^{2}=69.82$%). An examination of the studentised residuals revealed that one study (Fuller, 2014) had a value larger than ±2.39 and may be a potential outlier in the context of this model. According to the Cook's distances, none of the studies could be overly influential. All three studies were assessed as having a high risk of bias. With only three studies, moderation analyses and tests of publication bias were not appropriate.

*Effects of capacity building on women being more aware of their rights, and the roles and responsibilities of duty bearers*

**Figure 152:**
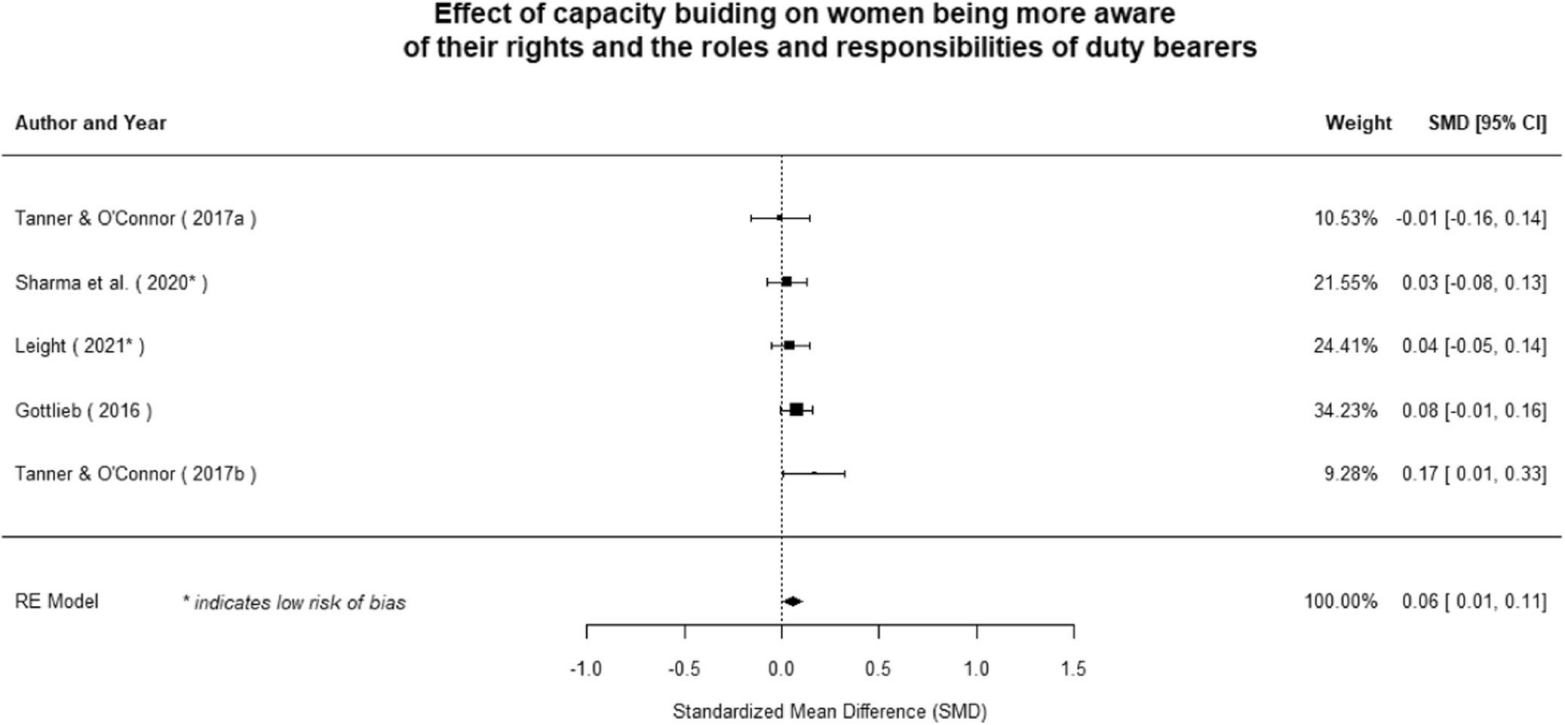
CBBA4: Forest plot showing the observed outcomes and the estimate of the random‐effects model. CI, confidence interval

We included a total of k=5 studies in the analysis. We assessed two studies as low risk of bias, two as some concerns, and one as high risk of bias. The observed outcomes ranged from −0.01 to 0.17. The estimated average outcome based on the random‐effects model was $\hat{\mu }=0.06$ (95% CI: 0.01 to 0.11). Therefore, the average outcome differed significantly from zero (z=2.33, p=0.02). A forest plot showing the observed outcomes and the estimate based on the random‐effects model is shown in Figure 152 (CBBA4).

According to the Q‐test, there was no significant amount of heterogeneity in the true outcomes (Q(4)=3.17, p=0.53, ${\hat{\tau }}^{2}=0.00$, $I^{2}=0.00$%), thus we did not test for moderation. An examination of the studentised residuals revealed that none of the studies had a value larger than ±2.58 and hence there was no indication of outliers in the context of this model. According to the Cook's distances, none of the studies could be overly influential.

*Effects of capacity building on women having more positive attitude towards taking action to claim their rights*

Decker et al.'s (2018) experimental study in Malawi was the only study evaluating the impact of life, social and livelihood skills and capacity building on women having more positive attitude towards taking action to claim their rights. Their report included three effects that fell into this outcome category (e.g., sexual violence disclosure) for all schools and for primary and secondary schools separately. The effect across all schools was a very small, positive point estimate (*g *=* *0.03, [95% CI: −0.03 to 0.09]), the effect across primary schools was a very small, point estimate (*g *=* *0.01, [95% CI: −0.05 to 0.08]), and the effect across secondary schools was a very small, positive point estimate (*g *=* *0.06, [95% CI: −0.07 to 0.18]). We assessed the study as having some risk of bias concerns.

*Effects of capacity building on reduced percentage of women agreeing with certain reasons that justify violence against women and girls*

**Figure 153:**
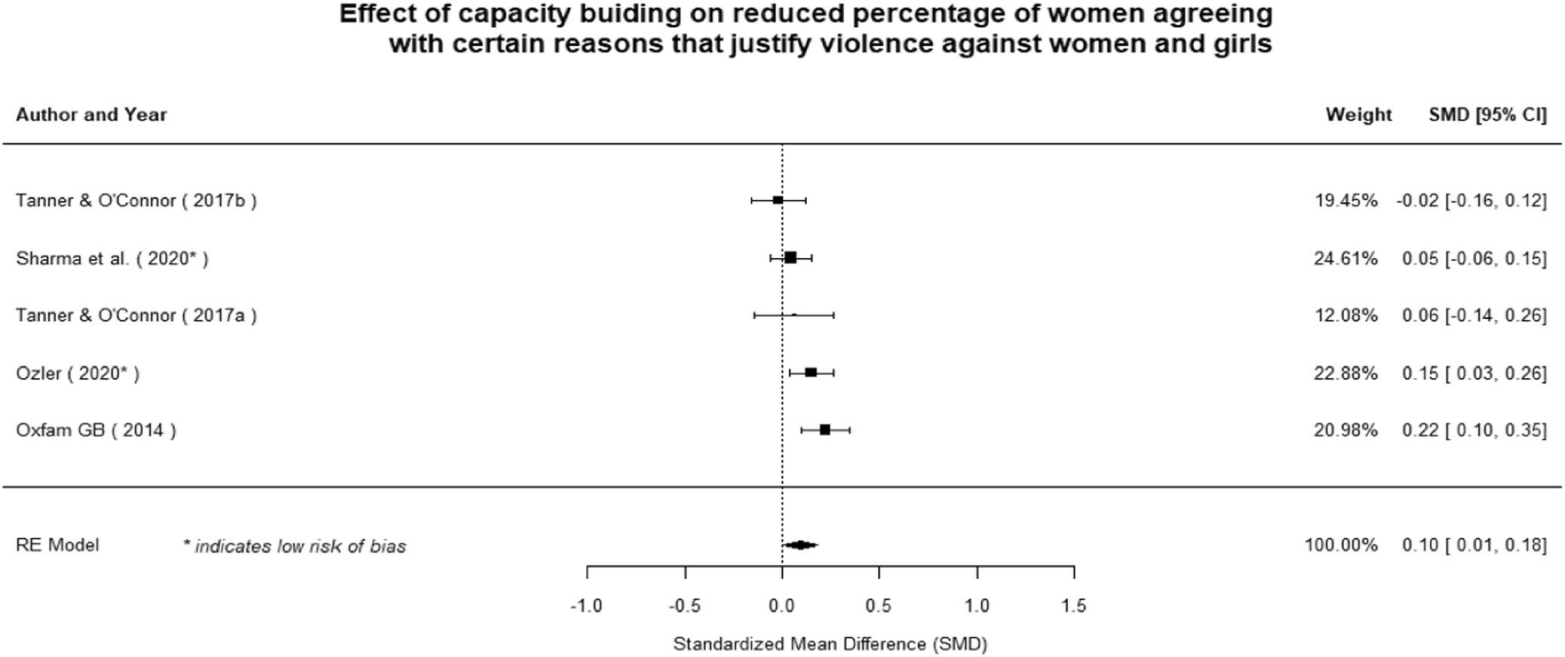
CBBA6: Forest plot showing the observed outcomes and the estimate of the random‐effects model. CI, confidence interval

We included a total of k=5 studies in the analysis. We assessed two studies as low risk of bias, two as some concerns, and one as high risk of bias. The observed outcomes ranged from −0.02 to 0.22. The estimated average outcome based on the random‐effects model was $\hat{\mu }=0.10$ (95% CI: 0.01 to 0.18). Therefore, the average outcome differed significantly from zero (z=2.21, p=0.03). A forest plot showing the observed outcomes and the estimate based on the random‐effects model is shown in Figure 153 (CBBA6). Tanner and O'Connor (2017a) is a study from the Democratic Republic of Congo, which Tanner and O'Connor (2017b) is a study from Ethiopia.

The Q‐test for heterogeneity was not significant, but some heterogeneity may still be present in the true outcomes (Q(4)=8.38, p=0.08, ${\hat{\tau }}^{2}=0.00$, $I^{2}=52.29$%). An examination of the studentised residuals revealed that none of the studies had a value larger than ±2.58 and hence there was no indication of outliers in the context of this model. According to the Cook's distances, none of the studies could be considered to be overly influential.

Studies with a high risk of bias were not significantly different than studies with some concerns or low risk of bias ($\hat{B}=-0.10,p=0.07$ [95% CI: −0.21 to 0.01]). Exposure to intervention in months ($\hat{B}=0.004,p=0.07$ [95% CI: −0.0003 to 0.008]) and evaluation period in months ($\hat{B}=-0.002,p=0.74$ [95% CI: −0.01 to 0.01]) were also not significant, no was publication year ($\hat{B}=-0.001,p=0.47$ [95% CI: −0.05 to 0.02]). The gender inequality score was a significant source of variation among effects ($\hat{B}=1.11,p=0.01$ [95% CI: 0.29 to 1.94]) such that programme effects were larger in areas with higher levels of gender inequality.

We could not test for variation in effects by study design (only 1 study used a QED). All models were adjusted for covariates. We also could not test by fragile state score because four of the studies had the same score. In addition, only one of the five studies targed a mixed‐gender sample and we were underpowered to test for country differences.

*Effects of capacity building on women having better life skills*

**Figure 154:**
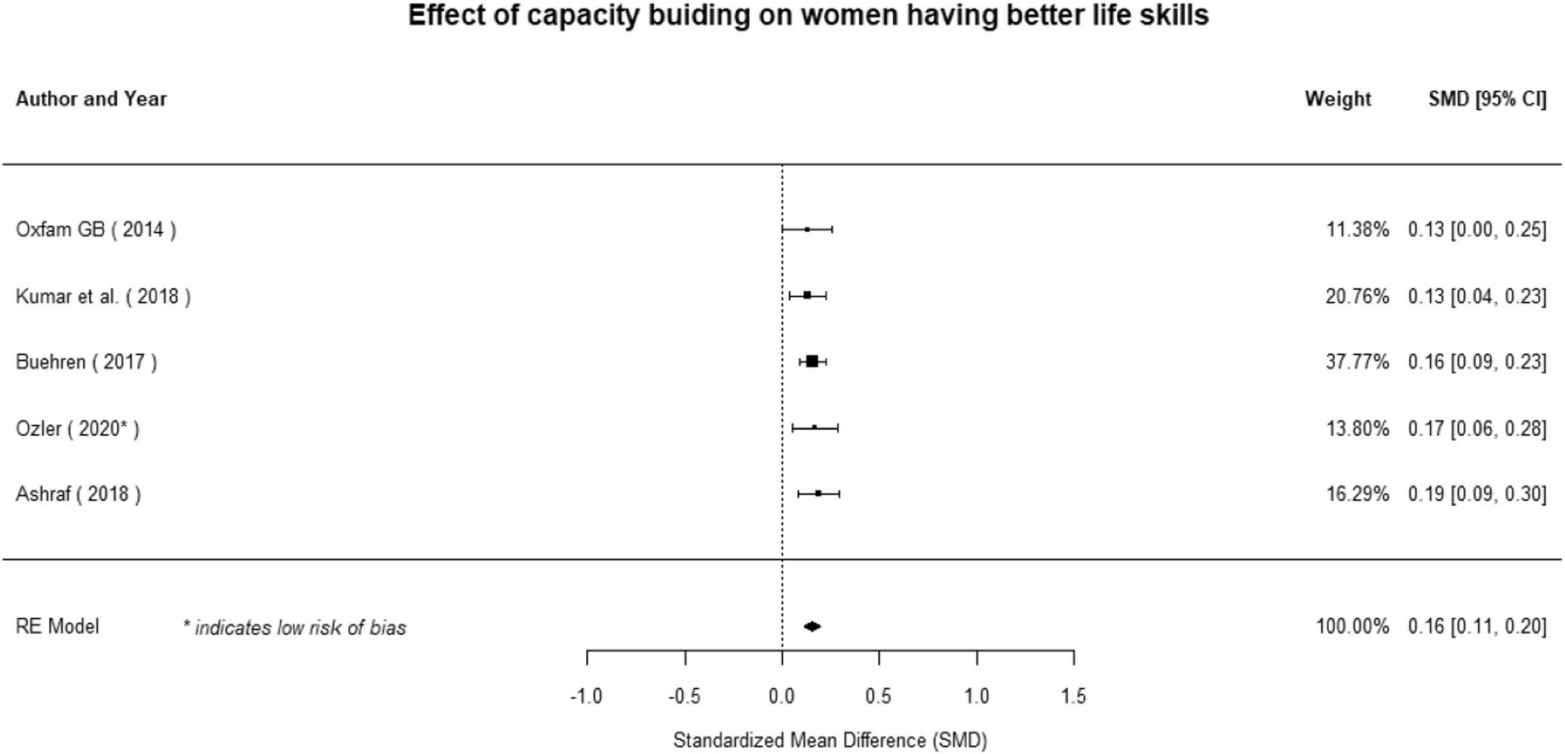
CBBA7: Forest plot showing the observed outcomes and the estimate of the random‐effects model. CI, confidence interval

We included a total of k=5 studies in the analysis. We assessed one study as low risk of bias, two as some concerns, and two as high risk of bias. The observed outcomes ranged from 0.13 to 0.19 The estimated average outcome based on the random‐effects model was $\hat{\mu }=0.16$ (95% CI: 0.11 to 0.20). Therefore, the average outcome differed significantly from zero (z=7.23, p<0.01). A forest plot showing the observed outcomes and the estimate based on the random‐effects model is shown in Figure 154 (CBBA7).

According to the Q‐test, there was no significant amount of heterogeneity in the true outcomes (Q(4)=0.90, p=0.92, ${\hat{\tau }}^{2}=0.00$, $I^{2}=0.00$%), thus we did not test for moderation. An examination of the studentised residuals revealed that none of the studies had a value larger than ±2.58 and hence there was no indication of outliers in the context of this model. According to the Cook's distances, none of the studies could be overly influential.

*Effects of capacity building on reduced support for or instances of child and forced marriage*

**Figure 155:**
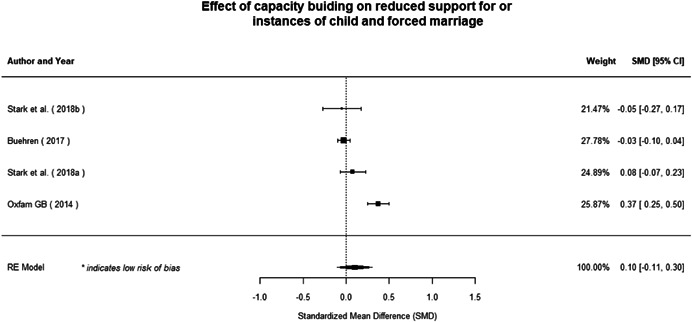
CBBA8: Forest plot showing the observed outcomes and the estimate of the random‐effects model. CI, confidence interval

We included a total of k=4 studies in the analysis. We assessed none of the studies as low risk of bias, two as some concerns, and two as high risk of bias. The observed outcomes ranged from −0.05 to 0.37. The estimated average outcome based on the random‐effects model was $\hat{\mu }=0.10$ (95% CI: −0.11 to 0.30). Therefore, the average outcome did not differ significantly from zero (z=0.93, p=0.35). A forest plot showing the observed outcomes and the estimate based on the random‐effects model is shown in Figure 155 (CBBA8). Stark, Asghar, et al. (2018) is a study that took place in Ethiopia, while Stark, Seff, et al. (2018) is a study that took place in the Democratic Republic of Congo. Both Stark studies were assessed as having a high risk of bias, and the other two studies were assessed as some concerns related to risk of bias.

According to the Q‐test, the true outcomes appear to be heterogeneous (Q(3)=30.66, p<0.01, ${\hat{\tau }}^{2}=0.04$, $I^{2}=90.22$%). An examination of the studentised residuals revealed that one study (Fuller, 2014) had a value larger than ±2.50 and may be a potential outlier in the context of this model. According to the Cook's distances, none of the studies could be overly influential.

Studies with a high risk of bias were not significantly different than studies with some concerns or low risk of bias ($\hat{B}=0.15,p=0.50$ [95% CI: −0.29 to 0.58]). Exposure to intervention in months ($\hat{B}=0.01,p=0.19$ [95% CI: −0.004 to 0.02]) was also not significant. Publication year was significant such that more recent studies found smaller effects ($\hat{B}=-0.10,p=0.01$ [95% CI: −0.17 to −0.02]), however the range of years is very small (2014 to 2018). Effects did not differ by gender inequality scores ($\hat{B}=0.64,p=0.81$ [95% CI: −4.52 to 5.79]), but they did differ by fragility ($\hat{B}=-0.02,p=0.02$ [95% CI: −0.004 to −0.003]) such that programme effects were smaller in more fragile areas.

Only one study was a quasi‐experimental study. We could also not test for variation by evaluation period, because three of the four studies collected data immediately after the end of the intervention. Additionally, all models were adjusted for covariates and all programmes target women only

*Effects of capacity building on increased participation in decision making by women at the household or community level*

**Figure 156:**
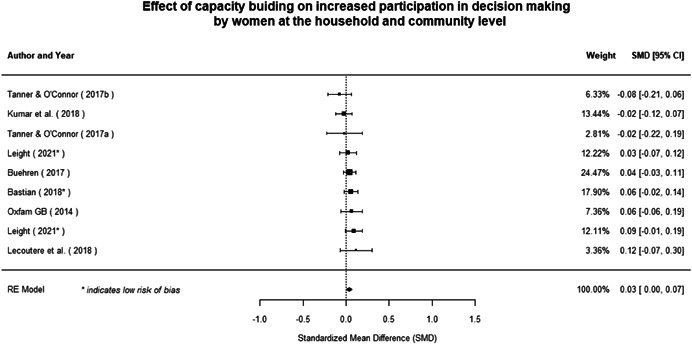
CBBB1: Forest plot showing the observed outcomes and the estimate of the random‐effects model. CI, confidence interval

We included a total of k=9 studies in the analysis. We assessed two studies as low risk of bias, four as some concerns, and two as high risk of bias. The observed outcomes ranged from −0.08 to 0.12. The estimated average outcome based on the random‐effects model was $\hat{\mu }=0.03$ (95% CI: 0.001 to 0.07). Therefore, the average outcome differed significantly from zero (z=2.00, p=0.046). A forest plot showing the observed outcomes and the estimate based on the random‐effects model is shown in Figure 156 (CBBB1). Tanner and O'Connor (2017a) reports on a study taking place in Democratic Republic of Congo, while Tanner and O'Connor (2017b) reports on a study taking place in Ethiopia. In addition, the two Leight et al. (2021) effects are independent, with one effect for women (*g *=* *0.03) and one for men (*g *=* *0.09).

According to the Q‐test, there was no significant amount of heterogeneity in the true outcomes (Q(8)=7.01, p=0.54, ${\hat{\tau }}^{2}=0.00$, $I^{2}=0.00$%), thus we did not test for sources of variation. An examination of the studentised residuals revealed that none of the studies had a value larger than ±2.77 and hence there was no indication of outliers in the context of this model. According to the Cook's distances, none of the studies could be considered to be overly influential. With no heterogeneity, we did not test for sources of heterogeneity.

*Effects of capacity building on women participating more in their community*

**Figure 157:**
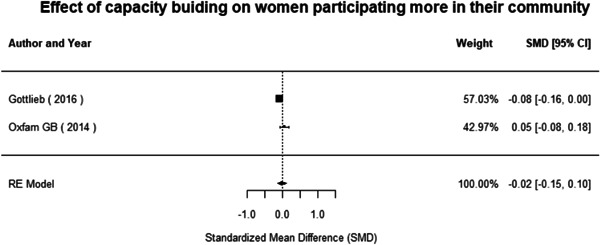
CBBB2: Forest plot showing the observed outcomes and the estimate of the random‐effects model. CI, confidence interval

Two studies examined the effects of capacity building on women participating more in their community, thus we included *k *=* *2 studies in the analysis. We assessed both studies as high risk of bias. The estimated average outcome based on the random‐effects model was $\hat{\mu }=-0.02$ (95% CI: −0.15to0.10). Therefore, the average outcome did not differ significantly from zero (z=−0.36, p=0.72). A forest plot showing the observed outcomes and the estimate based on the random‐effects model is shown in Figure 157 (CBBB2). Given the small number of studies, this result should be interpreted with caution. According to the Q‐test, there was no significant amount of heterogeneity in the true outcomes (Q(1)=2.82, p=0.09, ${\hat{\tau }}^{2}=0.01$, $I^{2}=64.49$%). With only two studies, moderator analyses were not possible and tests of publication bias are not valid.

*Effects of capacity building on whether relief and recovery initiatives in conflict and post conflict situations responded to the needs of women and girls, especially vulnerable groups*

Fuller (2014) quasi‐experimental study in Sierra Leone was the only study evaluating the impact of life, social and livelihood skills and capacity building on whether relief and recovery initiatives in conflict and post conflict situations responded to the needs of women and girls, especially vulnerable groups. There was a very small, but not statistically significant point estimate (*g *=* *−0.08, [95% CI: −0.20 to 0.05]), and we assessed the study as having high risk of bias.

*Effects of capacity building on increased community awareness of the issues affecting women*

Tanner and O'Connor (2017b) experimental study in Ethiopia was the only study evaluating the impact of life, social and livelihood skills and capacity building on increased community awareness of the issues affecting women. There was a very small, but not statistically significant, point estimate (*g *=* *−0.01, [95% CI −0.17 to 0.15]), and we assessed the study as having some risk of bias concerns.

*Effects of capacity building on communities having more positive attitudes towards women*

**Figure 158:**
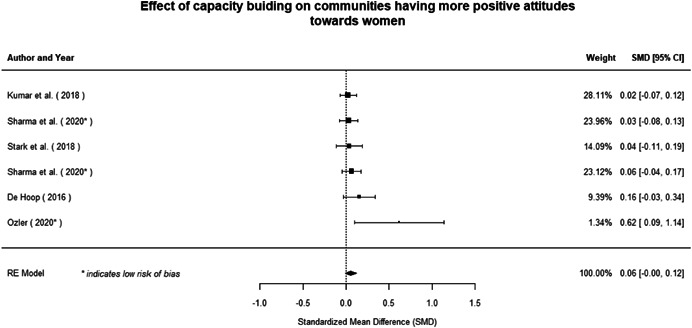
CBCB2: Forest plot showing the observed outcomes and the estimate of the random‐effects model. CI, confidence interval

We included a total of k=6 studies in the analysis. The observed outcomes ranged from 0.02 to 0.62. We assessed two studies as low risk of bias, two as some concerns, and one as high risk of bias. The estimated average outcome based on the random‐effects model was $\hat{\mu }=0.06$ (95% CI: −0.00 to 0.12). Therefore, the average outcome did not differ significantly from zero (z=1.81, p=0.07). A forest plot showing the observed outcomes and the estimate based on the random‐effects model is shown in Figure 158 (CBCB2). Once again, the Sharma (2020) estimates are separate for men (*g *=* *0.06) and women (*g *=* *0.03).

According to the Q‐test, there was no significant amount of heterogeneity in the true outcomes (Q(5)=6.29, p=0.28, ${\hat{\tau }}^{2}=0.00$, $I^{2}=20.52$%), thus we did not test for moderation. An examination of the studentised residuals revealed that none of the studies had a value larger than ±2.64 and hence there was no indication of outliers in the context of this model. According to the Cook's distances, none of the studies could be considered to be overly influential.

*Effects of capacity building on women having improved attitudes, self‐image and confidence*

**Figure 159:**
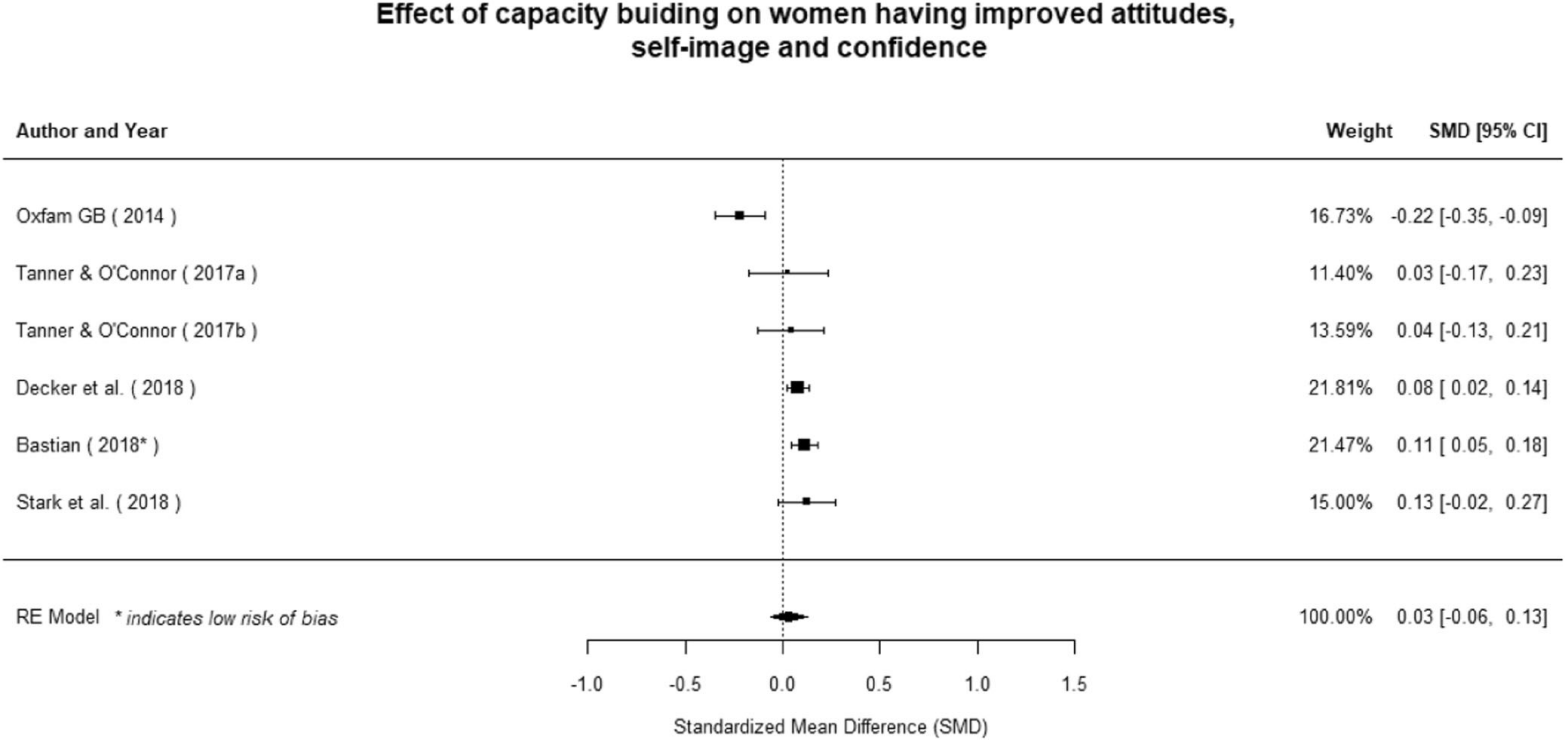
CBCB3: Forest plot showing the observed outcomes and the estimate of the random‐effects model. CI, confidence interval

We included a total of k=6 studies in the analysis. We assessed one study as low risk of bias, four as some concerns, and one as high risk of bias. The observed outcomes ranged from −0.22 to 0.13. The estimated average outcome based on the random‐effects model was $\hat{\mu }=0.03$ (95% CI: −0.06 to 0.13). Therefore, the average outcome did not differ significantly from zero (z=0.68, p=0.49). A forest plot showing the observed outcomes and the estimate based on the random‐effects model is shown in Figure 159 (CBCB3). One study was low risk of bias (Bastian et al., 2018), one study was high risk of bias (Fuller, 2014), and the remainder were assessed as some concerns related to risk of bias. Tanner and O'Connor (2017a) refers to a study from the Democratic Republic of Congo, while Tanner and O'Connor (2017b) refers to a study from Ethiopia, thus the samples are independent.

According to the Q‐test, the true outcomes appear to be heterogeneous (Q(5)=22.61, p<0.01, ${\hat{\tau }}^{2}=0.01$, $I^{2}=77.89$%). An examination of the studentised residuals revealed that one study (Fuller, 2014) had a value larger than ±2.64 and may be a potential outlier in the context of this model. According to the Cook's distances, one study (Fuller, 2014) could be overly influential. Indeed, sensitivity analyses leaving each study out indicated that removing Fuller (2014) would increase the overall average effect ($\hat{\mu }\;=\;$0.09 (95% CI: 0.05 to 0.13), and the effect would still be positive but would also become significant (*z *=* *4.46, *p *<* *0.001).

Exposure to intervention in months was significant ($\hat{B}=-0.01,p=0.002$ [95% CI: −0.01 to −0.003]) such that longer interventions reported smaller effect (a reduction of 0.01 standard deviation units per month of exposure). Evaluation period in months, however, was not a significant source of variation ($\hat{B}=0.01,p=0.39$ [95% CI: −0.01 to 0.01]). Effects also did not differ by level of gender inequality ($\hat{B}=-1.40,p=0.09$ [95% CI: −3.03 to 0.24]), nor by level of fragility ($\hat{B}=-0.002,p=0.80$ [95% CI: −0.02 to 0.02]).

Only one study used a QED, and only one study did not adjust their model for covariates. With only one high risk of bias study, we could not examine moderation by study quality. Finall, only one study targeted mixed genders while the rest targeted women only.

*Effects of capacity building on improved attitudes and increased support for women's economic, social and human rights*

**Figure 160:**
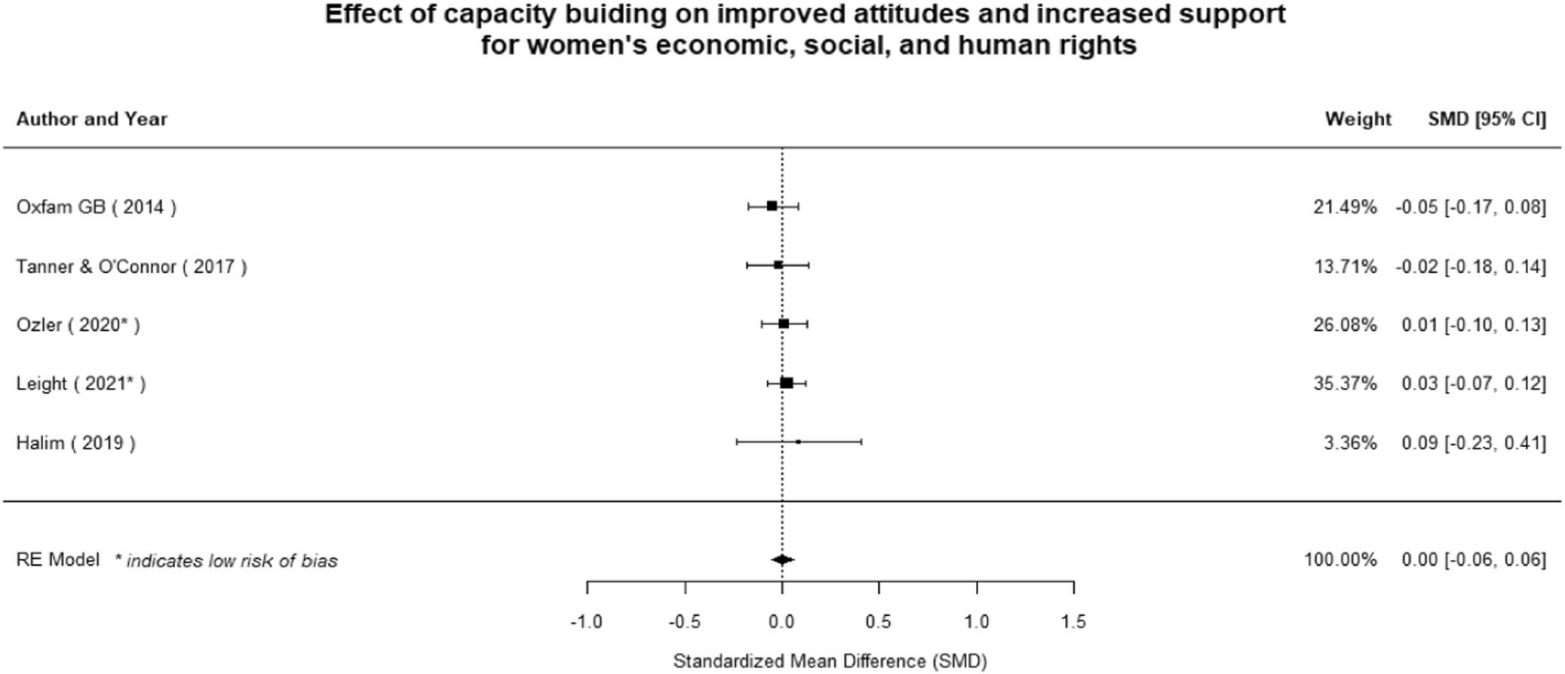
CBCB4: Forest plot showing the observed outcomes and the estimate of the random‐effects model. CI, confidence interval

We included a total of k=5 studies in the analysis. We assessed two studies as low risk of bias, one as some concerns, and two as high risk of bias. The observed outcomes ranged from −0.05 to 0.09. The estimated average outcome based on the random‐effects model was $\hat{\mu }=0.00$ (95% CI: −0.06 to 0.06). Therefore, the average outcome did not differ significantly from zero (z=0.07, p=0.94). A forest plot showing the observed outcomes and the estimate based on the random‐effects model is shown in Figure 160 (CBCB4).

According to the Q‐test, there was no significant amount of heterogeneity in the true outcomes (Q(4)=1.17, p=0.88, ${\hat{\tau }}^{2}=0.00$, $I^{2}=0.00$%), thus we did not test for moderation. An examination of the studentised residuals revealed that none of the studies had a value larger than ±2.58 and hence there was no indication of outliers in the context of this model. According to the Cook's distances, none of the studies could be considered overly influential.

*Effects of capacity building on decreased violence and discrimination at the household level*

**Figure 161:**
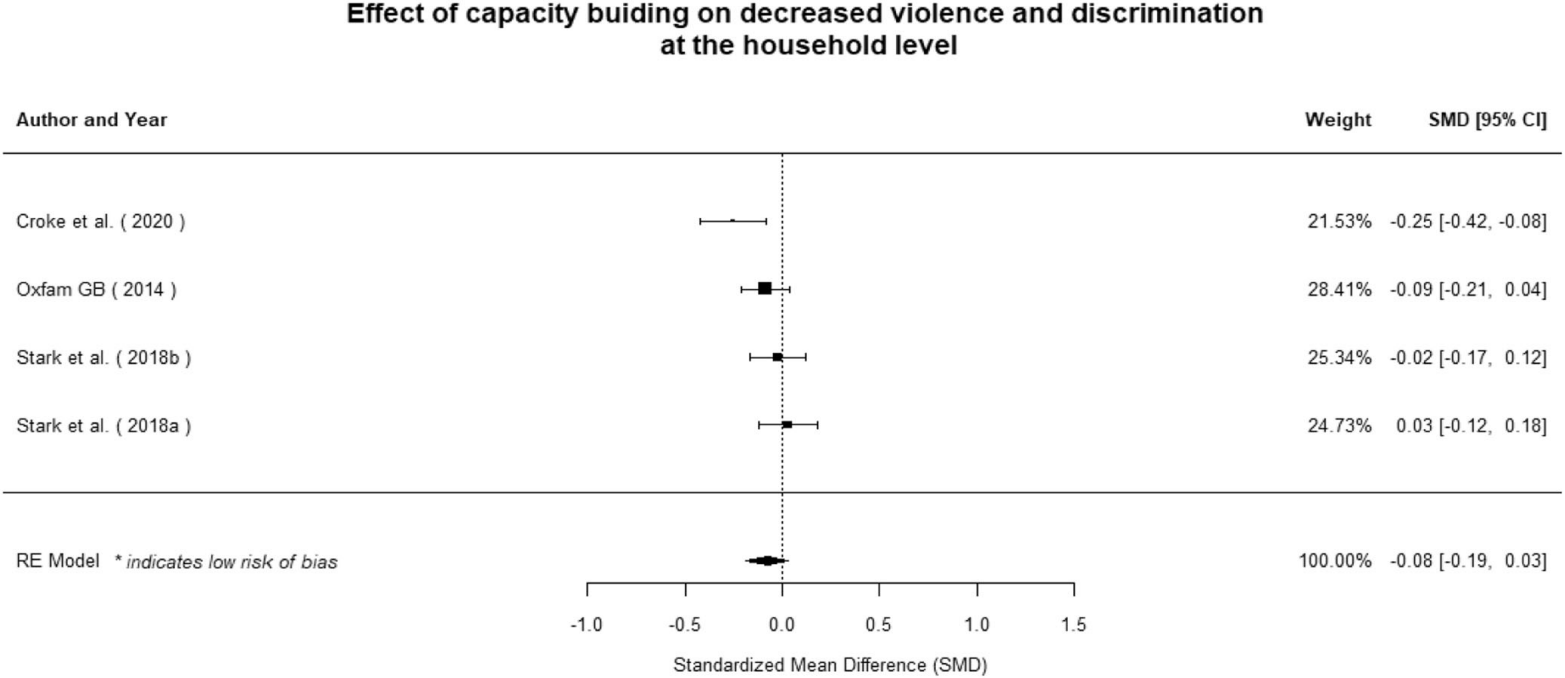
CBCB5: Forest plot showing the observed outcomes and the estimate of the random‐effects model. CI, confidence interval

We included a total of k=4 studies in the analysis. We assessed none of the studies as low risk of bias, two as some concerns, and two as high risk of bias. The observed outcomes ranged from −0.25 to 0.03. The estimated average outcome based on the random‐effects model was $\hat{\mu }=-0.08$ (95% CI: −0.19 to 0.03). Therefore, the average outcome did not differ significantly from zero (z=−1.38, p=0.17). A forest plot showing the observed outcomes and the estimate based on the random‐effects model is shown in Figure 161 (CBCB5). Stark, Seff, and colleagues (2018) refers to a study from Democratic Republic of Congo, while Stark, Asghar and colleagues (2018) refers to a study from Ethiopia, thus the samples are independent.

The Q‐test for heterogeneity was not significant, but some heterogeneity may still be present in the true outcomes (Q(3)=6.67, p=0.08, ${\hat{\tau }}^{2}=0.01$, $I^{2}=55.05$%), thus we still examined potential heterogenetiy. An examination of the studentised residuals revealed that none of the studies had a value larger than ±2.50 and hence there was no indication of outliers in the context of this model. According to the Cook's distances, none of the studies could be overly influential.

Effects from studies assessed as high risk of bias were not significantly different froms studies assessed as some concerns related to risk of bias ($\hat{B}=-0.16,p=0.07$ [95% CI: −0.32 to 0.01]). Exposure to intervention in months ($\hat{B}=-0.001,p=0.79$ [95% CI: −0.01 to 0.01]) and publication year ($\hat{B}=-0.01,p=0.65$ [95% CI: −0.08 to 0.05]) were also not significant. Finally, effects did not differ by level of gender inequality ($\hat{B}=-1.10,p=0.17$ [95% CI: −2.68 to 0.48]), nor by level of fragility ($\hat{B}=-0.005,p=0.54$ [95% CI: −0.02 to 0.01]).

We were not powered to test for moderation by country. In addition, only one study (Fuller, 2014) used a QED, and only one study targeted mixed genders. All studies measured the outcome immediately after the intervention was completed (thus the evaluation period was 0 for all studies), and all models were adjusted for covariates.

*Effects of capacity building on safer and more secure households and communities for women and girls*

**Figure 162:**
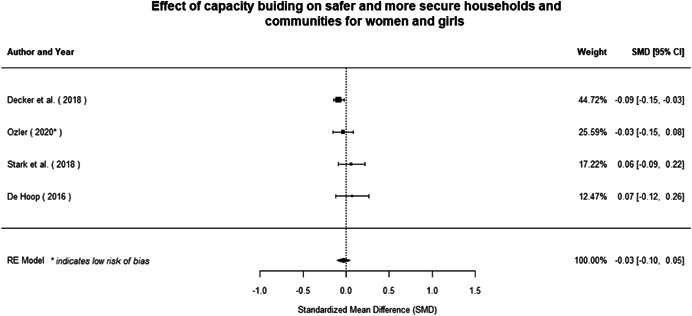
CBCB6: Forest plot showing the observed outcomes and the estimate of the random‐effects model. CI, confidence interval

We included a total of k=4 studies in the analysis. We assessed one study as low risk of bias, two as some concerns, and one as high risk of bias. The observed outcomes ranged from −0.09 to 0.07. The estimated average outcome based on the random‐effects model was $\hat{\mu }=-0.03$ (95% CI: −0.10 to 0.05). Therefore, the average outcome did not differ significantly from zero (z=−0.71, p=0.48). A forest plot showing the observed outcomes and the estimate based on the random‐effects model is shown in Figure 162 (CBCB6).

According to the Q‐test, there was no significant amount of heterogeneity in the true outcomes (Q(3)=5.01, p=0.17, ${\hat{\tau }}^{2}=0.00$, $I^{2}=40.18$%), thus we did not test for moderation. An examination of the studentised residuals revealed that none of the studies had a value larger than ±2.50 and hence there was no indication of outliers in the context of this model. According to the Cook's distances, none of the studies could be overly influential.

*Effects of capacity building on reduced frequency of physical violence by an intimate partner*

**Figure 163:**
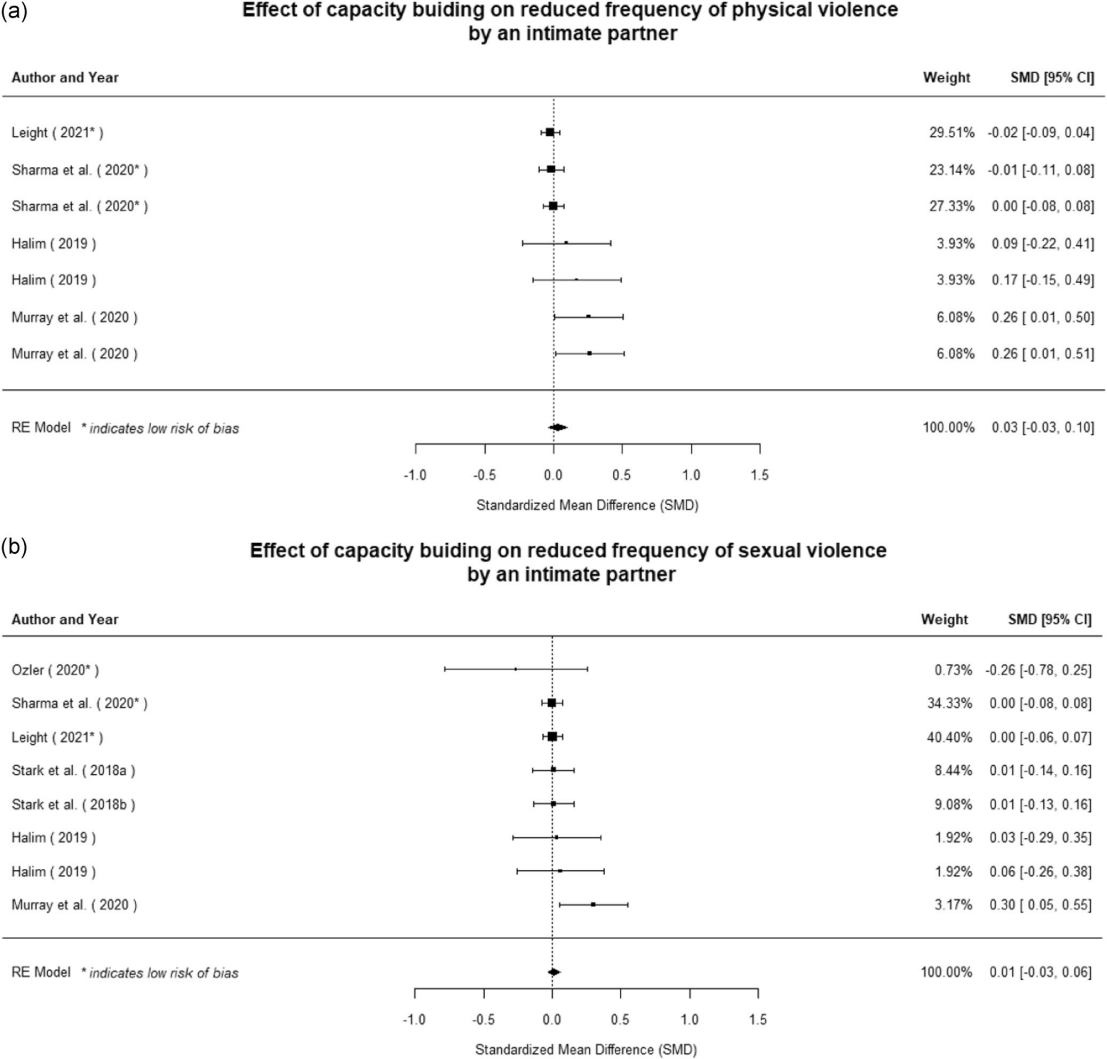
(a) CBCB7pv: Forest plot showing the observed outcomes and the estimate of the random‐effects model. (b) CBCB7sv: Forest plot showing the observed outcomes and the estimate of the random‐effects model. CI, confidence interval

We included a total of k=7 independent samples in the analysis. We assessed two studies as low risk of bias, none as some concerns, and two as high risk of bias. The observed outcomes ranged from −0.02 to 0.26. The estimated average outcome based on the random‐effects model was $\hat{\mu }=0.03$ (95% CI: −0.03 to 0.10). Therefore, the average outcome did not differ significantly from zero (z=0.93, p=0.35). A forest plot showing the observed outcomes and the estimate based on the random‐effects model is shown in Figure 163a (CBCB7pv). Three studies reported independently for men and women, thus we treated them as independent samples with separate control groups. These included Sharma and colleagues (2020; *g *=* *−0.01 for women and *g *=* *0.00 for men), Halim (2019; *g *=* *0.09 for men and *g *=* *0.17 for women), and Murray and colleagues (2020; *g *=* *0.26 for both women and men). Leight et al. (2021) and Sharma and colleagues (2020) were assessed as low risk of bias, while the other studies were assessed as high risk of bias.

According to the Q‐test, there was no significant amount of heterogeneity in the true outcomes (Q(6)=10.17, p=0.12, ${\hat{\tau }}^{2}=0.00$, $I^{2}=40.99$%), thus we did not test for moderation. An examination of the studentised residuals revealed that none of the studies had a value larger than ±2.69 and hence there was no indication of outliers in the context of this model. According to the Cook's distances, none of the studies could be overly influential.

*Effects of capacity building on reduced frequency of sexual violence by an intimate partner*

We included a total of k=8 studies in the analysis. The observed outcomes ranged from −0.26 to 0.30. The estimated average outcome based on the random‐effects model was $\hat{\mu }=0.01$ (95% CI: −0.03 to 0.06). Therefore, the average outcome did not differ significantly from zero (z=0.59, p=0.56). A forest plot showing the observed outcomes and the estimate based on the random‐effects model is shown in Figure 163b (CBCB7sv).

According to the Q‐test, there was no significant amount of heterogeneity in the true outcomes (Q(7)=6.47, p=0.49, ${\hat{\tau }}^{2}=0.00$, $I^{2}=0.00$%), thus we did not test for moderation. One study (Leight et al., 2021) had a relatively large weight compared to the rest of the studies (i.e., weight≥3/k, so a weight at least 3 times as large as having equal weights across studies). An examination of the studentized residuals revealed that none of the studies had a value larger than ±2.73 and hence there was no indication of outliers in the context of this model. According to the Cook's distances, none of the studies could be considered to be overly influential.

*Effects of capacity building on improved quality of relationships*

**Figure 164:**
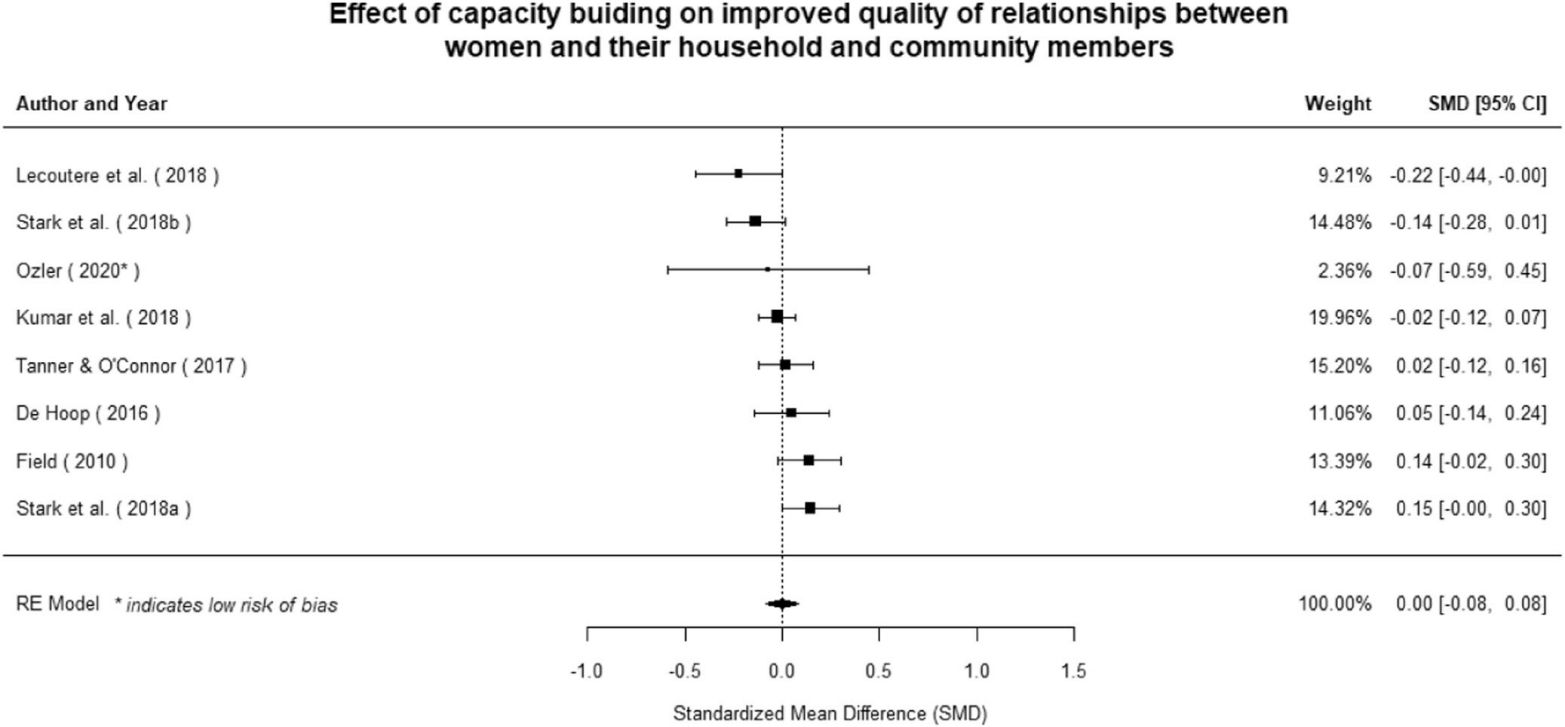
CBCB8: Forest plot showing the observed outcomes and the estimate of the random‐effects model. CI, confidence interval

We included a total of k=8 studies in the analysis. We assessed one study as low risk of bias, six as some concerns, and one as high risk of bias. The observed outcomes ranged from −0.22 to 0.15. The estimated average outcome based on the random‐effects model was $\hat{\mu }=0.00$ (95% CI: −0.08 to 0.08). Therefore, the average outcome did not differ significantly from zero (z=0.03, p=0.97). A forest plot showing the observed outcomes and the estimate based on the random‐effects model is shown in Figure 164 (CBCB8). Stark, Seff, and colleagues (2018) refers to a study from Democratic Republic of Congo, while Stark, Asghar and colleagues (2018) refers to a study from Ethiopia, thus the samples are independent.

According to the Q‐test, the true outcomes appear to be heterogeneous (Q(7)=14.33, p=0.05, ${\hat{\tau }}^{2}=0.01$, $I^{2}=51.14$%). An examination of the studentised residuals revealed that none of the studies had a value larger than ±2.73 and hence there was no indication of outliers in the context of this model. According to the Cook's distances, none of the studies could be overly influential.

Exposure to intervention in months ($\hat{B}=-0.001,p=0.75$ [95% CI: −0.007 to 0.005]) and evaluation period in months ($\hat{B}=0.01,p=0.52$ [95% CI: −0.03 to 0.06]) were not significant moderators of the effect. Nor was publication year ($\hat{B}=-0.02,p=0.14$ [95% CI: −0.05 to 0.01]). Effects also did not differ by scores on the GII ($\hat{B}=-0.50,p=0.62$ [95% CI: −2.45 to 1.46]) or the FSI ($\hat{B}=-0.01,p=0.13$ [95% CI: −0.01 to 0.002]).

Only one study was assessed as having high risk of bias (De Hoop 2016), thus we could not conduct sensitivity analysis by study quality. Only one model was not adjusted for covariates, and only one study did not take place in Africa. All studies used an experimental design.

*Effects of capacity building on women's empowerment index*

**Figure 165:**
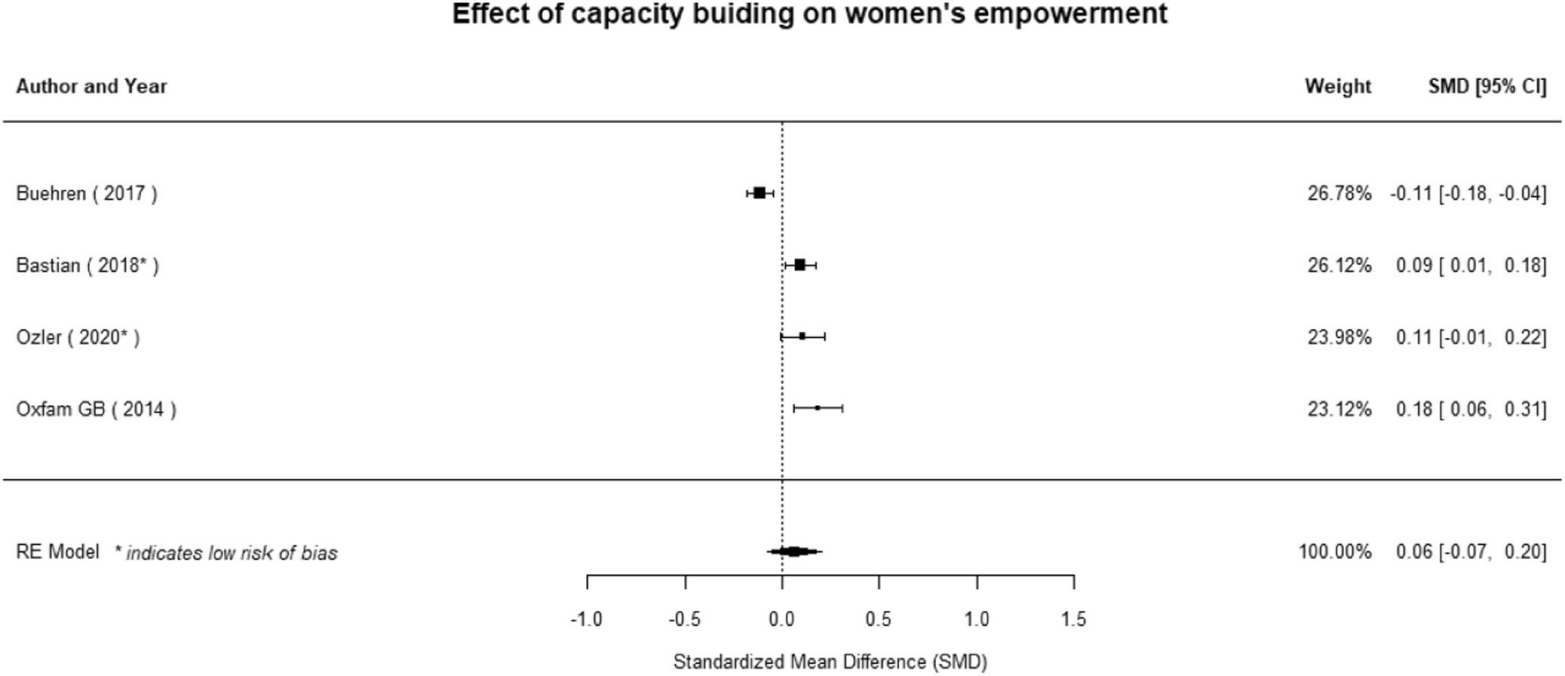
CBCC1: Forest plot showing the observed outcomes and the estimate of the random‐effects model

We included a total of k=4 studies in the analysis. We assessed two studies as low risk of bias, none as some concerns, and two as high risk of bias. The observed outcomes ranged from −0.11 to 0.18. The estimated average outcome based on the random‐effects model was $\hat{\mu }=0.06$ (95% CI: −0.07 to 0.20). Therefore, the average outcome did not differ significantly from zero (z=0.90, p=0.37). A forest plot showing the observed outcomes and the estimate based on the random‐effects model is shown in Figure 165 (CBCC1).

According to the Q‐test, the true outcomes appear to be heterogeneous (Q(3)=25.95, p<0.01, ${\hat{\tau }}^{2}=0.02$, $I^{2}=88.44$%). An examination of the studentised residuals revealed that one study (Buehren, Chakravarty, et al., 2017) had a value larger than ±2.50 and may be a potential outlier in the context of this model. According to the Cook's distances, none of the studies could be overly influential.

We were able to test several moderators in the context of this model. Studies with a high risk of bias were not significantly different than studies with low risk of bias ($\hat{B}=-0.04,p=0.61$ [95% CI: −0.18 to 0.11]). Exposure to intervention in months ($\hat{B}=-0.0001,p=0.99$ [95% CI: −0.01 to 0.01]) was not a significant source of heterogeneity, but evaluation period was ($\hat{B}=-0.02\;p<0.001$ [95% CI: −0.02 to 0.01]) such that smaller effects were observed as the time between the end of the intervention and data collection increased (specifically, the effect reduced by 0.02 standard deviation units for each month between the end of the intervention and the data collection). Publication year was not a significant source of variation among effects ($\hat{B}=-0.01\;p=0.78$ [95% CI: −0.08 to 0.06]). Finally, effects also did not differ by scores on the GII ($\hat{B}=0.40,p=0.43$ [95% CI: −0.60 to 1.41]) or the FSI ($\hat{B}=0.003,p=0.61$ [95% CI: −0.01 to 0.01]).

Only one study used a QED. In addition, all models were adjusted for covariates, all studies took place in Africa and all interventions target women only, thus none of these could be tested as potential sources of variation.

*Qualitative findings*

We conducted a thematic synthesis on the 16 linked qualitative studies to the included LSCB and capacity building interventions. As indicated above, this thematic synthesis aims to identify themes related to the interplay of intervention design, intervention implementation, target population, and contextual variables with intervention outcomes and effects. In total, we identified 27 descriptive themes, which we configured into six analytical themes (Supporting Information Appendix SN). These six analytical themes present the synthesis results and are discussed in more detail below.

*Theme 1: Contextual cultural norms determined the available space for LSCB capacity building*.

The qualitative synthesis identified a strong theme on the role of cultural norms across contexts. Norms on women's mobility, decision‐making in the household and gendered occupation mitigated the space in which LSCB capacity building could be designed and implemented (Gibbs et al., 2018; Lecoutere & Wuyts, 2021; Halim et al., 2019; Tanner & O'Connor, 2017a, 2017b). When content of LSCB programmes contrasted existing local norms, conflict often followed and closed the space in which the programme could operate. The qualitative evidence points to possible design strategies to mitigate this issue, which include adjusting content and targeted empowerment outcomes to reduce the gap with the prevailing norms (Gibbs et al., 2018; Tanner & O'Connor, 2017a, 2017b).

The ‘wall of patriarchy' is the metaphor used by Lecoutere and Wuyts (2021) to describe the barriers to women's decision‐making power within the household and to their empowerment. Evaluating the Gender Household Approach intervention in Uganda, a programme introducing a Participatory Intra Household Decision Making process via family counselling and couple seminars, and couple coaching, addressing entrenched patriarchal norms required a more structural programme design and approach to affect change. Women participants reported positive experiences with the programme overall, but also indicated no structural shifts in power in their household. They reported that ‘…if a woman's decision‐making power is restricted by the morals of the man, then yes, that is difficult to change […] Some women are better off because they cooperate more with their husbands. […] It came as a surprise that I acquired the power to make decisions over [strategic household] needs. My husband just changed’ (Lecoutere & Wuyts, 2021).

These findings were echoed by the Women for Women International's (WfWI) What Works to Prevent Violence Programme in Afghanistan. In this context, restrictive norms, particularly within religious structures, strongly constrained the programme space and potential to affect intended empowerment outcomes (Gibbs et al., 2018; Huber & Zupancic, 2015). Participants reported a lack of mobility and permission of male household heads to engage in income generating activities as the main barriers to their ability to apply newly gained skills in their daily lives. The qualitative evaluation concluded that ‘…women's lives remained structured by the wider constraints that they lived under’, and that the programme could have had a stronger impact if it was designed to engage family members and to address structural barriers at the household level (Huber & Zupancic, 2015).

We observed related themes in the Tanzanian context in the Together to End Violence Against Women (TEVAW) programme. Community level norms undermined efforts to encourage women's reporting of gender‐based violence (Halim et al., 2019). Furthermore, in the Congolese context with the implementation of the Opportunities through Mentoring, Parental Involvement and Safe Spaces (COMPASS) programme, implementers required careful awareness raising and negotiating to convince community leaders to accept the provided life skills content (Tanner & O'Connor, 2017a, 2017b). From a design perspective, the qualitative evidence suggested that restrictive norms and practices are difficult to change and need to be specifically targeted by dedicated programme components. The assumption that skills provision and awareness raising, for example, create spill‐over effects on empowerment outcomes was consistently rejected. More emphasis should be placed on design components that address the issue of restrictive norms and practices more directly based on a comprehensive and holistic concept of empowerment.

*Theme 2: Embedding the programme design with existing cultural norms and implementing through this embeddedness enhances acceptability and the space for programme implementation in restrictive contexts*.

Linked to the first theme was a rich set of data on the more strategic programme orientation that life skills interventions in restrictive contexts could explore. The themes in particular emerged from the programmes in Afghanistan and DRC referenced above (Huber & Zupancic, 2015; Tanner & O'Connor, 2017a, 2017b). They explored to what extent embedding programmes within existing restrictive contexts was strategic and defensible. Programme data from both contexts indicated that life skills programmes can enhance their reach in restrictive contexts by working with local actors that share existing social norms. This can refer to religious structures (in Afghanistan), existing sub‐societies (linked to FGM in DRC), and community leaders (in DRC and Tanzania).

Working with refugees living in camps on the Sudan/Ethiopia border, conflict‐affected communities in eastern Congo, and displaced populations in north‐west Pakistan, the COMPASS programme faced opposition from parents and community leaders in all countries. Participants and stakeholders questioned the appropriateness of the programme activities (Tanner & O'Connor, 2017a, 2017b). Consensus was achieved and implementation granted only through extensive discussion, house‐to‐house visits and negotiations with community leaders, local authorities and parents. Through trust and legitimacy, the programme was able to collaborate with leaders of ‘secret' societies associated with FGM, enhancing its reach to participants it would have not been able to access.

A similar theme emerged in the evaluation of the What Works to Prevent Violence Programme in Afghanistan, which pointed at the willingness to work with local religious institutions as a main facilitator to programme reach and impact (Gibbs et al., 2020; Huber & Zupancic, 2015). The evaluation illustrates that ‘…Women's rights are discussed from the Islamic perspective. With the training of mullahs, the WfWI team seeks to frame the discussions around women's rights as they relate to Islam, highlighting particular verses from the Quran that promote women's position in society. This can be viewed as generally in line with growing best practice in Afghanistan, where Islamic perspectives on women's rights are gaining ground among various stakeholders’.

However, a careful balance was struck to manage the level of integration of programmes and to guard against co‐option by these very structures. For instance, despite overall encouraging results, the COMPASS programme did not change women's own perceptions about restrictive norms. Women continued to hold attitudes that indicated their acceptance of gender inequality and SGBV. And, in the Afghanistan context, the programme resorted to paying men double the stipend it paid women, even though women were the main target beneficiaries. This led to questions around the programme's own commitment to gender equality (Huber & Zupancic, 2015).

*Theme 3: Ambiguous role of men in programme design*.

A key design question for life skills and capacity‐building refers to whether to create women‐only spaces or to also involve men as participants. We identified a mixed qualitative evidence base with data from different contexts and programmes, painting a rich picture (Gibbs et al., 2018; Lecoutere & Wuyts, 2021; Tanner & O'Connor, 2017a, 2017b). Overall, while women‐only programme designs were welcomed by women programme participants, programme implementers and decision‐makers emphasised that the lack of involvement of men in programmes impeded their impact. This appeared to be in particular the case in restrictive social settings where women are seen as unable to translate empowerment objectives in household decision‐making unless men are participating in the programme.

However, in the COMPASS programme, women participants across contexts reported a strong preference for single‐sex spaces. In Ethiopia, girls appreciated their protected space and even requested a further safe space which not even the life coach had access to. The safe space was seen as important in the refugee context and provided adolescent girls with a place to feel safe, learn and make friends. In addition, participants used it to report instances of SGBV and perceived strong ownership and a perception that men should not be allowed to partake. As the programmes progressed in each country, participants welcomed the inclusion of mothers and other caregivers (Tanner & O'Connor, 2017a, 2017b).

In contrast, data from the Uganda and Tanzania programmes reported programme limitations of not involving men participants. Lecoutere and Wuyts (2021) argue that any attempts of breaking the ‘Wall of Patriarchy’ requires the support and involvement of men. The programme, however, reported a lack of structural changes despite designing combined elements such as couple seminars and coaching to change intra household decision‐making; the qualitative data does illustrate this dilemma outlining different pathways to structural empowerment for women, all of which require the involvement and co‐operation of the men in the household. Likewise, a key finding of the evaluation of the What Works to Prevent Violence Programme in Afghanistan pointed to its limitation of only working with individual women. This was a key explanation for the lack of structural change in women's lives and recommended that ‘…engagement with male family members may prove a more effective means of facilitating women's social and economic empowerment’ (Huber & Zupancic, 2015).

*Theme 4: LSCB capacity building programmes and their design benefit from the inclusion of and coupling with TVET programmes*.

The qualitative evidence on programme design suggests integration of LSCB and TVET capacity building programme to be positive. The assessments of stand‐alone LSCB programmes and LSCB components within TVET programmes emphasised the interconnectedness of both types of skill sets. Programme participants expressed demand and need for both types of skills to enhance their life capabilities and opportunities. Within LSCB programmes, participants expressed a need for practical applications and a pathway for how new skills could lead to economic opportunities. Within TVET programmes, participants cited obstacles to the application of these skills to further their life being constrained by personal and structural issues.

In Uganda, women participants of a participatory intrahousehold decision‐making programme expressed a desire for agricultural skills, including farm management and cash crop cultivation, such as coffee. This knowledge was a means to economic development and contribute to shared household decision‐making and resource allocation (Lecoutere & Wuyts, 2021). In Afghanistan, participants had access to a range of TVET linked programme components, such as cash crop farming, animal husbandry, and embroidery within the wider empowerment programme. Participants regarded the start‐up capital and these elements as a main benefit (Gibbs et al., 2018). Likewise, in the COMPASS programme, career planning and business development were the highest rated training sessions.

This general theme of wanting to apply gained skills in daily contexts reflected a desire for practical tools and applications to further one's capabilities and personal development and growth. In combination with the qualitative data on TVET programmes, which mirror this theme, this points to the direction that capacity building design can benefit from intervention packages with multiple components aimed at structural change.

*Theme 5: Group and peer‐to‐peer design elements can target structural barriers to empowerment via social capital and collective action*.

The qualitative evidence indicated that life skills programmes that emphasised group and ‘peer‐to‐peer’ exchanges such as mentoring, and the formation of women‐only groups reported enhanced social capital and networks of women. Enhanced social capital can better target structural barriers to empowerment. This data emerged from the three‐country COMPASS programme which combined mentoring with parental involvement and safe spaces (Tanner & O'Connor, 2017a, 2017b) and the What Works to Prevent Violence Programme in Afghanistan, which created single sex networking spaces as part of the training programme (Gibbs et al., 2018; Huber & Zupancic, 2015).

In both programmes, themes centred on the benefit of having access to women peers for mentoring and learning from each other. This referred to both fellow programme participants and to the trainers and formal mentors provided by the programmes. The ability to learn from women in similar contexts and based on shared experiences and realities was a key benefit of the mentoring arrangements. In the COMPASS programme, mentors were carefully selected to be close in age to adolescent girl participants, from the same area, and to hold positive attitudes toward girls. In Afghanistan, women reported the programme to have been one of few opportunities to build a network outside of their families with one participant explaining that: ‘My life has improved. The year that I was in this course, when we came here once a week, we met other women and made friends, it was a good change for me’ (Gibbs et al., 2018).

In both programmes a transition from ‘shyness’ to ‘confidence’ was reported as women started to develop strong peer‐to‐peer relationships with each other and/or their mentors. In Ethiopia, this journey developed between mentor and mentees with both groups gaining in confidence as their relationship progressed, that is mentors, too, reported a growth in confidence and self‐worth from the success of the relationship and seeing their mentees progress. In Pakistan, women indicated their increased confidence to partake in household discussion and the interest of other women in their learning and growth. An illustration of this process was captured in this account: ‘When I go back to my home, I discuss what I learnt with people, and they praise me for being a scholar and worship what I have learnt while going to the course. It really gives great value to my talking and they stop working and listen while I am talking. All my friends, especially my sisters, have an interest in cow‐keeping. (…). In fact, they showed their interest in this course and further praised me a lot by saying: You are talented and have learnt so much so far’.

Finally, in both programmes, women indicated instances on how these peer‐to‐peer relationships might be able to grow into collective efforts to support each other. In the COMPASS programme, participants formed dedicated social networks that were observed to prevail beyond the programme. The participants reported stronger support networks, access to trusted nonfamily females, and a stronger sense of companionship. In addition, mentees provided mentoring services for the next cohort of participants in Ethiopia and DRC. In Afghanistan, women indicated forming social safety nets for each other by staying in touch post the intervention and supporting each other's business ventures. The evaluation concluded that ‘…the structure and approach to building social safety nets that brought women together in accepted spaces with the support of community leadership was in line with best practice in Afghanistan and appears to have facilitated opportunities for the effective development of safety nets for participants’ (Huber & Zupancic, 2015).

*Theme 6: A bundle of key implementation considerations apply across contexts*.

Across the LSCB programmes several implementation considerations were reported repeatedly as contributing factors to programme success. These implementation considerations are programmatic and granular. They are a bundle of considerations suggested across individual studies along the implementation cycle:

- Design and implement a sensitive and targeted awareness‐raising and outreach campaign to attract participants into the programme. For example, the COMPASS programme managed to build trust and legitimacy with the programme's constituencies through an in‐depth awareness raising campaign including house‐to‐house visits, community meetings, parents' meetings, and personal sessions with community and opinion leaders. This implementation factor is particularly important in restrictive social contexts.
- Identify and select a central and safe location that is easily accessible (e.g., via public transport, within walking distance of participants). For community acceptance, using an established and visible location can be a contributing factor. In Pakistan the training venue and safe space became more acceptable after it provided embroidery activities.
- The provision of childcare facilities was a highly desired aspect by programme participants and determined programme participation in Sudan/Ethiopia, DRC, Uganda and Pakistan.
- Training curricula and materials require careful adaptation to local context and translation into local languages. Participants of both the 3‐country COMPASS and What Works to Prevent Violence Programme in Afghanistan raised concerns about the lack of adapted materials and content.
- A key implementation element and driver of programme satisfaction was the skilfulness and approach of programme trainers. In the COMPASS programme, participants indicated positive feedback on the use of women as trainers in the single‐sex programme.
- Evaluation reports of a range of intervention outline the potential benefits of rapid programme iteration based on collected data. The COMPASS programme explicitly reported this as an applied implementation criterion. Sourcing ongoing participant feedback and consultation generated data that was used instantly to adapt programme design, for example identifying programme locations.

*Discussion*

The quantitative meta‐analysis revealed that LSCB interventions had positive significant effects on building women's life skills and women's capacity to understand and use financial, banking, and business serves effectively. In addition, positive significant but marginal effects were observed on women's participation in decision making at the household and community level; reduced agreement with reasons to justify violence against women and children; and women's awareness of their rights. The remainder of outcomes were nonsignificant.

Overall, the majority of community‐level outcomes, such as communities having more positive attitudes towards women, and being safer for women are nonsignificant and the same findings are established regarding empowerment outcomes such as decreased violence and discrimination at the household level and direct measures of women's empowerment. This is in contrast to individual outcomes related to women's skill development. The team used a complementary qualitative synthesis to explore explanations and recommendations to design more effective LSCB interventions that can go beyond narrow individual outcomes on skills development and targeting broader empowerment objectives and community level changes.

- A possible explanation for the limited effects on community level outcomes and downstream empowerment effects refers to the role of social norms and restrictive contexts that determine the space in which programmes can operate. The qualitative themes discussed in detail how programmes were limited in restrictive social contexts and not able to target a full spectrum of empowerment outcomes. In particular, programme implementer had to strike a careful balance between the depth of embedding programmes within restrictive context to be able operate and reach women and the adjustment of programme empowerment outcomes in order not too conflict with existing norms and practices too strongly. A reasonable observation is that the higher the level of embeddedness, the lower the scope to target radical empowerment outcomes in the short term (and in the evaluation timeframes). This does not negate the argument that this strategy translates into success in the medium‐ to long‐term and often was the only available programme path to at least improve women's' lives on a narrower scale related to individual skill development.
- A design implication from the qualitative themes on the programme limitations by restrictive contexts and norms is the need to develop complementary programme components that address these issues directly. More holistic programme design, involving multiple components addressing root causes as well as providing livelihood support and development, would be required for this from a design perspective. Specific suggestions from the qualitative evidence base points to programme components that foster women's peer‐to‐peer exchange and the building of a collective. This refers to designing mentoring elements into programmes and creating (or targeting existing) women's self‐help groups and clubs in which programme activities can be delivered. Such instruments seem to hold potential to build social capital, enhance women's networks, and to develop strategies for activism targeting broader empowerment outcomes.
- The qualitative evidence synthesis also identified themes around the role of men in programme implementation. While women themselves preferred single‐sex programmes in which men were not involved, programme implementers and decision‐makers identified the lack of men's engagement in programmes as a key barrier to empowerment outcomes and an explanation for why the positive effects on skill development did not translate into social change. We tested these qualitative findings against quantitative moderators on TVET and capacity building interventions but this was not supported by the quantitative data because of a lack of available data and this did not lead to significant results among the capacity building data.

##### 5.6.3.4 Summary of findings and discussion

We included 19 studies in 13 countries in South Asia and Sub‐Saharan Africa that evaluated the effect of life, social and livelihood skills and capacity building. We were able to examine effects on the following outcomes: Increased capacity of women to understand and use financial, banking, and business services effectively, women have increased access and ownership to assets, credit and income, women have more and better control over their bodies and sexual health, women are equipped with better life skills that allow them to be prepared for crisis or shocks and recover from them and women have improved attitudes, self‐image and confidence. Our included studies report against all of the secondary outcomes (Resources, Agency and Achievement) and eight of the nine immediate outcomes for our review. Overall, the GRADE assessments generally indicate a very low to low (with one moderate) certainty in this body of evidence.

Furthermore, the 16 identified qualitative linked studies provided six key analytical themes to buttress the quantitative findings. The success of LSCB programming is often determined by contextual and societal factors that can make or break the success of a programme. This means that embedding programming within these existing norms can enhance a community's acceptance of the offered programme, but at the same time limit the types of empowerment outcomes targeted. Similar to our findings in TVET interventions, group mentorship and peer exchange offer a route to collective action and socialisation that can encourage group uptake of a LSCB programme. Again, aligning with features of safe spaces programmes, LSCB programmes can see increased success when meeting spaces are secure and reachable by participants. Table 57 presents a summary of the quantitative results alongside our ratings of the certainty of the evidence (Table 52).

**Table 52:** GRADE summary of findings and certainty of evidence on life, social and livelihood skills and capacity building

Certainty assessment							Sample size	Effect	Certainty	Importance
No. of studies	Study design	Risk of bias	Inconsistency	Indirectness	Imprecision	Other considerations		Absolute (95% CI)		
*(AA1) Women have access to rights, services and opportunities*										
1	RCT	Serious^a^	Serious^b^	Not serious	Serious^c^	None	737	SMD 0.02 SD higher (0.12 lower to 0.16 higher)	⊕◯◯◯ VERY LOW	Limited importance
*(AB1) Increased capacity of women to understand and use financial, banking and business services effectively*										
3	RCT‐3	Serious^d^	Serious^e^	Not serious	Not serious	None	4991	SMD 0.14 SD higher (0.01 higher to 0.28 higher)	⊕⊕◯◯ LOW	Important, but not critical
*(AB2) Women have increased access and ownership to assets, credit and income*										
8	RCT‐6 QED‐2	Very serious^f^	Not serious	Not serious	Not serious	None	9970	SMD 0.09 SD higher (0.02 higher to 0.16 higher)	⊕⊕◯◯ LOW	Important, but not critical
*(AC2) Initiatives supported that facilitate women to access decent work (formal and informal employment), including people with disabilities*										
1	RCT	Very serious^h^	Serious^b^	Not serious	Not serious	None	3219	Two negative and three positive effect estimates with a 95% CI range of −0.08 to 0.16	⊕◯◯◯ VERY LOW	Important, but not critical
*(AC3) Improved capacity of women entrepreneurs*										
1	RCT	Very serious^i^	Serious^b^	Not serious	Not serious	None	3219	SMD 0.02 SD higher (0.09 lower to 0.05 higher)	⊕◯◯◯ VERY LOW	Important, but not critical
*(BA1) Women have improved success in the workplace*										
1	RCT	Not serious	Serious^b^	Not serious	Serious^j^	None	3543	Five positive effect estimates with a 95% CI range of −0.00 to 0.41	⊕⊕◯◯ LOW	Important, but not critical
*(BA2) Women have more and better control over their bodies and sexual health*										
7	RCT‐7	Serious^k^	Serious^l^	Not serious	Not serious	None	8359	SMD 0.06 SD higher (0.02 lower to 0.14 higher)	⊕⊕◯◯ LOW	Critical
*(BA3) Women have increased freedom of movement and association*										
3	RCT‐2 QED‐1	Very serious^m^	Serious^n^	Not serious	Not serious	None	5233	SMD 0.14 SD higher (0.02 higher to 0.26 higher)	⊕◯◯◯ VERY LOW	Limited importance
*(BA4) Women are more aware of their rights and the roles and responsibilities of duty bearers*										
5	RCT‐5	Serious^o^	Not serious	Not serious	Not serious	None	6565	SMD 0.06 SD higher (0.01 higher to 0.11 higher)	⊕⊕⊕◯ MODERATE	Limited importance
*(BA5) Women have more positive attitude towards taking action to claim their rights*										
1	RCT	Serious^p^	Serious^b^	Not serious	Not serious	None	4278	Six positive effect estimates with a 95% CI range of −0.07 to 0.19	⊕⊕◯◯ LOW	Limited importance
*(BA6) Reduced percentage of women agreeing with certain reasons that justify violence against women and girls*										
5	RCT‐4 QED‐ 1	Serious^q^	Serious^r^	Not serious	Not serious	None	4736	SMD 0.1 SD higher (0.01 higher to 0.35 higher)	⊕⊕◯◯ LOW	Critical
*(BA7) Women are equipped with better life skills that allow them to be prepared for crisis or shocks and recover from them*										
4	RCT‐4 QED‐1	Very serious^s^	Not serious	Not serious	Not serious	None	6750	SMD 0.16 SD higher (0.11 higher to 0.20 higher)	⊕⊕◯◯ LOW	Important, but not critical
*(BA8) Reduced instances of child or forced marriage*										
4	RCT‐3 QED‐1	Very serious^t^	Serious^u^	Not serious	Serious^v^	None	5178	SMD 0.1 SD higher (0.11 lower to 0.3 higher)	⊕◯◯◯ VERY LOW	Critical
*(BB1) Increased participation in decision making by Women at the household or community level, including during crisis response*										
9	RCT‐8 QED‐1	Serious^w^	Not serious	Not serious	Not serious	None	14,914	SMD 0.03 SD higher (0.0 higher to 0.07 higher)	⊕⊕⊕◯ MODERATE	Limited importance
*(BB2) Women participate more in their community*										
2	RCT‐1 QED‐1	Very serious^x^	Not serious	Not serious	Not serious	None	3203	SMD 0.02 SD lower (0.15 lower to 0.1 higher)	⊕⊕◯◯ LOW	Limited importance
*(CA8) Increased community support for women's and children's human, economic and legal rights*										
1	RCT	Very serious^ab^	Serious^b^	Not serious	Serious^ac^	None	968	SMD 0.07 SD lower (0.2 lower to 0.05 higher)	⊕◯◯◯ VERY LOW	Important, but not critical
*(CB1) Increased awareness in communities of the issues affecting women*										
1	RCT	Serious^y^	Serious^b^	Not serious	Serious^aa^	None	614	SMD 0.01 SD lower (0.17 lower to 0.15 higher)	⊕◯◯◯ VERY LOW	Limited importance
*(CB2) Communities have a more positive attitude towards women/marginalised groups*										
4	RCT‐4	Serious^ad^	Not serious	Not serious	Not serious	Publication bias strongly suspected	3872	SMD 0.07 SD higher (0.01 lower to 0.15 higher)	⊕⊕◯◯ LOW	Limited importance
*(CB3) Women have improved attitudes, self‐image and confidence*										
6	RCT‐5 QED‐1	Serious^ae^	Very serious^af^	Not serious	Serious^ag^	publication bias strongly suspected	10,481	SMD 0.03 SD higher (0.06 lower to 0.13 higher)	⊕◯◯◯ VERY LOW	Limited importance
*(CB4) Improved attitudes and increased support for women's economic, social and human rights by men, household and family members and community members*										
5	RCT‐5 QED‐1	Serious^ah^	Not serious	Not serious	Not serious	None	5122	SMD 0.06 SD (0.0 lower to 0.12 higher)	⊕⊕⊕◯ MODERATE	Limited importance
*(CB5) Decreased violence/discrimination at the household level*										
4	RCT‐3 QED‐1	Very serious^ai^	Serious^aj^	Not serious	Not serious	None	3438	SMD 0.08 SD lower (0.19 lower to 0.03 higher)	⊕◯◯◯ VERY LOW	Critical
*(CB6) Safer and more secure household, communities and areas/territories for women, girls, men and boys*										
4	RCT‐4	Serious^ak^	Not serious	Not serious	Not serious	publication bias strongly suspected	6563	SMD 0.03 SD lower (0.1 lower to 0.15 higher)	⊕⊕◯◯ LOW	Critical
*(CB7) Reduced frequency and distribution of types of violence by an intimate partner—physical violence (PV)*										
7	RCT‐7	Serious^al^	Serious^am^	Not serious	Not serious	Publication bias strongly suspected	6964	SMD 0.03 SD higher (0.03 lower to 0.1 higher)	⊕◯◯◯ VERY LOW	Critical
*(CB7) Reduced frequency and distribution of types of violence by an intimate partner—sexual violence (SV)*										
7	RCT‐7	Serious^an^	Not serious	Not serious	Serious^ao^	Publication bias strongly suspected	7809	SMD 0.02 SD higher (0.02 lower to 0.07 higher)	⊕◯◯◯ VERY LOW	Critical
*(CB8) Improved quality of relationships between women and their household and community members*										
7	RCT‐7	Serious^ap^	Serious^aq^	Not serious	Not serious	None	7036	SMD 0.00 SD higher (0.08 lower to 0.00 higher)	⊕⊕◯◯ LOW	Limited importance
*(CC1) Empowerment/Equality Index*										
4	RCT‐3 QED‐1	Serious^ar^	Very serious^as^	Not serious	Serious^at^	None	7690	SMD 0.06 SD higher (0.07 lower to 0.2 higher)	⊕◯◯◯ VERY LOW	Important, but not critical

Abbreviations: CI, confidence interval; GRADE, Grading of Recommendations, Assessment, Development and Evaluations; QED, quasi‐experimental design; RCT, randomised controlled trial; SMD, standardised mean difference.

^a^ Downgraded once due to uncertainty around bias relating to deviation from intended intervention.

^b^ All single studies downgraded once for inconsistency.

^c^ Downgraded because of very wide CIs that cross both sides of the threshold.

^d^ Downgraded once because one of the three studies is of high risk for bias, and another is unclear.

^e^ Downgraded once because point estimates vary considerably, and two of the studies' CIs do not overlap.

^f^ Downgraded twice because half of the body of evidence comes from high risk of bias studies with issues related to selection bias and deviations from intended bias.

^g^ While some CIs do not overlap, the point estimates are generally consistent and near to the effect estimate of the meta‐analysis.

^h^ Downgraded to very serious because of concerns related to selection bias and deviation from intended intervention.

^i^ Downgraded to very serious because of concerns related to selection bias and deviation from intended intervention.

^j^ Downgraded once to reflect the wide range of point estimates for this outcome within this study (0.06 to 0.35).

^k^ Downgraded once because there is uncertainty in one or more criteria across seven out of eight studies. That said, only one is rated as high risk of bias. If the high risk of bias study is removed, there is only a marginal change in the effect size.

^l^ Downgraded once because there are studies on both sides of the threshold whose CI do not overlap. That said, this larger body of evidence is mostly near the null hypothesis.

^m^ Downgraded twice because all three included studies are high risk of bias, including across multiple criteria.

^n^ In cognizance of the high risk of bias, this group was downgraded once because point estimates vary considerably and confidence intervals do not overlap for two studies.

^o^ Downgraded once because three of five studies present some risk of bias, and one is of high risk of bias. There are issues with deviation from intended intervention found in all studies.

^p^ Downgraded once due to uncertainty around bias relating to selection bias.

^q^ Downgraded once because three of five studies present some risk of bias, and one is of high risk of bias.

^r^ Downgraded once because all but one study report positive effect sizes. The outlier study is of high risk of bias, but does have considerable CI overlap with other studies.

^s^ Downgraded to very serious because all but one study presents risk of bias and half of the body of evidence is coming from high risk of bias studies. Selection bias and deviation from intended intervention are of serious concern in three of four studies.

^t^ Downgraded to very serious because all studies in this group present of bias, and half of the body of evidence is from high risk of bias studies. Deviation from intended intervention is prevalent.

^u^ Downgraded because one study has a point estimate that is very different than the others and has no overlap of CIs.

^v^ Downgraded because of wide CI's that cross over the threshold of interest widely on both sides.

^w^ Downgraded once because, despite two low risk of bias studies, six of eight present some risk, including two high risk of bias studies which both have significant issues related to deviation from intended intervention.

^x^ Downgraded twice because both studies included in this group are of high risk of bias, with particular concerns for deviations from intended interventions.

^y^ Downgraded once due to uncertainty around bias relating to deviation from intended intervention.

^aa^ Downgraded because of very wide CIs that cross both sides of the threshold.

^ab^ Downgraded to very serious because of concerns related to selection bias and deviation from intended intervention.

^ac^ Downgraded because of very wide CIs that cross both sides of the threshold.

^ad^ Downgraded once because two studies have risk of bias, and one has high risk. Both of the higher risk studies present reason for concern with respect to deviation from intended intervention.

^ae^ Downgraded once since although five of the six studies are RCT the QED is high risk and four RCTs raise some concerns related to risk of bias.

^af^ Downgraded twice because (1) effects on both side of the thresholds and CI not overlapping, (2) $I^{2}=77.89\%$ and *Q*‐test indicate the true outcomes is heterogeneous.

^ag^ downgraded once because *p* = .49 although sample size >400.

^ah^ Downgraded once because two studies present high risk of bias, with particular concerns for deviation from intended intervention.

^ai^ Downgraded twice because all studies present some risk of bias and two are of high risk. All studies are either unclear or of high risk of bias for deviations from intended interventions.

^aj^ While there is overlap of all studies' CIs, one study has a point estimate that varies considerably from the others.

^ak^ Downgraded because two studies at rated as some concern for risk of bias, while one is a high risk of bias with considerable deviation from intended intervention.

^al^ Two of four studies in this group are rated as high risk of bias with particular concern for deviation from intended intervention and performance bias.

^am^ While all studies are somewhat near to the threshold, several estimates are dissimilar to the others and have large CIs.

^an^ Downgraded once because although all studies are RCTs two of them are high risk of bias and two raise some concerns related to risk of bias.

^ao^ Downgraded once because although CI is small *p* = .34.

^ap^ While only one study is of high risk of, all but one have criteria of uncertain risk of bias. There is a particular risk with respect to deviation from intended intervention.

^aq^ Downgraded in cognizance of the wide range of point estimates (on both sides of the threshold) and confidence intervals that do not overlap.

^ar^ Downgraded once since the sample is a mix of RCT and QED and one RCT and one QED are high risk of bias while the two others are low risk.

^as^ Downgraded twice since (1) studies are on both side of the threshold and the CI are not systematically overlapping. (2) $I^{2}=88.44\%$ and *Q*‐test highlights the true outcomes appear to be heterogeneous.

^at^ Downgraded because although sample size >400, *p* = .37.

## 6 SUMMARY OF FINDINGS AND CONCLUSIONS

### 6.1 Summary of findings

#### 6.1.1 Search results

Following the adoption of UNSCR 1325 and the development of the WPS, there is an increased understanding of the gendered nature of conflict. This focus on women and their empowerment in fragile and conflict affected situations is at the core of the ToC of our SR.

**Figure 166:**
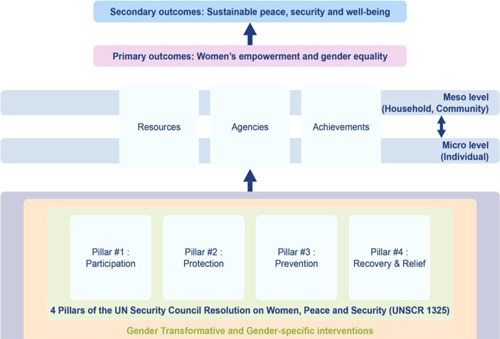
Systematic review theory of change

Our ToC is based on the recognition of the importance of the status of women in FCAS: women and girls are targets of war, are particularly vulnerable in fragile contexts and are affected by both fragility and conflict in unique ways ((United Nations Security Council, 2000). Women and girls, however, are not always passive, but can instead be powerful agents of change when they are directly involved in peacebuilding processes (Cockburn & Žarkov, 2002) and as observed in our review with interventions such as reconciliation and intergroup dialogues or community‐driven development. Thus, as per our ToC and observed in our findings, addressing women's needs and acting towards gender equality contributes to peace and inclusivity (Zuckerman & Greenberg, 2004). Prior evidence suggests that ‘countries with significant gendered inequality are more likely to prioritize violent masculinities and thus experience increased levels of organised violence’ (Klugman et al., 2021, p. 3). The recognition of the link between women's empowerment in FCAS and its contribution to peaceful and inclusive societies has been the driving principle of our review (as per our ToC in Figure 166): this contribution is done both through the role of women as agent of peace and the indirect effect of their empowerment on changing social norms towards more peaceful societies.

We have conducted a large and comprehensive SR on the effects of interventions to address women's empowerment and gender equality in FCAS. This review was based on the analysis of interventions under the four pillars of UNSCR 1325: Participation, Protection, Prevention, Recovery and relief. We identified 104 studies evaluating the effects of 55 programmes and 14 intervention types across three dimensions of women's empowerment, namely resources, agency and achievement. Our results suggest that most interventions have an overall positive effect on beneficiaries across one or more of these empowerment domains, and that interventions tend to have their largest effects on the intervention target, and often smaller or null effects on outcomes further along the causal chain. Table 58 presents an overview of the results of the quantitative synthesis for outcomes rated as critical or important where we had sufficient evidence to conduct a synthesis. Green boxes indicate significant positive effects and yellow boxes indicate nonsignificant effects. White or empty boxes indicate that there were no studies that met our inclusion criteria, while grey boxes indicate only one study was included, meaning that synthesis was not possible. The full table is presented in Supporting Information Appendix SQ.

There is a nexus at play: women's empowerment as a driver of peace and women as agents of peace (United Nations Security Council, 2000). Empowerment is a driver of peace as, by providing women with resources and agency, women's empowerment can lead to a change in social structures and norms contributing to more peaceful societies. Women can also be directly involved as peace stakeholders and actors sitting at the negociation table and acting for peace (United Nations Security Council, 2000). Putting the emphasis on only one of those elements will not enable long term and sustainable change since women need to be empowered to act as agents of peace and this role of agent will contribute to their empowerment in a virtious cycle. However, our review directly identifies only a few interventions that focus on the roles of women as agents of change, while a vast majority of interventions focus on their empowerment, demonstrating the gap in the evidence base on interventions directly focusing on women as agents of peace.

#### 6.1.2 Overview of effects

##### 6.1.2.1 Positive effects on targeted primary outcomes

Included interventions often have a positive effect on the primary outcomes they target (see Table 58). For example, in asset transfers (SMD* *=* *0.34, [95% confidence interval (CI): 0.22 to 0.46]) and cash transfers (SMD* *=* *0.22, [95% CI: 0.12 to 0.31]) we observe the largest effects on women's access to and ownership of assets, credit and income, while Village Savings and Loan Associations (SMD* *=* *0.24 [95% CI: 0.09 to 0. 39]) have their largest effects on women's capacity to use and understand financial, banking and business services effectively. Similarly, for life skills and capacity building programmes we find the largest effects on improved life skills (SMD* *=* *0.16 [95% CI: 0.11 to 0.20]), while inclusive community‐driven development has the largest effects on increased representation of women in local and subnational civil and political processes (SMD* *=* *0.28 [95% CI: 0.13 to 0.43]). Finally, interventions that focus on intra‐group dialogue have their largest effects on household and community support for women's economic, social, and human rights (e.g., community dialogues and reconciliations (SMD* *=* *0.18 [95% CI: 0.06 to 0.30]) and discussion groups (SMD* *=* *0.23 [95% CI: 0.05 to 0.39])). We find a paucity of evidence around AWPSs and community‐based services, neither of which have sufficient evidence to conduct a synthesis. We do not observe negative effects from any intervention on any of the main empowerment outcomes.

##### 6.1.2.2 Null effects on downstream outcomes

We find that most interventions do not achieve positive and significant effects for ‘downstream’, behavioural outcomes. For example, despite intimate partner violence (IPV) outcomes being included in seven bodies of evidence and six meta‐analyses, the nonsignificant results of included programmes on IPV outcomes are all very close to zero and even slightly negative in some cases. This finding suggests that the types of interventions included in this review are not affecting IPV, either because the causal chain is too complex and long for single interventions to realistically effect change, or because the programmes were not designed in such a way as to effectively target the outcome. Similarly, cash transfer programmes increase women's access to resources, but by and large they do not have an effect on norms (Table 53).

**Table 53:** Quantitative findings

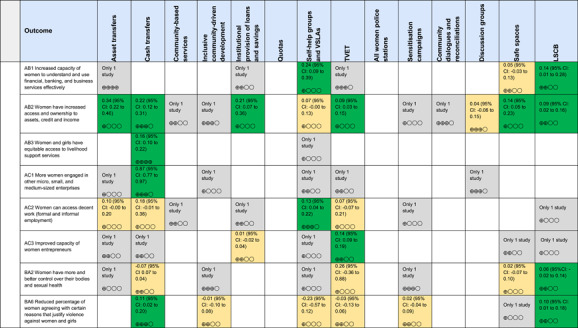
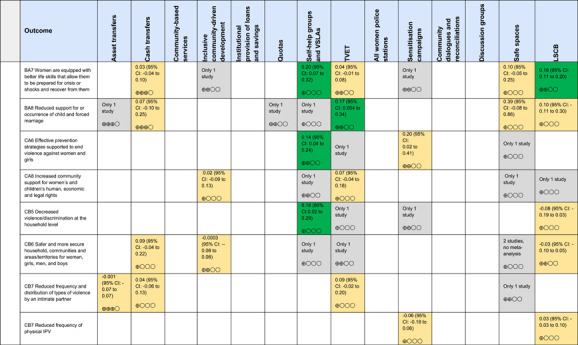
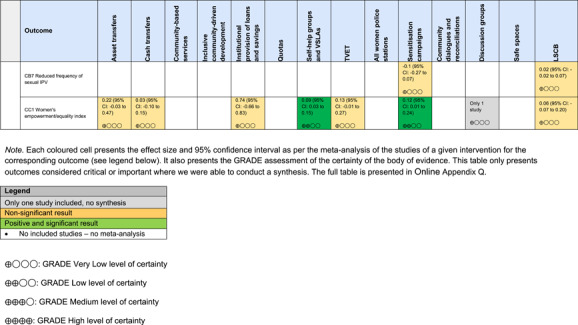

#### 6.1.3 Overview of qualitative findings and key considerations

We conducted thematic synthesis for eight intervention groups which includes over 90 linked qualitative studies. The other six groups do not have sufficient studies to conduct a qualitative synthesis. These 90 papers cover 53 unique programmes across our evidence base and provide a rich complement to our quantitative findings by generating 149 descriptive themes and 37 analytical themes. These interventions are funded by USAID, DFID/FCDO, the World Bank Group, the UN agencies, the Bill and Melinda Gate Foundation, the European Commission, National Governments and other foundations. They are implemented by organisations such as Oxfam, Care International, The International Initiative for Impact Evaluation (3ie), and various Universities, Academics and multilateral agencies.

##### 6.1.3.1 Multidimensionality of effects (or lack thereof)

Many interventions have positive effects on primary outcomes, but do not have substantial effects across multiple dimensions of empowerment. Although Kabeer's (1999) framework of women's empowerment suggests that the three dimensions of empowerment—resources, agency, achievement—are inextricably linked and interdependent: resources contribute to agency, contributing to achievement, then contributing to more resources, and so on, we observe that the effects of most interventions do not demonstrate this kind of inter‐dimensionality.

That said, there are some intervention types that positively and significantly affect outcomes across multiple dimensions. Cash transfers, self‐help groups, and TVET programmes produce beneficial effects across all three dimensions, while asset transfers, sensitisation campaigns, and capacity building programmes show positive and significant effects across two of the three dimensions: resources and agency for asset transfers and capacity building programmes, agency and achievements for sensitisation campaigns.

We also found examples of interventions that, in addition to their effect on immediate outcomes, affect outcomes further along the causal chain, such as women's increased participation in decision making, improved attitudes towards women, and less agreement with reasons justifying SGBV. These multidimensional effects are generally smaller in magnitude compared to those related to the main target of the intervention.

##### 6.1.3.2 The importance of social norms and context

We consistently find that norms and restrictive social contexts act as a barrier to improving women's empowerment in the programmes evaluated. Social norms and contexts related to gender determine the space in which interventions operate and attempt to institute positive changes in women's lives. Within programme design and implementation, restrictive norms and social structures block pathways to change and limit intervention effects. The influence of entrenched norms and societal structures can inhibit the ability of interventions to effect women's empowerment in a multi‐dimensional way despite norms' ability to affect targeted, primary outcomes such as enhanced skills and women's representation. This implies that, where the realisation of targeted intervention outcomes depends on the existence of facilitating norms and practices, interventions need to deliberately design programme components that target changing these norms and practices. This challenge is exemplified in our review, which found scant evidence on interventions' ability to change norms, and few interventions having large effects.

A challenging programme design and implementation decision is presented where interventions are taking place in highly restrictive social contexts with existing norms and practices at conflict with the empowerment outcomes targeted by interventions. The available evidence implies that programmes can only reach participants if they work through local agents and structures even though these might not share programme objectives and values.

##### 6.1.3.3 Intervention design and implementation

A key intervention design consideration is choosing whether to design single sex interventions or to opt for a mixed‐gender design approach. While qualitative evidence points to a strong preference by women participants for single‐sex programme designs, implementers and decision‐makers prefer a mixed‐sex programme approach. This was based on the precedence that underlying structural issues and power imbalances cannot be addressed unless men participate in programmes and contribute to joint solutions and activism for change. This observation from a qualitative perspective is, nonetheless, not corroborated by our quantitative data, which show no difference in the size of the effect between single‐sex and multi‐sex programmes where this was testable (e.g., TVET and capacity building).

Targeting the right beneficiaries is another key consideration for the design of programmes aimed at improving women's empowerment outcomes, especially for cash transfer, self‐help groups and Village Savings and Loan Associations and safe spaces interventions. Targeting practices can also act as a barrier to interventions' effectiveness when the process lacks transparency.

#### 6.1.4 Intervention‐specific findings

The following sections detail the summary of findings for each type of intervention.

##### 6.1.4.1 Asset transfer

Asset transfer interventions yield significant and positive effects on outcomes in two of the three dimensions of the resources‐agency‐achievement framework. Although asset transfers interventions target outcomes across all three dimensions of women's empowerment, they only produce positive and significant results on access to assets, credit and income (resources; SMD* *=* *0.34 [95% CI: 0.22 to 0.46], an estimated 13.3% change in the intervention group compared to the control group) and the participation in decision making (agency; SMD* *=* *0.07 [95% CI: 0.04 to 0.11] an estimated 2.8% change in the intervention group compared to the control group). We found no evidence that asset transfer interventions improve achievement‐related outcomes, such as improved self‐image and confidence, the reduction of intimate partner violence and the improved quality of relationships with community and households. The examination of potential sources of variation did not provide any conclusive findings due to a lack of sufficient data.

Qualitative findings suggest that the provision of assets or tools for cultivation or livestock farming can generally support asset endowment, creditworthiness and income. If assets are utilised productively, it opens pathways to credit use options and income‐generating activities. This was especially evident in multi‐component asset transfer programmes, such as the IGVGD programme in Bangladesh, which added a microfinance facility which allowed women to use loans for a multitude of purposes (Ahmed et al., 2007). Additionally, agency is bolstered when women are able to own and manage animals and/or crops. Women can then experience improved decision‐making and social status within households and around communities.

##### 6.1.4.2 Cash transfer

We find that cash transfers have positive effects on outcomes across the all three dimensions of the resources‐agency‐achievement framework. We observe improvements in access to assets, credit and income (SMD = 0.22 [95% CI: 0.12 to 0.31] an estimated 8.7% change in the intervention group compared to the control group), access to livelihood support services (SMD* *=* *0.16 [95% CI: 0.10 to 0.22] an estimated 6.4% change in the intervention group compared to the control group), engagement in enterprises (SMD* *=* *0.87 [95% CI: 0.77 to 0.97] an estimated 31.1% change in the intervention group compared to the control group), reduction of acceptability of violence (SMD* *=* *0.11 [95% CI: 0.02 to 0.20] an estimated 4.4% change in the intervention group compared to the control group) and increased political representation (SMD* *=* *0.13 [95% CI: 0.05 to 0.21] an estimated 5.2% change in the intervention group compared to the control group). However, we do not find any effects of cash transfer programmes on women's control over their bodies, freedom of movement and association, participation in decision making, better life skills, community support for women's needs, safer communities and reduced frequency of violence.

Many cash transfer programmes included additional components (e.g., a training component), which we hypothesised would be related to larger effects. However, our moderator analyses revealed no differences between the cash transfers alone and cash transfers plus an additional component on any of the women's empowerment outcomes. Similarly, we generally found no difference in the size of effects between conditional and unconditional cash transfers. The exception to this was women's participation in decision making, where we actually observed a significantly *smaller* effect of conditional transfers as compared to unconditional transfers.

Qualitative findings suggest that economic interventions should be tailored to both geographical and societal settings, including entrenched patriarchal norms. This is because gendered household and community roles often restrict women beneficiaries' ability to manage or decide how to spend money. Furthermore, implementation and community leadership are both critical pillars of influence in sensitising communities and households to participate in an intervention.

##### 6.1.4.3 Community‐based services

The effects of community‐based services on women's empowerment index outcomes are unknown. The one study that is included under this intervention group only examined outcomes related to access to and ownership of assets, credit and income (SMD* *=* *0.02, 95% CI [−0.05, 0.09] an estimated 0.8% change in the intervention group compared to the control group) and access to decent work (effects ranged from very small, negative effects (SMD* *=* *−0.02, 95% CI [−0.09, 0.05] an estimated −0.8% change in the intervention group compared to the control group) to very small, positive effects (SMD* *=* *0.02, 95% CI [−0.05, 0.09] an estimated 0.8% change in the intervention group compared to the control group). None of the effects of this single primary study reached statistical significance. Further, all of the evidence from the community‐based services intervention was found to be of low certainty, indicating that future studies of these interventions may produce different results.

##### 6.1.4.4 Inclusive community‐driven development

Inclusive community‐driven development (ICDD) only yields significant and beneficial effects in the achievement dimension of the Resources‐Agency‐Achievement framework. There are only positive and significant results on the increased political representation of women (SMD* *=* *0.28 [95% CI: 0.13 to 0.43] an estimated 11% change in the intervention group compared to the control group). The other outcomes targeted by ICDD programmes, such as freedom of movement and association, positive attitudes towards claiming rights, reduction of justification of violence, support from community on women's needs and the pursuit of safer communities, are not affected by the community‐driven development interventions. This echoes existing synthesis that found ICDD programmes use existing social cohesion rather than building it (White et al., 2018).

Based on qualitative evidence, representation within committees in terms of gender and background diversity as well as support and integration with local government structures emerge as key contributors to intervention success. Recognising embedded power dynamics also enables women to better participate in local and subnational political processes. However, entrenched patriarchal norms necessitate an increased buy‐in by these stakeholders such as local cultural or religious leaders for appropriate uptake at the community level. Regular and sustained interaction contributed to maintained uptake and long‐lasting effects at the community level.

##### 6.1.4.5 Institutional provision of loans and savings

We find institutional provision of loans and savings only have positive effects on resources, as measured by access to or ownership of assets, credit and income (SMD = 0.21 [95% CI: 0.07 to 0.36] an estimated 8.3% change in the intervention group compared to the control group), but fail to have significant and positive effects on the increased participation of women in decision‐making and the improved capacity of women entrepreneurs, and on women's empowerment index.

##### 6.1.4.6 Quotas

We are unable to provide strong conclusions regarding quotas due to the low number of included studies using quota interventions. Women's improved attitudes towards taking action to claim their rights was the only outcome with two studies contributing effects, but the synthesis found a null effect. This must be interpreted with caution given the very small number of included studies. Further, all of the evidence from the quota interventions was found to be of low certainty, indicating that future studies of quota interventions may produce different results.

##### 6.1.4.7 Self‐help groups and village savings and loans associations

We find that SHGs and VSLAs have positive effects on outcomes across the resources‐agency‐achievement framework, including capacity to use and understand financial and banking services (SMD* *=* *0.24 [95% CI: 0.09 to 0.39] an estimated 9.5% change in the intervention group compared to the control group), access to decent work (SMD* *=* *0.13 [95% CI: 0.04 to 0.22] an estimated 5.2% change in the intervention group compared to the control group), increased freedom of movement and association (SMD* *=* *0.18 [95% CI: 0.05 to 0.31] an estimated 7.1% change in the intervention group compared to the control group), rights awareness (SMD* *=* *0.10 [95% CI: 0.02 to 0.18] an estimated 4% change in the intervention group compared to the control group), positive attitudes towards taking action to claim rights (SMD* *=* *0.58 [95% CI: 0.03 to 1.14] an estimated 21.9% change in the intervention group compared to the control group), better life skills (SMD* *=* *0.20 [95% CI: 0.07 to 0.32] an estimated 7.9% change in the intervention group compared to the control group), representation in political processes (SMD* *=* *0.09 [95% CI: 0.04 to 0.14] an estimated 3.6% change in the intervention group compared to the control group), prevention of violence (SMD* *=* *0.14 [95% CI: 0.04 to 0.24] an estimated 5.6% change in the intervention group compared to the control group), decreased violence at the household level (SMD* *=* *0.16 [95% CI: 0.02 to 0.29] an estimated 6.4% change in the intervention group compared to the control group) and women's empowerment index (SMD* *=* *0.09 [95% CI: 0.03 to 0.15] an estimated 3.6% change in the intervention group compared to the control group). This type of intervention, however, fails to affect women's access to and ownership of assets, credit and income, reduction of justification of violence, participation in decision‐making, participation in the community, support from the community, more positive attitudes towards women, improved self‐image and confidence and better relations with community and household members.

The qualitative evidence base supports this; social capital gains and skill acquisition seems to directly augment economic‐focused outcomes rather than association and social‐focused outcomes. SHGs and VSLAs are an attractive and convenient option for women who have been systematically excluded from formal economic participation. However, similar to quantitative findings, other factors such as market access and conditions minimise the collective gains for other outcomes.

##### 6.1.4.8 Technical and vocational education and training

We find that TVET interventions have positive effects on outcomes across the three empowerment dimensions. This includes access to assets, credit and income (SMD* *=* *0.09 [95% CI: 0.03 to 0.15] an estimated 3.6% change in the intervention group compared to the control group), improved capacities of women entrepreneurs (SMD* *=* *0.14 [95% CI: 0.09 to 0.19] an estimated 5.6% change in the intervention group compared to the control group), reduction of forced and child marriage (SMD* *=* *0.17 [95% CI: 0.04 to 0.34] an estimated 6.7% change in the intervention group compared to the control group), political representation of women (SMD = 0.29 [95% CI: 0.03 to 0.55] an estimated 11.4% change in the intervention group compared to the control group) and improved image and self‐confidence (SMD* *=* *0.17 [95% CI: 0.01 to 0.33] an estimated 6.7% change in the intervention group compared to the control group). However, TVET fails to have significant and positive effects on women's control over their body, increased freedom of movement and association, reduced justification of violence, improved life skills, participation in decision making, community support, reduction of violence, quality of relationships, and women's empowerment index. Though we hypothesized that TVET would affect women's access to decent work, this was not supported by the evidence, which found a null effect of TVET programmes in fragile and gender unequal states on women's access to work. This is reflected in qualitative data as an effect of social norms since, although we observe a positive effect on capacities, this does not necessarily transform into employment for women. This is often due to conflating social norms that TVET interventions need to consider in varying contexts. Unless programmes are designed to address underlying norms that hinder women's participation in such a programme, there is likely little progress to be made regarding household power relations and women's empowerment within communities. For example, providing childcare and transportation stipends to women participants can offset the perception that they are abandoning their domestic work and offset the opportunity cost of attendance.

The qualitative evidence base indicates a need to design additional programme components within TVET to target outcomes beyond individual skill development and improved attitudes. Group mentorship and peer exchange offer a route to collective action and socialisation that can encourage group uptake of a life skills and capacity building programme. The most successful programmes in terms of uptake and outcomes were ones that enhanced the social aspect as well as the livelihoods aspect.

##### 6.1.4.9 AWPSs

We were unable to draw conclusions for AWPSs due to the low number of included AWPSs studies. The only included study found a significant negative impact on employment of women in law enforcement (as measured by the daily proportion of cases assigned to women (SMD = −0.45, 95% CI [−0.58, −0.32] an estimated 17.4% change in the intervention group compared to the control group), and a null effect on women having improved and equitable access to justice (SMD* *=* *0.07, 95% CI [−0.06, 0.19] an estimated 2.8% change in the intervention group compared to the control group). The evidence from AWPSs was found to be of low or very low certainty, indicating that future studies of AWPSs may produce different results.

##### 6.1.4.10 Sensitisation campaigns

Sensitisation campaigns have a beneficial effect on outcomes from two of the three dimensions of the women's empowerment framework. There is little data on resource outcomes from this type of intervention. Beneficial effects of sensitisation campaigns were found on women having more positive attitudes towards claiming their rights (SMD* *=* *0.18 [95% CI: 0.06 to 0.30] an estimated 7.1% change in the intervention group compared to the control group), increased awareness of issues affecting women (SMD* *=* *0.14 [95% CI: 0.02 to 0.25] an estimated 5.6% change in the intervention group compared to the control group) and women's empowerment index (SMD = 0.12 [95% CI: 0.01 to 0.24] an estimated 4.8% change in the intervention group compared to the control group). However, sensitisation campaigns fail to affect reduction of agreement with justification and occurrence of violence, improved self‐image, support from households and communities, reduction of physical and sexual IPV and improved quality of relationships with household and community members.

Based on the qualitative findings, sensitisation campaigns can be limited and restricted by social factors such as gender norms and roles. Programmes that, for example, worked with constructive religious and social messaging, often through community leaders in religious institutions, were able to act as a useful channel for disseminating information. Religious leaders and institutions acted as a conduit for participants to better understand gender imbalances and how to address them. Changes that are related to altering human behaviour through learning and communication often take time to realise.

Additionally, qualitative findings reveal that training that specifically targets men and boys is useful in building awareness on IPV and conflict resolution to advocate for men directly challenging gender norms. Training men to better understand women's roles is an important exercise in encouraging men to understand women's reality in the household and community.

##### 6.1.4.11 Community dialogues and reconciliations

We are unable to make strong conclusions related to community dialogue interventions given that only two studies were included. While the synthesis of the two studies found a positive and significant effect on an achievement‐related outcome: improved attitudes and support for women's economic, social and human rights by households and communities, this result should be interpreted with caution. With only two studies, we were also unable to examine any potential sources of heterogeneity. Further, we had a low level of certainty of this body of evidence, suggesting that future studies of community dialogue interventions may produce different results.

##### 6.1.4.12 Discussion groups

We are unable to make strong conclusions related to discussion group interventions given that only two studies were included. While the synthesis of the two studies found a positive and significant effect on an achievement‐related outcome: improved attitudes and support for women's economic, social, and human rights by households and communities, this result should be interpreted with caution as we have low certainty in the body of evidence related to this effect, suggestions that future studies may produce different results. With only two studies, we were also unable to examine any potential sources of heterogeneity.

##### 6.1.4.13 Safe spaces

Safe spaces only have a beneficial effect on the resource dimension of the empowerment framework through increased access to or ownership of assets, credit and income (SMD = 0.14 [95% CI: 0.05 to 0.23] an estimated 5.6% change in the intervention group compared to the control group). However, safe spaces fail to affect agency and achievement related outcomes in our sample of studies, including increased capacity of women to understand and use financial, banking, and business services effectively, better control over their bodies and sexual health, freedom of movement and association, support for or occurrence of child and forced marriage, participation in decision making by women at the household or community level, improved attitudes, self‐image and confidence, improved quality of relationships between women and their household and community members.

Although we would expect it to increase safe communities improve skills and capacity building and reduce violence, there was a null effect of safe spaces on these outcomes in fragile and gender unequal states. Contextual findings from qualitative synthesis show that uptake and participation are buttressed by the maintenance of physical space and planned activities to ensure safety of participants and implementing partners. Including mentorship as a key aspect provides participants and their family members with confidence in dismantling harmful social norms related to the rights of women and girls.

Additionally, targeting women or girls in particular age brackets proves to be successful. For example, a programme that targeted adolescent girls of marriageable age showed success in preventing early and forced marriage in the target community by combating harmful social and religious beliefs that pressured or forced these girls into marriage.

##### 6.1.4.14 Life, social, and livelihood skills and capacity building

Life, social, and livelihood skills and capacity building (LSCB) interventions result in positive and significant effects on outcomes within two of the three dimensions of the resources‐agency‐achievement framework. We find beneficial effects of LSCB programmes on both resources and agency outcomes. Specifically, understanding and use of financial and business services (SMD = 0.14 [95% CI: 0.01 to 0.28] an estimated 5.6% change in the intervention group compared to the control group), better control over their body and sexual health (SMD = 0.06 [95% CI: 0.02 to 0.14] an estimated 2.4% change in the intervention group compared to the control group), freedom of movement and association (SMD = 0.14 [95% CI: 0.02 to 0.26] an estimated 5.6% change in the intervention group compared to the control group), awareness of rights (SMD = 0.06 [95% CI: 0.01 to 0.11] an estimated 2.4% change in the intervention group compared to the control group), reduced agreement with justification of violence (SMD = 0.10 [95% CI: 0.01 to 0.18] an estimated 4% change in the intervention group compared to the control group), equipment with life skills (SMD = 0.16 [95% CI: 0.11 to 0.20] an estimated 6.4% change in the intervention group compared to the control group), participation in decision‐making (SMD = 0.03 [95% CI: 0.001 to 0.07] an estimated 1.2% change in the intervention group compared to the control group). However, LSCB interventions fail to effect access to and ownership of assets, credit, and income, reduction of child and forced marriage, participation in and support from the community, reduction of occurrence of violence, quality of women's relationships and women's self‐image and confidence.

The qualitative findings suggest that the success of life skills and capacity building programming is often determined by contextual and societal factors that can make or break the success of a programme. This means that embedding programming within these existing norms can enhance a community's acceptance of the offered programme. In contrast, the more embedded a programme is, the more difficult it may be to enact radical change challenging existing norms. Similar to our findings in technical and vocational education and training interventions, group mentorship and peer exchange offer a route to collective action and socialisation that can encourage group uptake of a life skills and capacity building programme.

### 6.2 Overall completeness of the evidence

The scope of this review is broad, assessing the effects of covering 14 different intervention types on three dimensions of empowerment. This means that while we reviewed a relatively large evidence base, this is generally spread thinly and we identify several important gaps. Specifically, for community‐based services (one included study), quotas (two included studies), community dialogues and reconciliations (two included studies), AWPSs (one included study) and discussion groups (five included studies) we identify few or no studies reporting on empowerment‐related outcomes. Further study of these intervention types is needed to provide any clear conclusions about the nature of their impact on women's empowerment, particularly in areas of conflict with high levels of gender inequality.

Considering the large number of extant interventions and IEs focusing on FCAS, there is a relatively low number of studies of interventions defined as gender‐specific or transformative. This is particularly true for interventions primarily focusing on the UNSCR recovery and relief pillar and on peacebuilding interventions in general. Although there is existing literature on peacebuilding processes, the vast majority of the available evidence is not gender‐specific or gender‐transformative and does not focus primarily on recovery and relief but more on participation, protection and prevention.

Our review covers evidence from 29 countries, but the geographical distribution of studies is uneven. We identified a large number of studies from Sub‐Saharan Africa and Southeast Asia. This is in line with the primary focus of our review on FCAS. However, the low number of studies from the Middle East and North Africa, Latin America and Central Asia indicates that the need for evidence is especially acute for these regions.

Finally, we conducted extensive targeted searches to identify qualitative studies, process evaluations and project documents associated with included experimental and quasi‐experimental studies to help us address our review questions. While we identified a number of relevant documents, the volume and quality of evidence limited the extent to which we are able to provide andomizedon findings at the intervention level to eight intervention types of the 14 identified. Additionally, six of the 14 intervention groups did not have sufficient qualitative evidence to conduct a synthesis.

### 6.3 Quality of the evidence

The review included studies that used andomizedon or other rigorous quasi‐experimental study designs to answer our review questions. About 74% of the included studies used a RCT design with randomly allocated treatments to either individuals or clusters and the remaining studies used QEDs. Among the QED studies, only 3% had an overall low risk of bias, while 45% had high risk of bias and 52% were assessed as some concerns. The quality of studies using RCT designs was relatively higher, with 28% of the studies assessed as low risk of bias and 28% of studies assessed as high risk of bias.

The results of GRADE ratings of each analysis included in this review demonstrate a varied certainty in the body of evidence. Of the analyses performed, 57% are found to be of very low certainty, 27% of low certainty, 14% of moderate certainty, and 2% of high certainty. While the reasons for low certainty vary across bodies of evidence, the most frequent reasons that certainty of evidence was downgraded are risk of bias of studies and inconsistency of effect sizes of studies included in meta‐analyses. These judgments consider the risk of bias of all individual studies included in a body of evidence, and some decisions are made based on metrics from the outputs of meta‐analyses themselves. See page 52 for further details on the GRADE approach.

Details on context, intervention design and implementation were sometimes missing, which made it difficult to assess what was delivered, by whom and their effects. Although most of the manuscripts reported sample characteristics, standard errors and standard deviations, some studies were missing details on the exact sample size or precision estimates. We contacted authors to collect missing data, but in the absence of responses, effects with missing or unclear data could not be included in the analysis.

In addition, a substantial number of studies met the geographical and thematic selection criteria but failed to meet the experimental and methodological criteria. This demonstrates that, although the evidence base is growing, there is a need for more rigorous methodological approaches using counterfactuals to measure women's empowerment and gender equality outcomes in fragile and conflict affected situations.

### 6.4 Agreement and disagreement with other studies

The 14 types of interventions analysed in this SR have all been the focus of other SRs, although few focused exclusively on FCAS and none had foci on both FCAS and gender specific and transformative interventions. The frequencies of interventions studied in the literature included in this review is consistent with existing reviews: SRs on interventions including cash transfers, TVET, asset transfer, and capacity building are more common, while there is less SR evidence on interventions such as AWPSs or community‐based services (except on health‐related outcomes). Our findings on the effectiveness of interventions on primary outcomes is consistent with other reviews: we observe similarly nuanced results in regard to IPV related outcomes to Spangaro's review (2013) who finds only a minority of studies successfully addressing the incidence of violence, or in the J‐PAL's (2021) synthesis of randomised evaluation on managing and preventing crime, violence and conflict observing both promising and negative influence of ecomic intervention of IPV occurrence and promising results of the livelihood and training based interventions. However, none of those reviews are exclusively focusing on FCAS. Compared to Langer et al. (2018) and De Koker et al. (2014), we often observe a similar magnitude of small to medium positive effects on empowerment related outcomes although we observe that many of the SRs on similar interventions and outcomes did not report effect sizes but only direction of effect. Conversely, we find the magnitude of effects relatively higher in comparison to the recent 3ie SR on social cohesion (Sonnenfeld et al., 2021), which assessed some similar interventions, but where the largest significant summary effect was 0.10 (SMD), while we find effects as large as 0.87 (SMD).

For AWPS, we found only two other reviews. While neither conducted a meta‐analysis, both reviews found no evidence in favour of AWPS, with Arango and colleagues (2014), specifying that there is a lack of sufficient evidence on AWPS in LMICs. This is similar to our review regarding the literature gap that does not allow us to state the effectiveness of this type of intervention.

Two gender‐specific or transformative reviews included asset transfers within their analysis, but neither separated asset transfers from other interventions such as cash transfers. In their analysis of combined interventions, Eggers del Campo and Steinert (2020) found an overall reduction in physical, sexual and emotional intimate partner violence that we did not observe in either of these intervention groups in our review.

Eight reviews included cash transfers as an intervention, but many of the reviews bundled cash transfers with asset transfers such as livestock. The results presented for cash transfers were mixed. Arango et al. (2014) found that there was not enough evidence in LMICs to come to a definitive conclusion, whereas McQueston and colleagues (2013) found nuanced evidence on the effectiveness of cash transfers on early marriage and childbearing. Also similar to our findings, Manley and colleagues (2013) found conditional and unconditional cash transfers had similar effects and Baird and colleagues (2013) found that conditional and unconditional cash transfers had similar effects for both boys and girls.

Only one review discussed community dialogues and reconciliations. In this case, this type of intervention was grouped with others, such as sensitisation campaigns. The interventions as a whole were found to have significant and positive effects on sexual intimate partner violence (Eggers del Campo & Steinert, 2020). While our included studies did not provide a measure of IPV, this is in line with our observations on improved attitudes and increased support for women's economic, social and human rights by men, household and family members and community members. However, we see that only a few studies included in the other review are gender‐ specific or transformative.

Community‐based services were examined in four reviews, which mainly focused on health outcomes. Both studies, which presented health outcomes, found community‐based services to have a significant and positive effect (Lassi et al., 2015), and those focused on women's empowerment outcomes found the same results (Arango et al., 2014; Eggers del Campo & Steinert, 2020). Eggers del Campo and Steinert, (2020) grouped community‐based services with asset transfers. This observation aligns with our findings and the low number of studies included under this category due to the exclusion of health from our scope.

Two reviews found evidence in favour of the effects of discussion groups, yet neither had a focus on LMICs. One review included one discussion group‐based intervention in an LMICs and found a reduction in violence perpetrated by men (De Koker et al., 2014). This is in line with our findings on improved attitudes and increased support for women's economic, social and human rights by men, household and family members and community members.

Five reviews looked at the institutional provision of loans and savings (with Vaessen and colleagues [2014] conducting a meta‐analysis) but found no consistent evidence for an effect of microcredit on women's control over household finances. This is in line with our findings that institutional provision of loans and credit improve both the capacities of women to use finance‐related services and their access to credit, but do not increase their participation in decision‐making at the household and community level.

Seven reviews assessed interventions focused on life, social and livelihood skills and capacity building (LSCB). One review, which utilised the GRADE methodology, found that life, social and livelihood skills and capacity building interventions led to an increase in women's formal wage employment, income and economic empowerment (Langer et al., 2018). Dickson and colleagues (2012) also found modest but significant effects in terms of the income generating ability of women and girls after being supported through these interventions. This is in line with our observations that LSCB led to increased capacities in the use of finance services.

Four reviews analysed safe spaces interventions with the main takeaway being that there is a lack of rigorous evidence around this intervention type in LMICs.

Two reviews looked at self‐help groups (SHGs) and Village Savings and Loan Associations (VSLAs). Brody and colleagues (2016) found an effect size through a meta‐analysis, with SMDs ranging from 0.06 to 0.41 in favour of self‐help group participant's economic and political empowerment. We found that SHGs and VSLAs have, among our list of interventions, a positive impact across the three dimensions of women's empowerment.

Six reviews looked at sensitisation campaigns and found very mixed results. LaCroix and colleagues (2014) found positive and significant effects of HIV sensitisation campaigns on women's HIV knowledge and condom use. Other studies, such as Arongo and colleagues (2014) found much more mixed results with a lack of sufficient evidence being a major issue. We found the same mixed results in our review with a positive and significant effect on improved awareness and responsiveness to the demands, claims, rights and inputs of women but no significant effects on other sets of outcomes.

Finally, four reviews analysed technical and vocational education and training (TVET) interventions. Langer and colleagues (2018) found the interventions led to an increase in women's income and empowerment. However, our effect sizes from FCAS are considerably lower on income, employment and empowerment compared to similar outcomes in LMICs in general in Chinen's (2017) review. Eggers del Campo & Steinert (2020) also found a positive effect of the interventions on women's experience of physical and emotional intimate partner violence. Our review shows that TVET interventions mainly affect the resources‐related outcomes of women's empowerment.

### 6.5 Discussion and conclusions

We have conducted a large and comprehensive SR on women's empowerment and gender equality in FCAS. We reviewed 104 studies evaluating the effects of interventions across 44 different outcomes. Our results suggest that most interventions have an overall positive effect on beneficiaries as compared to those not receiving these interventions. As expected, depending on which outcome we look at, different interventions produce the largest effects. Below we discuss some patterns, crosscutting observations and discussion points from our sample of included studies.

Our review finds multiple examples of interventions affecting change on primary outcomes that are closely related to the mechanism of the intervention. For example, asset transfers, cash transfers and the institutional provision of loans and savings mainly have positive and significant positive effects on economic and livelihood related outcomes. Similarly, inclusive community‐driven development and community dialogues mainly have positive and significant effects on the improvement of systems. Among our 14 interventions, SHGs and VSLAs, TVET and cash transfers are the only interventions having effects across all three dimensions of women's empowerment and gender equality.

Although theory dictates that the three dimensions of empowerment are inextricably linked, we observe that the effects of most interventions are generally limited to only one dimension. In line with this, we find a relative lack of multi‐component interventions that specifically target the three dimensions at the same time. Although the data of our 14 interventions does not allow us to posit on the effect of multi‐component interventions, the importance of multidimensional empowerment in the theoretical literature suggests that more research on multicomponent interventions would be of interest.

As presented in Table 59, our panel of interventions have beneficial effects on different outcomes related to their categories and dimensions within the empowerment framework. These findings can be aggregated at the UNSCR 1325 Pillar level:

- Interventions classified in the participation pillar yield positive and significant effects across outcomes in all three dimensions of the Resources‐Agency‐Achievement framework. That said, only three types of interventions exhibit a positive effect across the empowerment dimensions (i.e., cash transfer, SHGs and VSLAs and TVET) and a majority of the interventions under this pillar focus on resources and agency.
- Our sample of interventions under the prevention and protection pillars are not large enough to make general conclusions. However, there seems to be a stronger focus on the achievement dimension of the empowerment framework.
- Multi‐pillar interventions (i.e., discussion groups, life, social, and livelihood skills and capacity building and safe spaces) yield positive and significant effects on outcomes across a number of categories: economic and livelihood related resources, individual agency, community level agency, norms and behaviour change.
- Included interventions often yield positive and significant results on their targeted, primary outcomes, but are less effective at impacting change on more complex downstream outcomes like intimate partner violence (see Table 58). Table 59 also illustrates the absence of an intervention that will singlehandedly address all aspects of gender equality and women's empowerment (Table 54).

**Table 54:** Overview of interventions effectiveness

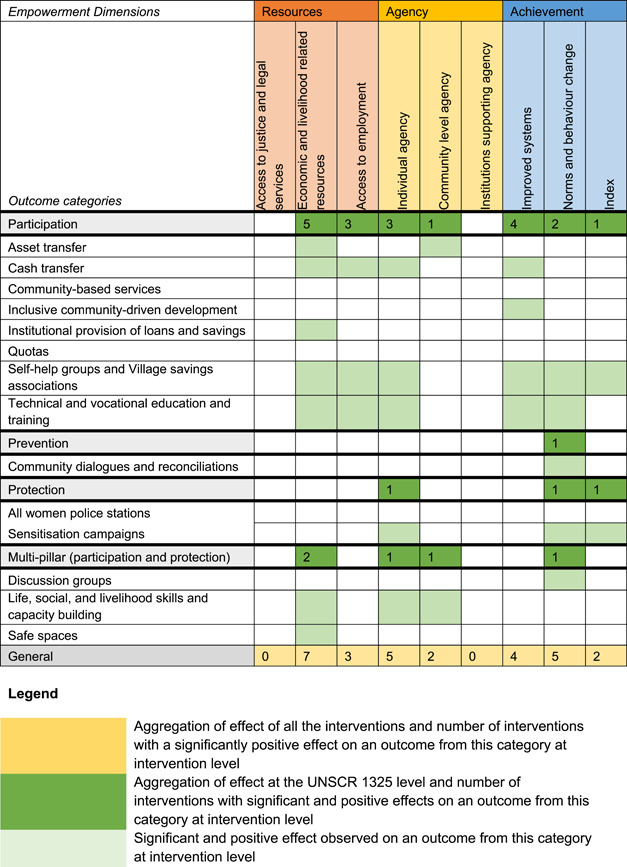

#### 6.5.1 Implications for decision‐making and practice

*To achieve transformational effects on women's empowerment outcomes, interventions need to be carefully designed to address key barriers and dimensions of women's empowerment*

- There is a need to create and implement explicit programme components that target empowerment outcomes, if such empowerment outcomes are desired.
- For interventions to have effects on multiple dimensions of empowerment or on outcomes that are difficult to shift, they need to be designed to address the different causal pathways of each intervention type to better target the outcomes where they have a significant effect.
- Programmes that yield impacts beyond the immediate, primary outcomes that they targeted reflect a clear understanding of the barriers to empowerment within their respective contexts and tailor the design of components accordingly, including through critical gender analysis.
- Applying a gendered implementation plan may contribute to programme effectiveness. Implementation plans require careful application of a gender lens to assess whether implementation choices are gender‐sensitive, from the choice of venue, to the outreach campaign, and personnel, to name a few.
- The potential of women peer‐to‐peer exchanges and collective‐building programme components such as mentoring and women's groups are important facilitators to be considered. These types of programme components are consistently linked to the creation of social capital and networks among women, which in return provides a step towards empowerment related outcomes.

*Programme design needs to be tailored to the local context and adjusted to work in restrictive contexts*

- Embedding programmes in existing structures contributes to enhanced legitimacy and access to participants, facilitating programme components that target improvements in women's livelihoods and economic situations. Programmes need to balance the risks of co‐option with the benefits of gaining access and the opportunity cost of women not receiving livelihood support activities on a case‐by‐case basis.
- A specific design consideration in these contexts is again the opportunity to apply programme components aimed at women peer‐to‐peer exchanges and collective‐building, which hold potential to create new structures and support systems as a platform for women's empowerment.
- Open dialogue forums may support buy‐in by stakeholders in areas where intrenched partriarchal norms necessitate increased buy‐in by local cultural or religious leaders for appropriate uptake at the community level. Further research is necessary in different contexts and at the community level.

*Targeting of the right beneficiaries is important to maximise impact*

- Appropriate targeting requires understanding of who are the right groups of beneficiaries to include in programmes. Baseline assessments and contextual analysis are key, including assessments of whether male targeting is advisable (e.g., the right target group for addressing norm change may, in some instances, be exclusively male)
- Targeting procedures should be tailored to minimise beneficiary selection errors and maximise trust in administrative processes, programme participation, and the achievement of aimed outcomes.
- Conflict and accusation of favouritism can be addressed proactively if targeting criteria are co‐designed with stakeholders and made transparent and explicit throughout.
- The most vulnerable households and women should be the main beneficiary targets, as they are not only able to realise the most gains related to meeting their basic needs, but also benefit most from access to credit and capital to engage in economic activities. The targeting process needs to be inclusive geographically, ensuring that vulnerable groups in remote locations are selected for these programmes.

- Age is another factor that needs to be considered for targeting. For instance, safe space programmes should target girls before they reach adolescence where they are more at risk of social pressures that may drive them into forced marriages and unwanted pregnancy.

- *Prioritisation of support to women in peace processes and recovery and relief context*
  Despite the broad reach of our review, many studies that exclusively focused on outcomes related to peacebuilding in fragile contexts did not have a gender component inclusive to our scope. Interventions focusing on peacebuilding need to be tailored to integrate a gender specific or transformative lens.
- *Prioritisation of gender‐transformative and gender‐specific interventions*
  Interventions and monitoring of results should go further than disaggregating data by gender to specifically targeting gender components and outcomes. This includes tailoring interventions with an equity focus, such as aligning cash disbursement schedules with women's time use.
- *Prioritisation of measuring peace and gender‐related outcomes*

The lack of evidence in this review on the recovery and relief pillar represents a need for improved resilience evaluations that are simultaneously gender‐sensitive or transformative.

There are a number of intervention specific implications for policy and practice and these are provided in the tables below (Tables 55, 56, 57, 58).

**Table 55:** Participation interventions implications

Intervention	Intervention works on	Intervention does not work on
Asset transfers	*Resources*	*Resources*
	Women have increased access to and ownership of assets, credit and income.	Women can access decent work (formal and informal employment)
	*Agency*	*Agency*
	Increased participation in decision making by women at the household or community level	Effect unknown–insufficient evidence
	*Achievement*	*Achievement*
	Effect unknown–insufficient evidence	Women have improved attitudes, self‐image and confidence Reduced frequency and distribution of types of violence by an intimate partner Improved quality of relationships between women and their household and community members Women's Empowerment/Equality Index
	*Intervention design suggestions to maximise benefits*	
	Identify and secure multiple convenient locations for training activities (e.g., agricultural education workshops) and for asset collection to improve participation and productive use of assets Design quality (participatory and ‘hands‐on’), comprehensive and inclusive training for recipients to promote understanding and successful adoption of relevant skills to carry out activities related to asset transfer such as agricultural production Conduct quality checks for asset transfers and provide necessary supporting inputs (e.g., fences and irrigation equipment) Ensure that adequate household level social and labour support systems for women beneficiaries are available. For instance, through sensitisation or training of family members, particularly husbands Explore multicomponent interventions that include legal support, microcredit, cash transfers, and capacity building more holistically to support the productive and sustained use of assets	
	*Contextual considerations to keep in mind*	
	Traditions and norms, such as gendered divisions of labour and women's rights to land hinder the type of activities women can participate in and eventual empowerment outcomes Programmes have attempted to address this through gender sensitisation campaigns targeted at partners Programme activities (including compulsory training) may disrupt usual women's day‐to‐day work obligations and affect time use preferences	
	*Good practice examples*	
	Burkina Faso's E‐HFP emphasises the need for additional input support to complement asset transfers meant for agricultural purposes. Specifically, whilst there was an indication of improved ability for women to use land for agricultural purposes, primary obstacles included insufficient water access, pest and animal attacks. Hence, the provision of additional inputs in the form of irrigation equipment, fences and pest control chemicals could have mitigated these challenges and enhanced the benefits realised from the assets transferred Bangladesh's TUP illustrates the importance of ensuring adequate social and labour support systems for women beneficiaries. The livestock and poultry that women received raised women's workloads at home and in some cases prevented women from being employed outside their homes. As an alleviating factor, the women benefited from children and husband assistance with selected tasks related to the care of their livestock. The Bangladesh's IGVGD asset transfer programme included a microfinance facility which enhanced economic involvement as it allowed women to use the borrowed money for various purposes including paying for rental on land they used to start and carry out agricultural activities related to the agricultural assets they had received	
**Intervention**	**Intervention works on**	**Intervention does not work on**
Cash transfers	*Resources*	*Resources*
	Women have increased access to and ownership of assets, credit and income Women and girls have equitable access to livelihood support services More women engaged in other micro, small, and medium‐sized enterprises	Women can access decent work (formal and informal employment)
		*Agency*
		Women have more and better control over their bodies and sexual health Women have increased freedom of movement and association Women are equipped with better life skills that allow them to be prepared for crisis or shocks and recover from them Reduced support for or occurrence of child and forced marriage Increased participation in decision making by women at the household or community level Women participate more in their community
	*Agency*	
	Reduced percentage of women agreeing with certain reasons that justify violence against women and girls	
		*Achievement*
		Women have improved attitudes, self‐image and confidence Improved attitudes and increased support for women's economic, social and human rights by men, household and family members and community members Safer and more secure household, communities and areas/territories for women, girls, men and boys Reduced frequency and distribution of types of violence by an intimate partner Improved quality of relationships between women and their household and community members Women's Empowerment/Equality Index
	*Achievement*	
	Increased representation of women in local and subnational civil and political processes, including during peacebuilding and post‐conflict restoration	
	*Intervention design suggestions to maximise benefits*	
	Incorporating couples' relationships, social and livelihood skills, mentoring and capacity building components can support household relationships and conflict resolution skills Targeting the most vulnerable households and women who struggle to meet their basic needs and those who do not have access to credit or capital to start businesses Targeting family units (compared to individuals) and carrying out clear awareness‐raising campaigns supports participation When determining transfer size, considerations should be made for inflation and household size, but relatively high cash transfers may discourage participation in wage labour activities. Mentorship, training and support from family members promotes adherence and compliance to programme requirements, especially in conditional cash transfer programmes. We find no difference in the effects of conditional transfers compared to unconditional transfers. This should be carefully considered given the higher administrative costs of conditional transfers.	
	*Contextual considerations to keep in mind*	
	Social norms, existing gender roles and rigid restrictive beliefs can preserve men's dominance (regardless of women's income contribution) in household decision making, including participation in these programmes.	
	*Good practice example*	
	After noticing slow uptake and lack of awareness in communities, the implementation team from the ZCTP in Malawi adopted a sensitisation campaign to help prospective participants understand the objectives of the intervention, which improved participation rates In the Takaful and Karama Programme in Egypt, severely poor people used a large part of the cash transfer to take care of their basic needs including food, clothing and medical expenses following the receipt of the cash transfer. Notably, a significant number of the beneficiaries were also highly in debt. Hence, the cash transfer alleviated short term needs for vulnerable beneficiaries to focus on more sustainable, long‐term economic goals The mentorship and coaching components in Kenya's Rural Entrepreneur Access Project (REAP) implemented by the BOMA Project helped ensure that participants adhered to the programme, enhanced school attendance, financial literacy and beneficiaries' knowledge	
**Intervention**	**Intervention works on**	**Intervention does not work on**
Community‐based services	*Resources*	*Resources*
	Effect unknown–insufficient evidence	Effect unknown–insufficient evidence
	*Agency*	*Agency*
	Effect unknown–insufficient evidence	Effect unknown–insufficient evidence
	*Achievement*	*Achievement*
	Effect unknown–insufficient evidence	Effect unknown–insufficient evidence
	*Intervention design suggestions to maximise benefits*	
	Not enough studies for qualitative synthesis	
	*Contextual considerations to keep in mind*	
	Not enough studies for qualitative synthesis	
	*Good practice example*	
	Not enough studies for qualitative synthesis	
**Intervention**	**Intervention works on**	**Intervention does not work on**
Inclusive community‐driven development	*Resources*	***Resources***
	Effect unknown–insufficient evidence	Effect unknown–insufficient evidence
	*Agency*	*Agency*
	Effect unknown–insufficient evidence	Women have increased freedom of movement and association Women have more positive attitude towards taking action to claim their rights Reduced percentage of women agreeing with certain reasons that justify violence against women and girls
	*Achievement*	
	Increased representation of women in local and subnational civil and political processes, including during peacebuilding and post‐conflict restoration	
		*Achievement*
		Increased attention and focus on the needs and priorities of women and girls, and other vulnerable groups during relief and recovery in conflict and post‐conflict settings Increased community support for women's and children's human, economic and legal rights Women have improved attitudes, self‐image and confidence Safer and more secure household, communities and areas/territories for women, girls, men and boys
	*Intervention design suggestions to maximise benefits*	
	Composition of committees and representatives in decision‐makers structures mattered largely for the legitimacies of these bodies within the communities targeted by the intervention (e.g., mandated parity) Include capacity building initiatives for established governance bodies Encourage collaborations between institutions to mitigate potential conflicts between multiple governance systems or bodies through regular meetings and combined working groups and balance politics and power relations between existing and new bodies	
	*Contextual considerations to keep in mind*	
	Governance structures introduced may coexist with traditional governance authorities (e.g., local chiefs) as well as local government structures. Navigating this coexistence requires a careful balancing of politics and power relations, which limited the space for project implementation and reach in some instances Powerful elites with a vested interest may undermine project implementation when the legitimacy and capacity of governance bodies is a challenge	
	*Good practice example*	
	The Democratic Republic of the Congo's Tuungane programme featured mandated gender parity in the composition of project committees, which tended to facilitate more women to participate in the programme In Afghanistan's NSP, collaboration between entities or institutions such as cultural or religious leaders, public and private agencies was crucial. Bottom‐up initiatives inclusive of diverse groups of community members helped legitimise the intervention's committees as local institutions Additionally, in Afghanistan, project success hinged on timely disbursement of funds, regular meetings and follow‐up on community initiatives. The timing of project implementation centred around it not coinciding with annual harvesting seasons, and the time use of community members, particularly women	
**Intervention**	**Intervention works on**	**Intervention does not work on**
Institutional provision of loans and savings	*Resources*	*Resources*
	Women have increased access to and ownership of assets, credit and income	Improved capacity of women entrepreneurs
	*Agency*	*Agency*
	Effect unknown–insufficient evidence	Increased participation in decision making by women at the household or community level
	*Achievement*	*Achievement*
	Empowerment index	Women's Empowerment/Equality Index
	*Intervention design suggestions to maximise benefits*	
	Not enough studies for qualitative synthesis	
	*Contextual considerations to keep in mind*	
	Not enough studies for qualitative synthesis	
	*Good practice example*	
	Not enough studies for qualitative synthesis	
**Intervention**	**Intervention works on**	**Intervention does not work on**
Quotas	*Resources*	*Resources*
	Effect unknown–insufficient evidence	Effect unknown–insufficient evidence
	*Agency*	*Agency*
	Effect unknown–insufficient evidence	Women have more positive attitude towards taking action to claim their rights
	*Achievement*	*Achievement*
	Effect unknown–insufficient evidence	Effect unknown–insufficient evidence
	*Intervention design suggestions to maximise benefits*	
	Not enough studies for qualitative synthesis	
	*Contextual considerations to keep in mind*	
	Not enough studies for qualitative synthesis	
	*Good practice example*	
	Not enough studies for qualitative synthesis	
**Intervention**	**Intervention works on**	**Intervention does not work on**
Self‐help groups and village savings and loans associations	*Resources*	*Resources*
	Increased capacity of women to understand and use financial, banking, and business services effectively Women have increased access to and ownership of assets, credit and income Women can access decent work (formal and informal employment)	Women have increased access to and ownership to assets, credit and income
		*Agency*
		Reduced percentage of women agreeing with certain reasons that justify violence against women and girls Increased participation in decision making by women at the household or community level Women participate more in their community
	*Agency*	
	Women are more aware of their rights and the roles and responsibilities of duty bearers Women have more positive attitude towards taking action to claim their rights Women are equipped with better life skills that allow them to be prepared for crisis or shocks and recover from them	
		*Achievement*
		Communities have a more positive attitude towards women/marginalised groups Women have improved attitudes, self‐image and confidence Improved quality of relationships between women and their household and community members
	*Achievement*	
	Effective prevention strategies supported to end violence against women and girls Decreased violence/discrimination at the household level Women's Empowerment/Equality Index	
	*Intervention design suggestions to maximise benefits*	
	Include training components (e.g., financial literacy and business skills) for participants and group leaders in self‐help groups and Village Savings and Loan Associations programmes to enhance confidence in leadership by participants To enhance participation, encourage existing groups to use traditional knowledge sharing platforms such as listening in meetings by prospective participants and motivational workshops In programme design, ensure that it is mandatory for groups to establish clear rules of participation and communication by groups to promote adherence and willingness to participate	
	*Contextual considerations to keep in mind*	
	Group compliance obligations and domination by elites may foster intergroup conflict, whereas restrictive legislative frameworks may stifle programme expansion, which could hinder programme impact Women may not receive support from their partners due to pervasive gender norms and roles Regularity of meetings ensures reliable participation and group success Meeting spaces should be in centralised locations to reduce transport burden on participants	
	*Good practice example*	
	During the Saving for Change (SfC) programme in Mali, some participants accepted the programme because replicating agents were considered legitimate and this legitimacy was derived from a formal training they underwent and the certificate they received. Trainers gained status and recognition by both clients and the broader community owing to this training In the same programme, group support was also seen to promote participation by trust building in the system of self‐help groups and Village Savings and Loan Associations first. For instance, there were groups that refused to join the association due to lock‐in costs. To garner interest and encourage participation, an agent brought women from different groups to visit and observe association meetings and report back to their respective groups	
**Intervention**	**Intervention works on**	**Intervention does not work on**
Technical and vocational education and training	*Resources*	*Resources*
	Women have increased access to and ownership of assets, credit and income Improved capacity of women entrepreneurs	Women can access decent work (formal and informal employment)
		*Agency*
	*Agency*	Women have more and better control over their bodies and sexual health Women have increased freedom of movement and association Reduced percentage of women agreeing with certain reasons that justify violence against women and girls Women are equipped with better life skills that allow them to be prepared for crisis or shocks and recover from them Increased participation in decision making by women at the household or community level
	Reduced support for or occurrence of child and forced marriage	
	*Achievement*	
	Increased representation of women in local and subnational civil and political processes, including during peacebuilding and post conflict restoration Women have improved attitudes, self‐image and confidence	
		*Achievement*
		Increased community support for women's and children's human, economic and legal rights Reduced frequency and distribution of types of violence by an intimate partner Improved quality of relationships between women and their household and community members Women's Empowerment/Equality Index
	*Intervention design suggestions to maximise benefits*	
	Design and implement a tailored and sensitive awareness‐raising and outreach campaign to attract participants into the programme Identify and select a central and safe location that is easily accessible (e.g., via public transport, within walking distance of participants) Provide allowances for transport or childcare to encourage participation and reduce barriers of access Target single sex participants (women) to allow space for open engagement and enhance feelings of safety An integration with life, social, and livelihood skills and capacity building (LSCB) capacity building programmes are promising as assessments of stand‐alone technical and vocational education and training (TVET) programmes and TVET components within LSCB programmes emphasise the interconnectedness of both types of skill sets A multicomponent or multi‐faceted design approach to support beyond the skill building component including mentoring, peer‐to‐peer exchange and ‘collective‐building’ formats such as women's self‐help groups and clubs	
	*Contextual considerations to keep in mind*	
	Training curricula and materials require careful adaptation to local context and translation into local languages The skilfulness and approach of programme trainers is a key implementation element and driver of programme satisfaction Tailoring programming to context is crucial, particularly in contexts where women or girls are barred from certain occupations	
	*Good practice example*	
	The Gender and Entrepreneurship Together (GET) Ahead for Women in Enterprise programme in Kenya was designed specifically with a gender lens throughout and aimed to address both the practical and strategic needs of low‐income women in enterprise by strengthening their basic business and people management skills as well as supporting networking and relationship building. Women beneficiaries, in addition to the freedom to express themselves without the censure of men, valued the ‘camaraderie’, support and mentorship they received from one another. Mentoring was seen to enhance business skill transfer and development by contextualising skills in the experience of an established entrepreneur The Empowerment and Livelihood for Adolescents (ELA) programme in Sierra Leone referred to its multifaceted approach to simultaneously tackle multiple disadvantages young women face, related to having agency over their bodies and barriers to accumulating human capital. This approach combines the provision of life skills, vocational skills and microfinance, delivered within a social club and safe space for women Liberia's Economic Empowerment of Adolescent Girls and Young Women (EPAG) programme deliberately designed a pairing, small group (3–4 girls) approach between girls with the aim of improving girls' skill development as well as to promote the creation of social capital, such as friendships, mutual trust and support networks among the girls. The girl participants appreciated this design element and reported that they helped each other during and outside of the classroom activities	

**Table 56:** Prevention interventions implications

Intervention	Intervention works on	Intervention does not work on
Community dialogues and reconciliations	*Resources*	*Resources*
	Effect unknown–insufficient evidence	Effect unknown–insufficient evidence
	*Agency*	*Agency*
	Effect unknown–insufficient evidence	Effect unknown–insufficient evidence
	*Achievement*	*Achievement*
	Improved attitudes and increased support for women's economic, social and human rights by men, household and family members and community members	Effect unknown–insufficient evidence
	*Intervention design suggestions to maximise benefits*	
	Not enough studies for qualitative synthesis	
	*Contextual considerations to keep in mind*	
	Not enough studies for qualitative synthesis	
	*Good practice example*	
	Not enough studies for qualitative synthesis	

**Table 57:** Protection interventions implications

Intervention	Intervention works on	Intervention does not work on
All‐women police stations	*Resources*	*Resources*
	• Effect unknown–insufficient evidence	• Effect unknown–insufficient evidence
	*Agency*	*Agency*
	• Effect unknown–insufficient evidence	• Effect unknown–insufficient evidence
	*Achievement*	*Achievement*
	• Effect unknown–insufficient evidence	• Effect unknown–insufficient evidence
	*Intervention design suggestions to maximise benefits*	
	• Not enough studies for qualitative synthesis	
	*Contextual considerations to keep in mind*	
	• Not enough studies for qualitative synthesis	
	*Good practice example*	
	• Not enough studies for qualitative synthesis	
**Intervention**	**Intervention works on**	**Intervention does not work on**
Sensitisation campaigns	*Resources*	*Resources*
	• Effect unknown–insufficient evidence	• Effect unknown–insufficient evidence
	*Agency*	*Agency*
	• Women have more positive attitude towards taking action to claim their rights	• Reduced percentage of women agreeing with certain reasons that justify violence against women and girls
	*Achievement*	*Achievement*
	• Increased awareness in communities of the issues affecting women • Women's Empowerment/Equality Index	• Effective prevention strategies supported to end violence against women and girls • Women have improved attitudes, self‐image and confidence • Improved attitudes and increased support for women's economic, social and human rights by men, household and family members and community members • Reduced frequency of physical intimate partner violence (IPV) • Reduced frequency of sexual IPV • Improved quality of relationships between women and their household and community members
	*Intervention design suggestions to maximise benefits*	
	• Design more frequent programme activities such as training and counselling over an extended period of time • Adopt in‐person platforms and intimate sessions that largely focus on couples, and men as perpetrators • Engage and utilise respected opinion leaders (e.g., religious leaders) to counsel, mentor and sensitise couples to IPV with focus on the tenets of healthy relationships and conflict resolution skills and knowledge • Tailor the types of community engagement to context, such as parent's evenings, dialogue groups and exchanges • Draw on religious messages to support change regarding gender equality • As seen in other intervention groups, sensitisation campaigns can be a key ‘add‐on’ to enhance the uptake and implementation of an intervention. As a result, sensitisation may be an ‘easy win’ to increase the opportunity of trickle‐down effects across programmes	
	*Contextual considerations to keep in mind*	
	• Gender norms, roles and culturally developed patriarchal attitudes are often the most entrenched and difficult barrier in successful sensitisation but engaging men, particularly local opinion leaders, may counter traditional gender roles • Issues of personal image, knowledge of rights and empowerment can be addressed gradually as they depend on the nature of close relationships and personal confidence from successful programme impact • Using communal and public platforms can work to reach more participants at the same time compared to intimate settings	
	*Good practice example*	
	• Training men to understand the value of women's roles better was an important exercise for Men's Action for Stopping Violence Against Women (MASVAW) participants in India. Particularly, activities that allowed men to imagine ‘the average day in a woman's life’ were instrumental in provoking reflection around gender roles and norms. Opinion leaders incorporated in India' s MASVAW programme expressed excitement to engage actively with other participants and supported programme staff in identifying and embedding knowledge of gender inequalities in their communities. Their involvement encouraged them to prioritise intimate partner violence prevention and wider engagement on gender issues with the community • In Rwanda, the Indashyikirwa programme's personalised settings were more conducive to engagement and listening through participatory methods of community engagement. These included community forums, to encourage openness, particularly regarding violence against women and gendered issues. Participatory training and community‐based activism in the Indashyikirwa programme encouraged a culture of community engagement to activate learning and third‐party intervention. In this way, participants were able to protect those that suffered from intimate partner violence.• A religious opinion leader in Rwanda Indashyikirwa's programme noted the importance of drawing on religious messages and principles to support change regarding gender equality as it allowed participants to understand and internalise that the genders were all equal	

**Table 58:** Protection interventions implications

Intervention	Intervention works on	Intervention does not work on
Discussion groups	*Resources*	*Resources*
	Effect unknown–insufficient evidence	Women have increased access to and ownership to assets, credit and income
	*Agency*	*Agency*
	Effect unknown–insufficient evidence	Effect unknown–insufficient evidence
	*Achievement*	*Achievement*
	Improved attitudes and increased support for women's economic, social and human rights by men, household and family members and community members	Effect unknown–insufficient evidence
	*Intervention design suggestions to maximise benefits*	
	Not enough studies for qualitative synthesis	
	*Contextual considerations to keep in mind*	
	Not enough studies for qualitative synthesis	
	*Good practice example*	
	Not enough studies for qualitative synthesis	
**Intervention**	**Intervention works on**	**Intervention does not work on**
Life, social, and livelihood skills and capacity building	*Resources*	*Resources*
	Increased capacity of women to understand and use financial, banking, and business services effectively	Women have increased access to and ownership to assets, credit and income
	*Agency*	*Agency*
	Women have increased freedom of movement and association Women are more aware of their rights and the roles and responsibilities of duty bearers Reduced percentage of women agreeing with certain reasons that justify violence against women and girls Women are equipped with better life skills that allow them to be prepared for crisis or shocks and recover from them Increased participation in decision making by women at the household or community level	Reduced support for or occurrence of child and forced marriage Women participate more in their community
		Achievement
		Communities have a more positive attitude towards women/marginalised groups Women have improved attitudes, self‐image and confidence Improved attitudes and increased support for women's economic, social and human rights by men, household and family members and community members Decreased violence/discrimination at the household level Safer and more secure household, communities and areas/territories for women, girls, men and boys Reduced frequency of physical IPV Reduced frequency of sexual IPV Improved quality of relationships between women and their household and community members Women's Empowerment/Equality Index
	*Achievement*	
	No significant effect identified in the list of studies	
	*Intervention design suggestions to maximise benefits*	
	Design and implementation of a sensitive and targeted awareness‐raising and outreach campaign is key to attract participants into the programmes Provide participants with a central and safe location that is easily accessible and/or provides childcare facilities promotes acceptability and programme participation Life, social and livelihood skills and capacity building design benefits from the inclusion of and coupling with technical and vocational education and training (TVET) programmes due to the interconnectedness of both types of skill sets, expressed by a demand and need for both types of skills to enhance participants life capabilities and opportunities Incorporate elements that address contexts and norms issues directly such as women's peer‐to‐peer exchange including mentoring elements, women's self‐help groups and clubs Trainer skills and approaches are an important consideration and factor for participant satisfaction Continuous sourcing of participant feedback and consultation to allow rapid programme iteration (e.g., programme locations)	
	*Contextual considerations to keep in mind*	
	Social norms and restrictive contexts determine the space in which programmes can operate and emphasise the need to strike balance between the depth of embedding programmes in these contexts to operate and reach women and the adjustment of programme empowerment outcomes to avoid strong conflict with existing norms and practices Failure to adapt the curriculum to each context may affect uptake and relevance Lack of men's engagement may be a key barrier to empowerment outcomes that may impede skill development to translate into social change	
	*Good practice example*	
	COMPASS programme faced opposition from parents and community leaders in Congo, Ethiopia and Pakistan with participants and stakeholders questioning the appropriateness of the programme activities. Only through extensive sensitive, targeted awareness‐raising and outreach campaigns that included extensive discussions, house‐to‐house visits and negotiations with community leaders, local authorities, and parents, was consensus achieved and implementation proceeded. Through trust and legitimacy, the programme was able to collaborate with leaders of ‘secret' societies associated with female genital mutilation enhancing its reach to participants it would have not been able to access In Afghanistan's participants What Works to Prevent Violence Programme participants had access to TVET‐linked programme components such as cash crop farming, animal husbandry, and embroidery within a wider empowerment programme and women peers for mentoring including fellow programme participants, trainers and formal mentors provided by the programmes. Access to start‐up capital and learning from women in similar contexts based on shared experiences and realities was reported as a key benefit of the arrangement. Women formed social safety nets for each other by staying in touch post the intervention and supporting each other's business ventures. These arrangements resulted in enhanced social capital, a crucial factor in targeting structural barriers to empowerment	
**Intervention**	**Intervention works on**	**Intervention does not work on**
Safe spaces	*Resources*	*Resources*
	Women have increased access to and ownership of assets, credit and income	Increased capacity of women to understand and use financial, banking, and business services effectively
	*Agency*	*Agency*
	Effect unknown–insufficient evidence	Women have more and better control over their bodies and sexual health Women have increased freedom of movement and association Women are equipped with better life skills that allow them to be prepared for crisis or shocks and recover from them Reduced support for or occurrence of child and forced marriage Increased participation in decision making by women at the household or community level
	*Achievement*	
	Effect unknown–insufficient evidence	
		*Achievement*
		Women have improved attitudes, self‐image and confidence Improved quality of relationships between women and their household and community members
	*Intervention design suggestions to maximise benefits*	
	Target adolescent girls before they are culturally considered ready to marry to combat trends of forced marriage or teenage pregnancy Promote mentorship to encourage openness, collaboration, interaction and learning issues beyond what is learnt through formal education Secure safe, convenient meeting locations and timing of the meetings Provide incentives and rewards, which can encourage wide participation of the targeted population in programmes, delivered timely and without too stringent conditions Incorporate appropriate awareness and communication strategies within households, especially when recruiting adolescent girls to tackle negative perceptions about programme intentions and debates around children's rights	
	*Contextual considerations to keep in mind*	
	Changes in long‐standing social and cultural norms and attitudes towards women or girls may need time to reflect Mentors' motivation level and capacity development in advocacy, outreach, communication and networking skills are key to winning the confidence of parents to encourage girls to participate Baseline analyses and participatory research before implementation support appropriate and effective safe spaces	
	*Good practice example*	
	The Ishraq programme in Egypt targeted girls 13–15 years of age, which was a factor that may contribute to programme impacts where girls 15 and older were considered ready for marriage. Awareness building among participants as well as their household members and the wider community at an early stage of the intervention played a critical role as many of such outcomes were compounded by social, cultural norms and traditions The Adolescent Girls Empowerment Programme in Zambia highlighted the importance and centrality of mentorship as it provided a platform for girls to open up to matters that they may not be able to share with their families and this built up their self‐esteem	

#### 6.5.2 Implications for research

- More research is needed regarding the effects of gender specific or transformative interventions on peace. Future syntheses and evaluations should address the need for being gender transformative in peacebuilding endeavours and evaluating their effectiveness on peace at the local and subnational levels.
- More rigorous evidence is needed regarding the effects of gender specific or transformative interventions in the Middle East, North Africa and Central and Latin America.
- Lesbian, Gay, Bisexual, Transgender, Queer and/or Questioning, Intersex, and Asexual (LGBTQIA+) groups should be specifically through gender‐specific or transformative interventions.
- More research and evidence production is needed on community‐based services, quotas, community dialogues and reconciliation, AWPSs and discussion group interventions.
- Quasi‐experimental studies can make positive contributions to the evidence base, but they need to be carefully designed methodologically to account for confounding and selection bias so that the quality of the studies can be similar to the quality of RCTs and the risks of bias are limited.
- To make meaningful knowledge contributions and to produce reliable evidence, evaluation studies need to follow rigorous methodological designs, and report their research in such a way that allows for transparency and assessment of study quality. Good IEs will register a pre‐analysis plan, establish an appropriate counterfactual (either through random assignment or through an appropriate QED) and collect baseline data from all participants. They will collect outcome data from all participants at intervals that are equivalent across the treatment and control conditions. In reporting their findings authors will account for multiple‐hypothesis testing in their analysis, and report on all outcomes, regardless of significance. When reporting on these evaluations, authors should also report information related to the assignment mechanism, baseline balance among treatment and control groups, attrition and differential attrition, deviations from the intended intervention, potential spill‐over effects, and potential performance bias. Ethical clearances and external validity should also be explicitly discussed.
- There is a need to standardize indicators of gender inequality, and for further research on the development of indices that allow to measure and compare gender‐related contexts at the local and/or community level. This includes the development of gender focused indicators.
- A vast majority of IEs measure the effect immediately after the end of an intervention. Investment in evaluations of the longer‐term impact of interventions will allow for an examination of whether these types of programmes have lasting effects on the lives of women and girls.
- Researchers should consider using existing scales with documented validity and reliability for measuring empowerment. This would allow for easier comparisons across studies and contexts.
- Researchers need to report sufficient quantitative data for effect size calculation in their IEs. This includes providing standard errors alongside regression coefficients, reporting exact p‐values (rather than just an asterisk), and providing standard deviations alongside group means.
- Researchers need to report information on the methods used to generate and analyse qualitative data within IEs reports. Without such information, the, the trustworthiness of the qualitative research becomes questionable and the information from these studies cannot be included in qualitative syntheses.
- A more fluid and widely accepted definition of fragility would result in more inclusion and evidence related to macro‐level experiences of conflict. The methods utilised in this review attempt to triangulate and verify the existing definitions, but as noted in the limitations, there is still a lack of global standards. There is a need for further research on the development of indices that allow for measurement and comparison of fragility‐related contexts at the local or community level.

### 6.6 Limitations, potential biases and deviations from the protocol

The following sections present the main limitations that may have impacted our findings and approach for analysis.

#### 6.6.1 Limitations related to the lack of international gender, fragility and conflict indices at the individual, household and community levels

One of the main challenges in defining our inclusion and exclusion criteria was translating international indices based on national‐level data into criteria for more local or macro‐level analysis. The existing indices, both in regard to gender and to fragility and conflict, were not always constant and may have flaws due to the indicators they use to measure empowerment, gender equality, fragility, conflict and so on. To make the most of sometimes incomplete global indices we adopted a pragmatic approach by combining complementary indices and using the information available in the study to make specific decisions: we have combined the GII, the WPS Index, the World Bank list of FCAS, the OECD list of FCAS and set thresholds with a margin of tolerance if the study context was highly relevant. Although we cover 29 countries worldwide, some countries, including South Africa, and parts of Latin America were not eligible. However, they may have had some interesting insights at the local level in areas where conflict or gender inequality are high. Additionally, countries that would not pass our threshold criteria may have interesting subnational insights, given the fact that many conflicts are either subnational or transnational. Future research and policy might consider setting more international, national and local criteria for the measurement of gender equality, women's empowerment, fragility and conflict as it will facilitate more focused research and more targeted implementation.

#### 6.6.2 Limitations related to the absence of standardised outcome measures for gender equality and women's empowerment in FCAS

Similarly, we observe the relative absence of standardised indicators and outcomes in the gender and FCAS context. Thus, we constructed our set of outcomes by combining existing sets related to gender equality, women's empowerment, peace and security, and the indicators of UNSCR 1325 into a broad set of outcomes that captured each dimension of women's empowerment.

#### 6.6.3 Limitations related to the availability of data for descriptive results and risk of bias

All descriptive results and risk of bias assessments are based on data reported in the included papers. Due to resource and time constraints, we could not contact all authors of the included studies to request additional and/or missing information about their respective study. This may result in some studies being assessed as some risk of bias concerns if they do not report information related to the assessment criteria. The risk of bias assessment may have been different had the information been reported, in which case the assessor could have determined more accurately whether the criteria was met or not met.

#### 6.6.4 Limitations related to the availability of data for qualitative synthesis

We only included studies in the qualitative review component that were linked to included IEs in the quantitative review component. This limits the sample of available evidence in the qualitative component, in particular where interventions are of small scale with little associated programme data publicly available. In addition, our review did not identify linked qualitative studies for all IEs and, what is more, for some intervention categories. This naturally impedes us from being able to conduct an integrated synthesis for these interventions. A final limitation was the lack of reporting of qualitative methods and research processes within IE reports that included qualitative data, which resulted in the exclusion of potentially relevant documents.

#### 6.6.5 Limitations related to the assessment of publication bias

It is not recommended to test for funnel plot asymmetry with fewer than 10 studies (Sterne et al., 2011), thus we were unable to test for publication bias in the majority of our analyses. This limits our ability to interpret heterogeneity, as it is still unclear whether the variation among effects is being driven by publication bias. More rigorous IEs are needed to have a sufficient evidence base to properly assess publication bias.

#### 6.6.6 Limitations related to quantitative study of the existing evidence base

In some instances, the number of included studies for each intervention type and outcome of interest limited our ability to make strong conclusions on the relative effectiveness of the different intervention types. This is notably the case for the following intervention groups: community‐based services, quotas, community dialogues and reconciliation, AWPSs, and discussion groups. In addition, in many instances, we were unable to undertake the full moderator analyses to explore sources of variation for all outcomes due to a limited number of included studies in each intervention type, or a lack of variation among included studies.

Another challenge we faced when conducting the quantitative analysis was the lack of a reported precision estimate in some of the included primary studies. In many cases, authors presented regression coefficients without their associated standard errors, and presented just an asterisk indicating the level of significance, rather than an exact *p*‐value. These poor reporting practices make it difficult to calculate precise effect estimates. In many cases, we had to estimate *t*‐values based solely on the asterisk indicating significance level (as described in our published protocol and our methods section). With a maximum assigned value of 2.8 (or −2.8, depending on the direction of the effect), it is likely that we are underestimating the impact of these interventions, given that most of the effects were positive, and that in reality, *t*‐values can certainly exceed 2.8.

### 6.7 Deviations from the protocol

#### 6.7.1 Adaptation of our list of interventions

Following the extraction process, the team decided to make the list of the interventions more specific to facilitate the use of findings and evidence for policy, practice and research. The main objective of this adaptation was to turn our intervention groups into more granular and specific intervention mechanisms that are directly related to implementation practices.

The team started from the list of included studies and intervention types allocated as per the protocol, which was used as the initial intervention code. The team then looked side by side at each of the studies under each of the original intervention types to identify a specific intervention mechanism. This exercise resulted in a reduction in the number of intervention types from 27 intervention types into 14 specific sets of interventions. This allowed us to avoid working in silos and truly work across the UNSCR pillars.

By linking this new intervention typology to the previous intervention types linked to the UNSCR pillars, the team was able to identify both specific interventions under each pillar and those that are working across pillars; in our case, across the participation and protection pillars (Table 59).

**Table 59:** Adaptation of our list of interventions

New intervention types	Protocol intervention types used for the new intervention types	UNSCR 1325 pillar
All‐women police stations	Gender‐sensitive policing	Protection
Asset transfers	Economic rights and entitlements	Participation
	Economic support asset transfers and livelihoods	
Cash transfers	Cash‐based approaches to support women's access to and participation in education and/or the economy	Participation
Community dialogues and reconciliations	Dialogue groups	Prevention
Community‐based services	Community and leisure activities	Participation
Discussion groups	Behaviour change communication around support for women's rights and preventing sexual and gender‐based violence (SGBV)	Participation
	Civic education and leadership	Protection
Inclusive community‐driven development	Voice and participation in local and subnational governance and development bodies	Participation
Institutional provision of loans and savings	Economic rights and entitlements	Participation
	Financial inclusion	
Life, social and livelihood skills and capacity building	Behaviour change communication around support for women's rights and preventing SGBV	Participation
	Capacity building and technical support to subnational government officials to strengthen service provision for women and gender equality	Protection
	Civic education and leadership	
	Financial inclusion	
	Legal rights education	
	Nonformal education	
	Preventative protection measures	
Quotas	Voice and participation in local and subnational governance and development bodies	Participation
Safe Spaces	Behaviour change communication around support for women's rights and preventing SGBV	Participation
		Protection
Self‐help groups and Village Savings and Loan Associations	Civil society associations and networks	Participation
	Financial inclusion	
Sensitisation campaigns	Behaviour change communication around support for women's rights and preventing SGBV	Protection
	Preventative protection measures	
Technical and vocational education and training	Technical and vocational education and training	Participation

#### 6.7.2 Adaptation related to the use of grading recommendations, assessment, development and evaluations

Grading of recommendations, assessment, development and evaluations (GRADE) traces its origins as a tool to rate the certainty of a body of evidence to use in the epidemiological and health sciences. As such, implementing it in the context of international development IE literature poses a number of challenges and limitations. The tool has been adapted across several domains to better fit the type of available data found in this body of literature and the realities associated with such a large review containing so many analyses. The main limitations that led to adjustments of the tool were:

Two approaches were used in our GRADE analyses that are not reflected in our protocol. First, GRADE recommends that a ‘large’ effect size be defined as a risk ratio of 2. Because this study converted all effect sizes to Cohen's *d*, that was not applicable. Furthermore, consensus on benchmarking using SMD in international development is not clear. We used Cochrane's converter tool to arrive at a D of 0.383 as a ‘large’ effect size. Second, the analyses in this review combined the results of studies that implement both QEDs and RCTs. As such, the ‘Study Design’ column of the Summary of Findings tables show the number of each type of study.

While some studies provided treatment and control group sample sizes, many only provided the total study sample size. GRADE tables in this review were adjusted to reflect that and only report total sample size.

#### 6.7.3 Other deviations

There were several additional deviations to the quantitative approach. First, the team initially expected to use a combination of robust variance estimation (RVE) and traditional meta‐analysis approaches. After extracting the data, we determined to use a unified strategy across all analyses. The traditional meta‐analysis approach using independent effects was our preferred approach, given that our data often had small numbers of studies that would not allow for accurate or reliable RVE estimates. We also initially intended to winsorise outliers and run models with both winsorised and non‐winsorised data. However, given time and resource constraints and the vast number of analyses, we opted for the outlier mechanisms built into the metafor package (Viechtbauer, 2010), including examining studentised residuals and Cook's distances.

Finally, in our protocol we stated that ‘Capitalising on recent shifts towards pre‐registration of studies and their associated pre‐analysis plans, we also examined whether studies that were pre‐registered (e.g., on platforms such as ClinicalTrials. gov, the Open Science Foundation, the American Economic Association's trial registry, or the Registry for International Development Impact Evaluations [RIDIE]) report on all of the outcomes that were proposed in their pre‐analysis plans. This additional analysis of outcome reporting bias may draw on methodologies used in previous work, such as the COMPare Trials Project (Goldacre et al., 2019)’. Due to time and resource constraints, we were unable to complete this process. Instead, we noted if a PAP was reported in the study and used the methods sections of the paper to look for any outcome reporting bias.

## CONTRIBUTIONS OF AUTHORS

Etienne Lwamba (EL), Shannon Shisler (SS), Ada Sonnenfeld (AS), Will Ridlehoover (WR) and Meital Kupfer (MK) are the core team for this review. EL is a research associate with experience in the conflict and development sector. SS is a systematic review and quantitative methods expert, with over a decade of experience in designing, managing and analysing quantitative research, including meta‐analyses. AS brings relevant field experience as a practitioner in social cohesion in fragile context and co‐led the development of previous systematic reviews and evidence and gap maps. WR is a research associate with experience in supporting the development, data analysis and management of systematic review projects. MK is a research associate with field experience in the sector of women's empowerment in FCAS.

Please note that this is the recommended optimal review team composition.

- Content: EL, AS, WR, MK, Nkululeko Tshabalala (NT), Promise Nduku (PN) developed the content of the review with inputs and quality assurance from SS and Laurenz Langer (LL) and with quality assurance from Bidisha Barooah (BB) and Birte Snilsveit (BS).
- Systematic review methods: EL, SS, AS, WR and MK drafted the review methods with inputs and support from the following experts: Laurenz Langer (Qualitative method expert), Bidisha Barooah (Senior Evaluation Specialist) and Sean Grant (GRADE methodology expert).
- Qualitative evidence synthesis: LL designed and oversaw the qualitative review component. NT managed and co‐ordinated the research process and activities. PN, MK, NT conducted the search for qualitative evidence, screening, and critical appraisal. NT, PN, LL and a pool of consultants conducted the thematic synthesis.
- Statistical analysis: SS oversaw the statistical analysis. SS, EL, WR, MK and a pool of consultants extracted effects data, with quality assurance from SS, while the core team led outcome classification and grouping with quality assurance and inputs from SS.
- Information retrieval: the core team developed the initial search strings and quality assured by John Eyers (Information specialist). EL, WR and MK led screening, grey literature review, snowballing and references checks with the support of a pool of consultants and under the oversight of AS.

## DECLARATIONS OF INTEREST

There are no reported conflicts of interest on this review. Several of the review authors are involved with the International Development Coordination Group of the Campbell Collaboration. However, the IDCG editor for this review is not involved in the review. The IDCG's independent cochair will also independently assure this review.

## PRELIMINARY TIMEFRAME

The planned time frame for this review is as follows:

- Draft and final protocols: December 2020–January 2021
- Draft and final report: June–October 2021

## PLANS FOR UPDATING THIS REVIEW

The search of this study has been completed in February 2021 based on a search strategy developed in December 2020. The core team will explore opportunities for funding an update of this review upon submission of the final report in 2021.

## Supporting information

Supporting information.

## Footnotes

1 The team used the following keywords for the literature search: ‘gender’, ‘women empowerment’, ‘adolescent girls’ empowerment’, ‘gender‐based violence’, ‘intimate partner violence’.

2 The FSI is an multifaceted indicator of state fragility managed by the Fund for Peace that captures content analysis, qualitative and quantitative data to ‘highlight not only the normal pressures that all states experience, but also … identify when those pressures are outweighing a states' capacity to manage those pressures to … make political risk assessment and early warning of conflict accessible to policy‐makers and the public at large’.

3 As per UNDP definition: ‘The GII is an inequality index. It measures gender inequalities in three important aspects of human development—reproductive health, measured by maternal mortality ratio and adolescent birth rates; empowerment, measured by proportion of parliamentary seats occupied by females and proportion of adult females and males aged 25 years and older with at least some secondary education; and economic status, expressed as labour market participation and measured by labour force participation rate of female and male populations aged 15 years and older. The GII is built on the same framework as the IHDI—to better expose differences in the distribution of achievements between women and men. It measures the human development costs of gender inequality. Thus the higher the GII value the more disparities between females and males and the more loss to human development’.

4 This did not rule out other synthesis approaches such as framework synthesis (Brunton et al., 2020).

5 Excludes studies of critical overall quality
