# Supplementary material for: Strengthening women's empowerment and gender equality in fragile contexts towards peaceful and inclusive societies: A systematic review and meta‐analysis
Source: Campbell Syst Rev. 2022 Mar 8;18(1):e1214. doi: 10.1002/cl2.1214 (PMC8904729; doi:10.1002/cl2.1214)
Supplement: Supplementary file 1 — Supporting information. [file CL2-18-e1214-s001.docx]

# Online appendixes

**Online appendix A: Qualitative analysis tool**

https://www.3ieimpact.org/sites/default/files/2021-10/SR47-Online-appendix-A-Qualitative-analysis-tool.pdf

**Online appendix B: Data extraction tools**

https://www.3ieimpact.org/sites/default/files/2021-10/SR47-Online-appendix-B-Data-extraction-tools.pdf

**Online appendix C: Risk of bias assessment tool**

https://www.3ieimpact.org/sites/default/files/2021-10/SR47-Online-appendix-C-Risk-of-bias-assessment-tool.pdf

**Online appendix D: Title and abstract screening procedure**

https://www.3ieimpact.org/sites/default/files/2021-10/SR47-Online-appendix-D-Title-and-abstract-screening-procedure.pdf

**Online appendix E: List of terms for the search strategy**

https://www.3ieimpact.org/sites/default/files/2021-10/SR47-Online-appendix-E-List-of-terms-for-the-search-strategy.pdf

**Online appendix F: Search Strategies**

https://www.3ieimpact.org/sites/default/files/2021-10/SR47-Online-appendix-F-Search-Strategies.pdf

**Online appendix G: Full Text Screening Checklist**

https://www.3ieimpact.org/sites/default/files/2021-10/SR47-Online-appendix-G-Full-Text-Screening-Checklist.pdf

**Online appendix H: List of Outcomes**

https://www.3ieimpact.org/sites/default/files/2021-10/SR47-Online-appendix-H-List-of-Outcomes.pdf

**Online appendix I: Additional Background Information**

https://www.3ieimpact.org/sites/default/files/2021-10/SR47-Online-appendix-I-Additional-Background-Information.pdf

**Online appendix J: List of excluded studies at full text screening**

https://www.3ieimpact.org/sites/default/files/2021-10/SR47-Online-appendix-J-List-of-excluded-studies-at-full-text-screening.pdf

**Online appendix K: Descriptive Extraction Dataset**

The dataset is available [here](https://docs.google.com/spreadsheets/d/1tVLjpq8FT-5hsW7uPrrV-WhiTsxJdxL6nc4PQeifYxU/edit#gid=531622095) and will be granted access upon request to the corresponding author.

**Online appendix L: Quantitative and Risk of Bias Datasets**

The dataset is available [here](https://docs.google.com/spreadsheets/d/1XxFB6crDcyDgAAv9q468m7kIUFgkKy8TgzEu76pWG1k/edit#gid=1925676659) and will be granted access upon request to the contact author.

**Online appendix M: Report of qualitative coding**

The dataset will be granted access upon request to the contact author.

**Online appendix N: Overview of descriptive and analytical themes**

https://www.3ieimpact.org/sites/default/files/2021-10/SR47-Online-appendix-N-Overview-of-descriptive-and-analytical-themes.pdf

**Online appendix O: Characteristics of included studies**

https://www.3ieimpact.org/sites/default/files/2021-10/SR47-Online-appendix-O-Characteristics-of-included-studies.pdf

**Online appendix P: Studies characteristics tables per intervention**

https://www.3ieimpact.org/sites/default/files/2021-10/SR47-Online-appendix-P-Studies-characteristics-tables-per-intervention.pdf

**Online appendix Q: Summary table of effects per intervention**

https://www.3ieimpact.org/sites/default/files/2021-10/SR47-Online-appendix-Q-Summary-table-of-effects-per-intervention.pdf

# References

## Included studies

Abramsky, T., Devries, K., Kiss, L., Nakuti, J., Kyegombe, N., Starmann, E., Cundill, B., Francisco, L., Kaye, D., and Musuya, T. (2014). Findings from the SASA! Study: A Cluster Randomized Controlled Trial to Assess the Impact of A Community Mobilization Intervention to Prevent Violence Against Women and Reduce HIV Risk in Kampala, Uganda. *BMC Medicine*, *12*(1), 1–17.

Adoho, F., Chakravarty, S., Korkoyah, D. T., Lundberg, M., and Tasneem, A. (2014). *The Impact of An Adolescent Girls Employment Program: The EPAG Project In Liberia* (No. 6832; Policy Research Working Paper). The World Bank.

Ahmed, A. U., Quisumbing, A. R., Nasreen, M., Hoddinott, J., and Bryan, E. (2009). *Comparing Food and Cash Transfers to The Ultra Poor in Bangladesh* (No. 163; Research Monograph). International Food Policy Research Institute.

Alemu, S. H., Van Kempe, L., and Ruben, R. (2018). Women Empowerment Through Self-help Groups: The Bittersweet Fruits of Collective Apple Cultivation in Highland Ethiopia. *Journal of Human Development and Capabilities*, *19*(3), 308–330.

Ambler, K., and De Brauw, A. (2017). *The Impacts of Cash Transfers on Women’s Empowerment: Learning from Pakistan’s BISP Program* (No. 1702; Social Protection and Labour Discussion Paper). World Bank.

Amin, S., Ahmed, J., Saha, J., Hossain, M., and Haque, E. (2016). *Delaying Child Marriage Through Community-based Skills-development Programs for Girls: Results from A Randomized Controlled Study in Rural Bangladesh*. Population Council.

Ashraf, N., Bau, N., Low, C., and McGinn, K. (2018). *Negotiating A Better Future: How Interpersonal Skills Facilitate Intergenerational Investment* (No. 18–104; Working Paper, pp. 1095–1151). Harvard Business School.

Ashraf, N., Bau, N., Low, C., and McGinn, K. (2020). Negotiating A Better Future: How Interpersonal Skills Facilitate Intergenerational Investment. *The Quarterly Journal of Economics*, *135*(2), 1095–1151.

Austrian, K., and Muthengi, E. (2014). Can Economic Assets Increase Girls’ Risk of Sexual Harassment? Evaluation Results from A Social, Health and Economic Asset-building Intervention for Vulnerable Adolescent Girls in Uganda. *Children and Youth Services Review*, *47*, 168–175.

Austrian, K., Soler-Hampejsek, E., Behrman, J. R., Digitale, J., Hachonda, N. J., Bweupe, M., and Hewett, P. C. (2020). The Impact of The Adolescent Girls Empowerment Program (AGEP) On Short- And Long-term Social, Economic, Education and Fertility Outcomes: A Cluster Randomized Controlled Trial in Zambia. *BMC Public Health*, *20*(1), 1–15.

Baird, S., McIntosh, C., and Özler, B. (2010). *Cash or Condition? Evidence from A Cash Transfer Experiment* (Impact Evaluation Series, pp. 1709–1753) [45]. World Bank.

Bandiera, O., Buehren, N., Burgess, R., Goldstein, M., Gulesci, S., Rasul, I., and Sulaiman, M. (2012). *Empowering Adolescent Girls: Evidence from A Randomized Control Trial in Uganda*. World Bank.

Bandiera, O., Buehren, N., Goldstein, M. P., Rasul, I., and Smurra, A. (2018). *The Economic Lives of Young Women in The Time of Ebola: Lessons from An Empowerment Program*. The World Bank.

Bandiera, O., Burgess, R., Das, N., Gulesci, S., Rasul, I., and Sulaiman, M. (2017). Labour Markets and Poverty in Village Economies. *The Quarterly Journal of Economics*, *132*(2), 811–870.

Bass, J., Murray, S., Cole, G., Bolton, P., Poulton, C., Robinette, K., Seban, J., Falb, K., and Annan, J. (2016). Economic, Social and Mental Health Impacts of An Economic Intervention for Female Sexual Violence Survivors in Eastern Democratic Republic of Congo. *Global Mental Health*, *3*.

Bastian, G., Bianchi, I., Goldstein, M., and Montalvao, J. (n.d.). *Short-term Impacts of Improved Access to Mobile Savings, with and Without Business Training: Experimental Evidence from Tanzania* (No. 478; Working Paper, p. 24). Centre for Global Development.

Beaman, L., Duflo, E., Pande, R., and Topalova, P. (2012). Female Leadership Raises Aspirations and Educational Attainment for Girls: A Policy Experiment in India. *Science*, *335*(6068), 582–586.

Beaman, L., Karlan, D., and Thuysbaert, B. (2014). *Saving for A (Not So) Rainy Day: A Randomized Evaluation of Savings Groups in Mali* (No. 20600; Working Paper). National Bureau of Economic Research.

Beath, A., Christia, F., and Enikolopov, R. (2015). The National Solidarity Programme: Assessing the Effects of Community-driven Development in Afghanistan. *International Peacekeeping*, *22*(4), 302–320.<https://doi.org/10.1080/13533312.2015.1059287>

Bedoya, G., Coville, idan, Haushofer, J., Isaqzadeh, M., and Shapiro, J. (n.d.). *Reducing Poverty and Improving Gender Equality Through A Big Push: Experimental Evidence from Afghanistan*. World Bank.

Bedoya, G., Coville, A., Haushofer, J., Isaqzadeh, M. R., and Shapiro, J. (2019). *No Household Left Behind: Afghanistan Targeting the Ultra Poor Impact Evaluation* (No. 8877; Policy Research Working Paper). The World Bank.

Blattman, C., Green, E. P., Jamison, J., Lehmann, M. C., and Annan, J. (2016). The Returns to Microenterprise Support Among the Ultrapoor: A Field Experiment in Postwar Uganda. *American Economic Journal: Applied Economics*, *8*(2), 35–64.

Bold, M. van den, Dillon, A., Olney, D., Ouedraogo, M., Pedehombga, A., and Quisumbing, A. (2015). Can Integrated Agriculture-nutrition Programmes Change Gender Norms on Land and Asset Ownership? Evidence from Burkina Faso. *The Journal of Development Studies*, *51*(9), 1155–1174.<https://doi.org/10.1080/00220388.2015.1036036>

Bonilla, J., Zarzur, R. C., Handa, S., Nowlin, C., Peterman, A., Ring, H., and Seidenfeld, D. (2017). Cash for Women’s Empowerment? A Mixed-methods Evaluation of The Government of Zambia’s Child Grant Program. *World Development*, *95*, 55–72.<https://doi.org/10.1016/j.worlddev.2017.02.017>

Bose, K. S., and Sarma, R. H. (1975). Delineation of The Intimate Details of The Backbone Conformation of Pyridine Nucleotide Coenzymes in Aqueous Solution. *Biochemical and Biophysical Research Communications*, *66*(4), 1173–1179.<https://doi.org/10.1016/0006-291x(75)90482-9>

Breisinger, C., Gilligan, D., Karachiwalla, N., Kurdi, S., El-Enbaby, H., Jilani, A., and Thai, G. (2018). *Impact Evaluation Study for Egypt’s Takaful And Karama Cash Transfer Program: Part 1: Quantitative Report* (No. 16; Working Paper). Intl Food Policy Res Inst.

Brooks, W., Donovan, K., Johnson, T. R., and Oluoch-Aridi, J. (2020). *Cash Transfers as A Response To COVID-19: Experimental Evidence from Kenya*.

Buehren, N., Chakravarty, S., Goldstein, M., Slavchevska, V., and Sulaiman, M. (2017). *Adolescent Girls’ Empowerment in Conflict-affected Settings: Experimental Evidence from South Sudan*.

Buehren, N., Goldstein, M., Gulesci, S., Sulaiman, M., and Yam, V. (2017). *Evaluation Of an Adolescent Development Program for Girls in Tanzania* (No. 7961; Policy Research Working Paper). The World Bank.

Carney, C., and Carney, M. H. (2018). Impact of Soil Conservation Adoption on Intra‐household Allocations in Zambia. *Review of Development Economics*, *22*(4), 1390–1408.

Chinen, M., and Elmeski, M. (2016). *Evaluation of The Transformative Potential of Positive Gender Socialization in Education for Peace Building.* American Institutes for Research.

Cilliers, J., Dube, O., and Siddiqi, B. (2018). *Can the Wounds of War Be Healed? Experimental Evidence on Reconciliation in Sierra Leone | 3ie* (No. 75; 3ie Impact Evaluation Report). 3ie.<https://www.3ieimpact.org/evidence-hub/publications/impact-evaluations/can-wounds-war-be-healed-experimental-evidence>

Clark, S., Kabiru, C. W., Laszlo, S., and Muthuri, S. (2019). The Impact of Childcare on Poor Urban Women’s Economic Empowerment in Africa. *Demography*, *56*(4), 1247–1272.

Clayton, A. (2015). Women’s political engagement under quota-mandated female representation: Evidence from a randomized policy experiment. *Comparative Political Studies*, *48*(3), 333–369.

Corps, M. (2015). *Improving Child and Maternal Health: Why Adolescent Girl Programming Matters. Post-intervention Evidence from Niger*. Mercy Corps.

Croke, K., Garcia Mora, M. E., Goldstein, M., Mensah, E. R., and O’Sullivan, M. (2020). *Up Before Dawn: Experimental Evidence from A Cross-border Trader Training at The Democratic Republic of Congo? Rwanda Border* (No. 9123; Policy Research Working Paper). World Bank.

Croke, K., Goldstein, M., and Holla, A. (2017). *Can Job Training Decrease Women’s Self-defeating Biases? Experimental Evidence from Nigeria* (No. 8141; Policy Research Working Paper). World Bank.

Das, A., Mogford, E., Singh, S. K., Barbhuiya, R. A., Chandra, S., and Wahl, R. (2012). Reviewing Responsibilities and Renewing Relationships: An Intervention with Men on Violence Against Women in India. *Culture, Health and Sexuality*, *14*(6), 659–675.

De Hoop, T., Van Kempen, L., Linssen, R., and Van Eerdewijk, A. (2010). *Women’s Autonomy and Subjective Well-being in India: How Village Norms Shape the Impact of Self-help Groups* (No. 25921; MPRA Paper). MPRA.

Decker, M. R., Wood, S. N., Hameeduddin, Z., Kennedy, S. R., Perrin, N., Tallam, C., Akumu, I., Wanjiru, I., Asira, B., Frankel, A., Omondi, B., Case, J., Clough, A., Otieno, R., Mwiti, M., and Glass, N. (2020). Safety Decision-making and Planning Mobile App for Intimate Partner Violence Prevention and Response: Randomised Controlled Trial in Kenya. *BMJ Global Health*, *5*(7), e002091.<https://doi.org/10.1136/bmjgh-2019-002091>

Decker, M. R., Wood, S. N., Ndinda, E., Yenokyan, G., Sinclair, J., Maksud, N., Ross, B., Omondi, B., and Ndirangu, M. (2018). Sexual Violence Among Adolescent Girls and Young Women in Malawi: A Cluster-randomized Controlled Implementation Trial of Empowerment Self-defence Training. *BMC Public Health*, *18*(1), 1–12.

Desaib, R. M., and Joshic, S. (2013). *Collective Action and Community Development: Evidence from Women’s Self-help Groups in Rural India*.

Deininger, K., & Liu, Y. (2013). Economic and Social Impacts of an Innovative Self-Help Group Model in India. World Development, 43, 149–163. <https://doi.org/10.1016/j.worlddev.2012.09.019>

Duflo, E., Banerjee, A., Glennerster, R., and Kinnan, C. G. (n.d.). *The Miracle of Microfinance? Evidence from A Randomized Evaluation* (No. 18950; NBER Working Paper Series, p. 62). NBER.

Dunkle, K., Stern, E., Chatterji, S., and Heise, L. (2020). Effective Prevention of Intimate Partner Violence Through Couples Training: A Randomised Controlled Trial of Indashyikirwa In Rwanda. *BMJ Global Health*, *5*(12), e002439.<https://doi.org/10.1136/bmjgh-2020-002439>

Erulkar, A., and Medhin, G. (2017). Evaluation of A Safe Spaces Program for Girls in Ethiopia. *Girlhood Studies*, *10*(1), 107–125.<https://doi.org/10.3167/ghs.2017.100108>

Eze Eze, D. (2019). Microfinance Programs and Domestic Violence in Northern Cameroon; The Case of The Familial Rural Income Improvement Program. *Review of Economics of the Household*, *17*(3), 947–967.<https://doi.org/10.1007/s11150-017-9393-x>

Field, E., Jayachandran, S., and Pande, R. (2010). Do Traditional Institutions Constrain Female Entrepreneurship? A Field Experiment on Business Training in India. *American Economic Review*, *100*(2), 125–129.<https://doi.org/10.1257/aer.100.2.125>

Figueroa, M. E., Poppe, P., Carrasco, M., Pinho, M. D., Massingue, F., Tanque, M., and Kwizera, A. (2016). Effectiveness of Community Dialogue in Changing Gender and Sexual Norms for HIV Prevention: Evaluation of The Tchova Tchova Program in Mozambique. *Journal of Health Communication*, *21*(5), 554–563.<https://doi.org/10.1080/10810730.2015.1114050>

Fuller, R. (2014). *Strengthening and Linking Women-Led Efforts to Promote Women’s Property and Literacy Rights in Sierra Leone—Project Effectiveness Review—Summary Report* (p. 17). Oxfam.

Gelagay, D. (n.d.). *Did conditional cash transfers in the Productive Safety Net Program empower women in Tigray, north-east Ethiopia?* (Discussion Paper, p. 31). Institute of Development Policy.

Gibbs, A., Corboz, J., Chirwa, E., Mann, C., Karim, F., Shafiq, M., Mecagni, A., Maxwell-Jones, C., Noble, E., and Jewkes, R. (2020). The Impacts of Combined Social and Economic Empowerment Training on Intimate Partner Violence, Depression, Gender Norms and Livelihoods Among Women: An Individually Randomised Controlled Trial and Qualitative Study in Afghanistan. *BMJ Global Health*, *5*(3), e001946.<https://doi.org/10.1136/bmjgh-2019-001946>

Glass, N., Perrin, N. A., Kohli, A., Campbell, J., and Remy, M. M. (2017). Randomised Controlled Trial of A Livestock Productive Asset Transfer Programme to Improve Economic and Health Outcomes and Reduce Intimate Partner Violence in A Post conflict Setting. *BMJ Global Health*, *2*(1), e000165.<https://doi.org/10.1136/bmjgh-2016-000165>

Gobin, V. J., Santos, P., and Toth, R. (2017). No Longer Trapped? Promoting Entrepreneurship Through Cash Transfers to Ultra-poor Women in Northern Kenya. *American Journal of Agricultural Economics*, *99*(5), 1362–1383.<https://doi.org/10.1093/ajae/aax037>

Gottlieb, J. (2016). Why Might Information Exacerbate the Gender Gap in Civic Participation? Evidence from Mali. *World Development*, *86*, 95–110.<https://doi.org/10.1016/j.worlddev.2016.05.010>

Green, D. P., Wilke, A. M., and Cooper, J. (2020). Countering Violence Against Women by Encouraging Disclosure: A Mass Media Experiment in Rural Uganda. *Comparative Political Studies*, *53*(14), 2283–2320.<https://doi.org/10.1177/0010414020912275>

Green, E. P., Blattman, C., Jamison, J., and Annan, J. (2015). Women’s Entrepreneurship and Intimate Partner Violence: A Cluster Randomized Trial of Microenterprise Assistance and Partner Participation in Post-conflict Uganda (SSM-D-14-01580R1). *Social Science and Medicine*, *133*, 177–188.<https://doi.org/10.1016/j.socscimed.2015.03.042>

Halim, N., Mzilangwe, E. S., Reich, N., Badi, L., Simmons, E., Servidone, M., II, N. B. H., Kawemama, P., and Messersmith, L. J. (2019). *Together to End Violence Against Women in Tanzania: Results of A Pilot Cluster Randomized Controlled Trial to Evaluate Preliminary Effectiveness of Interpersonal and Community Level Interventions to Address Intimate Partner Violence*. 1653–1668.

Handa, S., Peterman, A., Huang, C., Halpern, C., Pettifor, A., & Thirumurthy, H. (2015). Impact of the Kenya Cash Transfer for Orphans and Vulnerable Children on early pregnancy and marriage of adolescent girls. *Social Science & Medicine (1982)*, *141*, 36–45. <https://doi.org/10.1016/j.socscimed.2015.07.024>

Haushofer, J., and Shapiro, J. (n.d.). *The Long-term Impact of Unconditional Cash Transfers: Experimental Evidence from Kenya* (p. 64).

Heath, R., Hidrobo, M., and Roy, S. (2020). Cash Transfers, Polygamy, And Intimate Partner Violence: Experimental Evidence from Mali. *Journal of Development Economics*, *143*, 102410.<https://doi.org/10.1016/j.jdeveco.2019.102410>

Hossain, M., Zimmerman, C., Kiss, L., Abramsky, T., Kone, D., Bakayoko-Topolska, M., Annan, J., Lehmann, H., and Watts, C. (2014). Working with Men to Prevent Intimate Partner Violence in A Conflict-affected Setting: A Pilot Cluster Randomized Controlled Trial in Rural Côte D’ivoire. *BMC Public Health*, *14*(1), 339.<https://doi.org/10.1186/1471-2458-14-339>

Ifelunini, I. A., and Wosowei, E. C. (2012). Does Micro Finance Reduce Poverty Among Women Entrepreneurs in South-south Nigeria? Evidence from Propensity Score Matching Technique. *European Journal of Business and Management*, *4*, 13.

Iqbal, T., Farooq, S., and Padda, I. U. H. (2021). Can Empowerment Be Enhanced by Putting Cash in The Hands of Poor Women? Learning from Pakistan’s BISP Program. *The European Journal of Development Research*, *33*(3), 760–792.<https://doi.org/10.1057/s41287-020-00320-w>

Ismayilova, L., Karimli, L., Gaveras, E., Tô-Camier, A., Sanson, J., Chaffin, J., and Nanema, R. (2018). An Integrated Approach to Increasing Women’s Empowerment Status and Reducing Domestic Violence: Results of A Cluster-randomized Controlled Trial in A West African Country. *Psychology of Violence*, *8*(4), 448–459.<https://doi.org/10.1037/vio0000136>

Jassal, N. (2020). Gender, Law Enforcement, And Access to Justice: Evidence from All-women Police Stations in India. *American Political Science Review*, *114*(4), 1035–1054.<https://doi.org/10.1017/S0003055420000684>

Johnson, S. W. (2018). Post-conflict Reconstruction, Microfinance and Democratic Engagement. *Peace Economics, Peace Science and Public Policy*, *24*(3), 20170048.<https://doi.org/10.1515/peps-2017-0048>

Karimli, L., Lecoutere, E., Wells, C. R., and Ismayilova, L. (2021). More Assets, More Decision-making Power? Mediation Model in A Cluster-randomized Controlled Trial Evaluating the Effect of The Graduation Program on Women’s Empowerment in Burkina Faso. *World Development*, *137*, 105159.<https://doi.org/10.1016/j.worlddev.2020.105159>

Karlan, D., Savonitto, B., Thuysbaert, B., and Udry, C. (2017). Impact of savings groups on the lives of the poor. *Proceedings of the National Academy of Sciences*, *114*(12), 3079–3084.

Kumar, N., Nguyen, P. H., Harris, J., Harvey, D., Rawat, R., and Ruel, M. T. (2018). What It Takes: Evidence from A Nutrition- And Gender-sensitive Agriculture Intervention in Rural Zambia. *Journal of Development Effectiveness*, *10*(3), 341–372.<https://doi.org/10.1080/19439342.2018.1478874>

Larson, A. M., Solis, D., Duchelle, A. E., Atmadja, S., Resosudarmo, I. A. P., Dokken, T., and Komalasari, M. (2018). Gender Lessons for Climate Initiatives: A Comparative Study Of REDD+ Impacts on Subjective Wellbeing. *World Development*, *108*, 86–102.<https://doi.org/10.1016/j.worlddev.2018.02.027>

Laudati, A., Mvukiyehe, E., and van der Windt, P. (2018). *Participatory Development in Fragile and Conflict-affected Contexts: An Impact Evaluation of the Tuungane 1 Program in the Democratic Republic of the Congo*.

Lecoutere, E., and Wuyts, E. (2021a). Confronting the Wall of Patriarchy: Does Participatory Intrahousehold Decision Making Empower Women in Agricultural Households? *The Journal of Development Studies*, *57*(6), 882–905.<https://doi.org/10.1080/00220388.2020.1849620>

Lecoutere, E., and Wuyts, E. (2021b). Confronting the Wall of Patriarchy: Does Participatory Intrahousehold Decision Making Empower Women in Agricultural Households? *The Journal of Development Studies*, *57*(6), 882–905.<https://doi.org/10.1080/00220388.2020.1849620>

Leight, J., Deyessa, N., Verani, F., Tewolde, S., and Sharma, V. (2021). Community-level Spill over Effects of An Intervention to Prevent Intimate Partner Violence and HIV Transmission in Rural Ethiopia. *BMJ Global Health*, *6*(1), e004075.<https://doi.org/10.1136/bmjgh-2020-004075>

Lombardini, S., and Bowman, K. (2015). *Women’s Empowerment in Pakistan: Impact Evaluation of The Empowering Small-Scale Producers in The Dairy Sector Project*. Oxfam GB.<http://hdl.handle.net/10546/580463>

Lombardini, S., and Yoshikawa, K. (2015). *Women’s Empowerment in Uganda: Impact Evaluation of The Project ‘Piloting Gender Sensitive Livelihoods in Karamoja.’* Oxfam GB.<http://hdl.handle.net/10546/592575>

Lubega, P., Nakakawa, F., Narciso, G., Newman, C., and Kityo, C. (2017). *Inspiring women: Experimental evidence on sharing entrepreneurial skills in Uganda* (No. 2017; TEP Working Paper, p. 57).

Lwamba, E., Ridlehoover, W., Kupfer, M., Shisler, S., Sonnenfeld, A., Langer, L., Eyers, J., Grant, S., & Barooah, B. (2021). Protocol: Strengthening women’s empowerment and gender equality in fragile contexts towards peaceful and inclusive societies: A systematic review and meta-analysis. Campbell Systematic Reviews, 17(3), e1180. https://doi.org/10.1002/cl2.1180

Maitra, P., and Mani, S. (2017). Learning and Earning: Evidence from A Randomized Evaluation in India. *Labour Economics*, *45*, 116–130.<https://doi.org/10.1016/j.labeco.2016.11.007>

Malaeb, B., and Uzor, E. (2017). *Multidimensional impact evaluation: A randomized control trial on conflict-affected women in Northern Uganda* (Working Paper). Centre for the Study of the Economies of Africa.

Marniemi, J., and Parkki, M. G. (1975). Radiochemical Assay of Glutathione S-epoxide Transferase and Its Enhancement by Phenobarbital in Rat Liver In Vivo. *Biochemical Pharmacology*, *24*(17), 1569–1572.<https://doi.org/10.1016/0006-2952(75)90080-5>

Mekonnen, T. (2017). *Financing rural households and its impact: Evidence from randomized field experiment data* (Working Paper). Maastricht University.

Mukherjee, A. K., and Kundu, A. (n.d.). *Impact of Swarna Jayanti Gram Swarojgar Yojona (SGSY) on Health, Education and Women Empowerment* (No. 33258; MPRA Paper, p. 23). Munich Personal RePEc Archive.

Müller, A., Pape, U., and Ralston, L. (2019). *Broken Promises: Evaluating an Incomplete Cash Transfer Program* (Policy Research Working Paper). The World Bank.<https://elibrary.worldbank.org/doi/abs/10.1596/1813-9450-9016>

Murray, L. K., Kane, J. C., Glass, N., Wyk, S. S. van, Melendez, F., Paul, R., Danielson, C. K., Murray, S. M., Mayeya, J., Simenda, F., and Bolton, P. (2020). Effectiveness of The Common Elements Treatment Approach (CETA) In Reducing Intimate Partner Violence and Hazardous Alcohol Use in Zambia (VATU): A Randomized Controlled Trial. *PLOS Medicine*, *17*(4), e1003056.<https://doi.org/10.1371/journal.pmed.1003056>

Mvukiyehe, E. (2017). *Can Media Interventions Reduce Gender Gaps in Political Participation after Civil War? Evidence from a Field Experiment in Rural Liberia* (Policy Research Working Paper No. 7942). World Bank.

Nandi, A., Agarwal, P., Chandrashekar, A., and Harper, S. (2020). Access to Affordable Daycare And Women’s Economic Opportunities: Evidence from A Cluster-randomised Intervention in India. *Journal of Development Effectiveness*, *12*(3), 219–239.<https://doi.org/10.1080/19439342.2020.1773898>

Natali, L., Handa, S., Peterman, A., and Seidenfeld, D. (2016). *Making Money Work: Unconditional cash transfers allow women to save and re-invest in rural Zambia INNOCENTI WORKING PAPERS* [Innocenti Working Paper].

Nkonya, E., Phillip, D., Mogues, T., Pender, J., and Kato, E. (2012). Impacts of Community-driven Development Programs on Income and Asset Acquisition in Africa: The Case of Nigeria. *World Development*, *40*(9), 1824–1838.<https://doi.org/10.1016/j.worlddev.2012.04.028>

Noble, E., Corboz, J., Gibbs, A., Mann, C., Mecagni, A., and Jewkes, R. (2019). *IMPACT EVALUATION OF WOMEN FOR WOMEN INTERNATIONAL’S ECONOMIC AND SOCIAL EMPOWERMENT PROGRAMME IN AFGHANISTAN: AN EVIDENCE BRIEF*.

Olajide, D., Ikenwilo, D., Obembe, O. B., Ibeji, N., and Akindola, R. (2016). *The Impact of A Rural Microcredit Scheme Targeting Women on Household Vulnerability and Empowerment: Evidence from South West Nigeria* (SSRN Scholarly Paper ID 3167363). Social Science Research Network.<https://papers.ssrn.com/abstract=3167363>

Olney, D. K., Bliznashka, L., Pedehombga, A., Dillon, A., Ruel, M. T., and Heckert, J. (2016). A 2-year Integrated Agriculture and Nutrition Program Targeted to Mothers of Young Children in Burkina Faso Reduces Underweight Among Mothers and Increases Their Empowerment: A Cluster-randomized Controlled Trial. *The Journal of Nutrition*, *146*(5), 1109–1117.<https://doi.org/10.3945/jn.115.224261>

Özler, B., Hallman, K., Guimond, M.-F., Kelvin, E. A., Rogers, M., and Karnley, E. (2020). Girl Empower – A Gender Transformative Mentoring and Cash Transfer Intervention to Promote Adolescent Wellbeing: Impact Findings from A Cluster-randomized Controlled Trial in Liberia. *SSM - Population Health*, *10*, 100527. [https://doi.org/10.1016/j.ssmph.2019.100527](%20https://doi.org/10.1016/j.ssmph.2019.100527)

Pradhan, M. A. H., and Sulaiman, J. (2017). Impact of Vulnerable Group Development (VGD) Program on Improvement of Woman Headed Household Consumption Diversity in Bangladesh. *JOURNAL OF SOCIAL SCIENCE RESEARCH*, *11*(1), 2292–2305.<https://doi.org/10.24297/jssr.v11i1.5746>

R, Q., Agnes, Akhter, A., F, H., John, Audrey, P., and Shalini, R. (2020). *Designing for Empowerment Impact in Agricultural Development Projects: Experimental Evidence from The Agriculture, Nutrition, And Gender Linkages (Angel) Project in Bangladesh*. Intl Food Policy Res Inst.

Roy, S., Hidrobo, M., Hoddinott, J., and Ahmed, A. (2019). Transfers, Behaviour Change Communication, And Intimate Partner Violence: Postprogram Evidence from Rural Bangladesh. *The Review of Economics and Statistics*, *101*(5), 865–877.<https://doi.org/10.1162/rest_a_00791>

Scales, P. C., Benson, P. L., Dershem, L., Fraher, K., Makonnen, R., Nazneen, S., Syvertsen, A. K., and Titus, S. (2013). Building Developmental Assets to Empower Adolescent Girls in Rural Bangladesh: Evaluation of Project Kishoree Kontha. *Journal of Research on Adolescence*, *23*(1), 171–184.<https://doi.org/10.1111/j.1532-7795.2012.00805.x>

Schensul, S. L., Singh, R., Schensul, J. J., Verma, R. K., Burleson, J. A., and Nastasi, B. K. (2015). Community Gender Norms Change as A Part of A Multilevel Approach to Sexual Health Among Married Women in Mumbai, India. *American Journal of Community Psychology*, *56*(1–2), 57–68.<https://doi.org/10.1007/s10464-015-9731-1>

Sieverding, M., and Elbadawy, A. (2016). Empowering Adolescent Girls in Socially Conservative Settings: Impacts and Lessons Learned from the Ishraq Program in Rural Upper Egypt. *Studies in Family Planning*, *47*(2), 129–144.

Smith, R. J., and Bryant, R. G. (1975). Metal Substitutions Incarbonic Anhydrase: A Halide Ion Probe Study. *Biochemical and Biophysical Research Communications*, *66*(4), 1281–1286.<https://doi.org/10.1016/0006-291x(75)90498-2>

Stark, L., Asghar, K., Seff, I., Yu, G., Tesfay Gessesse, T., Ward, L., Assazenew Baysa, A., Neiman, A., and Falb, K. L. (2018). Preventing Violence Against Refugee Adolescent Girls: Findings from A Cluster Randomised Controlled Trial in Ethiopia. *BMJ Global Health*, *3*(5), e000825.<https://doi.org/10.1136/bmjgh-2018-000825>

Stark, L., Seff, I., Asghar, K., Roth, D., Bakamore, T., MacRae, M., Fanton D’Andon, C., and Falb, K. L. (2018). Building Caregivers’ Emotional, Parental and Social Support Skills to Prevent Violence Against Adolescent Girls: Findings from A Cluster Randomised Controlled Trial in Democratic Republic of Congo. *BMJ Global Health*, *3*(5), e000824.<https://doi.org/10.1136/bmjgh-2018-000824>

Tanner, S., and O’Connor, M. (2017). *A Safe Place to Shine: Creating opportunities and raising the voice of adolescent girls in humanitarian settings*. International Rescue Committee.

Tarozzi, A., Desai, J., and Johnson, K. (2013). *On the Impact of Microcredit: Evidence from a Randomized Intervention in Rural Ethiopia* (Working Paper, p. 37).

Vaillant, J., Koussoubé, E., Roth, D., Pierotti, R., Hossain, M., and Falb, K. L. (2020). Engaging Men to Transform Inequitable Gender Attitudes and Prevent Intimate Partner Violence: A Cluster Randomised Controlled Trial in North and South Kivu, Democratic Republic of Congo. *BMJ Global Health*, *5*(5), e002223.<https://doi.org/10.1136/bmjgh-2019-002223>

Vigneri, M., and Lombardini, S. (n.d.). *Women’s empowerment in Mali: Impact evaluation of the educational project “Girls CAN - Promoting secondary education in West Africa”* (p. 50). Oxfam.

Weber, O., and Ahmad, A. (2014). Empowerment Through Microfinance: The Relation Between Loan Cycle and Level of Empowerment. *World Development*, *62*, 75–87.<https://doi.org/10.1016/j.worlddev.2014.05.012>

World Bank, McKenzie, D., Puerto, S., International Labour Organization, Odhiambo, F., and Innovations for Poverty Action. (2019). *Unpacking the Determinants of Entrepreneurship Development and Economic Empowerment for Women in Kenya*. International Initiative for Impact Evaluation (3ie).<http://www.3ieimpact.org/evidence-hub/publications/impact-evaluations/unpacking-determinants-entrepreneurship-development>

Yaron, G., Gordon, R., Best, J., and Choudhary, S. (2018). Microfinance for The Marginalized: The Impact of The Rojiroti Approach in India. *Enterprise Development and Microfinance*, *29*(1), 80–93.<https://doi.org/10.3362/1755-1986.17-00011>

## Linked Studies

Baird, S., Chirwa, E., de Hoop, J., and Özler, B. (2013). *Girl Power: Cash Transfers and Adolescent Welfare. Evidence from A Cluster-randomized Experiment in Malawi* (No. 19479; p. w19479). National Bureau of Economic Research.<http://www.nber.org/papers/w19479.pdf>

Baird, S., Chirwa, E., McIntosh, C., and Özler, B. (2009). *The Short-term Impacts of A Schooling Conditional Cash Transfer Program on The Sexual Behaviour of Young Women* (Policy Research Working Paper No. 40; p. 33). World Bank.<https://openknowledge.worldbank.org/bitstream/handle/10986/4281/WPS5089.pdf>

Bandiera, O., Buehren, N., Burgess, R., Goldstein, M., Gulesci, S., Rasul, I., and Sulaiman, M. (2020). Women’s Empowerment in Action: Evidence from A Randomized Control Trial in Africa. *American Economic Journal: Applied Economics*, *12*(1), 210–259.<https://doi.org/10.1257/app.20170416>

Bandiera, O., Burgess, R., Das, N., Gulesci, S., Rasul, I., and Sulaiman, M. (2013). *Can Basic Entrepreneurship Transform the Economic Lives of The Poor?* (IZA Discussion Paper No. 7386; p. 56). IZA.<https://www.econstor.eu/bitstream/10419/80628/1/745852602.pdf>

Banerjee, A., Duflo, E., Glennerster, R., & Kinnan, C. (2015). The Miracle of Microfinance? Evidence from a Randomized Evaluation. American Economic Journal: Applied Economics, 7(1), 22–53. <https://doi.org/10.1257/app.20130533>

Beath, A., Christia, F., and Enikolopov, R. (2013a). *Randomized Impact Evaluation Of Afghanistan’s National Solidarity Programme* (No. 81107). World Bank.

Beath, A., Christia, F., and Enikolopov, R. (2013b). Empowering Women Through Development Aid: Evidence From A Field Experiment In Afghanistan. *American Political Science Review*, *107*(3), 540–557.<https://doi.org/10.1017/S0003055413000270>

Blattman, C., Fiala, N., Berlin, D., and Martinez, S. (2013). *The Economic And Social Returns To Cash Transfers: Evidence From A Ugandan Aid Program* (p. 53).

Das, N., Yasmin, R., and Ara, J. (2013). *How Do Intrahousehold Dynamics Change When Assets Are Transferred To Women?* (IFPRI Discussion Paper No. 01317; p. 44).

De Hoop, T., van Kempen, L., Linssen, R., & van Eerdewijk, A. (2014). Women’s Autonomy and Subjective Well-Being: How Gender Norms Shape the Impact of Self-Help Groups in Odisha, India. Feminist Economics, 20(3), 103–135. <https://doi.org/10.1080/13545701.2014.893388>

Gibbs, A., Corboz, J., Shafiq, M., Marofi, F., Mecagni, A., Mann, C., Karim, F., Chirwa, E., Maxwell-Jones, C., and Jewkes, R. (2018). An Individually Randomized Controlled Trial To Determine The Effectiveness Of The Women For Women International Programme In Reducing Intimate Partner Violence And Strengthening Livelihoods Amongst Women In Afghanistan: Trial Design, Methods And Baseline Findings. *BMC Public Health*, *18*(1), 164.<https://doi.org/10.1186/s12889-018-5029-1>

Green, D. P., Wilke, A., and Cooper, J. (2018). *Silence Begets Violence: A Mass Media Experiment To Prevent Violence Against Women In Rural Uganda*. Newhaven, CT: Innovations for Poverty.

Green, E. P., Blattman, C., Jamison, J., and Annan, J. (2016). Corrigendum To “Women’s Entrepreneurship And Intimate Partner Violence: A Cluster Randomized Trial Of Microenterprise Assistance And Partner Participation In Post-conflict Uganda” [Soc. Sci. Med. 133 (2015) 177–188]. *Social Science and Medicine*, *148*, 139–141.<https://doi.org/10.1016/j.socscimed.2015.11.042>

Guilbert, N., Vaillant, J., Falb, K., Mallinga, P., and Roth, D. (2016). *Preventing Intimate-partner Violence: Impact Evaluation Of Engaging Men Through Accountable Practice In Eastern DRC*. [RIDIE-STUDY-ID-576d513b31538](https://doi.org/RIDIE-STUDY-ID-576d513b31538)

Haushofer, J., Ringdal, C., Shapiro, J. P., and Wang, X. Y. (2019). *Income Changes And Intimate Partner Violence: Evidence From Unconditional Cash Transfers In Kenya*. National Bureau of Economic Research.

Haushofer, J., and Shapiro, J. (2013). *Household Response To Income Changes: Evidence From An Unconditional Cash Transfer Program In Kenya* (pp. 1–57).

Haushofer, J., and Shapiro, J. (2016). The Short-term Impact Of Unconditional Cash Transfers To The Poor: Experimental Evidence From Kenya. *The Quarterly Journal of Economics*, *131*(4), 1973–2042.

Iqbal, T., Padda, I. U. H., and Farooq, S. (2020). Unconditional Cash Transfers And Women Empowerment: The Case Of Benazir Income Support Programme (BISP) In Pakistan. *Journal of Business and Social Review in Emerging Economies*, *6*(2), 401–418.

Karimli, L., Bose, B., and Kagotho, N. (2019). Integrated Graduation Program And Its Effect On Women And Household Economic Well-being: Findings From A Randomised Controlled Trial In Burkina Faso. *The Journal of Development Studies*, *56*(7), 1277–1294.<https://doi.org/10.1080/00220388.2019.1677887>

Lecoutere, E. (2018). *Improving intrahousehold cooperation for efficient smallholder farming. A field experiment in central Uganda* [Working Paper]. Institute of Development Policy.

Roy, S., Ara, J., Das, N., and Quisumbing, A. R. (2013). *Asset Transfers And Intrahousehold Dynamics: Evidence From Brac’s ‘Targeting The Ultra Poor’program In Bangladesh*.

Roy, S., Ara, J., Das, N., and Quisumbing, A. R. (2015). “Flypaper Effects” In Transfers Targeted To Women: Evidence From Brac’s “Targeting The Ultra Poor” Program In Bangladesh. *Journal of Development Economics*, *117*, 1–19.

Sarah, B., McIntosh, C. T., and Özler, B. (2017). *When The Money Runs Out: Do Cash Transfers Have Sustained Effects On Human Capital Accumulation?* (No. 68; WPS). Center for Effective Global Action.<https://escholarship.org/content/qt2rd3f9jv/qt2rd3f9jv.pdf>

Sharma, V., Leight, J., Verani, F., Tewolde, S., and Deyessa, N. (2020). Effectiveness Of A Culturally Appropriate Intervention To Prevent Intimate Partner Violence And HIV Transmission Among Men, Women, And Couples In Rural Ethiopia: Findings From A Cluster-randomized Controlled Trial. *PLoS Medicine*, *17*(8), e1003274.

Stark, L., Seff, I., Assezenew, A., Eoomkham, J., Falb, K., and Ssewamala, F. M. (2018). Effects Of A Social Empowerment Intervention On Economic Vulnerability For Adolescent Refugee Girls In Ethiopia. *Journal of Adolescent Health*, *62*(1), S15–S20.

Tanner, S., and O’Connor, M. (2017a). *A Safe Place To Shine: Creating Opportunities And Raising Voices Of Adolescent Girls In Humanitarian Settings*. International Rescue Committee.

Tanner, S., and O’Connor, M. (2017b). *A Safe Place To Shine: Learning From The COMPASS Programme In Ethiopia*. International Rescue Committee.

Tarozzi, A., Desai, J., & Johnson, K. (2015). The Impacts of Microcredit: Evidence from Ethiopia. American Economic Journal: Applied Economics, 7(1), 54–89. <https://doi.org/10.1257/app.20130475>

Van der Windt, P. (2018). Can development aid empower women? Evidence from a field experiment in the Congo. *The Journal of Politics*, *80*(3), 1039–1044.

Van der Windt, P. (2020). *Assessing the Longer Term Impact of Community-Driven Development Programs: Evidence from a Field Experiment in the Democratic Republic of Congo* (No. 9140; Research Working Paper). World Bank.

Van der Windt, P., Humphreys, M., and de la Sierra, R. S. (2018). Gender Quotas In Development Programming: Null Results From A Field Experiment In Congo. *Journal of Development Economics*, *133*, 326–345.

Watts, C., Devries, K., Kiss, L., Abramsky, T., Kyegombe, N., and Michau, L. (2019). *The SASA! Study: A Cluster Randomised Trial To Assess The Impact Of A Violence And HIV Prevention Programme In Kampala, Uganda* (3ie Impact Evaluation Report No. 24). 3ie.<https://dataverse.harvard.edu/citation?persistentId=doi:10.7910/DVN/DRXLQF>

## Qualitative and process evaluations

Adoho, F., Chakravarty, S., Korkoyah, D. T., Lundberg, M., and Tasneem, A. (2014). The impact of an adolescent girls employment program: The EPAG project in Liberia. The World Bank.

Ahmed, A. U., Quisumbing, M. A. R., Hoddinott, J., Nasreen, M., and Bryan, E. (2007). Relative efficacy of food and cash transfers in improving food security and livelihoods of the ultra-poor in Bangladesh. WFP, World Food Programme.

Ahmed, A. U., Sraboni, E., and Shaba, F. K. (2014). Safety nets in Bangladesh: which form of transfer is most beneficial. Washington: International Food Policy Research Institute.

Amaral S., Bhalotraz S., and Prakash N. (2018). Gender, Crime And Punishment: Evidence From Women Police Stations In India. Essex: Economic And Social Research Council (ESRC), Research Centre On Microsocial Change (Misoc). Retrieved at:<https://ideas.repec.org/p>

Ambler, K., and De Brauw, A. (2017). The impacts of cash transfers on women’s empowerment: learning from Pakistan’s BISP program. World Bank.

Ayuku, D., Embleton, L., Koech, J., Atwoli, L., Hu, L., Ayaya, S., ... and Braitstein, P. (2014). The government of Kenya cash transfer for orphaned and vulnerable children: cross-sectional comparison of household and individual characteristics of those with and without. BMC international health and human rights, 14(1), 1-14.

Azadi, H., De Rudder, F., Vlassenroot, K., Nega, F., and Nyssen, J. (2017). Targeting international food aid programmes: The case of productive safety net programme in Tigray, Ethiopia. Sustainability, 9(10), 1716.

Baird, S., Chirwa, E., McIntosh, C., and Özler, B. (2015). What happens once the intervention ends? The medium-term impacts of a cash transfer programme in Malawi. 3ie Impact Evaluation Report, 27.

Baird, S., McIntosh, C., and Ozler, B. (2009). Designing cost-effective cash transfer programs to boost schooling among young women in Sub-Saharan Africa. World Bank Policy Research Working Paper, (5090).

Bandiera, O., Buehren, N., Goldstein, M. P., Rasul, I., and Smurra, A. (2019). The Economic Lives of Young Women in the Time of Ebola: Lessons from an Empowerment Program. The World Bank.

Barakat, S., and Strand, A. (2006). Mid-term evaluation report of the National Solidarity Programme (NSP), Afghanistan.

Bass, J., Bolton, P., Murray, S., Cole, G., Robinette, K., Poulton, C., ... and Annan, J. (2014). Study of effectiveness of a social-economic intervention for sexual violence survivors in Eastern DRC.

Bass, J., Murray, S., Cole, G., Bolton, P., Poulton, C., Robinette, K., ... and Annan, J. (2016). Economic, social and mental health impacts of an economic intervention for female sexual violence survivors in Eastern Democratic Republic of Congo. Global Mental Health, 3.

Beaman, L., Karlan, D., and Thuysbaert, B. (2014). Saving for a (not so) rainy day: A randomized evaluation of savings groups in Mali (No. w20600). National Bureau of Economic Research.

Bedoya, G., Coville, A., Haushofer, J., Isaqzadeh, M. R., and Shapiro, J. (2019). No household left behind: Afghanistan targeting the ultra poor impact evaluation. The World Bank.

Berlie, A. B. (2018). The Role of Productive Safety Net Programme (PSNP) in Improving Female-Headed Households' Food Security: The Case of Enebsie Sar Midir District of The Amhara Region, Ethiopia. Eastern Africa Social Science Research Review, 34(1), 109-136.

Bermudez, L., and Matuszeski, J. (2010). Ensuring continued success: Saving for Change in older program areas of Mali. Oxfam America, Boston, MA.

Bhatia, J., Jareer, N., and McIntosh, R. (2018). Community-driven development in Afghanistan: A case study of the national solidarity programme in Wardak. Asian Survey, 58(6), 1042-1065.

Blattman, C., Fiala, N., and Martinez, S. (2013). The economic and social returns to cash transfers: evidence from a Ugandan aid program. Columbia University, Departments of Political Science and International and Public Affairs.

Blattman, C., Green, E. P., Jamison, J., Lehmann, M. C., and Annan, J. (2016). The returns to microenterprise support among the ultrapoor: A field experiment in postwar Uganda. American economic journal: Applied economics, 8(2), 35-64.

Blattman, C., Green, E., Annan, J., and Jamison, J. (2013). Building women's economic and social empowerment through enterprise: an experimental assessment of the women's income generating support program in Uganda.

Boesen, I. W. (2004). From subjects to citizens: Local Participation in the national solidarity programme. Kabul: Afghanistan Research and Evaluation Unit.

BOMA (2018). An Analysis of the Endline Survey for the REAP Cohort Funded Through the Bill and Melinda Gates Foundation.

Bonilla, J., Zarzur, R. C., Handa, S., Nowlin, C., Peterman, A., Ring, H., ... and Team, Z. C. G. P. E. (2017). Cash for women’s empowerment? A mixed-methods evaluation of the government of Zambia’s child grant program. World Development, 95, 55-72.

Brady, M., Assaad, R., Ibrahim, B. L., Salem, A., Salem, R., and Zibani, N. (2007). Providing new opportunities to adolescent girls in socially conservative settings: The Ishraq program in rural Upper Egypt—full report.

Buchmann, N., Field, E., Glennerster, R., Nazneen, S., Pimkina, S., and Sen, I. (2016). The effect of conditional incentives and a girls’ empowerment curriculum on adolescent marriage, childbearing and education in rural Bangladesh: a community clustered randomized controlled trial. Working Paper.

Bureau of Applied Research In Anthropology and IPA (2008). Operational Evaluation of Saving for Change in Mali.

Chatterji, S., Stern, E., Dunkle, K., and Heise, L. (2020). Community activism as a strategy to reduce intimate partner violence (IPV) in rural Rwanda: Results of a community randomised trial. Journal of global health, 10(1).

Cherewick, M., and Glass, N. (2018). Caregiver and community insights on coping strategies used by adolescents living in conflict-affected communities. Global public health, 13(9), 1322-1336.

Cilliers, J., Dube, O., and Siddiqi, B. (2016). Reconciling after civil conflict increases social capital but decreases individual well-being. Science, 352(6287), 787-794.

Concern Worldwide (2017). Final Evaluation Report RAIN+ Project Concern Worldwide Zambia.

Deubel, T. F., and Boyer, M. (2019). Saving for Change in Mali: From women’s financial inclusion to public engagement. Oxfam Research Backgrounder series.

Deubel, T. F., and Boyer, M. (2020). Women’s Economic Empowerment and Political Accountability in Mali. IDS.

Diwan, F., Makana, G., McKenzie, D., and Paruzzolo, S. (2014). Invitation choice structure has no impact on attendance in a female business training program in Kenya. Plos one, 9(10), e109873.

Duby, Z., Zulu, C. N., and Austrian, K. (2016). Adolescent Girls Empowerment Programme in Zambia: Qualitative Evaluation Report.

Durr-E-Nayab, and Farooq, S. (2014). Effectiveness of cash transfer programmes for household welfare in Pakistan: The case of the Benazir Income Support Programme. The Pakistan Development Review, 53(2), 145-174.

ElDidi, H., El-Enbaby, H., Kassim, Y., Kurdi, S., Petesch, P., Moataz, Y., and Goessinger, K. Y. (2018). Impact evaluation study for Egypt's Takaful and Karama cash transfer program: Part 2: Qualitative Report (Vol. 15). Intl Food Policy Res Inst.

El-Enbaby, H., Gilligan, D., Karachiwalla, N., Kassim, Y., and Kurdi, S. (2019). Cash transfers and women’s control over decision-making and labor supply in Egypt (Vol. 25). Intl Food Policy Res Inst.

Emran, M. S., Robano, V., and Smith, S. C. (2014). Assessing the frontiers of ultrapoverty reduction: evidence from challenging the frontiers of poverty reduction/targeting the ultra-poor, an innovative program in Bangladesh. Economic Development and Cultural Change, 62(2), 339-380.

Falb, K. L., Tanner, S., Ward, L., Erksine, D., Noble, E., Assazenew, A., ... and Stark, L. (2016). Creating opportunities through mentorship, parental involvement, and safe spaces (COMPASS) program: multi-country study protocol to protect girls from violence in humanitarian settings. BMC public health, 16(1), 1-10.

Gelagay, M. D., and Lecoutere, E. (2019). Did conditional cash transfers in the Productive Safety Net Program empower women in Tigray, north-east Ethiopia?. IOB, Institute of Development Policy, University of Antwerp.

Gibbs, A., Corboz, J., and Jewkes, R. (2018). Factors associated with recent intimate partner violence experience amongst currently married women in Afghanistan and health impacts of IPV: a cross sectional study. BMC Public Health, 18(1), 1-10.

Gibbs, A., Corboz, J., Chirwa, E., Mann, C., Karim, F., Shafiq, M., ... and Jewkes, R. (2020). The impacts of combined social and economic empowerment training on intimate partner violence, depression, gender norms and livelihoods among women: an individually randomised controlled trial and qualitative study in Afghanistan. BMJ global health, 5(3), e001946.

Gibbs, A., Jewkes, R., Karim, F., Marofi, F., and Corboz, J. (2018). Understanding how Afghan women utilise a gender transformative and economic empowerment intervention: a qualitative study. Global public health, 13(11), 1702-1712.

Glass, N., Ramazani, P., Tosha, M., Mpanano, M., and Cinyabuguma, M. (2012). A Congolese–US participatory action research partnership to rebuild the lives of rape survivors and their families in eastern Democratic Republic of Congo. Global public health, 7(2), 184-195.

Gobin, V. J., and Santos, P. All together now: the impact of a multifaceted approach to poverty alleviation.

Gobin, V. J., Santos, P., and Toth, R. (2016). Poverty graduation with cash transfers: a randomized evaluation. Department of Economics Discussion Paper, 23, 16.

Gobin, V. J., Santos, P., and Toth, R. (2017). No longer trapped? Promoting entrepreneurship through cash transfers to ultra-poor women in northern Kenya. American Journal of Agricultural Economics, 99(5), 1362-1383.

Green, E. P., Blattman, C., Jamison, J., and Annan, J. (2015). Women's entrepreneurship and intimate partner violence: A cluster randomized trial of microenterprise assistance and partner participation in post-conflict Uganda (SSM-D-14-01580R1). Social science and medicine, 133, 177-188.

Handa, S., Tembo, G., Natali, L., Angeles, G., and Spektor, G. (2019). In search of the holy grail: Can unconditional cash transfers graduate households out of poverty in Zambia?.

Huber, M. S. and Zupancic, M. (2015). Women for Women International’s “Stronger Women, Stronger Afghanistan” Programme.

Humphreys, M., De La Sierra, R. S., and Van der Windt, P. (2012). Social and Economic Impacts of Tuungane Final Report on the Effects of a Community Driven Reconstruction Program in Eastern Democratic REPUBLIC of Congo.

IPA. (2013). Final impact evaluation of the Saving for Change program in Mali.

Ismayilova, L., Karimli, L., Gaveras, E., Tô-Camier, A., Sanson, J., Chaffin, J., and Nanema, R. (2018). An integrated approach to increasing women’s empowerment status and reducing domestic violence: Results of a cluster-randomized controlled trial in a West African country. Psychology of violence, 8(4), 448.

Jewkes, R., Corboz, J., and Gibbs, A. (2018). Trauma exposure and IPV experienced by Afghan women: analysis of the baseline of a randomised controlled trial. PLoS one, 13(10), e0201974.

Karimli, L., Bose, B., and Kagotho, N. (2020). Integrated Graduation Program and its Effect on Women and Household Economic Well-being: Findings from a Randomised Controlled Trial in Burkina Faso. The Journal of Development Studies, 56(7), 1277-1294.

Kohli, A., Perrin, N. A., Mpanano, R. M., Mullany, L. C., Murhula, C. M., Binkurhorhwa, A. K., ... and Glass, N. (2014). Risk for family rejection and associated mental health outcomes among conflict-affected adult women living in rural eastern Democratic Republic of the Congo. Health care for women international, 35(7-9), 789-807.

Komorowska, D. (2016). Citizen Voice in Afghanistan: Evaluation of National Solidarity Programme III.

Kumar, N., Nguyen, P. H., Harris, J., Harvey, D., Rawat, R., and Ruel, M. T. (2018). What it takes: evidence from a nutrition-and gender-sensitive agriculture intervention in rural Zambia. Journal of Development Effectiveness, 10(3), 341-372.

Kyegombe, N., Starmann, E., Devries, K. M., Michau, L., Nakuti, J., Musuya, T., ... and Heise, L. (2014). ‘SASA! is the medicine that treats violence’. Qualitative findings on how a community mobilisation intervention to prevent violence against women created change in Kampala, Uganda. Global health action, 7(1), 25082.

Laudati, A., Mvukiyehe, E., and van der Windt, P. (2018). Participatory Development in Fragile and Conflict-Affected Contexts: An Impact Evaluation of the Tuungane 1 Program in the Democratic Republic of the Congo.

Lecoutere, E., and Wuyts, E. (2020). Confronting the Wall of Patriarchy: Does Participatory Intrahousehold Decision Making Empower Women in Agricultural Households?. The Journal of Development Studies, 1-24.

Leight, J., Deyessa, N., and Sharma, V. (2021). Cost-effectiveness analysis of an intimate partner violence prevention intervention targeting men, women and couples in rural Ethiopia: evidence from the Unite for a Better Life randomised controlled trial. BMJ open, 11(3), e042365.

Leight, J., Deyessa, N., Verani, F., Tewolde, S., and Sharma, V. (2021). Community-Level spillover effects of an intervention to prevent intimate partner violence and HIV transmission in rural Ethiopia. BMJ global health, 6(1), e004075.

Mercy Corps (2015). Improving child and maternal health: why adolescent girl programming matters post intervention: Evidence from Niger.

Messersmith, L. J., Halim, N., Kawemama, P., Steven, E., and Reich, N. (2017). A mixed methods study to test the preliminary effect of World Education’s Together to End Violence Against Women (TEVAW), a program to address intimate partner violence in northern Tanzania: Baseline report.

Mogford, E., Irby, C. A., and Das, A. (2015). Changing Men to Change Gender: Combatting Hegemonic Masculinity through Antiviolence Activism in Northern India. International Journal of Sociology of the Family, 71-93.

Nair, S. (2012). ICTs and Economic Empowerment of Women: Evaluation of SEWA's ICT Activities.

Olney, D. K., Behrman, J. A., Iruhiriye, E., van den Bold, M., and Pedehombga, A. (2015). Helen Keller International’s enhanced homestead food production program in Burkina Faso: Results from a process evaluation.

Pierotti, R. S., Lake, M., and Lewis, C. (2018). Equality on his terms: Doing and undoing gender through men’s discussion groups. Gender and Society, 32(4), 540-562.

Raising Voices, LSHTM and CEDOVIP (2015). Is Violence Against Women Preventable? Findings from the SASA! Study summarized for general audiences. Kampala, Uganda: Raising Voices.

Ringler, K. (2009). A Review of the Ishraq Program's Quasi-Experimental Impact Evaluation (Master’s thesis, University of Minnesota, Minneapolis, United States). Retrieved at: https://conservancy.umn.edu/handle/11299/50227.

Saeed, M. K., and Hayat, M. A. (2020). The Impact of Social Cash Transfers on Poverty in Pakistan-A Case Study of Benazir Income Support Programme.

Sarkar, R. K., and Karim, K. M. (2018). Women's Participation in the Development Program and Household Decision Making Status among the Targeting Ultra Poor (TUP) in Rural Bangladesh. Antrocom: Online Journal of Anthropology, 14(1).

Seidenfeld, D., Handa, S., and Tembo, G. (2013). Social cash transfer Scheme: 24-month impact report for the child grant programme. American Institutes for Research. Retrieved at: https://assets.publishing.service.gov.uk/government/uploads/system/uploads/attachment_data/file/304213/Zambia-Child-Grant-Prog-24-Month-Impact-Report1.pdf.

Sharma, V., Leight, J., Verani, F., Tewolde, S., and Deyessa, N. (2020). Effectiveness of a culturally appropriate intervention to prevent intimate partner violence and HIV transmission among men, women, and couples in rural Ethiopia: Findings from a cluster-randomized controlled trial. PLoS medicine, 17(8), e1003274.

Stangl, A., Farley, K., Sievwright, K., Brady, L., and Fritz, K. (2015). Enhancing Women's Entrepreneurship in Kenya: Initial Qualitative Assessment of the ILO's GET Ahead Business Training Programme. ILO.

Starmann, E., Collumbien, M., Kyegombe, N., Devries, K., Michau, L., Musuya, T., ... and Heise, L. (2017). Exploring couples’ processes of change in the context of SASA!, a violence against women and HIV prevention intervention in Uganda. Prevention science, 18(2), 233-244.

Stern, E., and Niyibizi, L. L. (2018). Shifting perceptions of consequences of IPV among beneficiaries of Indashyikirwa: an IPV prevention program in Rwanda. Journal of interpersonal violence, 33(11), 1778-1804.

Stern, E., and Nyiratunga, R. (2017). A process review of the Indashyikirwa couples curriculum to prevent intimate partner violence and support healthy, equitable relationships in Rwanda. Social sciences, 6(2), 63.

Stern, E., Heise, L., and Cislaghi, B. (2020). Lessons learnt from engaging opinion leaders to address intimate partner violence in Rwanda. Development in Practice, 1-13.

Stern, E., Heise, L., Dunkle, K., and Chatterji, S. (2020). How the Indashyikirwa Intimate Partner Violence Prevention Programme in Rwanda Influenced Parenting and Violence against Children. Journal of Family Violence, 1-12.

Subah-Belleh Associates (2010). Economic Empowerment of Adolescent Girls and Young Women Project (EPAG) Exit Poll Report.

Tanner, S., and O’Connor, M. (2017). A safe place to shine: creating opportunities and raising voices of adolescent girls in humanitarian settings. New York: International Rescue Committee.

Vaillant, J., Koussoubé, E., Roth, D., Pierotti, R., Hossain, M., and Falb, K. L. (2020). Engaging men to transform inequitable gender attitudes and prevent intimate partner violence: a cluster randomised controlled trial in North and South Kivu, Democratic Republic of Congo. BMJ global health, 5(5), e002223.

van den Bold, M., Bliznashka, L., Ramani, G., Olney, D., Quisumbing, A., Pedehombga, A., and Ouedraogo, M. (2021). Nutrition‐sensitive agriculture programme impacts on time use and associations with nutrition outcomes. Maternal and Child Nutrition, 17(2), e13104.

Van den Bold, M., Pedehombga, A., Ouedraogo, M., Quisumbing, A. R., and Olney, D. (2013). Can integrated agriculture-nutrition programs change gender norms on land and asset ownership? Evidence from Burkina Faso.

Vigneri, M., and Lombardini, S. (2017). Women's Empowerment in Mali: Impact evaluation of the educational project:'Girls CAN-Promoting Secondary Education in West Africa'.

Wood, S. N. (2020). “He Tells You Your Work Is to Give Birth”: Reproductive Coercion and Covert Use of Contraception Among Female Intimate Partner Violence Survivors in Nairobi, Kenya (Doctoral dissertation, Johns Hopkins University).

Zaki, E. M. (2018). Cash transfers and state-citizen relation in Egypt: Takaful and Karama in a development context (Master’s thesis, American University in Cairo, Cairo, Egypt). Retrieved from: http://dar.aucegypt.edu/bitstream/handle/10526/5277/Cash%20Transfers%20and%20State%20citizen%20relation%20in%20Egypt.pdf?sequence=1

## Ongoing studies

A Cluster Randomised Controlled Trial To Evaluate The Impact Of Micronance And Participatory Gender Training On Intimate Partner Violence. (2019). RIDIE.<http://ridie.org>

Austrian, K., Muthengi, E., Mumah, J., Soler-Hampejsek, E., Kabiru, C. W., Abuya, B., and Maluccio, J. A. (2016). The Adolescent Girls Initiative-kenya (AGI-K): Study Protocol. BMC Public Health, 16(1), 210.<https://doi.org/10.1186/s12889-016-2888-1>

Bapolisi, W. A., Ferrari, G., Blampain, C., Makelele, J., Kono-Tange, L., Bisimwa, G., and Merten, S. (2020). Impact Of A Complex Gender-transformative Intervention On Maternal And Child Health Outcomes In The Eastern Democratic Republic Of Congo: Protocol Of A Longitudinal Parallel Mixed-methods Study. BMC Public Health, 20(1), 51.<https://doi.org/10.1186/s12889-019-8084-3>

Cherchi, L., Goldstein, M., Habyarimana, J., Montalvao, J., O’Sullivan, M., Udry, C., and Gruver, A. (2019). Empowering Women Through Equal Land Rights: Experimental Evidence From Rural Uganda (Policy Brief No. 33). Gender Innovation Lab.<https://doi.org/10.1596/31513>

Clark, C. J., Spencer, R. A., Shrestha, B., Ferguson, G., Oakes, J. M., and Gupta, J. (2017). Evaluating A Multicomponent Social Behaviour Change Communication Strategy To Reduce Intimate Partner Violence Among Married Couples: Study Protocol For A Cluster Randomized Trial In Nepal. BMC Public Health, 17(1), 75.<https://doi.org/10.1186/s12889-016-3909-9>

Constantine Manda, Donald Green, Dylan Groves, Rachel Jones, Bardia Rahmani, and Beatrice Montano. (2018). Mass Media Experiments to Reduce Violence Against Women in Tanzania. Innovations for Poverty Action.

Daruwalla, N., Machchhar, U., Pantvaidya, S., D’Souza, V., Gram, L., Copas, A., and Osrin, D. (2019). Community Interventions To Prevent Violence Against Women And Girls In Informal Settlements In Mumbai: The SNEHA-TARA Pragmatic Cluster Randomised Controlled Trial. Trials, 20(1), 743.<https://doi.org/10.1186/s13063-019-3817-2>

Empowering Adolescent Girls In The Sahel: Evidence From A Multi-country RCT Of The Sahel Women Empowerment And Demographic Dividend Project. (2020). RIDIE.<http://ridie.org>

Enhancing The Economic Impact Of Rural Roads On Women In Nicaragua. (2020). RIDIE.<http://ridie.org>

Falb, K. L., Tanner, S., Ward, L., Erksine, D., Noble, E., Assazenew, A., Bakomere, T., Graybill, E., Lowry, C., Mallinga, P., Neiman, A., Poulton, C., Robinette, K., Sommer, M., and Stark, L. (2016). Creating Opportunities Through Mentorship, Parental Involvement, And Safe Spaces (COMPASS) Program: Multi-country Study Protocol To Protect Girls From Violence In Humanitarian Settings. BMC Public Health, 16(1), 231.<https://doi.org/10.1186/s12889-016-2894-3>

Gibbs, A., Corboz, J., Shafiq, M., Marofi, F., Mecagni, A., Mann, C., Karim, F., Chirwa, E., Maxwell-Jones, C., and Jewkes, R. (2018a). An Individually Randomized Controlled Trial To Determine The Effectiveness Of The Women For Women International Programme In Reducing Intimate Partner Violence And Strengthening Livelihoods Amongst Women In Afghanistan: Trial Design, Methods And Baseline Findings. BMC Public Health, 18(1), 164.<https://doi.org/10.1186/s12889-018-5029-1>

Gibbs, A., Corboz, J., Shafiq, M., Marofi, F., Mecagni, A., Mann, C., Karim, F., Chirwa, E., Maxwell-Jones, C., and Jewkes, R. (2018b). An Individually Randomized Controlled Trial To Determine The Effectiveness Of The Women For Women International Programme In Reducing Intimate Partner Violence And Strengthening Livelihoods Amongst Women In Afghanistan: Trial Design, Methods And Baseline Findings. BMC Public Health, 18(1), 164.<https://doi.org/10.1186/s12889-018-5029-1>

Girl Empower: Studying The Impact Of Mentorship, Asset Building, Caregiver Discussion Groups, And Cash Transfers On Reducing Girl’s Vulnerability To Sexual Exploitation And Abuse. (2017). RIDIE.<http://ridie.org>

Glass, N., Perrin, N., Clough, A., Desgroppes, A., Kaburu, F. N., Melton, J., Rink, A., Read-Hamilton, S., and Marsh, M. (2018a). Evaluating The Communities Care Program: Best Practice For Rigorous Research To Evaluate Gender Based Violence Prevention And Response Programs In Humanitarian Settings. Conflict and Health, 12(1), 5.<https://doi.org/10.1186/s13031-018-0138-0>

Glass, N., Perrin, N., Clough, A., Desgroppes, A., Kaburu, F. N., Melton, J., Rink, A., Read-Hamilton, S., and Marsh, M. (2018b). Evaluating The Communities Care Program: Best Practice For Rigorous Research To Evaluate Gender Based Violence Prevention And Response Programs In Humanitarian Settings. Conflict and Health, 12(1), 5.<https://doi.org/10.1186/s13031-018-0138-0>

Glass, N., Perrin, N., Marsh, M., Clough, A., Desgroppes, A., Kaburu, F., Ross, B., and Read-Hamilton, S. (2019). Effectiveness Of The Communities Care Programme On Change In Social Norms Associated With Gender-based Violence (GBV) With Residents In Intervention Compared With Control Districts In Mogadishu, Somalia. BMJ Open, 9(3), e023819.<https://doi.org/10.1136/bmjopen-2018-023819>

Hallman, K., Kelvin, E., Ozler, B., Seban, J., Kuhlik, E., Alton, C., Kamara, J., and Goodman, S. (2016). Combining Mentoring Programs With Cash Transfers For Adolescent Girls In Liberia: Baseline Report (No. 7797; Policy Research Working Paper). World Bank, Washington, DC.<https://doi.org/10.1596/1813-9450-7797>

Hartmann, M. A., Datta, S., Banay, R. F., Caetano, V., Floreak, R., Appaiah, P., Sreevasthsa, A., Thomas, S., Selvam, S., Barnette, Q., and Srinivasan, K. (2018). Designing A Pilot Study Protocol To Test A Male Alcohol Use And Intimate Partner Violence Reduction Intervention In India: Beautiful Home. Frontiers in Public Health, 6, 218.<https://doi.org/10.3389/fpubh.2018.00218>

Harvey, S., Lees, S., Mshana, G., Pilger, D., Hansen, C., Kapiga, S., and Watts, C. (2018). A Cluster Randomized Controlled Trial To Assess The Impact On Intimate Partner Violence Of A 10-session Participatory Gender Training Curriculum Delivered To Women Taking Part In A Group-based Microfinance Loan Scheme In Tanzania (MAISHA CRT01): Study Protocol. BMC Women’s Health, 18(1), 55.<https://doi.org/10.1186/s12905-018-0546-8>

Hewett, P. C., Austrian, K., Soler-Hampejsek, E., Behrman, J. R., Bozzani, F., and Jackson-Hachonda, N. A. (2017). Cluster Randomized Evaluation Of Adolescent Girls Empowerment Programme (AGEP): Study Protocol. BMC Public Health, 17(1), 386.<https://doi.org/10.1186/s12889-017-4280-1>

Impact Evaluation Of Care’s Inspiring Married Adolescent Girls To Imagine New Empowered Futures (IMAGINE) Project. (2019). [RIDIE].<http://ridie.org>

Jeannie Annan, Christopher Boyer, and Günther Fink. (n.d.). Modern Man Challenge: Evaluating a Men-Focused Intervention to Prevent Intimate Partner Violence in Liberia. Innovations for Poverty Action.

Ketema, T., Bastian, G., Gras, O., Abro, Z., Manchester, K., and Carranza, E. (2015). Ethiopia Women Agribusiness Leaders Network Impact Evaluation: Baseline Survey Report. World Bank.<https://doi.org/10.1596/30314>

Khobarkar, V., Ingole, D. N., and Nage, G. V. (2016). Performance Of Self-help Groups In Micro Finance. Economic Affairs, 61(4), 609.<https://doi.org/10.5958/0976-4666.2016.00075.9>

Krishnan, S., Subbiah, K., Chandra, P., and Srinivasan, K. (2012). Minimizing Risks And Monitoring Safety Of An Antenatal Care Intervention To Mitigate Domestic Violence Among Young Indian Women: The Dil Mil Trial. BMC Public Health, 12(1), 943.<https://doi.org/10.1186/1471-2458-12-943>

Lerva, B. (n.d.). Power Dynamics In Traditional Households (AEARCTR-0001806) [Data set]. American Economic Association.<https://doi.org/10.1257/rct.1806-1.0>

Leveraging Youth Clubs For Women’s Empowerment In Niger | The Abdul Latif Jameel Poverty Action Lab. (2018). The Abdul Latif Jameel Poverty Action Lab (J-PAL).<https://www.povertyactionlab.org/evaluation/leveraging-youth-clubs-womens-empowerment-niger>

Mahmood, S. (2011). Microfinance And Women Entrepreneurs In Pakistan. International Journal of Gender and Entrepreneurship, 3(3), 265–274.<https://doi.org/10.1108/17566261111169340>

McKenzie, D., and Weber, M. (n.d.). The results of a pilot financial literacy and business planning training program for women in Uganda (No. 8; Finance and PSD Impact, p. 2). World Bank.

Patson Gondwe. (2020). Enabling Sustainable Graduation out of Poverty for the Extreme Poor in Malawi Umodzi Cohort 2: Successes and Learning Points. Concern.

Peter Carroll, Flora Myamba, Daniel Nielson, Joseph Price, Phillip Roessler, and Wayne Sandholtz. (n.d.). Fast Cash: The Impact of Up-Front Incentives on Young Women’s Savings in Tanzania. Innovations for Poverty Action.

Saeed Ali, T., Karmaliani, R., Mcfarlane, J., Khuwaja, H. M. A., Somani, Y., Chirwa, E. D., and Jewkes, R. (2017). Attitude Towards Gender Roles And Violence Against Women And Girls (VAWG): Baseline Findings From An RCT Of 1752 Youths In Pakistan. Global Health Action, 10(1), 1342454.<https://doi.org/10.1080/16549716.2017.1342454>

Sahel Women’s Empowerment And Demographic Dividend (SWEDD): Empowering Adolescent Girls In Mali. (2020). RIDIE.<http://ridie.org>

Sahel Women’s Empowerment And Demographic Dividend (SWEDD): Empowering Adolescent Girls Through Safe Spaces And Accompanying Measures In Burkina Faso. (2020). RIDIE.<http://ridie.org>

Sahel Women’s Empowerment And Demographic Dividend (SWEDD): Empowering Adolescent Girls Through Safe Spaces And Accompanying Measures In Cote D’ivoire. (2020). RIDIE.<http://ridie.org>

Sarnquist, C., Kang, J. L., Amuyunzu-Nyamongo, M., Oguda, G., Otieno, D., Mboya, B., Omondi, N., Kipkirui, D., and Baiocchi, M. (2019). A Protocol For A Cluster-randomized Controlled Trial Testing An Empowerment Intervention To Prevent Sexual Assault In Upper Primary School Adolescents In The Informal Settlements Of Nairobi, Kenya. BMC Public Health, 19(1), 834.<https://doi.org/10.1186/s12889-019-7154-x>

Sister Of Success Mentoring And Girls Groups In Liberia. (2014). RIDIE.<http://ridie.org>

Vaillant, J. (n.d.). Preventing Intimate-partner Violence: Impact Evaluation Of Engaging Men Through Accountable Practice In Eastern DRC (AEARCTR-0001218) [Data set]. American Economic Association.<https://doi.org/10.1257/rct.1218-4.0>

Women Agribusiness Leaders Network (WALN). (2018). RIDIE.<http://ridie.org>

Zupancic, Mateja. (2017). Knowledge, Attitudes And Practices On Violence And Harmful Practices Against Children In Afghanistan A Baseline Study. Save the Children.

## Other references

### Protocol

Aall, P., & Crocker, C. A. (2019). Building Resilience and Social Cohesion in Conflict. Global Policy, 10(S2), 68–75. <https://doi.org/10.1111/1758-5899.12681>

Abt, T., & Winship, C. (2016). What works in reducing community violence: A meta-review and field study for the northern triangle (p. 53). USAID. <https://www.usaid.gov/sites/default/files/USAID-2016-What-Works-in-Reducing-Community-Violence-Final-Report.pdf>

Agence Francaise de Developpement. (n.d.). Juillet 2020: L’AFD soutient 27 nouveaux projets d’ONG françaises. Retrieved November 27, 2020, from <https://www.afd.fr/fr/actualites/juillet-2020-l-afd-soutient-27-nouveaux-projets-ong-francaises>

Balarin, M., chinen, marjorie, Hoop, T., sennett, josh, & Alcázar, L. (2017). Vocational and business training to improve women’s labour market outcomes in low- and middle-income countries: A systematic review. Campbell Systematic Reviews, 13. <https://doi.org/10.4073/csr.2017.16>

Bangpan, M., Felix, L., Chiumento, A., & Dickson, K. (2016). The impact of mental health and psychosocial support programmes for populations affected by humanitarian emergencies: A systematic review protocol. Oxfam. <https://doi.org/10.21201/2016.605150>

Barnett, M., Kim, H., O’Donnell, M., & Sitea, L. (2007). Peacebuilding: What Is in a Name? Global Governance: A Review of Multilateralism and International Organizations, 13(1), 35–58. <https://doi.org/10.1163/19426720-01301004>

Begg, C. B., & Mazumdar, M. (1994). Operating characteristics of a rank correlation test for publication bias. Biometrics, 50(4), 1088–1101.

Berghof Foundation. (2019). Human security. Berghof Foundation. <https://berghof-foundation.org/themes/human-security>

Bigio, J., & Vogelsteing, R. (2016). How women’s participation in conflict prevention and resolution advances US Interests (p. 49). Council for Foreign Relations. <https://cdn.cfr.org/sites/default/files/pdf/2016/10/Discussion_Paper_Bigio_Vogelstein_Women%20in%20CPR_OR.pdf>

Björkdahl, A., & Höglund, K. (2013). Precarious peacebuilding: Friction in global–local encounters. Peacebuilding, 1(3), 289–299. <https://doi.org/10.1080/21647259.2013.813170>

Blair, R., Grossman, G., & Wilke, A. (2020). Community Policing in Uganda. <https://osf.io/df3jk>

Blattman, C., Green, D., Columbia University, Ortega, D., Development Bank of Latin America (CAF), Tobón, S., & University of Chicago and Innovations for Poverty Action. (2018). Hotspot interventions at scale: The effects of policing and city services on crime in Bogotá, Colombia (2018th ed.). International Initiative for Impact Evaluation (3ie). <https://doi.org/10.23846/DPW1IE88>

Blattman, C., Hartman, A., & Blair, R. (2011). Can we Teach Peace and Conflict Resolution?: Results from a randomized evaluation of the Community Empowerment Program (CEP) in Liberia: A Program to Build Peace, Human Rights, and Civic Participation. 2, 38.

Blattman, C., & Tobon, S. (n.d.). Contesting Criminal Gang Governance in Medellin: The Impacts of Intensive Municipal Governance and Community Organization on Gang Control and Governing of Neighborhoods [Data set]. American Economic Association. <https://doi.org/10.1257/rct.2622-1.0>

Borenstein, M., Hedges, L. V., Higgins, J. P. T., & Rothstein, H. R. (2009). Introduction to Meta-Analysis. John Wiley and Sons. <https://doi.org/10.1002/9780470743386>

Bourey, C., Bernstein, E., & Stephenson, R. (2015). Systematic review of structural interventions for intimate partner violence in low- and middle-income countries: Organizing evidence for prevention. BMC Public Health, 15. <https://doi.org/10.1186/s12889-015-2460-4>

Bouta, T., Frerks, G., & Bannon, I. (2004). Gender, Conflict, and Development. The World Bank. <https://doi.org/10.1596/0-8213-5968-1>

Brody, C., Hoop, T. de, Vojtkova, M., Warnock, R., Dunbar, M., Murthy, P., & Dworkin, S. L. (2017). Can self-help group programs improve women’s empowerment? A systematic review. Journal of Development Effectiveness, 9(1), 15–40. <https://doi.org/10.1080/19439342.2016.1206607>

Bronfenbrenner, U. (1979). The ecology of human development: Experiments by nature and design. Harvard University Press.

Buvinic, M., Das Gupta, M., Casabonne, U., & Verwimp, P. (2013). Violent Conflict and Gender Inequality: An Overview. The World Bank Research Observer, 28(1), 110–138. <https://doi.org/10.1093/wbro/lks011>

Buvinic, M., Gupta, M. D., Casabonne, U., & Verwimp, P. (2013). Violent Conflict and Gender Inequality: An Overview. 37.

Cameron, D., Brown, A., Mishra, A., Picon, M., Esper, H., Calvo, F., & Peterson, K. (2015). Evidence for peacebuilding: An evidence gap map | 3ie (Evidence Gap Map Report 1; p. 50). International Initiative for Impact Evaluation (3ie). <https://www.3ieimpact.org/evidence-hub/publications/evidence-gap-maps/evidence-peacebuilding-evidence-gap-map>

Campbell Collaboration. (2014). Campbell Collaboration Systematic Reviews: Policies and Guidelines. The Campbell Collaboration. <https://doi.org/10.4073/cpg.2016.1>

Caprioli, M. (2000). Gendered Conflict. Journal of Peace Research, 37(1), 51–68. <https://doi.org/10.1177/0022343300037001003>

CASP-Qualitative-Checklist-2018.pdf. (n.d.). Retrieved June 21, 2021, from <https://casp-uk.net/wp-content/uploads/2018/01/CASP-Qualitative-Checklist-2018.pdf>

Chant, S., & Sweetman, C. (2012). Fixing women or fixing the world? ‘Smart economics’, efficiency approaches, and gender equality in development. Gender & Development, 20(3), 517–529. <https://doi.org/10.1080/13552074.2012.731812>

Cheldelin, S., & Mutisi, M. (2016). Deconstructing women, peace and security: A critical review of approaches to gender and empowerment. <https://doi.org/10/9770>

Chen, M., Vanek, J., & Heintz, J. (2006). Informality, Gender and Poverty: A Global Picture. Economic and Political Weekly, 41, 2131–2139. <https://doi.org/10.2307/4418269>

Cochrane Collaboration. (n.d.). Cochrane Handbook for Systematic Reviews of Interventions. Retrieved November 27, 2020, from <https://handbook-5-1.cochrane.org/>

Coffey, C., International Initiative for Impact Evaluation (3ie), Gallagher, E., International Initiative for Impact Evaluation (3ie), Fenton Villar, P., International Initiative for Impact Evaluation (3ie), Stevenson, J., International Initiative for Impact Evaluation (3ie), Tsoli, S., International Initiative for Impact Evaluation (3ie), Dhanasekar, S., International Initiative for Impact Evaluation (3ie), Eyers, J., & Independent consultant. (2017). State-society relations in low- and middle-income countries: An evidence gap map (2017th ed.). International Initiative for Impact Evaluation (3ie). <https://doi.org/10.23846/EGM007>

Collazos, D., Garcia, E., Mejia, D., Ortega, D., & Tobon, S. (2019). Hot Spots Policing in a High Crime Environment: An Experimental Evaluation in Medellín (SSRN Scholarly Paper ID 3316968). Social Science Research Network. <https://doi.org/10.2139/ssrn.3316968>

Committee on the Elimination of Discrimination against Women. (2013). General recommendation No. 30 on women in conflict prevention, conflict and post conflict situations [Recommendation]. <https://www.ohchr.org/Documents/HRBodies/CEDAW/GComments/CEDAW.C.CG.30.pdf>

COMPare. (n.d.). Tracking switched outcomes in clinical trials. COMPare. Retrieved January 8, 2021, from <http://compare-trials.org>

Cooper, H., Hedges, L. V., & Valentine, J. C. (2009). The handbook of research synthesis and meta-analysis 2nd edition. The Hand. of Res. Synthesis and Meta-Analysis, 2nd Ed., 1–615.

Cornwall, A. (2000). Missing Men? Reflections on Men, Masculinities and Gender in GAD. IDS Bulletin, 31(2), 18–27. <https://doi.org/10.1111/j.1759-5436.2000.mp31002003.x>

Cornwall, A. (2016). Women’s Empowerment: What Works? Journal of International Development, 28(3), 342–359. <https://doi.org/10.1002/jid.3210>

Council on Foreign Relations. (n.d.). Including Women at the Peace Table Produces Better Outcomes. Council on Foreign Relations. Retrieved November 26, 2020, from <https://www.cfr.org/womens-participation-in-peace-processes>

Council on Foreign Relations. (2020). Including Women at the Peace Table Produces Better Outcomes. Council on Foreign Relations. <https://www.cfr.org/womens-participation-in-peace-processes>

Delkhosh, M., Ardalan, A., Rahimiforoushani, A., Keshtkar, A., Amiri Farahani, L., & Merghati Khoei, E. (2017). Interventions for Prevention of Intimate Partner Violence Against Women in Humanitarian Settings: A Protocol for a Systematic Review. PLoS Currents, 9. <https://doi.org/10.1371/currents.dis.f41d45fbdca13babe4ae5be0f9732e75>

Department for International Development. (n.d.-a). DevTracker Project GB-COH-1858644-114433—AAWAZ. Retrieved November 27, 2020, from <https://devtracker.fcdo.gov.uk/projects/GB-COH-1858644-114433>

Department for International Development. (n.d.-b). DevTracker Project GB-GOV-13-FUND--GCRF-AH_S004025_1—GCRF. Retrieved November 27, 2020, from <https://devtracker.fcdo.gov.uk/projects/GB-GOV-13-FUND--GCRF-AH_S004025_1>

Department for International Development. (n.d.-c). DFID Strategic Vision for Gender Equality: A call to action for her potential. 22.

Department for International Development. (2010). Building Peaceful States and Societies: A DFID Practice Paper (p. 60). DFID. <http://www.gsdrc.org/docs/open/con75.pdf>

DerSimonian, R., & Laird, N. (2015). Meta-Analysis in Clinical Trials Revisited. Contemporary Clinical Trials, 45(0 0), 139–145. <https://doi.org/10.1016/j.cct.2015.09.002>

Desmidt, S., & Davis, L. (2019). Rhetoric and real progress on the Women, Peace and Security agenda in Africa.

Dixon-Woods, M., Agarwal, S., Jones, D., Young, B., & Sutton, A. (2005). Synthesising qualitative and quantitative evidence: A review of possible methods. Journal of Health Services Research & Policy, 10(1), 45–53. <https://doi.org/10.1177/135581960501000110>

Doherty, J., Sonnenfeld, A., Glandon, D., Kozakiewicz, T., Snilstveit, B., & Sabet, D. (2020). Protocol: The effects of rule of law interventions on justice outcomes: An evidence gap map. 85.

Doocy, S., & Tappis, H. (2015). PROTOCOL: The effectiveness and efficiency of cash-based approaches in emergencies: A Systematic Review. Campbell Systematic Reviews, 11(1), 1–45. <https://doi.org/10.1002/CL2.150>

Doss, C. (2013). Intrahousehold Bargaining and Resource Allocation in Developing Countries. The World Bank Research Observer, 28(1), 52–78.

Dudouet, V. (2017). From Power Mediation to Dialogue Support? Assessing the European Union’s Capabilities for Multi-Track Diplomacy.

Duvendack, M., Hombrados, J. G., Palmer-Jones, R., & Waddington, H. (2012). Assessing ‘what works’ in international development: Meta-analysis for sophisticated dummies. Journal of Development Effectiveness, 4(3), 456–471. <https://doi.org/10.1080/19439342.2012.710642>

Egger, M., Smith, G. D., Schneider, M., & Minder, C. (1997). Bias in meta-analysis detected by a simple, graphical test. BMJ, 315(7109), 629–634. <https://doi.org/10.1136/bmj.315.7109.629>

Ellis, P. D. (2010). The Essential Guide to Effect Sizes: Statistical Power, Meta-Analysis, and the Interpretation of Research Results. Cambridge University Press. <https://doi.org/10.1017/CBO9780511761676>

ESCAP. (1999). Women’s Empowerment in the Context of Human Security. <https://www.un.org/womenwatch/ianwge/collaboration/finalcomm1999.htm>

Faust, J., Grävingholt, J., & Ziaja, S. (2013). Foreign Aid and the Fragile Consensus on State Fragility. <https://doi.org/10.2139/ssrn.1489936>

Fisher, Z., & Tipton, E. (2015). robumeta: An R-package for robust variance estimation in meta-analysis. ArXiv:1503.02220 [Stat]. <http://arxiv.org/abs/1503.02220>

Galtung, J. (1996). Peace by peaceful means: Peace and conflict, development and civilization (pp. viii, 280). Sage Publications, Inc.

Gibbs, A., Willan, S., Misselhorn, A., & Mangoma, J. (2012). Combined structural interventions for gender equality and livelihood security: A critical review of the evidence from southern and eastern Africa and the implications for young people. Journal of the International AIDS Society, 15 Suppl 1, 1–10. <https://doi.org/10.7448/IAS.15.3.17362>

Gillespie, L.-A., Gupta, P., Landis, D., Shannon, H., & Williamson, K. (n.d.). The impact of protection interventions on unaccompanied and separated children: A systematic review. 124.

Global Affairs Canada. (2017). Canada’s feminist international assistance policy, #HerVoiceHerChoice (p. 92). Global Affairs Canada. <https://www.international.gc.ca/world-monde/assets/pdfs/iap2-eng.pdf?_ga=2.237985280.167804930.1603192729-143277333.1603192729>

Global Affairs Ireland. (n.d.). A Simple Guide to Ireland’s NAP on Women, Peace and Security (p. 13). Retrieved November 27, 2020, from <https://www.dfa.ie/media/dfa/ourrolepolicies/peaceandsecurity/WPS-Simple-Guide.pdf>

Goetz, A. M. (2008). Who answers to women? Gender & accountability. United Nations Development Fund for Women.

Guyatt, G. H., Oxman, A. D., Vist, G. E., Kunz, R., Falck-Ytter, Y., Alonso-Coello, P., Schünemann, H. J., & GRADE Working Group. (2008). GRADE: An emerging consensus on rating quality of evidence and strength of recommendations. BMJ (Clinical Research Ed.), 336(7650), 924–926. <https://doi.org/10.1136/bmj.39489.470347.AD>

Hamilton, C., Naam, N., & Shepherd, L. J. (2020). Twenty Years of Women, Peace and Security National Action Plans: Analysis and Lessons Learned. 36.

Hammerstrøm, K., Wade, A., & Jørgensen, A. M. K. (2010a). Searching for studies: A guide to information retrieval for Campbell Systematic Reviews. Campbell Systematic Reviews, 1–75.

Hammerstrøm, K., Wade, A., & Jørgensen, A. M. K. (2010b). Searching for studies: A guide to information retrieval for Campbell Systematic Reviews. Campbell Systematic Reviews, 1–75.

Handanagic, S., Barbaric, J., Anglemyer, A., & Bozicevic, I. (2016). Community mobilization and empowerment interventions for preventing HIV in low and middle income countries: Protocols. In The Cochrane Library (Vol. 2016). <https://doi.org/10.1002/14651858.CD011186.pub2>

Hedges, L. V., Tipton, E., & Johnson, M. C. (2010). Robust variance estimation in meta-regression with dependent effect size estimates. Research Synthesis Methods, 1(1), 39–65. <https://doi.org/10.1002/jrsm.5>

Hedström, J., Senarathna, T., & International Institute for Democracy and Electoral Assistance (Eds.). (2015). Women in conflict and peace. International IDEA.

Higgins, J. P., Sterne, J. A., Savovic, J., Page, M. J., Hróbjartsson, A., Boutron, I., Reeves, B., & Eldridge, S. (2016). A revised tool for assessing risk of bias in randomized trials. Cochrane Database of Systematic Reviews, 10(Suppl 1), 29–31.

Higgins, J. P. T., & Thomas, J. (2020). Cochrane Handbook for Systematic Reviews of Interventions. [/handbook/current](https://doi.org/handbook/current)

Higgins, J. P. T., & Thompson, S. G. (2002). Quantifying heterogeneity in a meta-analysis. Statistics in Medicine, 21(11), 1539–1558. <https://doi.org/10.1002/sim.1186>

Hultcrantz, M., Rind, D., Akl, E. A., Treweek, S., Mustafa, R. A., Iorio, A., Alper, B. S., Meerpohl, J. J., Murad, M. H., Ansari, M. T., Katikireddi, S. V., Östlund, P., Tranæus, S., Christensen, R., Gartlehner, G., Brozek, J., Izcovich, A., Schünemann, H., & Guyatt, G. (2017). The GRADE Working Group clarifies the construct of certainty of evidence. Journal of Clinical Epidemiology, 87, 4–13. <https://doi.org/10.1016/j.jclinepi.2017.05.006>

Institute for Economics and Peace. (2020). Global Peace Index 2020: Measuring Peace in a Complex World. Institute for Economics & Peace. <https://visionofhumanity.org/wp-content/uploads/2020/10/GPI_2020_web.pdf>

Jong, S. de, & Kimm, S. (2017). The co-optation of feminisms: A research agenda. International Feminist Journal of Politics, 19(2), 185–200. <https://doi.org/10.1080/14616742.2017.1299582>

Kabeer, N. (1999). Resources, Agency, Achievements: Reflections on the Measurement of Women’s Empowerment. Development and Change, 30(3), 435–464. <https://doi.org/10.1111/1467-7660.00125>

Kabeer, N., & Waddington, H. (2015). Economic impacts of conditional cash transfer programmes: A systematic review and meta-analysis. Journal of Development Effectiveness, 7(3), 290–303. <https://doi.org/10.1080/19439342.2015.1068833>

Keef, S. P., & Roberts, L. A. (2004). The meta-analysis of partial effect sizes. British Journal of Mathematical and Statistical Psychology, 57(1), 97–129. <https://doi.org/10.1348/000711004849303>

Keuleers, P. (2016). Why building peaceful societies is part of the sustainable development agenda. UNDP. <https://www.undp.org/content/undp/en/home/blog/2016/5/18/Why-building-peaceful-societies-is-part-of-the-sustainable-development-agenda.html>

King, E. (2013). A Critical Review of Community-Driven Development Programmes in Conflict-Affected Contexts. [https://www.academia.edu/13841546/A_Critical_Review_of_
Community_Driven_Development_Programmes_in_Conflict_Affected_Contexts](https://www.academia.edu/13841546/A_Critical_Review_of_Community_Driven_Development_Programmes_in_Conflict_Affected_Contexts)

King, E., Samii, C., & Snilstveit, B. (2010). Interventions to promote social cohesion in sub-Saharan Africa. Journal of Development Effectiveness, 2(3), 336–370. <https://doi.org/10.1080/17449057.2010.504552>

Kraft, J., Wilkins, K., Morales, G., Widyono, M., & Middlestadt, S. (2014). An Evidence Review of Gender-Integrated Interventions in Reproductive and Maternal-Child Health. Journal of Health Communication, 19, 122–141. <https://doi.org/10.1080/10810730.2014.918216>

Kugley, S., Wade, A., Thomas, J., Mahood, Q., Jørgensen, A.-M. K., Hammerstrøm, K., & Sathe, N. (2017). Searching for studies: A guide to information retrieval for Campbell systematic reviews. Campbell Systematic Reviews, 13(1), 1–73. <https://doi.org/10.4073/cmg.2016.1>

Langenkamp, D. A. (n.d.). GIZ Gender Strategy. Gender reloaded: Vision needs Attitude – Attitude meets Action. 20.

Lewin, S., Booth, A., Glenton, C., Munthe-Kaas, H., Rashidian, A., Wainwright, M., Bohren, M. A., Tunçalp, Ö., Colvin, C. J., Garside, R., Carlsen, B., Langlois, E. V., & Noyes, J. (2018a). Applying GRADE-CERQual to qualitative evidence synthesis findings: Introduction to the series. Implementation Science, 13(1), 2. <https://doi.org/10.1186/s13012-017-0688-3>

Lewin, S., Booth, A., Glenton, C., Munthe-Kaas, H., Rashidian, A., Wainwright, M., Bohren, M. A., Tunçalp, Ö., Colvin, C. J., Garside, R., Carlsen, B., Langlois, E. V., & Noyes, J. (2018b). Applying GRADE-CERQual to qualitative evidence synthesis findings: Introduction to the series. Implementation Science, 13(1), 2. <https://doi.org/10.1186/s13012-017-0688-3>

Lipsey, M. W., & Wilson, D. B. (2001). Practical meta-analysis. Sage Publications. <http://search.ebscohost.com/login.aspx?direct=true&scope=site&db=nlebk&db=nlabk&AN=63274>

London School of Economics. (2019). LSE-WPS National Action Plans. LSE - Women, Peace Security. <https://www.wpsnaps.org/>

Lynch, U., McGrellis, S., Dutschke, M., Anderson, M., Arnsberger, P., & Macdonald, G. (2013). What is the evidence that the establishment or use of community accountability mechanisms and processes improves inclusive service delivery by governments, donors and NGOs to communities? Social Science Research Unit, Institute of Education, University of London. <https://pure.qub.ac.uk/en/publications/what-is-the-evidence-that-the-establishment-or-use-of-community-a>

Macdonald, G., Higgins, J. P., Ramchandani, P., Valentine, J. C., Bronger, L. P., Klein, P., O’Daniel, R., Pickering, M., Rademaker, B., Richardson, G., & Taylor, M. (2012). Cognitive-Behavioural Interventions for Children Who Have Been Sexually Abused: A Systematic Review. Campbell Systematic Reviews, 8(1), 1–111. <https://doi.org/10.4073/csr.2012.14>

McWilliams, M., & Kilmurray, A. (2015). From the global to the local: Grounding UNSCR 1325 on women, peace and security in post conflict policy making. Women’s Studies International Forum, 51, 128–135. <https://doi.org/10.1016/j.wsif.2014.11.006>

Miles, M. B., & Huberman, A. M. (1994). Qualitative data analysis: An expanded sourcebook.

Ministry of Foreign Affairs. (n.d.). Dutch National Action Plan on Resolution 1325: Taking a stand for women, peace and security. Ministry of Foreign Affairs (Netherland). Retrieved November 27, 2020, from <https://www.peacewomen.org/sites/default/files/dutch_nationalactionplan_december2007.pdf>

Montgomery, P., Movsisyan, A., Grant, S. P., Macdonald, G., & Rehfuess, E. A. (2019). Considerations of complexity in rating certainty of evidence in systematic reviews: A primer on using the GRADE approach in global health. BMJ Global Health, 4(Suppl 1), e000848. <https://doi.org/10.1136/bmjgh-2018-000848>

Moore, L., Chersich, M. F., Steen, R., Reza-Paul, S., Dhana, A., Vuylsteke, B., Lafort, Y., & Scorgie, F. (2014). Community empowerment and involvement of female sex workers in targeted sexual and reproductive health interventions in Africa: A systematic review. Globalization and Health, 10(1), 47. <https://doi.org/10.1186/1744-8603-10-47>

Mundkur, A., & Shepherd, L. (2018, January 23). How (not) to make WPS count. LSE Women, Peace and Security Blog. <https://blogs.lse.ac.uk/wps/2018/01/23/how-not-to-make-wps-count/>

Munro, J. (2000). Gender and Peacebuilding. 26.

Noyes, J., Booth, A., Flemming, K., Harden, A., Harris, J., Garside, R., Hannes, K., Pantoja, T., & Thomas, J. (2020). Cochrane Handbook for Systematic Reviews of Interventions version 6.1 Chapter 21: Qualitative evidence (6.1). Cochrane Collaboration. [/handbook/current/chapter-21](https://doi.org/handbook/current/chapter-21)

O’Driscoll, D. (2017). Women’s participation in peacebuilding and reconciliation in Iraq. <https://opendocs.ids.ac.uk/opendocs/handle/20.500.12413/13437>

OECD. (2013). Gender and Statebuilding in Fragile and Conflict-affected States (p. 92). OECD. <https://read.oecd-ilibrary.org/development/gender-and-statebuilding-in-fragile-and-conflict-affected-states_9789264202061-en>

OECD. (2016a). States of Fragility 2016| OECD iLibrary. <https://www.oecd-ilibrary.org/sites/9789264267213-en/index.html?itemId=/content/publication/9789264267213-en>

OECD. (2016b). Entrepreneurship at a Glance 2016. OECD. <https://doi.org/10.1787/entrepreneur_aag-2016-en>

OECD. (2020). States of Fragility 2020 | OECD iLibrary. [../../../../dcd-2020-185-en/index.html](https://doi.org/../../../../dcd-2020-185-en/index.html)

O’Mara-Eves, A., Thomas, J., McNaught, J., Miwa, M., & Ananiadou, S. (2015a). Using text mining for study identification in systematic reviews: A systematic review of current approaches. Systematic Reviews, 4. <https://doi.org/10.1186/2046-4053-4-5>

O’Mara-Eves, A., Thomas, J., McNaught, J., Miwa, M., & Ananiadou, S. (2015b). Using text mining for study identification in systematic reviews: A systematic review of current approaches. Systematic Reviews, 4(1), 5. <https://doi.org/10.1186/2046-4053-4-5>

O’Reilly, M. (2013). Issue Brief—Women in Conflict Mediation: Why it Matters. International Peace Institute. <https://www.ipinst.org/wp-content/uploads/publications/ipi_e_pub_women_in_conflict_med.pdf>

Pankhurst, D. T. (2000). Women, Gender and Peacebuilding [Working Paper]. <https://bradscholars.brad.ac.uk/handle/10454/939>

Peace Women. (n.d.). Action Plan on Transformative Financing for GEWE.pdf. Retrieved December 14, 2020, from <http://www.peacewomen.org/sites/default/files/Action%20Plan%20on%20Transformative%20Financing%20for%20GEWE.pdf>

Peace Women. (2014, November 14). WPS National-Level Implementation. PeaceWomen. <https://www.peacewomen.org/member-states>

Peace Women. (2016, June 23). Women, Peace and Security Financing. PeaceWomen. <http://www.peacewomen.org/WPS-Financing>

Peace Women. (2018, March 7). UN Resolution 1325: Significant But Lacking. PeaceWomen. <http://peacewomen.org/resource/un-resolution-1325-significant-lacking>

Perrin, N., Marsh, M., Clough, A., Desgroppes, A., Yope Phanuel, C., Abdi, A., Kaburu, F., Heitmann, S., Yamashina, M., Ross, B., Read-Hamilton, S., Turner, R., Heise, L., & Glass, N. (2019). Social norms and beliefs about gender based violence scale: A measure for use with gender based violence prevention programs in low-resource and humanitarian settings. Conflict and Health, 13(1), 6. <https://doi.org/10.1186/s13031-019-0189-x>

Peters, J. L., Sutton, A. J., Jones, D. R., Abrams, K. R., & Rushton, L. (2008). Contour-enhanced meta-analysis funnel plots help distinguish publication bias from other causes of asymmetry. Journal of Clinical Epidemiology, 61(10), 991–996. <https://doi.org/10.1016/j.jclinepi.2007.11.010>

Picon, M. G., Rankin, K., International Initiative for Impact Evaluation (3ie), Ludwig, J., International Initiative for Impact Evaluation (3ie), Sabet, S. M., International Initiative for Impact Evaluation (3ie), Delaney, A., Independent consultant, Holst, A., & Independent consultant. (2017). Intimate partner violence prevention: An evidence gap map (2017th ed.). International Initiative for Impact Evaluation. <https://doi.org/10.23846/EGM008>

Pluye et al. (2009). Proposal: A mixed methods appraisal tool for systematic mixed studies reviews. 37th NAPCRG Annual Conference. <https://www.webcitation.org/5tTRTc9yJ>

Polanin, J. R., Tanner-Smith, E. E., & Hennessy, E. A. (2016). Estimating the Difference Between Published and Unpublished Effect Sizes: A Meta-Review. Review of Educational Research, 86(1), 207–236. <https://doi.org/10.3102/0034654315582067>

Purgato, M., Gastaldon, C., Papola, D., van Ommeren, M., Barbui, C., & Tol, W. A. (2018). Psychological therapies for the treatment of mental disorders in low- and middle-income countries affected by humanitarian crises. The Cochrane Database of Systematic Reviews, 7, CD011849. <https://doi.org/10.1002/14651858.CD011849.pub2>

Purgato, M., Gross, A. L., Betancourt, T., Bolton, P., Bonetto, C., Gastaldon, C., Gordon, J., O’Callaghan, P., Papola, D., Peltonen, K., Punamaki, R.-L., Richards, J., Staples, J. K., Unterhitzenberger, J., van Ommeren, M., de Jong, J., Jordans, M. J. D., Tol, W. A., & Barbui, C. (2018). Focused psychosocial interventions for children in low-resource humanitarian settings: A systematic review and individual participant data meta-analysis. The Lancet. Global Health, 6(4), e390–e400. <https://doi.org/10.1016/S2214-109X(18)30046-9>

R Core Team. (2020). R: What is R? <https://www.r-project.org/about.html>

R Project. (n.d.). R: The R Project for Statistical Computing. Retrieved January 8, 2021, from <https://www.r-project.org/>

Red nacional de mujeres. (n.d.). Coalición 1325—Coalición 1325. Retrieved November 27, 2020, from <https://www.rednacionaldemujeres.org/index.php/publicaciones/coalicion-1325>

Rihoux, B. (2006). Qualitative Comparative Analysis (QCA) and Related Systematic Comparative Methods. International Sociology - INT SOCIOL, 21, 679–706. <https://doi.org/10.1177/0268580906067836>

Rivas, C., Ramsay, J., Sadowski, L., Davidson, L. L., Dunne, D., Eldridge, S., Hegarty, K., Taft, A., & Feder, G. (2015). Advocacy interventions to reduce or eliminate violence and promote the physical and psychosocial well-being of women who experience intimate partner abuse. The Cochrane Database of Systematic Reviews, 12, CD005043. <https://doi.org/10.1002/14651858.CD005043.pub3>

Rue, L. D. L., Polanin, J. R., Espelage, D. L., & Pigott, T. D. (2013). PROTOCOL: School-based Interventions to Reduce Dating and Sexual Violence: A Systematic Review. Campbell Systematic Reviews, 9(1), 1–43. <https://doi.org/10.1002/CL2.106>

Ruta pacifica de las mujeres. (2016). Plan Estrategico 2016—2020. Ruta pacifica de las mujeres. <https://rutapacifica.org.co/wp/wp-content/uploads/2018/06/PlanEstrategico2016_2020.pdf>

Sánchez-Meca, J., Marín-Martínez, F., & Chacón-Moscoso, S. (2003). Effect-size indices for dichotomized outcomes in meta-analysis. Psychological Methods, 8(4), 448–467. <https://doi.org/10.1037/1082-989X.8.4.448>

Santesso, N., Carrasco-Labra, A., Langendam, M., Brignardello-Petersen, R., Mustafa, R. A., Heus, P., Lasserson, T., Opiyo, N., Kunnamo, I., Sinclair, D., Garner, P., Treweek, S., Tovey, D., Akl, E. A., Tugwell, P., Brozek, J. L., Guyatt, G., & Schunemann, H. J. (2016). Improving GRADE evidence tables part 3: Detailed guidance for explanatory footnotes supports creating and understanding GRADE certainty in the evidence judgments. Journal of Clinical Epidemiology, 74, 28–39. <https://doi.org/10.1016/j.jclinepi.2015.12.006>

Santesso, N., Glenton, C., Dahm, P., Garner, P., Akl, E. A., Alper, B., Brignardello-Petersen, R., Carrasco-Labra, A., De Beer, H., Hultcrantz, M., Kuijpers, T., Meerpohl, J., Morgan, R., Mustafa, R., Skoetz, N., Sultan, S., Wiysonge, C., Guyatt, G., & Schünemann, H. J. (2020). GRADE guidelines 26: Informative statements to communicate the findings of systematic reviews of interventions. Journal of Clinical Epidemiology, 119, 126–135. <https://doi.org/10.1016/j.jclinepi.2019.10.014>

Senarathna, T. (2015). Women in peace processes—Real inclusivity or &quot;just add women&quot;? <https://www.academia.edu/32694680/Women_in_peace_processes_real_inclusivity_or_just_add_women_>

Shemilt, I., Mcdaid, D., Marsh, K., Henderson, C., Bertranou, E., Mallender, J., Drummond, M., Mugford, M., & Vale, L. (2013). Issues in the incorporation of economic perspectives and evidence into Cochrane reviews. Systematic Reviews, 2, 83. <https://doi.org/10.1186/2046-4053-2-83>

SIDA. (2015). Gender Toolbox Brief: Women, Peace and Security (Gender Toolbox, p. 4) [Brief]. SIDA. <https://www.sida.se/contentassets/3a820dbd152f4fca98bacde8a8101e15/women-peace-and-security.pdf>

Signorelli, M. C., Hillel, S., de Oliveira, D. C., Ayala Quintanilla, B. P., Hegarty, K., & Taft, A. (2018). Voices from low-income and middle income countries: A systematic review protocol of primary healthcare interventions within public health systems addressing intimate partner violence against women. <https://doi.org/10.1136/bmjopen-2017-019266>

Sigsworth, R., & Kumalo, L. (2016). Women, peace and security: Implementing the Maputo Protocol in Africa. 24.

Snilstveit, B. (2012). Systematic reviews: From ‘bare bones’ reviews to policy relevance. Journal of Development Effectiveness, 4(3), 388–408. <https://doi.org/10.1080/19439342.2012.709875>

Snilstveit, B., & al. (2015). Interventions for improving learning outcomes and access to education in low- and middleincome countries (No. 24; Systematic Review 24, p. 891). 3ie. <https://www.3ieimpact.org/sites/default/files/2019-01/SR24-education-review_2.pdf>

Sonnenfeld, A., Chirgwin, H., International Initiative for Impact Evaluation (3ie), Berretta, M., International Initiative for Impact Evaluation (3ie), Longman, K., Independent consultant, Krämer, M., German Institute for Development Evaluation (DEval), Snilstveit, B., & International Initiative for Impact Evaluation (3ie). (2020). Building peaceful societies: An evidence gap map (2020th ed.). International Initiative for Impact Evaluation (3ie). <https://doi.org/10.23846/EGM015>

Spangaro, J., Adogu, C., Ranmuthugala, G., Davies, G. P., Steinacker, L., & Zwi, A. (2013). What Evidence Exists for Initiatives to Reduce Risk and Incidence of Sexual Violence in Armed Conflict and Other Humanitarian Crises? A Systematic Review. PLOS ONE, 8(5), e62600. <https://doi.org/10.1371/journal.pone.0062600>

Spangaro, J., Zwi, A. B., Adogu, C., Ranmuthugala, G., Davies, G. P., Steinacker, L., University of London, Social Science Research Unit, & Evidence for Policy and Practice Information and Co-ordinating Centre. (2013). What is the evidence of the impact of initiatives to reduce risk and incidence of sexual violence in conflict and post-conflict zones and other humanitarian crises in lower- and middle-income countries?: A systematic review.

Speake, B. (2013, February 11). A Gendered Approach to Peacebuilding and Conflict Resolution. E-International Relations. <https://www.e-ir.info/2013/02/11/a-gendered-approach-to-peacebuilding-and-conflict-resolution/>

Sterne, J. A., Hernán, M. A., Reeves, B. C., Savović, J., Berkman, N. D., Viswanathan, M., Henry, D., Altman, D. G., Ansari, M. T., Boutron, I., Carpenter, J. R., Chan, A.-W., Churchill, R., Deeks, J. J., Hróbjartsson, A., Kirkham, J., Jüni, P., Loke, Y. K., Pigott, T. D., … Higgins, J. P. (2016). ROBINS-I: A tool for assessing risk of bias in non-randomised studies of interventions. BMJ, 355, i4919. <https://doi.org/10.1136/bmj.i4919>

Sterne, J. A. C., Sutton, A. J., Ioannidis, J. P. A., Terrin, N., Jones, D. R., Lau, J., Carpenter, J., Rücker, G., Harbord, R. M., Schmid, C. H., Tetzlaff, J., Deeks, J. J., Peters, J., Macaskill, P., Schwarzer, G., Duval, S., Altman, D. G., Moher, D., & Higgins, J. P. T. (2011). Recommendations for examining and interpreting funnel plot asymmetry in meta-analyses of randomised controlled trials. BMJ, 343, d4002. <https://doi.org/10.1136/bmj.d4002>

Strickland, R. (2004). To Have and To Hold: Women’s Property and Inheritance Rights in the Context of HIV/AIDS in Sub-Saharan Africa. 84.

Sweetman, C., & Rowlands, J. (2016). Introduction: Working on gender equality in fragile contexts. Gender & Development, 24(3), 337–351. <https://doi.org/10.1080/13552074.2016.1248033>

Tabachnick, B., & Fidell, Linda. S. (2007). Using Multivarite Statistics. In Boston: Allyn & Bacon (Vol. 3).

Thomas, J., & Brunton, J. (2010). EPPI-Reviewer 4: Software for Research Synthesis.

Thomas, J., & Harden, A. (2008a). Methods for the thematic synthesis of qualitative research in systematic reviews. BMC Medical Research Methodology, 8(1), 45. <https://doi.org/10.1186/1471-2288-8-45>

Thomas, J., & Harden, A. (2008b). Methods for the thematic synthesis of qualitative research in systematic reviews. BMC Medical Research Methodology, 8(1), 45. <https://doi.org/10.1186/1471-2288-8-45>

Thomas, J., McNaught, J., & Ananiadou, S. (2011). Applications of text mining within systematic reviews. Research Synthesis Methods, 2(1), 1–14. <https://doi.org/10.1002/jrsm.27>

Thomas, J., O’Mara-Eves, A., & Brunton, G. (2014). Using qualitative comparative analysis (QCA) in systematic reviews of complex interventions: A worked example. Systematic Reviews, 3(1), 67. <https://doi.org/10.1186/2046-4053-3-67>

Tian, N., Fleurant, A., Kuimova, A., Wezeman, P., & Wezeman, S. (2019). Trends in world military expenditure, 2018. 12.

Tol, W. A., Stavrou, V., Greene, M. C., Mergenthaler, C., van Ommeren, M., & García Moreno, C. (2013a). Sexual and gender-based violence in areas of armed conflict: A systematic review of mental health and psychosocial support interventions. Conflict and Health, 7(1), 16. <https://doi.org/10.1186/1752-1505-7-16>

Tol, W. A., Stavrou, V., Greene, M. C., Mergenthaler, C., van Ommeren, M., & García Moreno, C. (2013b). Sexual and gender-based violence in areas of armed conflict: A systematic review of mental health and psychosocial support interventions. Conflict and Health, 7(1), 16. <https://doi.org/10.1186/1752-1505-7-16>

Tyrer, R. A., & Fazel, M. (2014). School and Community-Based Interventions for Refugee and Asylum Seeking Children: A Systematic Review. PLOS ONE, 9(2), e89359. <https://doi.org/10.1371/journal.pone.0089359>

UN Development Programme. (2019). Parliament as partners supporting women peace and security agenda. UNDP. <https://www.undp.org/content/undp/en/home/librarypage/crisis-prevention-and-recovery/parliament-as-partners-supporting-women-peace-and-security-agend.html>

UN Women. (n.d.-a). Thematic Area Dashboard | UN Women Data Hub. Retrieved November 27, 2020, from <https://data.unwomen.org/data-portal/vaw-wps>

UN Women. (n.d.-b). Thematic Area Dashboard | UN Women Data Hub. Retrieved November 27, 2020, from <https://data.unwomen.org/data-portal/vaw-wps>

UN Women. (2015). Preventing Conflict, transforming justice, securing the peace.

UN Women. (2020). Facts and figures: Women, peace, and security | What we do. UN Women. <https://www.unwomen.org/en/what-we-do/peace-and-security/facts-and-figures>

UNICEF (Ed.). (2006). Women and children: The double dividend of gender equality. UNICEF.

United Nations (Ed.). (2010). Achieving gender equality, women’s empowerment and strengthening development cooperation: Dialogues at the Economic and Social Council. United Nations.

United Nations Human Rights Office of the High Commissioner. (2014). Women’s rights are human rights. United Nations. <https://www.ohchr.org/documents/events/whrd/womenrightsarehr.pdf>

United Nations Peacebuilding Support Office. (2010). UN Peacebuilding: An Orientation. United Nations. <https://www.un.org/peacebuilding/sites/www.un.org.peacebuilding/files/documents/peacebuilding_orientation.pdf>

United Nations Security Council. (2000). Resolution 1325 (2000) [Resolution]. <https://undocs.org/pdf?symbol=en/S/RES/1325(2000)>

United Nations Security Council. (2016). Resolution 2282 (2016) [Resolution]. <https://documents-dds-ny.un.org/doc/UNDOC/GEN/N16/118/51/PDF/N1611851.pdf?OpenElement>

United Nations Security Council. (2018). Resolution 2419 (2018) [Resolution]. <https://undocs.org/pdf?symbol=en/S/RES/2419(2018)>

United States Institute for Peace. (2012). Gender, War, and Peacebuilding. United States Institute of Peace. <https://www.usip.org/publications/2012/09/gender-war-and-peacebuilding>

UNOCHA. (2009). Human Security in Theory and Practice: Application of the Human Security Concept and the United Nations Trust Fund for Human Security. <https://www.unocha.org/sites/dms/HSU/Publications%20and%20Products/Human%20Security%20Tools/Human%20Security%20in%20Theory%20and%20Practice%20English.pdf>

USAID. (2007). Women and Conflict: An Introductury guide for programming (p. 29). USAID. [https://www.usaid.gov/sites/default/files/documents/1865/toolkit_women_and_
conflict_an_introductory_guide_for_programming.pdf](https://www.usaid.gov/sites/default/files/documents/1865/toolkit_women_and_conflict_an_introductory_guide_for_programming.pdf)

Valentine, J. C., Aloe, A. M., & Lau, T. S. (2015). Life After NHST: How to Describe Your Data Without “p-ing” Everywhere. Basic and Applied Social Psychology, 37(5), 260–273. <https://doi.org/10.1080/01973533.2015.1060240>

Viechtbauer, W. (2010). Conducting Meta-Analyses in R with the metafor Package. Journal of Statistical Software, 36(1), 1–48. <https://doi.org/10.18637/jss.v036.i03>

Viechtbauer, W., & Cheung, M. W.-L. (2010). Outlier and influence diagnostics for meta-analysis. Research Synthesis Methods, 1(2), 112–125. <https://doi.org/10.1002/jrsm.11>

Waddington, H., Aloe, A. M., Becker, B. J., Djimeu, E. W., Hombrados, J. G., Tugwell, P., Wells, G., & Reeves, B. (2017). Quasi-experimental study designs series—paper 6: Risk of bias assessment. Journal of Clinical Epidemiology, 89, 43–52. <https://doi.org/10.1016/j.jclinepi.2017.02.015>

Waddington, H., Stevenson, J., Sonnenfeld, A., & Gaarder, M. (2018). PROTOCOL: Participation, inclusion, transparency and accountability (PITA) to improve public services in low- and middle-income countries: a systematic review. Campbell Systematic Reviews, 14(1), 1–69. <https://doi.org/10.1002/CL2.205>

Waddington, H., White, H., Snilstveit, B., Hombrados, J. G., Vojtkova, M., Davies, P., Bhavsar, A., Eyers, J., Koehlmoos, T. P., Petticrew, M., Valentine, J. C., & Tugwell, P. (2012a). How to do a good systematic review of effects in international development: A tool kit. Journal of Development Effectiveness, 4(3), 359–387. <https://doi.org/10.1080/19439342.2012.711765>

Waddington, H., White, H., Snilstveit, B., Hombrados, J. G., Vojtkova, M., Davies, P., Bhavsar, A., Eyers, J., Koehlmoos, T. P., Petticrew, M., Valentine, J. C., & Tugwell, P. (2012b). How to do a good systematic review of effects in international development: A tool kit. Journal of Development Effectiveness, 4(3), 359–387. <https://doi.org/10.1080/19439342.2012.711765>

Waddington, H., White, H., Snilstveit, B., Hombrados, J. G., Vojtkova, M., Davies, P., Bhavsar, A., Eyers, J., Koehlmoos, T. P., Petticrew, M., Valentine, J. C., & Tugwell, P. (2012c). How to do a good systematic review of effects in international development: A tool kit. Journal of Development Effectiveness, 4(3), 359–387. <https://doi.org/10.1080/19439342.2012.711765>

Warren, E., Post, N., Hossain, M., Blanchet, K., & Roberts, B. (2015). Systematic review of the evidence on the effectiveness of sexual and reproductive health interventions in humanitarian crises. BMJ Open, 5, e008226. <https://doi.org/10.1136/bmjopen-2015-008226>

WebCite query result. (n.d.). Retrieved June 21, 2021, from <https://www.webcitation.org/5tTRTc9yJ>

White, H. (2009). Theory-based impact evaluation: Principles and practice. Journal of Development Effectiveness, 1(3), 271–284. <https://doi.org/10.1080/19439340903114628>

Wilson, D. B., Weisburd, D., & McClure, D. (2011). Use of DNA testing in police investigative work for increasing offender identification, arrest, conviction and case clearance. Campbell Systematic Reviews, 7(1), 1–53. <https://doi.org/10.4073/csr.2011.7>

World Bank. (2020). Classification of Fragile and Conflict-Affected Situations. World Bank. <https://www.worldbank.org/en/topic/fragilityconflictviolence/brief/harmonized-list-of-fragile-situations>

World Health Organisation. (n.d.). WHO Gender Responsive Assessment Scale: Criteria for assessing programmes and policies. World Health Organisation. Retrieved November 26, 2020, from <https://www.who.int/gender/mainstreaming/GMH_Participant_GenderAssessmentScale.pdf>

### Analysis and reporting

Amaral, S., Bhalotra, S., and Prakash, N. (2019). *Gender, Crime and Punishment: Evidence From Women Police Stations In India*. 63.

BMZ, F. M. for E. C. and D. (n.d.-a). *Further Information On Peace And Security*. Federal Ministry for Economic Cooperation and Development. Retrieved November 6, 2020, from<http://www.bmz.de/en/issues/state_fragility/dokumente/index.html>

BMZ, F. M. for E. C. and D. (n.d.-b). *German And International Activities In Fragile States*. Federal Ministry for Economic Cooperation and Development. Retrieved November 6, 2020, from<http://www.bmz.de/en/issues/state_fragility/deutschesengagement/index.html>

BMZ, F. M. for E. C. and D. (n.d.-c). *State Fragility – A Challenge For Development Policy*. Federal Ministry for Economic Cooperation and Development. Retrieved November 6, 2020, from<http://www.bmz.de/en/issues/state_fragility/index.html>

Bouta, T., Frerks, G., and Bannon, I. (2004). *Gender, Conflict, And Development*. The World Bank.<https://doi.org/10.1596/0-8213-5968-1>

*Brief_33_Gender_in_conflict.pdf*. (n.d.). Retrieved November 5, 2020, from<https://www.iss.europa.eu/sites/default/files/EUISSFiles/Brief_33_Gender_in_conflict.pdf>

CDA Collaborative Learning. (2019). *Sub-sector Review Of Evidecne From Reconciliation Programs* (p. 34). Peacebuilding Evaluation Consortium.<https://www.cdacollaborative.org/wp-content/uploads/2019/08/PEC-Reconciliation-Sector-Review-Final-March-2019-2.pdf>

*Classification Of Fragile And Conflict-affected Situations*. (n.d.). World Bank. Retrieved November 6, 2020, from [https://www.worldbank.org/en/topic/fragilityconflictviolence/
brief/harmonized-list-of-fragile-situations](%20https://www.worldbank.org/en/topic/fragilityconflictviolence/brief/harmonized-list-of-fragile-situations)

Cockburn, C., & Žarkov, D. (2002). The postwar moment: Militaries, masculinities and international peacekeeping. London: Lawrence & Wishart. <https://repository.ubn.ru.nl/handle/2066/62653>

Cohn, C. (2017). Beyond the “Women, Peace and Security” Agenda: Why We Need a Feminist Roadmap for Sustainable Peace (p. 18). Consortium on Gender, Security and Human Rights. <https://genderandsecurity.org/sites/default/files/Cohn_-_Beyond_the_Women_Peace_and_Security_Agenda_Why_We_Need_a_Feminist_Roadmap_for_Sustainable_Peace.pdf>

Das, N., Yasmin, R., Ara, J., Kamruzzaman, M., Davis, P., Behrman, J., Roy, S., and Quisumbing, A. R. (2013). *How Do Intrahousehold Dynamics Change When Assets Are Transferred To Women? Evidence From Brac’s Challenging The Frontiers Of Poverty Reduction — Targeting The Ultra Poor Program In Bangladesh* (SSRN Scholarly Paper ID 2405712). Social Science Research Network.<https://doi.org/10.2139/ssrn.2405712>

Di Pierro, J. (2021). CAPACITY BUILDING: Is It Only A Matter Of Training? *Lear*.<https://www.learlab.com/insights/capacity-building-is-it-only-a-matter-of-training/>

Dudwick, N., and Kuehnast, K. (n.d.). *Gender and Fragility: Ensuring a Golden Hour*. 8.

*FCSList-FY06toFY20.pdf*. (n.d.). Retrieved November 6, 2020, from<http://pubdocs.worldbank.org/en/176001594407411053/FCSList-FY06toFY20.pdf>

*FINAL States of Fragility Highlights document.pdf*. (n.d.). Retrieved November 6, 2020, from<http://www.oecd.org/dac/conflict-fragility-resilience/docs/FINAL%20States%20of%20Fragility%20Highlights%20document.pdf>

*Fragility Index for a Differentiated Approach*. (n.d.). 16.

*Gender Development Index (GDI) | Human Development Reports*. (n.d.). Retrieved November 6, 2020, from<http://hdr.undp.org/en/content/gender-development-index-gdi>

*Gender In Fragile And Conflict-affected Environments*. (n.d.). GSDRC. Retrieved November 5, 2020, from<https://gsdrc.org/topic-guides/gender/gender-in-fragile-and-conflict-affected-environments/>

Gholami, M., Pakdaman, A., Montazeri, A., and Virtanen, J. I. (2017). Evaluation Of The Impact Of A Mass Media Campaign On Periodontal Knowledge Among Iranian Adults: A Three-month Follow-up. *PloS One*, *12*(1), e0169668.<https://doi.org/10.1371/journal.pone.0169668>

*Global indexes*. (n.d.). Vision Of Humanity. Retrieved November 6, 2020, from<https://www.visionofhumanity.org/maps/>

Goetz, A. M. (2018, March 13). What Does The New Women, Peace, And Security Index Measure? *IPI Global Observatory*.<https://theglobalobservatory.org/2018/03/what-does-wps-index-measure/>

*GPI_2020_web.pdf*. (n.d.). Retrieved November 6, 2020, from<https://visionofhumanity.org/wp-content/uploads/2020/10/GPI_2020_web.pdf>

*Hdr2019_technical_notes.pdf*. (n.d.). Retrieved November 6, 2020, from<http://hdr.undp.org/sites/default/files/hdr2019_technical_notes.pdf>

Hemsteede, R. (2018). *Conditional Or Unconditional Cash Transfers? From Ideology To Policy Dialogue*. Socialprotection.Org.<https://socialprotection.org/discover/blog/conditional-or-unconditional-cash-transfers-ideology-policy-dialogue>

IDEA. (2021). *Gender Quotas | International IDEA*.<https://www.idea.int/data-tools/data/gender-quotas/quotas#what>

Khasnabis, C., Motsch, K. H., Achu, K., Jubah, K. A., Brodtkorb, S., Chervin, P., Coleridge, P., Davies, M., Deepak, S., Eklindh, K., Goerdt, A., Greer, C., Heinicke-Motsch, K., Hooper, D., Ilagan, V. B., Jessup, N., Khasnabis, C., Mulligan, D., Murray, B., … Lander, T. (2010). Self-help groups. In *Community-Based Rehabilitation: CBR Guidelines*. World Health Organization.<https://www.ncbi.nlm.nih.gov/books/NBK310972/>

Klugman, J., Nagel, R., & Viollaz, M. (2021). *Women’s empowerment as a path to peace*. 23.

Miller, C., Tsoka, M., Boyd-Boffa, M., Msiska, K., Brooks, M., Mtonga, D., Chambers, E., Cunningham, E., Mwanza, M., Henninger, N., Chimwaza, D., and Khonje, D. (2008). *Impact Evaluation Report External Evaluation of the Mchinji Social Cash Transfer Pilot*.

*OECD Highlights documents_web.pdf*. (n.d.). Retrieved November 6, 2020, from<https://www.oecd.org/dac/conflict-fragility-resilience/docs/OECD%20Highlights%20documents_web.pdf>

Perova, E., and Reynolds, S. A. (2017). Women’s Police Stations And Intimate Partner Violence: Evidence From Brazil. *Social Science and Medicine (1982)*, *174*, 188–196.<https://doi.org/10.1016/j.socscimed.2016.12.008>

Pompa, C. (2014). *TVET and skills training in fragile and conflict affected countries*. 28.

Search for Common Ground. (2016). *Community Dialogue Design Manual*. Common Ground Institute.<https://www.sfcg.org/wp-content/uploads/2017/07/CGI-Anglais-interactive.pdf>

*States of Fragility 2015: Meeting Post-2015 Ambitions | en | OECD*. (n.d.). Retrieved November 6, 2020, from<http://www.oecd.org/dac/states-of-fragility-2015-9789264227699-en.htm>

*Strickland, R. (n.d.). Gender Equity and Peacebuilding: From Rhetoric to Reality. 48.*

Sweetman, C., and Rowlands, J. (2016). Introduction: Working On Gender Equality In Fragile Contexts. *Gender and Development*, *24*(3), 337–351.<https://doi.org/10.1080/13552074.2016.1248033>

UNFPA. (2015). *UNFPA Women and Girls Safe Spaces Guidance*. [https://www.unfpa.org/sites/default/files/resource-pdf/UNFPA%20UNFPA%20Women
%20and%20Girls%20Safe%20Spaces%20Guidance%20%5B1%5D.pdf](%20https://www.unfpa.org/sites/default/files/resource-pdf/UNFPA%20UNFPA%20Women%20and%20Girls%20Safe%20Spaces%20Guidance%20%5B1%5D.pdf)

VSL Associates. (2021). *The VSLA Methodology*. VSL Associates.<https://www.vsla.net/the-vsla-methodology/>

White, H., Menon, R., and Waddington, H. (2018). *Community-driven development: Does it build social cohesion or infrastructure? A mixed-method evidence synthesis*. *1*.

*Women, peace and security index 2019/20 inclusion, justice and security.* (2019). Georgetown Institute for Women, Peace and Security (GIWPS).

Women, Peace, and Security Index. (n.d.). *Georgetown Institute of Women Peace and Security*. Retrieved November 6, 2020, from<https://giwps.georgetown.edu/the-index/>

World Bank. (2021a). *Community-Driven Development* [Text/HTML]. World Bank.<https://www.worldbank.org/en/topic/communitydrivendevelopment>

World Bank. (2021b). *Microfinance And Economic Development* [Text/HTML]. World Bank.<https://documents.worldbank.org/en/publication/documents-reports/documentdetail/107171511360386561/Microfinance-and-economic-development>

Zuckerman, E., & Greenberg, M. (2004). The gender dimensions of post-conflict reconstruction: An analytical framework for policymakers. Gender & Development, 12(3), 70–82. <https://doi.org/10.1080/13552070412331332330>

### Other systematic reviews

*A Review of the Evidence Developed for a Technical Consultation on Expanding Access to Injectable Contraception*. (n.d.). 51.

*A Review Of The Evidence Developed For A Technical.pdf*. (n.d.). Retrieved June 21, 2021, from<https://www.fhi360.org/sites/default/files/media/documents/A%20Review%20of%20the%20Evidence%20Developed%20for%20a%20Technical%20Consultation%20on%20Expanding%20Access%20to%20Injectable%20Contraception.pdf>

Arango, D. J., Morton, M., Gennari, F., Kiplesund, S., and Ellsberg, M. (n.d.). *Interventions To Prevent Or Reduce Violence Against Women And Girls: A Systematic Review Of Reviews*. 61.

*Arango et al. - Interventions to Prevent or Reduce Violence Again.pdf*. (n.d.). Retrieved June 21, 2021, from<https://openknowledge.worldbank.org/bitstream/handle/10986/21035/927130NWP0Wome00Box385382B00PUBLIC0.pdf?sequence=1>

Baird, S., Ferreira, F. H. G., Özler, B., and Woolcock, M. (2013). Relative Effectiveness Of Conditional And Unconditional Cash Transfers For Schooling Outcomes In Developing Countries: A Systematic Review. *Campbell Systematic Reviews*, *9*(1), 1–124.<https://doi.org/10.4073/csr.2013.8>

Brody, C., Hoop, T. de, Vojtkova, M., Warnock, R., Dunbar, M., Murthy, P., and Dworkin, S. L. (2017). Can Self-help Group Programs Improve Women’s Empowerment? A Systematic Review. *Journal of Development Effectiveness*, *9*(1), 15–40.<https://doi.org/10.1080/19439342.2016.1206607>

De Koker, P., Mathews, C., Zuch, M., Bastien, S., and Mason-Jones, A. J. (2014). A Systematic Review Of Interventions For Preventing Adolescent Intimate Partner Violence. *Journal of Adolescent Health*, *54*(1), 3–13.<https://doi.org/10.1016/j.jadohealth.2013.08.008>

Eggers del Campo, I., and Steinert, J. I. (2020). The Effect Of Female Economic Empowerment Interventions On The Risk Of Intimate Partner Violence: A Systematic Review And Meta-analysis. *Trauma, Violence, and Abuse*, 1524838020976088.<https://doi.org/10.1177/1524838020976088>

Emezi, Y., & Images, G. (2021). *Governance, Crime and Conflict Initiative Evidence Wrap-Up*. 109.

LaCroix, J. M., Snyder, L. B., Huedo-Medina, T. B., and Johnson, B. T. (2014). Effectiveness Of Mass Media Interventions For HIV Prevention, 1986–2013: A Meta-analysis. *JAIDS Journal of Acquired Immune Deficiency Syndromes*, *66*, S329.<https://doi.org/10.1097/QAI.0000000000000230>

Langer, L., Erasmus, Y., Tannous, N., Obuku, E., Ravat, Z., Chisoro, C., Opondo, M., Nduku, P., Tripney, J., van Rooyen, C., and Stewart, R. (2018). Women In Wage Labour: A Systematic Review Of The Effectiveness And Design Features Of Interventions Supporting Women’s Participation In Wage Labour In Higher-growth And/Or Male-dominated Sectors In Low- And Middle-income Countries. In *EPPI-Centre, Social Science Research Unit, UCL Institute of Education: London, UK.* [Report]. EPPI-Centre, Social Science Research Unit, UCL Institute of Education.<https://eppi.ioe.ac.uk/cms/Coursesseminars/Seminarsevents/Womeninwagelabour/tabid/3749/Default.aspx>

*Lassi et al. - 2015—Impact Of Service Provision Platforms On Maternal .pdf*. (n.d.). Retrieved June 21, 2021, from<https://conflictandhealth.biomedcentral.com/track/pdf/10.1186/s13031-015-0054-5.pdf>

Lassi, Z. S., Aftab, W., Ariff, S., Kumar, R., Hussain, I., Musavi, N. B., Memon, Z., Soofi, S. B., and Bhutta, Z. A. (2015). Impact Of Service Provision Platforms On Maternal And Newborn Health In Conflict Areas And Their Acceptability In Pakistan: A Systematic Review. *Conflict and Health*, *9*(1), 25.<https://doi.org/10.1186/s13031-015-0054-5>

Manley, J., Gitter, S., and Slavchevska, V. (2013). How Effective Are Cash Transfers At Improving Nutritional Status? *World Development*, *48*, 133–155.<https://doi.org/10.1016/j.worlddev.2013.03.010>

McQueston, K., Silverman, R., and Glassman, A. (2013). The Efficacy Of Interventions To Reduce Adolescent Childbearing In Low- And Middle-income Countries: A Systematic Review. *Studies in Family Planning*, *44*(4), 369–388.<https://doi.org/10.1111/j.1728-4465.2013.00365.x>

*Providing access to economic assets for girls and young women in low-and-lower middle-income countries: A systematic review of the evidence*. (2012). Social Science Research Unit.

Stark, L., Robinson, M. V., Seff, I., Gillespie, A., Colarelli, J., and Landis, D. (2021). The Effectiveness Of Women And Girls Safe Spaces: A Systematic Review Of Evidence To Address Violence Against Women And Girls In Humanitarian Contexts. *Trauma, Violence, and Abuse*, 1524838021991306.<https://doi.org/10.1177/1524838021991306>

Vaessen, J., Rivas, A., Duvendack, M., Jones, R. P., Leeuw, F., Gils, G. van, Lukach, R., Holvoet, N., Bastiaensen, J., Hombrados, J. G., and Waddington, H. (2014). The Effects Of Microcredit On Women’s Control Over Household Spending In Developing Countries: A Systematic Review And Meta-analysis. *Campbell Systematic Reviews*, *10*(1), 1–205.<https://doi.org/10.4073/csr.2014.8>

White, H., Menon, R., & Waddington, H. (2018). *Community-driven development: Does it build social cohesion or infrastructure?* (p. 52). 3ie. <https://www.3ieimpact.org/sites/default/files/2019-01/wp30-cdd_0.pdf>

**Other publications in the 3ie Systematic Review Series**

The following reviews are available at
<http://www.3ieimpact.org/evidence-hub/publications/systematic-reviews>

*Strengthening intergroup social cohesion in fragile situations*. 3ie Systematic Review 46. Sonnenfeld, A, Doherty, J, Berretta, M, Shisler, S, Snilstveit, B, Eyers, J, Castaman, K, Gupta, R, Anda Leon, MD, Franich, A, Yavuz, C, Baafi, A and Obaid, R, 2021.

*Effects of electricity access interventions on socio-economic outcomes in low- and middle-income countries*. 3ie Systematic Review 45. Moore, N, Glandon, D, Tripney, J, Kozakiewicz, T, Shisler, S, Eyers, J, Zalfou, R, Leon, MDA, Kurkjian, V, Snilstveit, B and Perdana, A. 2020.

*Incentives for climate mitigation in the land use sector – the effects of payment for environmental services (PES) on environmental and socio-economic outcomes in low- and middle-income countries: a mixed-method systematic review.* 3ie Systematic Review 44. Snilstveit, B, Stevenson, J, Langer, L, da Silva, N, Rabat, Z, Nduku, P, Polanin, J, Shemilt, I, Eyers, J, Ferraro, PJ, 2019.

*Does incorporating participation and accountability improve development outcomes? Meta-analysis and framework synthesis.* 3ie Systematic Review 43. Waddington, H, Sonnenfeld, A, Finetti, J, Gaarder, M and Stevenson, J, 2019.

*Impact of financial inclusion in low- and middle-income countries: a systematic review of reviews.* 3ie Systematic Review 42. Duvendack, M and Mader, P, 2019.

*Agricultural input subsidies for improving productivity, farm income, consumer welfare and wider growth in low- and middle-income countries: a systematic review. 3ie Systematic Review 41*. Hemming, DJ, Chirwa, EW, Ruffhead, HJ, Hill, R, Osborn, J, Langer, L, Harman, L, Coffey, C, Dorward, A and Phillips, D, 2018.

*Vocational and business training to improve women’s labour market outcomes in low- and middle-income countries: a systematic review. 3ie Systematic Review 40*. Chinen, M, De Hoop, T, Balarin, M, Alcázar, L, Sennett, J, and Mezarina, J, 2018.

*Interventions to improve the labour market for adults living with physical and/or sensory disabilities in low- and middle-income countries: a systematic review.* 3ie Systematic Review 39. Tripney, J, Roulstone, A, Vigurs, C, Hogrebe, N, Schmidt, E and Stewart, R, 2017.

*The effectiveness of contract farming in improving smallholder income and food security in low- and middle-income countries: a mixed-method systematic review. 3ie Systematic Review 38.* Ton, G, Desiere, S, Vellema, W, Weituschat, S and D’Haese, M (2017)

*Interventions to improve the labour market outcomes of youth: a systematic review of training, entrepreneurship promotion, employment services and subsidized employment interventions. 3ie Systematic Review 37*. Kluve J, Puerto S, Robalino D, Romero JM, Rother F, Stöterau J, Weidenkaff F and Witte M (2017)

*Promoting handwashing and sanitation behaviour change in low- and middle-income countries: a mixed-method systematic review. 3ie Systematic Review 36.* Buck, ED, Remoortel, HV, Hannes, K, Govender, T, Naidoo, S, Avau, B, Veegaete, AV, Musekiwa, A, Lutje, V, Cargo, M, Mosler, HJ, Vandekerckhove, P and Young T (2017)

*Incorporating the life cycle approach into WASH policies and programmes: A systematic review. 3ie Systematic Review 35.* Annamalai, TR, Narayanan, S, Devkar, G, Kumar, VS, Devaraj, R, Ayyangar, A and Mahalingam, A (2017)

*Effects of certification schemes for agricultural production on socio-economic outcomes in low- and middle-income countries: a systematic review 34.* Oya, C, Schaefer, F, Skalidou, D, McCosker, C and Langer, L (2017)

*Short-term WASH interventions in emergency response: a systematic review. 3ie Systematic Review 33*. Yates, T, Allen, J, Joseph, ML and Lantagne, D (2017)

*Community monitoring interventions to curb corruption and increase access and quality of service delivery in low- and middle-income countries. 3ie Systematic Review 32.* Molina E, Carella L, Pacheco A, Cruces, G and Gasparini, L (2016)

*Effects and mechanisms of market-based reforms on access to electricity in developing countries: a systematic review. 3ie Systematic Review 31.* Bensch, G, Sievert, M, Langbein, J, Kneppel, N (2016)

*Youth gang violence and preventative measures in low- and middle-income countries: a systematic review (Part II), 3ie Systematic Review 30.* Higginson, A, Benier, K, Shenderovich, Y, Bedford, L, Mazerolle, L, Murray, J (2016)

*Youth gang membership and violence in low- and middle-income countries: a systematic review (Part I), 3ie Systematic Review 29.* Higginson, A, Benier, K, Shenderovich, Y, Bedford, L, Mazerolle, L, Murray, J (2016)

*Cash-based approaches in humanitarian emergencies: a systematic review, 3ie Systematic Review Report 28.* Doocy, S and Tappis, H (2016)

*Factors affecting uptake of voluntary and community-based health insurance schemes in low-and middle-income countries: a systematic review, 3ie Systematic Review 27.* Panda, P, Dror, IH, Koehlmoos, TP, Hossain, SAS, John, D, Khan, JAM and Dror, DM (2016)

*Parental, community and familial support interventions to improve children’s literacy in developing countries: a systematic review, 3ie Systematic Review 26.* Spier, E, Britto, P, Pigott, T, Roehlkapartain, E, McCarthy, M, Kidron, Y, Song, M, Scales, P, Wagner, D, Lane, J and Glover, J (2016)

*Business support for small and medium enterprises in low- and middle-income countries: a systematic review, 3ie Systematic Review 25.* Piza, C, Cravo, T, Taylor, L, Gonzalez, L, Musse, I, Furtado, I, Sierra, AC and Abdelnour, S (2016)

*Interventions for improving learning outcomes and access to education in low- and middle- income countries: a systematic review, 3ie Systematic Review 24.* Snilstveit, B, Stevenson, J, Phillips, D, Vojtkova, M, Gallagher, E, Schmidt, T, Jobse, H, Geelen, M, Pastorello, M, and Eyers, J (2015)

*Economic self-help group programmes for improving women’s empowerment: a systematic review, 3ie Systematic Review 23*. Brody, C, De Hoop, T, Vojtkova, M, Warnock, R, Dunbar, M, Murthy, P and Dworkin, SL (2016)

*The identification and measurement of health-related spillovers in impact evaluations: a systematic review*, *3ie Systematic Review 22*. Benjamin-Chung, J, Abedin, J, Berger, D, Clark, A, Falcao, L, Jimenez, V, Konagaya, E, Tran, D, Arnold, B, Hubbard, A, Luby, S, Miguel, E and Colford, J (2015)

*The effects of school-based decision-making on educational outcomes in low- and middle-income countries: a systematic review*, 3ie Systematic Review Report 21. Carr-Hill, R, Rolleston, C, Pherali, T and Schendel, R, with Peart, E, and Jones, E (2015)

*Policing interventions for targeting interpersonal violence in developing countries: a systematic review*, *3ie Systematic Review 20*. Higginson, A, Mazerolle, L, Sydes, M, Davis, J, and Mengersen, K (2015)

*The effects of training, innovation and new technology on African smallholder farmers’ wealth and food security: a systematic review*, *3ie Systematic Review 19*. Stewart, R, Langer, L, Rebelo Da Silva N, Muchiri, E, Zaranyika, H, Erasmus, Y, Randall, N, Rafferty, S, Korth, M, Madinga, N and de Wet, T (2015)

*Community based rehabilitation for people with disabilities in low- and middle-income countries: a systematic review*, *3ie Systematic Review 18*. Iemmi, V, Gibson, L, Blanchet, K, Kumar, KS, Rath, S, Hartley, S, Murthy, GVS, Patel, V, Weber, J and Kuper H (2015)

*Payment for environmental services for reducing deforestation and poverty in low- and middle-income countries: a* [*systematic r*](http://www.3ieimpact.org/en/about/3ie-affiliates/3ie-donors/)*eview*, *3ie Systematic Review 17.* Samii, C, Lisiecki, M, Kulkarni, P, Paler, L and Chavis, L (2015)

*Decentralised forest management for reducing deforestation and poverty in low- and middle- income countries: a systematic review*, *3ie Systematic Review 16.* Samii, C, Lisiecki, M, Kulkarni, P, Paler, L and Chavis, L (2015)

*Supplementary feeding for improving the health of disadvantaged infants and young children: a systematic and realist review*, *3ie Systematic Review 15.* Kristjansson, E, Francis, D, Liberato, S, Greenhalgh, T, Welch, V, Jandu, MB, Batal, M, Rader, T, Noonan, E, Janzen, L, Shea, B, Wells, GA and Petticrew, M (2015)

*The impact of land property rights interventions on investment and agricultural productivity in developing countries: a systematic review*, 3ie Systematic Review Report 14. Lawry, S, Samii, C, Hall, R, Leopold, A, Hornby, D and Mtero, F, 2014.

*Slum upgrading strategies and their effects on health and socio-economic outcomes: a systematic review, 3ie Systematic Review 13.* Turley, R, Saith, R., Bhan, N, Rehfuess, E, and Carter, B (2013)

*Services for street-connected children and young people in low- and middle-income countries: a thematic synthesis, 3ie Systematic Review 12.* Coren, E, Hossain, R, Ramsbotham, K, Martin, AJ and Pardo, JP (2014)

*Why targeting matters: examining the relationship between selection, participation and outcomes in farmer field school programmes,* *3ie Systematic Review 11.* Phillips, D, Waddington, H and White, H (2015)

*The impact of export processing zones on employment, wages and labour conditions in developing countries, 3ie Systematic Review 10.* Cirera, X and Lakshman, R (2014)

*Interventions to reduce the prevalence of female genital mutilation/cutting in African countries, 3ie Systematic Review 9.* Berg, RC and Denision, E (2013)

*Behaviour change interventions to prevent HIV among women living in low and middle income countries, 3ie Systematic Review 8.* McCoy, S, Kangwende, RA and Padian, NS (2009)

*The impact of daycare programs on child health, nutrition and development in developing countries, 3ie Systematic Review 7*. Leroy, JL, Gadsden, P and Guijarro, M (2011)

*Willingness to pay for cleaner water in less developed countries: Systematic review of experimental evidence, 3ie Systematic Review 6*. Null, C, Hombrados, JG, Kremer, M, Meeks, R, Miguel, E and Zwane, AP (2012)

*Community-based intervention packages for reducing maternal morbidity and mortality and improving neonatal outcomes, 3ie Systematic Review 5.* Lassi, ZS, Haider, BA and Langou, GD (2011)

The effects of microcredit on women’s control over household spending: a systematic review, 3ie Systematic Review 4. Vaessen, J, Rivas, A, Duvendack, M, Jones, RP, Leeuw, F, van Gils, G, Lukach, R, Holvoet, N, Bastiaensen, J, Hombrados, JG and Waddington, H, (2013).

*Interventions in developing nations for improving primary and secondary school enrolment of children: a systematic review, 3ie Systematic Review 3.* Petrosino, A, Morgan, C, Fronius, T, Tanner-Smith, E, and Boruch, R, 2016.

*Interventions to promote social cohesion in Sub-Saharan Africa, 3ie Systematic Review 2.* King, E, Samii, C and Snilstveit, B (2010)

*Water, sanitation and hygiene interventions to combat childhood diarrhoea in developing countries, 3ie Systematic Review 1.* Waddington, H, Snilstveit, B, White, H and Fewtrell, L (2009**)**
